# Discovery of High-Affinity Cannabinoid Receptors Ligands through a 3D-QSAR Ushered by Scaffold-Hopping Analysis †

**DOI:** 10.3390/molecules23092183

**Published:** 2018-08-30

**Authors:** Giuseppe Floresta, Orapan Apirakkan, Antonio Rescifina, Vincenzo Abbate

**Affiliations:** 1 Department of Drug Sciences, University of Catania, V.le A. Doria, 95125 Catania, Italy; arescifina@unict.it; 2 Department of Chemical Sciences, University of Catania, V.le A. Doria, 95125 Catania, Italy; 3 Institute of Pharmaceutical Science, King’s College London, Stamford Street, London SE1 9NH, UK; 4 King’s Forensics, School of Population Health & Environmental Sciences, King’s College London, Franklin-Wilkins Building, 150 Stamford Street, London SE1 9NH, UK; orapan.apirakkan@kcl.ac.uk

**Keywords:** cannabinoid receptor, CB_1_ and CB_2_, 3D-QSAR, scaffold hopping, virtual screening, Forge and Spark software, bioisosteric replacements

## Abstract

Two 3D quantitative structure–activity relationships (3D-QSAR) models for predicting Cannabinoid receptor 1 and 2 (CB_1_ and CB_2_) ligands have been produced by way of creating a practical tool for the drug-design and optimization of CB_1_ and CB_2_ ligands. A set of 312 molecules have been used to build the model for the CB_1_ receptor, and a set of 187 molecules for the CB_2_ receptor. All of the molecules were recovered from the literature among those possessing measured *K*_i_ values, and Forge was used as software. The present model shows high and robust predictive potential, confirmed by the quality of the statistical analysis, and an adequate descriptive capability. A visual understanding of the hydrophobic, electrostatic, and shaping features highlighting the principal interactions for the CB_1_ and CB_2_ ligands was achieved with the construction of 3D maps. The predictive capabilities of the model were then used for a scaffold-hopping study of two selected compounds, with the generation of a library of new compounds with high affinity for the two receptors. Herein, we report two new 3D-QSAR models that comprehend a large number of chemically different CB_1_ and CB_2_ ligands and well account for the individual ligand affinities. These features will facilitate the recognition of new potent and selective molecules for CB_1_ and CB_2_ receptors.

## 1. Introduction

The receptors of the endocannabinoid system are the cannabinoid receptors. In the human body, this system is involved in different physiological processes, including memory, mood, pain-sensation, and appetite [1]. The cannabinoid receptors are part of the G protein-coupled receptor (GPCR) family, with two known subtypes: CB_1_ and CB_2_ receptors, both expressed in the membrane of the cells [2]. Since the discovery of the ∆^9^-tetrahydrocannabinol (∆^9^-THC), the main psychoactive component of *Cannabis sativa*, these receptors have been studied for their implication in different pathological and physiological conditions [3,4,5,6,7]. Interestingly, the type1 cannabinoid receptor is the most expressed GPCR in the human central and peripheral nervous system and is also expressed throughout the body [8]. CB_1_ selective agonists have shown therapeutic potential in a wide range of disorders, including multiple sclerosis, pain, inflammation, and neurodegenerative disorders [9,10,11]. On the other hand, such ligands also possess the potential of being abused for recreational purposes, and can give rise to mild to severe psychotropic effects. Indeed, in the last decade, a large number of synthetic cannabinoid receptor ligands have appeared on the recreational drug scene [12]. Conversely, the CB_2_ receptors distribution is more limited through the body, particularly high is their expression in the cells of the immune system, and among them with high levels in B lymphocytes and natural killer cells [13]. The exact physiology of CB_2_ receptors is still not completely understood but a number of preclinical studies support the advantage of using CB_2_ ligands for the treatment of different pathophysiological disorders, such as chronic pain, maintenance of bone density, reducing the progression of atherosclerotic lesions, asthma, autoimmune and inflammatory diseases, and multiple sclerosis [14,15]. The distribution of CB_2_ in the central nervous system is limited, therefore CB_2_ selective ligands do not possess significant psychoactive properties. Thus, discovering potent and selective ligands for either CB_1_ or CB_2_ represents an important—and yet only partially met—research goal.

**Figure 1:**
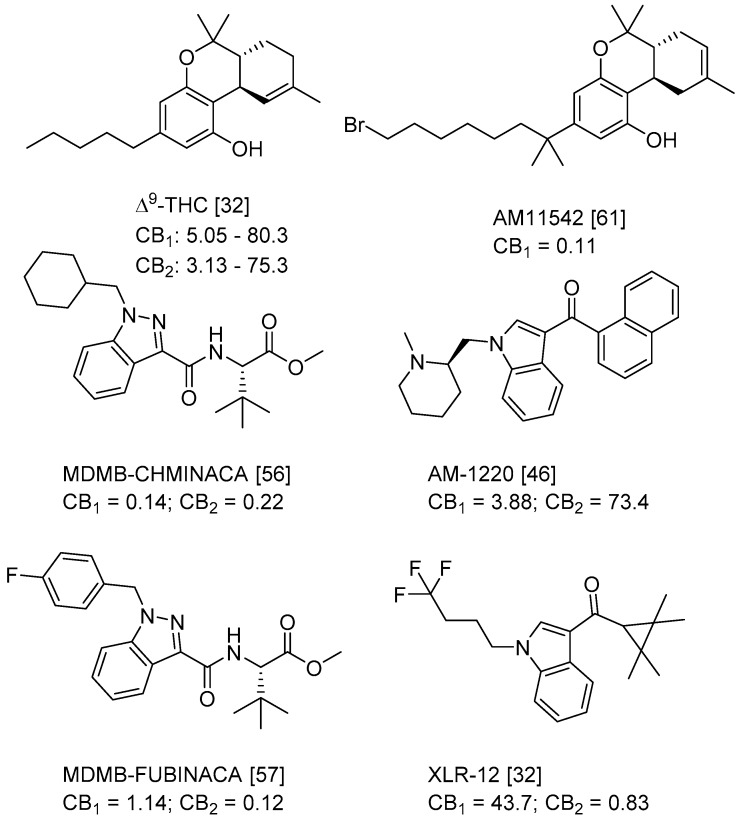
Structure of ∆^9^-THC, AM11542, MDMB-CHMINACA, AM-1220, MDMB-FUBINACA, and XLR-12 along with their *K*_i_ (nM).

QSAR models are used to help predicting or understanding patterns in the chemical, pharmacological/pharmaceutical and biological sciences [16,17,18,19]. There have been several attempts to build QSAR models for the CB_1_ and CB_2_ receptors, but all of them have been produced only using a restricted number of compounds, and only using compounds with similar structure, resulting in good models for restricted classes of cannabinoid receptors binders [20,21,22,23,24,25]. To facilitate the research and investigation among chemical datasets for CB_1_ and CB_2_ ligands capabilities, selectivity, and potency, herein we report the development of two different 3D-QSAR models. The models were created using a set of 312 CB_1_ receptor ligands, and 187 CB_2_ receptor ligands. Details of all the compounds having experimentally determined *K*_i_ values were retrieved from the literature (i.e., anandamide analogues, benzoyl/alkoyl-indoles, cyclohexylphenols, dibenzopyrans, indazole derivatives, indazole-carboxylates, indazole-carboxamides, indole-carboxylates, indole-carboxamides, naphthoyl-benzimidazoles, naphthoyl-indazoles, naphthoyl-indoles, naphthoyl-naphthalenes, naphthoyl-pyrroles and phenylacetyl-indoles) [26,27,28,29,30,31,32,33,34,35,36,37,38,39,40,41,42,43,44,45,46]. Figure 1 details the structures of some example of CB_1_ and CB_2_ ligands. Among them, the natural derived ∆^9^-THC; AM11542, which is the unique tetrahydrocannabinol derivative co-crystallized with the CB_1_ receptor; the two most potent CB_1_ and CB_2_ agonists (MDMB-CHMINACA and MDMB-FUBINACA), and the two most selective compounds for the CB_1_ and CB_2_ receptor (AM-1220 and XLR-12).

Even if there is an interest of the researchers in using the two cannabinoids receptors as pharmacological targets, the majority of the cannabinoids ligands nowadays are known because they are the most common substances used as new psychoactive drugs of abuse; indeed, an elaborate variety of synthetic cannabinoids ligands are designed in an attempt to avoid the legal restrictions on cannabis and spread into the black market (e.g., MDMB-CHMINACA and MDMB-FUBINACA can be defined as synthetic cannabinoids belonging to the a recently emerged superfamily of “New Psychoactive Substances” (NPS) [12]).

Unlike previously published models, the one reported here includes a wider range of chemically different compound (sub)classes. Moreover, the 3D-QSAR models generated, ushered by the scaffold-hopping analysis, have been employed to design different new series of potentially high-affinity and selective ligands of the CB_1_ and CB_2_ receptors. Forge, and Spark software (Cresset) were used for the development of the 3D-QSAR models and for the scaffold-hopping analysis, respectively [47]. Most of the 3D-QSAR methodologies, like CoMFA or CoMSIA, can calculate the molecular properties at the interception points of this 3D grid only after the surrounding the space (with a 3D grid) of the aligned molecules [48,49]. Differently, the software used by us has the ability to calculate the molecular properties only in particular positions that are defined directly from the field points (describing the electrostatic potential and the volume of each molecule, generated by a force field) of the aligned molecules in the training set, yielding exceptional results [50,51,52].

## 2. Results and Discussion

### 2.1. Statistical Analysis and Results

**Figure 2:**
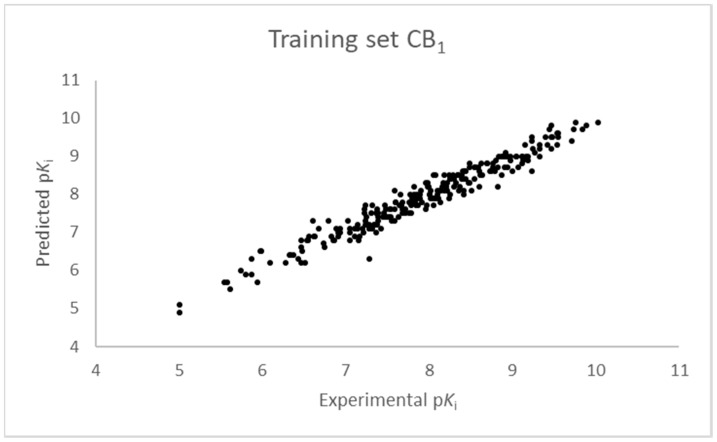
Ten component 3D-QSAR model experimental vs. predicted p*K*_i_ of the compounds in the training set for the CB_1_ receptor.

**Figure 3:**
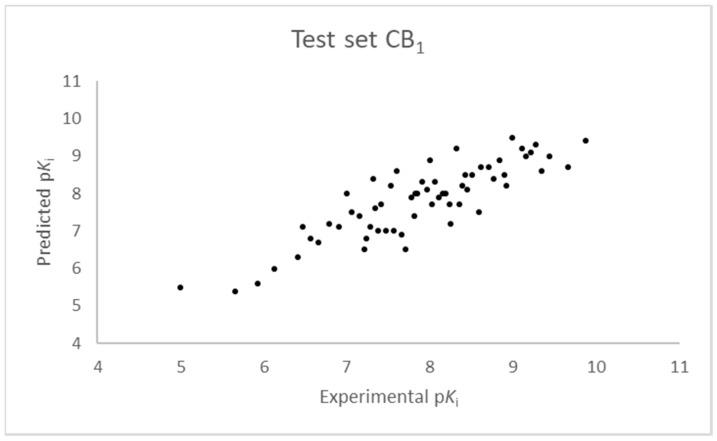
Ten component 3D-QSAR model experimental vs. predicted p*K*_i_ of the compounds in the test set for the CB_1_ receptor.

**Figure 4:**
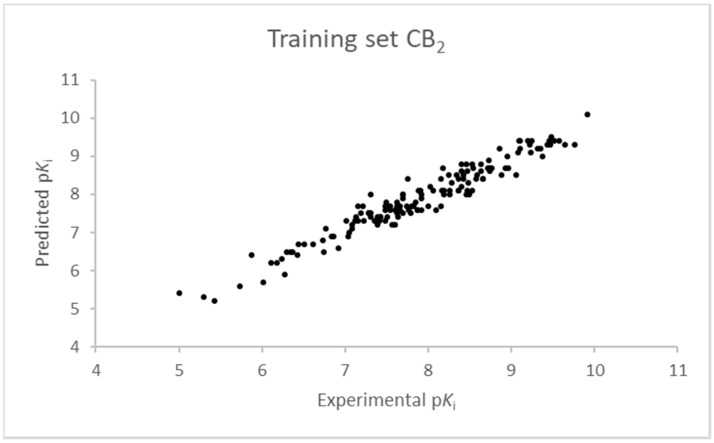
Seven component 3D-QSAR model experimental vs. predicted p*K*_i_ of the compounds in the training set for the CB_2_ receptor.

**Figure 5:**
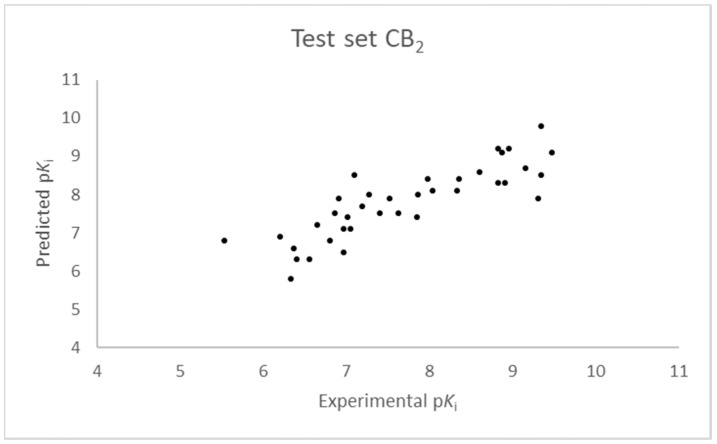
Seven component 3D-QSAR model experimental vs. predicted p*K*_i_ of the compounds in the test set for the CB_2_ receptor.

The SIMPLS algorithm (partial least squares regression method) is used by the software for the calculation of the model [53,54]. More information on the methodology used during the build and the validation of the two models are reported in the Supplementary Materials. The results of the 3D-QSAR models are shown in Figure 2, Figure 3, Figure 4 and Figure 5. The 10-component model for the CB_1_ receptor shows both optimum predictive and descriptive capabilities, demonstrated by the optimum r^2^ (0.95, training set) and q^2^ (0.62, cross-validated training set) values (Figure S5, Supplementary Materials) [55]. In parallel, excellent correlations were achieved by the 7-component model for the CB_2_ receptor, with a r^2^ of 0.93 and a q^2^ of 0.72 (Figure S6, Supplementary Materials). An adequate distribution of the experimental vs. predicted affinities values is shown by the graphs in Figure 2 and Figure 4 for the training set and in Figure 3 and Figure 5 for the test set. In this case, only a few outliers were revealed and excellent cross-validated r^2^ (0.72 and 0.73 for the CB_1_ and the CB_2_ receptor, respectively) are calculated.

**Figure 6:**
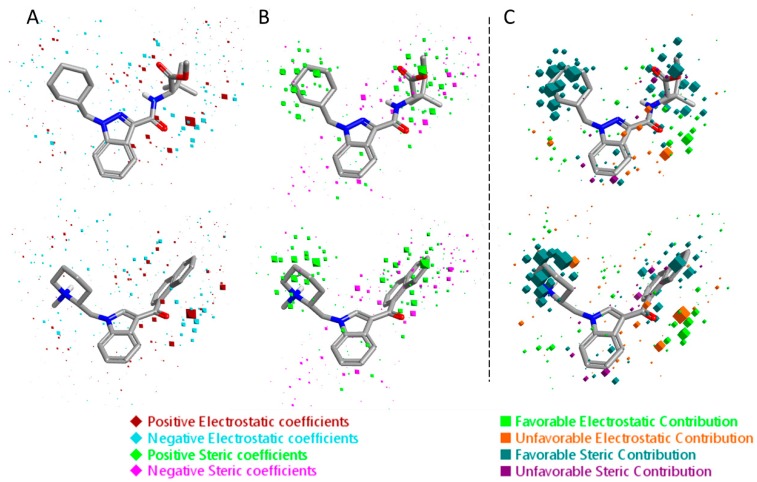
Electrostatic and steric coefficients for the CB_1_ model. (**A**) Electrostatic coefficients superimposed to MDMB-CHMINACA (up) and AM-1220 (down). (**B**) Steric coefficients superimposed to MDMB-CHMINACA (up) and AM-1220 (down). (**C**) Contributions to predicted affinity for MDMB-CHMINACA (up) and AM-1220 (down).

**Figure 7:**
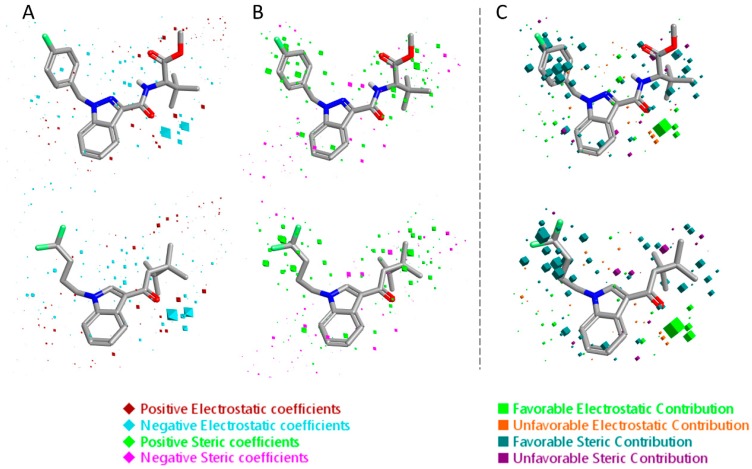
Electrostatic and steric coefficients for the CB_2_ model. (**A**) Electrostatic coefficients superimposed to MDMB-FUBINACA (up) and XLR-12 (down). (**B**) Steric coefficients superimposed to MDMB-FUBINACA (up) and XLR-12 (down). (**C**) Contributions to predicted affinity for MDMB-FUBINACA (up) and XLR-12 (down).

The 3D visualizations of the QSAR models are shown in Figure 6 and Figure 7, where the 3D-QSAR coefficients for the two models are superimposed to the most potent and the most selective compounds for the two cannabinoid receptors. These 3D-QSAR models are characterized by both electrostatic and steric effects, with important differences that encompass the different selectivity of the individual classes of studied compounds toward the CB_1_ and CB_2_ receptors. The model overviews show the areas where the QSAR model indicates that the molecular fields have a strong impact on ligand–receptor affinity. The larger the points (depicted as octahedrons), the stronger the correlation between the fields is (electrostatic and steric) in that position. The higher affinity related to the electrostatic potential is depicted in red for the positive values and in blue for the negative ones. For the steric bulk, the green area leads to higher receptor affinity, whereas the violet area leads to lower affinity.

**Figure 8:**
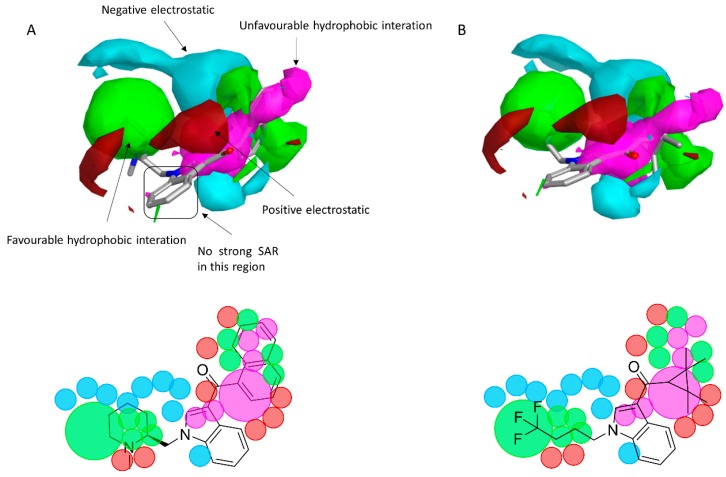
(**A**) The CB_1_ model map superimposed to AM-1220. (**B**) The CB_1_ model map is superimposed to XLR-12. Molecular insight of SAR mechanism models, revealing the different lead optimization sites of active compounds. Red color shows positive field region controlling the activity, and blue color the negative ones. Green color shows favorable shape/hydrophobic regions, and purple color the unfavorable ones.

**Figure 9:**
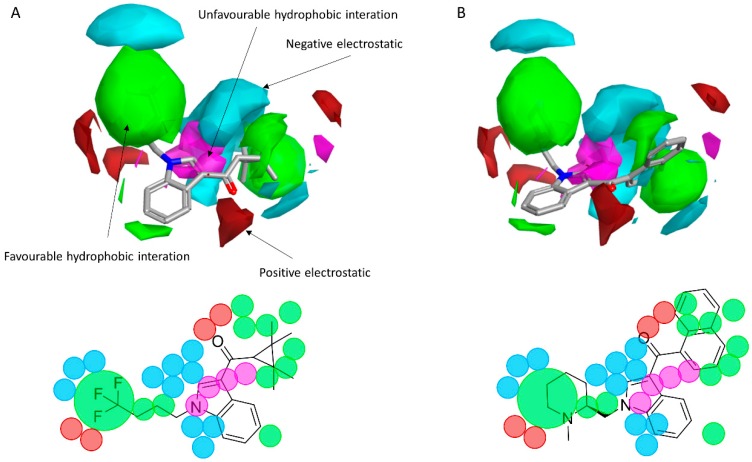
(**A**) The CB_2_ model map is superimposed to XLR-12. (**B**) The CB_2_ model map is superimposed to AM-1220. Molecular insight of SAR mechanism models, revealing the different lead optimization sites of active compounds. Red color shows positive field region controlling the activity, and blue color the negative ones. Green color shows favorable shape/hydrophobic regions, and purple color the unfavorable ones.

To reveal the key features of the studied set of compounds against the targeted cannabinoid receptors, activity-atlas (AA) was used to perform a structure–activity relationship (SAR) study. AA is a visualization software implemented in Forge that is able to summarize structure–activity data into 3D maps in a qualitative manner through a Bayesian statistics approach. Figure 8 and Figure 9 illustrate the results of the AA calculations for the CB_1_ and CB_2_ receptor, respectively. The model map is superimposed to the two most selective CB_1_ and CB_2_ receptor ligands, AM-1220 and XLR-12, respectively. The different colors on the maps derived from the AA calculations highlight electrostatic, hydrophobic, and shape features of the different set of compounds. A more positive electrostatic field increases the receptor-affinity in the red region, whereas in the blue area, a more negative electrostatic field increases the affinity. The green and the violet areas account for the steric and bulk/hydrophobic interactions. In the green area, a steric/bulk interaction improves the binding affinity; in the violet area, a steric/bulk interaction decreases the affinity. The two selected molecules (AM-1220 and XLR-12) can be dissected in three different regions: (i) the substituent at the N_1_ position of the heterocyclic nucleus; (ii) the substituent at the 3-position of the heterocyclic nucleus; (iii) the central core. Such molecular features are typically described nowadays to distinguish most of the novel and emerging synthetic cannabinoids [12]. Each of these moieties possesses a set of information for the CB_1_ or CB_2_ affinity/selectivity. Regarding the substitution of the N_1_ position, both models describe a wide favorable hydrophobic interaction area and in both models a negative electrostatic area is located at the end of the alkyl chain, suggesting that the introduction of electronegative atoms increases the affinity for both CB receptors. The occupancy of this area is fundamental for the affinity in both receptors; indeed, ligands that do not bear any substituent in this area result in low potency (e.g., JWH-042, *K*_i_ = 10,000 nM for CB_1_ and 5050 nM for CB_2_). On the other hand, this molecular portion appears to be linked with selectivity towards the CB_1_ receptor. A positive electrostatic region surrounds the initial part of that area, and a favorable interaction seems to be relevant for the selectivity toward the CB_1_ receptor (e.g., AM-1220). The same positive electrostatic area in the CB_2_ model doesn’t have any relevant interaction with potent and/or selective compounds for that receptor. For both models, there is not a strong SAR in the central core region, but a small favorable shape/hydrophobic region (green) is located near the positions 5 and 6 of the indole ring for the CB_2_ receptor, which means that small substituents in this region may increase the selectivity. The 2-position of the heterocyclic nucleus is located near an area in which the hydrophobic interactions are unfavorable. This area is larger for the CB_1_ model. Thus, in this case, the introduction of a small substituent may be better tolerated by the CB_2_ receptor. In the 3-position of the heterocyclic nucleus, the two models clearly describe different scenarios. For the CB_1_ receptor, the substituent in this branch is located within an unfavorable hydrophobic region; for the CB_2_ receptor, the situation is the opposite. This means that bulkier and hydrophobic substituents will raise the selectivity for the CB_2_ receptor and small/hydrophilic substituent will raise the selectivity toward the CB_1_ receptor. Furthermore, the same region in the CB_2_ model is contoured by a bigger negative electrostatic area, which means that the adding of an electronegative atom should increase the CB_2_ affinity. Also, it is interesting to note the different distribution of the 3D maps, among the two models, in the areas around the oxygen of the carbonyl group. In the CB_1_ model the oxygen is located near an area in which positive electrostatic coefficients will raise the affinity; this means that the oxygen atom in that position bears an unfavorable electrostatic contribution to the resultant ligand-receptor affinity. Conversely, the same oxygen in the CB_2_ model is located in an area where the negative electrostatic coefficient raises the potency; in fact, a relevant favorable electrostatic contribution is described by the model for the oxygen atom of the carbonyl group. Therefore, despite the carbonyl group present in most compounds designed as a CB_1_ ligand, the selection of a different connecting group might be exploited to improve the affinity for the CB_1_ receptor.

### 2.2. Finding Bioisosteres

**Figure 10:**
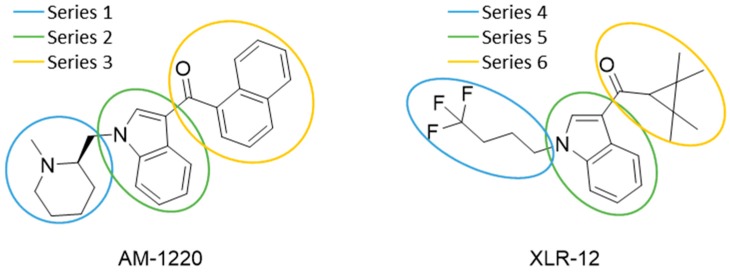
Bioisosteric replacement of selected compounds. The different studied parts of the molecules are highlighted in blue, green, and yellow.

Five hundred compounds were generated for each substitution, as reported in Figure 10, for a total of 3000 analogues, which were then evaluated by the superposition on the 3D-QSAR models. The top-scored compounds, by means of the CB_1_/CB_2_ predicted p*K*_i_, are reported in Table 1 and Table 2, while the full set of compounds is reported in the Supplementary Materials. Overall, the results indicate that the bioisosteric replacement and the following 3D-QSAR model evaluation generated new structures with the appropriate chemical features for the binding to the CB_1_ and the CB_2_ receptors. All the nine groups of the virtually evaluated compounds resulted in a series of molecules more potent than their precursors. The most interesting results for the CB_1_ receptor were achieved from the molecules belonging to the series 3 (Table 1). The most potent and selective compound for the CB_1_ receptor is the one in which the substituent at the 3-position of the heterocyclic nucleus was replaced by a 3-sulfonyl-indole. This molecule presents a predicted *K*_i_ of 1.3 and 199.5 nM for the CB_1_ and CB_2_ receptor, respectively, with a selectivity index (the selectivity index is defined as *K*_i_CB_2_/*K*_i_CB_1_) of 154.5 compared to an index of 18.8 for the original AM-1220 ligand (the higher the selectivity index, the higher the selectivity for CB_1_ receptor). The increase in selectivity has been achieved with a simple substitution of the group in 3-position to the central core. Further work around this area may provide additional molecular features to further increase selectivity for the CB_1_ receptor using scaffolds that have not been used yet. Interestingly, none of the compounds derived from the scaffold-hopping of XLR-12 (Table 1, series 4–6) achieve better selectivity upon the CB_1_ model.

On the other hand, in the case of the molecules virtually evaluated for the CB_2_ affinity, the most interesting results, in terms of affinity and selectivity, were achieved by four compounds present in series 4 and 6 (Table 2). The two compounds in the series 4 showed a predicted *K*_i_ of 12.6 and 15.8 nM for the CB_1_ receptor, and of 0.063 and 0.079 nM for the CB_2_ receptor. The calculated selectivity index is 0.005 for both compounds. The two compounds in series 6 both present a predicted *K*_i_ of 15.8 nM for the CB_1_ receptor, and of 0.063 and 0.079 nM for the CB_2_ receptor, with a calculated selectivity index of 0.004 and 0.005, respectively (the lower the selectivity index the higher the selectivity for CB_2_ receptor). Considering that the most selective molecule for CB_2_ receptor in our dataset has a selectivity index of 0.019, we can consider our results promising, and we can conclude that maintaining the central core unchanged, a large increase in the selectivity can be achieved by an appropriate substitution of the substituent at the N_1_ and at the 3-position of the heterocyclic nucleus.

## 3. Materials and Methods

### 3.1. Biological Data

The chemical structures of the 312 CB_1_ receptor ligands, and of the 187 CB_2_ receptor ligands were selected from the literature that reports the experimental *K*_i_ values, primarily involving assays where isolated CB_1_ and/or CB_2_ receptors were incubated with a predetermined fixed concentration of radiolabeled cannabinoid ([^3^H] CP 55,490) [26,27,28,29,30,31,32,33,34,35,36,37,38,39,40,41,42,43,56,57]. The binding affinity data of the selected dataset were converted into their negative decimal logarithm p*K*_i_ (p*K*_i_ = −log*K*_i_). Collected p*K*_i_ values fall into a range 5.00–10.03 and 5.00–9.92 for the CB_1_ and CB_2_ receptors, respectively.

### 3.2. Molecular Modeling

The structures of the studied molecules were built using Marvin Sketch (ChemAxon, Budapest, Hungary) [58]. The 2D structures were subjected to molecular mechanics energy minimization by Merck molecular force field (MMFF94) using Marvin Sketch [58]. A pH of 7.0 was assumed for the calculation of the protonation states of the molecules. The 3D structures derived from the force field minimization were further optimized at semi-empirical level using the parameterized model number 3 (PM3) Hamiltonian using MOPAC package (Stewart Computational Chemistry, Colorado Springs, CO, USA) (vMOPAC2016) as software [59,60,61].

### 3.3. Compound Alignment

All the 3D molecules, with their experimentally measured p*K*_i_ values, were imported into the chemistry software Forge (v10.4.2, Cresset, New Cambridge House, UK). Out of the 312 ligands for the CB_1_ receptor, 250 molecules (80%) were randomly selected as a training set. The remaining 62 compounds (20%) were used as test set for the model quality evaluation. For the CB_2_ receptor, we used the same procedure: selecting 150 ligands (80%) as training set and 37 compounds (20%) as test set [62]. For both training and test sets, the selected molecules covered a wide range of biological activities. All the molecules were aligned using AM11542 in its bioactive co-crystallized conformation as a template [63]. The field points electrostatic, shape, and hydrophobic field points of each molecule were generated using the extended electron distribution force field, proprietary of the Cresset group. A maximum common substructure algorithm was used to align the full set of molecules, a customized set-up was used (Figure S2, Supplementary Materials) [64]. Five hundred different conformers were generated for each molecule. The similarity between two different conformers was calculated by means of root-mean-square deviation of atomic positions (RMSD), two conformers with values less than 0.5 Å RMSD are considered identical. The gradient cutoff for conformers minimization was set to 0.1 kcal/mol, and the energy window was set to 2.5 kcal/mol. Conformers with calculated energy (kcal/mol) outside the energy window were rejected. All the alignments’ generated poses were manually checked. All the field points of the training set were analyzed by the software to reduce the number of descriptors to be considered. A distance of 1 Å between the sample points was used to calculate the sample values of each compound, in order to efficaciously described all the areas around the analyzed compound. Other information about the conformation calculation, the alignment, and the build of the model are reported in the Supplementary Materials (Figures S1–S6).

### 3.4. Bioisosteric Replacement

The bioisosteric replacement study was performed with Spark [65]. We studied the bioisosteric replacement in different portions of the two most selective compounds in our dataset for CB_1_ and CB_2_ receptors (Figure 1, AM-1220 and XLR-12). In those two molecules, the substitutions at the N_1_ and at the 3-position of the heterocyclic nucleus, and the substitution of the central core (Figure 10) were analyzed. Once the new virtual compounds were assembled, the new compounds were scored by the superposition on the different 3D-QSAR models, considering that if the fields of the compounds derived from the bioisosteric replacement are very similar to that of the original compounds, the resulting compounds will have similar biological properties [66,67]. The bioisosteric replacement was performed, in all the cases, using 240,068 fragments selected from ChEMBL, Zinc, and VEHICLe databases (See Supplementary Materials, Figure S7) [68,69,70].

## 4. Conclusions

Two 3D-QSAR models for CB_1_ and CB_2_ binders were developed in this study. The models were built and then used as a tool for the evaluation of different sets of compounds derived from a thorough scaffold-hopping analysis, for the design of novel high-affinity and selective molecules with novel scaffold and improved affinity/selectivity within such class of proteins. The QSAR models were built using Forge as a software and a set of 312 molecules for the CB_1_ receptor and 187 molecules for the CB_2_ receptor covering the whole range of different chemical classes of the ligands for these proteins. These two models are the first ones that include—within their descriptive capabilities—a wide range of chemically different compounds identified as cannabinoid receptors binders. The visualization of the models allowed to process the statistical data in a pharmacophoric description for both receptor, describing steric and electrostatic effects and rationalizing both potency and selectivity. The information reported in the 3D-QSAR models can ensure fruitful applications to speed up the design and the identification process of new potent CB_1_ and CB_2_ receptor ligands. However, since synthetic cannabinoids currently represent the most common substances used as NPS (from 2008 to 2017, a total of 179 new synthetic cannabinoids were reported to the European Monitoring Centre for Drugs and Drug Addiction (EMCDDA), and were the largest group of NPS [71]), and an elaborate variety of new synthetic cannabinoids are constantly designed in an attempt to avoid the legal restrictions on cannabis [12,72,73], the findings of this paper should also warn relevant forensic, public health, and legal authorities that new potent and unidentified scaffolds might be on their way to recreational use.

## Figures and Tables

**Table 1:** Chemical structures of the most potent compounds derived from the bioisosteric replacement for the CB_1_ receptor, the predicted *K*i value are presented as p*K*_i_.

**Series 1**	**Series 2**	**Series 3**
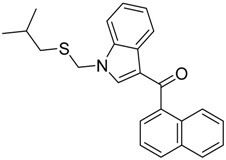	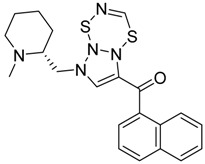	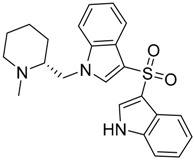
CB_1_ = 9.3 CB_2_ = 7.9	CB_1_ = 8.8 CB_2_ = 7.9	CB_1_ = 8.9 CB_2_ = 6.7
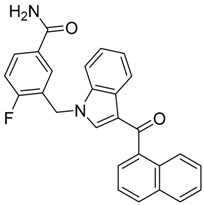	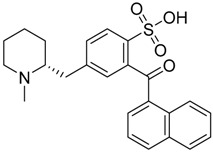	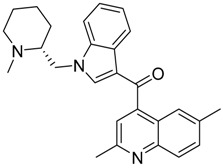
CB_1_ = 9.2 CB_2_ = 8.3	CB_1_ = 8.7 CB_2_ = 8.0	CB_1_ = 8.8 CB_2_ = 8.0
**Series 4**	**Series 5**	**Series 6**
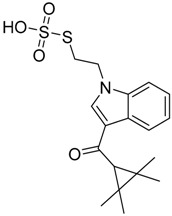	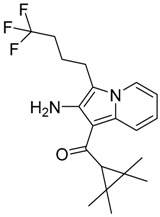	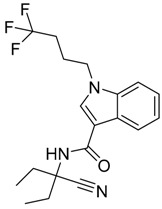
CB_1_ = 8.5 CB_2_ = 9.9	CB_1_ = 8.0 CB_2_ = 9.3	CB_1_ = 8.3 CB_2_ = 8.9
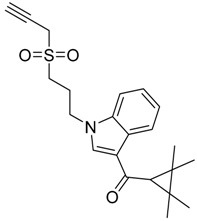	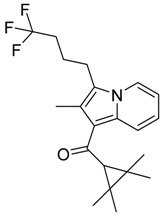	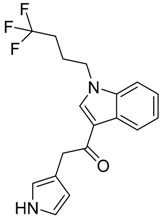
CB_1_ = 8.5 CB_2_ = 9.2	CB_1_ = 8.0 CB_2_ = 9.6	CB_1_ = 8.2 CB_2_ = 8.9

**Table 2:** Chemical structures of the most potent compounds derived from the bioisosteric replacement for the CB_2_ receptor, the predicted *K*i value are presented as p*K*_i_.

**Series 1**	**Series 2**	**Series 3**
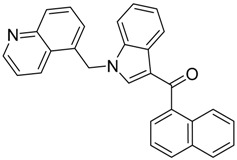	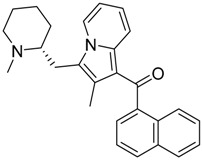	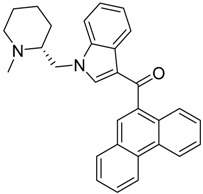
CB_2_ = 9.4 CB_1_ = 8.6	CB_2_ = 8.7 CB_1_ = 8.1	CB_2_ = 8.3 CB_1_ = 8.1
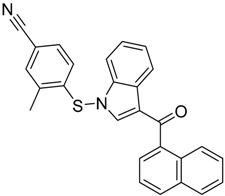	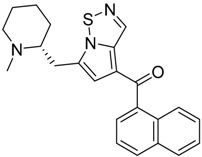	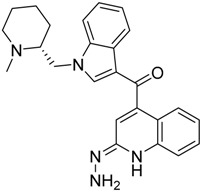
CB_2_ = 9.4 CB_1_ = 7.7	CB_2_ = 8.6 CB_1_ = 8.6	CB_2_ = 8.2 CB_1_ = 7.8
**Series 4**	**Series 5**	**Series 6**
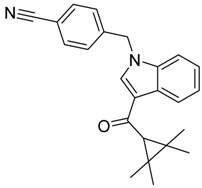	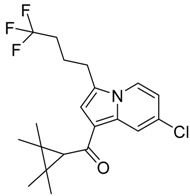	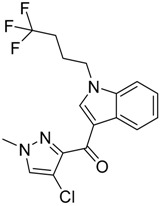
CB_2_ = 10.2 CB_1_ = 7.9	CB_2_ = 9.9 CB_1_ = 7.8	CB_2_ = 10.2 CB_1_ = 7.8
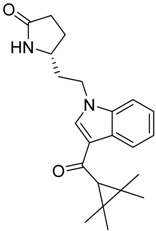	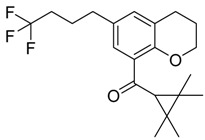	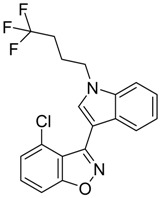
CB_2_ = 10.1 CB_1_ = 7.8	CB_2_ = 9.8 CB_1_ = 7.3	CB_2_ = 10.1 CB_1_ = 7.8

## Supplementary Materials

The following are available online. Statistical analysis information for the model, Figures S1–S7, Tables S1–S16.
