# Supplementary material for: Discovery of High-Affinity Cannabinoid Receptors Ligands through a 3D-QSAR Ushered by Scaffold-Hopping Analysis
Source: Molecules. 2018 Aug 30;23(9):2183. doi: 10.3390/molecules23092183 (PMC6225167; doi:10.3390/molecules23092183)
Supplement: Supplementary file 1 [file molecules-23-02183-s001.pdf]

molecules

MDPI

Supplementary material

Discovery of High-Affinity Cannabinoid Receptors Ligands through a 3D-QSAR Ushered by Scaffold-Hopping Analysis †

Giuseppe Floresta [1,2,3,*], Orapan Apirakkan [4], Antonio Rescifina [1] and Vincenzo Abbate [4,*]

1 Department of Drug Sciences, University of Catania, V.le A. Doria, 95125 Catania, Italy; arescifina@unict.it
2 Department of Chemical Sciences, University of Catania, V.le A. Doria, 95125 Catania, Italy
3 Institute of Pharmaceutical Science, King's College London, Stamford Street, London SE1 9NH, UK
4 King's Forensics, School of Population Health & Environmental Sciences, King's College London, Franklin-Wilkins Building, 150 Stamford Street, London SE1 9NH, UK; orapan.apirakkan@kcl.ac.uk
* Correspondence: giuseppe.floresta@unict.it (G.F.), vincenzo.abbate@kcl.ac.uk (V.A.)

† In memory of Professor Carmela Spatafora, a friend, colleague and distinguished scientist, on the second anniversary of her premature death.

Received: 23 August 2018; Accepted: 28 August 2018; Published: 30 August 2018

Table of content

Statistical analysis information for the model S2
Fig. S1. Forge's parameters used for the conformation hunt S3
Fig. S2. Forge's parameters used for the alignment S3
Fig. S4. Forge's parameters used for the build of the model S4
Fig. S5. Model statistics for CB_1 model S4
Fig. S6. Model statistics for CB_2 model S5
Fig. S7. Spark's parameters used for the bioisosteric replacement S5
Table S1. SMILES, experimental and predicted p*K*i values of the molecules in the training set for CB_1 receptor S6
Table S2. SMILES, experimental and predicted p*K*i values of the molecules in the test set for CB_1 receptor S12
Table S3. SMILES, experimental and predicted p*K*i values of the molecules in the training set for CB_2 receptor S13
Table S4. SMILES, experimental and predicted p*K*i values of the molecules in the test set for CB_2 receptor S17
Table S5. List, SMILE and predicted p*K*i values for Series 1 in CB_1 receptor S18
Table S6. List, SMILE and predicted p*K*i values for Series 2 in CB_1 receptor S30
Table S7. List, SMILE and predicted p*K*i values for Series 3 in CB_1 receptor S42
Table S8. List, SMILE and predicted p*K*i values for Series 4 in CB_1 receptor S54
Table S9. List, SMILE and predicted p*K*i values for Series 5 in CB_1 receptor S66
Table S10. List, SMILE and predicted p*K*i values for Series 6 in CB_1 receptor S78
Table S11. List, SMILE and predicted p*K*i values for Series 1 in CB_2 receptor S90
Table S12. List, SMILE and predicted p*K*i values for Series 2 in CB_2 receptor S102
Table S13. List, SMILE and predicted p*K*i values for Series 3 in CB_2 receptor S114
Table S14. List, SMILE and predicted p*K*i values for Series 4 in CB_2 receptor S126
Table S15. List, SMILE and predicted p*K*i values for Series 5 in CB_2 receptor S138
Table S16. List, SMILE and predicted p*K*i values for Series 6 in CB_2 receptor S151

Statistical analysis information for the model

The following conditions were used to calculate the field 3D-QSAR model. The leave-one-out method was used for the validation of the QSAR model. The maximum number of components to extract from the PLS regression was set to 20. The number of Y scrambles to use was set to 50, this means that in each scramble the activity values are randomly assigned to molecules and the model building process is repeated. More scramble sets provide stronger confirmation of statistical significance. The sample point minimum distance threshold was set to 1 Å. This option checks the sphere exclusion algorithm used to reduce the initial number of field sample positions down to a smaller set. Decrease the value sample point minimum distance increases the number of sample points, which may improve the model at the expense of increasing the probability of over-fitting. A value of 1 Å means that sample points must be at least 1 Å aside from each other. The predictive ability of the generated model was confirmed by different statistical tests. The leave-one-out method was used during the validation of the QSAR model which means that the model is built again but a single molecule left out of the process, this is then repeated leaving out each training set molecule in turn. The predicted activity for each molecule is the value obtained when it was left out of the model building process. The cross-validation regression coefficient (q^2) was calculated based on the prediction error sum of squares (PRESS) and the sum of squares of deviation of the experimental values from their mean (SSY):

$$q^2 - 1 - \frac{PRESS}{SSY} - 1 - \frac{\sum_{i=1}^{n}(Yexp - Ypred)^2}{\sum_{i=1}^{n}(Yexp - Ymean)^2}$$

where *Yexp* is the experimental activity of training set compound, *Ypred* is the predicted activity of training set compound, and *Ymean* is the mean values of the activity of training set compound.

The performance of the model was also validated through the determination of the coefficient in prediction, r^2_{test}, using the following equation:

$$r^2 test = 1 - \frac{\sum_{i=1}^{n}(Ypredtest - Ytest)^2}{\sum_{i=1}^{n}(Ytest - Ymean)^2}$$

where *Ypredtest* is the predicted activity of test set compound by QSAR equation, *Ytest* is the experimental activity of test set compound, and *Ymean* is the mean values of the activity of training set compound.

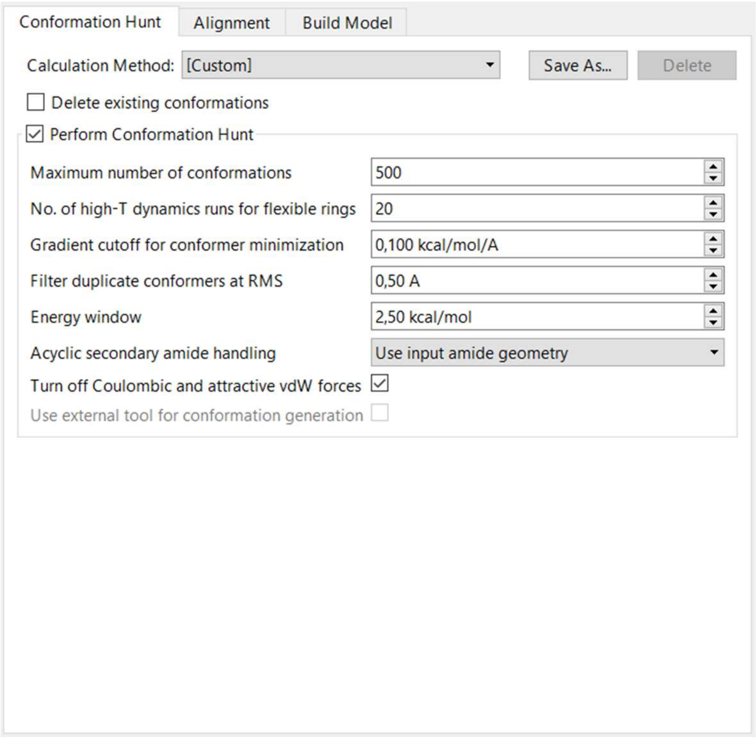

Fig. S1. Forge's parameters used for the conformation hunt.

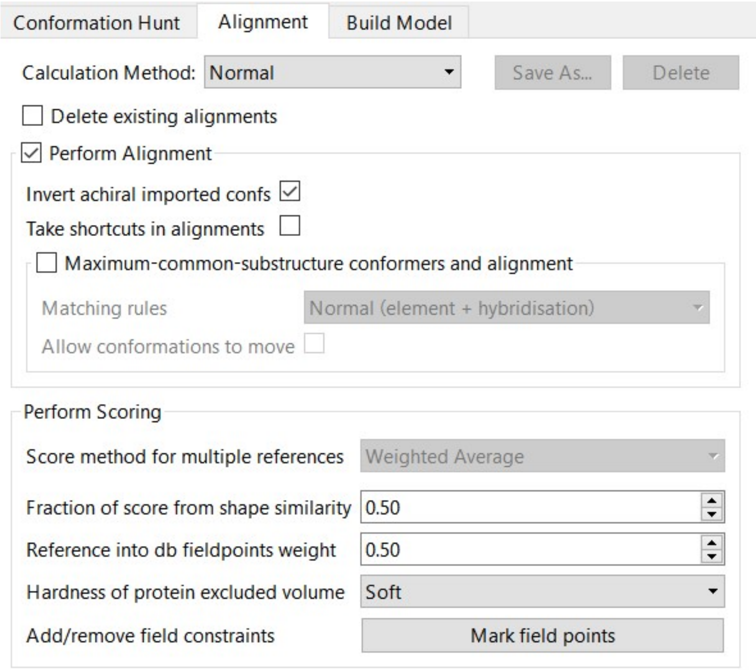

Fig. S2. Forge's parameters used for the alignment.

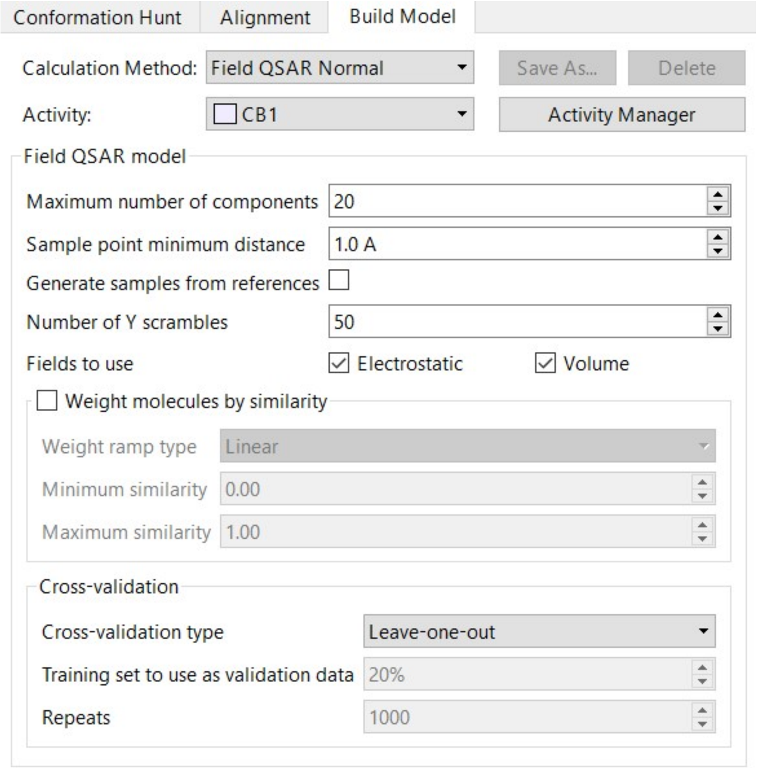

Fig. S4. Forge's parameters used for the build of the model.

```
Model statistics:
=================
```

Comps	R^2	Q^2	Test R^2	RMSE	RMSEpred	Tau	Tau-pred
0	-0.000	-0.008	-0.001	0.987	0.991	-0.188	-0.975
1	0.356	0.306	0.391	0.786	0.818	0.451	0.412
2	0.504	0.431	0.421	0.692	0.743	0.522	0.478
3	0.676	0.521	0.517	0.562	0.685	0.614	0.519
4	0.774	0.572	0.617	0.470	0.649	0.687	0.551
5	0.841	0.588	0.640	0.392	0.637	0.739	0.564
6	0.883	0.594	0.692	0.334	0.630	0.784	0.571
7	0.909	0.603	0.694	0.296	0.623	0.811	0.570
8	0.924	0.614	0.723	0.269	0.614	0.831	0.578
9	0.940	0.622	0.727	0.239	0.606	0.852	0.587
10*	0.947	0.624	0.726	0.224	0.604	0.864	0.586
11	0.959	0.613	0.725	0.196	0.613	0.879	0.585
12	0.968	0.601	0.721	0.175	0.622	0.894	0.581
13	0.973	0.585	0.718	0.161	0.634	0.903	0.575
14	0.978	0.569	0.717	0.143	0.646	0.912	0.568
15	0.983	0.552	0.704	0.125	0.659	0.924	0.560
16	0.987	0.541	0.704	0.111	0.667	0.934	0.557
17	0.990	0.531	0.692	0.098	0.675	0.944	0.553
18	0.992	0.525	0.681	0.089	0.679	0.947	0.549
19	0.994	0.520	0.665	0.077	0.683	0.954	0.549
20	0.995	0.517	0.658	0.070	0.686	0.959	0.547

Fig. S5. Model statistics for CB_1 model.

Model statistics:
=================

Comps	R^2	Q^2	Test R^2	RMSE	RMSEpred	Tau	Tau-pred
0	-0.000	-0.013	-0.030	0.975	0.981	0.101	-0.980
1	0.513	0.263	0.483	0.680	0.840	0.514	0.368
2	0.658	0.439	0.619	0.568	0.730	0.583	0.436
3	0.805	0.568	0.620	0.429	0.641	0.710	0.529
4	0.838	0.579	0.672	0.390	0.633	0.728	0.532
5	0.882	0.600	0.685	0.333	0.617	0.772	0.547
6	0.907	0.611	0.667	0.296	0.609	0.795	0.548
7*	0.933	0.613	0.720	0.250	0.607	0.822	0.546
8	0.955	0.608	0.732	0.205	0.611	0.859	0.548
9	0.965	0.600	0.688	0.181	0.618	0.880	0.544
10	0.974	0.589	0.678	0.154	0.627	0.893	0.532
11	0.982	0.584	0.662	0.129	0.632	0.917	0.534
12	0.987	0.581	0.632	0.109	0.634	0.927	0.530
13	0.991	0.583	0.622	0.092	0.632	0.943	0.532
14	0.992	0.586	0.603	0.083	0.630	0.951	0.533
15	0.995	0.590	0.586	0.070	0.627	0.955	0.541
16	0.996	0.590	0.586	0.059	0.627	0.964	0.543
17	0.998	0.590	0.575	0.047	0.628	0.968	0.540
18	0.998	0.588	0.572	0.039	0.629	0.975	0.539
19	0.999	0.584	0.563	0.032	0.632	0.980	0.538
20	0.999	0.580	0.562	0.026	0.635	0.984	0.538

Fig. S6. Model statistics for CB_2 model.

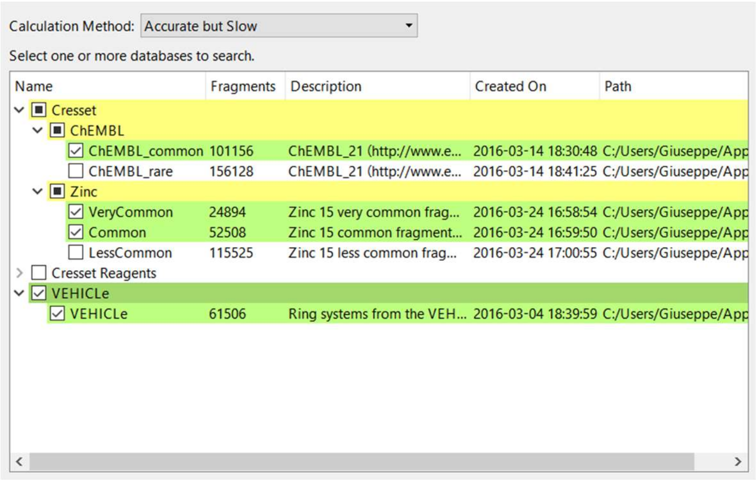

Fig. S7. Spark's parameters used for the bioisosteric replacement.

Table S1. SMILES, experimental and predicted pK_i values of the molecules in the training set for CB_1 receptor.

		pK_i	
**N°**	**SMILES**	**Exp**	**Pred**
1	O=C(c1c2ccccc2n(CCCCCC)c1C)c3cccc4ccccc43	7.32	7.1
2	O=C(c1c2ccccc2n(c1C)CCC)c3cccc4ccccc43	6.47	6.8
3	O=C(c1cn(CCCCC)c2ccccc21)c3cccc4ccccc43	8.05	7.9
4	O=C(c1c(n(c2ccccc21)C)C)c3cccc4ccccc43	5	5.1
5	O=C(c1c2ccccc2n(CCCC)c1C)c3cccc4ccc(cc43)C	7.23	7.2
6	O=C(c1c2ccccc2n(CCCCC)c1C)c3cccc4ccc(cc43)C	7.97	7.7
7	O=C(c1c2ccccc2n(CCCCCC)c1C)c3cccc4ccc(cc43)C	7.26	7.2
8	O=C(c1cn(c2ccccc21)CC)c3cccc4ccccc43	5.87	5.9
9	O=C(c1cn(CCCC)c2ccccc21)c3cccc4ccccc43	8.05	7.7
10	O=C(c1c2ccccc2n(CCC)c1)c3cccc4ccc(cc34)C	6.67	7.1
11	O=C(c1cn(c2ccccc21)CC)c3ccc(OC)c4ccccc43	6.09	6.2
12	O=C(c1cn(CCCCC)c2ccccc21)c3ccc(OC)c4ccccc43	8.92	9.1
13	O=C(c1c2ccccc2n(c1CCCC)CCC)c3ccc(OC)c4ccccc43	7.39	7.3
14	O=C(c1c2ccccc2n(c1C)CCC)c3ccc(OC)c4ccccc43	6.32	6.4
15	O=C(c1c2ccccc2n(c1CCCCC)CCCC)c3ccc(OC)c4ccccc43	6.85	6.8
16	O=C(c1c2ccccc2n(c1CCCCCC)CCCCC)c3ccc(OC)c4ccccc43	6.34	6.4
17	O=C(c1c2ccccc2n(CCCCCC)c1C)c3ccc(OC)c4ccccc43	7.45	7.4
18	O=C(c1c2ccccc2n(c1CC)CCCCC)c3cccc4ccccc43	7.28	7.2
19	O=C(c1cn(CCCCC)c2ccccc21)c3ccc(c4ccccc43)C	9.16	9
20	O=C(c1c2ccccc2n(CCCCC)c1C)c3ccc(c4ccccc43)C	8.3	8.2
21	O=C(c1c2ccccc2n(CCCCC)c1)c3cccc4ccc(OC)cc43	8.18	8.1
22	O=C(c1c2ccccc2n(CCCCC)c1)c3cccc4cc(OC)ccc34	7.36	7
23	O=C(c1cn(c2ccccc21)CCC)c3ccc(c4ccccc43)CCC	7.59	7.3
24	O=C(c1c2ccccc2n(c1C)CCC)c3ccc(c4ccccc34)CCC	7.28	6.3
25	O=C(c1cn(CC[N+]2CCOCC2)c3ccccc31)c4ccc(OC)c5ccccc54	8	8.2
26	O=C(c1cn(CC[N+]2CCOCC2)c3ccccc31)c4cccc5ccccc54	7.38	7.6
27	O=C(c1c2ccccc2n(c1C)CCC)c3ccc(c4ccccc34)CC	7.15	6.8
28	O=C(c1cn(c2ccccc21)CCC)c3ccc(c4ccccc34)CC	7.48	7.6
29	O=C(c1c2ccccc2n(CCCCC)c1C)c3ccc(c4ccccc34)CC	8.82	8.2
30	O=C(c1c2ccccc2n(CCCCC)c1)c3cccc4ccc(CC)cc34	8.08	7.9
31	O=C(c1c2ccccc2n(CCC)c1)c3cccc4ccc(CC)cc34	6.47	6.6
32	O=C(c1c2ccccc2n(CCC)c1C)c3cccc4ccc(CC)cc43	5.87	6.3
33	O=C(c1c2ccccc2n(c1C)CCC)c3ccc(CCCC)c4ccccc34	6.83	6.9
34	O=C(c1cn(CCCCC)c2ccccc21)c3ccc(OCC)c4ccccc43	8.34	8.5
35	O=C(c1c2ccccc2n(CCCCC)c1C)c3cccc4ccc(CC)cc43	7.55	7.3
36	Brc1ccc(C(=O)c2c3ccccc3n(c2C)CCC)c4ccccc14	6.43	6.3
37	Clc1ccc(C(=O)c2c3ccccc3n(c2C)CCC)c4ccccc14	6.73	6.7
38	Clc1ccc(c2ccccc12)C(=O)c3cn(c4ccccc43)CCC	7.03	7.3

39	Fc1ccc(c2ccccc12)C(=O)c3cn(CCCCC)c4ccccc43	8.14	8.1
40	Fc1ccc(c2ccccc12)C(=O)c3cn(c4ccccc43)CCC	6.62	6.9
41	Fc1ccc(C(=O)c2c3ccccc3n(c2C)CCC)c4ccccc14	6.28	6.2
42	FCCCCCn1cc(c2ccccc21)C(=O)c3ccc(c4ccccc43)C	8.8	8.9
43	F[C@@H](CCCn1cc(c2ccccc21)C(=O)c3ccc(c4ccccc43)C)C	8.49	8.8
44	FCCCCCn1cc(c2ccccc21)C(=O)c3ccc(c4ccccc34)CC	9.42	9.3
45	FCCCCCn1c2ccccc2c(n1)C(=O)c3cccc4ccccc43	8.87	8.5
46	FCCCCCn1cc(c2ccccc21)C(Oc3cccc4cccnc43)=O	9.33	9.2
47	Fc1ccccc1-n2c3ccccc3c(n2)C(Oc4ccc5ccccc5c4)=O	5	4.9
48	FCCCCCn1c2ccccc2c(n1)C(Oc3cccc4ccccc34)=O	8.59	8.7
49	FCCCCCn1cc(c2ccccc21)C(=O)NC34CC5CC(C3)CC(C4)C5	8.6	8.2
50	O=C(NCc1ccccc1)c2c3ccccc3n(CCCCC)c2	7.28	7.1
51	FCCCCCn1cc(c2ccccc21)C(=O)NCc3ccccc3	7.14	7.1
52	O=C(Nc1ccccc1)c2cn(CCCCC)c3ccccc32	6.79	7.3
53	O=C(Nc1cccc2ccccc21)c3cn(CCCCC)c4ccccc43	8.74	8.6
54	FCCCCCn1cc(c2ccccc21)C(=O)Nc3cccc4ccccc43	8.43	8.6
55	ClCCCCCn1cc(c2ccccc21)C(=O)Nc3cccc4ccccc43	8.43	8.6
56	Fc1ccc(Cn2c3ccccc3c(n2)C(=O)NC45CC6CC(C4)CC(C5)C6)cc1	8.97	9
57	O=C(Nc1cccc2ccccc21)c3c4ccccc4n(n3)CCCCC	8.41	8
58	O=C(c1c2ccccc2n(CCCCC)c1)c3ccccc3OC	7.58	8.1
59	O=C(c1c2ccccc2n([C@H](CCCCC)C)c1C)c3cccc4ccccc43	7.32	7.7
60	Brc1cccc2cccc(c12)C(=O)c3cn(CCCCC)c4ccccc43	7.68	7.6
61	FCCCCCn1cc(c2ccc([N+]([O-])=O)cc21)C(=O)c3cccc4ccccc43	8.82	8.7
62	O=C(c1cn(CCCCC#N)c2ccccc21)c3cccc4ccccc43	9.55	9.6
63	O=C(c1ccc(OCCCCC)c2ccccc12)c3cccc4ccccc43	7.82	7.9
64	FCCCCCn1cc(c2ccccc21)C(=O)NC(c3ccccc3)(C)C	7.3	7.5
65	O=C(N)[C@@H](NC(=O)c1c2ccccc2n(n1)CCCCC)C(C)C	8.54	8.4
66	Fc1ccc(Cn2c3ccccc3c(n2)C(=O)N[C@@H](C(C)C)C(=O)N)cc1	9.05	9
67	O=C(N)[C@@H](NC(=O)c1c2ccccc2n(n1)CC3CCCCC3)C(C)(C)C	9.54	9.3
68	O=C(OC)[C@@H](NC(=O)c1c2ccccc2n(n1)CC3CCCCC3)C(C)(C)C	10.03	9.9
69	Fc1ccc(Cn2c3ccccc3c(n2)C(=O)N[C@@H](C(C)(C)C)C(OC)=O)cc1	8.94	8.7
70	O=C(C1C(C1(C)C)(C)C)c2c3ccccc3n(CC4CCOCC4)c2	7.6	7.8
71	O=C(C12CC3CC(C1)CC(C2)C3)c4c5ccccc5n(CCCCC)c4	7.48	7.4
72	O=C(C1C(C1(C)C)(C)C)c2c3ccccc3n(CCCCC)c2	7.25	7.4
73	FCCCCCn1c(c(C(=O)C2C(C2(C)C)(C)C)c3ccccc31)C	7.71	7.5
74	FCCCCCn1c2ccccc2c(n1)C(=O)C3C(C3(C)C)(C)C	7.76	7.5
75	FC(F)(F)CCCn1cc(C(=O)C2C(C2(C)C)(C)C)c3ccccc31	7.36	7.5
76	O=C(c1cn(CCCCCCC)c(-c2ccccc2)c1)c3cccc4ccccc43	7.68	7.5
77	O=C(c1cn(CCCC)c(-c2ccccc2)c1)c3cccc4ccccc43	7.22	7.5
78	O=C(c1cn(CCCCC)c(-c2ccc(OC)cc2)c1)c3cccc4ccccc43	6.55	6.8
79	O=C(c1cn(CCCCC)c(-c2ccc(cc2)C)c1)c3cccc4ccccc43	6.89	7.1
80	Clc1ccc(-c2cc(cn2CCCCC)C(=O)c3cccc4ccccc43)cc1	6.56	6.9

81	O=C(c1cn(CCCCC)c(-c2ccccc2OC)c1)c3cccc4ccccc43	7.54	7.6
82	Fc1ccccc1-c2cc(cn2CCCCC)C(=O)c3cccc4ccccc43	8.11	7.9
83	O=C(c1cc(n(CCCCC)c1)-c2cccc3ccccc32)c4cccc5ccccc54	7.39	7.4
84	O=C(c1cn(CCCCC)c(-c2cccc(c2)C)c1)c3cccc4ccccc43	7.17	6.9
85	FC(F)(F)c1cccc(-c2cc(cn2CCCCC)C(=O)c3cccc4ccccc43)c1	6.61	7.3
86	Fc1cccc(-c2cc(cn2CCCCC)C(=O)c3cccc4ccccc43)c1	7.8	7.9
87	Clc1ccccc1-c2cc(cn2CCCCC)C(=O)c3cccc4ccccc43	8.1	8
88	O=C(c1cn(CCCCC)c(-c2ccc(CCCC)cc2)c1)c3cccc4ccccc43	7.38	7.2
89	FC(F)(F)c1ccccc1-c2cc(cn2CCCCC)C(=O)c3cccc4ccccc43	7.11	6.9
90	O=C(c1cn(c(-c2ccccc2CCCC)c1)CCCCC)c3cccc4ccccc43	7.22	7.6
91	FC(F)(F)c1ccccc1-c2cc(cn2CCCCC)C(=O)c3cccc4ccccc43	7.11	7.1
92	O=C(c1c2ccccc2n(CCCCC)c1)Cc3ccccc3	7.05	7
93	O=C(c1c2ccccc2n(CCCCC)c1C)Cc3ccccc3	6.91	6.9
94	Brc1ccc(CC(=O)c2c3ccccc3n(CCCCC)c2)cc1	5.99	6.5
95	Brc1ccccc1CC(=O)c2c3ccccc3n(CCCCC)c2	8.08	8.5
96	O=C(Cc1ccccc1OC)c2c3ccccc3n(CCCCC)c2	7.96	8.3
97	O=C(c1cn(CCCCC)c2ccccc21)Cc3ccccc3C	7.54	7.4
98	O=C(c1c2ccccc2n(CCCCC)c1)Cc3cccc(OC)c3	7.77	8
99	Clc1cccc(CC(=O)c2c3ccccc3n(CCCCC)c2C)c1	6.93	7.1
100	Brc1ccccc1CC(=O)c2c3ccccc3n(CCCCC)c2C	7.82	7.8
101	Fc1ccc(CC(=O)c2c3ccccc3n(CCCCC)c2)cc1	6.37	6.4
102	Fc1ccc(CC(=O)c2c3ccccc3n(CCCCC)c2C)cc1	5.54	5.7
103	Oc1cc(CCCCC)cc2c1-c3cc(ccc3C(O2)(C)C)C	6.51	6.2
104	FCCCCCc1cc(O)c2c(OC([C@@H]3CC=C(C[C@@H]23)C)(C)C)c1	7.24	7.3
105	O[C@@H]1CC[C@H]([C@@H](C1)c2ccc(C(CCCCCC)(C)C)cc2O)CCCO	9.23	9.4
106	O[C@H]1CC[C@@H]([C@H](C1)c2ccc(C(CCCCCC)(C)C)cc2O)CCCO	7.21	7
107	O=C(NCCO)CCC/C=C\C/C=C\C/C=C\C/C=C\CCCCC	7.05	7.1
108	CCCCC/C=C\C/C=C\C/C=C\C/C=C\CCCC[N+]1CCOCC1	5.94	5.7
109	CCCCC/C=C\C/C=C\C/C=C\C/C=C\CCCC[N+]CCOC	5.74	6
110	Fc1ccc(Cn2cc(c3ccccc32)C(=O)c4cccc5ccccc54)cc1	8.49	8.7
111	FCCCCCn1cc(c2ccccc21)C(=O)c3ccc(F)c4ccccc43	9.07	8.7
112	Clc1ccc(c2ccccc12)C(=O)c3cn(CCCCCF)c4ccccc43	9.11	9
113	FCCCCCn1c(nc2ccccc21)C(=O)c3cccc4ccccc43	6.53	6.8
114	O=C(OC)[C@@H](OC(=O)c1c2ccccc2n(n1)CC3CCCCC3)C(C)(C)C	7.98	8.2
115	Fc1ccc(Cn2cc(c3ccccc32)C(=O)Nc4cccc5ccccc54)cc1	8.13	7.8
116	FCCCCCn1cc(C(=O)N[C@@H](C(C)C)C(=O)N)c2ccccc21	7.46	7.5
117	O=C(N)[C@@H](NC(=O)c1c2ccccc2n(CC3CCCCC3)c1)C(C)(C)C	8.91	9
118	O=C(OC)[C@@H](NC(=O)c1c2ccccc2n(CC3CCCCC3)c1)C(C)(C)C	9.39	9.5
119	FCCCCCn1cc(c2ccccc21)C(=O)N[C@H](C(C)C)C(OC)=O	7.82	8.2
120	O=C(NC(c1ccccc1)(C)C)c2c3ccccc3n(n2)CC4CCOCC4	8.91	8.7
121	Fc1cccc(Cn2c3ccccc3c(n2)C(=O)N[C@@H](C(C)C)C(=O)N)c1	7.9	8.1
122	Fc1ccccc1Cn2c3ccccc3c(n2)C(=O)N[C@@H](C(C)C)C(=O)N	8.16	8.2

123	FCCCCCn1c2ccccc2c(n1)C(=O)N[C@@H](C(C)C)C(=O)N	8.3	8.4
124	FCCCCCn1c2ccccc2c(n1)C(=O)N[C@H]([C@H](CC)C)C(=O)N	8.51	8.1
125	O=C(N)[C@@H](NC(=O)c1c2ccccc2n(n1)CC3CCCC3)C(C)(C)C	9.48	9.5
126	Fc1ccc(Cn2c3ccccc3c(n2)C(=O)N[C@@H](C(C)(C)C)C(=O)N)cc1	9.44	9.7
127	O=C(OC)[C@H](NC(=O)c1c2ccccc2n(n1)CC3CCCCC3)C(C)C	9.47	9.2
128	O=C(N[C@@H](Cc1ccccc1)C(=O)N)c2c3ccccc3n(n2)CC4CCCCC4	8.01	8
129	Ic1ccccc1C(=O)c2c3ccccc3n(CCCCC)c2	7.87	7.7
130	O=C(N1CC[N+](CC1)C)c2c3ccccc3n(CCCCC)c2	5.58	5.7
131	O=C(OC)[C@@H](NC(=O)c1ccc2c(c3ccccc3n2CC4CCCCC4)c1)C(C)(C)C	8.24	8.5
132	O=C(c1ccc2c(c3ccccc3n2CCCCC)c1)c4cccc5ccccc54	8.14	8.1
133	FCCCCCn1c2ccc(cc2c3ccccc31)C(=O)c4cccc5ccccc54	7.65	8
134	O=C(N)[C@@H](NC(=O)c1c2ccccc2n(n1)CC3CCCCC3)C(C)(C)C	9.54	9.6
135	O=C(NC1CC1)[C@@H](NC(=O)c2c3ccccc3n(n2)CCCCC#N)C(C)(C)C	8.54	8.7
136	Clc1ccc2c(c(nn2CC3CCOCC3)C(=O)N[C@@H](C(C)(C)C)C(=O)N)c1	8.87	9
137	O=C(N[C@@H](CC(C)C)C(=O)N)c1c2ccccc2n(n1)CC3CCCCC3	8.47	8.3
138	Clc1ccc2c(c(nn2CC3CCOCC3)C(=O)N[C@@H](C(C)C)C(=O)N)c1	8.37	8.2
139	O=C(N)[C@@H](NC(=O)c1c2ccccc2n(n1)CC3CCOCC3)C(C)C	7.38	7.5
140	Clc1ccc2c(c(nn2CC3CCOCC3)C(=O)N[C@@H](C(C)(C)C)C(=O)NC4CC4)c1	7.58	7.6
141	Clc1ccc2c(c(nn2CC3CCOCC3)C(=O)N[C@@H](C(C)(C)C)C=4[N-]N=NN4)c1	7.89	8
142	Clc1ccc2c(c(nn2CC3CCOCC3)C(=O)N[C@@H](C(C)(C)C)C(=O)NC[C@@H](O)CO)c1	7.52	7.4
143	O=C(N)[C@@H](NC(=O)c1c2ccccc2n(n1)CC3CCOCC3)C(C)(C)C	8.73	8.6
144	O=C(N[C@@H](CC(C)C)C(=O)N)c1c2ccccc2n(n1)CC3CCOCC3	7.34	7.2
145	Fc1ccc2c(c(nn2CC3CCOCC3)C(=O)N[C@@H](C(C)(C)C)C(=O)NCCO)c1	9.76	9.9
146	O=C(OCC)[C@@H]1CCCC[C@@H]1NC(=O)c2c3ccccc3n(n2)CC4CCOCC4	7.86	8
147	Fc1ccc2c(c(nn2CC3CCCCC3)C(=O)N[C@@H](C(C)(C)C)C(=O)NCC(=O)N)c1	9.84	9.7
148	O=C(N)[C@@H](NC(=O)c1c2ccccc2n(n1)CCCCC#N)C(C)(C)C	8.35	8.5
149	O=C(N)[C@@H](NC(=O)c1c2ccccc2n(n1)CCCCC#N)C(C)C	7.96	7.6
150	O=C(N)[C@@H](NC(=O)c1c2cccnc2n(n1)CC3CCCCC3)C(C)(C)C	9.24	9.2
151	Fc1cccc2c1n(nc2C(=O)N[C@@H](C(C)(C)C)C(=O)NC3CC3)CC4CCOCC4	8.21	8.2
152	Fc1cccc2c1n(nc2C(=O)N[C@@H](C(C)(C)C)C(=O)NCCCO)CC3CCOCC3	9.15	9.3
153	O=C(N)[C@@H](NC(=O)c1c2ccccc2n(n1)C[C@@H]3CCCCO3)C(C)(C)C	8.39	8.5
154	Fc1cccc2c1n(nc2C(=O)N[C@@H](C(C)(C)C)C(=O)NCC(=O)N)CC3CCOCC3	8.76	8.7
155	Fc1ccc2c(c(nn2CC3CCCCC3)C(=O)N[C@H](C(=O)NCCCO)C(C)(C)C)c1	9.46	9.5
156	Fc1ccc2c(c(nn2CC3CCOCC3)C(=O)N[C@@H](C(C)(C)C)C(=O)NCCCO)c1	8.21	8.2
157	OC[C@@H](NC(=O)c1c2ccccc2n(n1)CC3CCCCC3)c4ccccc4	6.93	7
158	Fc1ccc2c(c(nn2CC3CCCCC3)C(=O)N[C@H](C(=O)NC4CC4)C(C)(C)C)c1	8.4	8.6
159	Fc1ccc2c(c(nn2CCCCC#N)C(=O)N[C@@H](C(C)(C)C)C(=O)N)c1	8.05	8.5
160	O=C(N[C@H]1CCCC[C@@H]1OCc2ccccc2)c3c4ccccc4n(n3)CC5CCCCC5	7.05	6.8
161	Fc1ccc2c(c(nn2CCCCC#N)C(=O)N[C@@H](C(C)(C)C)C(=O)NCC(=O)N)c1	8.22	8.1
162	O=C(NC1CC1)[C@@H](NC(=O)c2c3ccccc3n(n2)CC4CCCCC4)C(C)C	8.25	8.4
163	O=C(NCC([O-])=O)[C@@H](NC(=O)c1c2ccccc2n(n1)CCCC#N)C(C)(C)C	8.69	8.8
164	FC(F)(F)Oc1ccc2c(c(nn2CC3CCCCC3)C(=O)N[C@@H](C(C)(C)C)C(=O)NCCO)c1	7.48	7.4

165	O=C(NCC(=O)N)[C@@H](NC(=O)c1c2ccccc2n(n1)CC3CCCCC3)C(C)C	7.68	7.8
166	FC(F)(F)Oc1ccc2c(c(nn2CC3CCCCC3)C(=O)N[C@@H](C(C)(C)C)C(=O)NCC(=O)N)c1	9.32	9.3
167	O=C(NCCO)[C@@H](NC(=O)c1c2cc(OC)ccc2n(n1)CC3CCCCC3)C(C)(C)C	9.12	9
168	O=C(N)[C@@H](NC(=O)c1c2cc(OC)ccc2n(n1)CC3CCCCC3)C(C)(C)C	8.97	8.9
169	O[C@@H](CNC(=O)[C@@H](NC(=O)c1c2ccccc2n(n1)CC3CCOCC3)C(C)(C)C)CO	7.83	7.8
170	FC(F)(F)c1c(CNC(=O)c2c3ccccc3n(n2)CC4CCOCC4)cc(o1)C	8.76	8.8
171	Fc1cccc2c1n(nc2C(=O)N[C@@H](C(C)(C)C)C(=O)NCC3CCCCC3)CC4CCCCC4	7.63	7.4
172	Fc1cccc2c(nn(CC3CCCCC3)c12)C(=O)N[C@@H](C(C)(C)C)C(=O)NCCc4ccccc4	8.4	8.4
173	Clc1ccc2c(c(nn2CCCCC#N)C(=O)N[C@@H](C(C)(C)C)C(=O)N)c1	8.25	8.4
174	Clc1ccc2c(c(nn2CCCCC#N)C(=O)N[C@@H](C(C)(C)C)C(=O)NCCO)c1	7.79	7.5
175	Clc1ccc2c(c(nn2CCCCC#N)C(=O)N[C@@H](C(C)(C)C)C(OC)=O)c1	8.36	8.1
176	O=C(N[C@@H](C(C)(C)C)C(OC)=O)c1c2cccnc2n(n1)CC3CCOCC3	8.29	8.5
177	Fc1cccc2c(nn(CC3CCCCC3)c12)C(=O)N[C@H](C(=O)NCc4ccc(cc4)C(OC)=O)C(C)(C)C	7.78	7.7
178	O=C(OC)[C@@H](NC(=O)c1c2ccccc2n(n1)CC3CCOCC3)C(C)(C)C	9.23	9.5
179	Fc1cccc2c(nn(CC3CCCCC3)c12)C(=O)N[C@H](C(=O)NCc4cccc(OC)c4)C(C)(C)C	8.44	8.6
180	Fc1cccc2c(nn(CC3CCCCC3)c12)C(=O)N[C@H](C(=O)NCc4ccc(OC)cc4)C(C)(C)C	8.61	8.5
181	O=C(NC1CC1)[C@@H](NC(=O)c2c3ccccc3n(n2)CC4CCCCC4)C(C)(C)C	9.55	9.5
182	O=C(NC)[C@@H](NC(=O)c1c2ccccc2n(n1)CC3CCCCC3)C(C)(C)C	9.71	9.4
183	O=C(NCCC([O-])=O)[C@@H](NC(=O)c1c2ccccc2n(n1)CCCC#N)C(C)(C)C	7.72	7.6
184	O=C(N[C@@H](C(C)(C)C)c1nnc(o1)N)c2c3ccccc3n(n2)CCCCC#N	9.89	9.8
185	O=C(NCC(=O)N)[C@@H](NC(=O)c1c2ccccc2n(n1)CC3CCCCC3)C(C)(C)C	8.94	9
186	O=C(NCCO)[C@@H](NC(=O)c1c2ccccc2n(n1)CC3CCCCC3)C(C)(C)C	9.73	9.7
187	Fc1ccc2c(nn(CC3CCCCC3)c2c1)C(=O)N[C@H](C(=O)NC4CC4)C(C)(C)C	9.32	9
188	O=C(NC1CC1)[C@@H](NC(=O)c2c3ccccc3n(n2)CC4CCOCC4)C(C)(C)C	8.63	8.8
189	O=C(NC1CC1)[C@@H](NC(=O)c2c3ccccc3n(n2)CCCCC#C)C(C)(C)C	8.02	7.9
190	O=C(NCC(=O)N)[C@@H](NC(=O)c1c2ccccc2n(n1)CC3CCOCC3)C(C)(C)C	7.91	7.8
191	Fc1ccc2c(n(nc2C(=O)N[C@@H](C(C)(C)C)C(=O)NCCCO)CC3CCOCC3)c1	9.04	9
192	O=C(NC1CCC1)[C@@H](NC(=O)c2c3ccccc3n(n2)CCCCC#C)C(C)(C)C	7.84	7.7
193	Fc1ccc2c(n(nc2C(=O)N[C@@H](C(C)(C)C)C(=O)NCC(=O)N)CC3CCOCC3)c1	8.17	8.3
194	Fc1ccc2c(n(nc2C(=O)N[C@@H](C(C)(C)C)C(=O)N)CC3CCOCC3)c1	8.32	8.3
195	O=C(NCCO)[C@@H](NC(=O)c1c2ccccc2n(n1)CCCC#N)C(C)(C)C	8.02	8.1
196	Fc1ccc2c(n(nc2C(=O)N[C@@H](C(C)(C)C)C(=O)NCC(=O)N)CCCCC#N)c1	9.19	9
197	Clc1ccc(CC(=O)c2c3ccccc3n(CCCCC)c2C)cc1	5.8	5.9
198	O=C(c1c2ccccc2n(CCC)c1C)c3cccc4ccc(cc43)C	6.46	6.2
199	OC[C@@H]([N+]CCCC/C=C\C/C=C\C/C=C\C/C=C\CCCCC)C	6.86	6.8
200	O=C(N[C@@H](Cc1ccccc1)C(=O)N)c2c3ccccc3n(n2)CCCCC#N	5.61	5.5
201	O=C(c1c2ccccc2n(c1C)CCC)c3ccc(c4ccccc43)C	6.91	7
202	Fc1ccc(-c2cc(cn2CCCCC)C(=O)c3cccc4ccccc43)cc1	7.39	7.5
203	O=C(c1c2ccccc2n(CCCCC)c1)Cc3ccc(OC)cc3	5.97	6.5
204	Fc1cccc(CC(=O)c2c3ccccc3n(CCCCC)c2)c1	7.14	7.2
205	Clc1ccccc1CC(=O)c2c3ccccc3n(CCCCC)c2C	7.89	7.9
206	O=C(N[C@@H]1c2ccccc2CCC1)c3c4cccnc4n(n3)CC5CCOCC5	7.67	7.7

207	O=C(c1cn(c2ccccc21)CCC)c3ccc(OC)c4ccccc43	7.2	7.1
208	O=C(c1cn(C[C@H]2CCCC[N+]2C)c3ccccc31)c4cccc5ccccc54	8.41	8.1
209	O=C(c1c2ccccc2n(CCCCC)c1)Cc3ccc(C)cc3	6.75	6.6
210	Clc1cccc(CC(=O)c2c3ccccc3n(CCCCC)c2)c1	7.42	7.1
211	O=C(Nc1c(C)c(no1)C)c2c3ccccc3n(n2)CC4CCOCC4	7.34	7.2
212	Clc1ccc(C(=O)c2c3ccccc3n(CCCCC)c2C)c4ccccc14	8.05	7.9
213	FCCCCCn1cc(C(=O)C2C(C2(C)C)(C)C)c3ccccc31	7.53	7.6
214	Fc1ccccc1CC(=O)c2c3ccccc3n(CCCCC)c2	7.64	7.7
215	O=C(c1c2ccccc2n(CCCC)c1C)c3ccc(OC)c4ccccc43	7.47	7.7
216	O=C(c1c2ccccc2n(CCCCC)c1C)Cc3ccccc3OC	7.6	7.8
217	O=C(Oc1cccc2ccccc12)c3c4ccccc4n(n3)CCCCC	8.44	8.4
218	O=C(c1cc(n(CCCCC)c1)-c2ccc3ccccc3c2)c4cccc5ccccc54	6.48	6.5
219	Clc1ccccc1CC(=O)c2c3ccccc3n(CCCCC)c2	8.1	8.1
220	O=C(c1cn(CC[N+]2CCOCC2)c3ccccc31)c4ccc(c5ccccc54)C	8.22	8.3
221	FCCCCCn1cc(c2ccccc21)C(=O)Nc3ccc4ccccc4c3	6.63	6.9
222	O=C(N)[C@@H](NC(=O)c1c2ccccc2n(n1)CCCCC)C(C)(C)C	9.23	8.6
223	O=C(c1cn(CCCCC)c(-c2ccccc2)c1)c3cccc4ccccc43	7.85	7.9
224	O=C(c1cn(CCCCC)c(-c2cccc(OC)c2)c1)c3cccc4ccccc43	7.28	7.1
225	O=C(c1cn(CCCC)c2ccccc21)c3ccc(OC)c4ccccc43	8.25	8
226	O=C(c1cn(CCCCC)c2ccccc21)c3ccc(c4ccccc43)CCC	9.19	8.9
227	O=C(NC(c1ccccc1)(C)C)c2c3ccccc3n(CCCCC)c2	7.23	7.7
228	O=C(NC(c1ccccc1)(C)C)c2c3ccccc3n(CCCCC)c2	8.49	8.3
229	O=C(N)[C@@H](NC(=O)c1c2ccccc2n(n1)CC3CCCCC3)C(C)C	9.11	8.8
230	O=C(NCCCO)[C@@H](NC(=O)c1c2ccccc2n(n1)C[C@H]3CCC(O3)=O)C(C)(C)C	8.84	9
231	O=C(N[C@@H](C(C)(C)C)C(=O)N)c1c2cc(ccc2n(n1)CCCCC#N)C	8.24	7.9
232	O=C(c1cn(CCCCCC)c2ccccc21)c3cccc4ccccc43	8.01	8
233	FCCCCCn1cc(c2ccccc21)C(=O)c3cccc4ccccc43	9	8.6
234	O=C(NCCCO)[C@@H](NC(=O)c1c2ccccc2n(n1)CC3CCCCC3)C(C)C	7.82	8
235	O=C(NC1CCC1)[C@@H](NC(=O)c2c3ccccc3n(n2)CC4CCOCC4)C(C)(C)C	8.18	8.5
236	O=C(NCCO)[C@@H](NC(=O)c1c2ccccc2n(n1)CC3CCOCC3)C(C)(C)C	8.64	8.5
237	Fc1ccc2c(n(nc2C(=O)N[C@@H](C(C)(C)C)C(=O)NCCCO)CCCCC#N)c1	8.7	8.8
238	O=C(NC1CC1)[C@@H](NC(=O)c2c3cc(OC)ccc3n(n2)CC4CCCCC4)C(C)(C)C	8.35	8.5
239	Fc1ccc2c(c(nn2CC3CCCCC3)C(=O)N[C@@H](C(C)(C)C)C(=O)N)c1	8.79	8.6
240	Fc1cccc2c1n(nc2C(=O)N[C@@H](C(C)(C)C)C(=O)NC3CC3)CCCCC#C	8.55	8.7
241	O=C(NCCC([O-])=O)[C@@H](NC(=O)c1c2ccccc2n(n1)CCCCC#N)C(C)(C)C	9.13	8.9
242	Fc1cccc2c(nn(CC3CCCCC3)c12)C(=O)N[C@H](C(=O)NCc4ccc(cc4)C#N)C(C)(C)C	7.83	7.9
243	Brc1ccc(c2ccccc12)C(=O)c3cn(CCCCC)c4ccccc43	8.92	9
244	O=C(NCCCO)[C@@H](NC(=O)c1c2ccccc2n(n1)CC3CCCCC3)C(C)(C)C	9.27	9.1
245	Clc1ccc2c(c(nn2CC3CCOCC3)C(=O)N[C@@H](C(C)(C)C)C(=O)NCCO)c1	8.6	8.6
246	O=C(N)[C@@H](NC(=O)c1c2ccccc2n(n1)CC(=O)C(C)(C)C)C(C)(C)C	7.98	8.3
247	Clc1ccc2c(c(nn2CC3CCOCC3)C(=O)N[C@@H](C(C)(C)C)C(=O)NCC(=O)N)c1	7.76	7.8
248	Fc1ccc2c(c(nn2CC3CCCCC3)C(=O)N[C@@H](C(C)(C)C)C(=O)NCCO)c1	8.88	9

249	Fc1ccc2c(c(nn2CC3CCOCC3)C(=O)N[C@@H](C(C)(C)C)C(=O)NC4CC4)c1	9.47	9.8
250	Fc1cccc2c(nn(CC3CCCCC3)c12)C(=O)N[C@H](C(=O)NCc4ccc(cc4)C([O-])=O)C(C)(C)C	8.39	8.4

Table S2. SMILES, experimental and predicted p*K*i values of the molecules in the test set for CB_1 receptor.

		pKi	
**N°**	**SMILES**	**Exp**	**Pred**
1	O=C(c1c2ccccc2n(CCCCC)c1C)c3cccc4ccccc43	8.02	7.7
2	O=C(c1c2ccccc2n(CCCC)c1C)c3cccc4ccccc43	7.66	6.9
3	O=C(c1c2ccccc2n(c1C)CC)c3cccc4ccccc43	5.93	5.6
4	O=C(c1cn(c2ccccc21)C)c3cccc4ccccc43	5	5.5
5	O=C(c1c2ccccc2n(CCCCC)c1C)c3ccc(OC)c4ccccc43	8.35	7.7
6	O=C(c1cn(c2ccccc21)CCC)c3ccc(CCCC)c4ccccc34	6.47	7.1
7	O=C(c1c2ccccc2n(CCCCC)c1C)c3ccc(CCCC)c4ccccc34	7.38	7
8	Brc1ccc(c2ccccc12)C(=O)c3cn(c4ccccc43)CCC	6.79	7.2
9	Fc1ccc(C(=O)c2c3ccccc3n(CCCCC)c2C)c4ccccc14	7.85	8
10	O=C(c1c2ccccc2n(n1)CCCCC)c3cccc4ccccc43	8.23	7.7
11	Fc1ccc(Cn2cc(c3ccccc32)C(Oc4cccc5ccccc45)=O)cc1	8.92	8.2
12	O=C(NC12CC3CC(C1)CC(C2)C3)c4c5ccccc5n(CCCCC)c4	8.19	8
13	FCCCCCn1c2ccccc2c(n1)C(=O)NC34CC5CC(C3)CC(C4)C5	8.71	8.7
14	O=C(c1c2ccc([N+]([O-])=O)cc2n(C[C@H]3CCCC[N+]3C)c1C)c4cccc5ccccc54	7.28	7.1
15	O=C(N)[C@@H](NC(=O)c1c2ccccc2n(CCCCC)c1)C(C)(C)C	8.89	8.5
16	O=C(N)[C@@H](NC(=O)c1c2ccccc2n(n1)CC3CCCCC3)C(C)C	9.11	9.2
17	Fc1ccc(Cn2c3ccccc3c(n2)C(=O)N[C@@H](C(C)C)C(OC)=O)cc1	8	8.9
18	F[C@@H](Cn1cc(C(=O)C2C(C2(C)C)(C)C)c3ccccc31)CCC	7.23	6.8
19	O=C(c1cn(CCCCCC)c(-c2ccccc2)c1)c3cccc4ccccc43	7.96	8.1
20	Clc1cccc(-c2cc(cn2CCCCC)C(=O)c3cccc4ccccc43)c1	7.15	7.4
21	FC(F)(F)c1ccc(-c2cc(cn2CCCCC)C(=O)c3cccc4ccccc43)cc1	6.66	6.7
22	O=C(c1cn(CCCCC)c(-c2ccc(CC)cc2)c1)c3cccc4ccccc43	7.47	7
23	O=C(c1cn(c(-c2ccccc2CC)c1)CCCCC)c3cccc4ccccc43	7.77	7.9
24	O=C(c1cn(c(-c2ccccc2C)c1)CCCCC)c3cccc4ccccc43	8.25	7.2
25	Clc1ccc(CC(=O)c2c3ccccc3n(CCCCC)c2)cc1	6.41	6.3
26	O=C(c1c2ccccc2n(CCCCC)c1C)Cc3ccc(C)cc3	6.13	6
27	O=C(c1c2ccccc2n(CCCCC)c1C)Cc3cccc(OC)c3	7.21	6.5
28	Fc1ccccc1CC(=O)c2c3ccccc3n(CCCCC)c2C	7.41	7.7
29	O=C(OC)[C@H](NC(=O)c1c2ccccc2n(CCCCC)c1)C(C)C	7.82	8
30	ClCCCCCn1c2ccccc2c(n1)C(=O)N[C@@H](C(C)C)C(=O)N	8.39	8.2
31	FCCCCCn1c2ccccc2c(n1)C(=O)N[C@@H](C(C)(C)C)C(=O)N	8.84	8.9
32	FCCCCCn1c2ccccc2c(n1)C(=O)N[C@H](Cc3ccccc3)C(=O)N	6.9	7.1
33	O=C(c1c2ccccc2n(CC[N+]3CCOCC3)c1C)c4ccc(OC)cc4	5.65	5.4
34	O=C(OCC)[C@@H]1CCCC[C@@H]1NC(=O)c2c3ccccc3n(n2)CC4CCCCC4	8.42	8.5

35	O=C(N[C@@H](Cc1ccccc1)C(=O)N)c2c3ccccc3n(n2)CC4CCOCC4	6.56	6.8
36	Fc1cccc2c1n(nc2C(=O)N[C@@H](C(C)(C)C)C(=O)NCCCO)CCCCC#N	7.9	8.3
37	Fc1cccc2c1n(nc2C(=O)N[C@@H](C(C)(C)C)C(=O)N)CCCCC#N	8.59	7.5
38	OC[C@@H](NC(=O)c1c2ccccc2n(n1)CC3CCCCC3)C(C)(C)C	8.51	8.5
39	FC(F)(F)Oc1ccc2c(c(nn2CC3CCCCC3)C(=O)N[C@H](C(=O)NCCCO)C(C)(C)C)c1	8.06	8.3
40	O=C(N[C@@H](C(C)(C)C)C(=O)N)c1c2cccnc2n(n1)CCCCC#N	7.34	7.6
41	FC(F)(F)Oc1ccc2c(c(nn2CC3CCCCC3)C(=O)N[C@H](C(=O)NC4CC4)C(C)(C)C)c1	9.44	9
42	O=C(NCCCO)[C@@H](NC(=O)c1c2cc(OC)ccc2n(n1)CC3CCCCC3)C(C)(C)C	8.77	8.4
43	O=C(N[C@@H](C(C)(C)C)C(=O)NC1CC1)c2c3cc(ccc3n(n2)CCCCC#N)C	7.6	8.6
44	Fc1cccc2c(nn(CC3CCCCC3)c12)C(=O)N[C@H](C(=O)NCCCc4ccccc4)C(C)(C)C	8.61	8.7
45	Fc1cccc2c(nn(CC3CCCCC3)c12)C(=O)N[C@H](C(=O)NCc4cnccc4)C(C)(C)C	7.53	8.2
46	O=C(OC)[C@@H](NC(=O)c1c2ccccc2n(n1)CCCCC#N)C(C)(C)C	9.15	9
47	O=C(OC)[C@@H](NC(=O)c1c2ccccc2n(n1)CC(OC(C)(C)C)=O)C(C)(C)C	8.99	9.5
48	Fc1cccc2c(nn(CC3CCCCC3)c12)C(=O)N[C@H](C(=O)NCc4ccc(OC)cc4)C(C)(C)C	8.1	7.9
49	O=C(NC(C)C)[C@@H](NC(=O)c1c2ccccc2n(n1)CC3CCCCC3)C(C)(C)C	9.34	8.6
50	Fc1ccc2c(nn(CC3CCCCC3)c2c1)C(=O)N[C@@H](C(C)(C)C)C(=O)NCCO	9.27	9.3
51	O=C(NCCOC)[C@@H](NC(=O)c1c2ccccc2n(n1)CC3CCCCC3)C(C)(C)C	9.21	9.1
52	Fc1ccc2c(n(nc2C(=O)N[C@@H](C(C)(C)C)C(=O)NCCO)CC3CCOCC3)c1	7.81	7.4
53	O=C(NCCO)[C@@H](NC(=O)c1c2ccccc2n(n1)CCCCC#C)C(C)(C)C	8.45	8.1
54	FC(F)(F)CCCCc1cc(O)c2c(OC([C@@H]3CC=C(C[C@@H]23)C)(C)C)c1	7.7	6.5
55	O=C(c1cn(CCCCC)cc1)c2cccc3ccccc32	7.06	7.5
56	O=C(c1cn(CCCCC)c(-c2cccc([N+]([O-])=O)c2)c1)c3cccc4ccccc43	7	8
57	OC[C@@H](NC(=O)c1c2ccccc2n(n1)CC3CCCCC3)C(C)C	7.31	8.4
58	Fc1cccc2c1n(nc2C(=O)N[C@@H](C(C)(C)C)C(=O)N)CC3CCOCC3	7.56	7
59	O=C(OC)[C@@H](NC(=O)c1c2ccccc2n(n1)CC3CCCCC3)C(C)(C)C	9.87	9.4
60	Fc1ccc2c(nn(CC3CCCCC3)c2c1)C(=O)N[C@@H](C(C)(C)C)C(=O)N	8.32	9.2
61	Fc1ccc2c(n(nc2C(=O)N[C@@H](C(C)(C)C)C(=O)NC3CC3)CC4CCOCC4)c1	8.15	8
62	O=C(Oc1cccc2cccnc21)c3c4ccccc4n(CC5CCCCC5)c3	9.66	8.7

Table S3. SMILES, experimental and predicted pK_i values of the molecules in the training set for CB_2 receptor.

		pK_i	
**N°**	**SMILES**	**Exp**	**Pred**
1	O=C(c1c2ccccc2n(CCCCCC)c1C)c3cccc4ccccc43	8.4	8.2
2	O=C(c1cn(CCCCC)c2ccccc21)c3cccc4ccccc43	8.53	8.1
3	O=C(c1c(n(c2ccccc21)C)C)c3cccc4ccccc43	5.3	5.3
4	O=C(c1c2ccccc2n(CCCC)c1C)c3cccc4ccc(cc43)C	8.46	8.8
5	O=C(c1c2ccccc2n(CCCCCC)c1C)c3cccc4ccc(cc43)C	7.49	7.8
6	O=C(c1cn(CCCC)c2ccccc21)c3cccc4ccccc43	7.42	7.4
7	O=C(c1cn(CCCCC)c2ccccc21)c3ccc(OC)c4ccccc43	7.91	8.1
8	O=C(c1c2ccccc2n(c1CCCC)CCC)c3ccc(OC)c4ccccc43	7.23	7.3

9	O=C(c1c2ccccc2n(c1CCCCCC)CCCCC)c3ccc(OC)c4ccccc43	6.92	6.6
10	O=C(c1c2ccccc2n(CCCCC)c1)c3cccc4ccc(OC)cc43	8.16	8.1
11	O=C(c1cn(c2ccccc21)CCC)c3ccc(c4ccccc43)CCC	8.02	8.2
12	O=C(c1c2ccccc2n(c1C)CCC)c3ccc(c4ccccc34)CCC	7.92	7.9
13	O=C(c1c2ccccc2n(c1C)CCC)c3ccc(c4ccccc34)CC	7.92	7.6
14	O=C(c1cn(c2ccccc21)CCC)c3ccc(c4ccccc34)CC	8	7.7
15	O=C(c1c2ccccc2n(CCCCC)c1C)c3ccc(c4ccccc34)CC	9.38	9
16	O=C(c1c2ccccc2n(CCC)c1C)c3cccc4ccc(CC)cc43	6.62	6.7
17	O=C(c1c2ccccc2n(c1C)CCC)c3ccc(CCCC)c4ccccc34	7.31	7.5
18	O=C(c1c2ccccc2n(CCCCC)c1C)c3cccc4ccc(CC)cc43	8.25	8.5
19	Clc1ccc(C(=O)c2c3ccccc3n(c2C)CCC)c4ccccc14	7.66	7.6
20	Clc1ccc(c2ccccc12)C(=O)c3cn(c4ccccc43)CCC	7.36	7.3
21	Fc1ccc(c2ccccc12)C(=O)c3cn(CCCCC)c4ccccc43	8.49	8
22	Fc1ccc(c2ccccc12)C(=O)c3cn(c4ccccc43)CCC	7.48	7.3
23	Fc1ccc(C(=O)c2c3ccccc3n(c2C)CCC)c4ccccc14	7.42	7.4
24	FCCCCCn1cc(c2ccccc21)C(=O)c3ccc(c4ccccc43)C	9.24	9.1
25	F[C@@H](CCCn1cc(c2ccccc21)C(=O)c3ccc(c4ccccc43)C)C	9.09	9.4
26	FCCCCCn1cc(c2ccccc21)C(=O)c3ccc(c4ccccc34)CC	9.43	9.3
27	FCCCCCn1c2ccccc2c(n1)C(=O)c3cccc4ccccc43	8.88	8.5
28	FCCCCCn1cc(c2ccccc21)C(Oc3cccc4cccnc43)=O	9.2	9.4
29	Fc1ccc(Cn2cc(c3ccccc32)C(Oc4cccc5cccnc54)=O)cc1	9.32	9.2
30	FCCCCCn1c2ccccc2c(n1)C(Oc3cccc4ccccc34)=O	8.47	8.6
31	FCCCCCn1cc(c2ccccc21)C(=O)NC34CC5CC(C3)CC(C4)C5	9.1	9.2
32	O=C(NCc1ccccc1)c2c3ccccc3n(CCCCC)c2	6.73	6.8
33	FCCCCCn1cc(c2ccccc21)C(=O)NCc3ccccc3	6.37	6.5
34	O=C(Nc1cccc2ccccc21)c3cn(CCCCC)c4ccccc43	7.66	7.7
35	FCCCCCn1cc(c2ccccc21)C(=O)Nc3cccc4ccccc43	7.87	7.6
36	ClCCCCCn1cc(c2ccccc21)C(=O)Nc3cccc4ccccc43	7.87	7.6
37	Fc1ccc(Cn2c3ccccc3c(n2)C(=O)NC45CC6CC(C4)CC(C5)C6)cc1	9.76	9.3
38	O=C(Nc1cccc2ccccc21)c3c4ccccc4n(n3)CCCCC	8.46	8
39	O=C(c1c2ccccc2n([C@H](CCCCC)C)c1C)c3cccc4ccccc43	8.4	8.8
40	Brc1cccc2cccc(c12)C(=O)c3cn(CCCCC)c4ccccc43	8.26	8
41	Fc1ccc(Cn2c3ccccc3c(n2)C(=O)N[C@@H](C(C)(C)C)C(OC)=O)cc1	9.92	10.1
42	O=C(C12CC3CC(C1)CC(C2)C3)c4c5ccccc5n(CCCCC)c4	8.76	8.7
43	FCCCCCn1c2ccccc2c(n1)C(=O)C3C(C3(C)C)(C)C	9.35	9.2
44	FC(F)(F)CCCn1cc(C(=O)C2C(C2(C)C)(C)C)c3ccccc31	9.08	9.1
45	O=C(c1cn(CCCC)c(-c2ccccc2)c1)c3cccc4ccccc43	7.82	7.7
46	O=C(c1cn(CCCCC)c(-c2ccc(cc2)C)c1)c3cccc4ccccc43	7.74	7.7
47	Clc1ccc(-c2cc(cn2CCCCC)C(=O)c3cccc4ccccc43)cc1	7.6	7.2
48	O=C(c1cn(CCCCC)c(-c2ccccc2OC)c1)c3cccc4ccccc43	7.7	8
49	Fc1ccccc1-c2cc(cn2CCCCC)C(=O)c3cccc4ccccc43	8.48	8.3
50	O=C(c1cn(CCCCC)c(-c2cccc(c2)C)c1)c3cccc4ccccc43	7.41	7.3

51	O=C(c1cn(CCCCC)c(-c2ccc(CCCC)cc2)c1)c3cccc4ccccc43	7.19	7.5
52	O=C(c1cn(c(-c2ccccc2CCCC)c1)CCCCC)c3cccc4ccccc43	7.16	7.7
53	FC(F)(F)c1ccccc1-c2cc(cn2CCCCC)C(=O)c3cccc4ccccc43	7.62	7.8
54	Brc1ccc(CC(=O)c2c3ccccc3n(CCCCC)c2)cc1	6.18	6.2
55	Brc1ccccc1CC(=O)c2c3ccccc3n(CCCCC)c2	7.7	8
56	O=C(c1cn(CCCCC)c2ccccc21)Cc3ccccc3C	6.84	6.9
57	Clc1cccc(CC(=O)c2c3ccccc3n(CCCCC)c2C)c1	6.86	6.9
58	Brc1ccccc1CC(=O)c2c3ccccc3n(CCCCC)c2C	7.54	7.7
59	Fc1ccc(CC(=O)c2c3ccccc3n(CCCCC)c2)cc1	6.44	6.7
60	FCCCCCc1cc(O)c2c(OC([C@@H]3CC=C(C[C@@H]23)C)(C)C)c1	8.06	8.1
61	O[C@@H]1CC[C@H]([C@@H](C1)c2ccc(C(CCCCCC)(C)C)cc2O)CCCO	8.74	8.6
62	O[C@H]1CC[C@@H]([C@H](C1)c2ccc(C(CCCCCC)(C)C)cc2O)CCCO	7.63	7.5
63	O=C(NCCO)CCC/C=C\C/C=C\C/C=C\C/C=C\CCCCC	6.43	6.4
64	CCCCC/C=C\C/C=C\C/C=C\C/C=C\CCCC[N+]1CCOCC1	6.27	5.9
65	Clc1ccc(c2ccccc12)C(=O)c3cn(CCCCCF)c4ccccc43	8.93	8.7
66	O=C(OC)[C@@H](OC(=O)c1c2ccccc2n(n1)CC3CCCCC3)C(C)(C)C	8.95	9
67	FCCCCCn1cc(C(=O)N[C@@H](C(C)C)C(=O)N)c2ccccc21	7.05	7
68	O=C(OC)[C@@H](NC(=O)c1c2ccccc2n(CC3CCCCC3)c1)C(C)(C)C	9.46	9.4
69	FCCCCCn1cc(c2ccccc21)C(=O)N[C@H](C(C)C)C(OC)=O	7.7	7.9
70	O=C(NC(c1ccccc1)(C)C)c2c3ccccc3n(n2)CC4CCOCC4	8.86	9.2
71	Fc1cccc(Cn2c3ccccc3c(n2)C(=O)N[C@@H](C(C)C)C(=O)N)c1	7.28	7.5
72	FCCCCCn1c2ccccc2c(n1)C(=O)N[C@@H](C(C)C)C(=O)N	8.42	8.4
73	FCCCCCn1c2ccccc2c(n1)C(=O)N[C@H]([C@H](CC)C)C(=O)N	8.37	8.4
74	O=C(N)[C@@H](NC(=O)c1c2ccccc2n(n1)CC3CCCCC3)C(C)(C)C	9.48	9.5
75	O=C(OC)[C@H](NC(=O)c1c2ccccc2n(n1)CC3CCCCC3)C(C)C	9.52	9.4
76	Ic1ccccc1C(=O)c2c3ccccc3n(CCCCC)c2	7.31	8
77	O=C(N1CC[N+](CC1)C)c2c3ccccc3n(CCCCC)c2	5.73	5.6
78	O=C(OC)[C@@H](NC(=O)c1ccc2c(c3ccccc3n2CC4CCCCC4)c1)C(C)(C)C	8.18	8.7
79	O=C(c1ccc2c(c3ccccc3n2CCCCC)c1)c4cccc5ccccc54	8.64	8.6
80	FCCCCCn1c2ccc(cc2c3ccccc31)C(=O)c4cccc5ccccc54	8.36	8.1
81	O=C(c1c2ccccc2n(CCC)c1C)c3cccc4ccc(cc43)C	7.79	7.5
82	Fc1ccc(-c2cc(cn2CCCCC)C(=O)c3cccc4ccccc43)cc1	7.48	7.6
83	O=C(c1c2ccccc2n(CCCCC)c1)Cc3ccc(OC)cc3	6.35	6.5
84	Fc1cccc(CC(=O)c2c3ccccc3n(CCCCC)c2)c1	7.04	6.9
85	O=C(c1cn(c2ccccc21)CCC)c3ccc(OC)c4ccccc43	7.49	7.7
86	O=C(c1cn(C[C@H]2CCCC[N+]2C)c3ccccc31)c4cccc5ccccc54	7.13	7.4
87	O=C(c1c2ccccc2n(CCCCC)c1)Cc3ccc(C)cc3	6.24	6.3
88	FCCCCCn1cc(C(=O)C2C(C2(C)C)(C)C)c3ccccc31	9.22	9.3
89	O=C(c1c2ccccc2n(CCCCC)c1C)Cc3ccccc3OC	7.09	7.2
90	O=C(Oc1cccc2ccccc12)c3c4ccccc4n(n3)CCCCC	8.58	8.4
91	O=C(c1cc(n(CCCCC)c1)-c2ccc3ccccc3c2)c4cccc5ccccc54	6.77	7.1
92	Clc1ccccc1CC(=O)c2c3ccccc3n(CCCCC)c2	8.15	7.7

93	O=C(c1cn(CCCCC)c(-c2cccc(OC)c2)c1)c3cccc4ccccc43	7.64	7.4
94	O=C(c1cn(CCCC)c2ccccc21)c3ccc(OC)c4ccccc43	8.66	8.4
95	O=C(NC(c1ccccc1)(C)C)c2c3ccccc3n(CCCCC)c2	7.62	7.7
96	O=C(c1cn(CCCCCC)c2ccccc21)c3cccc4ccccc43	8.26	8.1
97	FCCCCCn1cc(c2ccccc21)C(=O)c3cccc4ccccc43	8.59	8.5
98	Brc1ccc(c2ccccc12)C(=O)c3cn(CCCCC)c4ccccc43	8.96	8.7
99	O=C(c1c2ccccc2n(CCCCC)c1C)c3cccc4ccccc43	8.53	8.8
100	O=C(c1c2ccccc2n(c1C)CC)c3cccc4ccccc43	6.02	5.7
101	O=C(c1cn(c2ccccc21)C)c3cccc4ccccc43	5	5.4
102	O=C(c1c2ccccc2n(CCCCC)c1C)c3ccc(OC)c4ccccc43	8.73	8.9
103	O=C(c1c2ccccc2n(CCCCC)c1C)c3ccc(CCCC)c4ccccc34	8.19	8.1
104	Brc1ccc(c2ccccc12)C(=O)c3cn(c4ccccc43)CCC	7.57	7.2
105	Fc1ccc(C(=O)c2c3ccccc3n(CCCCC)c2C)c4ccccc14	8.66	8.4
106	O=C(c1c2ccccc2n(n1)CCCCC)c3cccc4ccccc43	8.34	8.5
107	FCCCCCn1c2ccccc2c(n1)C(=O)NC34CC5CC(C3)CC(C4)C5	9.58	9.4
108	Fc1ccc(Cn2c3ccccc3c(n2)C(=O)N[C@@H](C(C)C)C(OC)=O)cc1	9.1	9.4
109	F[C@@H](Cn1cc(C(=O)C2C(C2(C)C)(C)C)c3ccccc31)CCC	8.74	8.7
110	O=C(c1cn(CCCCCC)c(-c2ccccc2)c1)c3cccc4ccccc43	8.15	8.4
111	Clc1cccc(-c2cc(cn2CCCCC)C(=O)c3cccc4ccccc43)c1	7.8	7.7
112	FC(F)(F)c1ccc(-c2cc(cn2CCCCC)C(=O)c3cccc4ccccc43)cc1	7.28	7.5
113	O=C(c1cn(CCCCC)c(-c2ccc(CC)cc2)c1)c3cccc4ccccc43	7.54	7.6
114	O=C(c1cn(c(-c2ccccc2C)c1)CCCCC)c3cccc4ccccc43	8.4	8.6
115	O=C(c1c2ccccc2n(CCCCC)c1C)Cc3ccc(C)cc3	5.87	6.4
116	Fc1ccccc1CC(=O)c2c3ccccc3n(CCCCC)c2C	7.12	7.3
117	ClCCCCCn1c2ccccc2c(n1)C(=O)N[C@@H](C(C)C)C(=O)N	7.92	8
118	FCCCCCn1c2ccccc2c(n1)C(=O)N[C@H](Cc3ccccc3)C(=O)N	7.76	7.6
119	O=C(c1cn(CCCCC)c(-c2cccc([N+]([O-])=O)c2)c1)c3cccc4ccccc43	7.39	7.2
120	O=C(OC)[C@@H](NC(=O)c1c2ccccc2n(n1)CC3CCCCC3)C(C)(C)C	9.65	9.3
121	O=C(Oc1cccc2cccnc21)c3c4ccccc4n(CC5CCCCC5)c3	9.47	9.3
122	O=C(C1C(C1(C)C)(C)C)c2c3ccccc3n(CC4CCOCC4)c2	9.25	9.4
123	O=C(N)[C@@H](NC(=O)c1c2ccccc2n(n1)CCCCC)C(C)C	9.06	8.5
124	FCCCCCn1cc(c2ccccc21)C(=O)c3ccc(F)c4ccccc43	8.72	8.7
125	Clc1ccc(C(=O)c2c3ccccc3n(CCCCC)c2C)c4ccccc14	8.64	8.8
126	O=C(c1c2ccccc2n(CCCCC)c1)c3ccccc3OC	8.54	8.7
127	O=C(c1cn(c(-c2ccccc2CC)c1)CCCCC)c3cccc4ccccc43	8.47	8.1
128	O=C(c1c2ccccc2n(CCCCC)c1)c3cccc4ccc(CC)cc34	8.42	8.5
129	O=C(c1c2ccccc2n(CCCC)c1C)c3cccc4ccccc43	8.37	8.1
130	Clc1ccccc1-c2cc(cn2CCCCC)C(=O)c3cccc4ccccc43	8.28	8.3
131	O=C(c1cn(CCCCC)c(-c2ccccc2)c1)c3cccc4ccccc43	8.19	8
132	FC(F)(F)c1ccccc1-c2cc(cn2CCCCC)C(=O)c3cccc4ccccc43	8.09	7.6
133	O=C(c1c2ccccc2n(CCCC)c1C)c3ccc(OC)c4ccccc43	7.88	8.1
134	O=C(OC)[C@H](NC(=O)c1c2ccccc2n(CCCCC)c1)C(C)C	7.85	7.8

135	O=C(c1c2ccccc2n(CCCCCC)c1C)c3ccc(OC)c4ccccc43	7.75	8.4
136	FCCCCCn1cc(c2ccc([N+]([O-])=O)cc21)C(=O)c3cccc4ccccc43	7.69	7.5
137	Clc1ccccc1CC(=O)c2c3ccccc3n(CCCCC)c2C	7.6	7.6
138	Brc1ccc(C(=O)c2c3ccccc3n(c2C)CCC)c4ccccc14	7.51	7.4
139	O=C(Cc1ccccc1OC)c2c3ccccc3n(CCCCC)c2	7.48	7.7
140	O=C(c1cn(CCCCC)c(-c2ccc(OC)cc2)c1)c3cccc4ccccc43	7.39	7.4
141	O=C(c1cc(n(CCCCC)c1)-c2cccc3ccccc32)c4cccc5ccccc54	7.31	7.4
142	O=C(c1cn(CCCCCCC)c(-c2ccccc2)c1)c3cccc4ccccc43	7.21	7.7
143	FC(F)(F)c1cccc(-c2cc(cn2CCCCC)C(=O)c3cccc4ccccc43)c1	7.15	7.3
144	O=C(c1c2ccccc2n(CCCCC)c1C)Cc3cccc(OC)c3	7.08	7.1
145	O=C(c1c2ccccc2n(c1C)CCC)c3ccc(OC)c4ccccc43	7.01	7.3
146	O=C(c1c2ccccc2n(CCCCC)c1C)Cc3ccccc3	6.74	6.5
147	O=C(c1c2ccccc2n(c1CCCCC)CCCC)c3ccc(OC)c4ccccc43	6.51	6.7
148	Clc1ccc(CC(=O)c2c3ccccc3n(CCCCC)c2)cc1	6.3	6.5
149	Fc1ccc(CC(=O)c2c3ccccc3n(CCCCC)c2C)cc1	6.11	6.2
150	Clc1ccc(CC(=O)c2c3ccccc3n(CCCCC)c2C)cc1	5.43	5.2

Table S4. SMILES, experimental and predicted pK_i values of the molecules in the test set for CB_2 receptor.

		**pK_i**	
**N°**	**SMILES**	**Exp**	**Pred**
1	O=C(c1c2ccccc2n(c1C)CCC)c3cccc4ccccc43	7.86	8
2	O=C(c1c2ccccc2n(CCCCC)c1C)c3cccc4ccc(cc43)C	9.31	7.9
3	O=C(c1cn(c2ccccc21)CC)c3cccc4ccccc43	5.53	6.8
4	O=C(c1c2ccccc2n(CCC)c1)c3cccc4ccc(cc34)C	6.97	7.1
5	O=C(c1cn(c2ccccc21)CC)c3ccc(OC)c4ccccc43	6.2	6.9
6	O=C(c1c2ccccc2n(CCC)c1)c3cccc4ccc(CC)cc34	6.91	7.9
7	Fc1ccccc1-n2c3ccccc3c(n2)C(Oc4ccc5ccccc5c4)=O	6.37	6.6
8	O=C(Nc1ccccc1)c2cn(CCCCC)c3ccccc32	6.56	6.3
9	FCCCCCn1cc(c2ccccc21)C(=O)NC(c3ccccc3)(C)C	7.1	8.5
10	O=C(C1C(C1(C)C)(C)C)c2c3ccccc3n(CCCCC)c2	8.83	8.3
11	FCCCCCn1c(c(C(=O)C2C(C2(C)C)(C)C)c3ccccc31)C	8.34	8.1
12	Fc1cccc(-c2cc(cn2CCCCC)C(=O)c3cccc4ccccc43)c1	8.04	8.1
13	O=C(c1c2ccccc2n(CCCCC)c1)Cc3ccccc3	6.8	6.8
14	O=C(c1c2ccccc2n(CCCCC)c1)Cc3cccc(OC)c3	7.05	7.1
15	Oc1cc(CCCCC)cc2c1-c3cc(ccc3C(O2)(C)C)C	7.02	7.4
16	CCCCC/C=C\C/C=C\C/C=C\C/C=C\CCCC[N+]CCOC	6.34	5.8
17	Fc1ccc(Cn2cc(c3ccccc32)C(=O)c4cccc5ccccc54)cc1	8.87	9.1
18	Fc1ccc(Cn2cc(c3ccccc32)C(=O)Nc4cccc5ccccc54)cc1	7.19	7.7
19	Fc1ccc(Cn2c3ccccc3c(n2)C(=O)N[C@@H](C(C)(C)C)C(=O)N)cc1	9.47	9.1
20	O=C(N[C@@H](Cc1ccccc1)C(=O)N)c2c3ccccc3n(n2)CC4CCCCC4	8.36	8.4

21	O=C(c1c2ccccc2n(c1C)CCC)c3ccc(c4ccccc43)C	7.85	7.4
22	Clc1cccc(CC(=O)c2c3ccccc3n(CCCCC)c2)c1	6.97	6.5
23	Fc1ccccc1CC(=O)c2c3ccccc3n(CCCCC)c2	7.41	7.5
24	FCCCCCn1cc(c2ccccc21)C(=O)Nc3ccc4ccccc4c3	6.65	7.2
25	O=C(c1cn(CCCCC)c2ccccc21)c3ccc(c4ccccc43)CCC	8.96	9.2
26	O=C(N)[C@@H](NC(=O)c1c2ccccc2n(n1)CC3CCCCC3)C(C)C	9.35	8.5
27	O=C(c1cn(c2ccccc21)CCC)c3ccc(CCCC)c4ccccc34	7.28	8
28	Fc1ccc(Cn2cc(c3ccccc32)C(Oc4cccc5ccccc45)=O)cc1	8.61	8.6
29	FC(F)(F)CCCCc1cc(O)c2c(OC([C@@H]3CC=C(C[C@@H]23)C)(C)C)c1	7.52	7.9
30	O=C(N)[C@@H](NC(=O)c1c2ccccc2n(n1)CC3CCCCC3)C(C)C	9.35	9.8
31	FCCCCCn1c2ccccc2c(n1)C(=O)N[C@@H](C(C)(C)C)C(=O)N	9.16	8.7
32	O=C(NC12CC3CC(C1)CC(C2)C3)c4c5ccccc5n(CCCCC)c4	8.91	8.3
33	O=C(c1cn(CCCCC#N)c2ccccc21)c3cccc4ccccc43	8.83	9.2
34	O=C(c1cn(CCCCC)c2ccccc21)c3ccc(OCC)c4ccccc43	7.98	8.4
35	FCCCCCn1c(nc2ccccc21)C(=O)c3cccc4ccccc43	7.63	7.5
36	O=C(NC(c1ccccc1)(C)C)c2c3ccccc3n(CCCCC)c2	6.87	7.5
37	OC[C@@H]([N+]CCCC/C=C\C/C=C\C/C=C\C/C=C\CCCCC)C	6.4	6.3

Table S5. List, SMILE and predicted pK_i values for Series 1 in CB_1 receptor.

N°	SMILES	Pred pK_i
1	O=C(c1cn(c2ccccc21)CSCC(C)C)c3cccc4ccccc43	9.3
2	Fc1ccc(cc1Cn2cc(c3ccccc32)C(=O)c4cccc5ccccc54)C(=O)N	9.2
3	OCc1ccncc1NCn2cc(c3ccccc32)C(=O)c4cccc5ccccc54	9.2
4	S=C(N)[C@@H]1CCC[N+](C1)Cn2cc(c3ccccc32)C(=O)c4cccc5ccccc54	9
5	Fc1c(F)cccc1Sn2cc(c3ccccc32)C(=O)c4cccc5ccccc54	9
6	OCCCSCn1cc(c2ccccc21)C(=O)c3cccc4ccccc43	9
7	FC(F)(F)CSCn1cc(c2ccccc21)C(=O)c3cccc4ccccc43	9
8	O=C(c1cn(NCCC(C)C)c2ccccc21)c3cccc4ccccc43	8.9
9	O=C(c1cn(c2ccccc21)CSCC3CC3)c4cccc5ccccc54	8.9
10	O=C(c1cn(C[N+]2CCCC(C2)(C)C)c3ccccc31)c4cccc5ccccc54	8.9
11	Clc1ccc(Cl)c(OCn2cc(c3ccccc32)C(=O)c4cccc5ccccc54)c1	8.9
12	O=C(c1cn(SCCCCC)c2ccccc21)c3cccc4ccccc43	8.9
13	Clc1cc(Cl)cc([C@@H](O)n2cc(c3ccccc32)C(=O)c4cccc5ccccc54)c1	8.8
14	O=C(c1cn([C@H]([N+])c2cc([N+]([O-])=O)ccc2C)c3ccccc13)c4cccc5ccccc54	8.8
15	O=C(c1cn(C[C@@H]2COCCC2)c3ccccc31)c4cccc5ccccc54	8.8
16	O=C(c1cn(C[C@H]2Cc3ccccc3C[N+]2C)c4ccccc41)c5cccc6ccccc65	8.8
17	Clc1cccc([C@@H]([N+])n2cc(c3ccccc32)C(=O)c4cccc5ccccc54)c1	8.8
18	O=C(c1cn(C[N+]2CCC[C@H](CC2)CC)c3ccccc31)c4cccc5ccccc54	8.8
19	Brc1csc(Cn2cc(c3ccccc32)C(=O)c4cccc5ccccc54)c1	8.7
20	O=C(c1cn(C[C@H]2CCCC[C@@H]2C)c3ccccc31)c4cccc5ccccc54	8.7
21	O=C(c1cn(C[N+]2CCC[C@H](C2)CC)c3ccccc31)c4cccc5ccccc54	8.7

22	FCCCCCn1cc(c2ccccc21)C(=O)c3cccc4ccccc43	8.7
23	FC(F)(F)CCSn1cc(c2ccccc21)C(=O)c3cccc4ccccc43	8.7
24	O=C(c1cn(CC[N+]C(C)(C)C)c2ccccc21)c3cccc4ccccc43	8.7
25	Brc1ccc(s1)Cn2cc(c3ccccc32)C(=O)c4cccc5ccccc54	8.7
26	O=C(c1cn(c2ccccc21)C[S@@](=O)CC)c3cccc4ccccc43	8.7
27	Clc1cc(ccc1Sn2cc(c3ccccc32)C(=O)c4cccc5ccccc54)C#N	8.7
28	Clc1cc(F)ccc1CSn2cc(c3ccccc32)C(=O)c4cccc5ccccc54	8.7
29	Clc1ccc(c([C@@H]([N+])n2cc(c3ccccc32)C(=O)c4cccc5ccccc54)c1)C	8.7
30	FC(F)(F)C[N+](C(C)C)Cn1cc(c2ccccc21)C(=O)c3cccc4ccccc43	8.7
31	O=C(c1cn(c2ccccc21)Cc3csc(n3)C[N+])c4cccc5ccccc54	8.6
32	Fc1ccc2CC[N+][C@@H](n3cc(c4ccccc43)C(=O)c5cccc6ccccc65)c2c1	8.6
33	Fc1ccc(O)c([C@@H]([N+])n2cc(c3ccccc32)C(=O)c4cccc5ccccc54)c1	8.6
34	O=S(=O)(Cn1cc(c2ccccc21)C(=O)c3cccc4ccccc43)CC#C	8.6
35	O=C(c1cn(c2ccccc21)C[C@@H](CC(C)C)C#N)c3cccc4ccccc43	8.6
36	O=C(c1cn(c2ccccc21)Cc3cccc4c3cccn4)c5cccc6ccccc65	8.6
37	O=C(c1cn(C[C@H]2C[C@@H](CC2)C)c3ccccc31)c4cccc5ccccc54	8.6
38	O=C(c1cn(Cc2cc(nn2C)C)c3ccccc31)c4cccc5ccccc54	8.6
39	Clc1ccc([N+]([O-])=O)c(Cn2cc(c3ccccc32)C(=O)c4cccc5ccccc54)c1	8.6
40	O=C(c1cn(c2ccccc21)Cc3ncc(s3)C)c4cccc5ccccc54	8.6
41	O=C(c1cn(c2ccccc21)Cc3cc(c(o3)C)C[N+])c4cccc5ccccc54	8.6
42	O[C@@H](n1cc(c2ccccc21)C(=O)c3cccc4ccccc43)c5cc(ccc5C)C	8.6
43	Fc1ccc(F)cc1Sn2cc(c3ccccc32)C(=O)c4cccc5ccccc54	8.6
44	O=C(c1cn(c2ccccc21)CC#CCC#C)c3cccc4ccccc43	8.6
45	O=C(c1cn(C[N+]2C[C@@H](C[C@@H](C2)C)C)c3ccccc31)c4cccc5ccccc54	8.6
46	O=C(c1cn(c2ccccc21)Cn3cc(nc3C)C)c4cccc5ccccc54	8.5
47	O=C(c1cn(OCCC(C)C)c2ccccc21)c3cccc4ccccc43	8.5
48	Clc1ccc(OC)c([C@@H]([N+])n2cc(c3ccccc32)C(=O)c4cccc5ccccc54)c1	8.5
49	O=C(c1cn(c2ccccc21)CSCCC[N+])c3cccc4ccccc43	8.5
50	O=C(c1cn(NCCCC)c2ccccc21)c3cccc4ccccc43	8.5
51	Fc1ccccc1Sn2cc(c3ccccc32)C(=O)c4cccc5ccccc54	8.5
52	O=C(c1cn(CCCCCC#C)c2ccccc21)c3cccc4ccccc43	8.5
53	O=C(c1cn(C[C@H]2CCC=CO2)c3ccccc31)c4cccc5ccccc54	8.5
54	Fc1cc(F)cc(On2cc(c3ccccc32)C(=O)c4cccc5ccccc54)c1	8.5
55	Fc1cc(F)cc([C@@H](O)n2cc(c3ccccc32)C(=O)c4cccc5ccccc54)c1	8.5
56	O=C(c1cn(c2ccccc21)CC#CCC)c3cccc4ccccc43	8.5
57	Fc1ccc(c(S(=O)(=O)n2cc(c3ccccc32)C(=O)c4cccc5ccccc54)c1)C	8.5
58	O=C(c1cn(c2ccccc21)Cc3csc4nccn43)c5cccc6ccccc65	8.5
59	O=C(c1cn([C@H]([N+])c2cccc(c2C)C)c3ccccc13)c4cccc5ccccc54	8.5
60	O=C(c1cn(C[N+]2CCC[C@H]([C@H]2C)C)c3ccccc31)c4cccc5ccccc54	8.5
61	Fc1ccc([N+]([O-])=O)c(Cn2cc(c3ccccc32)C(=O)c4cccc5ccccc54)c1	8.5
62	OCC[N+]CCn1cc(c2ccccc21)C(=O)c3cccc4ccccc43	8.5
63	O=C(c1cn(CC2([N+])CCCCC2)c3ccccc31)c4cccc5ccccc54	8.5

64	O=C(c1cn(Cc2cc(nn2C)CC)c3ccccc31)c4cccc5ccccc54	8.5
65	Oc1c(Cn2cc(c3ccccc32)C(=O)c4cccc5ccccc54)c(nc(O)n1)C	8.5
66	O=C(c1cn(c2ccccc21)Cc3cscc3C)c4cccc5ccccc54	8.5
67	O=C(c1cn([C@H](C[N+])c2cc(sc2C)C)c3ccccc31)c4cccc5ccccc54	8.5
68	O=C(c1cn(Nc2cscc2C)c3ccccc31)c4cccc5ccccc54	8.5
69	Clc1cn(nc1)Cn2cc(c3ccccc32)C(=O)c4cccc5ccccc54	8.5
70	O[C@@H](n1cc(c2ccccc21)C(=O)c3cccc4ccccc43)c5cscc5	8.5
71	O=C(c1cn(C[C@H]2CCCC[C@H]2[N+]CC)c3ccccc31)c4cccc5ccccc54	8.5
72	O=C(c1cn(C[C@@H]2[C@H]([N+]CCS2)C)c3ccccc31)c4cccc5ccccc54	8.5
73	O=C(c1cn(SS[C@@H](CC)C)c2ccccc21)c3cccc4ccccc43	8.5
74	O=C(c1cn(CCCCSC)c2ccccc21)c3cccc4ccccc43	8.4
75	O=C(c1cn([C@H]([N+])c2ccc(cc2C)C)c3ccccc13)c4cccc5ccccc54	8.4
76	O=C(c1cn(C[N+]2CCC[C@@H](CC2)C)c3ccccc31)c4cccc5ccccc54	8.4
77	O=C(c1cn(c2ccccc21)Cc3ccc[nH+]c3NCC)c4cccc5ccccc54	8.4
78	Cl/C=C/Cn1cc(c2ccccc21)C(=O)c3cccc4ccccc43	8.4
79	Fc1ccc(F)cc1[C@@H]([N+]C)n2cc(c3ccccc32)C(=O)c4cccc5ccccc54	8.4
80	O=S(=O)(Cn1cc(c2ccccc21)C(=O)c3cccc4ccccc43)CC	8.4
81	Cl/C=C(\Cl)Cn1cc(c2ccccc21)C(=O)c3cccc4ccccc43	8.4
82	Clc1ccc(F)c([C@@H]([N+])n2cc(c3ccccc32)C(=O)c4cccc5ccccc54)c1	8.4
83	O=C(c1cn(c2ccccc21)C/C=C/CC)c3cccc4ccccc43	8.4
84	O=C(c1cn([C@H]([N+])c2cc(sc2C)C)c3ccccc31)c4cccc5ccccc54	8.4
85	O=C(c1cn(c2ccccc21)Cn3ccnc3C)c4cccc5ccccc54	8.4
86	Fc1cccc([C@@H]([N+])n2cc(c3ccccc32)C(=O)c4cccc5ccccc54)c1	8.4
87	O=C(c1cn(Sc2ccccc2C[N+]C)c3ccccc31)c4cccc5ccccc54	8.4
88	Fc1cccc(F)c1[C@H](n2cc(c3ccccc32)C(=O)c4cccc5ccccc54)C[N+]	8.4
89	Brc1ccc(o1)Cn2cc(c3ccccc32)C(=O)c4cccc5ccccc54	8.4
90	Clc1ccnc(Sn2cc(c3ccccc32)C(=O)c4cccc5ccccc54)c1	8.4
91	Clc1cccc(F)c1C[C@@H]([N+])n2cc(c3ccccc32)C(=O)c4cccc5ccccc54	8.4
92	O=C(c1cn(SCCC#C)c2ccccc21)c3cccc4ccccc43	8.4
93	O=C(c1cn(CC[N+]CC#C)c2ccccc21)c3cccc4ccccc43	8.4
94	Fc1cc(c2CC[N+][C@@H](n3cc(c4ccccc43)C(=O)c5cccc6ccccc65)c2c1)C	8.4
95	O=C(c1cn(CC2C[C@H]3CC[C@H]([N+]3C)C2)c4ccccc41)c5cccc6ccccc65	8.4
96	O[C@H]1CCCC[C@@H]1Cn2cc(c3ccccc32)C(=O)c4cccc5ccccc54	8.4
97	O=C(c1cn(c2ccccc21)C[C@@](O)(CSC)C)c3cccc4ccccc43	8.4
98	Fc1ccc(c(Cn2cc(c3ccccc32)C(=O)c4cccc5ccccc54)c1)C[N+]	8.4
99	Clc1ccc(c(Cn2cc(c3ccccc32)C(=O)c4cccc5ccccc54)c1)C[N+]	8.4
100	O[C@@H](n1cc(c2ccccc21)C(=O)c3cccc4ccccc43)[C@H]5CCC[N+]C5	8.4
101	O=C(c1cn(C[C@@H]2COCC[N+]C2)c3ccccc31)c4cccc5ccccc54	8.4
102	Clc1ccc(Cl)cc1[C@@H]([N+])n2cc(c3ccccc32)C(=O)c4cccc5ccccc54	8.4
103	O=C(c1cn([C@H](C[N+])c2ccccc2C)c3ccccc31)c4cccc5ccccc54	8.4
104	Clc1cccc(F)c1[C@@H]([N+])n2cc(c3ccccc32)C(=O)c4cccc5ccccc54	8.4
105	O=C(c1cn(c2ccccc21)Cc3c(nc(s3)C)C)c4cccc5ccccc54	8.4

106	O=C(c1cn([C@H]([N+]C)c2cnccn2)c3ccccc31)c4cccc5ccccc54	8.4
107	O=C(c1cn(c2ccccc21)C/C=C/C(C)C)c3cccc4ccccc43	8.4
108	O=C(c1cn(Nc2cnccc2C[N+])c3ccccc31)c4cccc5ccccc54	8.4
109	O=C(c1cn(c2ccccc21)CSCC)c3cccc4ccccc43	8.3
110	Fc1ccccc1[C@@H](O)n2cc(c3ccccc32)C(=O)c4cccc5ccccc54	8.3
111	O=S(=O)(CC[N+])Cn1cc(c2ccccc21)C(=O)c3cccc4ccccc43	8.3
112	Clc1ccccc1[C@@H](O)n2cc(c3ccccc32)C(=O)c4cccc5ccccc54	8.3
113	O=C(c1cn(c2ccccc21)COc3cc(ccc3[C@@H]([N+])C)C)c4cccc5ccccc54	8.3
114	Fc1ccc([C@@H](O)n2cc(c3ccccc32)C(=O)c4cccc5ccccc54)c(c1)C	8.3
115	Clc1cc(Cl)ccc1[C@@H](O)n2cc(c3ccccc32)C(=O)c4cccc5ccccc54	8.3
116	O=C(c1cn(Sc2nc(cs2)C)c3ccccc31)c4cccc5ccccc54	8.3
117	Fc1ccc(F)cc1[C@H](n2cc(c3ccccc32)C(=O)c4cccc5ccccc54)C[N+]	8.3
118	O=C(c1cn([C@H]([N+])c2cc(oc2C)C)c3ccccc31)c4cccc5ccccc54	8.3
119	O=C(c1cn(c2ccccc21)Cc3nc(c(s3)C[N+])C)c4cccc5ccccc54	8.3
120	Fc1cccc(F)c1[C@@H]([N+])n2cc(c3ccccc32)C(=O)c4cccc5ccccc54	8.3
121	Fc1cc2CC[N+][C@@H](c2cc1C)Cn3cc(c4ccccc43)C(=O)c5cccc6ccccc65	8.3
122	O=C(c1cn(c2ccccc21)C[n+]3ccccc3N)c4cccc5ccccc54	8.3
123	Brc1cccc(F)c1Cn2cc(c3ccccc32)C(=O)c4cccc5ccccc54	8.3
124	O=C(c1cn(C[C@@H](CCC)C#N)c2ccccc21)c3cccc4ccccc43	8.3
125	Fc1cc(ccc1Sn2cc(c3ccccc32)C(=O)c4cccc5ccccc54)C#N	8.3
126	O=C(c1cn(CCCCC(N)=[N+])c2ccccc21)c3cccc4ccccc43	8.3
127	O=C(c1cn([C@H]([N+]C)c2cccs2)c3ccccc31)c4cccc5ccccc54	8.3
128	O=C(c1cn(c2ccccc21)Cc3cccc4cnccc43)c5cccc6ccccc65	8.3
129	Fc1cccc([C@@H](O)n2cc(c3ccccc32)C(=O)c4cccc5ccccc54)c1	8.3
130	O=C(c1cn(c2ccccc21)C#CCCCCC)c3cccc4ccccc43	8.3
131	O=C(c1cn([C@@H]([N+]C)C2CCCCC2)c3ccccc31)c4cccc5ccccc54	8.3
132	O=C(c1cn(c2ccccc21)CSCCCC[N+])c3cccc4ccccc43	8.3
133	O=C(c1cn([C@H]([N+])c2c(ccs2)C)c3ccccc13)c4cccc5ccccc54	8.3
134	Fc1c(cccc1Cn2cc(c3ccccc32)C(=O)c4cccc5ccccc54)C(F)(F)F	8.3
135	Clc1ccc(o1)Cn2cc(c3ccccc32)C(=O)c4cccc5ccccc54	8.3
136	Fc1ccc(cc1Cn2cc(c3ccccc32)C(=O)c4cccc5ccccc54)C	8.3
137	O[C@@H](CCn1cc(c2ccccc21)C(=O)c3cccc4ccccc43)C[N+]	8.3
138	Fc1ccc(c(Cn2cc(c3ccccc32)C(=O)c4cccc5ccccc54)c1)C#N	8.3
139	Fc1ccccc1[C@H](n2cc(c3ccccc32)C(=O)c4cccc5ccccc54)C[N+]	8.3
140	Clc1c(Cl)cccc1[C@@H]([N+])n2cc(c3ccccc32)C(=O)c4cccc5ccccc54	8.3
141	Brc1ccccc1[C@@H]([N+])n2cc(c3ccccc32)C(=O)c4cccc5ccccc54	8.3
142	O=C(c1cn(C[C@H]2CCC[N+]CC2)c3ccccc31)c4cccc5ccccc54	8.3
143	O=C(c1cn(c2ccccc21)Cc3cccc(C[N+])c3)c4cccc5ccccc54	8.3
144	O=C(c1cn(c2ccccc21)Cc3cc(c(o3)C[N+])C)c4cccc5ccccc54	8.3
145	O=C(c1cn(C[C@H]2CCCCC[N+]2)c3ccccc31)c4cccc5ccccc54	8.3
146	O=C(c1cn(C[N+]2CCCC[C@@H]2CC)c3ccccc31)c4cccc5ccccc54	8.3
147	Fc1ccc(c(C[N+])c1)Cn2cc(c3ccccc32)C(=O)c4cccc5ccccc54	8.2

148	Fc1ccccc1[C@@H]([N+]C)n2cc(c3ccccc32)C(=O)c4cccc5ccccc54	8.2
149	Fc1cc(F)cc(F)c1[C@@H]([N+])n2cc(c3ccccc32)C(=O)c4cccc5ccccc54	8.2
150	O=S(=O)(n1cc(c2ccccc21)C(=O)c3cccc4ccccc43)c5ccccc5C[N+]	8.2
151	FC(F)(F)C[N+](Cn1cc(c2ccccc21)C(=O)c3cccc4ccccc43)CC	8.2
152	O=C(c1cn(SCCC=C)c2ccccc21)c3cccc4ccccc43	8.2
153	O=C(c1cn(C[C@H]2CCC[C@H]([N+])C2)c3ccccc31)c4cccc5ccccc54	8.2
154	O=C(c1cn(c2ccccc21)Cc3cccc([N+]([O-])=O)c3C)c4cccc5ccccc54	8.2
155	Fc1ccc(S(=O)(=O)n2cc(c3ccccc32)C(=O)c4cccc5ccccc54)c(c1)C	8.2
156	O=C(c1cn([C@@H](C[N+])c2cscc2)c3ccccc31)c4cccc5ccccc54	8.2
157	O=C(c1cn(c2ccccc21)Cc3nc(C[N+]C)cs3)c4cccc5ccccc54	8.2
158	O=C(c1cn(Sc2ccccc2C[N+])c3ccccc31)c4cccc5ccccc54	8.2
159	Clc1ccc([C@@H]([N+])n2cc(c3ccccc32)C(=O)c4cccc5ccccc54)c(F)c1	8.2
160	Clc1cc(Cl)ccc1[C@@H]([N+])n2cc(c3ccccc32)C(=O)c4cccc5ccccc54	8.2
161	O[C@H]1CCCC[C@@H]1[N+]Cn2cc(c3ccccc32)C(=O)c4cccc5ccccc54	8.2
162	FCCCSn1cc(c2ccccc21)C(=O)c3cccc4ccccc43	8.2
163	O=C(c1cn(CCCCC[N+])c2ccccc21)c3cccc4ccccc43	8.2
164	Fc1ccc(F)cc1[C@@H]([N+])n2cc(c3ccccc32)C(=O)c4cccc5ccccc54	8.2
165	O=C(c1cn(C[C@@H]([N+]CC)CC)c2ccccc21)c3cccc4ccccc43	8.2
166	O=C(c1cn(c2ccccc21)C[n+]3cc(CC)ccc3C)c4cccc5ccccc54	8.2
167	O=C(c1cn(C[C@@H]2CCC[C@H](C2)C[N+])c3ccccc31)c4cccc5ccccc54	8.2
168	O=C(c1cn(C[C@H]2CCCC[C@@H]2C[N+])c3ccccc31)c4cccc5ccccc54	8.2
169	O=C(c1cn(NC2CCCCCC2)c3ccccc31)c4cccc5ccccc54	8.2
170	Clc1ccccc1[C@@H]([N+])n2cc(c3ccccc32)C(=O)c4cccc5ccccc54	8.2
171	O=C(c1cn(c2ccccc21)C3=CC=CN4C(SC=C34)=O)c5cccc6ccccc65	8.1
172	O=C(c1cn(CC[C@H]2CCCC[N+]2)c3ccccc31)c4cccc5ccccc54	8.1
173	Clc1cc(F)ccc1[C@@H]([N+])n2cc(c3ccccc32)C(=O)c4cccc5ccccc54	8.1
174	ClC(Cl)=C(Cl)Cn1cc(c2ccccc21)C(=O)c3cccc4ccccc43	8.1
175	O=C(c1cn(C[N+]2CC[C@H](C2)CC)c3ccccc31)c4cccc5ccccc54	8.1
176	O=C(c1cn(C[N+]2CCCC[C@@H]2C)c3ccccc31)c4cccc5ccccc54	8.1
177	O=C(c1c2ccccc2n(-[n+]3csc4ccccc43)c1)c5cccc6ccccc65	8.1
178	Fc1cc(F)ccc1[C@H](n2cc(c3ccccc32)C(=O)c4cccc5ccccc54)C[N+]	8.1
179	O=C(c1cn(CC[N+]CCC)c2ccccc21)c3cccc4ccccc43	8.1
180	O=C(c1cn(C[C@H]2C[N+](CCO2)CC)c3ccccc31)c4cccc5ccccc54	8.1
181	FC(SCn1cc(c2ccccc21)C(=O)c3cccc4ccccc43)F	8.1
182	O=C(c1cn(Cc2cnc(n2C)[N+]([O-])=O)c3ccccc31)c4cccc5ccccc54	8.1
183	O=C(c1cn(CC[N+]2CCCC2)c3ccccc31)c4cccc5ccccc54	8.1
184	Clc1ccc(C[N+]C)c(On2cc(c3ccccc32)C(=O)c4cccc5ccccc54)c1	8.1
185	O=C(c1cn(c2ccccc21)-c3cc[n+](c4ccccc43)C)c5cccc6ccccc65	8.1
186	Fc1cc(F)cc(F)c1[C@@H]([N+]C)n2cc(c3ccccc32)C(=O)c4cccc5ccccc54	8.1
187	FC1(F)C[N+](CC1)CCn2cc(c3ccccc32)C(=O)c4cccc5ccccc54	8.1
188	O=C(c1cn(C[C@H]2CNCC[N+]2C)c3ccccc31)c4cccc5ccccc54	8.1
189	Br/C=C/Cn1cc(c2ccccc21)C(=O)c3cccc4ccccc43	8.1

190	O=C(n1cc(c2ccccc21)C(=O)c3cccc4ccccc43)[C@H]5CCCC[N+]5C	8.1
191	Sc1nccn1Cn2cc(c3ccccc32)C(=O)c4cccc5ccccc54	8.1
192	Clc1ccccc1[C@H](n2cc(c3ccccc32)C(=O)c4cccc5ccccc54)[C@@H]([N+])C	8.1
193	Clc1cccc(F)c1[C@H](n2cc(c3ccccc32)C(=O)c4cccc5ccccc54)C[N+]	8.1
194	O=C(c1cn(Nc2ccncc2C)c3ccccc31)c4cccc5ccccc54	8.1
195	O=C(c1cn(C[C@H]2CCCC[N+]2C)c3ccccc31)c4cccc5ccccc54	8.1
196	O=C(c1cn(Nc2ccccc2[C@H]([N+])C)c3ccccc31)c4cccc5ccccc54	8.1
197	O=C(c1cn(C[C@H]2CCCS2)c3ccccc31)c4cccc5ccccc54	8.1
198	Fc1cc(F)cc(On2cc(c3ccccc32)C(=O)c4cccc5ccccc54)c1C	8.1
199	O=C(c1cn(CC[C@H]2CCCC[N+]2C)c3ccccc31)c4cccc5ccccc54	8.1
200	Fc1cnccc1Nn2cc(c3ccccc32)C(=O)c4cccc5ccccc54	8.1
201	O=C(c1cn(Sc2ncns2)c3ccccc31)c4cccc5ccccc54	8
202	O=C(c1cn(c2ccccc21)Cc3cc(oc3C)C[N+]C)c4cccc5ccccc54	8
203	O=C(c1cn(CC[N+](CCNC)C)c2ccccc21)c3cccc4ccccc43	8
204	O=C(c1cn([C@H]([N+])c2ccccc2C)c3ccccc13)c4cccc5ccccc54	8
205	Fc1ccc(F)cc1S(=O)(=O)n2cc(c3ccccc32)C(=O)c4cccc5ccccc54	8
206	O=C(c1cn(C[C@@H](C2CC2)C)c3ccccc31)c4cccc5ccccc54	8
207	O=C(c1cn(O/N=C(/SC)C)c2ccccc21)c3cccc4ccccc43	8
208	SCCSn1cc(c2ccccc21)C(=O)c3cccc4ccccc43	8
209	Fc1ccc(Nn2cc(c3ccccc32)C(=O)c4cccc5ccccc54)c(C[N+])c1	8
210	O=C(c1cn(C[N+](C2CCC2)C)c3ccccc31)c4cccc5ccccc54	8
211	O=C(c1cn(C[C@@H]2C[N+](CC2)C)c3ccccc31)c4cccc5ccccc54	8
212	O=C(c1cn(C[N+]2C[C@H]([C@H](C2)C)C)c3ccccc31)c4cccc5ccccc54	8
213	O=C(c1cn(CC[N+]2[C@@H](CC[C@H]2C)C)c3ccccc31)c4cccc5ccccc54	8
214	O=C(c1cn(C[N+](CCSC)C)c2ccccc21)c3cccc4ccccc43	8
215	FC(F)(F)[C@H](Cn1cc(c2ccccc21)C(=O)c3cccc4ccccc43)C	8
216	O=C(c1cn([C@H](C[N+])c2ccccn2)c3ccccc31)c4cccc5ccccc54	8
217	O=C(c1cn(c2ccccc21)Cc3ccnn3CC)c4cccc5ccccc54	8
218	O=C(c1cn(SCC(C)=C)c2ccccc12)c3cccc4ccccc43	8
219	O=C(c1cn([C@H](C[N+])c2cccs2)c3ccccc31)c4cccc5ccccc54	8
220	O=C(c1cn([C@H]([N+])c2ccccc2OC)c3ccccc31)c4cccc5ccccc54	8
221	O=C(c1cn(C[N+]2CCCCCCC2)c3ccccc31)c4cccc5ccccc54	8
222	O=C(c1cn(C[N+](CCOC)C)c2ccccc21)c3cccc4ccccc43	8
223	Clc1ccc(On2cc(c3ccccc32)C(=O)c4cccc5ccccc54)c(F)c1	8
224	O=S(=O)(C(C)C)Cn1cc(c2ccccc21)C(=O)c3cccc4ccccc43	8
225	Clc1c(F)cccc1Cn2cc(c3ccccc32)C(=O)c4cccc5ccccc54	8
226	O[C@]1(C[N+]CCC1)Cn2cc(c3ccccc32)C(=O)c4cccc5ccccc54	8
227	FC(F)(F)CSn1cc(c2ccccc21)C(=O)c3cccc4ccccc43	8
228	Clc1ccccc1[C@H](n2cc(c3ccccc32)C(=O)c4cccc5ccccc54)CC[N+]	8
229	O=C(c1cn(Sc2ccoc2C)c3ccccc31)c4cccc5ccccc54	8
230	O=C(c1cn(C[N+]2C[C@H](CC2)C)c3ccccc31)c4cccc5ccccc54	8
231	O=C(c1cn(CC[S+](C)C)c2ccccc12)c3cccc4ccccc43	8

232	O=C(c1cn([C@H]([N+])c2ccco2)c3ccccc13)c4cccc5ccccc54	8
233	O=C(c1cn([C@H]([N+])c2ccccc2OCC)c3ccccc31)c4cccc5ccccc54	8
234	FC(F)C[N+](Cn1cc(c2ccccc21)C(=O)c3cccc4ccccc43)C	8
235	O=C(c1cn(CC[C@@H](SC)C)c2ccccc21)c3cccc4ccccc43	8
236	O=C(c1cn(CC2([N+])CCCC2)c3ccccc31)c4cccc5ccccc54	8
237	O=C(c1cn([C@H]([N+])c2ccccc2C#N)c3ccccc31)c4cccc5ccccc54	8
238	O=C(c1cn(c2ccccc21)Cc3ccc(o3)C)c4cccc5ccccc54	8
239	Fc1ccccc1[C@@H]([N+])n2cc(c3ccccc32)C(=O)c4cccc5ccccc54	8
240	FC(F)(F)C[N+](Cn1cc(c2ccccc21)C(=O)c3cccc4ccccc43)C	8
241	Clc1cccc(Cl)c1[C@@H]([N+])n2cc(c3ccccc32)C(=O)c4cccc5ccccc54	8
242	O=C(c1cn(C[N+](CC)(CC)C)c2ccccc21)c3cccc4ccccc43	8
243	FC(F)(F)c1c(F)cccc1-n2cc(c3ccccc32)C(=O)c4cccc5ccccc54	8
244	O=S(=O)(N1CCC[N+]CC1)n2cc(c3ccccc32)C(=O)c4cccc5ccccc54	8
245	O=C(c1cn(Oc2ccc(C[N+])cc2)c3ccccc31)c4cccc5ccccc54	7.9
246	O=C(c1cn(C[C@H]2CCCCC[C@H]2[N+]C)c3ccccc31)c4cccc5ccccc54	7.9
247	O=C(c1cn(C[C@@H]2C[C@H]2C)c3ccccc31)c4cccc5ccccc54	7.9
248	O=C(c1cn(NC2CCCC2)c3ccccc31)c4cccc5ccccc54	7.9
249	O=C(c1cn(C[C@@H]2CCC[N+](C2)C)c3ccccc31)c4cccc5ccccc54	7.9
250	Fc1c(F)c(F)ccc1[C@@H]([N+])n2cc(c3ccccc32)C(=O)c4cccc5ccccc54	7.9
251	O=C(c1cn(SCCC)c2ccccc12)c3cccc4ccccc43	7.9
252	O=C(n1cc(c2ccccc21)C(=O)c3cccc4ccccc43)[C@H]5CCCC[N+]5CC	7.9
253	Clc1ccc(F)c(On2cc(c3ccccc32)C(=O)c4cccc5ccccc54)c1	7.9
254	Fc1cc(c2CC[N+][C@@H](c2c1)Cn3cc(c4ccccc43)C(=O)c5cccc6ccccc65)C	7.9
255	O=C(c1cn(c2ccccc21)COC3CC[N+]CC3)c4cccc5ccccc54	7.9
256	Fc1cc(O)cc2c1CC[N+][C@@H]2Cn3cc(c4ccccc43)C(=O)c5cccc6ccccc65	7.9
257	FCCSn1cc(c2ccccc21)C(=O)c3cccc4ccccc43	7.9
258	Fc1cccc(On2cc(c3ccccc32)C(=O)c4cccc5ccccc54)c1C[N+]	7.9
259	O=C(c1cn(NCC2CC2)c3ccccc13)c4cccc5ccccc54	7.9
260	Brc1ccccc1[C@H](O)n2cc(c3ccccc32)C(=O)c4cccc5ccccc54	7.9
261	Fc1ccc(On2cc(c3ccccc32)C(=O)c4cccc5ccccc54)c(c1)C#CC[N+]	7.9
262	O=C(c1c2ccccc2n(CCN(C(N)=[N+])C)c1)c3cccc4ccccc43	7.9
263	O=C(c1cn(C[C@@H](CC)C#N)c2ccccc21)c3cccc4ccccc43	7.9
264	O=C(c1cn(C[C@@H]2C[N+](CC2)CCC)c3ccccc31)c4cccc5ccccc54	7.9
265	O=C(c1cn(CC2CC=CC2)c3ccccc31)c4cccc5ccccc54	7.9
266	O=C(c1cn(NC[C@@H](CC)C)c2ccccc12)c3cccc4ccccc43	7.9
267	Fc1ccc([C@@H]([N+])n2cc(c3ccccc32)C(=O)c4cccc5ccccc54)c(c1)C	7.9
268	O=C(c1cn(C[N+]2CCCC[C@@H]2C3OCCO3)c4ccccc41)c5cccc6ccccc65	7.9
269	O=C(c1cn(O[C@@H]2CSCC2)c3ccccc31)c4cccc5ccccc54	7.9
270	O=C(c1cn(C[C@H]2CCCC[N+]2CC)c3ccccc31)c4cccc5ccccc54	7.9
271	O=C(c1cn(C[N+]2CCC[C@H]2C(C)C)c3ccccc31)c4cccc5ccccc54	7.9
272	O=C(c1cn(C[C@@H](C[N+]C)C)c2ccccc21)c3cccc4ccccc43	7.9
273	Fc1cccc(On2cc(c3ccccc32)C(=O)c4cccc5ccccc54)c1C(N)=[N+]	7.9

274	Clc1ccccc1[C@@H](n2cc(c3ccccc32)C(=O)c4cccc5ccccc54)C[N+]	7.9
275	O=C(c1cn(N2C=3C(=O)C=CC3SC=N2)c4ccccc41)c5cccc6ccccc65	7.9
276	O=C(c1cn(C[C@@H]2CCCC[N+]2)c3ccccc31)c4cccc5ccccc54	7.9
277	F[C@@H]1C[N+](CC1)Cn2cc(c3ccccc32)C(=O)c4cccc5ccccc54	7.9
278	O=S(=O)(N1CC[N+](C[C@@H]1C)C)n2cc(c3ccccc32)C(=O)c4cccc5ccccc54	7.9
279	O=C(c1cn(c2ccccc21)Cn3ccnc3[N+]([O-])=O)c4cccc5ccccc54	7.9
280	O=C(c1cn(C[N+]2CC(CC2)(C)C)c3ccccc31)c4cccc5ccccc54	7.9
281	O=C(c1cn(-n2ncoc-3csccn32)c4ccccc41)c5cccc6ccccc65	7.9
282	BrC(CSn1cc(c2ccccc21)C(=O)c3cccc4ccccc43)=C	7.9
283	O=C(c1cn(C[N+]2CCCCC2)c3ccccc31)c4cccc5ccccc54	7.8
284	O=C(c1cn(c2ccccc21)Cc3cccnc3C[N+])c4cccc5ccccc54	7.8
285	O=C(c1cn(C[C@H]2CCC[N+]2CCC)c3ccccc31)c4cccc5ccccc54	7.8
286	O=C(c1cn(Cc2c(nc(s2)CC[N+])C)c3ccccc31)c4cccc5ccccc54	7.8
287	Fc1cc(F)cc2c1CC[N+][C@@H]2Cn3cc(c4ccccc43)C(=O)c5cccc6ccccc65	7.8
288	O=C(c1cn(CC(C2CC2)C3CC3)c4ccccc41)c5cccc6ccccc65	7.8
289	O=C(c1cn(C[C@@H]([N+]CCCC)C)c2ccccc21)c3cccc4ccccc43	7.8
290	Fc1cc(F)c(F)cc1On2cc(c3ccccc32)C(=O)c4cccc5ccccc54	7.8
291	O=C(c1cn([C@@H]([N+]C)C2CCCC2)c3ccccc31)c4cccc5ccccc54	7.8
292	Fc1cc(F)cc(F)c1On2cc(c3ccccc32)C(=O)c4cccc5ccccc54	7.8
293	O=C(c1cn(C[N+]CCC)c2ccccc21)c3cccc4ccccc43	7.8
294	O=C(c1cn(CC[N+]2CCC2)c3ccccc31)c4cccc5ccccc54	7.8
295	O=S(=O)(n1cc(c2ccccc21)C(=O)c3cccc4ccccc43)c5ccccc5C	7.8
296	Fc1cc(ccc1Cn2cc(c3ccccc32)C(=O)c4cccc5ccccc54)C#CC[N+]	7.8
297	O=C(c1cn(NC2CC[N+]CC2)c3ccccc31)c4cccc5ccccc54	7.8
298	Fc1ccc(C[N+])cc1Cn2cc(c3ccccc32)C(=O)c4cccc5ccccc54	7.8
299	O=C(c1cn([C@@H]([N+])C2CCCC2)c3ccccc13)c4cccc5ccccc54	7.8
300	O=C(c1cn(c2ccccc21)Cn3cccc3C[N+])c4cccc5ccccc54	7.8
301	Fc1ccccc1S(=O)(=O)n2cc(c3ccccc32)C(=O)c4cccc5ccccc54	7.8
302	O=C(c1cn(C[N+]2CC=CCC2)c3ccccc31)c4cccc5ccccc54	7.8
303	O=C(c1cn(C[C@H]2CCC[N+]2)c3ccccc31)c4cccc5ccccc54	7.8
304	Fc1cccc(-n2cc(c3ccccc32)C(=O)c4cccc5ccccc54)c1OC6C[N+]C6	7.8
305	Fc1cc(C[N+])ccc1Cn2cc(c3ccccc32)C(=O)c4cccc5ccccc54	7.8
306	O=C(c1cn(C[N+]2CCCOCC2)c3ccccc31)c4cccc5ccccc54	7.8
307	O=C(c1cn(C[N+]2CSCC2)c3ccccc31)c4cccc5ccccc54	7.8
308	O=C(c1cn(C[C@H]2CCC[C@H]2[N+]C)c3ccccc31)c4cccc5ccccc54	7.8
309	O=C(c1cn(c2ccccc21)Cn3ccnc3C[N+])c4cccc5ccccc54	7.8
310	O=C(c1cn(CC[N+]2CC=CCC2)c3ccccc31)c4cccc5ccccc54	7.8
311	O[C@@H](n1cc(c2ccccc21)C(=O)c3cccc4ccccc43)C[N+](CC)CC	7.8
312	O=C(c1cn(C[N+]2CCCC[C@@H]2C(C)C)c3ccccc31)c4cccc5ccccc54	7.8
313	Fc1ccc(Sn2cc(c3ccccc32)C(=O)c4cccc5ccccc54)c([C@H]([N+])C)c1	7.8
314	O=C(c1cn(Oc2cc3c(OCO3)cc2C[N+])c4ccccc41)c5cccc6ccccc65	7.7
315	O=C(c1cn(c2ccccc21)C[n+]3ccccc3C)c4cccc5ccccc54	7.7

316	O[C@@H](n1cc(c2ccccc21)C(=O)c3cccc4ccccc43)[C@@H]5CCCC[N+]5	7.7
317	O=C(c1cn(c2ccccc21)Cc3ccccc3C[N+])c4cccc5ccccc54	7.7
318	F[C@@H]1C[C@H]([N+]C1)Cn2cc(c3ccccc32)C(=O)c4cccc5ccccc54	7.7
319	O=C(c1cn(CC[N+](C2CC2)C(C)C)c3ccccc31)c4cccc5ccccc54	7.7
320	O=C(c1cn(C[C@@H]2COCC[N+]2)c3ccccc31)c4cccc5ccccc54	7.7
321	O=C(c1cn(CC[N+]2C[C@@H]2C)c3ccccc31)c4cccc5ccccc54	7.7
322	O=C(c1cn(C[C@H]2CCC[C@H]2C[N+])c3ccccc31)c4cccc5ccccc54	7.7
323	O=C(c1cn(C[C@@H]2c3ccccc3CC[N+]2)c4ccccc41)c5cccc6ccccc65	7.7
324	O=C(c1cn(C[N+]2CCCC2)c3ccccc31)c4cccc5ccccc54	7.7
325	O=C(c1cn(C[C@H]([N+]CC)CCC)c2ccccc21)c3cccc4ccccc43	7.7
326	O=C(c1cn(C[C@@H]2CO[C@@H](C[N+]2)C)c3ccccc31)c4cccc5ccccc54	7.7
327	O=C(c1cn(Sc2ccc(cc2C)C#N)c3ccccc31)c4cccc5ccccc54	7.7
328	FC(F)C(F)(F)Cn1cc(c2ccccc21)C(=O)c3cccc4ccccc43	7.7
329	O=C(c1cn(c2ccccc21)/C=C(\C3CC3)C)c4cccc5ccccc54	7.7
330	O=C(c1cn(C[C@H]2CCC[N+]2CC=C)c3ccccc31)c4cccc5ccccc54	7.7
331	O=C(c1cn(Cc2c(nc(s2)C[N+])C)c3ccccc31)c4cccc5ccccc54	7.7
332	O=C(c1cn(CC[N+](C(C)C)C(C)C)c2ccccc21)c3cccc4ccccc43	7.7
333	O=C(c1cn(CC[N+]C)c2ccccc21)c3cccc4ccccc43	7.7
334	O=C(c1cn(CC[N+](CC)C)c2ccccc21)c3cccc4ccccc43	7.7
335	O=C(c1cn(CC[N+]2C[C@@H](CC2)C)c3ccccc31)c4cccc5ccccc54	7.7
336	O=C(c1cn(C[C@H]([N+](C)C)c2ccco2)c3ccccc31)c4cccc5ccccc54	7.7
337	O=C(c1cn(c2ccccc21)Cc3ccoc3C[N+]CC)c4cccc5ccccc54	7.7
338	O=C(c1c2ccccc2n([C@@H]([N+])[C@H]3CCCCO3)c1)c4cccc5ccccc54	7.7
339	O=C(c1cn(c2ccccc21)Cc3cc(C[N+])co3)c4cccc5ccccc54	7.7
340	O=C(c1cn(OC[C@H]2CC[N+]2)c3ccccc13)c4cccc5ccccc54	7.7
341	O=C(c1cn(C[C@H]2CCC[N+]2CC)c3ccccc31)c4cccc5ccccc54	7.7
342	Fc1ccc(On2cc(c3ccccc32)C(=O)c4cccc5ccccc54)c(C[N+])c1	7.6
343	FCCOn1cc(c2ccccc21)C(=O)c3cccc4ccccc43	7.6
344	Fc1cc(O)ccc1[C@@H]([N+])n2cc(c3ccccc32)C(=O)c4cccc5ccccc54	7.6
345	O=C(c1cn(C[C@@H]2C[N+](CC2)CC)c3ccccc31)c4cccc5ccccc54	7.6
346	O=C(c1cn(C[C@@H]([N+](CC)CC)C)c2ccccc21)c3cccc4ccccc43	7.6
347	O=C(c1cn(Cc2c[nH+]c(N(C)C)n2C)c3ccccc31)c4cccc5ccccc54	7.6
348	O=C(c1cn(Oc2ccccc2C[N+])c3ccccc31)c4cccc5ccccc54	7.6
349	ClC(CSn1cc(c2ccccc21)C(=O)c3cccc4ccccc43)=C	7.6
350	O=C(c1cn(Sc2c(nccn2)C[N+])c3ccccc31)c4cccc5ccccc54	7.6
351	O=C(c1cn(C[C@H]([N+]2CCCC2)CC)c3ccccc31)c4cccc5ccccc54	7.6
352	FC(F)(F)/C(=C\n1cc(c2ccccc21)C(=O)c3cccc4ccccc43)C	7.6
353	O=C(c1cn(C[C@H]2C[N+](CCN2)C)c3ccccc31)c4cccc5ccccc54	7.6
354	O=C(c1cn(c2ccccc21)C[N+]CC=C)c3cccc4ccccc43	7.6
355	O=C(c1cn(C[N+]2CCC[C@@H](C2)C)c3ccccc31)c4cccc5ccccc54	7.6
356	O=C(c1cn(C[C@@H]([N+]CCC)C)c2ccccc21)c3cccc4ccccc43	7.6
357	O=C(c1cn(c2ccccc21)Cc3ccoc3C[N+])c4cccc5ccccc54	7.6

358	O=C(c1cn(C[C@@H]2CCC[C@H]2[N+])c3ccccc31)c4cccc5ccccc54	7.6
359	O=C(c1cn([C@H]([N+])c2ccoc2C)c3ccccc13)c4cccc5ccccc54	7.6
360	BrC(CCn1cc(c2ccccc21)C(=O)c3cccc4ccccc43)=C	7.6
361	O=C(c1cn(C[C@H]([N+](C)C)C2CC2)c3ccccc13)c4cccc5ccccc54	7.6
362	Clc1ccc2c(ccc[n+]2-n3cc(c4ccccc43)C(=O)c5cccc6ccccc65)c1	7.6
363	O=C(c1cn(C[C@@H]([N+])[C@H](CC)C)c2ccccc21)c3cccc4ccccc43	7.5
364	FC(Sn1cc(c2ccccc21)C(=O)c3cccc4ccccc43)(F)C(F)F	7.5
365	O=C(c1cn(Oc2ccccc2[C@@H]([N+])C)c3ccccc31)c4cccc5ccccc54	7.5
366	O=C(c1cn(CC[N+]CC)c2ccccc21)c3cccc4ccccc43	7.5
367	O=C(c1cn(N[C@@H]2CSCC2)c3ccccc31)c4cccc5ccccc54	7.5
368	O=C(c1cn(CC[N+]([C@H](CC)C)C)c2ccccc21)c3cccc4ccccc43	7.5
369	O=C(c1cn(C[N+]2CCC[C@H]2C)c3ccccc31)c4cccc5ccccc54	7.5
370	FC1(F)CC(C1)Cn2cc(c3ccccc32)C(=O)c4cccc5ccccc54	7.5
371	Fc1cccc(-n2cc(c3ccccc32)C(=O)c4cccc5ccccc54)c1CC[N+]	7.5
372	O=C(c1cn(CC[N+]CC=C)c2ccccc21)c3cccc4ccccc43	7.5
373	O=C(c1cn(CC[N+](C2CCCCC2)C)c3ccccc31)c4cccc5ccccc54	7.5
374	O=C(c1cn(C[N+](C2CC2)CC)c3ccccc31)c4cccc5ccccc54	7.5
375	SC[C@@H]([N+])CCn1cc(c2ccccc21)C(=O)c3cccc4ccccc43	7.5
376	Fc1cc(OC)ccc1[C@@H]([N+])n2cc(c3ccccc32)C(=O)c4cccc5ccccc54	7.5
377	O=C(c1cn(c2ccccc21)-c3csc4cscc43)c5cccc6ccccc65	7.5
378	O=C(c1cn(C[C@@H]2C=CCC2)c3ccccc31)c4cccc5ccccc54	7.5
379	O=C(c1cn(SSCC[N+])c2ccccc21)c3cccc4ccccc43	7.5
380	Clc1ccccc1[C@H]([N+])Cn2cc(c3ccccc32)C(=O)c4cccc5ccccc54	7.5
381	O=C(c1cn(C[C@@H]([N+]C)C(C)C)c2ccccc21)c3cccc4ccccc43	7.5
382	O=C(c1cn(C[N+]2CCSC[C@@H]2C)c3ccccc31)c4cccc5ccccc54	7.4
383	O=S(=O)(n1cc(c2ccccc21)C(=O)c3cccc4ccccc43)c5ccsc5C[N+]	7.4
384	O=C(c1cn(C[C@H]([N+](C)C)CC)c2ccccc21)c3cccc4ccccc43	7.4
385	O=C(c1cn(C[C@H](CCC)C)c2ccccc21)c3cccc4ccccc43	7.4
386	O=C(c1cn(c2ccccc21)CO[C@@H](C[N+])C)c3cccc4ccccc43	7.4
387	Fc1c(OC)ccc(F)c1-n2cc(c3ccccc32)C(=O)c4cccc5ccccc54	7.4
388	O=C(c1cn(CC2([N+]C)CCCC2)c3ccccc31)c4cccc5ccccc54	7.4
389	O=C(c1cn(C[C@H]([N+])CC2CC2)c3ccccc31)c4cccc5ccccc54	7.4
390	O=C(c1cn([C@H](C2CCC2)C[N+])c3ccccc31)c4cccc5ccccc54	7.4
391	O[C@H](n1cc(c2ccccc21)C(=O)c3cccc4ccccc43)[C@H]5CCC[N+]5	7.4
392	O=C(c1cn([C@@H]([N+](CC)CC)C)c2ccccc12)c3cccc4ccccc43	7.4
393	O=C(c1cn(C[C@@H]([N+]CC)COC)c2ccccc21)c3cccc4ccccc43	7.4
394	O=C(c1cn(CC([N+](CC)CC)(C)C)c2ccccc21)c3cccc4ccccc43	7.4
395	O=C(c1cn(CC2(CCC2)C)c3ccccc31)c4cccc5ccccc54	7.3
396	O=C(c1cn(c2ccccc21)C[S@](=O)C)c3cccc4ccccc43	7.3
397	O=C(c1cn(C[C@H]([N+]C)C2CC2)c3ccccc31)c4cccc5ccccc54	7.3
398	O=C(c1c2ccccc2n([C@@H]([N+])CC(C)C)c1)c3cccc4ccccc43	7.3
399	Fc1c(OC)c(F)ccc1-n2cc(c3ccccc32)C(=O)c4cccc5ccccc54	7.3

400	Fc1c(-n2cc(c3ccccc32)C(=O)c4cccc5ccccc54)ccc(F)c1C[N+]	7.3
401	Fc1c(-n2cc(c3ccccc32)C(=O)c4cccc5ccccc54)ccc(F)c1C[N+]C	7.3
402	O=C(c1cn(C[C@H]([N+])C(C)C)c2ccccc21)c3cccc4ccccc43	7.3
403	O=C(c1cn(C[N+](C2CCCC2)C)c3ccccc31)c4cccc5ccccc54	7.3
404	O=C(c1cn(O[C@@H](C2CC2)C#C)c3ccccc13)c4cccc5ccccc54	7.3
405	ClC(CCn1cc(c2ccccc21)C(=O)c3cccc4ccccc43)=C	7.3
406	O=C(c1cn(CC[N+]2CC2)c3ccccc31)c4cccc5ccccc54	7.3
407	O=C(c1cn(c2ccccc21)CSC)c3cccc4ccccc43	7.3
408	Fc1c(On2cc(c3ccccc32)C(=O)c4cccc5ccccc54)cccn1	7.2
409	FC(F)(F)C(F)(F)Cn1cc(c2ccccc21)C(=O)c3cccc4ccccc43	7.2
410	O=C(c1cn(C[C@H]([N+]C)COC)c2ccccc21)c3cccc4ccccc43	7.2
411	FC(F)CSn1cc(c2ccccc21)C(=O)c3cccc4ccccc43	7.2
412	O=C(c1cn(CC[N+](CCCC)C)c2ccccc21)c3cccc4ccccc43	7.2
413	Fc1cc(ccc1On2cc(c3ccccc32)C(=O)c4cccc5ccccc54)C#N	7.2
414	O=C(c1cn(C[C@H]2CS2)c3ccccc31)c4cccc5ccccc54	7.2
415	O=C(c1cn(SCC#C)c2ccccc21)c3cccc4ccccc43	7.2
416	O=C(c1cn([C@@H]2CNCCC[N+]2CC)c3ccccc13)c4cccc5ccccc54	7.2
417	O=C(c1cn(C[C@@H]([N+]C)C#N)c2ccccc21)c3cccc4ccccc43	7.2
418	O=C(c1cn(c2ccccc21)C[N+](CC=C)C)c3cccc4ccccc43	7.2
419	OC[C@H]([N+]C)Cn1cc(c2ccccc21)C(=O)c3cccc4ccccc43	7.2
420	O=C(c1cn(c2ccccc21)COCC)c3cccc4ccccc43	7.2
421	O=C(c1cn([C@@H]([N+]2CCCC2)C)c3ccccc13)c4cccc5ccccc54	7.1
422	O=C(c1cn(CC(CC)CC)c2ccccc21)c3cccc4ccccc43	7.1
423	O=C(c1cn(c2ccccc21)CC(C)C)c3cccc4ccccc43	7.1
424	O=C(c1cn(CC[N+]2CCCCC2)c3ccccc31)c4cccc5ccccc54	7.1
425	SCC1(CC1)Cn2cc(c3ccccc32)C(=O)c4cccc5ccccc54	7.1
426	O=C(c1cn(C[C@@H]([N+]C)C)c2ccccc21)c3cccc4ccccc43	7.1
427	O=C(c1cn(CC[N+](CCC)C)c2ccccc21)c3cccc4ccccc43	7.1
428	O=C(c1cn(C[C@@H]2C[N+]CC2)c3ccccc31)c4cccc5ccccc54	7.1
429	O=C(c1cn(CC[N+]2CCC[C@@H]2C)c3ccccc31)c4cccc5ccccc54	7.1
430	S=C1N=NCN1Cn2cc(c3ccccc32)C(=O)c4cccc5ccccc54	7.1
431	O=C(c1cn(Cc2cc(oc2C)C[N+])c3ccccc31)c4cccc5ccccc54	7.1
432	O=C(c1cn([C@@H]([N+]C)C2CCC2)c3ccccc31)c4cccc5ccccc54	7.1
433	O=C(c1cn(CC[N+](C2CC2)C)c3ccccc31)c4cccc5ccccc54	7.1
434	O=C(c1cn(CC[C@@H](C[N+]C)C)c2ccccc21)c3cccc4ccccc43	7.1
435	O=C(c1cn(CC[N+](C)C)c2ccccc12)c3cccc4ccccc43	7.1
436	O=C(c1cn(CCC(C)=C)c2ccccc21)c3cccc4ccccc43	7.1
437	O=C(c1cn(CC2(C3CC3)CC2)c4ccccc41)c5cccc6ccccc65	7.1
438	O=C(c1cn([C@@H]([N+])[C@@H](CC)C)c2ccccc12)c3cccc4ccccc43	7
439	O=C(c1cn(C[C@@H]([N+](C)C)CN)c2ccccc21)c3cccc4ccccc43	7
440	O=C(c1cn(C[C@@H]([N+](C)C)C)c2ccccc21)c3cccc4ccccc43	7
441	O=C(c1cn(C[C@@H]([N+]CC)C)c2ccccc21)c3cccc4ccccc43	7

442	O=C(c1cn(C[N+](C2CC2)C)c3ccccc31)c4cccc5ccccc54	7
443	O=C(c1cn(CC[N+]2CCC[C@H]2CC)c3ccccc31)c4cccc5ccccc54	7
444	O=C(c1cn(C[N+]2CCC2)c3ccccc31)c4cccc5ccccc54	7
445	O=C(c1cn(C[C@H](C(C)C)C)c2ccccc21)c3cccc4ccccc43	7
446	O=C(c1cn(C[C@@H]([N+])c2cccs2)c3ccccc31)c4cccc5ccccc54	6.9
447	O=C(c1cn(CC2(CCC2)C[N+])c3ccccc31)c4cccc5ccccc54	6.9
448	O=C(c1cn(C2=CCC[N+](C2)CC)c3ccccc13)c4cccc5ccccc54	6.9
449	O=C(c1cn(OC2CSC2)c3ccccc31)c4cccc5ccccc54	6.9
450	O=C(c1cn(c2ccccc21)C[N+](CC#C)CC#C)c3cccc4ccccc43	6.9
451	BrC(Br)=Cn1cc(c2ccccc21)C(=O)c3cccc4ccccc43	6.9
452	O=C(c1cn(CC2(CC2)CC)c3ccccc31)c4cccc5ccccc54	6.9
453	S=C(On1cc(c2ccccc21)C(=O)c3cccc4ccccc43)N(C)C	6.9
454	Clc1c(F)cc(F)cc1-n2cc(c3ccccc32)C(=O)c4cccc5ccccc54	6.9
455	O=C(c1cn(c2ccccc21)C[S+](C)C)c3cccc4ccccc43	6.9
456	O=C(c1cn(C[C@H]([C@@H]([N+])C)C)c2ccccc21)c3cccc4ccccc43	6.8
457	O=C(c1cn([C@@H]([N+])C2CCC2)c3ccccc13)c4cccc5ccccc54	6.8
458	FC(Sn1cc(c2ccccc21)C(=O)c3cccc4ccccc43)F	6.8
459	O=C(c1cn(C[C@H]2CC[N+]2C)c3ccccc31)c4cccc5ccccc54	6.8
460	FC(F)CCn1cc(c2ccccc21)C(=O)c3cccc4ccccc43	6.8
461	ClC(Cl)=Cn1cc(c2ccccc21)C(=O)c3cccc4ccccc43	6.8
462	O=C(c1cn(c2ccccc21)CSCC[N+])c3cccc4ccccc43	6.8
463	O=C(c1cn(c2ccccc21)CN(OC)C)c3cccc4ccccc43	6.8
464	FC(F)Cn1cc(c2ccccc21)C(=O)c3cccc4ccccc43	6.8
465	O=C(c1cn([C@@H]([N+])C(CC)CC)c2ccccc12)c3cccc4ccccc43	6.8
466	BrC(Cn1cc(c2ccccc21)C(=O)c3cccc4ccccc43)=C	6.7
467	O=C(c1cn(C[C@H]([N+](CC#C)C)C)c2ccccc21)c3cccc4ccccc43	6.7
468	FC(Cn1cc(c2ccccc21)C(=O)c3cccc4ccccc43)=C	6.7
469	O=C(c1cn(CC[N+](CC(C)C)C)c2ccccc21)c3cccc4ccccc43	6.7
470	SC(=N)CCn1cc(c2ccccc21)C(=O)c3cccc4ccccc43	6.7
471	O=C(c1cn(CC2C[N+]C2)c3ccccc31)c4cccc5ccccc54	6.7
472	O=C(c1cn(S[C@H](C[N+]C)C)c2ccccc21)c3cccc4ccccc43	6.7
473	Brc1c(F)cccc1-n2cc(c3ccccc32)C(=O)c4cccc5ccccc54	6.7
474	ClC(Cn1cc(c2ccccc21)C(=O)c3cccc4ccccc43)=C	6.7
475	O=C(c1cn(C[C@@H](CC)C)c2ccccc21)c3cccc4ccccc43	6.7
476	O=C(c1cn(C[C@H](OC)C)c2ccccc21)c3cccc4ccccc43	6.7
477	O=C(c1cn([C@@H]([N+])[C@H]2C[C@H]2C)c3ccccc13)c4cccc5ccccc54	6.7
478	O=C(c1cn(C[N+]2CC2)c3ccccc31)c4cccc5ccccc54	6.7
479	O=C(c1cn(CC[N+](CC2CC2)C)c3ccccc31)c4cccc5ccccc54	6.6
480	Fc1c(F)cc(F)cc1-n2cc(c3ccccc32)C(=O)c4cccc5ccccc54	6.6
481	O=C(c1cn(c2ccccc21)CCC#C)c3cccc4ccccc43	6.5
482	O=C(c1cn(c2ccccc21)C[N+](C)C)c3cccc4ccccc43	6.4
483	O[C@H](n1cc(c2ccccc21)C(=O)c3cccc4ccccc43)[C@@H]([N+]C)CC	6.4

484	O=C(c1c2ccccc2n(C3=CCC[N+](C3)C)c1)c4cccc5ccccc54	6.4
485	O=C(c1cn(c2ccccc21)CSCC=C)c3cccc4ccccc43	6.4
486	O=C(c1cn(C[C@H](C(N)=[N+])C)c2ccccc21)c3cccc4ccccc43	6.4
487	O=C(c1cn(C[N+](CC)CC)c2ccccc21)c3cccc4ccccc43	6.3
488	O=C(c1cn([C@@H]([N+]C)C2CC2)c3ccccc31)c4cccc5ccccc54	6.3
489	O=C(c1cn(OC[N+](C)C)c2ccccc12)c3cccc4ccccc43	6.3
490	O=C(c1cn(C[N+]C(C)C)c2ccccc21)c3cccc4ccccc43	6.3
491	FC(F)(F)CCn1cc(c2ccccc21)C(=O)c3cccc4ccccc43	6.3
492	Fc1cc(F)cc(-n2cc(c3ccccc32)C(=O)c4cccc5ccccc54)c1C	6.3
493	O=C(c1cn(C[N+](CC)C)c2ccccc21)c3cccc4ccccc43	6.2
494	O=C(c1cn([C@@H]([N+])C(C)C)c2ccccc21)c3cccc4ccccc43	6.2
495	O=C(c1cn(c2ccccc21)C[N+](CC#C)C)c3cccc4ccccc43	6.2
496	O=C(c1cn(C[C@@H](C[N+])C)c2ccccc21)c3cccc4ccccc43	6.1
497	O=C(c1cn(C[C@@H]([N+]C)CC)c2ccccc21)c3cccc4ccccc43	6.1
498	O=C(c1cn(CCCC[N+])c2ccccc21)c3cccc4ccccc43	6
499	O=C(c1cn([C@@H]2[C@H](CCC[N+]2)C)c3ccccc31)c4cccc5ccccc54	6
500	O=C(c1cn(-[n+]2cscc2C)c3ccccc13)c4cccc5ccccc54	5.9

Table S6. List, SMILE and predicted pK_i values for Series 2 in CB_1 receptor.

N°	SMILES	Pred pK_i
1	O=C(c1cn(n2n1scns2)C[C@H]3CCCC[N+]3C)c4cccc5ccccc54	8.8
2	O=C(c1cc(C[C@H]2CCCC[N+]2C)ccc1S([O-])(=O)=O)c3cccc4ccccc43	8.7
3	O=C(c1cc(n2c1cns2)C[C@H]3CCCC[N+]3C)c4cccc5ccccc54	8.6
4	O=C(c1cc(n(c1C)C)C[C@H]2CCCC[N+]2C)c3cccc4ccccc43	8.6
5	Fc1ccc(C[C@H]2CCCC[N+]2C)cc1C(=O)c3cccc4ccccc43	8.5
6	O=C(c1c2ccc(O)cc2n(n1)C[C@H]3CCCC[N+]3C)c4cccc5ccccc54	8.5
7	O=C(c1c(sc(C[C@H]2CCCC[N+]2C)c1)C(=O)N)c3cccc4ccccc43	8.5
8	Fc1ccc(OC[C@H]2CCCC[N+]2C)c(CC(=O)c3cccc4ccccc43)c1	8.5
9	O=C(c1c2ccncn2c(C[C@H]3CCCC[N+]3C)c1)c4cccc5ccccc54	8.5
10	O=C(c1c2cc[nH]c2c(o1)C[C@H]3CCCC[N+]3C)c4cccc5ccccc54	8.5
11	O=C(c1c(c(c(s1)C[C@H]2CCCC[N+]2C)C)C)c3cccc4ccccc43	8.4
12	O=C(c1c(sc(C[C@H]2CCCC[N+]2C)c1)SC)c3cccc4ccccc43	8.4
13	O=C(c1c2c(n(n1)C[C@H]3CCCC[N+]3C)cns2)c4cccc5ccccc54	8.4
14	O=C(c1cn(C[C@H]2CCCC[N+]2C)c3C=CSC(=O)c31)c4cccc5ccccc54	8.4
15	O=C(c1cn(C[C@H]2CCCC[N+]2C)c3c1cc[nH]3)c4cccc5ccccc54	8.4
16	Fc1c(c(cc(C[C@H]2CCCC[N+]2C)c1)C(=O)c3cccc4ccccc43)C=O	8.4
17	O=C(c1c2ccccc2n(n1)C[C@H]3CCCC[N+]3C)c4cccc5ccccc54	8.4
18	Clc1ccc(c(OC[C@H]2CCCC[N+]2C)c1)CC(=O)c3cccc4ccccc43	8.4
19	O=C(c1cn(n2n1sncs2)C[C@H]3CCCC[N+]3C)c4cccc5ccccc54	8.4
20	O=C(c1c(sc(C[C@H]2CCCC[N+]2C)c1)OC)c3cccc4ccccc43	8.4
21	O=C(c1cn(C[C@H]2CCCC[N+]2C)c3ccncc31)c4cccc5ccccc54	8.4

22	O=C(c1cn(C[C@H]2CCCC[N+]2C)cc3-n1scns3)c4cccc5ccccc54	8.4
23	Fc1cc(C[C@H]2CCCC[N+]2C)cc(c1C)C(=O)c3cccc4ccccc43	8.4
24	Clc1c(nn(C[C@H]2CCCC[N+]2C)c1C)C(=O)c3cccc4ccccc43	8.4
25	O=C(c1c2cscc2n(n1)C[C@H]3CCCC[N+]3C)c4cccc5ccccc54	8.4
26	O=C(c1cn(C[C@H]2CCCC[N+]2C)c3cscc31)c4cccc5ccccc54	8.3
27	O=C(c1cn(C[C@H]2CCCC[N+]2C)c3c1SC(=O)N3)c4cccc5ccccc54	8.3
28	O=C(c1c2-n(sccs2)cc([nH]1)C[C@H]3CCCC[N+]3C)c4cccc5ccccc54	8.3
29	O=C(c1c2c(cc(cc2n(C[C@H]3CCCC[N+]3C)c1)C)C)c4cccc5ccccc54	8.3
30	O=C(C=1CCCN(C[C@H]2CCCC[N+]2C)C1)c3cccc4ccccc43	8.3
31	O=C(c1c(c(c([nH]1)C[C@H]2CCCC[N+]2C)C)CC)c3cccc4ccccc43	8.3
32	S=C1N(N=C(N1C)C[C@H]2CCCC[N+]2C)C(=O)c3cccc4ccccc43	8.3
33	Clc1c(cc(s1)C[C@H]2CCCC[N+]2C)C(=O)c3cccc4ccccc43	8.3
34	O=C(n1cc(n2[nH]ncsn12)C[C@H]3CCCC[N+]3C)c4cccc5ccccc54	8.3
35	O=C(c1c2C(SC=Cc2c([nH]1)C[C@H]3CCCC[N+]3C)=O)c4cccc5ccccc54	8.3
36	O=C(c1c2-n(scco2)cc([nH]1)C[C@H]3CCCC[N+]3C)c4cccc5ccccc54	8.3
37	Brc1c(cc(s1)C[C@H]2CCCC[N+]2C)C(=O)c3cccc4ccccc43	8.3
38	O=C(c1c2ccncc2c(o1)C[C@H]3CCCC[N+]3C)c4cccc5ccccc54	8.3
39	O=C(c1c2c(ocn2)cc(C[C@H]3CCCC[N+]3C)c1)c4cccc5ccccc54	8.3
40	O=C(c1cc(n2cc[nH]c12)C[C@H]3CCCC[N+]3C)c4cccc5ccccc54	8.3
41	Fc1c(F)cc(C[C@H]2CCCC[N+]2C)cc1C(=O)c3cccc4ccccc43	8.3
42	O=C(c1c2CCCCc2n(n1)C[C@H]3CCCC[N+]3C)c4cccc5ccccc54	8.3
43	Clc1ccc(C[C@H]2CCCC[N+]2C)cc1C(=O)c3cccc4ccccc43	8.3
44	O=S1(=O)N(c2ccccc2N1C[C@H]3CCCC[N+]3C)C(=O)c4cccc5ccccc54	8.3
45	O=C(c1cn(CC2CC2)c(C[C@H]3CCCC[N+]3C)c1)c4cccc5ccccc54	8.3
46	O=C(c1c2ccncc2cc(C[C@H]3CCCC[N+]3C)c1)c4cccc5ccccc54	8.3
47	Brc1cc([nH]c1C(=O)c2cccc3ccccc32)C[C@H]4CCCC[N+]4C	8.3
48	FC(F)(F)c1cc(cc(C[C@H]2CCCC[N+]2C)c1)C(=O)c3cccc4ccccc43	8.3
49	O=C(c1cn(C[C@H]2CCCC[N+]2C)cc3-n1sccs3)c4cccc5ccccc54	8.3
50	O=C(c1c(c(n(C[C@H]2CCCC[N+]2C)c1)N)C#N)c3cccc4ccccc43	8.3
51	O=C(C1=CC=CN(C[C@H]2CCCC[N+]2C)C1)c3cccc4ccccc43	8.3
52	O=S(=O)(N)c1cc(cc(C[C@H]2CCCC[N+]2C)c1)C(=O)c3cccc4ccccc43	8.3
53	O=C(c1cc(C[C@H]2CCCC[N+]2C)c3n1cns3)c4cccc5ccccc54	8.3
54	O=C(c1cn(C[C@H]2CCCC[N+]2C)c3c1SC(S3)=O)c4cccc5ccccc54	8.3
55	O=C(c1cc(C[C@H]2CCCC[N+]2C)cc(N(C)C)c1)c3cccc4ccccc43	8.3
56	O=C(c1cc(n2ccccc12)C[C@H]3CCCC[N+]3C)c4cccc5ccccc54	8.3
57	Fc1c(cc(C[C@H]2CCCC[N+]2C)cc1C(F)(F)F)C(=O)c3cccc4ccccc43	8.3
58	Fc1ccc2c(nn(C[C@H]3CCCC[N+]3C)c2c1)C(=O)c4cccc5ccccc54	8.3
59	O=C(c1cc(C[C@H]2CCCC[N+]2C)c(s1)N)c3cccc4ccccc43	8.3
60	O=C(c1cc(n(c1C)CC)C[C@H]2CCCC[N+]2C)c3cccc4ccccc43	8.3
61	O=C(c1cc(n2c1ccs2)C[C@H]3CCCC[N+]3C)c4cccc5ccccc54	8.3
62	Fc1c(SC)c(cc(C[C@H]2CCCC[N+]2C)c1)C(=O)c3cccc4ccccc43	8.3
63	O=C(c1c2-n(occo2)cc([nH]1)C[C@H]3CCCC[N+]3C)c4cccc5ccccc54	8.3

64	O=C(c1cc(C[C@H]2CCCC[N+]2C)cn3cccc13)c4cccc5ccccc54	8.2
65	Fc1c(cc(C[C@H]2CCCC[N+]2C)cn1)C(=O)c3cccc4ccccc43	8.2
66	O=C(c1c2cnccc2n(n1)C[C@H]3CCCC[N+]3C)c4cccc5ccccc54	8.2
67	O=C(c1c2cc([N+]([O-])=O)ccc2n(n1)C[C@H]3CCCC[N+]3C)c4cccc5ccccc54	8.2
68	O=C(c1cc(S([O-])(=O)=O)cc(C[C@H]2CCCC[N+]2C)c1)c3cccc4ccccc43	8.2
69	O=C(c1c(c(n(C[C@H]2CCCC[N+]2C)c1C)C)C)c3cccc4ccccc43	8.2
70	O=C(c1c2ccccc2c(o1)C[C@H]3CCCC[N+]3C)c4cccc5ccccc54	8.2
71	Fc1cc(O)c(C[C@H]2CCCC[N+]2C)cc1C(=O)c3cccc4ccccc43	8.2
72	O=C(c1cn(C[C@H]2CCCC[N+]2C)c3ccc(cc31)C#N)c4cccc5ccccc54	8.2
73	O=C(c1c2CC(CCc2c(s1)C[C@H]3CCCC[N+]3C)(C)C)c4cccc5ccccc54	8.2
74	O=C(c1c2ccncc2n(n1)C[C@H]3CCCC[N+]3C)c4cccc5ccccc54	8.2
75	O=C(c1c-2[nH]ccsn2c(C[C@H]3CCCC[N+]3C)c[nH]1)c4cccc5ccccc54	8.2
76	O=C(c1c2CCCc2n(n1)C[C@H]3CCCC[N+]3C)c4cccc5ccccc54	8.2
77	O=C(c1c2ccccn2c(C[C@H]3CCCC[N+]3C)c1N)c4cccc5ccccc54	8.2
78	Clc1c(cc(C[C@H]2CCCC[N+]2C)cc1C(F)(F)F)C(=O)c3cccc4ccccc43	8.2
79	O=C(c1c2cc[nH]c2c([nH]1)C[C@H]3CCCC[N+]3C)c4cccc5ccccc54	8.2
80	O=C(c1cc(C[C@H]2CCCC[N+]2C)c3cccc(n13)C)c4cccc5ccccc54	8.2
81	O=C(c1cc(n2C=CSC(=O)c12)C[C@H]3CCCC[N+]3C)c4cccc5ccccc54	8.2
82	Brc1cc(cc(C[C@H]2CCCC[N+]2C)c1)C(=O)c3cccc4ccccc43	8.2
83	O=C(c1c2cocc2cc(C[C@H]3CCCC[N+]3C)c1)c4cccc5ccccc54	8.2
84	Brc1c(F)c(cc(C[C@H]2CCCC[N+]2C)c1)C(=O)c3cccc4ccccc43	8.2
85	O=C(c1c2cocc2n(n1)C[C@H]3CCCC[N+]3C)c4cccc5ccccc54	8.2
86	O=C(c1c2cnsc2c(o1)C[C@H]3CCCC[N+]3C)c4cccc5ccccc54	8.2
87	Fc1cccc2c1c(nn2C[C@H]3CCCC[N+]3C)C(=O)c4cccc5ccccc54	8.2
88	O=C(C=1CC=CN(C[C@H]2CCCC[N+]2C)C1)c3cccc4ccccc43	8.2
89	O=C(c1cn(C[C@H]2CCCC[N+]2C)c3cnccc31)c4cccc5ccccc54	8.2
90	O=C(c1c(cc[n+](C[C@H]2CCCC[N+]2C)c1)C)c3cccc4ccccc43	8.2
91	O=C(c1cn(n2cccc12)C[C@H]3CCCC[N+]3C)c4cccc5ccccc54	8.2
92	O=C(c1cn(C[C@H]2CCCC[N+]2C)c3C=COC(=O)c31)c4cccc5ccccc54	8.2
93	O=C(c1cn(c(C[C@H]2CCCC[N+]2C)c1)C)c3cccc4ccccc43	8.2
94	Brc1c(cc(n1C)C[C@H]2CCCC[N+]2C)C(=O)c3cccc4ccccc43	8.2
95	Fc1c(OCC)c(cc(C[C@H]2CCCC[N+]2C)c1)C(=O)c3cccc4ccccc43	8.2
96	O=C(c1c2CCCCc2c([nH]1)C[C@H]3CCCC[N+]3C)c4cccc5ccccc54	8.2
97	Brc1c(OC)c(cc(C[C@H]2CCCC[N+]2C)c1)C(=O)c3cccc4ccccc43	8.2
98	O=C(c1c2c[nH]cc2n(n1)C[C@H]3CCCC[N+]3C)c4cccc5ccccc54	8.2
99	O=C(c1c2c(n[nH]n2)cc(C[C@H]3CCCC[N+]3C)c1)c4cccc5ccccc54	8.2
100	O=C(c1c2c(cc(C[C@H]3CCCC[N+]3C)c1)cncn2)c4cccc5ccccc54	8.2
101	Clc1c(Cl)c(nn1C[C@H]2CCCC[N+]2C)C(=O)c3cccc4ccccc43	8.2
102	Fc1cccc2c1c(cn2C[C@H]3CCCC[N+]3C)C(=O)c4cccc5ccccc54	8.2
103	Fc1c(F)c(O)c(C[C@H]2CCCC[N+]2C)cc1C(=O)c3cccc4ccccc43	8.2
104	O=C(c1c2C(=O)NC=Cc2c([nH]1)C[C@H]3CCCC[N+]3C)c4cccc5ccccc54	8.2
105	O=C(c1c2c(cc(C[C@H]3CCCC[N+]3C)c1)cco2)c4cccc5ccccc54	8.2

106	O=C(c1c2cc(ccc2n(n1)C[C@H]3CCCC[N+]3C)C#N)c4cccc5ccccc54	8.2
107	O=C(c1cn(C[C@H]2CCCC[N+]2C)c3cccc(c31)C(OC)=O)c4cccc5ccccc54	8.2
108	O=C(c1c2c(nc(C[C@H]3CCCC[N+]3C)c1)cncn2)c4cccc5ccccc54	8.2
109	O=C(c1cc(n2CCCc12)C[C@H]3CCCC[N+]3C)c4cccc5ccccc54	8.2
110	O=C(c1c2cnsc2n(n1)C[C@H]3CCCC[N+]3C)c4cccc5ccccc54	8.2
111	Clc1cccc2c1n(nc2C[C@H]3CCCC[N+]3C)C(=O)c4cccc5ccccc54	8.2
112	Clc1cc(cc(C[C@H]2CCCC[N+]2C)c1)C(=O)c3cccc4ccccc43	8.2
113	O=C(c1ccc[n+](C[C@H]2CCCC[N+]2C)c1)c3cccc4ccccc43	8.2
114	O=C(c1c(c(c([nH]1)C[C@H]2CCCC[N+]2C)C)C)c3cccc4ccccc43	8.2
115	Oc1c(cc(C[C@H]2CCCC[N+]2C)cn1)C(=O)c3cccc4ccccc43	8.2
116	O=C(c1cn(C[C@H]2CCCC[N+]2C)c3cccc([N+]([O-])=O)c31)c4cccc5ccccc54	8.2
117	Clc1c(c(cc(C[C@H]2CCCC[N+]2C)c1)C(=O)c3cccc4ccccc43)C=O	8.1
118	O=C(n1c2C(SC=Cc2c(n1)C[C@H]3CCCC[N+]3C)=O)c4cccc5ccccc54	8.1
119	O=C(c1c2c(n(n1)C[C@H]3CCCC[N+]3C)ccs2)c4cccc5ccccc54	8.1
120	O=C(c1cn(C[C@H]2CCCC[N+]2C)c3c1cns3)c4cccc5ccccc54	8.1
121	Brc1cc(C[C@H]2CCCC[N+]2C)cc(c1C)C(=O)c3cccc4ccccc43	8.1
122	O=C(c1c(OC)nc(N)c(C[C@H]2CCCC[N+]2C)c1)c3cccc4ccccc43	8.1
123	O=C(c1cc(C[C@H]2CCCC[N+]2C)c3ccc4ccccc4n13)c5cccc6ccccc65	8.1
124	O=C(C1=CN(C[C@H]2CCCC[N+]2C)C3=CSC(=O)N31)c4cccc5ccccc54	8.1
125	O=C(c1c2c(sc(n2)N)cc(C[C@H]3CCCC[N+]3C)c1)c4cccc5ccccc54	8.1
126	O=C(c1c2ccccn2c(C[C@H]3CCCC[N+]3C)c1C)c4cccc5ccccc54	8.1
127	O=C(c1cn(C[C@H]2CCCC[N+]2C)c3cocc31)c4cccc5ccccc54	8.1
128	S=C1N(N=C(N1CC)C[C@H]2CCCC[N+]2C)C(=O)c3cccc4ccccc43	8.1
129	O=C(c1c2cnsc2c(s1)C[C@H]3CCCC[N+]3C)c4cccc5ccccc54	8.1
130	Oc1c(cc(C[C@H]2CCCC[N+]2C)cc1C)C(=O)c3cccc4ccccc43	8.1
131	O=C(c1c2ccsc2cc(C[C@H]3CCCC[N+]3C)c1)c4cccc5ccccc54	8.1
132	O=C(c1c(OC)ncc(C[C@H]2CCCC[N+]2C)c1)c3cccc4ccccc43	8.1
133	O=C(c1cc2c(c(C[C@H]3CCCC[N+]3C)c1)csn2)c4cccc5ccccc54	8.1
134	O=C(c1cn(C[C@H]2CCCC[N+]2C)c3cccnc31)c4cccc5ccccc54	8.1
135	O=C(c1c2c(cc(C[C@H]3CCCC[N+]3C)c1)csn2)c4cccc5ccccc54	8.1
136	O=C(c1c2-n(sccs2)c(C[C@H]3CCCC[N+]3C)c[nH]1)c4cccc5ccccc54	8.1
137	Oc1c(c2ccccc2n1C[C@H]3CCCC[N+]3C)C(=O)c4cccc5ccccc54	8.1
138	O=C(c1cc2c(c[nH]c2c(C[C@H]3CCCC[N+]3C)c1)C)c4cccc5ccccc54	8.1
139	Clc1c(cc(n1C)C[C@H]2CCCC[N+]2C)C(=O)c3cccc4ccccc43	8.1
140	O=C(c1cn(C[C@H]2CCCC[N+]2C)c3cnncc31)c4cccc5ccccc54	8.1
141	O=C(c1c2COCCc2n(n1)C[C@H]3CCCC[N+]3C)c4cccc5ccccc54	8.1
142	O=C(c1cn(C[C@H]2CCCC[N+]2C)c3ccccc31)c4cccc5ccccc54	8.1
143	O=C(c1c(c(n(n1)C[C@H]2CCCC[N+]2C)C)C)c3cccc4ccccc43	8.1
144	Clc1c(O)c(cc(C[C@H]2CCCC[N+]2C)c1O)C(=O)c3cccc4ccccc43	8.1
145	O=C(n1c2c(c(n1)C[C@H]3CCCC[N+]3C)cccn2)c4cccc5ccccc54	8.1
146	O=C(c1cn(C[C@H]2CCCC[N+]2C)c3C=CNC(=O)c31)c4cccc5ccccc54	8.1
147	O=C(c1cc(C[C@H]2CCCC[N+]2C)cc3c1OCCO3)c4cccc5ccccc54	8.1

148	Brc1cc(cc(C[C@H]2CCCC[N+]2C)c1O)C(=O)c3cccc4ccccc43	8.1
149	O=C(c1c2c(nncn2)cc(C[C@H]3CCCC[N+]3C)c1)c4cccc5ccccc54	8.1
150	O=C(c1cc(C[C@H]2CCCC[N+]2C)cc3c1OCC3)c4cccc5ccccc54	8.1
151	Brc1cc(c(F)c(C[C@H]2CCCC[N+]2C)c1)C(=O)c3cccc4ccccc43	8.1
152	O=C(c1cn(C[C@H]2CCCC[N+]2C)c3cnsc31)c4cccc5ccccc54	8.1
153	Brc1c(c(sc1C[C@H]2CCCC[N+]2C)C(=O)c3cccc4ccccc43)C	8.1
154	O=C(c1cc(n2ccncc12)C[C@H]3CCCC[N+]3C)c4cccc5ccccc54	8.1
155	O=C(c1cn(C[C@H]2CCCC[N+]2C)c3c1cn[nH]3)c4cccc5ccccc54	8.1
156	Fc1ccc2c(n(C[C@H]3CCCC[N+]3C)cc2C(=O)c4cccc5ccccc54)c1	8.1
157	O=C(c1c2c(scn2)cc(C[C@H]3CCCC[N+]3C)c1)c4cccc5ccccc54	8.1
158	FC(F)(F)c1c(O)c(cc(C[C@H]2CCCC[N+]2C)c1)C(=O)c3cccc4ccccc43	8.1
159	O=C(c1c2n(c(C[C@H]3CCCC[N+]3C)c1)cco2)c4cccc5ccccc54	8.1
160	O=C(c1c2cnsc2c([nH]1)C[C@H]3CCCC[N+]3C)c4cccc5ccccc54	8.1
161	O=C(c1cn(n2ccsn2s1)C[C@H]3CCCC[N+]3C)c4cccc5ccccc54	8.1
162	O=C(c1cn(C[C@H]2CCCC[N+]2C)c3cccc(c31)C)c4cccc5ccccc54	8.1
163	Fc1c(N)cc(C[C@H]2CCCC[N+]2C)cc1C(=O)c3cccc4ccccc43	8.1
164	O=C(c1cn(C[C@H]2CCCC[N+]2C)c3c1NC(S3)=O)c4cccc5ccccc54	8.1
165	Oc1c(CC)cc(cc1C[C@H]2CCCC[N+]2C)C(=O)c3cccc4ccccc43	8.1
166	O=C(c1cn(C[C@H]2CCCC[N+]2C)c3cccc(O)c31)c4cccc5ccccc54	8.1
167	O=C(c1c2c(c(s1)C[C@H]3CCCC[N+]3C)ccs2)c4cccc5ccccc54	8.1
168	Brc1c(Cl)c(cc(C[C@H]2CCCC[N+]2C)c1)C(=O)c3cccc4ccccc43	8.1
169	O=C(c1cn(C[C@H]2CCCC[N+]2C)cc3-n1sncs3)c4cccc5ccccc54	8.1
170	O=C(c1cn(C[C@H]2CCCC[N+]2C)c3c1C(=O)C=CS3)c4cccc5ccccc54	8.1
171	O=C(c1c2ccoc2c([nH]1)C[C@H]3CCCC[N+]3C)c4cccc5ccccc54	8.1
172	O=C(c1cn(C[C@H]2CCCC[N+]2C)c3cc[nH]c31)c4cccc5ccccc54	8.1
173	O=C(c1c2ccc(cc2n(n1)C[C@H]3CCCC[N+]3C)C)c4cccc5ccccc54	8.1
174	Clc1c(F)cc(C[C@H]2CCCC[N+]2C)cc1C(=O)c3cccc4ccccc43	8.1
175	Fc1cc(cc(C[C@H]2CCCC[N+]2C)c1)C(=O)c3cccc4ccccc43	8.1
176	O=C(c1c(N#C)ccc(C[C@H]2CCCC[N+]2C)c1)c3cccc4ccccc43	8
177	Clc1c(F)c(cc(C[C@H]2CCCC[N+]2C)c1)C(=O)c3cccc4ccccc43	8
178	O=C(c1cn(C[C@H]2CCCC[N+]2C)c3ccc([N+]([O-])=O)cc31)c4cccc5ccccc54	8
179	O=C(c1cn(C[C@H]2CCCC[N+]2C)c3c1cccn3)c4cccc5ccccc54	8
180	O=C(n1cc(C[C@H]2CCCC[N+]2C)c3C=CSC(=O)c31)c4cccc5ccccc54	8
181	Fc1cc(c(N)c(C[C@H]2CCCC[N+]2C)c1)C(=O)c3cccc4ccccc43	8
182	Brc1cc(c[n+](C[C@H]2CCCC[N+]2C)c1)C(=O)c3cccc4ccccc43	8
183	O=C(c1cc2cc[nH]c2c(C[C@H]3CCCC[N+]3C)c1)c4cccc5ccccc54	8
184	Fc1ccc2c(c(nn2C[C@H]3CCCC[N+]3C)C(=O)c4cccc5ccccc54)c1	8
185	O=C(c1cc2c(c([nH]c2c(C[C@H]3CCCC[N+]3C)c1)C)C)c4cccc5ccccc54	8
186	Clc1cccc2c1c(cn2C[C@H]3CCCC[N+]3C)C(=O)c4cccc5ccccc54	8
187	Clc1c(O)c(cc(C[C@H]2CCCC[N+]2C)c1)C(=O)c3cccc4ccccc43	8
188	O=C(c1c2c(n(n1)C[C@H]3CCCC[N+]3C)cccn2)c4cccc5ccccc54	8
189	Sc1c2c(n(C[C@H]3CCCC[N+]3C)cc2C(=O)c4cccc5ccccc54)ncn1	8

190	O=C(c1cn(C[C@H]2CCCC[N+]2C)c3cncn31)c4cccc5ccccc54	8
191	O=C(c1c2cc(ccc2n(n1)C[C@H]3CCCC[N+]3C)C)c4cccc5ccccc54	8
192	Brc1c(O)c(cc(C[C@H]2CCCC[N+]2C)c1O)C(=O)c3cccc4ccccc43	8
193	O=C(c1cn(n2[nH]ccn2s1)C[C@H]3CCCC[N+]3C)c4cccc5ccccc54	8
194	O=C(c1c-2[nH]ncsn2cc([nH]1)C[C@H]3CCCC[N+]3C)c4cccc5ccccc54	8
195	Clc1c(OC)cc(C[C@H]2CCCC[N+]2C)cc1C(=O)c3cccc4ccccc43	8
196	O=C(c1c(N)c(n2CCCc12)C[C@H]3CCCC[N+]3C)c4cccc5ccccc54	8
197	Brc1cc(O)c(C[C@H]2CCCC[N+]2C)cc1C(=O)c3cccc4ccccc43	8
198	O=C(c1cn(C[C@H]2CCCC[N+]2C)c3c1ncs3)c4cccc5ccccc54	8
199	O=C(c1cccc(C[C@H]2CCCC[N+]2C)c1)c3cccc4ccccc43	8
200	O=C(n1cc2-n([nH]ccs2)c(C[C@H]3CCCC[N+]3C)c1)c4cccc5ccccc54	8
201	OCc1c(c2ccccc2n1C[C@H]3CCCC[N+]3C)C(=O)c4cccc5ccccc54	8
202	O=C(c1cn(C[C@H]2CCCC[N+]2C)cc3-n1ocno3)c4cccc5ccccc54	8
203	O=C(c1c2cnccc2cc(C[C@H]3CCCC[N+]3C)c1)c4cccc5ccccc54	8
204	O=C(c1c2c(n(C[C@H]3CCCC[N+]3C)c1)cc(cn2)C)c4cccc5ccccc54	8
205	O=C(c1cc(OC)cc(C[C@H]2CCCC[N+]2C)c1)c3cccc4ccccc43	8
206	Fc1ccc2c(c(cn2C[C@H]3CCCC[N+]3C)C(=O)c4cccc5ccccc54)c1	8
207	Fc1c(O)c(cc(C[C@H]2CCCC[N+]2C)c1)C(=O)c3cccc4ccccc43	8
208	O=C(c1cc(C[C@H]2CCCC[N+]2C)cc3ccnn31)c4cccc5ccccc54	8
209	O=C(c1c2c(c(o1)C[C@H]3CCCC[N+]3C)ccs2)c4cccc5ccccc54	8
210	O=C(n1cc(C[C@H]2CCCC[N+]2C)c3C=CNC(=O)c31)c4cccc5ccccc54	8
211	O=C(c1cn(C[C@H]2CCCC[N+]2C)c3c1c(O)ncn3)c4cccc5ccccc54	8
212	O=C(c1cc(SC)cc(C[C@H]2CCCC[N+]2C)c1)c3cccc4ccccc43	8
213	O=C(c1cn(C[C@H]2CCCC[N+]2C)c3csnc31)c4cccc5ccccc54	8
214	O=C(c1cn(C[C@H]2CCCC[N+]2C)c3cccc(OC)c31)c4cccc5ccccc54	8
215	O=C(c1cc(C[C@H]2CCCC[N+]2C)cc3cccn31)c4cccc5ccccc54	8
216	O=C(c1cn(C[C@H]2CCCC[N+]2C)c3c1cco3)c4cccc5ccccc54	8
217	O=C(c1cn(C[C@H]2CCCC[N+]2C)c3ccsc31)c4cccc5ccccc54	8
218	O=C(c1cc(C[C@H]2CCCC[N+]2C)c3C=CSC(=O)n31)c4cccc5ccccc54	8
219	O=C(c1c2C(SC=Cc2c(o1)C[C@H]3CCCC[N+]3C)=O)c4cccc5ccccc54	8
220	FC(F)(F)Cn1c(c(cc1C[C@H]2CCCC[N+]2C)C(=O)c3cccc4ccccc43)C	8
221	O=C(c1cn(C[C@H]2CCCC[N+]2C)c3c1cncn3)c4cccc5ccccc54	8
222	O=C(c1cc(n(C[C@H]2CCCC[N+]2C)c1)C)c3cccc4ccccc43	8
223	Clc1ccn2c(c(cc2C[C@H]3CCCC[N+]3C)C(=O)c4cccc5ccccc54)c1	8
224	O=C(c1cc(NC)cc(C[C@H]2CCCC[N+]2C)c1)c3cccc4ccccc43	8
225	O=C(c1c2ccsc2n(n1)C[C@H]3CCCC[N+]3C)c4cccc5ccccc54	8
226	O=C(c1c(OCC)ncc(C[C@H]2CCCC[N+]2C)c1)c3cccc4ccccc43	8
227	O=C(c1cn(C[C@H]2CCCC[N+]2C)c3c1ccs3)c4cccc5ccccc54	8
228	O=C(c1c2n(ncs2)c(C[C@H]3CCCC[N+]3C)c1)c4cccc5ccccc54	8
229	O=C(c1cc(n(c1C)CC=C)C[C@H]2CCCC[N+]2C)c3cccc4ccccc43	8
230	Clc1ccc2c(c(nn2C[C@H]3CCCC[N+]3C)C(=O)c4cccc5ccccc54)c1	8
231	Clc1c2c(n(C[C@H]3CCCC[N+]3C)cc2C(=O)c4cccc5ccccc54)ncn1	8

232	O=C(c1c2c(c([nH]1)C[C@H]3CCCC[N+]3C)ccs2)c4cccc5ccccc54	8
233	O=C(c1c2n(c(n1)C[C@H]3CCCC[N+]3C)ccs2)c4cccc5ccccc54	8
234	O=C(c1cn(C[C@H]2CCCC[N+]2C)cc3-n1snco3)c4cccc5ccccc54	8
235	O=C(c1cn(C[C@H]2CCCC[N+]2C)c3ccc(OCC)cc31)c4cccc5ccccc54	8
236	Fc1ccc(C[C@H]2CCCC[N+]2C)c(O)c1C(=O)c3cccc4ccccc43	8
237	O=C(c1cn(n2[nH]ccsn12)C[C@H]3CCCC[N+]3C)c4cccc5ccccc54	8
238	O=C(c1c(n(C[C@H]2CCCC[N+]2C)c(c1C)C)N)c3cccc4ccccc43	8
239	O=C(c1c2cccn2n(n1)C[C@H]3CCCC[N+]3C)c4cccc5ccccc54	8
240	Clc1c(N)c(cc(C[C@H]2CCCC[N+]2C)c1)C(=O)c3cccc4ccccc43	8
241	Clc1c(Cl)cc(C[C@H]2CCCC[N+]2C)cc1C(=O)c3cccc4ccccc43	8
242	FC(F)c1cc(nn1C[C@H]2CCCC[N+]2C)C(=O)c3cccc4ccccc43	8
243	Clc1c(OCC)c(cc(C[C@H]2CCCC[N+]2C)c1)C(=O)c3cccc4ccccc43	8
244	O=C(c1c2cnccc2c(s1)C[C@H]3CCCC[N+]3C)c4cccc5ccccc54	8
245	Fc1c(OCC)cc(C[C@H]2CCCC[N+]2C)cc1C(=O)c3cccc4ccccc43	8
246	O=C(c1cc(N)c([nH]1)C[C@H]2CCCC[N+]2C)c3cccc4ccccc43	8
247	O=C(n1c[n+](C[C@H]2CCCC[N+]2C)c3ccccc31)c4cccc5ccccc54	8
248	Fc1ccc2c(c(n(C[C@H]3CCCC[N+]3C)c2c1)C)C(=O)c4cccc5ccccc54	7.9
249	O=C(c1cc(C[C@H]2CCCC[N+]2C)cc(C(C)(C)C)c1)c3cccc4ccccc43	7.9
250	O=C(c1c2C(OC=Cc2c([nH]1)C[C@H]3CCCC[N+]3C)=O)c4cccc5ccccc54	7.9
251	O=C(c1cn(C[C@H]2CCCC[N+]2C)c3cc4c(OCO4)cc31)c5cccc6ccccc65	7.9
252	O=C(c1cn(C[C@H]2CCCC[N+]2C)c3ccc(cc31)-c4ccco4)c5cccc6ccccc65	7.9
253	O=C(c1cn(C[C@H]2CCCC[N+]2C)c3ccc(OC(C)C)cc31)c4cccc5ccccc54	7.9
254	Oc1c(c2ccccc2cc1C[C@H]3CCCC[N+]3C)C(=O)c4cccc5ccccc54	7.9
255	Clc1cc(c(N)c(C[C@H]2CCCC[N+]2C)c1)C(=O)c3cccc4ccccc43	7.9
256	O=C(c1c2c(onn2)cc(C[C@H]3CCCC[N+]3C)c1)c4cccc5ccccc54	7.9
257	Clc1cc(C[C@H]2CCCC[N+]2C)cc(c1C)C(=O)c3cccc4ccccc43	7.9
258	O=C1N(c2cscc2N1C[C@H]3CCCC[N+]3C)C(=O)c4cccc5ccccc54	7.9
259	O=C(c1c2c(scn2)c([nH]1)C[C@H]3CCCC[N+]3C)c4cccc5ccccc54	7.9
260	O=C(c1c2c(OCO2)cc(C[C@H]3CCCC[N+]3C)c1)c4cccc5ccccc54	7.9
261	Fc1c(c(cc(C[C@H]2CCCC[N+]2C)c1)C(=O)c3cccc4ccccc43)C#N	7.9
262	O=C(c1cn(C[C@H]2CCCC[N+]2C)cc3-n1oncs3)c4cccc5ccccc54	7.9
263	Oc1ccc(cc1C[C@H]2CCCC[N+]2C)C(=O)c3cccc4ccccc43	7.9
264	Fc1cc(c(O)c(C[C@H]2CCCC[N+]2C)c1)C(=O)c3cccc4ccccc43	7.9
265	Clc1cc(c(O)c(C[C@H]2CCCC[N+]2C)c1)C(=O)c3cccc4ccccc43	7.9
266	O=C(c1cn(n2cncc12)C[C@H]3CCCC[N+]3C)c4cccc5ccccc54	7.9
267	O=C(c1cn(C[C@H]2CCCC[N+]2C)c3ccc(cc31)CC)c4cccc5ccccc54	7.9
268	Fc1c(OC)cc(C[C@H]2CCCC[N+]2C)cc1C(=O)c3cccc4ccccc43	7.9
269	O=C(c1cn(C[C@H]2CCCC[N+]2C)c3c1nccn3)c4cccc5ccccc54	7.9
270	O=C(c1c2cc3c(OCCO3)cc2n(C[C@H]4CCCC[N+]4C)c1)c5cccc6ccccc65	7.9
271	O=C(c1cc(cc(C[C@H]2CCCC[N+]2C)c1N)C)c3cccc4ccccc43	7.9
272	O=C(c1cc2CCCNc2c(C[C@H]3CCCC[N+]3C)c1)c4cccc5ccccc54	7.9
273	O=C(c1c2ccncc2c([nH]1)C[C@H]3CCCC[N+]3C)c4cccc5ccccc54	7.9

274	O=C(c1c2c(cc(C[C@H]3CCCC[N+]3C)c1)ccc(O)n2)c4cccc5ccccc54	7.9
275	O=C(c1cn(C[C@H]2CCCC[N+]2C)c3cc(ccc13)C(=O)N)c4cccc5ccccc54	7.9
276	O=C(c1c2c(nnnn2)cc(C[C@H]3CCCC[N+]3C)c1)c4cccc5ccccc54	7.9
277	O=C(N1C(N(C[C@H]2CCCC[N+]2C)c3ccccc31)=N)c4cccc5ccccc54	7.9
278	Clc1ccc2c(nn(C[C@H]3CCCC[N+]3C)c2c1)C(=O)c4cccc5ccccc54	7.9
279	O=C(c1nn(C[C@H]2CCCC[N+]2C)c3cccn13)c4cccc5ccccc54	7.9
280	O=C(c1cc(cc(C[C@H]2CCCC[N+]2C)c1)CC)c3cccc4ccccc43	7.9
281	O=C(c1cn(n2cnsn2s1)C[C@H]3CCCC[N+]3C)c4cccc5ccccc54	7.9
282	Clc1ccc2c(c(cn2C[C@H]3CCCC[N+]3C)C(=O)c4cccc5ccccc54)c1	7.9
283	O=C(c1c2C(SC=Cc2c(s1)C[C@H]3CCCC[N+]3C)=O)c4cccc5ccccc54	7.9
284	O=C(c1cn(C[C@H]2CCCC[N+]2C)c3ccoc31)c4cccc5ccccc54	7.9
285	O=C(c1cc2c(ocn2)c(C[C@H]3CCCC[N+]3C)c1)c4cccc5ccccc54	7.9
286	O=C(c1cn(C[C@H]2CCCC[N+]2C)cc3-n1onco3)c4cccc5ccccc54	7.9
287	O=C(c1c2ccccc2c(s1)C[C@H]3CCCC[N+]3C)c4cccc5ccccc54	7.9
288	O=C(c1c2cncn2n(n1)C[C@H]3CCCC[N+]3C)c4cccc5ccccc54	7.9
289	Clc1c(c2cc(F)ccc2n1C[C@H]3CCCC[N+]3C)C(=O)c4cccc5ccccc54	7.9
290	Clc1c(cc(C[C@H]2CCCC[N+]2C)cc1CC)C(=O)c3cccc4ccccc43	7.9
291	O=C(n1cc(C[C@H]2CCCC[N+]2C)c3C=COC(=O)c31)c4cccc5ccccc54	7.9
292	Clc1cc(c(c(C[C@H]2CCCC[N+]2C)c1)C)C(=O)c3cccc4ccccc43	7.9
293	O=C(c1c2C=CCCc2c(s1)C[C@H]3CCCC[N+]3C)c4cccc5ccccc54	7.9
294	O=C(c1cn(C[C@H]2CCCC[N+]2C)c3cc(ccc13)C)c4cccc5ccccc54	7.9
295	O=C(c1c2c(nc(C[C@H]3CCCC[N+]3C)c1)cccn2)c4cccc5ccccc54	7.9
296	O=C(c1c2c(OCCO2)c(s1)C[C@H]3CCCC[N+]3C)c4cccc5ccccc54	7.9
297	Clc1c(Cl)c(sc1C[C@H]2CCCC[N+]2C)C(=O)c3cccc4ccccc43	7.9
298	O=C(c1cc(C[C@H]2CCCC[N+]2C)cc3c1cc[nH]3)c4cccc5ccccc54	7.9
299	O=C(c1c2c(c(s1)C[C@H]3CCCC[N+]3C)cc[nH]2)c4cccc5ccccc54	7.9
300	O=C(c1cc(cc(C[C@H]2CCCC[N+]2C)c1)C)c3cccc4ccccc43	7.9
301	O=C(c1cc(c([nH]1)C[C@H]2CCCC[N+]2C)C)c3cccc4ccccc43	7.9
302	O=C(c1c-2[nH]ccsn2cc([nH]1)C[C@H]3CCCC[N+]3C)c4cccc5ccccc54	7.9
303	O=C(c1cc(n2ccc(cc12)C)C[C@H]3CCCC[N+]3C)c4cccc5ccccc54	7.9
304	O=C(c1cc(C[C@H]2CCCC[N+]2C)cc3c(cccc31)C)c4cccc5ccccc54	7.9
305	FC(F)Oc1ccc2c(c(nn2C[C@H]3CCCC[N+]3C)C(=O)c4cccc5ccccc54)c1	7.9
306	O=C(c1cn(C[C@H]2CCCC[N+]2C)c3CCCc13)c4cccc5ccccc54	7.9
307	O=C(c1cn(C[C@H]2CCCC[N+]2C)c3cn[nH]c31)c4cccc5ccccc54	7.9
308	O=C(c1cc(C[C@H]2CCCC[N+]2C)c3ccnnn31)c4cccc5ccccc54	7.9
309	O=C(C=1C2=CSC(=O)N2C=C(C[C@H]3CCCC[N+]3C)C1)c4cccc5ccccc54	7.9
310	O=C(c1cn(C[C@H]2CCCC[N+]2C)cc3-n1onno3)c4cccc5ccccc54	7.9
311	Brc1cc(c(N)c(C[C@H]2CCCC[N+]2C)c1)C(=O)c3cccc4ccccc43	7.9
312	O=C(c1c2C(=O)C=CSc2c([nH]1)C[C@H]3CCCC[N+]3C)c4cccc5ccccc54	7.9
313	O=C(c1cc2c([nH]cn2)c(C[C@H]3CCCC[N+]3C)c1)c4cccc5ccccc54	7.9
314	O=S(=O)(c1cc(cc(C[C@H]2CCCC[N+]2C)c1)C(=O)c3cccc4ccccc43)C	7.9
315	O=C(c1c2cc(N)ccc2n(n1)C[C@H]3CCCC[N+]3C)c4cccc5ccccc54	7.9

316	O=C(c1cn(C[C@H]2CCCC[N+]2C)c3cccn31)c4cccc5ccccc54	7.9
317	O=C(c1c2c(ncnn2)cc(C[C@H]3CCCC[N+]3C)c1)c4cccc5ccccc54	7.9
318	Brc1c(cn(n1)C[C@H]2CCCC[N+]2C)C(=O)c3cccc4ccccc43	7.9
319	Clc1ccc2c(n(C[C@H]3CCCC[N+]3C)cc2C(=O)c4cccc5ccccc54)c1	7.9
320	Fc1c(N)c(cc(C[C@H]2CCCC[N+]2C)c1)C(=O)c3cccc4ccccc43	7.9
321	O=C(c1cn(C[C@H]2CCCC[N+]2C)c3ccc(OC)cc31)c4cccc5ccccc54	7.9
322	Brc1c(Cl)cc(C[C@H]2CCCC[N+]2C)cc1C(=O)c3cccc4ccccc43	7.9
323	O=C(c1cn(C[C@H]2CCCC[N+]2C)c3c(O)cccc31)c4cccc5ccccc54	7.9
324	O=C(c1c2ccnn2cc(C[C@H]3CCCC[N+]3C)c1)c4cccc5ccccc54	7.9
325	O=C(c1cc(C[C@H]2CCCC[N+]2C)cc3c1ccn3CC)c4cccc5ccccc54	7.9
326	O=C(c1cc[n+](C[C@H]2CCCC[N+]2C)c3cc(N)ccc13)c4cccc5ccccc54	7.8
327	Clc1c(N)cc(C[C@H]2CCCC[N+]2C)cc1C(=O)c3cccc4ccccc43	7.8
328	O=C(c1cc(C[C@H]2CCCC[N+]2C)cc3c1cc(o3)C)c4cccc5ccccc54	7.8
329	Clc1cccc2c1c(nn2C[C@H]3CCCC[N+]3C)C(=O)c4cccc5ccccc54	7.8
330	O=C(c1cn(C[C@H]2CCCC[N+]2C)c3c(ccc(c31)C)C)c4cccc5ccccc54	7.8
331	O=C(c1cn(C[C@H]2CCCC[N+]2C)c3cc(O)ccc31)c4cccc5ccccc54	7.8
332	O=C(c1cn(n2c1ccn2)C[C@H]3CCCC[N+]3C)c4cccc5ccccc54	7.8
333	Clc1c(Cl)c([nH]c1C[C@H]2CCCC[N+]2C)C(=O)c3cccc4ccccc43	7.8
334	Clc1c(Cl)c(O)c(C[C@H]2CCCC[N+]2C)cc1C(=O)c3cccc4ccccc43	7.8
335	Clc1ccc2c(c(n(C[C@H]3CCCC[N+]3C)c2c1)C)C(=O)c4cccc5ccccc54	7.8
336	Clc1c(cc(C[C@H]2CCCC[N+]2C)cc1C)C(=O)c3cccc4ccccc43	7.8
337	O=C(c1cc(C[C@H]2CCCC[N+]2C)c(s1)CCC)c3cccc4ccccc43	7.8
338	Fc1c(C(=O)c2cccc3ccccc32)cc(F)cc1C[C@H]4CCCC[N+]4C	7.8
339	Brc1c(c([nH]c1C[C@H]2CCCC[N+]2C)C(=O)c3cccc4ccccc43)C#N	7.8
340	O=C(c1c2n(c(C[C@H]3CCCC[N+]3C)c1)ccs2)c4cccc5ccccc54	7.8
341	O=C(c1cn(C[C@H]2CCCC[N+]2C)c3c[nH]cc13)c4cccc5ccccc54	7.8
342	Clc1ccc2c(n(C[C@H]3CCCC[N+]3C)cc2C(=O)c4cccc5ccccc54)c1C	7.8
343	O=C(c1cc[n+](C[C@H]2CCCC[N+]2C)c3ccccc13)c4cccc5ccccc54	7.8
344	O=C(c1c2cscc2cc(C[C@H]3CCCC[N+]3C)c1)c4cccc5ccccc54	7.8
345	O=C(c1cn(C[C@H]2CCCC[N+]2C)c3c1cnc(n3)C)c4cccc5ccccc54	7.8
346	Fc1c(cc(C[C@H]2CCCC[N+]2C)cc1C)C(=O)c3cccc4ccccc43	7.8
347	Clc1c(OC)cc2c(n(C[C@H]3CCCC[N+]3C)cc2C(=O)c4cccc5ccccc54)c1	7.8
348	O=C(c1cc(n(C[C@H]2CCCC[N+]2C)c1)C[N+]C)c3cccc4ccccc43	7.8
349	O=C(c1cc(c(N)c(C[C@H]2CCCC[N+]2C)c1)C(=O)N)c3cccc4ccccc43	7.8
350	O=C(c1c2ccccc2c([nH]1)C[C@H]3CCCC[N+]3C)c4cccc5ccccc54	7.8
351	O=C(c1cc(C[C@H]2CCCC[N+]2C)cc(n1)C)c3cccc4ccccc43	7.8
352	O=C(c1c2c(cc(C[C@H]3CCCC[N+]3C)c1)ccs2)c4cccc5ccccc54	7.8
353	O=C(c1c2c([C@H]3CC[C@@H]2C3)c([nH]1)C[C@H]4CCCC[N+]4C)c5cccc6ccccc65	7.8
354	Fc1c(F)cc(C[C@H]2CCCC[N+]2C)c(O)c1C(=O)c3cccc4ccccc43	7.8
355	O=C(c1cc(c[n+](C[C@H]2CCCC[N+]2C)c1)C)c3cccc4ccccc43	7.8
356	O=C(c1cc(C[C@H]2CCCC[N+]2C)cc3c1ccn3C)c4cccc5ccccc54	7.8
357	O=C(c1cn(C[C@H]2CCCC[N+]2C)c3c1ccc(n3)C)c4cccc5ccccc54	7.8

358	O=C(c1c(nc(s1)C[C@H]2CCCC[N+]2C)CC(C)C)c3cccc4ccccc43	7.8
359	O=C(c1c(n(C[C@H]2CCCC[N+]2C)c3ccccc13)C=O)c4cccc5ccccc54	7.8
360	O=C(c1cn(C[C@H]2CCCC[N+]2C)c3cc(c(cc13)C)C)c4cccc5ccccc54	7.8
361	O=C(c1cn(C[C@H]2CCCC[N+]2C)c3ccc(C(C)(C)C)cc13)c4cccc5ccccc54	7.8
362	O=C(c1cc(C[C@H]2CCCC[N+]2C)cc(C(C)C)c1)c3cccc4ccccc43	7.8
363	O=C1CCCc2c1c(sc2C[C@H]3CCCC[N+]3C)C(=O)c4cccc5ccccc54	7.8
364	O=C(c1c2C[C@@H]3C[C@@H]3c2n(n1)C[C@H]4CCCC[N+]4C)c5cccc6ccccc65	7.8
365	O=C(c1c2C(=O)C=CSc2c(s1)C[C@H]3CCCC[N+]3C)c4cccc5ccccc54	7.8
366	O=C(c1c2c(ncs2)cc(C[C@H]3CCCC[N+]3C)c1)c4cccc5ccccc54	7.8
367	O=C(c1c2ccoc2cc(C[C@H]3CCCC[N+]3C)c1)c4cccc5ccccc54	7.8
368	Clc1c(c2cc(OC)ccc2n1C[C@H]3CCCC[N+]3C)C(=O)c4cccc5ccccc54	7.8
369	Oc1c(sc(c1C[C@H]2CCCC[N+]2C)C)C(=O)c3cccc4ccccc43	7.8
370	Oc1c(c2cc(ccc2n1C[C@H]3CCCC[N+]3C)C)C(=O)c4cccc5ccccc54	7.8
371	O=C(c1cn(C[C@H]2CCCC[N+]2C)c(n1)CCC)c3cccc4ccccc43	7.8
372	O=C(c1cn(n2-c(s1)cocn2)C[C@H]3CCCC[N+]3C)c4cccc5ccccc54	7.7
373	Clc1cc(cc(C[C@H]2CCCC[N+]2C)c1CC)C(=O)c3cccc4ccccc43	7.7
374	Clc1cc(C[C@H]2CCCC[N+]2C)cc(n1)C(=O)c3cccc4ccccc43	7.7
375	FC(F)(F)c1ccc2c(c(cn2C[C@H]3CCCC[N+]3C)C(=O)c4cccc5ccccc54)c1	7.7
376	O[C@@H]1CCCc2c1c(nn2C[C@H]3CCCC[N+]3C)C(=O)c4cccc5ccccc54	7.7
377	O=C(c1cn(C[C@H]2CCCC[N+]2C)c3nccn31)c4cccc5ccccc54	7.7
378	O=C(c1cn(C[C@H]2CCCC[N+]2C)c3c1c(O)nc(n3)N)c4cccc5ccccc54	7.7
379	O=C(c1c2ccccc2cc(C[C@H]3CCCC[N+]3C)c1)c4cccc5ccccc54	7.7
380	O=C(c1cn(C[C@H]2CCCC[N+]2C)c3c1csn3)c4cccc5ccccc54	7.7
381	Clc1c(c2ccc(Cl)cc2n1C[C@H]3CCCC[N+]3C)C(=O)c4cccc5ccccc54	7.7
382	Fc1c(cc(C[C@H]2CCCC[N+]2C)cc1CO)C(=O)c3cccc4ccccc43	7.7
383	O=C(c1cc(c(o1)C[C@H]2CCCC[N+]2C)CC#N)c3cccc4ccccc43	7.7
384	O=C(c1c2c(nno2)cc(C[C@H]3CCCC[N+]3C)c1)c4cccc5ccccc54	7.7
385	Brc1cc(cn1C[C@H]2CCCC[N+]2C)C(=O)c3cccc4ccccc43	7.7
386	Clc1c(Cl)cc(C[C@H]2CCCC[N+]2C)c(O)c1C(=O)c3cccc4ccccc43	7.7
387	O=C1C(=C2C(SC=CS2)=C1C[C@H]3CCCC[N+]3C)C(=O)c4cccc5ccccc54	7.7
388	O=C(c1cn(C[C@H]2CCCC[N+]2C)c3c1c(OC)nc(n3)N)c4cccc5ccccc54	7.7
389	O=C1C(=C2C(OC=CS2)=C1C[C@H]3CCCC[N+]3C)C(=O)c4cccc5ccccc54	7.7
390	Brc1c(N)c(cc(C[C@H]2CCCC[N+]2C)c1)C(=O)c3cccc4ccccc43	7.7
391	Oc1c(N)cc(cc1C[C@H]2CCCC[N+]2C)C(=O)c3cccc4ccccc43	7.7
392	Clc1c(c2ccc(cc2n1C[C@H]3CCCC[N+]3C)C)C(=O)c4cccc5ccccc54	7.7
393	Clc1c(c2cc(c(cc2n1C[C@H]3CCCC[N+]3C)C)C)C(=O)c4cccc5ccccc54	7.7
394	O=C(c1c2c(c([nH]1)C[C@H]3CCCC[N+]3C)cccn2)c4cccc5ccccc54	7.7
395	Clc1c(c2c(cc(cc2n1C[C@H]3CCCC[N+]3C)C)C)C(=O)c4cccc5ccccc54	7.7
396	Oc1c(C(=O)c2cccc3ccccc32)cc(cc1C[C@H]4CCCC[N+]4C)C	7.7
397	O=C(c1cc(C[C@H]2CCCC[N+]2C)cc3c[nH]cc31)c4cccc5ccccc54	7.7
398	Clc1c(C(=O)c2cccc3ccccc32)cc(Cl)cc1C[C@H]4CCCC[N+]4C	7.7
399	O=C(c1cn(n2ncsn2s1)C[C@H]3CCCC[N+]3C)c4cccc5ccccc54	7.7

400	O=C(c1cn(C[C@H]2CCCC[N+]2C)cc3-n1occo3)c4cccc5ccccc54	7.7
401	FC(F)(F)c1ccc2c(cn(C[C@H]3CCCC[N+]3C)c2c1)C(=O)c4cccc5ccccc54	7.7
402	Clc1cc(c(F)c(C[C@H]2CCCC[N+]2C)c1)C(=O)c3cccc4ccccc43	7.7
403	O=C(c1cn(C[C@H]2CCCC[N+]2C)c3c1c(OC)ncn3)c4cccc5ccccc54	7.7
404	O=C(c1c2c(nccn2)cc(C[C@H]3CCCC[N+]3C)c1)c4cccc5ccccc54	7.7
405	O=C(c1cn(C[C@H]2CCCC[N+]2C)c3CC[N+]Cc13)c4cccc5ccccc54	7.7
406	O=C(n1cc(C[C@H]2CCCC[N+]2C)c3c1C(=O)C=CS3)c4cccc5ccccc54	7.7
407	Clc1c(OC)c(O)c(C[C@H]2CCCC[N+]2C)cc1C(=O)c3cccc4ccccc43	7.7
408	O=C(c1cn(C[C@H]2CCCC[N+]2C)c3ccc(cc31)C)c4cccc5ccccc54	7.7
409	Fc1ccc2c(c(c(n2C[C@H]3CCCC[N+]3C)C)C(=O)c4cccc5ccccc54)c1	7.7
410	O=C(c1cc(C[C@H]2CCCC[N+]2C)cc3c1nc(O)cc3C)c4cccc5ccccc54	7.6
411	O=C(c1cn(C[C@H]2CCCC[N+]2C)c3ccc(O)cc31)c4cccc5ccccc54	7.6
412	Brc1cc(C[C@H]2CCCC[N+]2C)cc(n1)C(=O)c3cccc4ccccc43	7.6
413	Fc1cccc2c1n(C[C@H]3CCCC[N+]3C)cc2C(=O)c4cccc5ccccc54	7.6
414	O=C(c1cc(n2cccc2n1)C[C@H]3CCCC[N+]3C)c4cccc5ccccc54	7.6
415	O=C(c1cn(C[C@H]2CCCC[N+]2C)c3ccc(C(C)C)cc13)c4cccc5ccccc54	7.6
416	O=C(c1cn(C[C@H]2CCCC[N+]2C)c3c1scn3)c4cccc5ccccc54	7.6
417	O=C(c1cn(C[C@H]2CCCC[N+]2C)cc3-n1occs3)c4cccc5ccccc54	7.6
418	O=C(c1cc(C[C@H]2CCCC[N+]2C)cc3c1OCCC3)c4cccc5ccccc54	7.6
419	O=C(n1c([n+](C[C@H]2CCCC[N+]2C)c3ccccc31)C)c4cccc5ccccc54	7.6
420	O=C(c1c2c(OC)cccc2n(n1)C[C@H]3CCCC[N+]3C)c4cccc5ccccc54	7.6
421	Clc1c(OC)c(cc(C[C@H]2CCCC[N+]2C)c1)C(=O)c3cccc4ccccc43	7.6
422	O=C(c1c2cc(OC)c(OC)cc2n(C[C@H]3CCCC[N+]3C)c1)c4cccc5ccccc54	7.6
423	O=C(c1c(n(C[C@H]2CCCC[N+]2C)c3ccc(cc13)C)C)c4cccc5ccccc54	7.6
424	Oc1c(cc(C[C@H]2CCCC[N+]2C)cc1CO)C(=O)c3cccc4ccccc43	7.6
425	O=C(c1cc(OCC)cc(C[C@H]2CCCC[N+]2C)c1)c3cccc4ccccc43	7.6
426	O=C(c1c(OC)c(cc(C[C@H]2CCCC[N+]2C)c1)CC)c3cccc4ccccc43	7.6
427	O=C(c1cc(n(C2CC2)c1)C[C@H]3CCCC[N+]3C)c4cccc5ccccc54	7.6
428	Clc1c(c2ccccc2n1C[C@H]3CCCC[N+]3C)C(=O)c4cccc5ccccc54	7.6
429	O=C(c1c2cccc(O)c2n(C[C@H]3CCCC[N+]3C)c1C)c4cccc5ccccc54	7.6
430	O=C(c1cc2c(c(C[C@H]3CCCC[N+]3C)c1)c[nH]n2)c4cccc5ccccc54	7.6
431	O=C(c1cc(C[C@H]2CCCC[N+]2C)cc3c1nccc3C)c4cccc5ccccc54	7.6
432	O=C(c1c2ccsc2n(C[C@H]3CCCC[N+]3C)c1C)c4cccc5ccccc54	7.6
433	O=C(c1cn(C[C@H]2CCCC[N+]2C)c3c1ccc(c3C)C)c4cccc5ccccc54	7.6
434	Clc1cc(c(O)c(C[C@H]2CCCC[N+]2C)c1O)C(=O)c3cccc4ccccc43	7.6
435	Clc1cccc2c1n(C[C@H]3CCCC[N+]3C)cc2C(=O)c4cccc5ccccc54	7.6
436	Oc1c2ccccc2c(cc1C[C@H]3CCCC[N+]3C)C(=O)c4cccc5ccccc54	7.6
437	O=C(c1cn(C[C@H]2CCCC[N+]2C)c3c(cccc31)C)c4cccc5ccccc54	7.6
438	O=C(c1cn(C[C@H]2CCCC[N+]2C)c(SC)n1)c3cccc4ccccc43	7.6
439	Clc1ccc2c(c(c(n2C[C@H]3CCCC[N+]3C)C)C(=O)c4cccc5ccccc54)c1	7.6
440	O=C(c1cc(cc(C[C@H]2CCCC[N+]2C)c1)COC)c3cccc4ccccc43	7.6
441	Oc1c(cc(OC)cc1C[C@H]2CCCC[N+]2C)C(=O)c3cccc4ccccc43	7.6

442	O=C(c1cn(C[C@H]2CCCC[N+]2C)cc3-n1scco3)c4cccc5ccccc54	7.6
443	Brc1cc(c(O)c(C[C@H]2CCCC[N+]2C)c1)C(=O)c3cccc4ccccc43	7.6
444	O=C(c1c2cc(OC)ccc2n(C[C@H]3CCCC[N+]3C)c1C)c4cccc5ccccc54	7.6
445	O=C(c1cn(C[C@H]2CCCC[N+]2C)c3cc(OC)cc(OC)c31)c4cccc5ccccc54	7.6
446	O=C(c1c2ccccc2n(C[C@H]3CCCC[N+]3C)c1C)c4cccc5ccccc54	7.6
447	O=C(c1cn(C[C@H]2CCCC[N+]2C)c(C[N+])c1)c3cccc4ccccc43	7.6
448	O=C(c1cn(C[C@H]2CCCC[N+]2C)c3cc(OC)ccc31)c4cccc5ccccc54	7.6
449	Oc1c(C(=O)c2cccc3ccccc32)ccc(O)c1C[C@H]4CCCC[N+]4C	7.5
450	O=C(c1cn(C[C@H]2CCCC[N+]2C)c3c1cccc3CC)c4cccc5ccccc54	7.5
451	O=C(c1c2cc(O)ccc2n(C[C@H]3CCCC[N+]3C)c1C)c4cccc5ccccc54	7.5
452	O=C(C1=CN(C[C@H]2CCCC[N+]2C)C=3C(=O)C=CC3S1)c4cccc5ccccc54	7.5
453	Clc1c(c2cc(ccc2n1C[C@H]3CCCC[N+]3C)C)C(=O)c4cccc5ccccc54	7.5
454	O=C(c1cn(n2n1occs2)C[C@H]3CCCC[N+]3C)c4cccc5ccccc54	7.5
455	O=C(c1c2cc3c(OCO3)cc2n(C[C@H]4CCCC[N+]4C)c1C)c5cccc6ccccc65	7.5
456	O=C(c1cc(C[C@H]2CCCC[N+]2C)c(s1)CC)c3cccc4ccccc43	7.5
457	Brc1cc(cc(C[C@H]2CCCC[N+]2C)c1NC)C(=O)c3cccc4ccccc43	7.5
458	Brc1cc(c(O)c(C[C@H]2CCCC[N+]2C)c1O)C(=O)c3cccc4ccccc43	7.5
459	Oc1c(C(=O)c2cccc3ccccc32)cc(N)c(O)c1C[C@H]4CCCC[N+]4C	7.5
460	O=C(c1cc2CCNc2c(C[C@H]3CCCC[N+]3C)c1)c4cccc5ccccc54	7.5
461	Clc1cc(cc(C[C@H]2CCCC[N+]2C)c1NC)C(=O)c3cccc4ccccc43	7.5
462	Clc1c(c2cc(CC)ccc2n1C[C@H]3CCCC[N+]3C)C(=O)c4cccc5ccccc54	7.5
463	O=C(c1c(N)c(C[C@H]2CCCC[N+]2C)c(s1)C)c3cccc4ccccc43	7.5
464	O=C(c1c(n(C[C@H]2CCCC[N+]2C)c3ccc(cc13)CC)C)c4cccc5ccccc54	7.5
465	O=C(c1cn(C[C@H]2CCCC[N+]2C)c3c(OC)cc(cc13)C)c4cccc5ccccc54	7.5
466	O=C(c1c2ccc(cc2n(C[C@H]3CCCC[N+]3C)c1C)C)c4cccc5ccccc54	7.5
467	O=C(c1cn(C[C@H]2CCCC[N+]2C)c3c1ccc(OC)n3)c4cccc5ccccc54	7.5
468	OCc1cc(cn1C[C@H]2CCCC[N+]2C)C(=O)c3cccc4ccccc43	7.4
469	O[C@@H](c1cc(cc(C[C@H]2CCCC[N+]2C)c1)C(=O)c3cccc4ccccc43)C	7.4
470	O=C(c1c2ccc(OC)cc2n(C[C@H]3CCCC[N+]3C)c1C)c4cccc5ccccc54	7.4
471	O=C(c1cc(C[C@H]2CCCC[N+]2C)c(s1)C(C)C)c3cccc4ccccc43	7.4
472	O=C(c1cc(nn2cccc12)C[C@H]3CCCC[N+]3C)c4cccc5ccccc54	7.4
473	O=C(c1c2ccc(OC)cc2n(n1)C[C@H]3CCCC[N+]3C)c4cccc5ccccc54	7.4
474	Clc1c(c2cc(Cl)ccc2n1C[C@H]3CCCC[N+]3C)C(=O)c4cccc5ccccc54	7.4
475	O=C(c1cn(n2n1scco2)C[C@H]3CCCC[N+]3C)c4cccc5ccccc54	7.4
476	OCc1c(C[C@H]2CCCC[N+]2C)cc(s1)C(=O)c3cccc4ccccc43	7.4
477	O=C(c1cn(C[C@H]2CCCC[N+]2C)c3c1cc(cc3C)C)c4cccc5ccccc54	7.4
478	Fc1cccc2c(c(n(C[C@H]3CCCC[N+]3C)c12)C)C(=O)c4cccc5ccccc54	7.4
479	O=C(c1cc2CCCc2c(C[C@H]3CCCC[N+]3C)c1)c4cccc5ccccc54	7.4
480	O=C(c1cn(n2-c(s1)cscn2)C[C@H]3CCCC[N+]3C)c4cccc5ccccc54	7.4
481	Oc1c(OC)cc(cc1C[C@H]2CCCC[N+]2C)C(=O)c3cccc4ccccc43	7.4
482	O=C(c1cn(C[C@H]2CCCC[N+]2C)c3c1cccc3C(C)C)c4cccc5ccccc54	7.3
483	OCc1cc(cc(C[C@H]2CCCC[N+]2C)c1)C(=O)c3cccc4ccccc43	7.3

484	O=C(c1cn(C[C@H]2CCCC[N+]2C)c3c(OC)cccc13)c4cccc5ccccc54	7.3
485	Clc1c(c2cccc(Cl)c2n1C[C@H]3CCCC[N+]3C)C(=O)c4cccc5ccccc54	7.3
486	Clc1c(c2cccc(F)c2n1C[C@H]3CCCC[N+]3C)C(=O)c4cccc5ccccc54	7.3
487	O=C(c1cn(C[C@H]2CCCC[N+]2C)c3ccnn31)c4cccc5ccccc54	7.3
488	O=C(c1cc(C[C@H]2CCCC[N+]2C)c(s1)C[N+])c3cccc4ccccc43	7.2
489	O=C(c1c2ccc3ccccc3c2n(C[C@H]4CCCC[N+]4C)c1)c5cccc6ccccc65	7.2
490	O=C(c1c(n(C[C@H]2CCCC[N+]2C)c3c(cccc13)C)C)c4cccc5ccccc54	7.2
491	Clc1c(c2cccc(c2n1C[C@H]3CCCC[N+]3C)C)C(=O)c4cccc5ccccc54	7.1
492	O=C(c1c(N)c(OC)cc(C[C@H]2CCCC[N+]2C)c1)c3cccc4ccccc43	7.1
493	O=C(c1c(c(c(s1)C[C@H]2CCCC[N+]2C)C(=O)N)C)c3cccc4ccccc43	7.1
494	O=C(c1cc(C[C@H]2CCCC[N+]2C)c(s1)CCOC)c3cccc4ccccc43	7.1
495	O=C(c1c(n(C[C@H]2CCCC[N+]2C)c3c(CC)cccc13)C)c4cccc5ccccc54	7.1
496	OCCc1c(C[C@H]2CCCC[N+]2C)cc(s1)C(=O)c3cccc4ccccc43	7
497	Clc1c(c2cccc(c2n1C[C@H]3CCCC[N+]3C)CC)C(=O)c4cccc5ccccc54	7
498	O=C(c1cn(C[C@H]2CCCC[N+]2C)c3c(OCC)cccc13)c4cccc5ccccc54	7
499	O=C1c2csnc2N(C[C@H]3CCCC[N+]3C)C=C1C(=O)c4cccc5ccccc54	7
500	Clc1c(c2cccc(OC)c2n1C[C@H]3CCCC[N+]3C)C(=O)c4cccc5ccccc54	7

Table S7. List, SMILE and predicted pK_i values for Series 3 in CB_1 receptor.

N°	SMILES	Pred pK_i
1	O=S(=O)(c1c2ccccc2n(C[C@H]3CCCC[N+]3C)c1)c4c[nH]c5ccccc54	8.9
2	O=C(c1c2ccccc2n(C[C@H]3CCCC[N+]3C)c1)c4cc(nc5ccc(cc45)C)C	8.8
3	O=C(N[C@@H]1c2ccccc2C[C@H]1C)c3cn(C[C@H]4CCCC[N+]4C)c5ccccc53	8.8
4	O=C(c1c2ccccc2n(C[C@H]3CCCC[N+]3C)c1)c4c5cc(cc(c5nc(c4)C)C)C	8.8
5	O=C(c1cn(C[C@H]2CCCC[N+]2C)c3ccccc31)c4c5ccccc5ccc4C	8.6
6	O=C(NCC(CC)CC)c1c2ccccc2n(C[C@H]3CCCC[N+]3C)c1	8.6
7	O=C(c1c2ccccc2n(C[C@H]3CCCC[N+]3C)c1)c4cc(nc5c(C)cccc45)C	8.6
8	O=C([C@@H]1c2ccccc2CCC1)c3cn(C[C@H]4CCCC[N+]4C)c5ccccc53	8.5
9	O=C(c1cn(C[C@H]2CCCC[N+]2C)c3ccccc31)c4c5ccccc5nc6CCCc46	8.5
10	Clc1ccc2c(C(C(=O)c3c4ccccc4n(C[C@H]5CCCC[N+]5C)c3)=CC(=O)N2)c1	8.5
11	O=S(=O)(c1cn(C[C@H]2CCCC[N+]2C)c3ccccc31)c4csc5ccccc54	8.5
12	O=C(c1cn(C[C@H]2CCCC[N+]2C)c3ccccc31)c4ccnc5ccccc54	8.5
13	O=C(CN1c2ccccc2C[C@@H]1C)c3cn(C[C@H]4CCCC[N+]4C)c5ccccc53	8.4
14	O=S(=O)(c1cn(C[C@H]2CCCC[N+]2C)c3ccccc31)c4cn(c5ccccc54)C	8.4
15	O=C([C@@H]1c2ccsc2CCS1)c3c4ccccc4n(C[C@H]5CCCC[N+]5C)c3	8.4
16	O=C(n1c(c(c2ccccc21)C)C)c3cn(C[C@H]4CCCC[N+]4C)c5ccccc53	8.4
17	O=C(C1CCCCCC1)c2c3ccccc3n(C[C@H]4CCCC[N+]4C)c2	8.4
18	O=C(c1c2ccccc2n(C[C@H]3CCCC[N+]3C)c1)c4ccnc5ccc(OC)cc54	8.4
19	O=S1(=O)c2ccccc2N(CC1)C(=O)c3cn(C[C@H]4CCCC[N+]4C)c5ccccc53	8.4
20	O=C(c1cn(C[C@H]2CCCC[N+]2C)c3ccccc31)c4c5ccccc5cc6ccccc64	8.4
21	Clc1cccc(c1NC(=O)c2c3ccccc3n(C[C@H]4CCCC[N+]4C)c2)C(F)(F)F	8.4

22	O=S(=O)(c1cn(C[C@H]2CCCC[N+]2C)c3ccccc31)c4ccccc4S(=O)(=O)C	8.3
23	O=C(n1c(nc2ccccc21)C)c3cn(C[C@H]4CCCC[N+]4C)c5ccccc53	8.3
24	O=C(N[C@@H]1CCCC[C@H]1CC)c2cn(C[C@H]3CCCC[N+]3C)c4ccccc42	8.3
25	Brc1cc(c(c(c1C)C)C)C(=O)c2c3ccccc3n(C[C@H]4CCCC[N+]4C)c2	8.3
26	O=[S@](c1c2ccccc2n(C[C@H]3CCCC[N+]3C)c1)c4cccc(N)c4C#N	8.3
27	Fc1ccc2c(c(C(=O)c3c4ccccc4n(C[C@H]5CCCC[N+]5C)c3)cc(n2)C)c1	8.3
28	O=S(=O)(c1c2ccccc2n(C[C@H]3CCCC[N+]3C)c1)c4c[nH]c5cc(N)ccc54	8.3
29	O=C(N[C@@H]1CCCC[C@H]1SCC)c2c3ccccc3n(C[C@H]4CCCC[N+]4C)c2	8.2
30	O=C(c1c2ccccc2n(C[C@H]3CCCC[N+]3C)c1)c4ccc(c(N)c4)C	8.2
31	O=C(C1=C(c2ccccc2C1)C)c3c4ccccc4n(C[C@H]5CCCC[N+]5C)c3	8.2
32	O=C(c1cn(C[C@H]2CCCC[N+]2C)c3ccccc31)c4cc(nc5ccccc54)N	8.2
33	O=S(=O)(N1CCC[C@H]2CCCC[C@@H]21)c3c4ccccc4n(C[C@H]5CCCC[N+]5C)c3	8.2
34	O=C1C=C(c2ccccc2N1C)C(=O)c3cn(C[C@H]4CCCC[N+]4C)c5ccccc53	8.2
35	O=C(Nc1ccccc1OC)c2cn(C[C@H]3CCCC[N+]3C)c4ccccc24	8.1
36	O=C(c1c2ccccc2n(C[C@H]3CCCC[N+]3C)c1)c4ccnc(NCC)c4	8.1
37	O=C(N[C@@H]1CCCC[C@H]1C(C)C)c2c3ccccc3n(C[C@H]4CCCC[N+]4C)c2	8.1
38	O=C(n1c2ccccc2c3CCCCc31)c4cn(C[C@H]5CCCC[N+]5C)c6ccccc64	8.1
39	O=S(=O)(c1c2ccccc2n(C[C@H]3CCCC[N+]3C)c1)c4c(C)ccc(c4)C	8.1
40	O=C(c1c2ccccc2n(C[C@H]3CCCC[N+]3C)c1)c4cc5ccccc5c6ccccc64	8.1
41	Clc1c(c(Cl)ccc1S(=O)(=O)c2c3ccccc3n(C[C@H]4CCCC[N+]4C)c2)C	8.1
42	O=S(=O)(c1c2ccccc2n(C[C@H]3CCCC[N+]3C)c1)c4cccc5cccnc54	8.1
43	O=C(N1c2ccccc2N(CC1)CC)c3cn(C[C@H]4CCCC[N+]4C)c5ccccc53	8.1
44	O=C(c1cccc2c1CCCN2)c3c4ccccc4n(C[C@H]5CCCC[N+]5C)c3	8.1
45	O=C(N[C@@H]1C[C@@H](CC[C@H]1C(C)C)C)c2c3ccccc3n(C[C@H]4CCCC[N+]4C)c2	8.1
46	Clc1cc(c2ccccc2n1)C(=O)c3cn(C[C@H]4CCCC[N+]4C)c5ccccc53	8.1
47	Fc1ccc2c(N(CCN2C)C(=O)c3c4ccccc4n(C[C@H]5CCCC[N+]5C)c3)c1	8.1
48	O=C(c1cn(C[C@H]2CCCC[N+]2C)c3ccccc31)c4cc(O)nc5ccccc54	8.1
49	O=C(N1c2ccccc2S[C@H](C1)C)c3cn(C[C@H]4CCCC[N+]4C)c5ccccc53	8
50	Clc1cccc(c1NC(=O)c2c3ccccc3n(C[C@H]4CCCC[N+]4C)c2)C	8
51	O=C(c1cn(C[C@H]2CCCC[N+]2C)c3ccccc31)c4cc(cc5ccccc54)C#N	8
52	O=S(=O)(c1c2ccccc2n(C[C@H]3CCCC[N+]3C)c1)c4cccc5ccc(nc54)C	8
53	O=C([C@H]1c2ccccc2OCC1)c3c4ccccc4n(C[C@H]5CCCC[N+]5C)c3	8
54	O=C(c1cn(C[C@H]2CCCC[N+]2C)c3ccccc31)c4ccc(c5ccccc54)C	8
55	O=C(c1cn(C[C@H]2CCCC[N+]2C)c3ccccc31)c4cnnc5ccccc54	8
56	Clc1cccc(F)c1NC(=O)c2c3ccccc3n(C[C@H]4CCCC[N+]4C)c2	8
57	O=C(c1c2ccccc2n(C[C@H]3CCCC[N+]3C)c1)c4c5cccc(N)c5c(C)cn4	8
58	Fc1cccc(F)c1NC(=O)c2c3ccccc3n(C[C@H]4CCCC[N+]4C)c2	8
59	Brc1cc(O)c(O)cc1C(=O)c2cn(C[C@H]3CCCC[N+]3C)c4ccccc42	8
60	O[C@H](c1cn(C[C@H]2CCCC[N+]2C)c3ccccc31)c4cccc5ccccc54	8
61	O=S(=O)(c1cn(C[C@H]2CCCC[N+]2C)c3ccccc31)Cc4ccccc4C	8
62	O=C(c1cn(C[C@H]2CCCC[N+]2C)c3ccccc31)C4=CC(=O)Nc5ccccc54	8
63	Clc1ccc(c2ccccc12)C(=O)c3cn(C[C@H]4CCCC[N+]4C)c5ccccc53	8

64	Clc1ccc(Cl)c(C(=O)c2c3ccccc3n(C[C@H]4CCCC[N+]4C)c2)c1Cl	7.9
65	O=S(=O)(c1c2ccccc2n(C[C@H]3CCCC[N+]3C)c1)c4ccccc4CC	7.9
66	Clc1cccc(S(=O)(=O)c2c3ccccc3n(C[C@H]4CCCC[N+]4C)c2)c1F	7.9
67	O=S(=O)(c1c2ccccc2n(C[C@H]3CCCC[N+]3C)c1)c4ccccc4	7.9
68	Clc1ccc(C(=O)c2c3ccccc3n(C[C@H]4CCCC[N+]4C)c2)cc1C	7.9
69	O=C(c1cn(C[C@H]2CCCC[N+]2C)c3ccccc31)Cc4ccccc4C	7.9
70	O=C(N1c2cc(ccc2OCC1)C)c3c4ccccc4n(C[C@H]5CCCC[N+]5C)c3	7.9
71	O=C(c1c2ccccc2n(C[C@H]3CCCC[N+]3C)c1)c4cccc(OC)c4N	7.9
72	O=C(c1cn(C[C@H]2CCCC[N+]2C)c3ccccc31)c4cccc(OC)c4OC	7.9
73	O=C(Nc1ccccc1CC)c2cn(C[C@H]3CCCC[N+]3C)c4ccccc42	7.9
74	O=C(c1c2ccccc2n(C[C@H]3CCCC[N+]3C)c1)c4cccc(N)c4C	7.9
75	Fc1ccc(S(=O)(=O)C)c(C(=O)c2c3ccccc3n(C[C@H]4CCCC[N+]4C)c2)c1	7.9
76	O=C([C@@H]1CSCCS1)c2c3ccccc3n(C[C@H]4CCCC[N+]4C)c2	7.9
77	O=C(N1c2ccccc2CC[C@H]1C)c3cn(C[C@H]4CCCC[N+]4C)c5ccccc53	7.9
78	O=C(c1cn(C[C@H]2CCCC[N+]2C)c3ccccc31)c4cc(CC)ccc4CC	7.9
79	O=C(N1c2ccccc2[C@@H](CC1)C)c3cn(C[C@H]4CCCC[N+]4C)c5ccccc53	7.9
80	Clc1cccc(F)c1CS(=O)(=O)c2c3ccccc3n(C[C@H]4CCCC[N+]4C)c2	7.9
81	O=S(=O)(c1c2ccccc2n(C[C@H]3CCCC[N+]3C)c1)Cc4cccc(OC)c4	7.9
82	O=C(C1CCCCCCC1)c2cn(C[C@H]3CCCC[N+]3C)c4ccccc42	7.9
83	Clc1ccc2c(N(C[C@@H](O2)C)C(=O)c3c4ccccc4n(C[C@H]5CCCC[N+]5C)c3)c1	7.9
84	Brc1cccc([C@H](O)c2c3ccccc3n(C[C@H]4CCCC[N+]4C)c2)c1	7.9
85	O=C(c1c2ccccc2n(C[C@H]3CCCC[N+]3C)c1)c4c5cccc(N)c5ccn4	7.9
86	O=C(c1cn(C[C@H]2CCCC[N+]2C)c3ccccc31)[C@H]4c5ccccc5CCS4	7.9
87	O=C(Nc1ccccc1SC)c2cn(C[C@H]3CCCC[N+]3C)c4ccccc24	7.9
88	Fc1ccc(c(NC(=O)c2c3ccccc3n(C[C@H]4CCCC[N+]4C)c2)c1)C	7.9
89	Clc1cc(C(=O)c2c3ccccc3n(C[C@H]4CCCC[N+]4C)c2)cc(NC)n1	7.9
90	Clc1ccc(Cl)c(S(=O)(=O)c2c3ccccc3n(C[C@H]4CCCC[N+]4C)c2)c1	7.9
91	O=C(c1c(ccc(C(C)(C)C)c1)C)c2c3ccccc3n(C[C@H]4CCCC[N+]4C)c2	7.8
92	O=C(c1c2ccccc2n(C[C@H]3CCCC[N+]3C)c1)c4ccc5CCc6cccc4c65	7.8
93	Clc1cccc(Cl)c1OCC(=O)c2c3ccccc3n(C[C@H]4CCCC[N+]4C)c2	7.8
94	O=C(N1CCC[C@H](C1)CC)c2c3ccccc3n(C[C@H]4CCCC[N+]4C)c2	7.8
95	O=C(C1CCSCC1)c2c3ccccc3n(C[C@H]4CCCC[N+]4C)c2	7.8
96	Brc1ccc(c(C(=O)c2c3ccccc3n(C[C@H]4CCCC[N+]4C)c2)c1)C	7.8
97	O=C(Nc1ccccc1C(C)C)c2cn(C[C@H]3CCCC[N+]3C)c4ccccc42	7.8
98	O=S(=O)(c1cn(C[C@H]2CCCC[N+]2C)c3ccccc31)c4cccc5ccccc54	7.8
99	Fc1ccc2c(nc(cc2C(=O)c3c4ccccc4n(C[C@H]5CCCC[N+]5C)c3)C)c1	7.8
100	O=C(N1c2cc(cc(c2CCC1)C)C)c3c4ccccc4n(C[C@H]5CCCC[N+]5C)c3	7.8
101	O=C(c1c2ccccc2n(C[C@H]3CCCC[N+]3C)c1)c4cccc(SCC#N)c4	7.8
102	Clc1cccc(S(=O)(=O)c2c3ccccc3n(C[C@H]4CCCC[N+]4C)c2)c1C	7.8
103	O=C(c1c2ccccc2n(C[C@H]3CCCC[N+]3C)c1)c4c5CCC(Cc5c(s4)C)(C)C	7.8
104	Clc1c(cc(O)c(C(=O)c2c3ccccc3n(C[C@H]4CCCC[N+]4C)c2)c1C)C	7.8
105	O=C(c1cn(C[C@H]2CCCC[N+]2C)c3ccccc31)C4=C/C(Nc5ccccc54)=N/N	7.8

106	O=C(N1c2ccccc2O[C@H](C1)C)c3cn(C[C@H]4CCCC[N+]4C)c5ccccc53	7.8
107	O=[S@@](Cc1cccc(c1)C(=O)c2c3ccccc3n(C[C@H]4CCCC[N+]4C)c2)C	7.8
108	O=C(c1c2ccccc2n(C[C@H]3CCCC[N+]3C)c1)c4cccc5c4cccn5	7.8
109	O=C(c1cn(C[C@H]2CCCC[N+]2C)c3ccccc31)c4cc(OC)nc5ccccc54	7.8
110	O=C(c1c2ccccc2n(C[C@H]3CCCC[N+]3C)c1)c4c(C)ccc(c4)C	7.8
111	Brc1c(nn(c1C(=O)c2c3ccccc3n(C[C@H]4CCCC[N+]4C)c2)C)C	7.8
112	O=C(N1c2cc(OC)ccc2OCC1)c3c4ccccc4n(C[C@H]5CCCC[N+]5C)c3	7.8
113	O=C(N1c2ccc(OC)cc2CCC1)c3c4ccccc4n(C[C@H]5CCCC[N+]5C)c3	7.8
114	Clc1ccc(SCC)c(C(=O)c2c3ccccc3n(C[C@H]4CCCC[N+]4C)c2)c1	7.8
115	Clc1cc(Cl)cc(C(=O)c2c3ccccc3n(C[C@H]4CCCC[N+]4C)c2)c1OC	7.8
116	O[C@@H](c1ccccc1NC(=O)c2cn(C[C@H]3CCCC[N+]3C)c4ccccc42)C	7.8
117	O=C(c1c2ccccc2n(C[C@H]3CCCC[N+]3C)c1)/C=C/C(C)(C)C	7.8
118	O=C(c1cn(C[C@H]2CCCC[N+]2C)c3ccccc31)c4cc(nc5ccccc54)C	7.8
119	Clc1cccc(Cl)c1NC(=O)c2c3ccccc3n(C[C@H]4CCCC[N+]4C)c2	7.8
120	O=C(c1c2ccccc2n(C[C@H]3CCCC[N+]3C)c1)c4ccc(OC)cc4C	7.8
121	Clc1cc(C(=O)c2c3ccccc3n(C[C@H]4CCCC[N+]4C)c2)ccc1OC	7.8
122	O=C(c1ccc(c(O)c1C)C)c2c3ccccc3n(C[C@H]4CCCC[N+]4C)c2	7.8
123	Fc1ccccc1/C=C(/C(=O)c2c3ccccc3n(C[C@H]4CCCC[N+]4C)c2)C	7.8
124	O=C(c1c2ccccc2n(C[C@H]3CCCC[N+]3C)c1)c4ccncc4C	7.8
125	Clc1c(F)c(C(=O)c2c3ccccc3n(C[C@H]4CCCC[N+]4C)c2)ccn1	7.8
126	O[C@@H](c1c2ccccc2n(C[C@H]3CCCC[N+]3C)c1)c4cccc(c4)C	7.8
127	O=S(=O)(Nc1ccccc1C)c2cn(C[C@H]3CCCC[N+]3C)c4ccccc42	7.8
128	Clc1cc(c(cc1C(=O)c2cn(C[C@H]3CCCC[N+]3C)c4ccccc42)C)C	7.7
129	O=C(c1cn(C[C@H]2CCCC[N+]2C)c3ccccc31)c4c(OC)ccc5ccccc54	7.7
130	O=C(Oc1ccccc1C(C)(C)C)c2c3ccccc3n(C[C@H]4CCCC[N+]4C)c2	7.7
131	C[N+]1CCCC[C@@H]1Cn2cc(Nc3cccc4cccnc43)c5ccccc52	7.7
132	O=C(c1c2ccccc2n(C[C@H]3CCCC[N+]3C)c1)c4cccc(c4N)C	7.7
133	O=C(c1cc(n(c1C)C)C)c2c3ccccc3n(C[C@H]4CCCC[N+]4C)c2	7.7
134	O=C(N1CCS[C@@H](C1)CC)c2c3ccccc3n(C[C@H]4CCCC[N+]4C)c2	7.7
135	O=C(c1c2ccccc2n(C[C@H]3CCCC[N+]3C)c1)c4cccc(c4)CC#N	7.7
136	Clc1cc(F)ccc1S(=O)(=O)c2c3ccccc3n(C[C@H]4CCCC[N+]4C)c2	7.7
137	O=S(=O)(c1ccccc1C(=O)c2c3ccccc3n(C[C@H]4CCCC[N+]4C)c2)C	7.7
138	O=C(N1c2cnccc2OCC1)c3c4ccccc4n(C[C@H]5CCCC[N+]5C)c3	7.7
139	FC(Sc1ccccc1C(=O)c2c3ccccc3n(C[C@H]4CCCC[N+]4C)c2)F	7.7
140	O=S(=O)(c1c2ccccc2n(C[C@H]3CCCC[N+]3C)c1)c4cccc5cc(cnc54)C	7.7
141	O=C(c1c2ccccc2n(C[C@H]3CCCC[N+]3C)c1)c4cc(ccc4C(C)C)C	7.7
142	O=C(N1[C@@H](CCC[C@H](C1)C)C)c2c3ccccc3n(C[C@H]4CCCC[N+]4C)c2	7.7
143	Brc1c(C)ccc(C(=O)c2c3ccccc3n(C[C@H]4CCCC[N+]4C)c2)c1	7.7
144	O=C(c1c2ccccc2n(C[C@H]3CCCC[N+]3C)c1)[C@@H]4COc5ccccc5O4	7.7
145	Fc1ccccc1S(=O)(=O)c2c3ccccc3n(C[C@H]4CCCC[N+]4C)c2	7.7
146	O=C(C[C@@H]1c2ccccc2CCO1)c3cn(C[C@H]4CCCC[N+]4C)c5ccccc53	7.7
147	Brc1cc(C(=O)c2c3ccccc3n(C[C@H]4CCCC[N+]4C)c2)cs1	7.7

148	O=C(c1c2ccccc2n(C[C@H]3CCCC[N+]3C)c1)c4c5c(N)cccc5ccn4	7.7
149	O=C(c1c([nH]c2ccc(cc21)C)C)c3c4ccccc4n(C[C@H]5CCCC[N+]5C)c3	7.7
150	Clc1cccc(C(=O)c2c3ccccc3n(C[C@H]4CCCC[N+]4C)c2)c1N	7.7
151	O=C(C1CCCCC1)c2c3ccccc3n(C[C@H]4CCCC[N+]4C)c2	7.7
152	C[N+]1CCCC[C@@H]1Cn2cc(O[C@H]3c4ccccc4CCC3)c5ccccc52	7.7
153	O=C(c1cn(C[C@H]2CCCC[N+]2C)c3ccccc31)c4c(oc5ccccc54)CC	7.7
154	O=C(N1c2ccccc2C[C@@H](C1)C)c3cn(C[C@H]4CCCC[N+]4C)c5ccccc53	7.7
155	Brc1ccc(Cl)cc1C(=O)c2c3ccccc3n(C[C@H]4CCCC[N+]4C)c2	7.6
156	O=S(=O)(c1cc(N)cc(c1)C(=O)c2c3ccccc3n(C[C@H]4CCCC[N+]4C)c2)C	7.6
157	Clc1ccc(C(=O)c2c3ccccc3n(C[C@H]4CCCC[N+]4C)c2)c(C)c1	7.6
158	Brc1ccc(cc1C(=O)c2c3ccccc3n(C[C@H]4CCCC[N+]4C)c2)C	7.6
159	O=C(c1cn(C[C@H]2CCCC[N+]2C)c3ccccc31)c4c(snn4)-c5ccccc5	7.6
160	Fc1cc(C)ccc1C(=O)c2c3ccccc3n(C[C@H]4CCCC[N+]4C)c2	7.6
161	O=C(c1cn(C[C@H]2CCCC[N+]2C)c3ccccc31)c4cncc5ccccc54	7.6
162	O=C(c1c2c(nn1C)CCC2)c3c4ccccc4n(C[C@H]5CCCC[N+]5C)c3	7.6
163	O=C(c1c2ccccc2n(C[C@H]3CCCC[N+]3C)c1)c4cccc(OC)c4	7.6
164	O=C(c1c2ccccc2n(C[C@H]3CCCC[N+]3C)c1)c4csc(c4C)C	7.6
165	O=C(c1cn(C[C@H]2CCCC[N+]2C)c3ccccc31)c4c5ccccc5ccn4	7.6
166	Clc1cc(N)c2ccnc(c2c1)C(=O)c3c4ccccc4n(C[C@H]5CCCC[N+]5C)c3	7.6
167	FC(F)Oc1cccc(C(=O)c2c3ccccc3n(C[C@H]4CCCC[N+]4C)c2)c1	7.6
168	O=C(c1c2ccccc2n(C[C@H]3CCCC[N+]3C)c1)c4c5ccc(N)cc5ccn4	7.6
169	Sc1ccccc1C(=O)c2c3ccccc3n(C[C@H]4CCCC[N+]4C)c2	7.6
170	Clc1cccc(C(=O)c2c3ccccc3n(C[C@H]4CCCC[N+]4C)c2)c1F	7.6
171	Clc1ccccc1S(=O)(=O)c2c3ccccc3n(C[C@H]4CCCC[N+]4C)c2	7.6
172	Fc1ccc(c2ccccc12)C(=O)c3cn(C[C@H]4CCCC[N+]4C)c5ccccc53	7.6
173	O=C(N1c2ccccc2N(CC1)C)c3cn(C[C@H]4CCCC[N+]4C)c5ccccc53	7.6
174	O=C(c1csc2CCCCCc12)c3c4ccccc4n(C[C@H]5CCCC[N+]5C)c3	7.6
175	C[N+]1CCCC[C@@H]1Cn2cc(c3ccccc32)C(c4cccc5ccccc54)=C	7.6
176	FC(F)(F)c1ccccc1S(=O)(=O)c2c3ccccc3n(C[C@H]4CCCC[N+]4C)c2	7.6
177	O=C(c1c2ccccc2n(C[C@H]3CCCC[N+]3C)c1)c4ccccc4C#N	7.6
178	O=C(c1c2ccccc2n(C[C@H]3CCCC[N+]3C)c1)c4cccc(C[N+](C)C)c4	7.6
179	Clc1cccc(C(=O)c2c3ccccc3n(C[C@H]4CCCC[N+]4C)c2)c1C	7.6
180	O=C(O[C@@H](C(C)C)C)c1c2ccccc2n(C[C@H]3CCCC[N+]3C)c1	7.6
181	FC(F)(F)c1ccccc1C(=O)c2c3ccccc3n(C[C@H]4CCCC[N+]4C)c2	7.6
182	Fc1ccc2c(N(CCC2)C(=O)c3c4ccccc4n(C[C@H]5CCCC[N+]5C)c3)c1	7.6
183	O=C(c1c2ccccc2n(C[C@H]3CCCC[N+]3C)c1)c4cccc(c4)C(C)(C)C#N	7.6
184	Brc1cccc(C(=O)c2c3ccccc3n(C[C@H]4CCCC[N+]4C)c2)c1N	7.6
185	O=C(c1c2ccccc2n(C[C@H]3CCCC[N+]3C)c1)c4cccc5CCOc45	7.6
186	O=C(c1c2ccccc2n(C[C@H]3CCCC[N+]3C)c1)c4cncnc4C(C)C	7.6
187	O=C(c1c2ccccc2n(C[C@H]3CCCC[N+]3C)c1)c4c5cccc(N)c5cc(n4)C	7.6
188	N=C(c1c2ccccc2n(C[C@H]3CCCC[N+]3C)c1)c4ccccc4	7.6
189	OC1(CCCCC1)C(=O)c2c3ccccc3n(C[C@H]4CCCC[N+]4C)c2	7.6

190	Brc1cncc(C(=O)c2c3ccccc3n(C[C@H]4CCCC[N+]4C)c2)c1	7.6
191	Brc1ccc(C(=O)c2c3ccccc3n(C[C@H]4CCCC[N+]4C)c2)c(C)c1	7.6
192	O=C([C@H]1CCCC[C@@H]1C)c2c3ccccc3n(C[C@H]4CCCC[N+]4C)c2	7.6
193	Clc1c(ccc(F)c1C(=O)c2c3ccccc3n(C[C@H]4CCCC[N+]4C)c2)C	7.5
194	Brc1ccc(OC)cc1C(=O)c2c3ccccc3n(C[C@H]4CCCC[N+]4C)c2	7.5
195	O=C([C@H]1CCC[C@H]([N+])[C@@H]1C)c2c3ccccc3n(C[C@H]4CCCC[N+]4C)c2	7.5
196	O=C(c1c2ccccc2n(C[C@H]3CCCC[N+]3C)c1)c4cccc(C(C)(C)C)c4	7.5
197	O=C(Oc1ccccc1C)c2c3ccccc3n(C[C@H]4CCCC[N+]4C)c2	7.5
198	Clc1c(cc(cc1C(=O)c2c3ccccc3n(C[C@H]4CCCC[N+]4C)c2)C)C	7.5
199	O=C(c1c2ccccc2n(C[C@H]3CCCC[N+]3C)c1)c4cccnc4SC	7.5
200	Clc1cc(F)c(C(=O)c2c3ccccc3n(C[C@H]4CCCC[N+]4C)c2)cc1	7.5
201	Clc1cc(F)cc(C(=O)c2c3ccccc3n(C[C@H]4CCCC[N+]4C)c2)c1O	7.5
202	O=S(=O)(c1c2ccccc2n(C[C@H]3CCCC[N+]3C)c1)c4ccccc4C(OC)=O	7.5
203	O=C(c1cn(C[C@H]2CCCC[N+]2C)c3ccccc31)c4cccc5ccccc54	7.5
204	C[N+]1CCCC[C@@H]1Cn2cc(Oc3cccc(CC)c3)c4ccccc42	7.5
205	O=S(=O)(c1c2ccccc2n(C[C@H]3CCCC[N+]3C)c1)c4cccc(c4C(OC)=O)C	7.5
206	O=C(c1c2ccccc2n(C[C@H]3CCCC[N+]3C)c1)c4csc(c4CC)C	7.5
207	Fc1cc(C(=O)c2c3ccccc3n(C[C@H]4CCCC[N+]4C)c2)cc(c1)C	7.5
208	Clc1cccc2c1cccc2S(=O)(=O)c3c4ccccc4n(C[C@H]5CCCC[N+]5C)c3	7.5
209	O=C(c1c(cccc1N)C)c2c3ccccc3n(C[C@H]4CCCC[N+]4C)c2	7.5
210	Clc1cccc2c1cccc2C(=O)c3c4ccccc4n(C[C@H]5CCCC[N+]5C)c3	7.5
211	O=C(c1c2ccccc2n(C[C@H]3CCCC[N+]3C)c1)c4c(SC)nsc4SC	7.5
212	O=C(c1c2ccccc2n(C[C@H]3CCCC[N+]3C)c1)c4c(oc(c4)C)C	7.5
213	O=C(Nc1c(C)csc1)c2c3ccccc3n(C[C@H]4CCCC[N+]4C)c2	7.5
214	Brc1cccc(F)c1C(=O)c2c3ccccc3n(C[C@H]4CCCC[N+]4C)c2	7.5
215	Clc1ccc(C(=O)c2c3ccccc3n(C[C@H]4CCCC[N+]4C)c2)cc1OC	7.5
216	FC(F)Oc1ccccc1C(=O)c2c3ccccc3n(C[C@H]4CCCC[N+]4C)c2	7.5
217	O=C(c1c2ccccc2n(C[C@H]3CCCC[N+]3C)c1)c4cccc(N)c4	7.5
218	O=C(c1c2ccccc2n(C[C@H]3CCCC[N+]3C)c1)c4csc(c4)CC	7.5
219	Clc1cccc([N+]([O-])=O)c1C(=O)c2c3ccccc3n(C[C@H]4CCCC[N+]4C)c2	7.5
220	Clc1ccc(cc1C(=O)c2c3ccccc3n(C[C@H]4CCCC[N+]4C)c2)C	7.5
221	Brc1ccccc1C(=O)c2c3ccccc3n(C[C@H]4CCCC[N+]4C)c2	7.5
222	O=C(c1c2ccccc2n(C[C@H]3CCCC[N+]3C)c1)c4cccc(c4)C	7.5
223	Fc1ccc(cc1S(=O)(=O)c2c3ccccc3n(C[C@H]4CCCC[N+]4C)c2)C	7.5
224	Brc1ccc(F)cc1C(=O)c2c3ccccc3n(C[C@H]4CCCC[N+]4C)c2	7.5
225	O=C(c1c2ccccc2n(C[C@H]3CCCC[N+]3C)c1)c4cccc(O)c4C	7.5
226	O=C(N1c2ccc(cc2CCC1)C)c3c4ccccc4n(C[C@H]5CCCC[N+]5C)c3	7.5
227	O=C(c1c2ccccc2n(C[C@H]3CCCC[N+]3C)c1)c4c5cc(N)ccc5ccn4	7.5
228	O=C(c1cn(C[C@H]2CCCC[N+]2C)c3ccccc31)c4c5ccccc5c(O)nn4	7.5
229	O=C(c1cn(C[C@H]2CCCC[N+]2C)c3ccccc31)c4c(O)ccc5ccccc54	7.5
230	O=C(Oc1ccccc1SC)c2c3ccccc3n(C[C@H]4CCCC[N+]4C)c2	7.5
231	Clc1ccc(N)cc1C(=O)c2c3ccccc3n(C[C@H]4CCCC[N+]4C)c2	7.5

232	O=C(N1c2ccc(N)cc2CCC1)c3c4ccccc4n(C[C@H]5CCCC[N+]5C)c3	7.5
233	O=C(c1cn(C[C@H]2CCCC[N+]2C)c3ccccc31)c4cccc5Cc6ccccc6-c45	7.5
234	O=C(C1=CCCCCC1)c2c3ccccc3n(C[C@H]4CCCC[N+]4C)c2	7.5
235	Clc1cc(c(OC)c(c1)C)C(=O)c2c3ccccc3n(C[C@H]4CCCC[N+]4C)c2	7.5
236	O=C(c1cn(C[C@H]2CCCC[N+]2C)c3ccccc31)[C@H]4c5ccccc5CCO4	7.5
237	O=C(N1c2ccccc2OCC1)c3cn(C[C@H]4CCCC[N+]4C)c5ccccc53	7.5
238	O=C(c1c2ccccc2n(C[C@H]3CCCC[N+]3C)c1)c4cccc(COC)c4	7.5
239	O=C(N1c2ccc[nH+]c2N(CC1)CC)c3c4ccccc4n(C[C@H]5CCCC[N+]5C)c3	7.4
240	Fc1ccc([C@@H](O)C(=O)c2c3ccccc3n(C[C@H]4CCCC[N+]4C)c2)cc1C	7.4
241	Fc1ccc2c(N(CCO2)C(=O)c3c4ccccc4n(C[C@H]5CCCC[N+]5C)c3)c1	7.4
242	O=C(Nc1ccc([nH+]c1C)N)c2c3ccccc3n(C[C@H]4CCCC[N+]4C)c2	7.4
243	O=C([C@@H]1CCC[C@@H](C1)C)c2c3ccccc3n(C[C@H]4CCCC[N+]4C)c2	7.4
244	Fc1cccc2c1N(CCC2)C(=O)c3c4ccccc4n(C[C@H]5CCCC[N+]5C)c3	7.4
245	O=C([C@@H]1C[C@H]2CC[C@@H]1O2)c3c4ccccc4n(C[C@H]5CCCC[N+]5C)c3	7.4
246	Fc1ccccc1C(=O)c2c3ccccc3n(C[C@H]4CCCC[N+]4C)c2	7.4
247	Clc1ccc2c(N(CCO2)C(=O)c3c4ccccc4n(C[C@H]5CCCC[N+]5C)c3)c1	7.4
248	Fc1ccc(F)c(C(=O)c2c3ccccc3n(C[C@H]4CCCC[N+]4C)c2)c1	7.4
249	Oc1ccc(C(=O)c2c3ccccc3n(C[C@H]4CCCC[N+]4C)c2)cc1OC	7.4
250	Oc1c(OC)cccc1C(=O)c2c3ccccc3n(C[C@H]4CCCC[N+]4C)c2	7.4
251	O=C(c1cn(C[C@H]2CCCC[N+]2C)c3ccccc31)c4cc(c(cc4OC)C)C	7.4
252	Clc1cccc(C(=N)c2c3ccccc3n(C[C@H]4CCCC[N+]4C)c2)c1	7.4
253	FC(F)(F)[C@H]1CCC[C@H](C1)C(=O)c2c3ccccc3n(C[C@H]4CCCC[N+]4C)c2	7.4
254	Brc1ccc(OC)c(C(=O)c2c3ccccc3n(C[C@H]4CCCC[N+]4C)c2)c1	7.4
255	O=C(c1c2ccccc2n(C[C@H]3CCCC[N+]3C)c1)c4c5cc(cc(N)c5ccn4)C	7.4
256	Brc1ccc(N)cc1C(=O)c2c3ccccc3n(C[C@H]4CCCC[N+]4C)c2	7.4
257	Brc1ccccc1S(=O)(=O)c2c3ccccc3n(C[C@H]4CCCC[N+]4C)c2	7.4
258	O=C(c1ccc(cc1C)C)c2c3ccccc3n(C[C@H]4CCCC[N+]4C)c2	7.4
259	O=C(c1c2ccccc2n(C[C@H]3CCCC[N+]3C)c1)c4cc(C)ccn4	7.4
260	O=C(NC1CCCCC1)c2c3ccccc3n(C[C@H]4CCCC[N+]4C)c2	7.4
261	Brc1cccc(C(=O)c2c3ccccc3n(C[C@H]4CCCC[N+]4C)c2)c1C	7.4
262	O=C(c1c2ccccc2n(C[C@H]3CCCC[N+]3C)c1)c4csc(c4)C	7.4
263	O=C(c1cn(C[C@H]2CCCC[N+]2C)c3ccccc31)c4ccccc4NC	7.4
264	O=C(c1cn(C[C@H]2CCCC[N+]2C)c3ccccc31)c4c(sc(n4)C)-c5ccccc5	7.4
265	Clc1ccc(N)cc1S(=O)(=O)c2c3ccccc3n(C[C@H]4CCCC[N+]4C)c2	7.4
266	O=C(c1c2ccccc2n(C[C@H]3CCCC[N+]3C)c1)c4ccccc4CC#N	7.4
267	Brc1cc(F)ccc1C(=O)c2c3ccccc3n(C[C@H]4CCCC[N+]4C)c2	7.4
268	O=C(N1c2ccccc2C[C@H]1C)c3cn(C[C@H]4CCCC[N+]4C)c5ccccc53	7.4
269	O=C(c1c2ccccc2n(C[C@H]3CCCC[N+]3C)c1)c4ccccc4SCC	7.4
270	Clc1ccccc1CC(=O)c2c3ccccc3n(C[C@H]4CCCC[N+]4C)c2	7.4
271	Clc1ccc(F)c(C(=O)c2c3ccccc3n(C[C@H]4CCCC[N+]4C)c2)c1	7.4
272	Fc1c(F)cc(F)c(C(=O)c2c3ccccc3n(C[C@H]4CCCC[N+]4C)c2)c1	7.4
273	O=C([C@H]1CCCC[C@@H]1CO)c2c3ccccc3n(C[C@H]4CCCC[N+]4C)c2	7.4

274	O=C(c1c2ccccc2n(C[C@H]3CCCC[N+]3C)c1)c4cc(ccc4SC)C	7.4
275	Fc1ccc(cc1C(=O)c2c3ccccc3n(C[C@H]4CCCC[N+]4C)c2)C(F)(F)F	7.4
276	O=C(c1c2ccccc2n(C[C@H]3CCCC[N+]3C)c1)c4c5ccc(c(N)c5ccn4)C	7.4
277	Oc1cccc2c1N(CCC2)C(=O)c3c4ccccc4n(C[C@H]5CCCC[N+]5C)c3	7.4
278	O=C(c1c2ccccc2n(C[C@H]3CCCC[N+]3C)c1)c4cc(N)ccc4C	7.4
279	Fc1ccc(cc1C(=O)c2c3ccccc3n(C[C@H]4CCCC[N+]4C)c2)C	7.3
280	O=C(N[C@@H]1C=CCCC1)c2c3ccccc3n(C[C@H]4CCCC[N+]4C)c2	7.3
281	Clc1cccc(C(=O)c2c3ccccc3n(C[C@H]4CCCC[N+]4C)c2)c1	7.3
282	Brc1ccc(F)c(C(=O)c2c3ccccc3n(C[C@H]4CCCC[N+]4C)c2)c1	7.3
283	O=C(c1c2ccccc2n(C[C@H]3CCCC[N+]3C)c1)c4ccccc4	7.3
284	Clc1ccc(SC)cc1C(=O)c2c3ccccc3n(C[C@H]4CCCC[N+]4C)c2	7.3
285	O=C(c1cn(C[C@H]2CCCC[N+]2C)c3ccccc31)c4cccc5c4nccn5	7.3
286	O=C(N1c2ccc[nH+]c2N(CC1)C)c3c4ccccc4n(C[C@H]5CCCC[N+]5C)c3	7.3
287	Oc1cc(OC)cc(C(=O)c2c3ccccc3n(C[C@H]4CCCC[N+]4C)c2)c1	7.3
288	FC(F)CN(C1CC1)C(=O)c2c3ccccc3n(C[C@H]4CCCC[N+]4C)c2	7.3
289	O=[S@@](c1ccccc1C(=O)c2c3ccccc3n(C[C@H]4CCCC[N+]4C)c2)CC	7.3
290	Oc1ccc2c(SCO2)c1C(=O)c3c4ccccc4n(C[C@H]5CCCC[N+]5C)c3	7.3
291	Fc1cnccc1C(=O)c2c3ccccc3n(C[C@H]4CCCC[N+]4C)c2	7.3
292	O=C(c1c2ccccc2n(C[C@H]3CCCC[N+]3C)c1)c4cccc(c4C)C	7.3
293	Oc1ccc(C(=O)c2c3ccccc3n(C[C@H]4CCCC[N+]4C)c2)c(c1)C	7.3
294	O=C(c1c2ccccc2n(C[C@H]3CCCC[N+]3C)c1)c4csc(CCC)c4	7.3
295	O=C(N1c2cc(ccc2O[C@@H](C1)C)C)c3c4ccccc4n(C[C@H]5CCCC[N+]5C)c3	7.3
296	Clc1ccc(cc1S(=O)(=O)c2c3ccccc3n(C[C@H]4CCCC[N+]4C)c2)C	7.3
297	O=C(c1c2ccccc2n(C[C@H]3CCCC[N+]3C)c1)c4csc5c4CC[C@H](C5)C	7.3
298	O=S(=O)(c1c2ccccc2n(C[C@H]3CCCC[N+]3C)c1)c4cc[nH]c4	7.3
299	O=C(c1c2ccccc2n(C[C@H]3CCCC[N+]3C)c1)C(=O)c4c[nH]c5ccccc54	7.3
300	O=C(N(CC(C)C)C)c1c2ccccc2n(C[C@H]3CCCC[N+]3C)c1	7.3
301	Fc1cccc(F)c1C(=O)c2c3ccccc3n(C[C@H]4CCCC[N+]4C)c2	7.3
302	Brc1ccsc1S(=O)(=O)c2c3ccccc3n(C[C@H]4CCCC[N+]4C)c2	7.3
303	Clc1ccc(c(C(=O)c2c3ccccc3n(C[C@H]4CCCC[N+]4C)c2)c1)C	7.3
304	O=C(c1c2ccccc2n(C[C@H]3CCCC[N+]3C)c1)c4cc(c(OC)c(c4C)C)C	7.3
305	O=C(c1c2ccccc2n(C[C@H]3CCCC[N+]3C)c1)c4cc(ccc4OC)C	7.3
306	O=C(c1c2ccccc2n(C[C@H]3CCCC[N+]3C)c1)c4cccc5C(=O)c6ccccc6-c54	7.3
307	Oc1c(C(=O)c2c3ccccc3n(C[C@H]4CCCC[N+]4C)c2)c(cc(n1)C)C	7.3
308	Clc1cncc(Cl)c1C(=O)c2c3ccccc3n(C[C@H]4CCCC[N+]4C)c2	7.3
309	O=C([C@H]1c2cccc(c2C[N+]C1)C)c3c4ccccc4n(C[C@H]5CCCC[N+]5C)c3	7.3
310	O=C(c1c2ccccc2n(C[C@H]3CCCC[N+]3C)c1)c4cccc5ccoc54	7.3
311	O=C(c1c2ccccc2n(C[C@H]3CCCC[N+]3C)c1)C4=C(OCCC4)C	7.3
312	Fc1cc(N)c2ccnc(C(=O)c3c4ccccc4n(C[C@H]5CCCC[N+]5C)c3)c2c1	7.3
313	O=C(c1c2ccccc2n(C[C@H]3CCCC[N+]3C)c1)c4ccccc4OC	7.3
314	Fc1c(cccc1C(=O)c2c3ccccc3n(C[C@H]4CCCC[N+]4C)c2)C	7.3
315	O=C(N1CCC[C@H]2CCC[C@H]21)c3c4ccccc4n(C[C@H]5CCCC[N+]5C)c3	7.3

316	O=C(c1c2ccccc2n(C[C@H]3CCCC[N+]3C)c1)/C=C/C(C)C	7.3
317	O=C(c1c2ccccc2n(C[C@H]3CCCC[N+]3C)c1)c4ccccc4C	7.3
318	Brc1cc(F)c(N)cc1C(=O)c2c3ccccc3n(C[C@H]4CCCC[N+]4C)c2	7.3
319	Fc1cc(F)ccc1C(=O)c2c3ccccc3n(C[C@H]4CCCC[N+]4C)c2	7.3
320	Fc1c(C)ccc(C(=O)c2c3ccccc3n(C[C@H]4CCCC[N+]4C)c2)c1	7.2
321	O=S(=O)(N1CCC[C@H]1C(=O)c2c3ccccc3n(C[C@H]4CCCC[N+]4C)c2)C	7.2
322	O=C(N[C@H]1CC=CCC1)c2c3ccccc3n(C[C@H]4CCCC[N+]4C)c2	7.2
323	O=C(NC1CCCCCC1)c2c3ccccc3n(C[C@H]4CCCC[N+]4C)c2	7.2
324	O=C(c1c2ccccc2n(C[C@H]3CCCC[N+]3C)c1)c4cccnc4OCC	7.2
325	O=C(N[C@H](c1cccs1)C)c2c3ccccc3n(C[C@H]4CCCC[N+]4C)c2	7.2
326	Fc1cccc(C(=O)c2cn(C[C@H]3CCCC[N+]3C)c4ccccc42)c1	7.2
327	Clc1cccc(N)c1C(=O)c2c3ccccc3n(C[C@H]4CCCC[N+]4C)c2	7.2
328	O=S(=O)(N)c1ccccc1C(=O)c2c3ccccc3n(C[C@H]4CCCC[N+]4C)c2	7.2
329	O=C(N1CCS[C@H]1CCC)c2c3ccccc3n(C[C@H]4CCCC[N+]4C)c2	7.2
330	O=C(c1cn(C[C@H]2CCCC[N+]2C)c3ccccc31)c4ccccc4N	7.2
331	Cl[C@@H](/C=C/C(=O)c1c2ccccc2n(C[C@H]3CCCC[N+]3C)c1)C	7.2
332	Clc1ccc(NCC)c(C(=O)c2c3ccccc3n(C[C@H]4CCCC[N+]4C)c2)c1	7.2
333	Brc1c(C(=O)c2c3ccccc3n(C[C@H]4CCCC[N+]4C)c2)cccn1	7.2
334	O=C(N(c1ccccn1)C)c2c3ccccc3n(C[C@H]4CCCC[N+]4C)c2	7.2
335	Fc1ccc(F)c(C(=O)c2c3ccccc3n(C[C@H]4CCCC[N+]4C)c2)c1F	7.2
336	O=C([C@H]1[C@@H](CCC[N+]1)C)c2cn(C[C@H]3CCCC[N+]3C)c4ccccc42	7.2
337	O=C(c1c2ccccc2n(C[C@H]3CCCC[N+]3C)c1)c4cnccc4C	7.2
338	O=C(c1c2ccccc2n(C[C@H]3CCCC[N+]3C)c1)c4cccc5cnccc54	7.2
339	O=C(c1c2ccccc2n(C[C@H]3CCCC[N+]3C)c1)c4c(ncc(N)c4)C	7.2
340	Fc1cc(F)cnc1C(=O)c2c3ccccc3n(C[C@H]4CCCC[N+]4C)c2	7.2
341	Fc1cc(F)c(F)c(C(=O)c2c3ccccc3n(C[C@H]4CCCC[N+]4C)c2)c1F	7.2
342	Clc1cnccc1C(=O)c2c3ccccc3n(C[C@H]4CCCC[N+]4C)c2	7.2
343	Fc1ccccc1[C@@H](O)c2c3ccccc3n(C[C@H]4CCCC[N+]4C)c2	7.2
344	Fc1ccc(N)c2c1ccnc2C(=O)c3c4ccccc4n(C[C@H]5CCCC[N+]5C)c3	7.2
345	O=C(c1c2ccccc2n(C[C@H]3CCCC[N+]3C)c1)c4ccccc4C(C)C	7.2
346	O=C(O[C@@H]1CCCC[C@H]1CC)c2cn(C[C@H]3CCCC[N+]3C)c4ccccc42	7.2
347	O=C(c1c2ccccc2n(C[C@H]3CCCC[N+]3C)c1)c4cc(cc(c4OC)C)C	7.2
348	O=C(c1c2ccccc2n(C[C@H]3CCCC[N+]3C)c1)c4ccsc4N	7.2
349	O=C(c1c2ccccc2n(C[C@H]3CCCC[N+]3C)c1)c4ccccc4SCC#N	7.2
350	Clc1cc(Cl)cc(O)c1C(=O)c2c3ccccc3n(C[C@H]4CCCC[N+]4C)c2	7.2
351	Oc1cccc(C(=O)c2c3ccccc3n(C[C@H]4CCCC[N+]4C)c2)c1	7.2
352	O=C(C1(CCCC1)c2cccs2)c3c4ccccc4n(C[C@H]5CCCC[N+]5C)c3	7.2
353	Clc1cc(Cl)c(Cl)c(C(=O)c2c3ccccc3n(C[C@H]4CCCC[N+]4C)c2)c1O	7.2
354	Clc1ccc2c(nccc2c1N)C(=O)c3c4ccccc4n(C[C@H]5CCCC[N+]5C)c3	7.2
355	FC[C@@H]1CCCN1S(=O)(=O)c2c3ccccc3n(C[C@H]4CCCC[N+]4C)c2	7.2
356	Clc1cccc([C@@H](O)c2c3ccccc3n(C[C@H]4CCCC[N+]4C)c2)c1	7.2
357	O=C(c1c(cccc1[N+]([O-])=O)C)c2c3ccccc3n(C[C@H]4CCCC[N+]4C)c2	7.2

358	Clc1ccc(Cl)cc1Oc2c3ccccc3n(C[C@H]4CCCC[N+]4C)c2	7.2
359	O=C(N1CCc2cccc(c21)C)c3c4ccccc4n(C[C@H]5CCCC[N+]5C)c3	7.2
360	O=C(c1c2ccccc2n(C[C@H]3CCCC[N+]3C)c1)C(CC(C)C)(C)C	7.2
361	O=C(c1c2ccccc2n(C[C@H]3CCCC[N+]3C)c1)c4cc(OC)cc(OC)c4	7.2
362	O=C([C@H]1c2ccsc2CCC1)c3c4ccccc4n(C[C@H]5CCCC[N+]5C)c3	7.2
363	O=C(c1c2ccccc2n(C[C@H]3CCCC[N+]3C)c1)c4cccc(SCC#C)c4	7.2
364	C[N+]1CCCC[C@@H]1Cn2cc(c3ccccc32)-c4non-5cc[nH]cc5s4	7.2
365	Fc1ccc(c(C(=O)c2c3ccccc3n(C[C@H]4CCCC[N+]4C)c2)c1)C	7.2
366	FC(F)(F)c1cccc(c1N)C(=O)c2c3ccccc3n(C[C@H]4CCCC[N+]4C)c2	7.2
367	O=C(c1c2ccccc2n(C[C@H]3CCCC[N+]3C)c1)c4cccc(c4)C=C	7.2
368	O=C(c1c2ccccc2n(C[C@H]3CCCC[N+]3C)c1)c4cc(SC)ccc4C	7.2
369	Brc1cccc(C(=O)c2c3ccccc3n(C[C@H]4CCCC[N+]4C)c2)c1	7.2
370	O=C(c1c2ccccc2n(C[C@H]3CCCC[N+]3C)c1)c4cccc(c4)C#C	7.2
371	Clc1ccc2c(CCCN2C(=O)c3c4ccccc4n(C[C@H]5CCCC[N+]5C)c3)c1	7.2
372	Fc1cc(C(=O)c2c3ccccc3n(C[C@H]4CCCC[N+]4C)c2)cc(C(F)(F)F)c1	7.2
373	O=C(CC(CC)CC)c1c2ccccc2n(C[C@H]3CCCC[N+]3C)c1	7.1
374	Fc1c(F)ccc(C(=O)c2c3ccccc3n(C[C@H]4CCCC[N+]4C)c2)c1F	7.1
375	O=C(c1cn(C[C@H]2CCCC[N+]2C)c3ccccc31)c4c(-c5ccccc5)ccs4	7.1
376	O=C(N1c2ccccc2C[C@@H]([N+])C1)c3cn(C[C@H]4CCCC[N+]4C)c5ccccc53	7.1
377	O=C(c1c2ccccc2n(C[C@H]3CCCC[N+]3C)c1)c4ccccc4CO	7.1
378	O=C(c1c2ccccc2n(C[C@H]3CCCC[N+]3C)c1)c4cc(cc(c4O)C)C	7.1
379	O=C(c1cn(C[C@H]2CCCC[N+]2C)c3ccccc31)c4ccccc4-c5ncc[nH]5	7.1
380	Clc1cc(C)ccc1C(=O)c2c3ccccc3n(C[C@H]4CCCC[N+]4C)c2	7.1
381	S=C(N)c1ccccc1NC(=O)c2cn(C[C@H]3CCCC[N+]3C)c4ccccc42	7.1
382	O=C(c1c2ccccc2n(C[C@H]3CCCC[N+]3C)c1)c4cccnc4SC(C)C	7.1
383	Fc1cccc(C2(CC2)C(=O)c3c4ccccc4n(C[C@H]5CCCC[N+]5C)c3)c1	7.1
384	O=S(=O)(C[C@H]1CCCO1)c2c3ccccc3n(C[C@H]4CCCC[N+]4C)c2	7.1
385	Clc1ccccc1C(=O)c2c3ccccc3n(C[C@H]4CCCC[N+]4C)c2	7.1
386	O=C([C@@H](CC1CCCC1)C)c2c3ccccc3n(C[C@H]4CCCC[N+]4C)c2	7.1
387	Oc1cc(O)cc(C(=O)c2c3ccccc3n(C[C@H]4CCCC[N+]4C)c2)c1	7.1
388	O=C(c1c2ccccc2n(C[C@H]3CCCC[N+]3C)c1)c4cccc(OC(C)C)c4	7.1
389	Fc1c(F)cccc1C(=O)c2cn(C[C@H]3CCCC[N+]3C)c4ccccc42	7.1
390	O=C(c1c2ccccc2n(C[C@H]3CCCC[N+]3C)c1)c4cccc(OCC)c4	7.1
391	O=C(C[C@H](c1ccccc1)C)c2c3ccccc3n(C[C@H]4CCCC[N+]4C)c2	7.1
392	Fc1ccc(N)c(C(=O)c2c3ccccc3n(C[C@H]4CCCC[N+]4C)c2)c1	7.1
393	O=C(c1c2ccccc2n(C[C@H]3CCCC[N+]3C)c1)c4cccc(CC[N+])c4	7.1
394	O=C(c1c2ccccc2n(C[C@H]3CCCC[N+]3C)c1)/C(=N/OC)c4ccccc4	7.1
395	O=C(c1c2ccccc2n(C[C@H]3CCCC[N+]3C)c1)c4cccc(c4O)C	7.1
396	Clc1cccc(c1C(=O)c2c3ccccc3n(C[C@H]4CCCC[N+]4C)c2)C	7.1
397	Clc1ccccc1[C@@H](O)c2c3ccccc3n(C[C@H]4CCCC[N+]4C)c2	7.1
398	Fc1cccc(C(=O)c2cn(C[C@H]3CCCC[N+]3C)c4ccccc42)c1N	7.1
399	O=C([C@H]1c2ccccc2CC[N+]1)c3cn(C[C@H]4CCCC[N+]4C)c5ccccc53	7.1

400	Clc1ccc(F)cc1C(=O)c2c3ccccc3n(C[C@H]4CCCC[N+]4C)c2	7.1
401	Brc1ccc(Cl)c(C(=O)c2c3ccccc3n(C[C@H]4CCCC[N+]4C)c2)c1	7.1
402	O=C(c1c2ccccc2n(C[C@H]3CCCC[N+]3C)c1)/C(OC)=C/c4ccccc4	7.1
403	Fc1cccc(c1C)C(=O)c2cn(C[C@H]3CCCC[N+]3C)c4ccccc42	7.1
404	O=C(c1c2ccccc2n(C[C@H]3CCCC[N+]3C)c1)c4c(C)csc4	7.1
405	Fc1ccc(F)c(C(=O)C(=O)c2c3ccccc3n(C[C@H]4CCCC[N+]4C)c2)c1	7.1
406	O=C(C[C@H]1C[C@H]2CC[C@@H]1C2)c3c4ccccc4n(C[C@H]5CCCC[N+]5C)c3	7.1
407	O=C(c1cn(C[C@H]2CCCC[N+]2C)c3ccccc31)c4ccccc4C(=O)C	7.1
408	O=C(N[C@@H]1CCCC[C@H]1C[N+])c2c3ccccc3n(C[C@H]4CCCC[N+]4C)c2	7.1
409	O=C(C1=CCCCC1)c2c3ccccc3n(C[C@H]4CCCC[N+]4C)c2	7.1
410	FC(F)Oc1ccsc1C(=O)c2c3ccccc3n(C[C@H]4CCCC[N+]4C)c2	7.1
411	Clc1cccc(C(=O)c2c3ccccc3n(C[C@H]4CCCC[N+]4C)c2)c1O	7.1
412	O=C(c1c2ccccc2n(C[C@H]3CCCC[N+]3C)c1)c4cccc(NC(C)C)c4	7.1
413	O=C(c1c2ccccc2n(C[C@H]3CCCC[N+]3C)c1)c4c(O)ccc(c4)C	7.1
414	Clc1c(N)cccc1C(=O)c2c3ccccc3n(C[C@H]4CCCC[N+]4C)c2	7.1
415	Oc1ccc(C(C)C)cc1C(=O)c2c3ccccc3n(C[C@H]4CCCC[N+]4C)c2	7.1
416	Clc1c(cccc1C(=O)c2c3ccccc3n(C[C@H]4CCCC[N+]4C)c2)C	7
417	Sc1c(C(=O)c2c3ccccc3n(C[C@H]4CCCC[N+]4C)c2)cccn1	7
418	Clc1ccc(NC)c(C(=O)c2c3ccccc3n(C[C@H]4CCCC[N+]4C)c2)c1	7
419	O=C(C[C@H](CCC)C)c1c2ccccc2n(C[C@H]3CCCC[N+]3C)c1	7
420	Clc1cccc(Cl)c1OC(=O)c2c3ccccc3n(C[C@H]4CCCC[N+]4C)c2	7
421	Oc1ccc(O)c(C(=O)c2c3ccccc3n(C[C@H]4CCCC[N+]4C)c2)c1	7
422	O=C(c1c2ccccc2n(C[C@H]3CCCC[N+]3C)c1)c4c(CC)ccs4	7
423	Fc1ccc(N)cc1C(=O)c2c3ccccc3n(C[C@H]4CCCC[N+]4C)c2	7
424	O=C(N(c1cccc(c1)C)C)c2c3ccccc3n(C[C@H]4CCCC[N+]4C)c2	7
425	O=C(c1c2ccccc2n(C[C@H]3CCCC[N+]3C)c1)c4cncnc4C	7
426	Clc1ccc(Cl)nc1C(=O)c2c3ccccc3n(C[C@H]4CCCC[N+]4C)c2	7
427	Clc1c(F)cccc1C(=O)c2cn(C[C@H]3CCCC[N+]3C)c4ccccc42	7
428	Fc1ccc2c(OCCN2C(=O)c3c4ccccc4n(C[C@H]5CCCC[N+]5C)c3)c1	7
429	Fc1cc(F)cc2c1N(CCC2)C(=O)c3c4ccccc4n(C[C@H]5CCCC[N+]5C)c3	7
430	O=C(c1c2ccccc2n(C[C@H]3CCCC[N+]3C)c1)c4cc(C)cs4	7
431	O=C(CC1CCC1)c2c3ccccc3n(C[C@H]4CCCC[N+]4C)c2	7
432	O=S(=O)(c1c2ccccc2n(C[C@H]3CCCC[N+]3C)c1)c4cccs4	7
433	S=C(N)Cc1ccccc1C(=O)c2c3ccccc3n(C[C@H]4CCCC[N+]4C)c2	7
434	Clc1cc(Cl)cc(Cl)c1C(=O)c2c3ccccc3n(C[C@H]4CCCC[N+]4C)c2	7
435	O=C(c1c2ccccc2n(C[C@H]3CCCC[N+]3C)c1)c4c(C)ccs4	7
436	O=C([C@H]1CCCC[C@H]1C[N+])c2c3ccccc3n(C[C@H]4CCCC[N+]4C)c2	7
437	O=C(c1c2ccccc2n(C[C@H]3CCCC[N+]3C)c1)c4ccsc4	7
438	Clc1ccc(nc1C(=O)c2c3ccccc3n(C[C@H]4CCCC[N+]4C)c2)NC	7
439	Fc1cccc(C(=O)c2cn(C[C@H]3CCCC[N+]3C)c4ccccc42)c1OCC	7
440	O=C(N(c1ccc(cc1)C)C)c2c3ccccc3n(C[C@H]4CCCC[N+]4C)c2	7
441	Fc1c(ccc(F)c1C(=O)c2c3ccccc3n(C[C@H]4CCCC[N+]4C)c2)C	7

442	Oc1ccccc1C(=O)c2c3ccccc3n(C[C@H]4CCCC[N+]4C)c2	7
443	Fc1cccc(c1NCC)C(=O)c2cn(C[C@H]3CCCC[N+]3C)c4ccccc42	7
444	O=C([C@@H](C1CCCC1)C)c2c3ccccc3n(C[C@H]4CCCC[N+]4C)c2	6.9
445	Fc1ccccc1OCC(=O)c2c3ccccc3n(C[C@H]4CCCC[N+]4C)c2	6.9
446	Oc1ccc(OC)cc1C(=O)c2c3ccccc3n(C[C@H]4CCCC[N+]4C)c2	6.9
447	Fc1cccnc1C(=O)c2c3ccccc3n(C[C@H]4CCCC[N+]4C)c2	6.9
448	Clc1cccc(F)c1C(=O)c2c3ccccc3n(C[C@H]4CCCC[N+]4C)c2	6.9
449	O=C(C(CC)CC)c1c2ccccc2n(C[C@H]3CCCC[N+]3C)c1	6.9
450	O=C(c1cn(C[C@H]2CCCC[N+]2C)c3ccccc31)c4ccccc4-n5cccc5	6.9
451	FC(F)(F)c1cccnc1C(=O)c2c3ccccc3n(C[C@H]4CCCC[N+]4C)c2	6.9
452	Clc1ccc(nc1C(=O)c2c3ccccc3n(C[C@H]4CCCC[N+]4C)c2)N	6.9
453	O=C(c1c2ccccc2n(C[C@H]3CCCC[N+]3C)c1)c4c(C(C)C)ccs4	6.9
454	O=C([C@@H](OCCC)C)c1c2ccccc2n(C[C@H]3CCCC[N+]3C)c1	6.9
455	O=C([C@@H](C(C)C)C)c1c2ccccc2n(C[C@H]3CCCC[N+]3C)c1	6.9
456	Clc1ccc(S(=O)(=O)C)cc1C(=O)c2c3ccccc3n(C[C@H]4CCCC[N+]4C)c2	6.9
457	Fc1ccc(F)c(C(=O)c2c3ccccc3n(C[C@H]4CCCC[N+]4C)c2)c1OC	6.9
458	O=C(N[C@H]1CCSC1)c2c3ccccc3n(C[C@H]4CCCC[N+]4C)c2	6.9
459	Brc1ccc([N+]([O-])=O)cc1C(=O)c2c3ccccc3n(C[C@H]4CCCC[N+]4C)c2	6.9
460	Fc1cccc(C(=O)c2cn(C[C@H]3CCCC[N+]3C)c4ccccc42)c1NC	6.9
461	Clc1ccc(-n2cccn2)cc1C(=O)c3c4ccccc4n(C[C@H]5CCCC[N+]5C)c3	6.9
462	O=C([C@@H]1[C@](C1)(CC)C)c2c3ccccc3n(C[C@H]4CCCC[N+]4C)c2	6.9
463	O=C(c1cn(C[C@H]2CCCC[N+]2C)c3ccccc13)c4cccc5cc6ccccc6cc54	6.8
464	Clc1cccc(N(C(=O)c2c3ccccc3n(C[C@H]4CCCC[N+]4C)c2)CC)c1	6.8
465	Clc1cccc(Cl)c1C(=O)c2c3ccccc3n(C[C@H]4CCCC[N+]4C)c2	6.8
466	O=C(c1c2ccccc2n(C[C@H]3CCCC[N+]3C)c1)[C@@H](CC#C)C	6.8
467	O=C(c1c2ccccc2n(C[C@H]3CCCC[N+]3C)c1)c4cccc(C[N+])c4	6.8
468	O=C(c1c2ccccc2n(C[C@H]3CCCC[N+]3C)c1)/C=C/CC	6.8
469	O=C(C[C@@H]1C=CCC1)c2c3ccccc3n(C[C@H]4CCCC[N+]4C)c2	6.8
470	O=C(C1(CC1)c2cccc(c2)C)c3c4ccccc4n(C[C@H]5CCCC[N+]5C)c3	6.8
471	Clc1cccc(Cl)c1CS(=O)(=O)c2c3ccccc3n(C[C@H]4CCCC[N+]4C)c2	6.8
472	O=C(N1CCC[C@H]1C(C)C)c2c3ccccc3n(C[C@H]4CCCC[N+]4C)c2	6.8
473	O=C(CC1CCCCC1)c2c3ccccc3n(C[C@H]4CCCC[N+]4C)c2	6.8
474	O=C([C@H]1CCCC[C@@H]1[N+])c2c3ccccc3n(C[C@H]4CCCC[N+]4C)c2	6.8
475	Clc1cc(Cl)ccc1CS(=O)(=O)c2c3ccccc3n(C[C@H]4CCCC[N+]4C)c2	6.8
476	O=C([C@@H](SC)CC)c1c2ccccc2n(C[C@H]3CCCC[N+]3C)c1	6.8
477	FC(F)Oc1c(sc(c1)C)C(=O)c2c3ccccc3n(C[C@H]4CCCC[N+]4C)c2	6.8
478	O=C(c1c2ccccc2n(C[C@H]3CCCC[N+]3C)c1)c4cccc(c4)C(=O)N	6.8
479	Clc1cccc(O)c1C(=O)c2c3ccccc3n(C[C@H]4CCCC[N+]4C)c2	6.8
480	Oc1cccc(C(=O)c2c3ccccc3n(C[C@H]4CCCC[N+]4C)c2)c1O	6.8
481	Fc1cccc(C(=O)c2cn(C[C@H]3CCCC[N+]3C)c4ccccc42)c1NN	6.8
482	O=C(c1cn(C[C@H]2CCCC[N+]2C)c3ccccc31)c4ccccc4C(OC)=O	6.8
483	O=C(c1c2ccccc2n(C[C@H]3CCCC[N+]3C)c1)C(c4ccccc4)=C	6.7

484	O=C(C(C1CC1)C2CC2)c3c4ccccc4n(C[C@H]5CCCC[N+]5C)c3	6.7
485	O=C(c1c2ccccc2n(C[C@H]3CCCC[N+]3C)c1)c4ccccc4CC	6.7
486	O=C(c1cn(C[C@H]2CCCC[N+]2C)c3ccccc31)c4ccccc4-c5cnccc5	6.7
487	O=C([C@H]([N+]1CCCCCC1)C)c2cn(C[C@H]3CCCC[N+]3C)c4ccccc42	6.7
488	O=C(c1c2ccccc2n(C[C@H]3CCCC[N+]3C)c1)[C@@H](OCC=C)C	6.7
489	O=C(CC1CCCCCC1)c2c3ccccc3n(C[C@H]4CCCC[N+]4C)c2	6.6
490	O=C(c1c2ccccc2n(C[C@H]3CCCC[N+]3C)c1)COCC(C)C	6.6
491	O=C([C@@H](C1CC1)C)c2c3ccccc3n(C[C@H]4CCCC[N+]4C)c2	6.5
492	O=C(c1c2ccccc2n(C[C@H]3CCCC[N+]3C)c1)c4cccs4	6.5
493	Clc1cccc(C2(CC2)C(=O)c3c4ccccc4n(C[C@H]5CCCC[N+]5C)c3)c1	6.5
494	O=C(c1c2ccccc2n(C[C@H]3CCCC[N+]3C)c1)CCc4ccccc4	6.4
495	O=C([C@@]1(C[C@@H]1C[N+])c2cccs2)c3c4ccccc4n(C[C@H]5CCCC[N+]5C)c3	6.4
496	O=C(c1c2ccccc2n(C[C@H]3CCCC[N+]3C)c1)Cc4ccsc4	6.3
497	Fc1ccc(C(=O)C(=O)c2c3ccccc3n(C[C@H]4CCCC[N+]4C)c2)cc1	6.2
498	FC(F)(F)[C@@H](CC(=O)c1c2ccccc2n(C[C@H]3CCCC[N+]3C)c1)C	6.2
499	O=C(c1c2ccccc2n(C[C@H]3CCCC[N+]3C)c1)/C(C)=C/C	5.9
500	O=C([C@H]([N+])c1ccc(CC)cc1)c2c3ccccc3n(C[C@H]4CCCC[N+]4C)c2	5.8

Table S8. List, SMILE and predicted pK_i values for Series 4 in CB_1 receptor.

**N°**	**SMILES**	**Pred pK_i**
1	O=C(C1C(C1(C)C)(C)C)c2cn(c3ccccc32)CCSS([O-])(=O)=O	8.5
2	O=S(=O)(CCCn1cc(C(=O)C2C(C2(C)C)(C)C)c3ccccc31)CC#C	8.5
3	O=C(C1C(C1(C)C)(C)C)c2c3ccccc3n(CC4CCSCC4)c2	8.4
4	O=C(C1C(C1(C)C)(C)C)c2c3ccccc3n(CCCC4CCCC4)c2	8.3
5	O=C(C1C(C1(C)C)(C)C)c2c3ccccc3n(CCn4ccc(n4)C)c2	8.3
6	O=C(C1C(C1(C)C)(C)C)c2c3ccccc3n(Cc4ccnc(c4)C)c2	8.3
7	Fc1ccc(CCn2cc(C(=O)C3C(C3(C)C)(C)C)c4ccccc42)cc1C	8.2
8	O=C(C1C(C1(C)C)(C)C)c2c3ccccc3n(C[C@H](CC(C)(C)C)C)c2	8.2
9	O=C(C1C(C1(C)C)(C)C)c2c3ccccc3n(CCc4cnccc4)c2	8.2
10	O=C(C1C(C1(C)C)(C)C)c2cn(CC[C@@H]3C[C@H]4CC[C@@H]3C4)c5ccccc52	8.2
11	O=C(C1C(C1(C)C)(C)C)c2cn(c3ccccc32)CCSCC(C)C	8.2
12	O=C(C1C(C1(C)C)(C)C)c2c3ccccc3n(CC[C@H](O)C(C)=C)c2	8.1
13	Fc1ccc(Cn2cc(C(=O)C3C(C3(C)C)(C)C)c4ccccc42)cc1OC	8.1
14	Fc1cccc(Sn2cc(C(=O)C3C(C3(C)C)(C)C)c4ccccc42)c1	8.1
15	O=C(C1C(C1(C)C)(C)C)c2c3ccccc3n(CCC4=CCCCC4)c2	8.1
16	O=C(C1C(C1(C)C)(C)C)c2c3ccccc3n(Cc4ccsc4)c2	8.1
17	Clc1cc(Cn2cc(C(=O)C3C(C3(C)C)(C)C)c4ccccc42)ccn1	8.1
18	O=C(C1C(C1(C)C)(C)C)c2c3ccccc3n(Cc4ccncc4)c2	8.1
19	Fc1c(C)ccc(Cn2cc(C(=O)C3C(C3(C)C)(C)C)c4ccccc42)c1	8.1
20	O=C(C1C(C1(C)C)(C)C)c2c3ccccc3n(CCc4ccncc4)c2	8.1
21	Fc1cccc(Cn2cc(C(=O)C3C(C3(C)C)(C)C)c4ccccc42)c1	8.1

22	O=C(C1C(C1(C)C)(C)C)c2c3ccccc3n([C@@H](O)c4ccsc4)c2	8.1
23	O=C(C1C(C1(C)C)(C)C)c2c3ccccc3n(CCCCC(C)C)c2	8.1
24	O=C(C1C(C1(C)C)(C)C)c2c3ccccc3n(CCCCC=C)c2	8.1
25	Fc1ccc(O)c(Cn2cc(C(=O)C3C(C3(C)C)(C)C)c4ccccc42)c1	8
26	O=C(C1C(C1(C)C)(C)C)c2c3ccccc3n(CCCCCCC)c2	8
27	O[C@H](C1CC1)Cn2cc(C(=O)C3C(C3(C)C)(C)C)c4ccccc42	8
28	O=C(C1C(C1(C)C)(C)C)c2c3ccccc3n(Cc4ccc(s4)C)c2	8
29	Fc1c(O)ccc(Cn2cc(C(=O)C3C(C3(C)C)(C)C)c4ccccc42)c1	8
30	O=C(C1C(C1(C)C)(C)C)c2cn(SC3CCCCC3)c4ccccc42	8
31	Clc1ccc(Cn2cc(C(=O)C3C(C3(C)C)(C)C)c4ccccc42)cc1	8
32	FC(F)(F)C[C@@H](O)Cn1cc(C(=O)C2C(C2(C)C)(C)C)c3ccccc31	8
33	O=C(C1C(C1(C)C)(C)C)c2c3ccccc3n(CCCC(C)=C)c2	8
34	O=C(C1C(C1(C)C)(C)C)c2c3ccccc3n(CCCCCCC#N)c2	8
35	O=C(C1C(C1(C)C)(C)C)c2c3ccccc3n(CSCC4CC4)c2	8
36	O=C(C1C(C1(C)C)(C)C)c2c3ccccc3n(C[C@H](CC(C)C)C#N)c2	8
37	O=C(C1C(C1(C)C)(C)C)c2c3ccccc3n(CCC4CCCC4)c2	8
38	Clc1cccc(Cn2cc(C(=O)C3C(C3(C)C)(C)C)c4ccccc42)c1	7.9
39	O=C(C1C(C1(C)C)(C)C)c2c3ccccc3n(C[C@H](SCC)C)c2	7.9
40	O=C(C1C(C1(C)C)(C)C)c2c3ccccc3n(Cc4ccc(cc4)C#N)c2	7.9
41	O=S(=O)(CCS(=O)(=O)n1cc(C(=O)C2C(C2(C)C)(C)C)c3ccccc31)C	7.9
42	FC(F)(COCn1cc(C(=O)C2C(C2(C)C)(C)C)c3ccccc31)C(F)F	7.9
43	O=C(C1C(C1(C)C)(C)C)c2c3ccccc3n(CCCC4CCCCC4)c2	7.9
44	Clc1ccc(s1)Cn2cc(C(=O)C3C(C3(C)C)(C)C)c4ccccc42	7.9
45	O=C(C1C(C1(C)C)(C)C)c2c3ccccc3n(Cc4cnc(O)cc4)c2	7.9
46	ClC1(Cl)[C@H](C1)COn2cc(C(=O)C3C(C3(C)C)(C)C)c4ccccc42	7.9
47	O=C(C1C(C1(C)C)(C)C)c2c3ccccc3n(CC[C@@H]4COCCC4)c2	7.9
48	Clc1c(F)ccc(Cn2cc(C(=O)C3C(C3(C)C)(C)C)c4ccccc42)c1	7.9
49	O=C(C1C(C1(C)C)(C)C)c2c3ccccc3n(CC4=CC[C@@H](CC4)C)c2	7.9
50	O=C(C1C(C1(C)C)(C)C)c2cn([C@@H]([N+])CC3CCCC3)c4ccccc42	7.9
51	FC(F)(F)[C@@H](O)CSn1cc(C(=O)C2C(C2(C)C)(C)C)c3ccccc31	7.9
52	O=C(C1C(C1(C)C)(C)C)c2c3ccccc3n(CCCc4cnccc4)c2	7.9
53	Fc1cc(F)ccc1Cn2cc(C(=O)C3C(C3(C)C)(C)C)c4ccccc42	7.9
54	Clc1cc(F)c(Cn2cc(C(=O)C3C(C3(C)C)(C)C)c4ccccc42)cc1	7.9
55	O=C(C1C(C1(C)C)(C)C)c2cn(NCC[C@@H](O)C)c3ccccc32	7.9
56	O=S1(=O)CC[C@H](C1)CCn2cc(C(=O)C3C(C3(C)C)(C)C)c4ccccc42	7.9
57	O=C(C1C(C1(C)C)(C)C)c2c3ccccc3n(Cc4ccc5c(non5)c4)c2	7.9
58	O=C(C1C(C1(C)C)(C)C)c2c3ccccc3n(CC/C=C(\CO)C)c2	7.9
59	O=C(C1C(C1(C)C)(C)C)c2c3ccccc3n(C[C@@H]4CC[C@H](C4)C)c2	7.9
60	O=C(C1C(C1(C)C)(C)C)c2c3ccccc3n(CC[S@@](=O)C)c2	7.9
61	Fc1c(F)ccc(Sn2cc(C(=O)C3C(C3(C)C)(C)C)c4ccccc42)c1	7.8
62	Brc1cc(Cn2cc(C(=O)C3C(C3(C)C)(C)C)c4ccccc42)cs1	7.8
63	O=C(C1C(C1(C)C)(C)C)c2cn(NCC3CCC3)c4ccccc42	7.8

64	O=C(C1C(C1(C)C)(C)C)c2c3ccccc3n(CCC4CCC4)c2	7.8
65	O=C(C1C(C1(C)C)(C)C)c2cn(NCCC(C)(C)C)c3ccccc32	7.8
66	O=C(C1C(C1(C)C)(C)C)c2c3ccccc3n(CC4CCCC4)c2	7.8
67	FC(F)(F)OCCn1cc(C(=O)C2C(C2(C)C)(C)C)c3ccccc31	7.8
68	O=C(C1C(C1(C)C)(C)C)c2c3ccccc3n(Cc4ccoc4)c2	7.8
69	O=C(C1C(C1(C)C)(C)C)c2cn(SCC3CCCC3)c4ccccc42	7.8
70	FC(F)(F)CCCn1cc(C(=O)C2C(C2(C)C)(C)C)c3ccccc31	7.8
71	O=C(C1C(C1(C)C)(C)C)c2c3ccccc3n(CCCSCC)c2	7.8
72	O=C(C1C(C1(C)C)(C)C)c2c3ccccc3n(CCS(=O)(=O)C)c2	7.8
73	Fc1cncc(CCn2cc(C(=O)C3C(C3(C)C)(C)C)c4ccccc42)c1	7.8
74	O=C(C1C(C1(C)C)(C)C)c2c3ccccc3n(Cc4cnccc4)c2	7.8
75	Clc1ccc(s1)CSn2cc(C(=O)C3C(C3(C)C)(C)C)c4ccccc42	7.8
76	O=C(C1C(C1(C)C)(C)C)c2c3ccccc3n(C[S@@](=O)[C@H](CC)C)c2	7.8
77	O=C(C1C(C1(C)C)(C)C)c2c3ccccc3n(CC[C@H]4CCC(=O)N4)c2	7.8
78	O=C(C1C(C1(C)C)(C)C)c2c3ccccc3n(CCCCCC#C)c2	7.8
79	Clc1ccc(s1)CCn2cc(C(=O)C3C(C3(C)C)(C)C)c4ccccc42	7.8
80	O=C(C1C(C1(C)C)(C)C)c2c3ccccc3n(CCSC4CCOCC4)c2	7.8
81	O=C(C1C(C1(C)C)(C)C)c2c3ccccc3n(CCSC(C)(C)C)c2	7.8
82	O=C(C1C(C1(C)C)(C)C)c2c3ccccc3n(CCCC(C)(C)C)c2	7.8
83	FC(F)(F)CCNn1cc(C(=O)C2C(C2(C)C)(C)C)c3ccccc31	7.8
84	O=C(C1C(C1(C)C)(C)C)c2cn(c3ccccc32)CCSCC=C	7.8
85	FC1(F)CCC(CC1)Cn2cc(C(=O)C3C(C3(C)C)(C)C)c4ccccc42	7.8
86	O=C(C1C(C1(C)C)(C)C)c2cn(SC[C@H](CC)C)c3ccccc32	7.7
87	O=C(C1C(C1(C)C)(C)C)c2c3ccccc3n(CCCSCC#N)c2	7.7
88	O=C(C1C(C1(C)C)(C)C)c2cn(CCSCCOC)c3ccccc32	7.7
89	O=C(C1C(C1(C)C)(C)C)c2c3ccccc3n(CCc4ccc[nH]4)c2	7.7
90	O=C(C1C(C1(C)C)(C)C)c2c3ccccc3n(CCC4CC4)c2	7.7
91	O=C(C1C(C1(C)C)(C)C)c2c3ccccc3n(COCC(C)C)c2	7.7
92	O=C(C1C(C1(C)C)(C)C)c2cn([C@@H]([N+])Cc3cnccc3)c4ccccc42	7.7
93	O=C(C1C(C1(C)C)(C)C)c2c3ccccc3n(Cc4ccncn4)c2	7.7
94	O=C(C1C(C1(C)C)(C)C)c2c3ccccc3n(Cc4cc(cs4)C#N)c2	7.7
95	FC(F)(CCCn1cc(C(=O)C2C(C2(C)C)(C)C)c3ccccc31)C(F)(F)F	7.7
96	O=C(C1C(C1(C)C)(C)C)c2cn(Sc3cc(ncn3)C)c4ccccc42	7.7
97	Fc1c(F)ccc(CSn2cc(C(=O)C3C(C3(C)C)(C)C)c4ccccc42)c1	7.7
98	Clc1ccc(Cn2cc(C(=O)C3C(C3(C)C)(C)C)c4ccccc42)cc1F	7.7
99	O[C@H](C1CC1)CCn2cc(C(=O)C3C(C3(C)C)(C)C)c4ccccc42	7.7
100	ClC(Cl)CCn1cc(C(=O)C2C(C2(C)C)(C)C)c3ccccc31	7.7
101	O=S(=O)(CCCn1cc(C(=O)C2C(C2(C)C)(C)C)c3ccccc31)C	7.7
102	O=C(C1C(C1(C)C)(C)C)c2c3ccccc3n(C[N+]4CCCCC4)c2	7.7
103	O=C(C1C(C1(C)C)(C)C)c2c3ccccc3n(c2)CC#CCCC	7.7
104	Fc1cccc(SCn2cc(C(=O)C3C(C3(C)C)(C)C)c4ccccc42)c1	7.7
105	Clc1cccc(Sn2cc(C(=O)C3C(C3(C)C)(C)C)c4ccccc42)c1	7.7

106	O=C(C1C(C1(C)C)(C)C)c2cn(c3ccccc32)CCSCCC	7.7
107	O=C(C1C(C1(C)C)(C)C)c2cn(CCC(C)(C)C#N)c3ccccc32	7.7
108	O[C@H]([C@@H](CC)C)Cn1cc(C(=O)C2C(C2(C)C)(C)C)c3ccccc31	7.7
109	Fc1cnc(Cn2cc(C(=O)C3C(C3(C)C)(C)C)c4ccccc42)cc1	7.7
110	O=C(C1C(C1(C)C)(C)C)c2c3ccccc3n(CCSSC)c2	7.7
111	O=C(C1C(C1(C)C)(C)C)c2c3ccccc3n(Cc4cscc4C)c2	7.7
112	O=C(C1C(C1(C)C)(C)C)c2c3ccccc3n(CCC=C(C)C)c2	7.7
113	O=C(C1C(C1(C)C)(C)C)c2cn(NC[C@H]3CC=CCC3)c4ccccc42	7.7
114	O=C(C1C(C1(C)C)(C)C)c2c3ccccc3n(CCCC(C)C)c2	7.7
115	O=C(C1C(C1(C)C)(C)C)c2c3ccccc3n(Cc4ccc(o4)C)c2	7.7
116	SC(=S)NCCn1cc(C(=O)C2C(C2(C)C)(C)C)c3ccccc31	7.7
117	O=C(C1C(C1(C)C)(C)C)c2cn(SCc3cnccc3)c4ccccc42	7.6
118	Fc1ccc(Cn2cc(C(=O)C3C(C3(C)C)(C)C)c4ccccc42)cc1C	7.6
119	FC(SCCn1cc(C(=O)C2C(C2(C)C)(C)C)c3ccccc31)(F)F	7.6
120	Clc1cc(F)ccc1Sn2cc(C(=O)C3C(C3(C)C)(C)C)c4ccccc42	7.6
121	ClC(C[N+]Cn1cc(C(=O)C2C(C2(C)C)(C)C)c3ccccc31)=C	7.6
122	O=C(C1C(C1(C)C)(C)C)c2c3ccccc3n(CSCC#N)c2	7.6
123	Fc1c(F)ccc(Cn2cc(C(=O)C3C(C3(C)C)(C)C)c4ccccc42)c1	7.6
124	O=C(C1C(C1(C)C)(C)C)c2cn(Sc3ccncn3)c4ccccc42	7.6
125	Fc1ccc([C@H](O)n2cc(C(=O)C3C(C3(C)C)(C)C)c4ccccc42)cc1	7.6
126	O=C(C1C(C1(C)C)(C)C)c2c3ccccc3n(Cc4cc(C(OC)=O)co4)c2	7.6
127	FC(F)(F)COCCn1cc(C(=O)C2C(C2(C)C)(C)C)c3ccccc31	7.6
128	O=C(C1C(C1(C)C)(C)C)c2cn(NCC3CC3)c4ccccc42	7.6
129	O=C(C1C(C1(C)C)(C)C)c2c3ccccc3n(CCCc4c[nH]nc4C)c2	7.6
130	O=C(C1C(C1(C)C)(C)C)c2cn(SCCC(C)(C)C)c3ccccc32	7.6
131	O=C(C1C(C1(C)C)(C)C)c2c3ccccc3n(Cc4csc([N+]([O-])=O)c4)c2	7.6
132	O=C(C1C(C1(C)C)(C)C)c2c3ccccc3n(CSCC4CCCC4)c2	7.6
133	ClC(CCn1cc(C(=O)C2C(C2(C)C)(C)C)c3ccccc31)(C)C	7.6
134	O=C(C1C(C1(C)C)(C)C)c2cn(SCc3ccsc3)c4ccccc42	7.6
135	O=C(C1C(C1(C)C)(C)C)c2c3ccccc3n(CCCO[N+]([O-])=O)c2	7.6
136	O=C(C1C(C1(C)C)(C)C)c2c3ccccc3n(CC[C@@H]4C(C(OC4)=O)=C)c2	7.6
137	O=C(C1C(C1(C)C)(C)C)c2cn(OCc3ccsc3)c4ccccc42	7.6
138	O=C(C1C(C1(C)C)(C)C)c2c3ccccc3n(C[n+]4ccc(C)cc4)c2	7.6
139	O=C(C1C(C1(C)C)(C)C)c2c3ccccc3n(CCCCOC)c2	7.6
140	O=C(C1C(C1(C)C)(C)C)c2c3ccccc3n(CCC(CC)CC)c2	7.6
141	SCC1(Cn2cc(C(=O)C3C(C3(C)C)(C)C)c4ccccc42)CC1	7.6
142	O=C(C1C(C1(C)C)(C)C)c2cn(OCCCC)c3ccccc32	7.6
143	O=C(C1C(C1(C)C)(C)C)c2c3ccccc3n(C[N+][C@@H]4C(C4)(C)C)c2	7.6
144	O=C(C1C(C1(C)C)(C)C)c2c3ccccc3n(CC[C@@H](C)C#N)c2	7.6
145	O=C(C1C(C1(C)C)(C)C)c2c3ccccc3n(C[n+]4cscc4)c2	7.6
146	FCCCNn1cc(C(=O)C2C(C2(C)C)(C)C)c3ccccc31	7.6
147	O=C(C1C(C1(C)C)(C)C)c2c3ccccc3n(CC[C@@H](SC)C)c2	7.6

148	O=C(C1C(C1(C)C)(C)C)c2cn(SS[C@@H](CC)C)c3ccccc32	7.6
149	O=C(C1C(C1(C)C)(C)C)c2c3ccccc3n(CC[C@H]4CCC[C@H](C4)C)c2	7.6
150	O=C(C1C(C1(C)C)(C)C)c2c3ccccc3n(CC4CC(C4)C)c2	7.6
151	O=C(C1C(C1(C)C)(C)C)c2cn(OCC3CCC3)c4ccccc42	7.6
152	FC(F)(F)CCSn1cc(C(=O)C2C(C2(C)C)(C)C)c3ccccc31	7.6
153	O=C(C1C(C1(C)C)(C)C)c2cn(CCSCC)c3ccccc32	7.6
154	O=C(C1C(C1(C)C)(C)C)c2c3ccccc3n(CNc4ccncn4)c2	7.6
155	FC(F)(Cn1cc(C(=O)C2C(C2(C)C)(C)C)c3ccccc31)[C@H](F)C(F)(F)F	7.6
156	O=C(C1C(C1(C)C)(C)C)c2c3ccccc3n(CCCC=4[N-]N=NN4)c2	7.6
157	O=C(C1C(C1(C)C)(C)C)c2c3ccccc3n(C[S@@](=O)CC)c2	7.6
158	Clc1ccc(Sn2cc(C(=O)C3C(C3(C)C)(C)C)c4ccccc42)cc1	7.6
159	Fc1ccc(Cn2cc(C(=O)C3C(C3(C)C)(C)C)c4ccccc42)cc1	7.6
160	O=C(C1C(C1(C)C)(C)C)c2cn(OC[C@@H]3[C@H](C3)C)c4ccccc42	7.6
161	O=C(C1C(C1(C)C)(C)C)c2c3ccccc3n(C[n+]4ccccc4)c2	7.5
162	Fc1cn(nn1)Cn2cc(C(=O)C3C(C3(C)C)(C)C)c4ccccc42	7.5
163	O=C(C1C(C1(C)C)(C)C)c2cn(NCCSC)c3ccccc32	7.5
164	O=C(C1C(C1(C)C)(C)C)c2c3ccccc3n(CCCc4cn[nH]c4C)c2	7.5
165	O=C(C1C(C1(C)C)(C)C)c2c3ccccc3n([C@@H]([N+])CCC(C)C)c2	7.5
166	O=C(C1C(C1(C)C)(C)C)c2c3ccccc3n(Cc4ccco4)c2	7.5
167	O=C(C1C(C1(C)C)(C)C)c2c3ccccc3n(CCCC4CC4)c2	7.5
168	O=C(C1C(C1(C)C)(C)C)c2cn(OC[C@H]3CS3)c4ccccc42	7.5
169	ClC(Cl)=CCOn1cc(C(=O)C2C(C2(C)C)(C)C)c3ccccc31	7.5
170	O=C(C1C(C1(C)C)(C)C)c2c3ccccc3n(CCc4cncs4)c2	7.5
171	O=C(C1C(C1(C)C)(C)C)c2c3ccccc3n(CSC4CCCC4)c2	7.5
172	O=C(C1C(C1(C)C)(C)C)c2c3ccccc3n(CCCOC(C)C)c2	7.5
173	Fc1ccc([S@@](=O)n2cc(C(=O)C3C(C3(C)C)(C)C)c4ccccc42)cc1	7.5
174	O=C(C1C(C1(C)C)(C)C)c2cn(Sc3ncns3)c4ccccc42	7.5
175	O=C(C1C(C1(C)C)(C)C)c2cn(Sc3cnccc3)c4ccccc42	7.5
176	Cl/C=C/COn1cc(C(=O)C2C(C2(C)C)(C)C)c3ccccc31	7.5
177	O=C(C1C(C1(C)C)(C)C)c2c3ccccc3n(CC[N+]C(C)C)c2	7.5
178	FC(F)(F)C[C@H](Cn1cc(C(=O)C2C(C2(C)C)(C)C)c3ccccc31)C	7.5
179	O=C(C1C(C1(C)C)(C)C)c2cn(SCCCC)c3ccccc32	7.5
180	O=C(C1C(C1(C)C)(C)C)c2cn(OCCS(=O)(=O)C)c3ccccc32	7.5
181	O=C(C1C(C1(C)C)(C)C)c2c3ccccc3n(CSCCCC)c2	7.5
182	O=C(C1C(C1(C)C)(C)C)c2c3ccccc3n(CC[C@H]4C=CCC4)c2	7.5
183	Brc1ccc[n+](Cn2cc(C(=O)C3C(C3(C)C)(C)C)c4ccccc42)c1	7.5
184	O=C(C1C(C1(C)C)(C)C)c2cn(SCCc3ccncc3)c4ccccc42	7.5
185	Fc1cc(Cn2cc(C(=O)C3C(C3(C)C)(C)C)c4ccccc42)ccn1	7.5
186	FC(F)(F)CSCn1cc(C(=O)C2C(C2(C)C)(C)C)c3ccccc31	7.5
187	FC(F)(F)C[N+]Cn1cc(C(=O)C2C(C2(C)C)(C)C)c3ccccc31	7.5
188	O=C(C1C(C1(C)C)(C)C)c2c3ccccc3n(CC(CC)CC)c2	7.5
189	O=C(C1C(C1(C)C)(C)C)c2c3ccccc3n(C[N+][C@H]4[C@H](CCC4)C)c2	7.5

190	O=C(C1C(C1(C)C)(C)C)c2c3ccccc3n(C[S@@](=O)C4CCCC4)c2	7.5
191	O=C(C1C(C1(C)C)(C)C)c2c3ccccc3n(CC[N+]4CC[C@H](C4)C)c2	7.5
192	O=C(C1C(C1(C)C)(C)C)c2cn(c3ccccc32)CCC(C)(C)C	7.4
193	O=C(C1C(C1(C)C)(C)C)c2c3ccccc3n(C[C@H](c4ccsc4)C)c2	7.4
194	O=C(C1C(C1(C)C)(C)C)c2cn(SCCSCC)c3ccccc32	7.4
195	O=C(C1C(C1(C)C)(C)C)c2cn(SSC3CCCCC3)c4ccccc42	7.4
196	O=C(C1C(C1(C)C)(C)C)c2cn(Sc3ccncc3)c4ccccc42	7.4
197	FC(SCn1cc(C(=O)C2C(C2(C)C)(C)C)c3ccccc31)(F)F	7.4
198	O=C(C1C(C1(C)C)(C)C)c2c3ccccc3n(CC[C@@H]4CCOC4)c2	7.4
199	O=C(C1C(C1(C)C)(C)C)c2c3ccccc3n(CC[C@@H]([S@](=O)C)C)c2	7.4
200	O=C(C1C(C1(C)C)(C)C)c2cn([S@](=O)CCCC)c3ccccc32	7.4
201	O=C(C1C(C1(C)C)(C)C)c2c3ccccc3n(CSCCCCC)c2	7.4
202	O=C(C1C(C1(C)C)(C)C)c2cn(SCC3CCOCC3)c4ccccc42	7.4
203	O=C(C1C(C1(C)C)(C)C)c2c3ccccc3n(CCCCC)c2	7.4
204	O=C(C1C(C1(C)C)(C)C)c2c3ccccc3n(CO[C@H]4CCOC4)c2	7.4
205	O=C(C1C(C1(C)C)(C)C)c2cn(NCCC(C)C)c3ccccc32	7.4
206	O=C(C1C(C1(C)C)(C)C)c2c3ccccc3n(S(=O)(=O)NC4CCC4)c2	7.4
207	O=C(C1C(C1(C)C)(C)C)c2cn(OCCSCC)c3ccccc32	7.4
208	O=C(C1C(C1(C)C)(C)C)c2c3ccccc3n(CC[C@H](CC)C)c2	7.4
209	O=C(C1C(C1(C)C)(C)C)c2c3ccccc3n(CCC4CCC(CC4)C)c2	7.4
210	O=C(C1C(C1(C)C)(C)C)c2c3ccccc3n(CC4=CCCCC4)c2	7.4
211	Fc1c(F)c(F)cc(Cn2cc(C(=O)C3C(C3(C)C)(C)C)c4ccccc42)c1	7.4
212	O=C(C1C(C1(C)C)(C)C)c2c3ccccc3n(CS[C@@H](CC)C)c2	7.4
213	O=C(C1C(C1(C)C)(C)C)c2cn(SCCS(=O)(=O)C)c3ccccc32	7.4
214	O=C(C1C(C1(C)C)(C)C)c2c3ccccc3n(CCCCC#C)c2	7.4
215	O=C(C1C(C1(C)C)(C)C)c2c3ccccc3n(CCCOC=C)c2	7.4
216	O=C(C1C(C1(C)C)(C)C)c2cn(Sc3cnc(cn3)C#N)c4ccccc42	7.4
217	O=C(C1C(C1(C)C)(C)C)c2cn(SCC3CCCCC3)c4ccccc42	7.4
218	Fc1ccc(Sn2cc(C(=O)C3C(C3(C)C)(C)C)c4ccccc42)cc1	7.4
219	O=C(C1C(C1(C)C)(C)C)c2c3ccccc3n(COC4CCCC4)c2	7.4
220	O=C(C1C(C1(C)C)(C)C)c2c3ccccc3n(C[N+]CCS([O-])(=O)=O)c2	7.4
221	O=C(C1C(C1(C)C)(C)C)c2c3ccccc3n(COCC4CC4)c2	7.4
222	O=C(C1C(C1(C)C)(C)C)c2c3ccccc3n(COCc4ccncc4)c2	7.4
223	O=C(C1C(C1(C)C)(C)C)c2c3ccccc3n(C/C=C/CC)c2	7.4
224	O=C(C1C(C1(C)C)(C)C)c2cn(c3ccccc32)/C=C\CC(C)C	7.4
225	O=C(C1C(C1(C)C)(C)C)c2c3ccccc3n(C[N+]CC4CCC4)c2	7.4
226	O=C(C1C(C1(C)C)(C)C)c2cn(SC[C@@H](O)CC)c3ccccc32	7.4
227	S=C(N)CCCn1cc(C(=O)C2C(C2(C)C)(C)C)c3ccccc31	7.4
228	O=C(C1C(C1(C)C)(C)C)c2cn(Sc3ccc(o3)C=O)c4ccccc42	7.4
229	O=C(C1C(C1(C)C)(C)C)c2cn(SCCc3cnccc3)c4ccccc42	7.4
230	O=C(C1C(C1(C)C)(C)C)c2c3ccccc3n(COC4CCOCC4)c2	7.4
231	O=C(C1C(C1(C)C)(C)C)c2cn(NCCC)c3ccccc32	7.4

232	O=C(C1C(C1(C)C)(C)C)c2c3ccccc3n(CCC[C@@H]4CCOC4)c2	7.4
233	O=C(C1C(C1(C)C)(C)C)c2c3ccccc3n(CCC[C@@H]([N+]C)C)c2	7.4
234	O=C(C1C(C1(C)C)(C)C)c2c3ccccc3n(CC[C@H]4CO4)c2	7.3
235	O=C(C1C(C1(C)C)(C)C)c2c3ccccc3n(CSCC(C)C)c2	7.3
236	O=C(C1C(C1(C)C)(C)C)c2cn(SCCC(C)C)c3ccccc32	7.3
237	Fc1c(F)ccc(CCn2cc(C(=O)C3C(C3(C)C)(C)C)c4ccccc42)c1	7.3
238	O=C(C1C(C1(C)C)(C)C)c2c3ccccc3n(C[N+]C4CCC4)c2	7.3
239	O=C(C1C(C1(C)C)(C)C)c2c3ccccc3n(C[C@@H]4C[C@@H](OC)C[N+]4)c2	7.3
240	O=C(C1C(C1(C)C)(C)C)c2cn(SCC3CC3)c4ccccc42	7.3
241	O=C(C1C(C1(C)C)(C)C)c2c3ccccc3n(C[S@@](=O)c4cccs4)c2	7.3
242	O=C(C1C(C1(C)C)(C)C)c2c3ccccc3n(CC4=CCCC4)c2	7.3
243	O=C(C1C(C1(C)C)(C)C)c2c3ccccc3n(C[S+]4CCCC4)c2	7.3
244	O=C(C1C(C1(C)C)(C)C)c2c3ccccc3n(CCc4ccco4)c2	7.3
245	O=C(C1C(C1(C)C)(C)C)c2c3ccccc3n(CCOC=C)c2	7.3
246	FCCCCCn1cc(C(=O)C2C(C2(C)C)(C)C)c3ccccc31	7.3
247	O=C(C1C(C1(C)C)(C)C)c2c3ccccc3n(C/C=C/C(C)C)c2	7.3
248	O=C(C1C(C1(C)C)(C)C)c2c3ccccc3n(COC4CC4)c2	7.3
249	O=C(C1C(C1(C)C)(C)C)c2cn(NC[C@@H]3CCOC3)c4ccccc42	7.3
250	O=C(C1C(C1(C)C)(C)C)c2c3ccccc3n(COc4ccncc4)c2	7.3
251	O=C(C1C(C1(C)C)(C)C)c2c3ccccc3n(C[C@@H]([N+])CCC)c2	7.3
252	O=C(C1C(C1(C)C)(C)C)c2c3ccccc3n(C[n+]4csc(c4)C)c2	7.3
253	O=C(C1C(C1(C)C)(C)C)c2c3ccccc3n(CCC4CCCCC4)c2	7.3
254	O=C(C1C(C1(C)C)(C)C)c2cn([C@H](CCSC)C)c3ccccc32	7.3
255	I/C=C/Cn1cc(C(=O)C2C(C2(C)C)(C)C)c3ccccc31	7.3
256	Clc1cc(CCn2cc(C(=O)C3C(C3(C)C)(C)C)c4ccccc42)cs1	7.3
257	O=C(C1C(C1(C)C)(C)C)c2c3ccccc3n(CC[N+][C@H](C4CC4)C)c2	7.3
258	O=S(=O)(CC(C)C)Cn1cc(C(=O)C2C(C2(C)C)(C)C)c3ccccc31	7.3
259	IC#CCn1cc(C(=O)C2C(C2(C)C)(C)C)c3ccccc31	7.3
260	O=C(C1C(C1(C)C)(C)C)c2c3ccccc3n(c2)CC#CCC#C	7.3
261	O=C(C1C(C1(C)C)(C)C)c2c3ccccc3n(CCCCC(=O)C)c2	7.3
262	Fc1cccc(CSn2cc(C(=O)C3C(C3(C)C)(C)C)c4ccccc42)c1	7.2
263	O=C(C1C(C1(C)C)(C)C)c2c3ccccc3n(CC[N+]C(C)(C)C)c2	7.2
264	O=C(C1C(C1(C)C)(C)C)c2cn(SCc3cccs3)c4ccccc42	7.2
265	O=C(C1C(C1(C)C)(C)C)c2cn(SC[C@H]3CCC[N+]3)c4ccccc42	7.2
266	O=C(C1C(C1(C)C)(C)C)c2cn(NCCCSC)c3ccccc32	7.2
267	O=C(C1C(C1(C)C)(C)C)c2cn(NCCC#N)c3ccccc32	7.2
268	O=C(C1C(C1(C)C)(C)C)c2c3ccccc3n(CCCC#C)c2	7.2
269	O=C(C1C(C1(C)C)(C)C)c2c3ccccc3n(Cc4ccc(o4)C=O)c2	7.2
270	O=C(C1C(C1(C)C)(C)C)c2c3ccccc3n(CC[N+]4CC=CCC4)c2	7.2
271	O=C(C1C(C1(C)C)(C)C)c2cn(SC3CCCC3)c4ccccc42	7.2
272	O=C(C1C(C1(C)C)(C)C)c2c3ccccc3n(CCCCSC)c2	7.2
273	O=C(C1C(C1(C)C)(C)C)c2cn(SCCC#C)c3ccccc32	7.2

274	O=C(C1C(C1(C)C)(C)C)c2c3ccccc3n(CC[N+]4CC4)c2	7.2
275	FC(F)(F)CSn1cc(C(=O)C2C(C2(C)C)(C)C)c3ccccc31	7.2
276	SCCCCn1cc(C(=O)C2C(C2(C)C)(C)C)c3ccccc31	7.2
277	O=C(C1C(C1(C)C)(C)C)c2c3ccccc3n(C[C@H](C4CC4)C)c2	7.2
278	O=C(C1C(C1(C)C)(C)C)c2cn(SC/C=C/C)c3ccccc32	7.2
279	FCCOCCn1cc(C(=O)C2C(C2(C)C)(C)C)c3ccccc31	7.2
280	O=C(C1C(C1(C)C)(C)C)c2c3ccccc3n(c2)CC=C(C)C	7.2
281	O=C(C1C(C1(C)C)(C)C)c2c3ccccc3n(C[N+][C@@H](CC)C)c2	7.2
282	O=C(C1C(C1(C)C)(C)C)c2cn(NC[C@@H]3CCC=CO3)c4ccccc42	7.2
283	O=C(C1C(C1(C)C)(C)C)c2c3ccccc3n(CCSC(C)C)c2	7.2
284	Clc1c(F)ccc(Sn2cc(C(=O)C3C(C3(C)C)(C)C)c4ccccc42)c1	7.2
285	FC(F)(F)CCCCn1cc(C(=O)C2C(C2(C)C)(C)C)c3ccccc31	7.2
286	O=C(C1C(C1(C)C)(C)C)c2cn([C@@H]([N+])CC3CC3)c4ccccc42	7.2
287	O=C(C1C(C1(C)C)(C)C)c2c3ccccc3n(C[C@@H]4[C@H](C4)C)c2	7.2
288	O=C(C1C(C1(C)C)(C)C)c2c3ccccc3n(CCCCCC)c2	7.2
289	O=C(C1C(C1(C)C)(C)C)c2cn(c3ccccc32)CCSC	7.2
290	O=C(C1C(C1(C)C)(C)C)c2c3ccccc3n(CCCc4c(noc4C)C)c2	7.2
291	O=C(C1C(C1(C)C)(C)C)c2c3ccccc3n(COCCC)c2	7.2
292	FC(F)CNn1cc(C(=O)C2C(C2(C)C)(C)C)c3ccccc31	7.2
293	O=C(C1C(C1(C)C)(C)C)c2cn(NCC#CC)c3ccccc32	7.2
294	F[C@H]1CC[C@@H]([N+]Cn2cc(C(=O)C3C(C3(C)C)(C)C)c4ccccc42)C1	7.2
295	O=C(C1C(C1(C)C)(C)C)c2c3ccccc3n(CC[C@H]4CCCC[N+]4)c2	7.2
296	FCCCOn1cc(C(=O)C2C(C2(C)C)(C)C)c3ccccc31	7.1
297	O=C(C1C(C1(C)C)(C)C)c2c3ccccc3n(CCC4(N=N4)C)c2	7.1
298	O=C(C1C(C1(C)C)(C)C)c2cn(c3ccccc32)/C=C\C=C/CC	7.1
299	O=C(C1C(C1(C)C)(C)C)c2c3ccccc3n(CCCN(C)C(=O)C)c2	7.1
300	O=C(C1C(C1(C)C)(C)C)c2c3ccccc3n(CSc4cccs4)c2	7.1
301	O=C(C1C(C1(C)C)(C)C)c2c3ccccc3n(Cc4ccc(o4)C#N)c2	7.1
302	O=C(C1C(C1(C)C)(C)C)c2c3ccccc3n(CC/C=C\CC)c2	7.1
303	O=C(C1C(C1(C)C)(C)C)c2cn(SSSC)c3ccccc32	7.1
304	O=C(C1C(C1(C)C)(C)C)c2c3ccccc3n(C/C=C/C)c2	7.1
305	O=C(C1C(C1(C)C)(C)C)c2cn(c3ccccc32)C(CCCC)=C	7.1
306	O=C(C1C(C1(C)C)(C)C)c2c3ccccc3n(CC[C@H](O)CC)c2	7.1
307	O=C(C1C(C1(C)C)(C)C)c2c3ccccc3n(CCc4cnccn4)c2	7.1
308	O=C(C1C(C1(C)C)(C)C)c2c3ccccc3n(CC[C@@H](O)C)c2	7.1
309	ClC(Cl)=CCn1cc(C(=O)C2C(C2(C)C)(C)C)c3ccccc31	7.1
310	O=C(C1C(C1(C)C)(C)C)c2cn(N/C=C/C)c3ccccc32	7.1
311	O=C(C1C(C1(C)C)(C)C)c2c3ccccc3n(CCSc4cnn[nH]4)c2	7.1
312	BrC(C[N+]Cn1cc(C(=O)C2C(C2(C)C)(C)C)c3ccccc31)=C	7.1
313	O=C(C1C(C1(C)C)(C)C)c2c3ccccc3n(CC/C=C/C)c2	7.1
314	O=C(C1C(C1(C)C)(C)C)c2c3ccccc3n(S(=O)(=O)NCC(C)(C)C)c2	7.1
315	O=C(C1C(C1(C)C)(C)C)c2cn([C@@H]([N+])CCCCC)c3ccccc32	7.1

316	O=C(C1C(C1(C)C)(C)C)c2cn(OCCSC)c3ccccc32	7.1
317	O=C(C1C(C1(C)C)(C)C)c2c3ccccc3n(C[C@H](CCC)C)c2	7.1
318	O=C(C1C(C1(C)C)(C)C)c2cn(OCC3CC3)c4ccccc42	7.1
319	O=C(C1C(C1(C)C)(C)C)c2c3ccccc3n(CCC(C)C)c2	7.1
320	FC(F)(Cn1cc(C(=O)C2C(C2(C)C)(C)C)c3ccccc31)C(F)F	7.1
321	O=C(C1C(C1(C)C)(C)C)c2c3ccccc3n(CC[C@@H]4CC[N+]C4)c2	7.1
322	FC(F)(F)C[N+](CCn1cc(C(=O)C2C(C2(C)C)(C)C)c3ccccc31)C	7.1
323	FC1(F)CC[N+](C1)CCn2cc(C(=O)C3C(C3(C)C)(C)C)c4ccccc42	7.1
324	FC([S@@](=O)n1cc(C(=O)C2C(C2(C)C)(C)C)c3ccccc31)(F)C(F)F	7.1
325	O=C(C1C(C1(C)C)(C)C)c2c3ccccc3n(CSC(C)C)c2	7.1
326	FC(F)(F)CCn1cc(C(=O)C2C(C2(C)C)(C)C)c3ccccc31	7.1
327	O=[S@](CC(C)C)Cn1cc(C(=O)C2C(C2(C)C)(C)C)c3ccccc31	7.1
328	O=C(C1C(C1(C)C)(C)C)c2c3ccccc3n(C[N+]C4CCCC4)c2	7.1
329	O=C(C1C(C1(C)C)(C)C)c2c3ccccc3n(CNC=4CCCCC[N+]4)c2	7.1
330	O=C(C1C(C1(C)C)(C)C)c2c3ccccc3n(CCCOCC=C)c2	7.1
331	Cl/C(=C\Cn1cc(C(=O)C2C(C2(C)C)(C)C)c3ccccc31)C	7.1
332	O=C(C1C(C1(C)C)(C)C)c2c3ccccc3n(C[N+]CC(C)C)c2	7.1
333	Cl[C@@H](F)C([S@@](=O)n1cc(C(=O)C2C(C2(C)C)(C)C)c3ccccc31)(F)F	7.1
334	Cl[C@@H](F)C(Sn1cc(C(=O)C2C(C2(C)C)(C)C)c3ccccc31)(F)F	7.1
335	O=C(C1C(C1(C)C)(C)C)c2cn(SCC=C(C)C)c3ccccc32	7.1
336	O=C(C1C(C1(C)C)(C)C)c2c3ccccc3n(C[C@@H]4CCC[N+]4)c2	7
337	FC(F)COCCn1cc(C(=O)C2C(C2(C)C)(C)C)c3ccccc31	7
338	O=C(C1C(C1(C)C)(C)C)c2c3ccccc3n(COCCC(C)C)c2	7
339	O=S1(=O)CC[C@@H](Sn2cc(C(=O)C3C(C3(C)C)(C)C)c4ccccc42)C1	7
340	O=C(C1C(C1(C)C)(C)C)c2c3ccccc3n(C[N+]4CCCC4)c2	7
341	O=C(C1C(C1(C)C)(C)C)c2c3ccccc3n(C[N+]C4CC4)c2	7
342	O=C(C1C(C1(C)C)(C)C)c2c3ccccc3n(CC[S@@](=O)CC)c2	7
343	O=C(C1C(C1(C)C)(C)C)c2cn(SSCC)c3ccccc32	7
344	O=C(C1C(C1(C)C)(C)C)c2cn(NCCCCC)c3ccccc32	7
345	O=C(C1C(C1(C)C)(C)C)c2c3ccccc3n(CC[N+]4CCC4)c2	7
346	O=C(C1C(C1(C)C)(C)C)c2c3ccccc3n([C@H](O)CC[N+](C)C)c2	7
347	O=C(C1C(C1(C)C)(C)C)c2c3ccccc3n(C[C@H]4[C@@H](C4)CO)c2	7
348	O=C(C1C(C1(C)C)(C)C)c2cn(SC3COC3)c4ccccc42	7
349	FC1(F)CC(C1)Cn2cc(C(=O)C3C(C3(C)C)(C)C)c4ccccc42	7
350	O=C(C1C(C1(C)C)(C)C)c2c3ccccc3n(CSCCOC)c2	7
351	O=C(C1C(C1(C)C)(C)C)c2c3ccccc3n(COC(C)C)c2	7
352	O=C(C1C(C1(C)C)(C)C)c2cn([C@@H]([N+])CC(C)C)c3ccccc32	7
353	O=C(C1C(C1(C)C)(C)C)c2c3ccccc3n(CCCN(S(=O)(=O)C)C)c2	7
354	FC(F)C[N+](Cn1cc(C(=O)C2C(C2(C)C)(C)C)c3ccccc31)C	7
355	O=C(C1C(C1(C)C)(C)C)c2cn(SCSC)c3ccccc32	7
356	FCCCn1cc(C(=O)C2C(C2(C)C)(C)C)c3ccccc31	7
357	O=C(C1C(C1(C)C)(C)C)c2c3ccccc3n(Cc4ccc(o4)CO)c2	7

358	O=C(C1C(C1(C)C)(C)C)c2c3ccccc3n(C[C@H]([N+]CC#C)C)c2	6.9
359	O=C(C1C(C1(C)C)(C)C)c2c3ccccc3n(C[C@H]4CCCC[N+]4)c2	6.9
360	O=C(C1C(C1(C)C)(C)C)c2c3ccccc3n(C/C=C/C#C)c2	6.9
361	FC(F)CCn1cc(C(=O)C2C(C2(C)C)(C)C)c3ccccc31	6.9
362	O=C(C1C(C1(C)C)(C)C)c2c3ccccc3n(CCCSC)c2	6.9
363	O=C(C1C(C1(C)C)(C)C)c2c3ccccc3n(CCCCC#N)c2	6.9
364	O=C(C1C(C1(C)C)(C)C)c2c3ccccc3n(CC[C@H]([N+]C)C)c2	6.9
365	O=S(=O)(Cn1cc(C(=O)C2C(C2(C)C)(C)C)c3ccccc31)CC	6.9
366	O=C(C1C(C1(C)C)(C)C)c2cn([C@@H]([N+])CCC)c3ccccc32	6.9
367	O=C(C1C(C1(C)C)(C)C)c2cn(OCSC)c3ccccc32	6.9
368	O=C(C1C(C1(C)C)(C)C)c2c3ccccc3n(CCCOC)c2	6.9
369	SCCCOn1cc(C(=O)C2C(C2(C)C)(C)C)c3ccccc31	6.9
370	O=C(C1C(C1(C)C)(C)C)c2c3ccccc3n(C[N+]Cc4ccoc4)c2	6.9
371	BrC(CCn1cc(C(=O)C2C(C2(C)C)(C)C)c3ccccc31)=C	6.9
372	FCCCCn1cc(C(=O)C2C(C2(C)C)(C)C)c3ccccc31	6.9
373	O=C(C1C(C1(C)C)(C)C)c2c3ccccc3n(CC[C@@H](C(C)C)C#N)c2	6.9
374	FC(F)(F)CCOn1cc(C(=O)C2C(C2(C)C)(C)C)c3ccccc31	6.9
375	O=C(C1C(C1(C)C)(C)C)c2c3ccccc3n(COCC)c2	6.9
376	FCCOn1cc(C(=O)C2C(C2(C)C)(C)C)c3ccccc31	6.9
377	O=C(C1C(C1(C)C)(C)C)c2c3ccccc3n(C[N+]Cc4ccc[nH]4)c2	6.9
378	O=C(C1C(C1(C)C)(C)C)c2c3ccccc3n(CCCC=C)c2	6.9
379	FC(F)(F)COCn1cc(C(=O)C2C(C2(C)C)(C)C)c3ccccc31	6.9
380	FC(F)COn1cc(C(=O)C2C(C2(C)C)(C)C)c3ccccc31	6.9
381	Br/C=C/Cn1cc(C(=O)C2C(C2(C)C)(C)C)c3ccccc31	6.9
382	FC(F)(F)C[N+]CCn1cc(C(=O)C2C(C2(C)C)(C)C)c3ccccc31	6.9
383	O=C(C1C(C1(C)C)(C)C)c2cn([C@@H]([N+])CCCC)c3ccccc32	6.9
384	O=C(C1C(C1(C)C)(C)C)c2c3ccccc3n(CCC=C)c2	6.9
385	Brc1ccc(o1)Cn2cc(C(=O)C3C(C3(C)C)(C)C)c4ccccc42	6.9
386	Clc1cnn(Cn2cc(C(=O)C3C(C3(C)C)(C)C)c4ccccc42)c1	6.9
387	O=C(C1C(C1(C)C)(C)C)c2cn(NCc3cccs3)c4ccccc42	6.9
388	O=C(C1C(C1(C)C)(C)C)c2c3ccccc3n(CC[N+]4[C@@H](C4)C)c2	6.8
389	O=C(C1C(C1(C)C)(C)C)c2cn([C@@H]([N+])CSC)c3ccccc32	6.8
390	O=C(C1C(C1(C)C)(C)C)c2cn(SC[C@@H]3CCOC3)c4ccccc42	6.8
391	FC(Sn1cc(C(=O)C2C(C2(C)C)(C)C)c3ccccc31)(F)C(F)F	6.8
392	O=C(C1C(C1(C)C)(C)C)c2c3ccccc3n(CSC4CCOCC4)c2	6.8
393	SCCOn1cc(C(=O)C2C(C2(C)C)(C)C)c3ccccc31	6.8
394	FC(F)CNS(=O)(=O)n1cc(C(=O)C2C(C2(C)C)(C)C)c3ccccc31	6.8
395	O=C(C1C(C1(C)C)(C)C)c2cn(SC[C@H]([N+])CC)c3ccccc32	6.8
396	Cl/C(Cn1cc(C(=O)C2C(C2(C)C)(C)C)c3ccccc31)=C\Cl	6.8
397	S/C(NCCn1cc(C(=O)C2C(C2(C)C)(C)C)c3ccccc31)=[N+]\C	6.8
398	FC(F)(F)CC[N+]Cn1cc(C(=O)C2C(C2(C)C)(C)C)c3ccccc31	6.8
399	O=C(C1C(C1(C)C)(C)C)c2c3ccccc3n(CC[N+]4CCC[C@@H](C4)C)c2	6.8

400	Cl/C=C/Cn1cc(C(=O)C2C(C2(C)C)(C)C)c3ccccc31	6.8
401	FC(CCn1cc(C(=O)C2C(C2(C)C)(C)C)c3ccccc31)=C(F)F	6.8
402	O=C(C1C(C1(C)C)(C)C)c2c3ccccc3n(C[N+]C(C)(C)C)c2	6.8
403	O=C(C1C(C1(C)C)(C)C)c2c3ccccc3n(CCCC#CC)c2	6.8
404	O=C(C1C(C1(C)C)(C)C)c2c3ccccc3n(CC4=CCCOC4)c2	6.8
405	O=C(C1C(C1(C)C)(C)C)c2cn(c3ccccc32)CCSCC#C	6.8
406	O=C(C1C(C1(C)C)(C)C)c2c3ccccc3n(C[C@@H]4C=CCC4)c2	6.8
407	O=C(C1C(C1(C)C)(C)C)c2cn(NCC#C)c3ccccc32	6.8
408	O=C(C1C(C1(C)C)(C)C)c2c3ccccc3n(C[C@@H]([N+])CC(C)C)c2	6.8
409	FCCCSn1cc(C(=O)C2C(C2(C)C)(C)C)c3ccccc31	6.8
410	O=C(C1C(C1(C)C)(C)C)c2c3ccccc3n(CC[C@H]([N+])C4CC4)c2	6.8
411	O=C(C1C(C1(C)C)(C)C)c2c3ccccc3n(CC[C@H](CCC)C#N)c2	6.8
412	O=C(C1C(C1(C)C)(C)C)c2c3ccccc3n(CCCC(C)(C)C#N)c2	6.8
413	S/C(=[N+]/Cn1cc(C(=O)C2C(C2(C)C)(C)C)c3ccccc31)N	6.7
414	SCCSn1cc(C(=O)C2C(C2(C)C)(C)C)c3ccccc31	6.7
415	O=C(C1C(C1(C)C)(C)C)c2cn(c3ccccc32)/C=C\CCCC	6.7
416	O=C(C1C(C1(C)C)(C)C)c2c3ccccc3n(CC[C@@H](OC)C)c2	6.7
417	FC(F)(F)COn1cc(C(=O)C2C(C2(C)C)(C)C)c3ccccc31	6.7
418	FC(SCn1cc(C(=O)C2C(C2(C)C)(C)C)c3ccccc31)F	6.7
419	FCCSn1cc(C(=O)C2C(C2(C)C)(C)C)c3ccccc31	6.7
420	O=C(C1C(C1(C)C)(C)C)c2c3ccccc3n(CC4CC4)c2	6.7
421	FC(F)C[N+]Cn1cc(C(=O)C2C(C2(C)C)(C)C)c3ccccc31	6.7
422	O[C@H](CCCn1cc(C(=O)C2C(C2(C)C)(C)C)c3ccccc31)C	6.7
423	FCCOCn1cc(C(=O)C2C(C2(C)C)(C)C)c3ccccc31	6.7
424	O=C(C1C(C1(C)C)(C)C)c2cn(SSC(C)C)c3ccccc32	6.7
425	O=C(C1C(C1(C)C)(C)C)c2c3ccccc3n(CSCCC)c2	6.7
426	O=C(C1C(C1(C)C)(C)C)c2cn(SC3CCOCC3)c4ccccc42	6.7
427	O=C(C1C(C1(C)C)(C)C)c2c3ccccc3n(CCC#C)c2	6.7
428	O=C(C1C(C1(C)C)(C)C)c2c3ccccc3n(CC[C@H]4CCC[N+]4)c2	6.7
429	O=C(C1C(C1(C)C)(C)C)c2c3ccccc3n(C[N+][C@H]4CCSC4)c2	6.7
430	Clc1ccc(s1)CNn2cc(C(=O)C3C(C3(C)C)(C)C)c4ccccc42	6.7
431	O=C(C1C(C1(C)C)(C)C)c2c3ccccc3n(COCC#C)c2	6.7
432	SC(=S)NCn1cc(C(=O)C2C(C2(C)C)(C)C)c3ccccc31	6.7
433	FC[C@H](O)Cn1cc(C(=O)C2C(C2(C)C)(C)C)c3ccccc31	6.7
434	O=C(C1C(C1(C)C)(C)C)c2c3ccccc3n(C[N+]C(C)C)c2	6.6
435	O=C(C1C(C1(C)C)(C)C)c2cn(NCCCC#N)c3ccccc32	6.6
436	O=C(C1C(C1(C)C)(C)C)c2cn(SCCCO)c3ccccc32	6.6
437	O=C(C1C(C1(C)C)(C)C)c2c3ccccc3n(C[N+]CC#C)c2	6.6
438	O=C(C1C(C1(C)C)(C)C)c2c3ccccc3n(c2)CC#CC	6.6
439	O=C(C1C(C1(C)C)(C)C)c2c3ccccc3n(CCC#CC)c2	6.6
440	O=C(C1C(C1(C)C)(C)C)c2c3ccccc3n(C[C@H]4CC[N+]4)c2	6.6
441	O=C(C1C(C1(C)C)(C)C)c2c3ccccc3n(CCCC)c2	6.6

442	O=C(C1C(C1(C)C)(C)C)c2c3ccccc3n(C[C@H]4CS4)c2	6.6
443	O=C(C1C(C1(C)C)(C)C)c2c3ccccc3n(CC[N+](CC)C)c2	6.6
444	O=C(C1C(C1(C)C)(C)C)c2c3ccccc3n(CC[N+]C)c2	6.6
445	O=C(C1C(C1(C)C)(C)C)c2c3ccccc3n(CCC[S+](C)C)c2	6.6
446	Fc1cccc(CCn2cc(C(=O)C3C(C3(C)C)(C)C)c4ccccc42)c1	6.6
447	FC(F)CSn1cc(C(=O)C2C(C2(C)C)(C)C)c3ccccc31	6.6
448	O=C(C1C(C1(C)C)(C)C)c2c3ccccc3n(C[N+]CC=4[N-]N=NN4)c2	6.6
449	SC[C@@H]([N+])CCn1cc(C(=O)C2C(C2(C)C)(C)C)c3ccccc31	6.5
450	O=C(C1C(C1(C)C)(C)C)c2cn(SCC#CC)c3ccccc32	6.5
451	O=C(C1C(C1(C)C)(C)C)c2c3ccccc3n(COC/C=C/C)c2	6.5
452	O=C(C1C(C1(C)C)(C)C)c2cn(SCC(C)C)c3ccccc32	6.5
453	O=C(C1C(C1(C)C)(C)C)c2c3ccccc3n(C/C=C\CO)c2	6.5
454	O=C(C1C(C1(C)C)(C)C)c2c3ccccc3n(CSCC)c2	6.5
455	O=C(C1C(C1(C)C)(C)C)c2c3ccccc3n(C[N+][C@H]4[C@@H](C4)C)c2	6.5
456	O=C(C1C(C1(C)C)(C)C)c2c3ccccc3n(CCc4ccoc4)c2	6.5
457	O=C(C1C(C1(C)C)(C)C)c2c3ccccc3n(CCCC#N)c2	6.4
458	O=C(C1C(C1(C)C)(C)C)c2c3ccccc3n(C[N+]CCSC)c2	6.4
459	O=C(C1C(C1(C)C)(C)C)c2c3ccccc3n(C[S@](=O)CCC)c2	6.4
460	O=C(C1C(C1(C)C)(C)C)c2c3ccccc3n(CC[N+]4CCCC4)c2	6.4
461	O=C(C1C(C1(C)C)(C)C)c2c3ccccc3n(c2)CC#CCC	6.4
462	O=C(C1C(C1(C)C)(C)C)c2cn(NCC)c3ccccc32	6.4
463	O=C(C1C(C1(C)C)(C)C)c2c3ccccc3n(C[N+]CC(C)=C)c2	6.4
464	SCCCn1cc(C(=O)C2C(C2(C)C)(C)C)c3ccccc31	6.4
465	SCC[N+]Cn1cc(C(=O)C2C(C2(C)C)(C)C)c3ccccc31	6.3
466	ClC(Cl)Cn1cc(C(=O)C2C(C2(C)C)(C)C)c3ccccc31	6.3
467	O=C(C1C(C1(C)C)(C)C)c2cn(c3ccccc32)[C@@H](O)CC#C	6.3
468	O=C(C1C(C1(C)C)(C)C)c2c3ccccc3n(C[N+]CC#CC)c2	6.3
469	O=C(C1C(C1(C)C)(C)C)c2c3ccccc3n(CCC4CCOCC4)c2	6.3
470	FC(Sn1cc(C(=O)C2C(C2(C)C)(C)C)c3ccccc31)F	6.3
471	FC(Cn1cc(C(=O)C2C(C2(C)C)(C)C)c3ccccc31)=C	6.3
472	SCCn1cc(C(=O)C2C(C2(C)C)(C)C)c3ccccc31	6.3
473	O=C(C1C(C1(C)C)(C)C)c2c3ccccc3n(CSC)c2	6.3
474	O=C(C1C(C1(C)C)(C)C)c2c3ccccc3n(CC[N+]CC)c2	6.2
475	O=C(C1C(C1(C)C)(C)C)c2c3ccccc3n(CCCSC(N)=[N+])c2	6.2
476	O=C(C1C(C1(C)C)(C)C)c2c3ccccc3n(CSCC=C)c2	6.2
477	FC(F)(F)Cn1cc(C(=O)C2C(C2(C)C)(C)C)c3ccccc31	6.2
478	O=C(C1C(C1(C)C)(C)C)c2cn(NCc3ccco3)c4ccccc42	6.2
479	O=C(C1C(C1(C)C)(C)C)c2c3ccccc3n(COC)c2	6.2
480	O=C(C1C(C1(C)C)(C)C)c2cn(SCCOC)c3ccccc32	6.2
481	S=C(N)CCSn1cc(C(=O)C2C(C2(C)C)(C)C)c3ccccc31	6.2
482	O=C(C1C(C1(C)C)(C)C)c2cn([C@@H]([N+])CC#C)c3ccccc32	6.1
483	O=C(C1C(C1(C)C)(C)C)c2c3ccccc3n(CCC)c2	6.1

484	O=C(C1C(C1(C)C)(C)C)c2c3ccccc3n(C[N+]CCC)c2	6.1
485	O=C(C1C(C1(C)C)(C)C)c2c3ccccc3n([C@@H]([N+])C4CC4)c2	6.1
486	FC(Sn1cc(C(=O)C2C(C2(C)C)(C)C)c3ccccc31)(F)F	6
487	O=C(C1C(C1(C)C)(C)C)c2cn(SCC#CCO)c3ccccc32	6
488	O=C(C1C(C1(C)C)(C)C)c2c3ccccc3n(CCCO)c2	6
489	FCC[N+]Cn1cc(C(=O)C2C(C2(C)C)(C)C)c3ccccc31	6
490	O=C(C1C(C1(C)C)(C)C)c2c3ccccc3n(C[N+]CC)c2	6
491	O=C(C1C(C1(C)C)(C)C)c2cn(SCC#C)c3ccccc32	6
492	O=C(C1C(C1(C)C)(C)C)c2c3ccccc3n(c2)CC#C	6
493	O=C(C1C(C1(C)C)(C)C)c2c3ccccc3n(C[N+]4CC4)c2	5.9
494	O=C(C1C(C1(C)C)(C)C)c2c3ccccc3n([C@@H]([N+])CC)c2	5.8
495	O=C(C1C(C1(C)C)(C)C)c2cn(SSCC[N+])c3ccccc32	5.8
496	FCSn1cc(C(=O)C2C(C2(C)C)(C)C)c3ccccc31	5.7
497	FC(F)Cn1cc(C(=O)C2C(C2(C)C)(C)C)c3ccccc31	5.7
498	FCCn1cc(C(=O)C2C(C2(C)C)(C)C)c3ccccc31	5.7
499	SCn1cc(C(=O)C2C(C2(C)C)(C)C)c3ccccc31	5.5
500	O=C(C1C(C1(C)C)(C)C)c2c3ccccc3n(C[N+]C)c2	5.3

Table S9. List, SMILE and predicted pK_i values for Series 5 in CB_1 receptor.

N°	SMILES	Pred pK_i
1	FC(F)(F)CCCc1c(N)c(C(=O)C2C(C2(C)C)(C)C)c3ccccn31	8
2	FC(F)(F)CCCc1c(c(C(=O)C2C(C2(C)C)(C)C)c3ccccn13)C	8
3	FC(F)(F)CCCn1cc(C(=O)C2C(C2(C)C)(C)C)c3ccc(cc31)C(=O)N	8
4	FC(F)(F)CCCc1nc(C(=O)C2C(C2(C)C)(C)C)c3ccncn31	8
5	Brc1c(C(=O)C2C(C2(C)C)(C)C)cc(n1C)CCCC(F)(F)F	7.9
6	FC(F)(F)CCCc1c(N)c(cc(C(=O)C2C(C2(C)C)(C)C)c1)CC	7.9
7	Brc1cc(C(=O)C2C(C2(C)C)(C)C)cc(CCCC(F)(F)F)c1N	7.9
8	FC(F)(F)CCCn1c2CCOCc2c(n1)C(=O)C3C(C3(C)C)(C)C	7.9
9	Clc1cc(N)c(CCCC(F)(F)F)cc1C(=O)C2C(C2(C)C)(C)C	7.9
10	FC(F)(F)CCCc1cc(F)c(c(C(=O)C2C(C2(C)C)(C)C)c1)C=O	7.9
11	FC(F)(F)CCCc1cc(C(=O)C2C(C2(C)C)(C)C)c(n1CC(F)(F)F)C	7.9
12	FC(F)(F)CCCOc1ccc(F)cc1CC(=O)C2C(C2(C)C)(C)C	7.9
13	FC(F)(F)CCCc1cc(C(=O)C2C(C2(C)C)(C)C)c3C(SC=Cn31)=O	7.9
14	FC(F)(F)CCCc1cc(C(=O)C2C(C2(C)C)(C)C)cc(c1N)C	7.8
15	FC(F)(F)CCCc1cc(c2CCCn12)C(=O)C3C(C3(C)C)(C)C	7.8
16	Brc1cc(CCCC(F)(F)F)cc(C(=O)C2C(C2(C)C)(C)C)c1OC	7.8
17	FC(F)(F)CCCn1cc(C(=O)C2C(C2(C)C)(C)C)c3ccc(OC)cc31	7.8
18	FC(F)(F)CCCc1c2N=CSC(=O)n2c(C(=O)C3C(C3(C)C)(C)C)c1	7.8
19	FC(F)(F)CCCc1c(sc(C(=O)C2C(C2(C)C)(C)C)c1)N	7.8
20	FC(F)(F)CCCc1cc(C(=O)C2C(C2(C)C)(C)C)c3C(OC=Cn31)=O	7.8
21	FC(F)(F)CCCc1c(c(c([nH]1)C(=O)C2C(C2(C)C)(C)C)C(=O)C)CC	7.8

22	FC(F)(F)CCCc1cc(C(=O)C2C(C2(C)C)(C)C)c3ccncn31	7.8
23	FC(F)(F)CCCn1cc(C(=O)C2C(C2(C)C)(C)C)c3c(F)cccc31	7.8
24	Clc1ccn2c(c(C(=O)C3C(C3(C)C)(C)C)cc2CCCC(F)(F)F)c1	7.8
25	FC(F)(F)CCCn1c2CCCCc2c(n1)C(=O)C3C(C3(C)C)(C)C	7.8
26	Fc1c(cc(CCCC(F)(F)F)cc1C(=O)C2C(C2(C)C)(C)C)C(F)(F)F	7.8
27	FC(F)(F)CCCc1c(c(c([nH]1)C(=O)C2C(C2(C)C)(C)C)C(=O)C)C	7.8
28	FC(F)(F)CCCc1c2c(c(s1)C(=O)C3C(C3(C)C)(C)C)CCC(C2)(C)C	7.8
29	FC(F)(F)CCCn1c2ccccc2c(n1)C(=O)C3C(C3(C)C)(C)C	7.8
30	FC(F)(F)CCCc1cc(C(=O)C2C(C2(C)C)(C)C)c3ccsn31	7.8
31	FC(F)(F)CCCc1cc(C(=O)C2C(C2(C)C)(C)C)c3ccccn31	7.8
32	FC(F)(F)CCCc1c2C=COC(=O)n2c(C(=O)C3C(C3(C)C)(C)C)c1	7.8
33	FC(F)(F)CCCn1c2CCCc2c(n1)C(=O)C3C(C3(C)C)(C)C	7.8
34	FC(F)(F)CCCc1c(N)cc(F)c(C(=O)C2C(C2(C)C)(C)C)c1	7.8
35	FC(F)(F)CCCc1cc(C(=O)C2C(C2(C)C)(C)C)c3C(=O)NC=Cn31	7.8
36	FC(F)(F)CCCn1c2cc(F)ccc2c(n1)C(=O)C3C(C3(C)C)(C)C	7.8
37	FC(F)(F)CCCn1c2C=CSC(=O)c2c(n1)C(=O)C3C(C3(C)C)(C)C	7.8
38	FC(F)(F)CCCc1c2C=CSC(=O)n2c(C(=O)C3C(C3(C)C)(C)C)c1	7.8
39	FC(F)(F)CCCc1c2C=CSC(=O)c2n(n1)C(=O)C3C(C3(C)C)(C)C	7.8
40	FC(F)(F)CCCn1cc(C(=O)C2C(C2(C)C)(C)C)c3ccc(O)cc31	7.7
41	FC(F)(F)CCCn1c2C=CSC(=O)c2c(C(=O)C3C(C3(C)C)(C)C)c1	7.7
42	FC(F)(F)CCCc1cc(C(=O)C2C(C2(C)C)(C)C)c3n1ccs3	7.7
43	Clc1cccc2c1c(nn2CCCC(F)(F)F)C(=O)C3C(C3(C)C)(C)C	7.7
44	FC(F)(F)CCCn1c2c(c(C(=O)C3C(C3(C)C)(C)C)c1)C(=O)NC(S2)=O	7.7
45	FC(F)(F)CCCc1cc2COC(=O)c2c(C(=O)C3C(C3(C)C)(C)C)c1	7.7
46	FC(F)(F)CCCc1c2C=CCCc2c(s1)C(=O)C3C(C3(C)C)(C)C	7.7
47	FC(F)(F)CCCc1cc(C(=O)C2C(C2(C)C)(C)C)c3C(SC=Nn31)=O	7.7
48	FC(F)(F)CCCc1c2C=CSC(=O)c2c(s1)C(=O)C3C(C3(C)C)(C)C	7.7
49	FC(F)(F)CCCc1cc(C(=O)C2C(C2(C)C)(C)C)cn1C	7.7
50	Clc1ccc2c(n(nc2C(=O)C3C(C3(C)C)(C)C)CCCC(F)(F)F)c1	7.7
51	FC(F)(F)CCCc1cc(C(=O)C2C(C2(C)C)(C)C)c(n1C)C	7.7
52	FC(F)(F)CCCc1c(O)c(cc(C(=O)C2C(C2(C)C)(C)C)c1)CC	7.7
53	FC(F)(F)CCCN1C2=CSC(=O)N2C(C(=O)C3C(C3(C)C)(C)C)=C1	7.7
54	FC(F)(F)CCCc1cc(n2c1cns2)C(=O)C3C(C3(C)C)(C)C	7.7
55	FC(F)(F)CCCc1c2C=CNC(=O)c2c(o1)C(=O)C3C(C3(C)C)(C)C	7.7
56	FC(F)(F)CCCc1c2C=COC(=O)c2c(s1)C(=O)C3C(C3(C)C)(C)C	7.7
57	FC(F)(F)CCCc1c2ccccc2c(s1)C(=O)C3C(C3(C)C)(C)C	7.7
58	FC(F)(F)CCCc1cc(C(=O)C2C(C2(C)C)(C)C)c3cnccn31	7.7
59	Clc1cc(C(=O)C2C(C2(C)C)(C)C)cc(CCCC(F)(F)F)c1N	7.7
60	FC(F)(F)CCCn1cc(c2C(=O)CC(Cc21)(C)C)C(=O)C3C(C3(C)C)(C)C	7.7
61	FC(F)(F)CCCc1cc(C(=O)C2C(C2(C)C)(C)C)c3cc(ccn13)C	7.7
62	FC(F)(F)CCCc1c(c(c(o1)C(=O)C2C(C2(C)C)(C)C)C)C	7.7
63	Clc1cccc2c1c(C(=O)C3C(C3(C)C)(C)C)cn2CCCC(F)(F)F	7.7

64	FC(F)(F)CCCn1c2ccsc2c(n1)C(=O)C3C(C3(C)C)(C)C	7.7
65	FC(F)(F)CCCn1cc(C(=O)C2C(C2(C)C)(C)C)c3c(OC)cccc31	7.7
66	FC(F)(F)CCCc1nc(C(=O)C2C(C2(C)C)(C)C)cc3cccn31	7.7
67	FC(F)(F)CCCn1c2c(c(C(=O)C3C(C3(C)C)(C)C)c1)C(SC=N2)=O	7.7
68	Brc1c(sc(CCCC(F)(F)F)c1C)C(=O)C2C(C2(C)C)(C)C	7.7
69	Clc1ccc2c(c(nn2CCCC(F)(F)F)C(=O)C3C(C3(C)C)(C)C)c1	7.7
70	FC(F)(F)CCCc1cc(N)c(F)c(C(=O)C2C(C2(C)C)(C)C)c1	7.7
71	FC(F)(F)CCCc1cc(C(=O)C2C(C2(C)C)(C)C)cn1CC3CC3	7.7
72	FC(F)(F)CCCn1c2cnsc2c(n1)C(=O)C3C(C3(C)C)(C)C	7.7
73	FC(F)(F)CCCn1c2CCCC(=O)c2c(n1)C(=O)C3C(C3(C)C)(C)C	7.7
74	FC(F)(F)CCCc1cc(C(=O)C2C(C2(C)C)(C)C)cc3CCCNc31	7.7
75	FC(F)(F)CCCn1c2cocc2c(n1)C(=O)C3C(C3(C)C)(C)C	7.7
76	FC(F)(F)CCCc1c2C=CSC(=O)c2c(o1)C(=O)C3C(C3(C)C)(C)C	7.7
77	FC(F)(F)CCCn1c2C=COC(=O)c2c(C(=O)C3C(C3(C)C)(C)C)c1	7.7
78	FC(F)(F)CCCn1cc(C(=O)C2C(C2(C)C)(C)C)c3cc(OC(C)C)ccc31	7.7
79	FC(F)(F)CCCc1c(c(c([nH]1)C(=O)C2C(C2(C)C)(C)C)CC)C	7.6
80	FC(F)(F)CCCn1c2cnccc2c(n1)C(=O)C3C(C3(C)C)(C)C	7.6
81	FC(F)(F)CCCn1c2cscc2c(n1)C(=O)C3C(C3(C)C)(C)C	7.6
82	FC(F)(F)CCCc1cc(F)c(c(C(=O)C2C(C2(C)C)(C)C)c1)C#N	7.6
83	FC(F)(F)CCCc1c2ccccc2cc(C(=O)C3C(C3(C)C)(C)C)c1	7.6
84	Clc1c(Cl)c(O)c(CCCC(F)(F)F)cc1C(=O)C2C(C2(C)C)(C)C	7.6
85	FC(F)(F)CCCc1cc(C(=O)C2C(C2(C)C)(C)C)c3n1cns3	7.6
86	FC(F)(F)CCCn1c2cc(F)ccc2c(C(=O)C3C(C3(C)C)(C)C)c1C	7.6
87	Brc1cc(C(=O)C2C(C2(C)C)(C)C)cc(CCCC(F)(F)F)c1NC	7.6
88	FC(F)(F)CCCn1cc(C(=O)C2C(C2(C)C)(C)C)c3CCCc31	7.6
89	Brc1c(nn(CCCC(F)(F)F)c1C)C(=O)C2C(C2(C)C)(C)C	7.6
90	FC(F)(F)CCCn1c2ccc(cc2c(n1)C(=O)C3C(C3(C)C)(C)C)C	7.6
91	FC(F)(F)CCCn1c2c(c(n1)C(=O)C3C(C3(C)C)(C)C)ccs2	7.6
92	FC(F)(F)CCCc1cc(C(=O)C2C(C2(C)C)(C)C)cc3CCNc31	7.6
93	FC(F)(F)CCCc1c2CCCC(=O)c2c(s1)C(=O)C3C(C3(C)C)(C)C	7.6
94	FC(F)(F)CCCn1c2c(c(C(=O)C3C(C3(C)C)(C)C)c1)c(OC)ncn2	7.6
95	FC(F)(F)CCCn1c(c(C(=O)C2C(C2(C)C)(C)C)c3ccccc31)C	7.6
96	FC(F)(F)CCCn1cc(C(=O)C2C(C2(C)C)(C)C)c3c[nH]cc31	7.6
97	FC(F)(F)CCCn1cc(C(=O)C2C(C2(C)C)(C)C)c3cc(OCC)ccc31	7.6
98	FC(F)(F)CCCn1cc(C(=O)C2C(C2(C)C)(C)C)c3cccc(O)c31	7.6
99	FC(F)(F)CCCc1c(N)ccc(C(=O)C2C(C2(C)C)(C)C)c1	7.6
100	FC(F)(F)CCCn1cc(C(=O)C2C(C2(C)C)(C)C)c3c(cccc31)C(OC)=O	7.6
101	FC(F)(F)CCCn1c2c(c(n1)C(=O)C3C(C3(C)C)(C)C)C(=O)C=CS2	7.6
102	FC(F)(F)CCCc1c2ccccc2c(o1)C(=O)C3C(C3(C)C)(C)C	7.6
103	FC(F)(F)CCCc1cc(C(=O)C2C(C2(C)C)(C)C)c(s1)C(=O)C	7.6
104	FC(F)(F)CCCc1cc(C(=O)C2C(C2(C)C)(C)C)cc3cc[nH]c31	7.6
105	Clc1c(C(=O)C2C(C2(C)C)(C)C)cc(n1C)CCCC(F)(F)F	7.6

106	Brc1c(O)c(C(=O)C2C(C2(C)C)(C)C)cc(CCCC(F)(F)F)c1O	7.6
107	FC(F)(F)CCCc1c2c(c([nH]1)C(=O)C3C(C3(C)C)(C)C)cc[nH]2	7.6
108	FC(F)(F)CCCn1c2cccc(F)c2c(n1)C(=O)C3C(C3(C)C)(C)C	7.6
109	FC(F)(F)CCCn1cc(C(=O)C2C(C2(C)C)(C)C)c(n1)C(F)F	7.6
110	FC(F)(F)CCCn1cc(C(=O)C2C(C2(C)C)(C)C)c3c1NC(S3)=O	7.6
111	FC(F)(F)CCCn1c2cc(OC)ccc2c(n1)C(=O)C3C(C3(C)C)(C)C	7.5
112	FC(F)(F)CCCc1cc(C(=O)C2C(C2(C)C)(C)C)cc3CCCc31	7.5
113	FC(F)(F)CCCc1cc([N+]([O-])=O)c(O)c(C(=O)C2C(C2(C)C)(C)C)c1	7.5
114	FC(F)(F)CCCc1nc(C(=O)C2C(C2(C)C)(C)C)c3n1ccs3	7.5
115	FC(F)(F)CCCc1c(O)c(cc(C(=O)C2C(C2(C)C)(C)C)c1)C(C)(C)C	7.5
116	Clc1c(cc(CCCC(F)(F)F)cc1C(=O)C2C(C2(C)C)(C)C)C(F)(F)F	7.5
117	FC(F)(F)CCCc1c2C=CC(=O)Nc2c(s1)C(=O)C3C(C3(C)C)(C)C	7.5
118	FC(F)(F)CCCn1cc(C(=O)C2C(C2(C)C)(C)C)c3ccc(cc31)C	7.5
119	FC(F)(F)CCCc1c2ccsc2c(s1)C(=O)C3C(C3(C)C)(C)C	7.5
120	FC(F)(F)CCCc1c2C=CSC(=O)c2n(C(=O)C3C(C3(C)C)(C)C)c1	7.5
121	FC(F)(F)CCCc1cc(C(=O)C2C(C2(C)C)(C)C)c(n1CC)C	7.5
122	FC(F)(F)CCCc1cc2c(c(C(=O)C3C(C3(C)C)(C)C)c1)cc[nH]2	7.5
123	FC(F)(F)CCCn1cc(C(=O)C2C(C2(C)C)(C)C)c3cc(OC)c(OC)cc31	7.5
124	FC(F)(F)CCCn1cc(C(=O)C2C(C2(C)C)(C)C)c3cc(F)ccc31	7.5
125	FC(F)(F)CCCc1c2n(c(C(=O)C3C(C3(C)C)(C)C)c1)C(=O)C=CS2	7.5
126	FC(F)(F)CCCc1cc2c(OC(S2)=O)c(C(=O)C3C(C3(C)C)(C)C)c1	7.5
127	FC(F)(F)CCCc1cc2c(nco2)c(C(=O)C3C(C3(C)C)(C)C)c1	7.5
128	Clc1ccc(CCCC(F)(F)F)cc1C(=O)C2C(C2(C)C)(C)C	7.5
129	Clc1c(Cl)c(nn1CCCC(F)(F)F)C(=O)C2C(C2(C)C)(C)C	7.5
130	FC(F)(F)CCCn1c2ccc(cc2c(C(=O)C3C(C3(C)C)(C)C)c1)C#N	7.5
131	FC(F)(F)CCCc1c2c(c([nH]1)C(=O)C3C(C3(C)C)(C)C)C(SC=N2)=O	7.5
132	FC(F)(F)CCCc1cc2ccoc2c(C(=O)C3C(C3(C)C)(C)C)c1	7.5
133	FC(F)(F)c1cc(CCCC(F)(F)F)cc(C(=O)C2C(C2(C)C)(C)C)c1O	7.5
134	FC(F)(F)CCCc1c2C=CSC(=O)c2c([nH]1)C(=O)C3C(C3(C)C)(C)C	7.5
135	FC(F)(F)CCCc1cn(C(=O)C2C(C2(C)C)(C)C)c3C(=O)NC=Cc31	7.5
136	FC(F)(F)CCCc1cc(C(=O)C2C(C2(C)C)(C)C)cc(c1)C(F)(F)F	7.5
137	FC(F)(F)CCCc1c2ccccc2c([nH]1)C(=O)C3C(C3(C)C)(C)C	7.5
138	Brc1cc(CCCC(F)(F)F)cc(C(=O)C2C(C2(C)C)(C)C)c1Cl	7.5
139	FC(F)(F)CCCc1c2C=COC(=O)c2c(o1)C(=O)C3C(C3(C)C)(C)C	7.5
140	FC(F)(F)CCCn1c2c(c(C(=O)C3C(C3(C)C)(C)C)c1)C(OC=N2)=O	7.5
141	FC(F)(F)CCCn1cc(C(=O)C2C(C2(C)C)(C)C)c3c1csn3	7.5
142	FC(F)(F)CCCn1cc(sn2n1ccs2)C(=O)C3C(C3(C)C)(C)C	7.5
143	Clc1c(O)c(C(=O)C2C(C2(C)C)(C)C)cc(CCCC(F)(F)F)c1O	7.5
144	FC(F)(F)CCCn1c2c(SC(S2)=O)c(C(=O)C3C(C3(C)C)(C)C)c1	7.5
145	FC(F)(F)CCCc1cc(C(=O)C2C(C2(C)C)(C)C)c3n1ncs3	7.5
146	FC(F)(F)CCCc1c2c(c(o1)C(=O)C3C(C3(C)C)(C)C)cco2	7.5
147	FC(F)(F)CCCc1c2CCCCc2c([nH]1)C(=O)C3C(C3(C)C)(C)C	7.5

148	FC(F)(F)CCCc1c2c(c(o1)C(=O)C3C(C3(C)C)(C)C)C(=O)C=CS2	7.5
149	FC(F)(F)CCCc1cc(C(=O)C2C(C2(C)C)(C)C)c3ccon31	7.5
150	FC(F)(F)CCCc1c(O)cc(F)c(C(=O)C2C(C2(C)C)(C)C)c1	7.5
151	FC(F)(F)CCCc1cc(C(=O)C2C(C2(C)C)(C)C)c(n1CC=C)C	7.5
152	FC(F)(F)CCCc1cc(F)c(F)c(C(=O)C2C(C2(C)C)(C)C)c1	7.5
153	Clc1c(CC)cc(CCCC(F)(F)F)cc1C(=O)C2C(C2(C)C)(C)C	7.5
154	FC(F)(F)CCCc1c2C=CNC(=O)c2c([nH]1)C(=O)C3C(C3(C)C)(C)C	7.5
155	Sc1c2c(n(CCCC(F)(F)F)cc2C(=O)C3C(C3(C)C)(C)C)ncn1	7.5
156	FC(F)(F)CCCc1cc2c(OCC2)c(C(=O)C3C(C3(C)C)(C)C)c1	7.5
157	Clc1cc(CCCC(F)(F)F)cc(C(=O)C2C(C2(C)C)(C)C)c1F	7.5
158	FC(F)(F)CCCc1cc(c(O)c(C(=O)C2C(C2(C)C)(C)C)c1)C	7.5
159	FC(F)(F)CCCn1cc(C(=O)C2C(C2(C)C)(C)C)c3c(cccc31)C	7.5
160	FC(F)(F)CCCc1c2c(c(o1)C(=O)C3C(C3(C)C)(C)C)cns2	7.5
161	FC(F)(F)CCCc1c2c([C@H]3CC[C@@H]2C3)c([nH]1)C(=O)C4C(C4(C)C)(C)C	7.5
162	Fc1c(cc(CCCC(F)(F)F)cc1C(=O)C2C(C2(C)C)(C)C)CO	7.5
163	FC(F)(F)CCCc1c(O)c(F)c(F)c(C(=O)C2C(C2(C)C)(C)C)c1	7.5
164	FC(F)(F)CCCN1C=C(C(=O)C2C(C2(C)C)(C)C)CCC1	7.5
165	FC(F)(F)CCCc1c(NC)ccc(C(=O)C2C(C2(C)C)(C)C)c1	7.5
166	FC(F)(F)CCCc1cc([n+]([O-])c2ccccc21)C(=O)C3C(C3(C)C)(C)C	7.5
167	FC(F)(F)CCCN1c2ccccc2N(C(=O)C3C(C3(C)C)(C)C)C1=N	7.5
168	FC(F)(F)CCCc1cc(C(=O)C2C(C2(C)C)(C)C)cc(c1)C	7.5
169	FC(F)(F)CCCn1c2c(c(C(=O)C3C(C3(C)C)(C)C)c1)C(=O)C=NO2	7.5
170	Sc1ccc(CCCC(F)(F)F)cc1C(=O)C2C(C2(C)C)(C)C	7.5
171	FC(F)(F)CCCn1cc(C(=O)C2C(C2(C)C)(C)C)c3cc[nH]c31	7.5
172	FC(F)(F)CCCn1cc(C(=O)C2C(C2(C)C)(C)C)c3ccccc31	7.5
173	FC(F)(F)CCCc1cc(cc(C(=O)C2C(C2(C)C)(C)C)c1N)C	7.5
174	FC(F)(F)CCCc1cc2c(OC(=O)C=N2)c(C(=O)C3C(C3(C)C)(C)C)c1	7.5
175	FC(F)(F)CCCc1c(O)c(N)cc(C(=O)C2C(C2(C)C)(C)C)c1	7.5
176	FC(F)(F)CCCc1cc(C(=O)C2C(C2(C)C)(C)C)cc3c1[nH]cc3CC	7.5
177	FC(F)(F)CCCn1c2cc(O)ccc2c(n1)C(=O)C3C(C3(C)C)(C)C	7.5
178	FC(F)(F)CCCn1c2ccc([N+]([O-])=O)cc2c(n1)C(=O)C3C(C3(C)C)(C)C	7.5
179	FC(F)(F)CCCc1cc2csnc2c(C(=O)C3C(C3(C)C)(C)C)c1	7.5
180	FC(F)(F)CCCn1cc(C(=O)C2C(C2(C)C)(C)C)c3ccc(F)cc31	7.5
181	FC(F)(F)CCCc1cc(sc1CCC)C(=O)C2C(C2(C)C)(C)C	7.4
182	FC(F)(F)CCCn1cc(C(=O)C2C(C2(C)C)(C)C)c3c1ccs3	7.4
183	FC(F)(F)CCCn1cc(C(=O)C2C(C2(C)C)(C)C)cc1C	7.4
184	FC(F)(F)CCCc1ccc(F)c(C(=O)C2C(C2(C)C)(C)C)c1	7.4
185	FC(F)(F)CCCn1cc(C(=O)C2C(C2(C)C)(C)C)c3cc([N+]([O-])=O)ccc31	7.4
186	FC(F)(F)CCCc1cc2c(ncs2)c(C(=O)C3C(C3(C)C)(C)C)c1	7.4
187	Clc1c(nn(CCCC(F)(F)F)c1C)C(=O)C2C(C2(C)C)(C)C	7.4
188	Brc1cc(CCCC(F)(F)F)c(N)c(C(=O)C2C(C2(C)C)(C)C)c1	7.4
189	FC(F)(F)CCCc1cc(C(=O)C2C(C2(C)C)(C)C)cc(c1)CO	7.4

190	FC(F)(F)CCCc1ccc(c(C(=O)C2C(C2(C)C)(C)C)c1)C#N	7.4
191	FC(F)(F)CCCc1c2c(c(s1)C(=O)C3C(C3(C)C)(C)C)cns2	7.4
192	FC(F)(F)CCCn1cc(C(=O)C2C(C2(C)C)(C)C)c3c1cco3	7.4
193	Brc1cc(CCCC(F)(F)F)cc(C(=O)C2C(C2(C)C)(C)C)c1O	7.4
194	Clc1ccc2c(c(C(=O)C3C(C3(C)C)(C)C)cn2CCCC(F)(F)F)c1	7.4
195	FC(F)(F)CCCN1C=CCC(C(=O)C2C(C2(C)C)(C)C)=C1	7.4
196	Brc1cc([nH]c1C(=O)C2C(C2(C)C)(C)C)CCCC(F)(F)F	7.4
197	FC(F)(F)CCCn1cc(C(=O)C2C(C2(C)C)(C)C)c(n1)C(=O)C	7.4
198	FC(F)(F)CCCn1cc2-n(oncs2)c(C(=O)C3C(C3(C)C)(C)C)c1	7.4
199	FC(F)(F)CCCn1c2c(c(C(=O)C3C(C3(C)C)(C)C)c1)C(=O)C=CS2	7.4
200	FC(F)(F)CCCc1cc(C(=O)C2C(C2(C)C)(C)C)cc(c1C)C	7.4
201	FC(F)(F)CCCn1cc(C(=O)C2C(C2(C)C)(C)C)c3ccc(cc31)C(F)(F)F	7.4
202	FC(F)(F)CCCc1cc(C(=O)C2C(C2(C)C)(C)C)cc3cocc31	7.4
203	Clc1cc(C(=O)C2C(C2(C)C)(C)C)cc(CCCC(F)(F)F)c1NC	7.4
204	FC(F)(F)CCCn1c2cc(ccc2c(C(=O)C3C(C3(C)C)(C)C)c1C)C	7.4
205	FC(F)(F)CCCc1cc(C(=O)C2C(C2(C)C)(C)C)cc(c1O)C(C)C	7.4
206	Fc1c(cc(CCCC(F)(F)F)cc1C(=O)C2C(C2(C)C)(C)C)C	7.4
207	FC(F)(F)CCCc1c2ccnnn2c(C(=O)C3C(C3(C)C)(C)C)c1	7.4
208	FC(F)(F)CCCn1c(N)c(c(C(=O)C2C(C2(C)C)(C)C)c1)C#N	7.4
209	FC(F)(F)CCCc1c(c(c(s1)C(=O)C2C(C2(C)C)(C)C)C)C	7.4
210	FC(F)(F)CCCn1c2ccc(F)cc2c(C(=O)C3C(C3(C)C)(C)C)c1C	7.4
211	FC(F)(F)CCCn1c2c(c(C(=O)C3C(C3(C)C)(C)C)c1C)ccs2	7.4
212	FC(F)(F)CCCc1c2cnccc2c(s1)C(=O)C3C(C3(C)C)(C)C	7.4
213	FC(F)(F)CCCc1cc(C(=O)C2C(C2(C)C)(C)C)cc3c1[nH]cc3C	7.4
214	Clc1c(C(=O)C2C(C2(C)C)(C)C)c3ccc(Cl)cc3n1CCCC(F)(F)F	7.4
215	FC(F)(F)CCCn1c2c(SC(O2)=O)c(C(=O)C3C(C3(C)C)(C)C)c1	7.4
216	FC(F)(F)CCCc1c2ccsc2c([nH]1)C(=O)C3C(C3(C)C)(C)C	7.4
217	FC(F)(F)CCCc1cc2c(nnnn2)c(C(=O)C3C(C3(C)C)(C)C)c1	7.4
218	FC(F)(F)CCCc1c2C=COC(=O)c2n(C(=O)C3C(C3(C)C)(C)C)c1	7.4
219	FC(F)(F)CCCc1cc(cc(C(=O)C2C(C2(C)C)(C)C)c1)C#N	7.4
220	FC(F)(F)CCCc1cc(C(=O)C2C(C2(C)C)(C)C)cc3c1csn3	7.4
221	FC(F)(F)CCCn1c2cc(cc(c2c(C(=O)C3C(C3(C)C)(C)C)c1)C)C	7.4
222	FC(F)(F)CCCn1nc(C(=O)C2C(C2(C)C)(C)C)c3cccn31	7.4
223	FC(F)(F)CCCn1c2c(c(C(=O)C3C(C3(C)C)(C)C)c1)c(O)nc(n2)N	7.4
224	FC(F)(F)CCCc1cc2ccsc2c(C(=O)C3C(C3(C)C)(C)C)c1	7.4
225	Clc1c2c(n(CCCC(F)(F)F)cc2C(=O)C3C(C3(C)C)(C)C)ncn1	7.4
226	FC(F)(F)CCCn1cc(C(=O)C2C(C2(C)C)(C)C)c3cc(OC)ccc31	7.4
227	Clc1c(Cl)cc(CCCC(F)(F)F)cc1C(=O)C2C(C2(C)C)(C)C	7.4
228	FC(F)(F)CCCc1cc(C(=O)C2C(C2(C)C)(C)C)c3cccc(n31)C	7.4
229	FC(F)(F)CCCc1c(O)c(O)c(O)c(C(=O)C2C(C2(C)C)(C)C)c1	7.4
230	Clc1cc(CCCC(F)(F)F)cc(C(=O)C2C(C2(C)C)(C)C)c1O	7.4
231	FC(F)(F)CCCn1cc(C(=O)C2C(C2(C)C)(C)C)c3ccncc31	7.4

232	FC(F)(F)CCCn1cc(C(=O)C2C(C2(C)C)(C)C)c3c1cccn3	7.4
233	FC(F)(F)CCCc1cc(C(C)C)cc(C(=O)C2C(C2(C)C)(C)C)c1	7.4
234	FC(F)(F)CCCc1cc2ccnnc2c(C(=O)C3C(C3(C)C)(C)C)c1	7.4
235	FC(F)(F)CCCc1cc(cc(C(=O)C2C(C2(C)C)(C)C)c1)CC	7.4
236	Clc1ccc2c(n(CCCC(F)(F)F)cc2C(=O)C3C(C3(C)C)(C)C)c1	7.4
237	FC(F)(F)CCCc1cc(C(=O)C2C(C2(C)C)(C)C)cn1C(C)C	7.4
238	FC(F)(F)CCCc1cc(F)c(O)c(C(=O)C2C(C2(C)C)(C)C)c1	7.4
239	FC(F)(F)CCCc1c2c(c(s1)C(=O)C3C(C3(C)C)(C)C)C(=O)C=CS2	7.4
240	FC(F)(F)CCCn1cc(C(=O)C2C(C2(C)C)(C)C)c3ccc(nc31)C	7.4
241	FC(F)(F)CCCc1cc(sc1CO)C(=O)C2C(C2(C)C)(C)C	7.4
242	FC(F)(F)CCCc1cc(n2cc[nH]c12)C(=O)C3C(C3(C)C)(C)C	7.4
243	FC(F)(F)CCCn1cc(C(=O)C2C(C2(C)C)(C)C)c3cscc31	7.4
244	Clc1c(F)cc(CCCC(F)(F)F)cc1C(=O)C2C(C2(C)C)(C)C	7.3
245	FC(F)(F)CCCc1cc(C(=O)C2C(C2(C)C)(C)C)c(o1)C	7.3
246	FC(F)(F)CCCn1cc(C(=O)C2C(C2(C)C)(C)C)c3cocc31	7.3
247	FC(F)(F)CCCc1cc(C(=O)C2C(C2(C)C)(C)C)cc3c(c([nH]c31)C)C	7.3
248	FC(F)(F)CCCc1cc(cc(C(=O)C2C(C2(C)C)(C)C)c1)C(C)(C)C	7.3
249	Clc1cc(CCCC(F)(F)F)cc(C(=O)C2C(C2(C)C)(C)C)c1	7.3
250	FC(F)(F)CCCn1cc(C(=O)C2C(C2(C)C)(C)C)c3cc(c(cc31)C)C	7.3
251	FC(F)(F)CCCc1c2C=COC(=O)c2c([nH]1)C(=O)C3C(C3(C)C)(C)C	7.3
252	Brc1cc(CCCC(F)(F)F)cc(C(=O)C2C(C2(C)C)(C)C)c1C	7.3
253	FC(F)(F)CCCc1c[n+](C(=O)C2C(C2(C)C)(C)C)cn1C	7.3
254	FC(F)(F)CCCn1cc(C(=O)C2C(C2(C)C)(C)C)cc1CO	7.3
255	FC(F)(F)CCCc1cc2cccnc2c(C(=O)C3C(C3(C)C)(C)C)c1	7.3
256	FC(F)(F)CCCc1c2ccncc2c([nH]1)C(=O)C3C(C3(C)C)(C)C	7.3
257	FC(F)(F)CCCn1cc(C(=O)C2C(C2(C)C)(C)C)c3c1ncs3	7.3
258	FC(F)(F)CCCN1C=CC=C(C(=O)C2C(C2(C)C)(C)C)C1	7.3
259	FC(F)(F)CCCn1c2cc(ccc2c(n1)C(=O)C3C(C3(C)C)(C)C)C	7.3
260	Clc1c(C(=O)C2C(C2(C)C)(C)C)c3ccccc3n1CCCC(F)(F)F	7.3
261	FC(F)(F)CCCn1c2ccc(F)cc2c(n1)C(=O)C3C(C3(C)C)(C)C	7.3
262	Brc1c(O)c(C(=O)C2C(C2(C)C)(C)C)c(O)c(CCCC(F)(F)F)c1	7.3
263	FC(F)(F)CCCn1cc(C(=O)C2C(C2(C)C)(C)C)c3cccc(F)c31	7.3
264	FC(F)(F)CCCn1c2ccc(cc2c(C(=O)C3C(C3(C)C)(C)C)c1)C	7.3
265	FC(F)(F)CCCn1cc(C(=O)C2C(C2(C)C)(C)C)c3c(O)cccc31	7.3
266	FC(F)(F)CCCc1cc(C(=O)C2C(C2(C)C)(C)C)c3c(CCCO3)c1	7.3
267	FC(F)(F)CCCc1c(c(c([nH]1)C(=O)C2C(C2(C)C)(C)C)C)C	7.3
268	FC(F)(F)CCCc1c2c(c([nH]1)C(=O)C3C(C3(C)C)(C)C)cns2	7.3
269	FC(F)(F)CCCc1cc2c(OCCO2)c(C(=O)C3C(C3(C)C)(C)C)c1	7.3
270	FC(F)(F)CCCn1c2c(c(C(=O)C3C(C3(C)C)(C)C)c1)ccs2	7.3
271	FC(F)(F)CCCc1c2c(n(C(=O)C3C(C3(C)C)(C)C)c1)C(=O)C=CO2	7.3
272	FC(F)(F)CCCn1c2ccc(cc2c(C(=O)C3C(C3(C)C)(C)C)c1)C(F)(F)F	7.3
273	FC(F)(F)CCCc1c(c(c(s1)C(=O)C2C(C2(C)C)(C)C)C#N)C	7.3

274	FC(F)(F)CCCn1cc(C(=O)C2C(C2(C)C)(C)C)c3cc4c(OCCO4)cc31	7.3
275	FC(F)(F)CCCc1cc(cc(C(=O)C2C(C2(C)C)(C)C)c1)CC#N	7.3
276	FC(F)(F)CCCn1c2c(ncs2)c(C(=O)C3C(C3(C)C)(C)C)c1	7.3
277	FC(F)(F)CCCn1cc2-n(scns2)c(C(=O)C3C(C3(C)C)(C)C)c1	7.3
278	Clc1c(C(=O)C2C(C2(C)C)(C)C)c3ccc(cc3n1CCCC(F)(F)F)C	7.3
279	FC(F)(F)CCCn1cc(C(=O)C2C(C2(C)C)(C)C)c3c1cns3	7.3
280	FC(F)(F)CCCc1cc2c(ncnn2)c(C(=O)C3C(C3(C)C)(C)C)c1	7.3
281	FC(F)(F)CCCn1c2c(SC(=O)C=N2)c(C(=O)C3C(C3(C)C)(C)C)c1	7.3
282	FC(F)(F)CCCn1c2ccc(cc2c(n1)C(=O)C3C(C3(C)C)(C)C)C#N	7.3
283	FC(F)(F)CCCc1cccc(C(=O)C2C(C2(C)C)(C)C)c1	7.3
284	FC(F)(F)CCCn1cc(C(=O)C2C(C2(C)C)(C)C)c3c1cc[nH]3	7.3
285	FC(F)(F)CCCc1cc(C(=O)C2C(C2(C)C)(C)C)c(s1)C	7.3
286	Clc1c(C(=O)C2C(C2(C)C)(C)C)cc(s1)CCCC(F)(F)F	7.3
287	FC(F)(F)CCCn1cc(C(=O)C2C(C2(C)C)(C)C)c3cc4c(OCO4)cc31	7.3
288	FC(F)(F)CCC[n+]1cc(C(=O)C2C(C2(C)C)(C)C)c(n1C)N	7.3
289	FC(F)(F)CCCn1cc(n2n1[nH]ccs2)C(=O)C3C(C3(C)C)(C)C	7.3
290	FC(F)(F)c1cn(CCCC(F)(F)F)cc1C(=O)C2C(C2(C)C)(C)C	7.3
291	FC(F)(F)CCCc1cc(C(=O)C2C(C2(C)C)(C)C)c(s1)OC	7.3
292	Clc1c(O)c(C(=O)C2C(C2(C)C)(C)C)c(O)c(CCCC(F)(F)F)c1	7.3
293	FC(F)(F)CCCn1c2cnncc2c(C(=O)C3C(C3(C)C)(C)C)c1	7.3
294	FC(F)(F)CCCc1c[n+](cc(C(=O)C2C(C2(C)C)(C)C)c1)C	7.3
295	FC(F)(F)CCCc1c2c(c([nH]1)C(=O)C3C(C3(C)C)(C)C)cco2	7.3
296	FC(F)(F)CCCn1c2c(c(C(=O)C3C(C3(C)C)(C)C)c1)cco2	7.3
297	FC(F)(F)CCCn1c(O)c(C(=O)C2C(C2(C)C)(C)C)c3ccccc31	7.3
298	FC(F)(F)CCCc1cc(C(=O)C2C(C2(C)C)(C)C)cc3c1[nH]cn3	7.3
299	FC(F)(F)CCCn1cc(n2n1scco2)C(=O)C3C(C3(C)C)(C)C	7.3
300	FC(F)(F)CCCn1cc(C(=O)C2C(C2(C)C)(C)C)c3c1cc(cn3)C	7.3
301	FC(F)(F)CCCc1cc(C(=O)C2C(C2(C)C)(C)C)cc3c[nH]cc31	7.3
302	FC(F)(F)CCCn1cc2-n(ocns2)c(C(=O)C3C(C3(C)C)(C)C)c1	7.3
303	FC(F)(F)CCCn1c2c(snn2)c(C(=O)C3C(C3(C)C)(C)C)c1	7.3
304	FC(F)(F)CCCn1c2c(c(C(=O)C3C(C3(C)C)(C)C)c1)cnc(n2)C	7.3
305	FC(F)(F)CCCn1c2ccncc2c(C(=O)C3C(C3(C)C)(C)C)c1	7.3
306	Clc1c(cc(CCCC(F)(F)F)cc1C(=O)C2C(C2(C)C)(C)C)C	7.3
307	FC(F)(F)CCCc1cc(C(=O)C2C(C2(C)C)(C)C)cn1CCC	7.3
308	FC(F)(F)CCCn1cc(C(=O)C2C(C2(C)C)(C)C)c3C(=O)NC=Cc31	7.3
309	FC(F)(F)CCCn1c2c(c(C(=O)C3C(C3(C)C)(C)C)c1)c(O)ncn2	7.3
310	FC(F)(F)CCCn1cc(C(=O)C2C(C2(C)C)(C)C)cc1C[N+]C	7.3
311	FC(F)(F)CCCn1c2c(C[C@@H]3C[C@H]23)c(n1)C(=O)C4C(C4(C)C)(C)C	7.3
312	FC(F)(F)CCCc1cc(C(=O)C2C(C2(C)C)(C)C)cn1CC	7.3
313	FC(F)(F)CCCn1c2c(c(C(=O)C3C(C3(C)C)(C)C)c1)ccc(OC)n2	7.3
314	FC(F)(F)CCCn1c2cccn2c(n1)C(=O)C3C(C3(C)C)(C)C	7.3
315	FC(F)(F)CCCn1cc(C(=O)C2C(C2(C)C)(C)C)c3c1nccn3	7.3

316	FC(F)(F)CCCc1cc(C(=O)C2C(C2(C)C)(C)C)cc3cncn31	7.3
317	FC(F)(F)CCCc1cn2-c(sncs2)c([nH]1)C(=O)C3C(C3(C)C)(C)C	7.3
318	Clc1cc(CCCC(F)(F)F)cc(C(=O)C2C(C2(C)C)(C)C)c1C#N	7.3
319	Clc1c(C(=O)C2C(C2(C)C)(C)C)c3cc(F)ccc3n1CCCC(F)(F)F	7.3
320	FC(F)(F)CCC[n+]1cc(n2CCCc21)C(=O)C3C(C3(C)C)(C)C	7.3
321	Clc1c(Cl)cc(CCCC(F)(F)F)c(O)c1C(=O)C2C(C2(C)C)(C)C	7.3
322	Clc1c(C(=O)C2C(C2(C)C)(C)C)c3cc(ccc3n1CCCC(F)(F)F)C	7.3
323	FC(F)(F)CCCc1cc(C(=O)C2C(C2(C)C)(C)C)cc(C3(CC3)C#N)c1	7.3
324	Brc1cc(CCCC(F)(F)F)cc(C(=O)C2C(C2(C)C)(C)C)c1	7.3
325	FC(F)(F)CCCn1c2c(c(C(=O)C3C(C3(C)C)(C)C)c1)cccn2	7.3
326	FC(F)(F)CCCn1c2ccc(cc2c(C(=O)C3C(C3(C)C)(C)C)c1C)C	7.3
327	FC(F)(F)CCCn1cc2-n(onco2)c(C(=O)C3C(C3(C)C)(C)C)c1	7.3
328	FC(F)(F)CCCc1cc(C(=O)C2C(C2(C)C)(C)C)c(n1CCC)C	7.2
329	Clc1ccc2c(n(CCCC(F)(F)F)cc2C(=O)C3C(C3(C)C)(C)C)c1C	7.2
330	FC(F)(F)CCCc1cc2c(nccn2)c(C(=O)C3C(C3(C)C)(C)C)c1	7.2
331	FC(F)(F)CCCc1cc2cscc2c(C(=O)C3C(C3(C)C)(C)C)c1	7.2
332	FC(F)(F)CCCn1cc2-n(sccs2)c(C(=O)C3C(C3(C)C)(C)C)c1	7.2
333	FC(F)(F)CCCc1cc2cncnc2c(C(=O)C3C(C3(C)C)(C)C)c1	7.2
334	Clc1c(Cl)c([nH]c1CCCC(F)(F)F)C(=O)C2C(C2(C)C)(C)C	7.2
335	FC(F)(F)CCC[n+]1c2n(c(C(=O)C3C(C3(C)C)(C)C)c1)CCCS2	7.2
336	FC(F)(F)CCCc1cc(C(=O)C2C(C2(C)C)(C)C)c(s1)SC	7.2
337	FC(F)(F)CCCc1cc2c(nncn2)c(C(=O)C3C(C3(C)C)(C)C)c1	7.2
338	FC(F)(F)CCCc1cc(C(=O)C2C(C2(C)C)(C)C)cn1C3CC3	7.2
339	FC(F)(F)CCCc1cn2cccc2c(C(=O)C3C(C3(C)C)(C)C)c1	7.2
340	FC(F)(F)CCCc1cc2c(onn2)c(C(=O)C3C(C3(C)C)(C)C)c1	7.2
341	FC(F)(F)CCCn1cc(C(=O)C2C(C2(C)C)(C)C)c3cc(-c4ccco4)ccc31	7.2
342	FC(F)(F)CCCc1cc(C(=O)C2C(C2(C)C)(C)C)c(s1)C#N	7.2
343	FC(F)(F)CCCn1c(c(c(C(=O)C2C(C2(C)C)(C)C)c1N)C)C	7.2
344	FC(F)(F)CCCn1c2c(c(C(=O)C3C(C3(C)C)(C)C)c1)c(OC)nc(n2)N	7.2
345	FC(F)(F)CCCc1cc(C(=O)C2C(C2(C)C)(C)C)c(s1)C=O	7.2
346	FC(F)(F)CCCc1c2ccsc2c(o1)C(=O)C3C(C3(C)C)(C)C	7.2
347	FC(F)(F)CCCc1cc(OC)cc(C(=O)C2C(C2(C)C)(C)C)c1	7.2
348	FC(F)(F)CCCn1cc(C(=O)C2C(C2(C)C)(C)C)c3cccn31	7.2
349	FC(F)(F)CCCc1c2c(n(C(=O)C3C(C3(C)C)(C)C)c1)C(=O)C=CS2	7.2
350	FC(F)(F)CCCc1c2c(c([nH]1)C(=O)C3C(C3(C)C)(C)C)C(=O)C=CS2	7.2
351	FC(F)(F)CCCn1c2ccc(cc2c(C(=O)C3C(C3(C)C)(C)C)c1O)C	7.2
352	FC(F)(F)CCCn1cc(C(=O)C2C(C2(C)C)(C)C)c3c1SC(=O)N3	7.2
353	FC(F)(F)CCCn1c2c(c(C(=O)C3C(C3(C)C)(C)C)c1)cncn2	7.2
354	Clc1c(Cl)c(sc1CCCC(F)(F)F)C(=O)C2C(C2(C)C)(C)C	7.2
355	FC(F)(F)CCCn1cc(C(=O)C2C(C2(C)C)(C)C)c3cc(CC)ccc31	7.2
356	FC(F)(F)CCCc1ccc(c(C(=O)C2C(C2(C)C)(C)C)c1)C	7.2
357	Brc1c(C(=O)C2C(C2(C)C)(C)C)cc(o1)CCCC(F)(F)F	7.2

358	FC(F)(F)CCCc1cc2c(n[nH]n2)c(C(=O)C3C(C3(C)C)(C)C)c1	7.2
359	Brc1cc(C(=O)C2C(C2(C)C)(C)C)cn1CCCC(F)(F)F	7.2
360	FC(F)(F)CCCc1cc2c(c(C(=O)C3C(C3(C)C)(C)C)c1)ccs2	7.2
361	FC(F)(F)CCCc1ccc(F)c(C(=O)C2C(C2(C)C)(C)C)c1O	7.2
362	FC(F)(F)CCC[n+]1cc(F)c(OC)c(C(=O)C2C(C2(C)C)(C)C)c1	7.2
363	FC(F)(F)CCCc1c2c(nco2)cc(C(=O)C3C(C3(C)C)(C)C)c1	7.2
364	FC(F)(F)CCCC1=C2C(SC=CS2)=C(C(=O)C3C(C3(C)C)(C)C)C1=O	7.2
365	FC(F)(F)CCCn1c2c(c(C(=O)C3C(C3(C)C)(C)C)c1)ccnn2	7.2
366	FC(F)(F)CCCc1c(O)c2ccccc2c(C(=O)C3C(C3(C)C)(C)C)c1	7.2
367	FC(F)(F)CCCc1cc(C(=O)C2C(C2(C)C)(C)C)c3c(scn3)n1	7.2
368	FC(F)(F)CCCc1cc(OCC)c(F)c(C(=O)C2C(C2(C)C)(C)C)c1	7.2
369	Clc1c(C(=O)C2C(C2(C)C)(C)C)c3cc(Cl)ccc3n1CCCC(F)(F)F	7.2
370	FC(F)(F)CCCn1cc(n2cccc21)C(=O)C3C(C3(C)C)(C)C	7.2
371	FC(F)(F)CCCc1cc(nc2cccn21)C(=O)C3C(C3(C)C)(C)C	7.2
372	FC(F)(F)CCCc1c2cnoc2cc(C(=O)C3C(C3(C)C)(C)C)c1	7.2
373	FC(F)(F)CCCn1cc2-n(occs2)c(C(=O)C3C(C3(C)C)(C)C)c1	7.2
374	FC(F)(F)CCCn1cc2-n(scco2)c(C(=O)C3C(C3(C)C)(C)C)c1	7.2
375	FC(F)(F)CCCc1cc(C(=O)C2C(C2(C)C)(C)C)c(c(n1)C)C#N	7.2
376	Clc1c(OC)cc2c(n(CCCC(F)(F)F)cc2C(=O)C3C(C3(C)C)(C)C)c1	7.2
377	FC(F)(F)CCCc1cc(C(=O)C2C(C2(C)C)(C)C)cc3c1cco3	7.2
378	FC(F)(F)CCCc1cc2ccnn2c(C(=O)C3C(C3(C)C)(C)C)c1	7.2
379	FC(F)(F)CCCn1c2c(c(C(=O)C3C(C3(C)C)(C)C)c1)con2	7.2
380	FC(F)(F)CCCc1c2c(NC(S2)=O)c(s1)C(=O)C3C(C3(C)C)(C)C	7.2
381	FC(F)(F)CCCn1c2c(O)cccc2c(C(=O)C3C(C3(C)C)(C)C)c1C	7.2
382	FC(F)(F)CCCc1c2c(scn2)c([nH]1)C(=O)C3C(C3(C)C)(C)C	7.2
383	FC(F)(F)CCCn1c2c(c(C(=O)C3C(C3(C)C)(C)C)c1)csn2	7.2
384	FC(F)(F)CCCn1cc(C(=O)C2C(C2(C)C)(C)C)c3cc(O)ccc31	7.2
385	Clc1c(C(=O)C2C(C2(C)C)(C)C)cc(o1)CCCC(F)(F)F	7.1
386	FC(F)(F)CCCc1cc(F)c(c(C(=O)C2C(C2(C)C)(C)C)c1)C	7.1
387	FC(F)(F)CCCn1cc(C(=O)C2C(C2(C)C)(C)C)c3ccc4ccccc4c31	7.1
388	FC(F)(F)CCCc1c2cnccc2c(o1)C(=O)C3C(C3(C)C)(C)C	7.1
389	Brc1c(c([nH]c1CCCC(F)(F)F)C(=O)C2C(C2(C)C)(C)C)C#N	7.1
390	FC(F)(F)CCCc1ccc(c(C(=O)C2C(C2(C)C)(C)C)c1)C#C	7.1
391	FC(F)(F)CCCc1c2c(n(C(=O)C3C(C3(C)C)(C)C)c1)C(SC=N2)=O	7.1
392	Clc1c(C(=O)C2C(C2(C)C)(C)C)c3cc(c(cc3n1CCCC(F)(F)F)C)C	7.1
393	FC(F)(F)CCCn1cc2-n(snco2)c(C(=O)C3C(C3(C)C)(C)C)c1	7.1
394	FC(F)(F)CCCc1cc(sc1CCO)C(=O)C2C(C2(C)C)(C)C	7.1
395	FC(F)(F)CCCc1cc(c(c(C(=O)C2C(C2(C)C)(C)C)c1)C#N)C	7.1
396	Clc1c(C(=O)C2C(C2(C)C)(C)C)c3cccc(F)c3n1CCCC(F)(F)F	7.1
397	FC(F)(F)CCCc1cc2cnccc2c(C(=O)C3C(C3(C)C)(C)C)c1	7.1
398	Clc1ccc(CCCC(F)(F)F)c(O)c1C(=O)C2C(C2(C)C)(C)C	7.1
399	FC(F)(F)CCCn1cc(C(=O)C2C(C2(C)C)(C)C)c3cc(C(C)C)ccc31	7.1

400	FC(F)(F)CCCc1cc2c(nc(O)cc2C)c(C(=O)C3C(C3(C)C)(C)C)c1	7.1
401	FC(F)(F)CCCc1cc2c(c(C(=O)C3C(C3(C)C)(C)C)c1)cns2	7.1
402	FC(F)(F)CCCc1cc(SC)cc(C(=O)C2C(C2(C)C)(C)C)c1	7.1
403	FC(F)(F)CCCc1cc(F)cc(C(=O)C2C(C2(C)C)(C)C)c1O	7.1
404	FC(F)(F)CCC[n+]1c(N)ccc(C(=O)C2C(C2(C)C)(C)C)c1	7.1
405	FC(F)(F)CCCn1cc(C(=O)C2C(C2(C)C)(C)C)c3cccc(OCC)c31	7.1
406	FC(F)(F)CCCc1cc2c(N=CC(S2)=O)c(C(=O)C3C(C3(C)C)(C)C)c1	7.1
407	Brc1cc(CCCC(F)(F)F)cc(C(=O)C2C(C2(C)C)(C)C)c1F	7.1
408	FC(F)(F)CCCn1cc(C(=O)C2C(C2(C)C)(C)C)c3C[N+]CCc31	7.1
409	FC(F)(F)CCCc1cc(C(=O)C2C(C2(C)C)(C)C)c3ccn(c3c1)C	7.1
410	FC(F)(F)CCCn1c2ccncc2c(n1)C(=O)C3C(C3(C)C)(C)C	7.1
411	FC(F)(F)CCCn1c2c(c(C(=O)C3C(C3(C)C)(C)C)c1)cno2	7.1
412	Brc1c(C(=O)C2C(C2(C)C)(C)C)cc(s1)CCCC(F)(F)F	7.1
413	FC(F)(F)CCCc1cc2c(nno2)c(C(=O)C3C(C3(C)C)(C)C)c1	7.1
414	FC(F)(F)CCCc1cc(OCC)cc(C(=O)C2C(C2(C)C)(C)C)c1	7.1
415	FC(F)(F)CCCn1c2c(c(n1)C(=O)C3C(C3(C)C)(C)C)cns2	7.1
416	FC(F)(F)CCCc1cc(C(=O)C2C(C2(C)C)(C)C)c3c(n1)ccnn3	7.1
417	FC(F)(F)CCCn1cc(C(=O)C2C(C2(C)C)(C)C)c3cccc(OC)c31	7.1
418	FC(F)(F)CCCc1cc(F)c(F)c(C(=O)C2C(C2(C)C)(C)C)c1O	7.1
419	Clc1cccc2c1n(CCCC(F)(F)F)cc2C(=O)C3C(C3(C)C)(C)C	7.1
420	FC(F)(F)CCCc1cc2c(OCO2)c(C(=O)C3C(C3(C)C)(C)C)c1	7.1
421	FC(F)(F)CCCc1cc(cc(C(=O)C2C(C2(C)C)(C)C)c1)[C@@H](O)C	7.1
422	FC(F)(F)CCCc1cc(C(=O)C2C(C2(C)C)(C)C)c3c(n1)cccn3	7.1
423	Brc1c(cc(C(=O)C2C(C2(C)C)(C)C)cc1CCCC(F)(F)F)C	7.1
424	FC(F)(F)CCCc1cc(F)cc(C(=O)C2C(C2(C)C)(C)C)c1	7.1
425	FC(F)(F)CCCN1c2ccccc2N(S1(=O)=O)C(=O)C3C(C3(C)C)(C)C	7.1
426	FC(F)(F)CCCc1c(C[N+])cc([nH]1)C(=O)C2C(C2(C)C)(C)C	7.1
427	FC(F)(F)CCCc1cc2ccc(O)nc2c(C(=O)C3C(C3(C)C)(C)C)c1	7.1
428	FC(F)(F)CCCc1c(c(c([nH]1)C(=O)C2C(C2(C)C)(C)C)C#N)C	7.1
429	FC(F)(F)CCCc1c(CC)c(c(s1)C(=O)C2C(C2(C)C)(C)C)C#N	7
430	FC(F)(F)CCCn1cc2-n(onno2)c(C(=O)C3C(C3(C)C)(C)C)c1	7
431	FC(F)(F)CCCc1cnc(c(C(=O)C2C(C2(C)C)(C)C)c1)C#N	7
432	FC(F)(F)CCCc1cc(c([nH]1)C(=O)C2C(C2(C)C)(C)C)C	7
433	FC(F)(F)CCCc1cc(F)c(SC)c(C(=O)C2C(C2(C)C)(C)C)c1	7
434	FC(F)(F)CCCn1cc(C(=O)C2C(C2(C)C)(C)C)c3c1ccc(C(C)(C)C)c3	7
435	FC(F)(F)CCCc1c(sc(C(=O)C2C(C2(C)C)(C)C)c1)C(C)C	7
436	FC(F)(F)CCCN1c2c(C(=O)C(C(=O)C3C(C3(C)C)(C)C)=C1)csn2	7
437	FC(F)(F)CCC[n+]1cc(cc(C(=O)C2C(C2(C)C)(C)C)c1)C	7
438	FC(F)(F)CCCn1c2cc(OC)cc(OC)c2c(C(=O)C3C(C3(C)C)(C)C)c1	7
439	FC(F)(F)CCCn1cc(C(=O)C2C(C2(C)C)(C)C)c3c(C)ccc(c31)C	7
440	FC(F)(F)CCCn1cc(C(=O)C2C(C2(C)C)(C)C)c3cc(cc(OC)c31)C	7
441	FC(F)(F)CCCc1cn(C(=O)C2C(C2(C)C)(C)C)c[n+]1C	7

442	FC(F)(F)CCCc1cc2cccn2c(C(=O)C3C(C3(C)C)(C)C)c1	7
443	FC(F)(F)CCCn1cc2-n([nH]ccs2)c(C(=O)C3C(C3(C)C)(C)C)c1	7
444	FC(F)(F)CCCn1cc2-n(ocno2)c(C(=O)C3C(C3(C)C)(C)C)c1	7
445	FC(F)(F)CCCn1c2c(c(C(=O)C3C(C3(C)C)(C)C)c1)cns2	7
446	FC(F)(F)CCCc1cc(C(=O)C2C(C2(C)C)(C)C)cc(N(C)C)c1	7
447	FC(F)(F)CCCn1cc(C(=O)C2C(C2(C)C)(C)C)c3ccnn31	7
448	Clc1c(OC)cc(CCCC(F)(F)F)cc1C(=O)C2C(C2(C)C)(C)C	7
449	FC(F)(F)CCCc1cc2c(nccc2C)c(C(=O)C3C(C3(C)C)(C)C)c1	7
450	FC(F)(F)CCCc1cccc(C(=O)C2C(C2(C)C)(C)C)c1O	7
451	Brc1c(CCCC(F)(F)F)cc([nH]1)C(=O)C2C(C2(C)C)(C)C	7
452	FC(F)(F)CCCn1cc(C(=O)C2C(C2(C)C)(C)C)cc1C[N+]	7
453	FC(F)(F)CCCn1c2c(c(C(=O)C3C(C3(C)C)(C)C)c1)c(ncn2)NO	7
454	FC(F)(F)CCC[n+]1cc(n2CCCCCc21)C(=O)C3C(C3(C)C)(C)C	7
455	Brc1cc(CCCC(F)(F)F)c(O)c(C(=O)C2C(C2(C)C)(C)C)c1	6.9
456	FC(F)(F)CCCc1cc(S(=O)(=O)N)cc(C(=O)C2C(C2(C)C)(C)C)c1	6.9
457	FC(F)(F)CCCn1cc(C(=O)C2C(C2(C)C)(C)C)c3cc(cc(c31)C)C	6.9
458	FC(F)(F)CCC[n+]1cccc(C(=O)C2C(C2(C)C)(C)C)c1	6.9
459	Clc1cc(CCCC(F)(F)F)cc(C(=O)C2C(C2(C)C)(C)C)c1C	6.9
460	FC(F)(F)CCCn1cc(C(=O)C2C(C2(C)C)(C)C)c3ccc(c(c31)C)C	6.9
461	FC(F)(F)CCCc1ccc(N#C)c(C(=O)C2C(C2(C)C)(C)C)c1	6.9
462	FC(F)(F)CCCc1cc2c(cccc2c(C(=O)C3C(C3(C)C)(C)C)c1)C	6.9
463	FC(F)(F)CCCn1cc(C(=O)C2C(C2(C)C)(C)C)c3cccc(c31)CC	6.9
464	FC(F)(F)CCCc1cc(cc(C(=O)C2C(C2(C)C)(C)C)c1O)C	6.9
465	FC(F)(F)CCCc1c(CC[N+])cc([nH]1)C(=O)C2C(C2(C)C)(C)C	6.9
466	FC(F)(F)CCCc1cc2c(c(C(=O)C3C(C3(C)C)(C)C)c1)cncn2	6.9
467	FC(F)(F)CCCn1cc(C(=O)C2C(C2(C)C)(C)C)c3c1cn[nH]3	6.9
468	Brc1c[n+](CCCC(F)(F)F)cc(C(=O)C2C(C2(C)C)(C)C)c1	6.9
469	FC(F)(F)CCCc1cc(F)c(c(C(=O)C2C(C2(C)C)(C)C)c1)C(=O)C	6.9
470	FC(F)(F)CCCn1cc(C(=O)C2C(C2(C)C)(C)C)c3cn[nH]c31	6.9
471	FC(F)(F)CCCc1cc2ccncc2c(C(=O)C3C(C3(C)C)(C)C)c1	6.9
472	FC(F)(F)CCCc1cc2ccccc2c(C(=O)C3C(C3(C)C)(C)C)c1O	6.9
473	FC(F)(F)CCCc1cn2c(c(C(=O)C3C(C3(C)C)(C)C)c1)ccn2	6.9
474	FC(F)(F)CCCc1cc(sc1CC)C(=O)C2C(C2(C)C)(C)C	6.9
475	FC(F)(F)CCC[n+]1ccc(c(C(=O)C2C(C2(C)C)(C)C)c1)C	6.9
476	FC(F)(F)CCCC1=CN(N2C(SC=C12)=O)C(=O)C3C(C3(C)C)(C)C	6.9
477	FC(F)(F)CCCC1=CN2C(SC=C2C(C(=O)C3C(C3(C)C)(C)C)=C1)=O	6.9
478	FC(F)(F)CCCn1cc(C(=O)C2C(C2(C)C)(C)C)c3cc(OC)cc(c31)C	6.9
479	FC(F)(F)CCCc1c2c(cc(C(=O)C3C(C3(C)C)(C)C)c1)cns2	6.9
480	FC(F)(F)CCCn1cc(C(=O)C2C(C2(C)C)(C)C)c3cncn31	6.8
481	Clc1cc(CCCC(F)(F)F)c(O)c(C(=O)C2C(C2(C)C)(C)C)c1C	6.8
482	FC(F)(F)CCCn1cc2-n(occo2)c(C(=O)C3C(C3(C)C)(C)C)c1	6.8
483	FC(F)(F)CCCn1c2nccn2c(C(=O)C3C(C3(C)C)(C)C)c1	6.8

484	Clc1c[n+](C(=O)C2C(C2(C)C)(C)C)cn1CCCC(F)(F)F	6.8
485	FC(F)(F)CCCn1cc(sc-2cocnn21)C(=O)C3C(C3(C)C)(C)C	6.8
486	FC(F)(F)CCC[n+]1cc(C(=O)C2C(C2(C)C)(C)C)cc3ccccc31	6.8
487	FC(F)(F)CCCc1cc(sc1C[N+])C(=O)C2C(C2(C)C)(C)C	6.8
488	FC(F)(F)CCCn1c2cncn2c(C(=O)C3C(C3(C)C)(C)C)c1	6.8
489	Fc1c(C(=O)C2C(C2(C)C)(C)C)cc(CCCC(F)(F)F)cc1C[N+]	6.8
490	Clc1cc(CCCC(F)(F)F)c(O)c(C(=O)C2C(C2(C)C)(C)C)c1	6.8
491	FC(F)(F)CCCn1cc(C(=O)C2C(C2(C)C)(C)C)c3cccc(C(C)C)c31	6.8
492	FC(F)(F)CCCn1cc2-n(sncs2)c(C(=O)C3C(C3(C)C)(C)C)c1	6.7
493	FC(F)(F)CCCn1cc(C(=O)C2C(C2(C)C)(C)C)c3c(ccc(OC)c31)C	6.7
494	FC(F)(F)CCCc1cc(F)c(N)c(C(=O)C2C(C2(C)C)(C)C)c1	6.7
495	Brc1c(Cl)cc(CCCC(F)(F)F)cc1C(=O)C2C(C2(C)C)(C)C	6.7
496	Clc1ccc(OC)c2c1n(CCCC(F)(F)F)cc2C(=O)C3C(C3(C)C)(C)C	6.7
497	FC(F)(F)CCCn1c2ccccc2[n+](C(=O)C3C(C3(C)C)(C)C)c1	6.7
498	FC(F)(F)CCCc1cc(CC)c(OC)c(C(=O)C2C(C2(C)C)(C)C)c1	6.6
499	FC(F)(F)CCCc1cc(F)c(NC)c(C(=O)C2C(C2(C)C)(C)C)c1	6.6
500	FC(F)(F)CCCn1c2c(cccc2c(C(=O)C3C(C3(C)C)(C)C)c1)C	6.6

Table S10. List, SMILE and predicted pK_i values for Series 6 in CB_1 receptor.

N°	SMILES	Pred pK_i
1	FC(F)(F)CCCn1cc(c2ccccc21)C(=O)NC(CC)(CC)C#N	8.3
2	FC(F)(F)CCCn1cc(c2ccccc21)C(=O)Cc3cc[nH]c3	8.2
3	FC(F)(F)CCCn1cc(C(=O)CC(C2CC2)C3CC3)c4ccccc41	8.2
4	FC(F)(F)CCCn1cc(c2ccccc21)C(=O)NCCCC	8.2
5	Clc1cc(N)ccc1C(=O)c2c3ccccc3n(CCCC(F)(F)F)c2	8.2
6	FC(F)(F)CCCn1cc(-c2c3c(no2)CCCC3)c4ccccc41	8.1
7	FC(F)(F)CCCn1cc(c2ccccc21)C(=O)c3cccc4c3cc[nH]4	8.1
8	FC(F)(F)CCCn1cc(c2ccccc21)C(=O)c3ccn4ccsc34	8.1
9	FC(F)(F)CCCn1cc(c2ccccc21)C(=O)C=C(C3CC3)C4CC4	8
10	FC(F)(F)CCCn1cc(c2ccccc21)-c3c4ccccc4cnn3	8
11	Clc1c(cccc1C(=O)c2c3ccccc3n(CCCC(F)(F)F)c2)C	8
12	FC(F)(F)CCCn1cc(C(=O)CC(C(C)C)C(C)C)c2ccccc21	7.9
13	FC(F)(F)CCCn1cc(c2ccccc21)C(=O)c3ccccc3OC	7.9
14	FC(F)(F)CCCn1cc(c2ccccc21)C(=O)CS(=O)(=O)CC#C	7.9
15	FC(F)(F)CCCn1cc(c2ccccc21)C(=O)c3ccc(N)cc3F	7.9
16	FC(F)(F)CCCn1cc(c2ccccc21)C(=O)[C@H](OC)c3ccccc3	7.9
17	FC(F)(F)CCCn1cc(c2ccccc21)-c3c4cc(OC)ccc4on3	7.9
18	FC(F)(F)CCCn1cc(c2ccccc21)-c3cc(no3)C4CC4	7.9
19	FC(F)(F)CCCn1cc(c2ccccc21)C(=O)CCC(F)(F)F	7.9
20	FC(F)(F)CCCn1cc(c2ccccc21)C(=O)N[C@@H](CCC)C	7.9
21	FC(F)(F)CCCn1cc(c2ccccc21)C(=O)c3cccc4c3cc(o4)C	7.9

22	FC(F)(F)CCCn1cc(C(=O)[C@@H]2CC32CCC3)c4ccccc41	7.9
23	FC(F)(F)CCCn1cc(c2ccccc21)-c3c4ccc(O)cc4on3	7.9
24	Clc1cccc2c1c(no2)-c3c4ccccc4n(CCCC(F)(F)F)c3	7.9
25	FC(F)(F)CCCn1cc(C(=O)[C@@]23CCC[C@H]4C[C@@H](C2)CC[C@H]43)c5ccccc51	7.9
26	Cl[C@@H](C(=O)c1c2ccccc2n(CCCC(F)(F)F)c1)c3ccccc3	7.9
27	FC(F)(F)CCCn1c2ccccc2c(S(=O)(=O)c3ccccc3F)c1	7.8
28	FC(F)(F)CCCn1cc(c2ccccc21)C(=O)[C@@H]3c4ccccc4C3	7.8
29	FC(F)(F)CCCn1cc(c2ccccc21)C(=O)/C=C/C(C)(C)C	7.8
30	FC1(F)CC(C1)C(=O)c2c3ccccc3n(CCCC(F)(F)F)c2	7.8
31	FC(F)(F)CCCn1cc(c2ccccc21)C(=O)[C@H](C(C)C)c3ccccc3	7.8
32	FC(F)(F)CCCn1cc(C=2[C@@H]3CCCC[C@H]3ON2)c4ccccc41	7.8
33	FC(F)(F)CCCn1cc(C(=O)C2[C@H]3CCCC[C@@H]23)c4ccccc41	7.8
34	FC(F)(F)CCCn1cc(c2ccccc21)C(=O)c3cc(F)ccc3N	7.8
35	FC(F)(F)CCCn1cc(C(=O)[C@H]2CCCS2)c3ccccc31	7.8
36	FC(F)(F)CCCn1cc(C(=O)[C@H]2C[C@H]3C=C[C@@H]2C3)c4ccccc41	7.8
37	FC(F)(F)CCCn1cc(C(=O)C2CC(=O)C2)c3ccccc31	7.8
38	ClC1(Cl)[C@](C1)(C(=O)c2c3ccccc3n(CCCC(F)(F)F)c2)C	7.8
39	FC(F)(F)CCCn1cc(C(=O)C[C@@H](C(C)C)C[N+])c2ccccc21	7.8
40	FC(F)(F)CCCn1cc(C(OC(C(C)C)C(C)C)=O)c2ccccc21	7.8
41	Brc1cc(C(=O)c2c3ccccc3n(CCCC(F)(F)F)c2)cs1	7.8
42	FC(F)(F)CCCn1cc(c2ccccc21)C(=O)N[C@H](C(C)C)C	7.7
43	FC(F)(F)CCCn1cc(c2ccccc21)C(=O)[C@@H](C(C)(C)C)C#N	7.7
44	FC(F)(F)CCCn1cc(c2ccccc21)C(=O)NC[C@@H](CC)C	7.7
45	FC(F)(F)CCCn1cc(c2ccccc21)C(=O)C3=CSCCO3	7.7
46	FC(F)(F)CCCn1cc(c2ccccc21)C(=O)N[C@@H](C3CCC3)C	7.7
47	Brc1ccc2c(c(no2)-c3c4ccccc4n(CCCC(F)(F)F)c3)c1	7.7
48	FC(F)(F)CCCn1cc(-c2c3c(no2)CCC3)c4ccccc41	7.7
49	FC(F)(F)CCCn1cc(C(=O)[C@@H](c2ccccc2)C)c3ccccc31	7.7
50	FC(F)(F)CCCn1cc(-n2c(C(C)C)cnn2)c3ccccc31	7.7
51	FC(F)(F)CCCn1cc(c2ccccc21)C(=O)c3ccc(O)cc3F	7.7
52	Brc1c[nH]nc1C(=O)c2c3ccccc3n(CCCC(F)(F)F)c2	7.7
53	FC(F)(F)CCCn1cc(C(=O)C2C3CC4CC(CC2C4)C3)c5ccccc51	7.7
54	FC(F)(F)CCCn1cc(C(=O)C2(CC2)c3cccc(c3)C)c4ccccc41	7.7
55	FC(F)(F)CCCn1cc(c2ccccc21)C(=O)c3csc(c3CC)C	7.7
56	FC(F)(F)CCCn1cc(c2ccccc21)C(=O)/C=C(/C(C)C)C	7.7
57	FC(F)(F)CCCn1cc(c2ccccc21)C(=O)c3cccc(c3F)C	7.7
58	FC(F)(F)CCCn1cc(c2ccccc21)C(=O)c3cccc(OC)c3F	7.7
59	FC(F)(F)CCCn1cc(c2ccccc21)C(=O)c3ccccc3	7.7
60	FC(F)(F)CCCn1cc(-n2c3c(nn2)CCCC3)c4ccccc41	7.7
61	FC(F)(F)CCCn1cc(c2ccccc21)C(=O)NC3(CC3)C	7.7
62	Clc1cc(C)ccc1C(=O)c2c3ccccc3n(CCCC(F)(F)F)c2	7.7
63	FC(F)(F)CCCn1cc(c2ccccc21)C(=O)c3c(oc(c3)C)C(F)(F)F	7.7

64	F[C@@H]1CCN(C1)C(=O)c2c3ccccc3n(CCCC(F)(F)F)c2	7.7
65	FC(F)(F)CCCn1cc(c2ccccc21)C(=O)/C=C/CCCC	7.7
66	FC(F)(F)CCCn1cc(c2ccccc21)-c3c4cc(O)ccc4on3	7.7
67	FC(F)(F)CCCn1cc(-c2c3c(on2)CCCC3)c4ccccc41	7.7
68	FC(F)(F)CCCn1cc(c2ccccc21)-c3c4ccccc4on3	7.7
69	FC(F)(F)CCCn1cc(C(=O)[C@H]2CSCCS2)c3ccccc31	7.7
70	FC(F)(F)CCCn1cc(C(=O)C2(OC)CCCC2)c3ccccc31	7.7
71	FC(F)(F)CCCn1cc(c2ccccc21)C(=O)NCCC	7.7
72	FC(F)(F)CCCn1cc(C(=O)C[C@@H](C(C)C)C)c2ccccc21	7.7
73	FC(F)(F)CCCn1cc(c2ccccc21)C(=O)Cc3cccc(F)c3	7.7
74	FC(F)(F)CCCn1cc(C(=O)[C@@H](CCC)C#N)c2ccccc21	7.6
75	FC(F)(F)CCCn1cc(c2ccccc21)-c3c(C)c(no3)C(F)(F)F	7.6
76	FC(F)(F)CCCn1cc(c2ccccc21)C(=O)c3cc(ccc3SC)C	7.6
77	FC(F)(F)CCCn1cc(C(=O)C[C@H](C(C)C)CC)c2ccccc21	7.6
78	FC(F)(F)CCCn1cc(-n2c3ccc(F)cc3nn2)c4ccccc41	7.6
79	FC(F)(F)CCCn1cc(c2ccccc21)C(=O)c3ccccc3C(OC)=O	7.6
80	Clc1cccc(C(=O)c2c3ccccc3n(CCCC(F)(F)F)c2)c1	7.6
81	FC(F)(F)CCCn1cc(c2ccccc21)C(=O)c3ccc(C)cc3	7.6
82	FC(F)(F)CCCn1cc(c2ccccc21)C(=O)c3ccncc3C	7.6
83	FC(F)(F)CCCn1cc(c2ccccc21)C(=O)NCC/C=C/C	7.6
84	FC(F)(F)CCCn1cc(c2ccccc21)C(=O)c3cc(F)ccc3[N+]([O-])=O	7.6
85	FC(F)(F)CCCn1cc(c2ccccc21)C(=O)C[C@@H](C(F)(F)F)C	7.6
86	Clc1cc(c(cc1C(=O)c2cn(CCCC(F)(F)F)c3ccccc32)C)C	7.6
87	ClC1(Cl)[C@@H]([C@@]1(CC)C(=O)c2c3ccccc3n(CCCC(F)(F)F)c2)C	7.6
88	FC(F)(F)CCCn1cc(-c2c3c(no2)CC[C@@H](C3)C)c4ccccc41	7.6
89	Clc1cn[nH]c1C(=O)c2c3ccccc3n(CCCC(F)(F)F)c2	7.6
90	FC(F)(F)CCCn1cc(c2ccccc21)-c3c4ccccc4no3	7.6
91	FC(F)(F)CCCn1cc(C(=O)C2=CCCCCC2)c3ccccc31	7.6
92	FC(F)(F)CCCn1c2ccccc2c(C(=O)C3(CCCC3)c4cccs4)c1	7.6
93	Clc1ccc(s1)[C@H](C(=O)c2c3ccccc3n(CCCC(F)(F)F)c2)C	7.6
94	Clc1c(Cl)snc1C(=O)c2c3ccccc3n(CCCC(F)(F)F)c2	7.6
95	SCc1cc(on1)-c2c3ccccc3n(CCCC(F)(F)F)c2	7.6
96	FC(F)(F)CCCn1cc(-n2c3ccc(cc3nn2)C)c4ccccc41	7.6
97	FC(F)(F)CCCn1cc(C(=O)C2CCOCC2)c3ccccc31	7.6
98	FC(F)(F)CCCn1c2ccccc2c(C(=O)C34CC5CC(C3)CC(C4)C5)c1	7.6
99	FC(F)(F)CCCn1cc(c2ccccc21)C(=O)c3c([N+]([O-])=O)cn[nH]3	7.6
100	FC(F)(F)CCCn1cc(c2ccccc21)C(=O)c3ccccc3CC	7.6
101	FC(F)(F)CCCn1cc(c2ccccc21)-c3nnc4n3ccs4	7.6
102	FC(F)(F)CCCn1cc(c2ccccc21)C(=O)NCCC=C	7.6
103	FC(F)(F)CCCn1cc(c2ccccc21)C(=O)Cc3cccs3	7.6
104	FC(F)(F)CCCn1cc(c2ccccc21)-c3c4ccc(F)cc4on3	7.6
105	FC(F)(F)CCCn1cc(c2ccccc21)C(=O)c3c(C)csc3	7.5

106	FC(F)(F)CCCn1cc(c2ccccc21)C(=O)/C=C/C(C)C	7.5
107	FC(F)(F)CCCn1cc(c2ccccc21)C(=O)CCCCC#C	7.5
108	FC(F)(F)CCCn1cc(c2ccccc21)C(=O)[C@H](SCC)C(C)C	7.5
109	FC(F)(F)CCCn1cc(C(=O)[C@@H]2C([C@H]2C=C(C)C)(C)C)c3ccccc31	7.5
110	FC(F)(F)CCCn1cc(c2ccccc21)C(=O)c3cc(c(s3)C)C	7.5
111	Brc1ccc(s1)C(=O)c2c3ccccc3n(CCCC(F)(F)F)c2	7.5
112	S=C(N)[C@H](C(=O)c1c2ccccc2n(CCCC(F)(F)F)c1)CC	7.5
113	FC(F)(F)CCCn1cc(c2ccccc21)C(=O)C3=CCCCC3	7.5
114	Sc1ccccc1C(=O)c2c3ccccc3n(CCCC(F)(F)F)c2	7.5
115	Clc1cnccc1C(=O)c2c3ccccc3n(CCCC(F)(F)F)c2	7.5
116	FC(F)(F)CCCn1cc(C(=O)C(C2CC2)C3CC3)c4ccccc41	7.5
117	FC(F)(F)CCCn1cc(-c2c3c(on2)CC[C@@H](C3)C)c4ccccc41	7.5
118	FC(F)(F)CCCn1cc(c2ccccc21)-c3c4c(on3)ccc(c4)C	7.5
119	FC(F)(F)CCCn1cc(C(=O)C2[C@@H]3CCC[C@H]23)c4ccccc41	7.5
120	FC(F)(F)CCCn1cc(c2ccccc21)C(=O)CC(CC)CC	7.5
121	FC(F)(F)CCCn1cc(C(=O)NCC(C)C)c2ccccc21	7.5
122	FC(F)(F)CCCn1cc(c2ccccc21)C(=O)C3=CCCCO3	7.5
123	FC(F)(F)CCCn1cc(c2ccccc21)C(=O)[C@@H](CC)C#N	7.5
124	FC(F)(F)CCCn1cc(c2ccccc21)C(=O)C3=C(OCCS3)C	7.5
125	FC(F)(F)CCCn1cc(c2ccccc21)C(=O)c3cccc(F)c3F	7.5
126	FC(F)(F)CCCn1cc(c2ccccc21)C(=O)CC3(CCCCC3)C	7.5
127	Clc1cc(F)c(cc1C(=O)c2c3ccccc3n(CCCC(F)(F)F)c2)C	7.5
128	Clc1ccc2c(c(no2)-c3c4ccccc4n(CCCC(F)(F)F)c3)c1	7.5
129	FC(F)(F)CCCn1cc(c2ccccc21)C(OC(CC)CC)=O	7.4
130	S=C(N)[C@H](CCC)C(=O)c1c2ccccc2n(CCCC(F)(F)F)c1	7.4
131	Brc1cc(C(=O)c2c3ccccc3n(CCCC(F)(F)F)c2)cc(c1)C	7.4
132	FC(F)(F)CCCn1cc(-n2c3ccccc3nn2)c4ccccc41	7.4
133	FC(F)(F)CCCn1cc(c2ccccc21)C(=O)c3cc(F)ccc3OC	7.4
134	FC(F)(F)CCCn1cc(c2ccccc21)C(=O)c3ccccc3F	7.4
135	FC(F)(F)CCCn1cc(c2ccccc21)C(=O)c3cnc(s3)C	7.4
136	FC(F)(F)CCCn1c2ccccc2c(C(=O)C(C(F)(F)F)(C(F)(F)F)C)c1	7.4
137	FC(F)(F)CCCn1cc(c2ccccc21)C(=O)CC3CCCCCC3	7.4
138	FC(F)(F)CCCn1cc(C(=O)[C@@H](c2cccs2)C)c3ccccc31	7.4
139	FC(F)(F)CCCn1cc(c2ccccc21)C(=O)/C=C/SC	7.4
140	FC(F)(F)CCCn1cc(c2ccccc21)C(=O)c3ccoc3C	7.4
141	Clc1ccc2c(n(nn2)-c3c4ccccc4n(CCCC(F)(F)F)c3)c1	7.4
142	Clc1ccsc1C(=O)c2c3ccccc3n(CCCC(F)(F)F)c2	7.4
143	FC(F)(F)CCCn1cc(C=2[C@@H]3CCC[C@@H]3ON2)c4ccccc41	7.4
144	FC(F)(F)CCCn1cc(c2ccccc21)C(=O)c3c(F)cc(F)cc3F	7.4
145	FC(F)(F)CCCn1cc(C(=O)C2(CC2)c3ccccc3)c4ccccc41	7.4
146	Fc1cc(C(=O)c2c3ccccc3n(CCCC(F)(F)F)c2)cc(c1)C	7.4
147	Cl[C@H](F)C(S(=O)(=O)c1c2ccccc2n(CCCC(F)(F)F)c1)(F)F	7.4

148	FC(F)(F)CCCn1cc(c2ccccc21)C(=O)C=C3CCC(CC3)C	7.4
149	FC(F)(F)CCCn1cc(c2ccccc21)C(=O)[C@H]([N+](C)C)c3cnccc3	7.4
150	FC(F)(F)CCCn1cc(c2ccccc21)C(=O)c3ccc(o3)N	7.4
151	Clc1cc(F)cc(C(=O)c2c3ccccc3n(CCCC(F)(F)F)c2)c1	7.4
152	FC(F)(F)CCCn1cc(c2ccccc21)C(=O)c3ccccc3OCC	7.4
153	FC(F)(F)CCCn1c2ccccc2c(C(=O)C34C5C[C@@H](C3)C[C@@H](C4)C5)c1	7.4
154	FC(F)(F)CCCn1cc(c2ccccc21)C(=O)/C(C)=C/CO	7.4
155	S[C@@H](C(=O)c1c2ccccc2n(CCCC(F)(F)F)c1)C(C)C	7.4
156	FC(F)(F)CCCn1cc(c2ccccc21)C(=O)NCC3CCC3	7.4
157	FC(F)(F)CCCn1cc(c2ccccc21)-c3cc(no3)C	7.4
158	Clc1c(F)cccc1C(=O)c2cn(CCCC(F)(F)F)c3ccccc32	7.4
159	Clc1c(nn(C)c1)C(=O)c2c3ccccc3n(CCCC(F)(F)F)c2	7.4
160	FC(F)(F)CCCn1cc(c2ccccc21)C(=O)c3c(SC)ccs3	7.4
161	FC(F)(F)CCCn1cc(c2ccccc21)C(=O)[C@H](OC)CC	7.4
162	FC(F)CN(C1CC1)C(=O)c2c3ccccc3n(CCCC(F)(F)F)c2	7.4
163	FC(F)(F)CCCn1cc(c2ccccc21)C(=O)NCCC(C)C	7.4
164	FC(F)(F)CCCn1cc(c2ccccc21)C(=O)c3csc(c3)C	7.4
165	FC(F)(F)CCCn1cc(C(=O)[C@@H](c2ccsc2)C)c3ccccc31	7.4
166	FC(F)(F)CCCn1cc(c2ccccc21)-c3c4cc(F)ccc4on3	7.4
167	FC(F)(F)CCCn1cc(c2ccccc21)C(=O)c3ccc(F)cc3F	7.4
168	FC(F)(F)CCCn1cc(C(=O)C2(CCC2)c3cccc(F)c3)c4ccccc41	7.4
169	FC(F)(F)CCCn1cc(C(=O)[C@H]2C[C@@H]3CC[C@H]2C3)c4ccccc41	7.4
170	FC(F)(F)CCCn1cc(C(=O)N2CCS[C@@H]2CCC)c3ccccc31	7.4
171	FC(F)(F)CCCn1cc(C(=O)N2[C@@H](CC[C@@H]2C)C)c3ccccc31	7.4
172	FC(F)(F)CCCn1cc(c2ccccc21)C(=O)c3c([nH]c(c3)C)C	7.4
173	FC(F)(F)CCCn1cc(c2ccccc21)C(=O)CC3CCCC3	7.4
174	FC(F)(F)CCCn1cc(c2ccccc21)C(=O)c3c([N+]([O-])=O)ccc(c3)C	7.4
175	FC(F)(F)CCCn1cc(c2ccccc21)C(=O)[C@H]([N+](C)C)c3ccccc3	7.4
176	Clc1ccncc1C(=O)c2c3ccccc3n(CCCC(F)(F)F)c2	7.4
177	FC(F)(F)CCCn1cc(C(=O)[C@@H](C2CCOCC2)C)c3ccccc31	7.3
178	FC(F)(F)CCCn1cc(c2ccccc21)C(=O)c3ccc(F)c(N)c3	7.3
179	FC(F)(F)CCCn1cc(C(=O)C2=COCCC2)c3ccccc31	7.3
180	FC(F)(F)CCCn1cc(c2ccccc21)C(OC3(CC3)C)=O	7.3
181	FC(F)(F)CCCn1cc(C(=O)[C@H](CCC)CC)c2ccccc21	7.3
182	FC(F)(F)CCCn1cc(c2ccccc21)C(=O)/C=C(\C3CC3)C	7.3
183	FC(F)(F)CCCn1cc(c2ccccc21)C(SC(C)(C)C)=O	7.3
184	FC(F)(F)CCCn1cc(c2ccccc21)C(=O)c3ccc(O)c(F)c3	7.3
185	Clc1ccc(s1)C(=O)c2c3ccccc3n(CCCC(F)(F)F)c2	7.3
186	FC(F)(F)CCCn1cc(C(=O)C2CCCCC2)c3ccccc31	7.3
187	FC(F)(F)CCCn1cc(c2ccccc21)C(=O)c3csnc3C	7.3
188	FC(F)(F)CCCn1cc(c2ccccc21)C(O[C@H](C3CC3)C)=O	7.3
189	FC(F)(F)CCCn1cc(c2ccccc21)C(=O)c3cc[nH]c3C	7.3

190	Clc1nnc(s1)-c2c3ccccc3n(CCCC(F)(F)F)c2	7.3
191	FC(F)(F)CCCn1cc(C(=O)[C@@H]2CCO[C@H]2C)c3ccccc31	7.3
192	FC(F)(F)CCCn1c2ccccc2c(C(=O)C3(CCC3)COC)c1	7.3
193	FC(F)(F)CCCn1cc(c2ccccc21)C(=O)Cc3ccc[nH]3	7.3
194	FC(F)(F)CCCn1cc(c2ccccc21)C(=O)c3ccccc3C#N	7.3
195	FC(F)(F)CCCn1cc(c2ccccc21)C(=O)c3cncc(F)c3	7.3
196	FC(F)(F)CCCn1cc(c2ccccc21)-c3c(C)c(no3)C	7.3
197	FC(F)(F)CCCn1cc(C(=O)C2CCSCC2)c3ccccc31	7.3
198	Clc1cc(C(=O)c2c3ccccc3n(CCCC(F)(F)F)c2)c(Cl)s1	7.3
199	FC(F)(F)CCCn1cc(c2ccccc21)C(=O)C3=C(OCCO3)C	7.3
200	FC(F)(F)CCCn1cc(c2ccccc21)C(=O)NCC	7.3
201	FC(S(=O)(=O)c1c2ccccc2n(CCCC(F)(F)F)c1)(F)C(F)F	7.3
202	FC(F)(F)CCCn1cc(c2ccccc21)C(=O)C[C@@H](C3CC3)C	7.3
203	FC(F)(F)CCCn1cc(c2ccccc21)C(=O)[C@H]3[C@H](CCO3)C	7.3
204	FC(F)(F)CCCn1cc(c2ccccc21)C(=O)/C=C(/CC)C	7.3
205	FC(F)(F)CCCn1cc(c2ccccc21)C(=O)[C@H](O)C(C)(C)C	7.3
206	FC(F)(F)CCCn1cc(C(=O)[C@@H]2[C@@H]([N+]([O-])=O)C2)c3ccccc31	7.3
207	Clc1ccc2c(onc2-c3c4ccccc4n(CCCC(F)(F)F)c3)c1	7.3
208	FC(F)(F)CCCn1cc(C(=O)N2CCC[C@H]([C@H]2C)C)c3ccccc31	7.3
209	FC(F)(F)CCCn1cc(c2ccccc21)C(=O)c3ccco3	7.3
210	FC(F)(F)CCCn1cc(c2ccccc21)-c3cc(no3)CC	7.3
211	FC(F)(F)CCCn1cc(c2ccccc21)C(=O)Cc3ccoc3	7.3
212	FC(F)(F)CCCn1cc(C(=O)C(CC)CC)c2ccccc21	7.3
213	FC(F)(F)CCCn1cc(C(=O)[C@@H]2CCCC[C@@H]2C)c3ccccc31	7.3
214	FC(F)(F)CCCn1cc(c2ccccc21)C(=O)C=C(SC)SC	7.2
215	FC(F)(F)CCCn1cc(c2ccccc21)C(=O)c3ccc(F)cc3C	7.2
216	FC(F)(F)CCCn1cc(c2ccccc21)C(=O)c3cc[nH]c3	7.2
217	FC(F)(F)CCCn1cc(c2ccccc21)C(=O)c3ccc(F)cc3	7.2
218	FC(F)(F)CCCn1cc(c2ccccc21)C(=O)CCCC=C	7.2
219	FC(F)(F)CCCn1cc(c2ccccc21)C(=O)Cc3cc(F)c(F)cc3F	7.2
220	FC(F)(F)CCCn1cc(C(=O)C2CCC2)c3ccccc31	7.2
221	FC(F)(F)CCCn1cc(c2ccccc21)C(=O)NCC(F)F	7.2
222	FC(F)(F)[C@H]1CCCC[C@H]1C(=O)c2c3ccccc3n(CCCC(F)(F)F)c2	7.2
223	FC(F)(F)CCCn1cc(c2ccccc21)C(=O)C(CC)=C	7.2
224	ClC(Cl)=C[C@H]1C([C@H]1C(=O)c2c3ccccc3n(CCCC(F)(F)F)c2)(C)C	7.2
225	SC[C@@H](C(=O)c1c2ccccc2n(CCCC(F)(F)F)c1)C	7.2
226	FC(F)(F)CCCn1c2ccccc2c(C(=O)N3CCCC3)c1	7.2
227	FC(F)(F)CCCn1cc(C(=O)[C@@H]2C(C2)(C)C)c3ccccc31	7.2
228	FC(F)(F)CCCn1cc(c2ccccc21)C(=O)/C=C/CC	7.2
229	FC(F)(F)CCCn1cc(c2ccccc21)C(=O)/C=C/C=C/C	7.2
230	FC(F)(F)CCCn1cc(c2ccccc21)C(=O)c3ccsc3	7.2
231	FC(F)(F)CCCn1cc(c2ccccc21)C(=O)c3c4c(OCCO4)cs3	7.2

232	FC(F)(F)CCCn1cc(c2ccccc21)C(=O)NOCC=C	7.2
233	FC(F)(F)CCCn1cc(c2ccccc21)C(=O)c3cc4ccoc4s3	7.2
234	FC(F)(F)CCCn1cc(c2ccccc21)C(=O)c3ccccc3SC	7.2
235	Brc1cc(c(o1)C(=O)c2c3ccccc3n(CCCC(F)(F)F)c2)C	7.2
236	FC(F)(F)CCCn1cc(C(=O)C2CC=CC2)c3ccccc31	7.2
237	FC(F)(F)CCCn1cc(C(=O)[C@H]([N+])C2CCCC2)c3ccccc31	7.2
238	FC(F)(F)CCCn1cc(c2ccccc21)C(=O)c3c(ccs3)C#N	7.2
239	FC(F)(F)CCCn1cc(c2ccccc21)C(=O)/C=C/C	7.2
240	FC(F)(F)CCCn1cc(c2ccccc21)C(=O)c3ccc[nH]3	7.2
241	FC(F)(F)CCCn1cc(c2ccccc21)C(=O)c3cccc(F)c3	7.2
242	FC(F)(F)CCCn1cc(C(=O)[C@@H]2[C@](C2)(CC)C)c3ccccc31	7.2
243	Clc1ccc(Cl)c(C(=O)c2c3ccccc3n(CCCC(F)(F)F)c2)c1	7.2
244	FC(F)(F)CCCn1cc([S@@](=O)CCC)c2ccccc21	7.2
245	FC(F)(F)CCCn1cc(C(=O)C2CCCC2)c3ccccc31	7.2
246	FC(F)(F)CCCn1cc(c2ccccc21)C(=O)c3ccns3	7.2
247	FC(F)(F)CCCn1cc(C(=O)C2(CC2)c3ccc(F)cc3)c4ccccc41	7.2
248	ClC1(CC(C1)(C(=O)c2c3ccccc3n(CCCC(F)(F)F)c2)C)C	7.2
249	FC(F)(F)CCCn1cc(c2ccccc21)C(O[C@H](CC)C)=O	7.2
250	FC(F)(F)CCCn1cc(c2ccccc21)C(=O)c3ccncc3F	7.2
251	FC(F)(F)CCCn1cc(c2ccccc21)C(=O)C=C3CCC3	7.2
252	FC(F)(F)CCCn1cc(CC2C(C2(C)C)(C)C)c3ccccc31	7.2
253	FC(F)(F)CCCn1cc(C(=O)C2CCCCCCC2)c3ccccc31	7.2
254	FC(F)(F)CCCn1cc(c2ccccc21)C(=O)c3c(cc([nH]3)C)C	7.2
255	Clc1c[nH]nc1C(=O)c2c3ccccc3n(CCCC(F)(F)F)c2	7.2
256	FC(F)(F)CCCn1cc(c2ccccc21)C(=O)c3ccc(s3)C	7.2
257	FC(F)(F)CCCn1c2ccccc2c(C(=O)C3CC(C3)(C)C)c1	7.1
258	FC(F)(F)CCCn1cc(c2ccccc21)C(=O)C[C@@H](CC)C	7.1
259	FC(F)(F)CCCn1cc(C(=O)C2(CC2)C#N)c3ccccc31	7.1
260	FC(F)(F)CCCn1cc(C(=O)[C@@H]([S@@](=O)CC)C)c2ccccc21	7.1
261	FC(F)(F)CCCn1cc(c2ccccc21)C(=O)CCCC	7.1
262	FC(F)(F)CCCn1cc(c2ccccc21)C(=O)c3c(C)ccs3	7.1
263	FC(F)(F)CCCn1cc(c2ccccc21)C(=O)c3cccc(c3)C	7.1
264	FC(F)(F)CCCn1cc(C(=O)C2=CCC[N+](C2)C)c3ccccc31	7.1
265	Clc1ccc(o1)C(=O)c2c3ccccc3n(CCCC(F)(F)F)c2	7.1
266	FC(F)(F)CCCn1c2ccccc2c(C(=O)N3CC[C@H](C3)C)c1	7.1
267	Brc1ccoc1C(=O)c2c3ccccc3n(CCCC(F)(F)F)c2	7.1
268	FC(F)(F)CCCn1cc(c2ccccc21)C(=O)c3ccoc3	7.1
269	FC(F)(F)CCCn1cc(c2ccccc21)C(=O)C3=CC[N+]CC3	7.1
270	FC(F)(F)CCCn1cc(c2ccccc21)C(=O)c3coc(c3)C	7.1
271	FC(F)(F)CCCn1c2ccccc2c(C(=O)[C@@H](CSC)C)c1	7.1
272	FC(F)(F)CCCn1cc(c2ccccc21)-c3cc(C)c(nn3)C	7.1
273	FC(F)(F)CCCn1c2ccccc2c(C(=O)C3(CCCCCC3)C#N)c1	7.1

274	FC(F)(F)CCCn1cc(C(=O)[C@H]2CCOC2)c3ccccc31	7.1
275	FC(F)(F)CS(=O)(=O)c1c2ccccc2n(CCCC(F)(F)F)c1	7.1
276	FC(F)(F)CCCn1cc(c2ccccc21)C(=O)[C@H](SC)CC	7.1
277	FC(F)(F)CCCn1c2ccccc2c(S(=O)(=O)C(CC)CC)c1	7.1
278	FC(F)(F)CCCn1cc(c2ccccc21)C(=O)CCCCC	7.1
279	Brc1ccsc1C(=O)c2c3ccccc3n(CCCC(F)(F)F)c2	7.1
280	Brc1csc(C(=O)c2c3ccccc3n(CCCC(F)(F)F)c2)c1	7.1
281	FC(F)(F)CCCn1cc(c2ccccc21)C(=O)c3c(F)cccn3	7.1
282	FC(F)(C(=O)c1c2ccccc2n(CCCC(F)(F)F)c1)C(F)(F)F	7.1
283	FC(F)(F)CCCn1c2ccccc2c(C(=O)C(C(F)(F)F)(C)C)c1	7.1
284	FC(F)(F)CCCn1cc(c2ccccc21)C(=O)C[C@H](CCC)C	7.1
285	FC1(F)C[C@@H]1C(=O)c2c3ccccc3n(CCCC(F)(F)F)c2	7.1
286	FC(F)(F)CCCn1cc(c2ccccc21)C(=O)c3ccncc3CC	7.1
287	FC(F)(F)CCCn1cc(c2ccccc21)C(=O)c3cscn3	7.1
288	FC(F)(F)CCCn1c2ccccc2c(C(=O)C3(CCCC3)CC)c1	7.1
289	FC(F)(F)CCCn1cc(c2ccccc21)C(=O)N[C@@H](CC)C	7.1
290	Clc1c(scn1)C(=O)c2c3ccccc3n(CCCC(F)(F)F)c2	7.1
291	FC(F)(F)CCCn1cc(c2ccccc21)C(=O)C3=CCCC3	7.1
292	S=C(N)C[C@H](C(=O)c1c2ccccc2n(CCCC(F)(F)F)c1)C	7.1
293	FC(F)(F)CCCn1cc(c2ccccc21)C(=O)c3c(CC)ccs3	7.1
294	FC(F)(F)CCCn1cc(-c2c3c(on2)CCC3)c4ccccc41	7.1
295	FC(F)(F)CCCn1cc(c2ccccc21)C(=O)CSCCC	7.1
296	FC(F)(F)CCCn1cc(C(=O)[C@@H](C2CCCC2)C)c3ccccc31	7.1
297	FC(F)(F)CCCn1cc(C(=O)C2(O)CCC2)c3ccccc31	7.1
298	FC(F)(F)CCCn1cc(C(=O)[C@@H](n2cccn2)C)c3ccccc31	7.1
299	FC(F)(F)CCCn1cc(C(=O)C2CC2)c3ccccc31	7.1
300	Brc1c(noc1-c2c3ccccc3n(CCCC(F)(F)F)c2)C	7.1
301	FC(F)(F)CCCn1cc(c2ccccc21)C(=O)c3c(n[nH]n3)C	7.1
302	FC(F)(F)CCCn1c2ccccc2c(C(=O)[C@](O)(C(F)(F)F)C)c1	7.1
303	FC(F)(F)CCCn1cc(C(=O)[C@H]2CCCCO2)c3ccccc31	7.1
304	FC(F)(F)CCCn1cc(c2ccccc21)C(=O)c3coc(n3)C	7.1
305	FC(F)(F)CCCn1cc(c2ccccc21)C(=O)c3nc(C)cs3	7.1
306	FC(F)(F)CCCn1cc(c2ccccc21)C(=O)c3ccc(o3)C	7.1
307	FC(F)(F)CCCn1cc(c2ccccc21)-c3c(C)cno3	7.1
308	FC(F)(F)CCCn1cc(c2ccccc21)C(=O)Cc3c(nco3)C	7.1
309	FC(F)(F)CCCn1cc(c2ccccc21)C(=O)c3c(oc(n3)C)C	7.1
310	FC(F)(F)CCCn1cc(c2ccccc21)C(=O)C=C(C)C	7.1
311	FC(F)(F)CCCn1cc(c2ccccc21)C(=O)C3=COCC3	7.1
312	FC(F)(F)CCCn1cc(c2ccccc21)C(=O)c3cc(C)cs3	7.1
313	FC(F)(F)CCCn1c2ccccc2c(C(=O)N3CCSC3)c1	7.1
314	FC(F)(F)CCCn1cc(c2ccccc21)C(=O)/C=C/CCC	7
315	FC(F)(F)CCCn1cc(c2ccccc21)C(OC(C)C)=O	7

316	FC(F)(F)CCCn1c2ccccc2c(C(=O)C(CO)(C)C)c1	7
317	FC(F)(F)CCCn1c2ccccc2c(C(=O)C3(CCC3)C#N)c1	7
318	FC(F)(F)CCCn1cc(C(=O)[C@H]2CCC[C@@H](C2)C)c3ccccc31	7
319	FC(F)(F)CCCn1cc(c2ccccc21)C(=O)c3nccs3	7
320	FC(F)(F)CCCn1cc(c2ccccc21)C(=O)CC(C)=C	7
321	FC1(F)CCC(CC1)C(=O)c2c3ccccc3n(CCCC(F)(F)F)c2	7
322	FC(F)(F)CCCn1cc(c2ccccc21)C(=O)c3cncs3	7
323	FC(F)(F)CCCn1cc(c2ccccc21)C(=O)CCC=C	7
324	FC(F)(F)CCCn1cc(C(=O)[C@@H]2[C@@H](CCC2)C#N)c3ccccc31	7
325	FC(F)(F)CCCn1cc(c2ccccc21)C(=O)c3ccc([nH]3)C	7
326	FC(F)(F)CCCn1cc(S(=O)(=O)CC2CCC2)c3ccccc31	7
327	FC(F)(F)CCCn1cc(C(=O)[C@@H]2CC=CCC2)c3ccccc31	7
328	FC(F)(F)CCCn1cc(c2ccccc21)C(=O)CS(=O)(=O)C	7
329	FC(F)(F)CCCn1cc(c2ccccc21)C(=O)c3c(oc(c3)C)C	7
330	FC(F)(F)CCCn1c2ccccc2c(C(=O)N3CC=CC3)c1	7
331	FC(F)(F)CCCn1cc(C(=O)[C@@H]2[C@@H](C2)C)c3ccccc31	7
332	FC(F)(F)CCCn1c2ccccc2c(C(=O)C3(CC3)C)c1	7
333	FC(F)(F)CCCn1cc(c2ccccc21)C(=O)c3c(cc(o3)C)C	7
334	Clc1ccc(F)cc1C(=O)c2c3ccccc3n(CCCC(F)(F)F)c2	7
335	FC(F)(F)CCCn1c2ccccc2c(C(=O)C(CC(C)C)(C)C)c1	7
336	FC(F)(F)CCCn1cc(C(=O)[C@H]2COCCC2)c3ccccc31	7
337	FC(F)(F)CCCn1cc(c2ccccc21)C(=O)c3cc(F)ccc3F	7
338	FC(F)(F)CCCn1cc(C(=O)C[C@H](O)C(C)C)c2ccccc21	7
339	FC(F)(F)CCCn1cc(C(=O)[C@H]2[C@@H](CCC[N+]2)C)c3ccccc31	7
340	FC(F)(F)CCCn1cc(c2ccccc21)C(SCC)=O	7
341	FC(F)(F)CCCn1c2ccccc2c(C(=O)C3(CCCCC3)C)c1	7
342	FC(F)(F)CCCn1cc(c2ccccc21)C(=O)c3c(ocn3)C	7
343	FC(F)(F)CCCn1cc(c2ccccc21)C(=O)CSC(C)(C)C	7
344	FC(F)(F)CCCn1cc(OC[C@H]2CS2)c3ccccc31	7
345	F[C@@H]1C[C@H]1C(=O)c2c3ccccc3n(CCCC(F)(F)F)c2	7
346	Clc1cc(on1)-c2c3ccccc3n(CCCC(F)(F)F)c2	7
347	FC(F)(F)CCCn1cc(C(=O)[C@H](CC)C)c2ccccc21	7
348	FC(F)(F)CCCn1cc(C(=O)C2=CC[C@H]([N+])C2)c3ccccc31	7
349	FC(F)(F)CCCn1cc(C(=O)[C@@H]([N+](C)C)[C@H](CC)C)c2ccccc21	6.9
350	FC(F)(F)CCCn1cc(c2ccccc21)C(=O)CC(C)(C)C	6.9
351	FC(F)(F)CCCn1cc(c2ccccc21)C(=O)C(C(C)C)=C	6.9
352	FC(F)(F)CCCn1cc(c2ccccc21)-c3ccno3	6.9
353	FC(F)(F)CCCn1cc(C(=O)CCC(C)C)c2ccccc21	6.9
354	FC(F)(F)CCCn1cc(c2ccccc21)C(=O)c3csc(n3)C	6.9
355	FC(F)(F)CCCn1cc(C(=O)[C@H](CCC)C)c2ccccc21	6.9
356	FC(F)(F)CCCn1cc(c2ccccc21)C(=O)C=C3CCCCC3	6.9
357	FC(F)(F)CCCn1cc(S(=O)(=O)CC(C)=C)c2ccccc21	6.9

358	FC(F)(F)CCCn1cc(c2ccccc21)C(=O)c3c(ncs3)C	6.9
359	FC(F)(F)CCCn1c2ccccc2c(C(=O)[C@@H](C[N+](C)C)C)c1	6.9
360	FC(F)(F)CCCn1cc(c2ccccc21)-c3cc(on3)C	6.9
361	FC(F)(F)CCCn1cc(c2ccccc21)C(=O)/C(C)=C/C	6.9
362	FC(F)(F)CCCn1cc(S(=O)(=O)CC=C)c2ccccc21	6.9
363	FC(F)(F)CCCn1cc(c2ccccc21)C(=O)C/C=C/C	6.9
364	Br[C@@H](C(=O)c1c2ccccc2n(CCCC(F)(F)F)c1)C(C)(C)C	6.9
365	FC(F)(F)CCCn1cc(c2ccccc21)C(=O)CCc3ccco3	6.9
366	FC(F)(F)CCCn1cc(c2ccccc21)C(=O)C3=C(OCCC3)C	6.9
367	FC(F)(F)C1(CC1)C(=O)c2c3ccccc3n(CCCC(F)(F)F)c2	6.9
368	FC(F)(F)CCCn1cc(C(=O)[C@H]2CCSC2)c3ccccc31	6.9
369	FC(F)(F)CCCn1cc(C(=O)C2C(C2(C)C)(C)C)c3ccccc31	6.9
370	FC(F)(F)CCCn1cc(C(=O)[C@H](C(C)C)CC)c2ccccc21	6.9
371	FC(F)(F)CCCn1c2ccccc2c(C(=O)C(C(F)(F)F)C(F)(F)F)c1	6.9
372	FC(F)(F)CCCn1cc(C(=O)C(OC)(C)C)c2ccccc21	6.9
373	Clc1ccccc1C(=O)c2c3ccccc3n(CCCC(F)(F)F)c2	6.9
374	FC(F)(F)CCCn1c2ccccc2c(C(=O)C3(CCCC3)C)c1	6.9
375	FC(F)(C(F)(F)C(=O)c1c2ccccc2n(CCCC(F)(F)F)c1)C(F)(F)F	6.9
376	FC(F)(F)CCCn1cc(C(=O)[C@@H](CC#C)C)c2ccccc21	6.8
377	FC(F)(F)CCCn1cc(c2ccccc21)C(=O)CC(C)C	6.8
378	S=C(N)[C@@H](C(=O)c1c2ccccc2n(CCCC(F)(F)F)c1)C(C)C	6.8
379	FCCCS(=O)(=O)c1c2ccccc2n(CCCC(F)(F)F)c1	6.8
380	FC(F)(F)CCCn1cc(C(=O)[C@@H]2CC[C@@H](O2)C)c3ccccc31	6.8
381	FC(F)(F)CCCn1cc(C(=O)[C@@H](n2cncn2)C)c3ccccc31	6.8
382	FC(F)(F)CCCn1c2ccccc2c(C(=O)[C@@H](COC)C)c1	6.8
383	FC(F)(F)CCCn1c2ccccc2c(C(=O)C(CCC)(C)C)c1	6.8
384	FC(F)(F)CCCn1cc(c2ccccc21)C(=O)c3c(C)cco3	6.8
385	ClC1(Cl)C[C@H]1C(=O)c2c3ccccc3n(CCCC(F)(F)F)c2	6.8
386	FC(F)(F)CCCn1cc(C(=O)[C@@H]2CC32CC[N+]CC3)c4ccccc41	6.8
387	FC(F)(F)CCCn1cc(c2ccccc21)C(=O)CSC(F)(F)F	6.8
388	FC(F)(F)CCCn1cc(c2ccccc21)C(=O)c3cnsn3	6.8
389	FC(F)(F)CCCn1cc(c2ccccc21)C(=O)CC#CC	6.8
390	Br[C@@H](C(=O)c1c2ccccc2n(CCCC(F)(F)F)c1)C(C)C	6.8
391	FC(F)(F)CCCn1c2ccccc2c(C(=O)C(CC)(C)C)c1	6.8
392	FC(F)(F)CCCn1cc(c2ccccc21)-c3cc(C)cnn3	6.8
393	FC(F)(F)CCCn1cc(c2ccccc21)C(=O)C[C@H](C)C#N	6.8
394	FC(F)(F)CCCn1cc(c2ccccc21)C(=O)C(F)=C(C)C	6.8
395	FC(F)(F)CCCn1cc(C(=O)[C@@H]2C[C@@H]2C3CC3)c4ccccc41	6.8
396	FC(F)(F)CCCn1cc(c2ccccc21)C(=O)CCOC	6.8
397	FC(F)(F)CCCn1c2ccccc2c(C(=O)[C@@H](S(=O)(=O)C(C)C)C)c1	6.8
398	FC(F)(F)CCCn1cc(c2ccccc21)C(=O)C(CCC)=C	6.8
399	FC(F)(F)CCCn1cc(C(=O)[C@H](C[N+])CC)c2ccccc21	6.8

400	FC(F)(F)CCCn1cc(c2ccccc21)C([S@@](=O)CC)=O	6.8
401	FC(F)(F)CCCn1cc(c2ccccc21)C(=O)c3cccs3	6.8
402	FC(F)(F)CCCn1cc(c2ccccc21)C(=O)c3cocn3	6.8
403	FC(F)(F)CCCn1cc(c2ccccc21)C(=O)C/C=C/CC	6.8
404	FC(F)(F)CCCn1cc(OC(C(F)(F)F)C(F)(F)F)c2ccccc21	6.7
405	FC(F)(F)CCCn1cc(C(=O)C(O)(CC)CC)c2ccccc21	6.7
406	FC(F)(F)CCCn1cc(c2ccccc21)C(=O)[C@@H]([N+](C)C)C(C)C	6.7
407	FC(F)(F)CCCn1cc(c2ccccc21)C=3CCON3	6.7
408	Brc1c(onc1-c2c3ccccc3n(CCCC(F)(F)F)c2)C	6.7
409	FC(F)(F)CCCn1cc(C(=O)[C@H]2CSC[N+]2)c3ccccc31	6.7
410	FC(F)(F)CCCn1cc(c2ccccc21)C(=O)CC=C	6.7
411	Clc1ccc(nn1)-c2c3ccccc3n(CCCC(F)(F)F)c2	6.7
412	FC(F)(F)CCCn1c2ccccc2c(C(=O)C(C)(C)C)c1	6.7
413	S[C@@H]([C@@H](CC)C)C(=O)c1c2ccccc2n(CCCC(F)(F)F)c1	6.7
414	FC(F)(F)CCCn1cc(c2ccccc21)-c3ccon3	6.7
415	FC(F)(F)CCCn1cc(C(=O)C2CCC(O)CC2)c3ccccc31	6.7
416	FC(F)(F)CCCn1cc(C(=O)[C@@H]2CCCO2)c3ccccc31	6.7
417	SC(C(=O)c1c2ccccc2n(CCCC(F)(F)F)c1)(C)C	6.7
418	FC(F)(F)CCCn1cc(C(=O)[C@H]([N+]C)CC)c2ccccc21	6.7
419	FC(F)(F)CCCn1cc(C(=O)CS(=O)(=O)CC)c2ccccc21	6.7
420	FC(F)(F)CCCn1cc(C(=O)[C@@H](C2CC2)C)c3ccccc31	6.7
421	FC(F)(F)CCCn1cc(C(=O)[C@@H]2CCC[C@@H](O)C2)c3ccccc31	6.7
422	FC(F)(F)CCCn1cc(c2ccccc21)C(=O)C=C	6.7
423	FC(F)(F)CCCn1cc(c2ccccc21)C(=O)N[C@@H](CC[N+])C	6.7
424	Clc1cc(F)ccc1C(=O)c2c3ccccc3n(CCCC(F)(F)F)c2	6.7
425	FC(F)(F)CCCn1cc(c2ccccc21)C(=O)C(C)=C	6.7
426	FC(F)(F)CCCn1cc(C(=O)[C@@H]2CC[N+]2)c3ccccc31	6.6
427	ClC(C(=O)c1c2ccccc2n(CCCC(F)(F)F)c1)=C	6.6
428	FC(F)(F)CCCn1cc(C(=O)[C@H]([N+]C)C(C)C)c2ccccc21	6.6
429	FC(F)(F)CCCn1cc(c2ccccc21)C(SC)=O	6.6
430	BrC(Br)C(=O)c1c2ccccc2n(CCCC(F)(F)F)c1	6.6
431	FC(F)(F)CCCn1cc(c2ccccc21)C(=O)C[C@H](SC)C	6.6
432	FC(F)(F)CCCn1cc(c2ccccc21)C(=O)[C@@H](SCC)C	6.6
433	FC(F)(F)CCCn1cc(c2ccccc21)C(=O)CC3CCC3	6.6
434	FC(F)(F)CCCn1cc(c2ccccc21)C(=O)c3c(sc(c3)C)C	6.6
435	FC(F)(F)CCCn1cc(c2ccccc21)C(=O)CCC=O	6.6
436	FC(F)(F)CCCn1cc(c2ccccc21)C(=O)CSCC3CC3	6.6
437	FC(F)(F)CCCn1cc(C(=O)CCC2CC2)c3ccccc31	6.6
438	FC(F)(F)CCCn1cc(C(=O)C[C@H]2CCC[N+]2)c3ccccc31	6.6
439	ClC(Cl)C(=O)c1c2ccccc2n(CCCC(F)(F)F)c1	6.6
440	FC(F)(F)CCCn1cc(c2ccccc21)C(=O)[C@H](OC)C	6.6
441	Br[C@H](C(=O)c1c2ccccc2n(CCCC(F)(F)F)c1)C	6.6

442	FC(F)(F)CCCn1cc(C(=O)[C@@]2(CCCS2)C)c3ccccc31	6.6
443	BrC(C(=O)c1c2ccccc2n(CCCC(F)(F)F)c1)(C)C	6.6
444	FC(F)(F)CCCn1cc(c2ccccc21)C(=O)CCC#N	6.6
445	FC(F)(F)CCCn1cc(c2ccccc21)C(=O)CCO[N+]([O-])=O	6.6
446	FC(F)(F)CCCn1cc(C(=O)[C@H]([N+])CCC)c2ccccc21	6.6
447	FC(F)(F)CCCn1cc(C(=O)[C@@H]([N+](CCC)C)C)c2ccccc21	6.5
448	FC(F)(F)CCCn1cc(c2ccccc21)C(=O)[C@@H](SC)C	6.5
449	FC(F)(F)CCCn1c2ccccc2c(C(=O)C3(CCC3)C[N+])c1	6.5
450	SCC(=O)c1c2ccccc2n(CCCC(F)(F)F)c1	6.5
451	ClC(Cl)(Cl)C(=O)c1c2ccccc2n(CCCC(F)(F)F)c1	6.5
452	FC(F)(F)CCCn1cc(C(=O)[C@@H](C(C)C)C)c2ccccc21	6.5
453	FC(F)(F)CCCn1cc(c2ccccc21)C(=O)c3c(F)cc(F)cn3	6.5
454	FC(F)(F)CCCn1cc(c2ccccc21)C(SCCC)=O	6.5
455	F[C@@H](C(=O)c1c2ccccc2n(CCCC(F)(F)F)c1)C	6.5
456	FC(F)(F)CCCn1cc(C(=O)[C@@H]2CC[N+](C2)C)c3ccccc31	6.5
457	FC(F)(F)CCCn1cc(c2ccccc21)C(SCF)=O	6.5
458	FC(F)(F)CCCn1cc(c2ccccc21)C(=O)CN#C	6.5
459	FC(F)(F)CCCn1cc(c2ccccc21)C(=O)C[S@@](=O)C	6.5
460	FC(F)(F)CCCn1cc(c2ccccc21)-c3c(C)con3	6.5
461	FC(F)(F)CCCn1cc(S(=O)(=O)CCCC)c2ccccc21	6.5
462	FC(F)(F)CCCn1cc(c2ccccc21)C(=O)CSCC	6.5
463	FC(F)(F)CCCn1cc(c2ccccc21)C(=O)CCC	6.5
464	FC(F)(F)CCCn1cc([S@@](=O)CC)c2ccccc21	6.5
465	FC(F)(F)CCCn1cc(c2ccccc21)C(=O)CC(F)(F)F	6.5
466	FC(F)(C(=O)c1c2ccccc2n(CCCC(F)(F)F)c1)C(F)F	6.4
467	FC(F)(F)CCCn1cc(c2ccccc21)C(=O)C(CCCC)=C	6.4
468	FC(F)(F)CCCn1cc(c2ccccc21)C(=O)CCC#C	6.4
469	FC(F)(F)CCCn1cc(c2ccccc21)C(=O)CC3CC3	6.4
470	BrC(C(=O)c1c2ccccc2n(CCCC(F)(F)F)c1)=C	6.4
471	FC(F)(F)CCCn1cc(C(=O)[C@H]([N+])C(C)C)c2ccccc21	6.4
472	FC(F)(F)CCCn1cc(OCC2CC2)c3ccccc31	6.4
473	FC(F)(F)CCCn1cc(c2ccccc21)C(=O)[C@H]([N+])C(C)(C)C	6.4
474	FC(F)(F)CCCn1cc(c2ccccc21)C(=O)C(F)(F)F	6.4
475	FC(F)(F)CCCn1cc(c2ccccc21)C(=O)CC[C@@H](O)C	6.4
476	FC(F)(F)CCCn1cc(c2ccccc21)C(OCC3CC3)=O	6.4
477	FC(F)(F)CCCn1c2ccccc2c(C(=O)[C@@]([N+])(C(F)(F)F)C)c1	6.3
478	FC(F)(F)CCCn1cc(c2ccccc21)-c3c(snn3)C	6.3
479	FC(F)(F)CCCn1cc(c2ccccc21)C(=O)C[C@H](CC)C#N	6.3
480	FC(F)(F)CCCn1c2ccccc2c(C(=O)C3(CC3)C(N)=[N+])c1	6.3
481	FC(F)(F)CCCn1cc(c2ccccc21)C(=O)CCCC[N+]	6.3
482	FC(F)(F)CCCn1cc(c2ccccc21)C(=O)CCCC#N	6.3
483	FC(F)(F)CCCn1cc(c2ccccc21)C(SC(C)C)=O	6.3

484	FC(C(=O)c1c2ccccc2n(CCCC(F)(F)F)c1)(C)C	6.3
485	Cl[C@@H](C(=O)c1c2ccccc2n(CCCC(F)(F)F)c1)C	6.3
486	FC(F)(F)CCCn1cc(c2ccccc21)C(=O)CCCC#C	6.3
487	FC(F)(F)CCCn1cc(C(=O)[C@@H]([N+](C)C)CC)c2ccccc21	6.2
488	FC(F)(F)CCCn1cc(c2ccccc21)C(=O)CSC	6.2
489	FC(F)(F)CCCn1cc(C(=O)C(C)C)c2ccccc21	6.2
490	FC(F)(F)CCCn1cc(C(=O)[C@H]2[C@H](C2)C(OC)=O)c3ccccc31	6.2
491	FC(F)(F)CCCn1cc(c2ccccc21)C(=O)CC	6.2
492	FC(F)(F)CCCn1cc(c2ccccc21)C(=O)C[N+](C)(C)C	6.2
493	S[C@H](C(=O)c1c2ccccc2n(CCCC(F)(F)F)c1)C	6.2
494	FC(F)(F)CCCn1cc(C(=O)[C@@H]([N+](CC)C)C)c2ccccc21	6.2
495	ClC(F)(F)C(=O)c1c2ccccc2n(CCCC(F)(F)F)c1	6.1
496	FC(F)(F)CCCn1cc(C(=O)[C@H](CC#N)C)c2ccccc21	6.1
497	FC(F)(F)CCCn1cc(C(=O)[C@@H]2[C@H]([N+])C2)c3ccccc31	6.1
498	FC(F)(F)CCCn1c2ccccc2c(C(=O)C3([N+])CC3)c1	6
499	FC(F)(F)CCCn1cc(c2ccccc21)C(=O)CC[N+]	5.7
500	FC(F)(F)CCCn1c2ccccc2c(C(=O)[C@@]3(CCC[N+]3)C)c1	5.5

Table S11. List, SMILE and predicted pK_i values for Series 1 in CB_2 receptor.

N°	SMILES	Pred pK_i
1	O=C(c1cn(c2ccccc21)Cc3cccc4c3cccn4)c5cccc6ccccc65	9.4
2	O=C(c1cn(Sc2ccc(cc2C)C#N)c3ccccc31)c4cccc5ccccc54	9.4
3	O=C(c1cn(Nc2ccncc2C)c3ccccc31)c4cccc5ccccc54	9.4
4	O=C(c1cn(Cc2cnc(n2C)[N+]([O-])=O)c3ccccc31)c4cccc5ccccc54	9.3
5	O[C@@H](n1cc(c2ccccc21)C(=O)c3cccc4ccccc43)c5cc(ccc5C)C	9.3
6	O=C(c1cn(c2ccccc21)Cn3cc(nc3C)C)c4cccc5ccccc54	9.2
7	Clc1cc(Cl)ccc1[C@@H](O)n2cc(c3ccccc32)C(=O)c4cccc5ccccc54	9.2
8	Fc1cc(ccc1Sn2cc(c3ccccc32)C(=O)c4cccc5ccccc54)C#N	9.2
9	Fc1cnccc1Nn2cc(c3ccccc32)C(=O)c4cccc5ccccc54	9.2
10	O=C(c1cn(Cc2cc(nn2C)C)c3ccccc31)c4cccc5ccccc54	9.1
11	Fc1ccccc1[C@@H](O)n2cc(c3ccccc32)C(=O)c4cccc5ccccc54	9
12	Clc1ccccc1[C@@H](O)n2cc(c3ccccc32)C(=O)c4cccc5ccccc54	9
13	O=C(c1cn(c2ccccc21)Cc3csc4nccn43)c5cccc6ccccc65	9
14	O=C(c1cn(NCCC(C)C)c2ccccc21)c3cccc4ccccc43	9
15	Fc1c(F)cccc1Sn2cc(c3ccccc32)C(=O)c4cccc5ccccc54	9
16	Clc1cc(ccc1Sn2cc(c3ccccc32)C(=O)c4cccc5ccccc54)C#N	9
17	Oc1c(Cn2cc(c3ccccc32)C(=O)c4cccc5ccccc54)c(nc(O)n1)C	9
18	Brc1csc(Cn2cc(c3ccccc32)C(=O)c4cccc5ccccc54)c1	8.9
19	O=C(c1cn(NCCCC)c2ccccc21)c3cccc4ccccc43	8.9
20	FC(F)(F)[C@H](Cn1cc(c2ccccc21)C(=O)c3cccc4ccccc43)C	8.9
21	O=C(c1cn(c2ccccc21)Cn3ccnc3C)c4cccc5ccccc54	8.9

22	O=C(c1cn(c2ccccc21)Cc3ccnn3CC)c4cccc5ccccc54	8.9
23	O=C(c1cn(NC2CCCCCC2)c3ccccc31)c4cccc5ccccc54	8.9
24	Clc1cn(nc1)Cn2cc(c3ccccc32)C(=O)c4cccc5ccccc54	8.9
25	Clc1cc(Cl)cc([C@@H](O)n2cc(c3ccccc32)C(=O)c4cccc5ccccc54)c1	8.8
26	Fc1ccc(c(S(=O)(=O)n2cc(c3ccccc32)C(=O)c4cccc5ccccc54)c1)C	8.8
27	O=C(c1cn([C@H]([N+])c2cc([N+]([O-])=O)ccc2C)c3ccccc13)c4cccc5ccccc54	8.8
28	O=C(c1cn(C[C@H]2C[C@@H](CC2)C)c3ccccc31)c4cccc5ccccc54	8.8
29	O=C(c1cn(C[C@@H]2COCCC2)c3ccccc31)c4cccc5ccccc54	8.8
30	Sc1nccn1Cn2cc(c3ccccc32)C(=O)c4cccc5ccccc54	8.8
31	OCc1ccncc1NCn2cc(c3ccccc32)C(=O)c4cccc5ccccc54	8.8
32	Fc1ccc(F)cc1Sn2cc(c3ccccc32)C(=O)c4cccc5ccccc54	8.8
33	O=C(c1cn(Sc2ncns2)c3ccccc31)c4cccc5ccccc54	8.7
34	O=C(c1cn(c2ccccc21)C3=CC=CN4C(SC=C34)=O)c5cccc6ccccc65	8.7
35	O=C(c1cn(OCCC(C)C)c2ccccc21)c3cccc4ccccc43	8.7
36	Fc1cc(ccc1On2cc(c3ccccc32)C(=O)c4cccc5ccccc54)C#N	8.7
37	O=C(c1cn(SCC(C)=C)c2ccccc12)c3cccc4ccccc43	8.7
38	O=C(c1cn(c2ccccc21)C[n+]3ccccc3N)c4cccc5ccccc54	8.7
39	FC(F)(F)CSn1cc(c2ccccc21)C(=O)c3cccc4ccccc43	8.7
40	Brc1ccc(s1)Cn2cc(c3ccccc32)C(=O)c4cccc5ccccc54	8.7
41	O=C(c1cn(Cc2cc(nn2C)CC)c3ccccc31)c4cccc5ccccc54	8.7
42	Fc1ccc(cc1Cn2cc(c3ccccc32)C(=O)c4cccc5ccccc54)C	8.7
43	O=C(c1cn(c2ccccc21)Cc3ccc(o3)C)c4cccc5ccccc54	8.7
44	O=C(c1cn(c2ccccc21)Cc3cscc3C)c4cccc5ccccc54	8.7
45	O=C(c1cn(Nc2cscc2C)c3ccccc31)c4cccc5ccccc54	8.7
46	O=C(c1cn(c2ccccc21)C/C=C/C(C)C)c3cccc4ccccc43	8.7
47	O=C(c1cn(C[C@@H]2C[C@H]2C)c3ccccc31)c4cccc5ccccc54	8.6
48	Cl/C=C/Cn1cc(c2ccccc21)C(=O)c3cccc4ccccc43	8.6
49	Fc1ccc(O)c([C@@H]([N+])n2cc(c3ccccc32)C(=O)c4cccc5ccccc54)c1	8.6
50	Fc1ccc(Nn2cc(c3ccccc32)C(=O)c4cccc5ccccc54)c(C[N+])c1	8.6
51	FC1(F)CC(C1)Cn2cc(c3ccccc32)C(=O)c4cccc5ccccc54	8.6
52	Brc1cccc(F)c1Cn2cc(c3ccccc32)C(=O)c4cccc5ccccc54	8.6
53	O=C(c1cn(Sc2ccoc2C)c3ccccc31)c4cccc5ccccc54	8.6
54	O=C(c1cn(Nc2ccccc2[C@H]([N+])C)c3ccccc31)c4cccc5ccccc54	8.6
55	Clc1cccc([C@@H]([N+])n2cc(c3ccccc32)C(=O)c4cccc5ccccc54)c1	8.6
56	O=C(c1cn(C[N+]2CCC[C@H](CC2)CC)c3ccccc31)c4cccc5ccccc54	8.6
57	Fc1cc(F)cc(On2cc(c3ccccc32)C(=O)c4cccc5ccccc54)c1C	8.6
58	Clc1ccc(Cl)c(OCn2cc(c3ccccc32)C(=O)c4cccc5ccccc54)c1	8.6
59	F[C@@H]1C[N+](CC1)Cn2cc(c3ccccc32)C(=O)c4cccc5ccccc54	8.6
60	O=C(c1cn(c2ccccc21)Cc3c(nc(s3)C)C)c4cccc5ccccc54	8.6
61	O=C(c1cn(SS[C@@H](CC)C)c2ccccc21)c3cccc4ccccc43	8.6
62	FC(F)(F)C[N+](C(C)C)Cn1cc(c2ccccc21)C(=O)c3cccc4ccccc43	8.6
63	O=C(c1cn(-n2ncoc-3csccn32)c4ccccc41)c5cccc6ccccc65	8.6

64	O=C(c1cn(Nc2cnccc2C[N+])c3ccccc31)c4cccc5ccccc54	8.6
65	ClC(Cl)=C(Cl)Cn1cc(c2ccccc21)C(=O)c3cccc4ccccc43	8.5
66	Fc1ccc2CC[N+][C@@H](n3cc(c4ccccc43)C(=O)c5cccc6ccccc65)c2c1	8.5
67	O=C(c1cn(c2ccccc21)Cc3ccc[nH+]c3NCC)c4cccc5ccccc54	8.5
68	O=C(c1cn(Sc2nc(cs2)C)c3ccccc31)c4cccc5ccccc54	8.5
69	Cl/C=C(\Cl)Cn1cc(c2ccccc21)C(=O)c3cccc4ccccc43	8.5
70	Fc1cc(F)cc(F)c1On2cc(c3ccccc32)C(=O)c4cccc5ccccc54	8.5
71	Fc1cc(F)cc(On2cc(c3ccccc32)C(=O)c4cccc5ccccc54)c1	8.5
72	Fc1cc(F)cc([C@@H](O)n2cc(c3ccccc32)C(=O)c4cccc5ccccc54)c1	8.5
73	O=C(c1cn(C[N+](CCSC)C)c2ccccc21)c3cccc4ccccc43	8.5
74	O=C(c1cn(C[N+]2CCC[C@H](C2)CC)c3ccccc31)c4cccc5ccccc54	8.5
75	O=C(c1cn(c2ccccc21)Cc3cccc([N+]([O-])=O)c3C)c4cccc5ccccc54	8.5
76	Fc1ccc(S(=O)(=O)n2cc(c3ccccc32)C(=O)c4cccc5ccccc54)c(c1)C	8.5
77	O=C(c1cn(NC[C@@H](CC)C)c2ccccc12)c3cccc4ccccc43	8.5
78	Brc1ccc(o1)Cn2cc(c3ccccc32)C(=O)c4cccc5ccccc54	8.5
79	Clc1ccc([N+]([O-])=O)c(Cn2cc(c3ccccc32)C(=O)c4cccc5ccccc54)c1	8.5
80	Fc1cc(c2CC[N+][C@@H](n3cc(c4ccccc43)C(=O)c5cccc6ccccc65)c2c1)C	8.5
81	O=C(c1cn(c2ccccc21)C[C@@](O)(CSC)C)c3cccc4ccccc43	8.5
82	O=C(c1cn(CC[C@@H](SC)C)c2ccccc21)c3cccc4ccccc43	8.5
83	O=C(c1cn(c2ccccc21)-c3csc4cscc43)c5cccc6ccccc65	8.5
84	O=C(c1cn(N2C=3C(=O)C=CC3SC=N2)c4ccccc41)c5cccc6ccccc65	8.5
85	O=C(c1cn(C[N+]2CCCC(C2)(C)C)c3ccccc31)c4cccc5ccccc54	8.5
86	O=C(c1cn([C@H](C[N+])c2cc(sc2C)C)c3ccccc31)c4cccc5ccccc54	8.5
87	Clc1ccc(c([C@@H]([N+])n2cc(c3ccccc32)C(=O)c4cccc5ccccc54)c1)C	8.5
88	O=C(c1cn(c2ccccc21)CC#CCC#C)c3cccc4ccccc43	8.5
89	O=C(c1cn(CCCCSC)c2ccccc21)c3cccc4ccccc43	8.4
90	Fc1ccc(c(C[N+])c1)Cn2cc(c3ccccc32)C(=O)c4cccc5ccccc54	8.4
91	Clc1ccc(F)c(On2cc(c3ccccc32)C(=O)c4cccc5ccccc54)c1	8.4
92	O=C(c1cn(C[N+]2CC[C@H](C2)CC)c3ccccc31)c4cccc5ccccc54	8.4
93	Fc1ccc([C@@H](O)n2cc(c3ccccc32)C(=O)c4cccc5ccccc54)c(c1)C	8.4
94	Fc1cc(F)c(F)cc1On2cc(c3ccccc32)C(=O)c4cccc5ccccc54	8.4
95	O=C(c1cn(C[N+](C2CCC2)C)c3ccccc31)c4cccc5ccccc54	8.4
96	O=S(=O)(n1cc(c2ccccc21)C(=O)c3cccc4ccccc43)c5ccccc5C	8.4
97	O=C(c1cn(c2ccccc21)C/C=C/CC)c3cccc4ccccc43	8.4
98	Fc1ccc([C@@H]([N+])n2cc(c3ccccc32)C(=O)c4cccc5ccccc54)c(c1)C	8.4
99	Clc1c(F)cccc1Cn2cc(c3ccccc32)C(=O)c4cccc5ccccc54	8.4
100	O=C(c1cn(c2ccccc21)Cc3cccc4cnccc43)c5cccc6ccccc65	8.4
101	O=C(c1cn(C[N+]2CCCOCC2)c3ccccc31)c4cccc5ccccc54	8.4
102	O=C(c1cn(C[N+]2CSCC2)c3ccccc31)c4cccc5ccccc54	8.4
103	Clc1ccc(c(Cn2cc(c3ccccc32)C(=O)c4cccc5ccccc54)c1)C[N+]	8.4
104	Clc1cc(F)ccc1CSn2cc(c3ccccc32)C(=O)c4cccc5ccccc54	8.4
105	FC(F)(F)C[N+](Cn1cc(c2ccccc21)C(=O)c3cccc4ccccc43)C	8.4

106	Clc1ccc(Cl)cc1[C@@H]([N+])n2cc(c3ccccc32)C(=O)c4cccc5ccccc54	8.4
107	FC(F)(F)CSCn1cc(c2ccccc21)C(=O)c3cccc4ccccc43	8.4
108	O=C(c1cn(c2ccccc21)Cn3ccnc3[N+]([O-])=O)c4cccc5ccccc54	8.4
109	O=C(c1cn(C[N+]2CC(CC2)(C)C)c3ccccc31)c4cccc5ccccc54	8.4
110	O=C(c1cn(NC2CCCC2)c3ccccc31)c4cccc5ccccc54	8.3
111	Clc1ccc(OC)c([C@@H]([N+])n2cc(c3ccccc32)C(=O)c4cccc5ccccc54)c1	8.3
112	Fc1ccc(cc1Cn2cc(c3ccccc32)C(=O)c4cccc5ccccc54)C(=O)N	8.3
113	O=C(c1cn(C[N+]2CCC[C@@H](CC2)C)c3ccccc31)c4cccc5ccccc54	8.3
114	O=C(c1cn(O/N=C(/SC)C)c2ccccc21)c3cccc4ccccc43	8.3
115	O=C(c1cn(C[C@H]2CCCC[C@@H]2C)c3ccccc31)c4cccc5ccccc54	8.3
116	O=C(c1cn(c2ccccc21)C[C@@H](CC(C)C)C#N)c3cccc4ccccc43	8.3
117	O=C(c1cn(SCCC=C)c2ccccc21)c3cccc4ccccc43	8.3
118	O=C(c1cn(c2ccccc21)CC#CCC)c3cccc4ccccc43	8.3
119	O=C(c1cn([C@H]([N+])c2cc(sc2C)C)c3ccccc31)c4cccc5ccccc54	8.3
120	O=C(c1cn(CC2CC=CC2)c3ccccc31)c4cccc5ccccc54	8.3
121	FC(F)(F)CCSn1cc(c2ccccc21)C(=O)c3cccc4ccccc43	8.3
122	O=C(c1cn(O[C@@H]2CSCC2)c3ccccc31)c4cccc5ccccc54	8.3
123	O=C(c1cn(c2ccccc21)Cc3ncc(s3)C)c4cccc5ccccc54	8.3
124	O=C(c1cn(C[N+]2C[C@H](CC2)C)c3ccccc31)c4cccc5ccccc54	8.3
125	Fc1ccc([N+]([O-])=O)c(Cn2cc(c3ccccc32)C(=O)c4cccc5ccccc54)c1	8.3
126	Fc1ccc(c(Cn2cc(c3ccccc32)C(=O)c4cccc5ccccc54)c1)C[N+]	8.3
127	O[C@@H](n1cc(c2ccccc21)C(=O)c3cccc4ccccc43)c5cscc5	8.3
128	O=C(c1cn([C@H](C[N+])c2ccccc2C)c3ccccc31)c4cccc5ccccc54	8.3
129	FC(F)(F)c1c(F)cccc1-n2cc(c3ccccc32)C(=O)c4cccc5ccccc54	8.3
130	O=C(c1cn([C@H]([N+])c2ccc(cc2C)C)c3ccccc13)c4cccc5ccccc54	8.2
131	O=C(c1cn(CC(C2CC2)C3CC3)c4ccccc41)c5cccc6ccccc65	8.2
132	Fc1ccccc1Sn2cc(c3ccccc32)C(=O)c4cccc5ccccc54	8.2
133	Fc1ccc(F)cc1[C@H](n2cc(c3ccccc32)C(=O)c4cccc5ccccc54)C[N+]	8.2
134	O=C(c1cn(N[C@@H]2CSCC2)c3ccccc31)c4cccc5ccccc54	8.2
135	FC(F)(F)C[N+](Cn1cc(c2ccccc21)C(=O)c3cccc4ccccc43)CC	8.2
136	Clc1ccc(F)c([C@@H]([N+])n2cc(c3ccccc32)C(=O)c4cccc5ccccc54)c1	8.2
137	FCCCCCn1cc(c2ccccc21)C(=O)c3cccc4ccccc43	8.2
138	O=C(c1cn(c2ccccc21)-c3cc[n+](c4ccccc43)C)c5cccc6ccccc65	8.2
139	Fc1cccc([C@@H]([N+])n2cc(c3ccccc32)C(=O)c4cccc5ccccc54)c1	8.2
140	O=C(c1cn(Sc2ccccc2C[N+]C)c3ccccc31)c4cccc5ccccc54	8.2
141	O=C(c1cn(C[C@@H](CCC)C#N)c2ccccc21)c3cccc4ccccc43	8.2
142	Clc1ccc(On2cc(c3ccccc32)C(=O)c4cccc5ccccc54)c(F)c1	8.2
143	FCCCSn1cc(c2ccccc21)C(=O)c3cccc4ccccc43	8.2
144	Fc1cccc([C@@H](O)n2cc(c3ccccc32)C(=O)c4cccc5ccccc54)c1	8.2
145	O=C(c1cn(c2ccccc21)C#CCCCCC)c3cccc4ccccc43	8.2
146	O=C(c1cn([C@@H]([N+]C)C2CCCCC2)c3ccccc31)c4cccc5ccccc54	8.2
147	O=C(c1cn(C[N+]2CCC[C@H]2C(C)C)c3ccccc31)c4cccc5ccccc54	8.2

148	O=C(c1cn(CC2C[C@H]3CC[C@H]([N+]3C)C2)c4ccccc41)c5cccc6ccccc65	8.2
149	O=C(c1cn([C@H]([N+])c2c(ccs2)C)c3ccccc13)c4cccc5ccccc54	8.2
150	O[C@H]1CCCC[C@@H]1Cn2cc(c3ccccc32)C(=O)c4cccc5ccccc54	8.2
151	Clc1ccc(o1)Cn2cc(c3ccccc32)C(=O)c4cccc5ccccc54	8.2
152	O=C(c1cn(c2ccccc21)Cn3ccnc3C[N+])c4cccc5ccccc54	8.2
153	Fc1cccc(On2cc(c3ccccc32)C(=O)c4cccc5ccccc54)c1C(N)=[N+]	8.2
154	O=C(c1cn(C[C@H]2CCCS2)c3ccccc31)c4cccc5ccccc54	8.2
155	Fc1ccc(c(Cn2cc(c3ccccc32)C(=O)c4cccc5ccccc54)c1)C#N	8.2
156	O=C(c1cn(SCCCCC)c2ccccc21)c3cccc4ccccc43	8.2
157	O[C@@H](n1cc(c2ccccc21)C(=O)c3cccc4ccccc43)C[N+](CC)CC	8.2
158	O=C(c1cn(C[C@H]([N+](C)C)C2CC2)c3ccccc13)c4cccc5ccccc54	8.2
159	O=C(c1cn(c2ccccc21)Cc3cccc(C[N+])c3)c4cccc5ccccc54	8.2
160	Fc1ccc(Sn2cc(c3ccccc32)C(=O)c4cccc5ccccc54)c([C@H]([N+])C)c1	8.2
161	Fc1ccc(On2cc(c3ccccc32)C(=O)c4cccc5ccccc54)c(C[N+])c1	8.1
162	O=C(c1cn(c2ccccc21)C[n+]3ccccc3C)c4cccc5ccccc54	8.1
163	Clc1cc(F)ccc1[C@@H]([N+])n2cc(c3ccccc32)C(=O)c4cccc5ccccc54	8.1
164	O=C(c1cn(C[C@@H](C2CC2)C)c3ccccc31)c4cccc5ccccc54	8.1
165	FC(Sn1cc(c2ccccc21)C(=O)c3cccc4ccccc43)(F)C(F)F	8.1
166	O=C(c1cn(CCCCCC#C)c2ccccc21)c3cccc4ccccc43	8.1
167	Fc1cc(F)ccc1[C@H](n2cc(c3ccccc32)C(=O)c4cccc5ccccc54)C[N+]	8.1
168	O=C(c1cn(C[C@H]2C[N+](CCO2)CC)c3ccccc31)c4cccc5ccccc54	8.1
169	O=C(c1cn(C[C@H]2CCC=CO2)c3ccccc31)c4cccc5ccccc54	8.1
170	FC(SCn1cc(c2ccccc21)C(=O)c3cccc4ccccc43)F	8.1
171	ClC(CSn1cc(c2ccccc21)C(=O)c3cccc4ccccc43)=C	8.1
172	O=C(c1cn(C[N+]2C[C@H]([C@H](C2)C)C)c3ccccc31)c4cccc5ccccc54	8.1
173	Brc1ccccc1[C@H](O)n2cc(c3ccccc32)C(=O)c4cccc5ccccc54	8.1
174	Clc1ccc(C[N+]C)c(On2cc(c3ccccc32)C(=O)c4cccc5ccccc54)c1	8.1
175	O=C(c1cn([C@H]([N+])c2cccc(c2C)C)c3ccccc13)c4cccc5ccccc54	8.1
176	Clc1ccnc(Sn2cc(c3ccccc32)C(=O)c4cccc5ccccc54)c1	8.1
177	O=C(c1cn(C[N+](C2CCCC2)C)c3ccccc31)c4cccc5ccccc54	8.1
178	O=C(c1cn(O[C@@H](C2CC2)C#C)c3ccccc13)c4cccc5ccccc54	8.1
179	Clc1cc(Cl)ccc1[C@@H]([N+])n2cc(c3ccccc32)C(=O)c4cccc5ccccc54	8.1
180	O=C(c1cn(C[N+]2CC=CCC2)c3ccccc31)c4cccc5ccccc54	8.1
181	FC(F)C(F)(F)Cn1cc(c2ccccc21)C(=O)c3cccc4ccccc43	8.1
182	Clc1ccccc1[C@H](n2cc(c3ccccc32)C(=O)c4cccc5ccccc54)CC[N+]	8.1
183	O=C(c1cn(c2ccccc21)/C=C(\C3CC3)C)c4cccc5ccccc54	8.1
184	Br/C=C/Cn1cc(c2ccccc21)C(=O)c3cccc4ccccc43	8.1
185	FC(F)(F)/C(=C\n1cc(c2ccccc21)C(=O)c3cccc4ccccc43)C	8.1
186	O=C(c1cn(CC2([N+])CCCCC2)c3ccccc31)c4cccc5ccccc54	8.1
187	Fc1cc(OC)ccc1[C@@H]([N+])n2cc(c3ccccc32)C(=O)c4cccc5ccccc54	8.1
188	Fc1c(cccc1Cn2cc(c3ccccc32)C(=O)c4cccc5ccccc54)C(F)(F)F	8.1
189	FC(F)C[N+](Cn1cc(c2ccccc21)C(=O)c3cccc4ccccc43)C	8.1

190	O=C(c1cn(CC2([N+])CCCC2)c3ccccc31)c4cccc5ccccc54	8.1
191	O=C(c1cn(C[C@@H]2C=CCC2)c3ccccc31)c4cccc5ccccc54	8.1
192	O=C(c1cn(c2ccccc21)Cc3ccoc3C[N+])c4cccc5ccccc54	8.1
193	O=C(c1cn(CC[N+]2CC=CCC2)c3ccccc31)c4cccc5ccccc54	8.1
194	O=C(c1cn(C[N+]2C[C@@H](C[C@@H](C2)C)C)c3ccccc31)c4cccc5ccccc54	8.1
195	O=C(c1cn(C[C@H]2CCCCC[N+]2)c3ccccc31)c4cccc5ccccc54	8.1
196	O=C(c1cn(CC2(CCC2)C)c3ccccc31)c4cccc5ccccc54	8
197	O=C(c1cn(Oc2cc3c(OCO3)cc2C[N+])c4ccccc41)c5cccc6ccccc65	8
198	O=C(c1cn(C[N+]2CCCCC2)c3ccccc31)c4cccc5ccccc54	8
199	S=C(N)[C@@H]1CCC[N+](C1)Cn2cc(c3ccccc32)C(=O)c4cccc5ccccc54	8
200	O=C(c1cn([C@H]([N+])c2ccccc2C)c3ccccc13)c4cccc5ccccc54	8
201	Fc1cc(F)cc(F)c1[C@@H]([N+])n2cc(c3ccccc32)C(=O)c4cccc5ccccc54	8
202	Fc1ccc(F)cc1S(=O)(=O)n2cc(c3ccccc32)C(=O)c4cccc5ccccc54	8
203	O=C(c1cn(C[C@@H]([N+]CCCC)C)c2ccccc21)c3cccc4ccccc43	8
204	Fc1ccc(F)cc1[C@@H]([N+]C)n2cc(c3ccccc32)C(=O)c4cccc5ccccc54	8
205	SCCSn1cc(c2ccccc21)C(=O)c3cccc4ccccc43	8
206	O=C(c1cn(C[N+]CCC)c2ccccc21)c3cccc4ccccc43	8
207	O=C(c1cn(C[C@@H]2C[N+](CC2)C)c3ccccc31)c4cccc5ccccc54	8
208	O=C(c1cn(NCC2CC2)c3ccccc13)c4cccc5ccccc54	8
209	O=C(c1cn(C[C@H]2CCC[C@H]([N+])C2)c3ccccc31)c4cccc5ccccc54	8
210	Fc1cc(F)cc(F)c1[C@@H]([N+]C)n2cc(c3ccccc32)C(=O)c4cccc5ccccc54	8
211	O=C(c1cn(C[N+]2CCCC2)c3ccccc31)c4cccc5ccccc54	8
212	O=C(c1cn(C[N+]2CCCCCCC2)c3ccccc31)c4cccc5ccccc54	8
213	O=C(c1cn(c2ccccc21)Cn3cccc3C[N+])c4cccc5ccccc54	8
214	Clc1ccc([C@@H]([N+])n2cc(c3ccccc32)C(=O)c4cccc5ccccc54)c(F)c1	8
215	O=C(c1cn(C[N+](CCOC)C)c2ccccc21)c3cccc4ccccc43	8
216	O=C(c1cn([C@H]([N+]C)c2cccs2)c3ccccc31)c4cccc5ccccc54	8
217	O=C(c1cn(C[N+]2CCC[C@H]([C@H]2C)C)c3ccccc31)c4cccc5ccccc54	8
218	O=C(c1cn(c2ccccc21)CSCC3CC3)c4cccc5ccccc54	8
219	OCCCSCn1cc(c2ccccc21)C(=O)c3cccc4ccccc43	8
220	O=C(c1cn(c2ccccc21)C[n+]3cc(CC)ccc3C)c4cccc5ccccc54	8
221	O=C(c1cn(C[C@@H]2CCC[C@H](C2)C[N+])c3ccccc31)c4cccc5ccccc54	8
222	O=C(c1cn(CC[C@H]2CCCC[N+]2C)c3ccccc31)c4cccc5ccccc54	8
223	Fc1ccccc1[C@H](n2cc(c3ccccc32)C(=O)c4cccc5ccccc54)C[N+]	8
224	O=C(c1cn(C[C@H]2CCCC[C@H]2[N+]CC)c3ccccc31)c4cccc5ccccc54	8
225	O=C(c1cn(C[C@@H]2[C@H]([N+]CCS2)C)c3ccccc31)c4cccc5ccccc54	8
226	Clc1ccccc1[C@@H]([N+])n2cc(c3ccccc32)C(=O)c4cccc5ccccc54	8
227	BrC(CSn1cc(c2ccccc21)C(=O)c3cccc4ccccc43)=C	8
228	O=C(c1cn(C[C@H]2CCCCC[C@H]2[N+]C)c3ccccc31)c4cccc5ccccc54	7.9
229	O=C(c1cn(c2ccccc21)CSCC)c3cccc4ccccc43	7.9
230	O=C(c1cn(c2ccccc21)Cc3csc(n3)C[N+])c4cccc5ccccc54	7.9
231	Fc1c(F)c(F)ccc1[C@@H]([N+])n2cc(c3ccccc32)C(=O)c4cccc5ccccc54	7.9

232	O=C(c1cn(c2ccccc21)Cc3cccnc3C[N+])c4cccc5ccccc54	7.9
233	Fc1c(On2cc(c3ccccc32)C(=O)c4cccc5ccccc54)cccn1	7.9
234	O=C(c1cn(c2ccccc21)COc3cc(ccc3[C@@H]([N+])C)C)c4cccc5ccccc54	7.9
235	O=C(c1cn(c2ccccc21)Cc3ccccc3C[N+])c4cccc5ccccc54	7.9
236	Fc1ccc(On2cc(c3ccccc32)C(=O)c4cccc5ccccc54)c(c1)C#CC[N+]	7.9
237	O=C(c1cn([C@H](C[N+])c2ccccn2)c3ccccc31)c4cccc5ccccc54	7.9
238	O=C(c1cn(C[C@@H](CC)C#N)c2ccccc21)c3cccc4ccccc43	7.9
239	O=C(c1cn([C@@H](C[N+])c2cscc2)c3ccccc31)c4cccc5ccccc54	7.9
240	O=C(c1cn([C@H](C[N+])c2cccs2)c3ccccc31)c4cccc5ccccc54	7.9
241	O=C(c1cn(C[C@H]([N+])C(C)C)c2ccccc21)c3cccc4ccccc43	7.9
242	O=C(c1cn(CC2([N+]C)CCCC2)c3ccccc31)c4cccc5ccccc54	7.9
243	O=C(c1cn(CC[N+]C(C)(C)C)c2ccccc21)c3cccc4ccccc43	7.9
244	O=C(c1cn(OC2CSC2)c3ccccc31)c4cccc5ccccc54	7.9
245	Fc1ccccc1S(=O)(=O)n2cc(c3ccccc32)C(=O)c4cccc5ccccc54	7.9
246	O=C(c1cn(C[C@H]2Cc3ccccc3C[N+]2C)c4ccccc41)c5cccc6ccccc65	7.9
247	O[C@H]1CCCC[C@@H]1[N+]Cn2cc(c3ccccc32)C(=O)c4cccc5ccccc54	7.9
248	O=C(c1cn(SCCC#C)c2ccccc21)c3cccc4ccccc43	7.9
249	BrC(Br)=Cn1cc(c2ccccc21)C(=O)c3cccc4ccccc43	7.9
250	O=C(c1cn([C@H]([N+])c2ccco2)c3ccccc13)c4cccc5ccccc54	7.9
251	OCC[N+]CCn1cc(c2ccccc21)C(=O)c3cccc4ccccc43	7.9
252	Clc1c(F)cc(F)cc1-n2cc(c3ccccc32)C(=O)c4cccc5ccccc54	7.9
253	FC(F)Cn1cc(c2ccccc21)C(=O)c3cccc4ccccc43	7.9
254	O=C(c1cn(c2ccccc21)CSCC(C)C)c3cccc4ccccc43	7.9
255	Brc1ccccc1[C@@H]([N+])n2cc(c3ccccc32)C(=O)c4cccc5ccccc54	7.9
256	BrC(CCn1cc(c2ccccc21)C(=O)c3cccc4ccccc43)=C	7.9
257	Clc1ccc2c(ccc[n+]2-n3cc(c4ccccc43)C(=O)c5cccc6ccccc65)c1	7.9
258	Fc1ccccc1[C@@H]([N+]C)n2cc(c3ccccc32)C(=O)c4cccc5ccccc54	7.8
259	O=C(c1cn(SCCC)c2ccccc12)c3cccc4ccccc43	7.8
260	O=C(c1cn(CC(CC)CC)c2ccccc21)c3cccc4ccccc43	7.8
261	O=C(c1cn(CC[N+](CCNC)C)c2ccccc21)c3cccc4ccccc43	7.8
262	O=C(c1cn(c2ccccc21)CC(C)C)c3cccc4ccccc43	7.8
263	O=S(=O)(Cn1cc(c2ccccc21)C(=O)c3cccc4ccccc43)CC	7.8
264	O=C(c1cn(C[C@@H]2C[N+](CC2)CC)c3ccccc31)c4cccc5ccccc54	7.8
265	O=C(c1cn(C[N+]2CCCC[C@@H]2C)c3ccccc31)c4cccc5ccccc54	7.8
266	O=C(c1c2ccccc2n(-[n+]3csc4ccccc43)c1)c5cccc6ccccc65	7.8
267	F[C@@H]1C[C@H]([N+]C1)Cn2cc(c3ccccc32)C(=O)c4cccc5ccccc54	7.8
268	O=C(c1cn(C[C@H]([N+]C)C2CC2)c3ccccc31)c4cccc5ccccc54	7.8
269	Fc1cc(O)cc2c1CC[N+][C@@H]2Cn3cc(c4ccccc43)C(=O)c5cccc6ccccc65	7.8
270	O=C(c1cn(CC[N+]CCC)c2ccccc21)c3cccc4ccccc43	7.8
271	O=C(c1cn(Cc2c[nH+]c(N(C)C)n2C)c3ccccc31)c4cccc5ccccc54	7.8
272	SCC1(CC1)Cn2cc(c3ccccc32)C(=O)c4cccc5ccccc54	7.8
273	O=C(c1cn(CC[N+]2CCCC2)c3ccccc31)c4cccc5ccccc54	7.8

274	Fc1cc(ccc1Cn2cc(c3ccccc32)C(=O)c4cccc5ccccc54)C#CC[N+]	7.8
275	O=C(c1cn([C@H]([N+])c2ccccc2OC)c3ccccc31)c4cccc5ccccc54	7.8
276	O=C(c1cn(Sc2ccccc2C[N+])c3ccccc31)c4cccc5ccccc54	7.8
277	O=C(c1cn(CCCCC(N)=[N+])c2ccccc21)c3cccc4ccccc43	7.8
278	O=C(c1cn(C[C@H]2CCC[N+]2)c3ccccc31)c4cccc5ccccc54	7.8
279	O=S(=O)(C(C)C)Cn1cc(c2ccccc21)C(=O)c3cccc4ccccc43	7.8
280	O=C(c1cn(CC[N+]CC#C)c2ccccc21)c3cccc4ccccc43	7.8
281	O=C(c1cn([C@@H]([N+]C)C2CCC2)c3ccccc31)c4cccc5ccccc54	7.8
282	Brc1c(F)cccc1-n2cc(c3ccccc32)C(=O)c4cccc5ccccc54	7.8
283	Fc1ccc(F)cc1[C@@H]([N+])n2cc(c3ccccc32)C(=O)c4cccc5ccccc54	7.8
284	O=C(c1cn(c2ccccc21)CSCCCC[N+])c3cccc4ccccc43	7.8
285	S=C(On1cc(c2ccccc21)C(=O)c3cccc4ccccc43)N(C)C	7.8
286	O=C(c1cn([C@H]([N+])c2ccccc2OCC)c3ccccc31)c4cccc5ccccc54	7.8
287	O=C(c1cn(c2ccccc21)C[S@@](=O)CC)c3cccc4ccccc43	7.8
288	O=C(c1cn(C[C@@H](CC)C)c2ccccc21)c3cccc4ccccc43	7.8
289	ClC(CCn1cc(c2ccccc21)C(=O)c3cccc4ccccc43)=C	7.8
290	O=C(c1cn(CC[N+](C2CC2)C)c3ccccc31)c4cccc5ccccc54	7.8
291	O=C(c1cn(C[C@H](OC)C)c2ccccc21)c3cccc4ccccc43	7.8
292	Clc1ccccc1[C@@H](n2cc(c3ccccc32)C(=O)c4cccc5ccccc54)C[N+]	7.8
293	Fc1ccccc1[C@@H]([N+])n2cc(c3ccccc32)C(=O)c4cccc5ccccc54	7.8
294	O=C(c1cn(C[C@H]2CCCC[C@@H]2C[N+])c3ccccc31)c4cccc5ccccc54	7.8
295	O=C(c1cn(C[C@H](C(C)C)C)c2ccccc21)c3cccc4ccccc43	7.8
296	O=C(c1cn(C[C@@H]2CCC[C@H]2[N+])c3ccccc31)c4cccc5ccccc54	7.8
297	O=C(c1cn(c2ccccc21)Cc3ccoc3C[N+]CC)c4cccc5ccccc54	7.8
298	Clc1c(Cl)cccc1[C@@H]([N+])n2cc(c3ccccc32)C(=O)c4cccc5ccccc54	7.8
299	O=C(c1cn(C[C@H]2CCC[N+]CC2)c3ccccc31)c4cccc5ccccc54	7.8
300	O=C(c1cn(CC2(C3CC3)CC2)c4ccccc41)c5cccc6ccccc65	7.8
301	O=C(c1cn(OC[C@H]2CC[N+]2)c3ccccc13)c4cccc5ccccc54	7.8
302	O=C(c1cn(C[C@H]2CCC[N+]2CC)c3ccccc31)c4cccc5ccccc54	7.8
303	O=C(c1cn([C@H]([N+]C)c2cnccn2)c3ccccc31)c4cccc5ccccc54	7.8
304	O=C(c1cn(Oc2ccc(C[N+])cc2)c3ccccc31)c4cccc5ccccc54	7.7
305	O=C(c1cn([C@@H]([N+])[C@@H](CC)C)c2ccccc12)c3cccc4ccccc43	7.7
306	O=C(c1cn([C@@H]([N+]2CCCC2)C)c3ccccc13)c4cccc5ccccc54	7.7
307	FC(F)(F)C(F)(F)Cn1cc(c2ccccc21)C(=O)c3cccc4ccccc43	7.7
308	Fc1cc(O)ccc1[C@@H]([N+])n2cc(c3ccccc32)C(=O)c4cccc5ccccc54	7.7
309	O=C(c1cn([C@@H]([N+]C)C2CCCC2)c3ccccc31)c4cccc5ccccc54	7.7
310	O=C(c1cn([C@H]([N+])c2cc(oc2C)C)c3ccccc31)c4cccc5ccccc54	7.7
311	O=S(=O)(Cn1cc(c2ccccc21)C(=O)c3cccc4ccccc43)CC#C	7.7
312	FCCSn1cc(c2ccccc21)C(=O)c3cccc4ccccc43	7.7
313	O=C(c1c2ccccc2n([C@@H]([N+])CC(C)C)c1)c3cccc4ccccc43	7.7
314	FC(Sn1cc(c2ccccc21)C(=O)c3cccc4ccccc43)F	7.7
315	O=C(c1cn(C[N+]2CCC[C@H]2C)c3ccccc31)c4cccc5ccccc54	7.7

316	O=C(c1cn(C[C@H]2CCC[C@H]2C[N+])c3ccccc31)c4cccc5ccccc54	7.7
317	Fc1cccc(F)c1[C@H](n2cc(c3ccccc32)C(=O)c4cccc5ccccc54)C[N+]	7.7
318	Fc1c(OC)ccc(F)c1-n2cc(c3ccccc32)C(=O)c4cccc5ccccc54	7.7
319	Fc1ccc(C[N+])cc1Cn2cc(c3ccccc32)C(=O)c4cccc5ccccc54	7.7
320	O=C(c1cn([C@@H]([N+])C2CCCC2)c3ccccc13)c4cccc5ccccc54	7.7
321	O=C(c1cn(CC[N+]CC=C)c2ccccc21)c3cccc4ccccc43	7.7
322	O=C(c1cn(C[C@H]([N+])CC2CC2)c3ccccc31)c4cccc5ccccc54	7.7
323	Clc1cccc(F)c1C[C@@H]([N+])n2cc(c3ccccc32)C(=O)c4cccc5ccccc54	7.7
324	S=C1N=NCN1Cn2cc(c3ccccc32)C(=O)c4cccc5ccccc54	7.7
325	O=C(c1cn(c2ccccc21)C[N+](CC#C)CC#C)c3cccc4ccccc43	7.7
326	O=C(c1cn(c2ccccc21)Cc3cc(c(o3)C)C[N+])c4cccc5ccccc54	7.7
327	ClC(Cl)=Cn1cc(c2ccccc21)C(=O)c3cccc4ccccc43	7.7
328	SC[C@@H]([N+])CCn1cc(c2ccccc21)C(=O)c3cccc4ccccc43	7.7
329	O=C(c1cn(CC2(CC2)CC)c3ccccc31)c4cccc5ccccc54	7.7
330	Clc1cccc(F)c1[C@H](n2cc(c3ccccc32)C(=O)c4cccc5ccccc54)C[N+]	7.7
331	O=C(c1cn(c2ccccc21)CN(OC)C)c3cccc4ccccc43	7.7
332	O=C(c1cn(CCC(C)=C)c2ccccc21)c3cccc4ccccc43	7.7
333	O=C(c1cn([C@H]([N+])c2ccoc2C)c3ccccc13)c4cccc5ccccc54	7.7
334	O=C(c1c2ccccc2n([C@@H]([N+])[C@H]3CCCCO3)c1)c4cccc5ccccc54	7.7
335	Clc1cccc(Cl)c1[C@@H]([N+])n2cc(c3ccccc32)C(=O)c4cccc5ccccc54	7.7
336	O=C(c1cn(C[N+](CC)(CC)C)c2ccccc21)c3cccc4ccccc43	7.7
337	O=C(c1cn(C[N+]2CCCC[C@@H]2CC)c3ccccc31)c4cccc5ccccc54	7.7
338	O=C(c1cn(c2ccccc21)Cc3cc(oc3C)C[N+]C)c4cccc5ccccc54	7.6
339	O=C(c1cn(C[C@H]2CCC[N+]2CCC)c3ccccc31)c4cccc5ccccc54	7.6
340	O=C(n1cc(c2ccccc21)C(=O)c3cccc4ccccc43)[C@H]5CCCC[N+]5CC	7.6
341	FCCOn1cc(c2ccccc21)C(=O)c3cccc4ccccc43	7.6
342	O=C(c1cn(C[N+]2CCSC[C@@H]2C)c3ccccc31)c4cccc5ccccc54	7.6
343	O=C(c1cn(Oc2ccccc2[C@@H]([N+])C)c3ccccc31)c4cccc5ccccc54	7.6
344	O=C(c1cn(Oc2ccccc2C[N+])c3ccccc31)c4cccc5ccccc54	7.6
345	O=C(c1cn(C[C@@H]2COCC[N+]2)c3ccccc31)c4cccc5ccccc54	7.6
346	Fc1cccc(F)c1[C@@H]([N+])n2cc(c3ccccc32)C(=O)c4cccc5ccccc54	7.6
347	O=C(c1cn(C[C@H]2CS2)c3ccccc31)c4cccc5ccccc54	7.6
348	O=C(c1cn(C[C@H](CCC)C)c2ccccc21)c3cccc4ccccc43	7.6
349	SC(=N)CCn1cc(c2ccccc21)C(=O)c3cccc4ccccc43	7.6
350	O=C(c1cn(NC2CC[N+]CC2)c3ccccc31)c4cccc5ccccc54	7.6
351	O=C(c1cn(c2ccccc21)Cc3nc(C[N+]C)cs3)c4cccc5ccccc54	7.6
352	O=C(c1cn(C[C@H]([N+]2CCCC2)CC)c3ccccc31)c4cccc5ccccc54	7.6
353	FC1(F)C[N+](CC1)CCn2cc(c3ccccc32)C(=O)c4cccc5ccccc54	7.6
354	O=C(c1cn(CC[N+](C2CCCCC2)C)c3ccccc31)c4cccc5ccccc54	7.6
355	O=C(c1cn(SCC#C)c2ccccc21)c3cccc4ccccc43	7.6
356	O=C(c1cn(Cc2cc(oc2C)C[N+])c3ccccc31)c4cccc5ccccc54	7.6
357	O=C(c1cn(CC[S+](C)C)c2ccccc12)c3cccc4ccccc43	7.6

358	O=C(c1cn(C[C@H]2C[N+](CCN2)C)c3ccccc31)c4cccc5ccccc54	7.6
359	O[C@H](n1cc(c2ccccc21)C(=O)c3cccc4ccccc43)[C@H]5CCC[N+]5	7.6
360	Clc1ccccc1[C@H](n2cc(c3ccccc32)C(=O)c4cccc5ccccc54)[C@@H]([N+])C	7.6
361	O=C(c1cn(C[C@H]2CCC[C@H]2[N+]C)c3ccccc31)c4cccc5ccccc54	7.6
362	O=C(c1cn(C[C@@H](C[N+]C)C)c2ccccc21)c3cccc4ccccc43	7.6
363	O=C(c1cn(c2ccccc21)C[N+]CC=C)c3cccc4ccccc43	7.6
364	O=C(c1cn(C[N+]2CCC[C@@H](C2)C)c3ccccc31)c4cccc5ccccc54	7.6
365	Clc1cccc(F)c1[C@@H]([N+])n2cc(c3ccccc32)C(=O)c4cccc5ccccc54	7.6
366	O=C(c1cn(c2ccccc21)Cc3cc(c(o3)C[N+])C)c4cccc5ccccc54	7.6
367	BrC(Cn1cc(c2ccccc21)C(=O)c3cccc4ccccc43)=C	7.5
368	O=C(c1cn(CC[C@H]2CCCC[N+]2)c3ccccc31)c4cccc5ccccc54	7.5
369	O=C(c1cn(c2ccccc21)C[S@](=O)C)c3cccc4ccccc43	7.5
370	O=C(c1cn(Cc2c(nc(s2)CC[N+])C)c3ccccc31)c4cccc5ccccc54	7.5
371	Fc1cc(c2CC[N+][C@@H](c2c1)Cn3cc(c4ccccc43)C(=O)c5cccc6ccccc65)C	7.5
372	O=S(=O)(n1cc(c2ccccc21)C(=O)c3cccc4ccccc43)c5ccccc5C[N+]	7.5
373	O=C(c1cn(C[C@@H]([N+](CC)CC)C)c2ccccc21)c3cccc4ccccc43	7.5
374	O=C(c1cn(CC[N+](C2CC2)C(C)C)c3ccccc31)c4cccc5ccccc54	7.5
375	Fc1cccc(On2cc(c3ccccc32)C(=O)c4cccc5ccccc54)c1C[N+]	7.5
376	O=C(c1cn(Sc2c(nccn2)C[N+])c3ccccc31)c4cccc5ccccc54	7.5
377	O=S(=O)(n1cc(c2ccccc21)C(=O)c3cccc4ccccc43)c5ccsc5C[N+]	7.5
378	Fc1c(OC)c(F)ccc1-n2cc(c3ccccc32)C(=O)c4cccc5ccccc54	7.5
379	O=C(c1cn(c2ccccc21)CSCC=C)c3cccc4ccccc43	7.5
380	O=C(c1cn(C[N+]2CCCC[C@@H]2C3OCCO3)c4ccccc41)c5cccc6ccccc65	7.5
381	O=C(c1cn(C[C@H]([N+]CC)CCC)c2ccccc21)c3cccc4ccccc43	7.5
382	Fc1cc2CC[N+][C@@H](c2cc1C)Cn3cc(c4ccccc43)C(=O)c5cccc6ccccc65	7.5
383	O=C(c1cn(C[C@@H]([N+]CC)C)c2ccccc21)c3cccc4ccccc43	7.5
384	O=C(c1cn(C[C@@H]2CO[C@@H](C[N+]2)C)c3ccccc31)c4cccc5ccccc54	7.5
385	Fc1cc(C[N+])ccc1Cn2cc(c3ccccc32)C(=O)c4cccc5ccccc54	7.5
386	O=C(c1cn(C[C@H]2CCCC[N+]2CC)c3ccccc31)c4cccc5ccccc54	7.5
387	O=C(c1cn(C[C@H]2CCC[N+]2CC=C)c3ccccc31)c4cccc5ccccc54	7.5
388	O=C(c1cn(C[C@H]2CNCC[N+]2C)c3ccccc31)c4cccc5ccccc54	7.5
389	O=C(c1cn(Cc2c(nc(s2)C[N+])C)c3ccccc31)c4cccc5ccccc54	7.5
390	O=C(c1cn(CCCCC[N+])c2ccccc21)c3cccc4ccccc43	7.5
391	O=C(n1cc(c2ccccc21)C(=O)c3cccc4ccccc43)[C@H]5CCCC[N+]5C	7.5
392	O=C(c1cn(C[C@@H]([N+]CC)CC)c2ccccc21)c3cccc4ccccc43	7.5
393	ClC(Cn1cc(c2ccccc21)C(=O)c3cccc4ccccc43)=C	7.5
394	O=C(c1cn(CC[N+](C(C)C)C(C)C)c2ccccc21)c3cccc4ccccc43	7.5
395	O[C@@H](n1cc(c2ccccc21)C(=O)c3cccc4ccccc43)[C@H]5CCC[N+]C5	7.5
396	O=C(c1cn(CC[N+]2C[C@@H](CC2)C)c3ccccc31)c4cccc5ccccc54	7.5
397	O=C(c1cn(C[N+]2CCC2)c3ccccc31)c4cccc5ccccc54	7.5
398	O=C(c1cn(C[C@@H]2CCCC[N+]2)c3ccccc31)c4cccc5ccccc54	7.5
399	Clc1ccccc1[C@H]([N+])Cn2cc(c3ccccc32)C(=O)c4cccc5ccccc54	7.5

400	O=C(c1cn(c2ccccc21)Cc3cc(C[N+])co3)c4cccc5ccccc54	7.5
401	O=S(=O)(N1CCC[N+]CC1)n2cc(c3ccccc32)C(=O)c4cccc5ccccc54	7.5
402	O=C(c1cn(C[C@@H]2CCC[N+](C2)C)c3ccccc31)c4cccc5ccccc54	7.4
403	Fc1cc(F)cc2c1CC[N+][C@@H]2Cn3cc(c4ccccc43)C(=O)c5cccc6ccccc65	7.4
404	O[C@@H](n1cc(c2ccccc21)C(=O)c3cccc4ccccc43)[C@@H]5CCCC[N+]5	7.4
405	O=C(c1cn(CC[N+]CC)c2ccccc21)c3cccc4ccccc43	7.4
406	FC(F)CSn1cc(c2ccccc21)C(=O)c3cccc4ccccc43	7.4
407	FC(Cn1cc(c2ccccc21)C(=O)c3cccc4ccccc43)=C	7.4
408	O=C(c1cn(CC[N+](CCCC)C)c2ccccc21)c3cccc4ccccc43	7.4
409	O=C(c1cn(CC[N+]2[C@@H](CC[C@H]2C)C)c3ccccc31)c4cccc5ccccc54	7.4
410	O=C(c1cn(C[C@@H]2C[N+](CC2)CCC)c3ccccc31)c4cccc5ccccc54	7.4
411	O=C(c1cn(C[C@@H]2c3ccccc3CC[N+]2)c4ccccc41)c5cccc6ccccc65	7.4
412	O[C@]1(C[N+]CCC1)Cn2cc(c3ccccc32)C(=O)c4cccc5ccccc54	7.4
413	O=C(c1cn(C[N+](C2CC2)CC)c3ccccc31)c4cccc5ccccc54	7.4
414	O=C(c1cn(C[N+](C2CC2)C)c3ccccc31)c4cccc5ccccc54	7.4
415	O=C(c1cn([C@H]([N+])c2ccccc2C#N)c3ccccc31)c4cccc5ccccc54	7.4
416	O=C(c1cn(CC[N+](CC)C)c2ccccc21)c3cccc4ccccc43	7.4
417	O=C(c1cn(C[C@@H]2COCC[N+]C2)c3ccccc31)c4cccc5ccccc54	7.4
418	O=C(c1cn(C[C@@H]([N+]CCC)C)c2ccccc21)c3cccc4ccccc43	7.4
419	O=C(c1cn(C[N+]2CCCC[C@@H]2C(C)C)c3ccccc31)c4cccc5ccccc54	7.4
420	Fc1cc(F)cc(-n2cc(c3ccccc32)C(=O)c4cccc5ccccc54)c1C	7.4
421	O=C(c1cn(c2ccccc21)CSC)c3cccc4ccccc43	7.4
422	O=C(c1cn(C[C@@H]([N+]C)C(C)C)c2ccccc21)c3cccc4ccccc43	7.4
423	O=C(c1cn(c2ccccc21)COC3CC[N+]CC3)c4cccc5ccccc54	7.3
424	O=C(c1cn([C@@H]([N+])C2CCC2)c3ccccc13)c4cccc5ccccc54	7.3
425	O=C(c1cn(CC2(CCC2)C[N+])c3ccccc31)c4cccc5ccccc54	7.3
426	O=C(c1cn(c2ccccc21)Cc3nc(c(s3)C[N+])C)c4cccc5ccccc54	7.3
427	O=C(c1c2ccccc2n(CCN(C(N)=[N+])C)c1)c3cccc4ccccc43	7.3
428	FC(F)CCn1cc(c2ccccc21)C(=O)c3cccc4ccccc43	7.3
429	Fc1cccc(-n2cc(c3ccccc32)C(=O)c4cccc5ccccc54)c1CC[N+]	7.3
430	Fc1c(-n2cc(c3ccccc32)C(=O)c4cccc5ccccc54)ccc(F)c1C[N+]C	7.3
431	O=C(c1cn(S[C@H](C[N+]C)C)c2ccccc21)c3cccc4ccccc43	7.3
432	Fc1cccc(-n2cc(c3ccccc32)C(=O)c4cccc5ccccc54)c1OC6C[N+]C6	7.3
433	FC(F)(F)CCn1cc(c2ccccc21)C(=O)c3cccc4ccccc43	7.3
434	Fc1c(F)cc(F)cc1-n2cc(c3ccccc32)C(=O)c4cccc5ccccc54	7.3
435	O=C(c1cn(C[C@H]2CCCC[N+]2C)c3ccccc31)c4cccc5ccccc54	7.3
436	O=C(c1cn([C@@H]([N+](CC)CC)C)c2ccccc12)c3cccc4ccccc43	7.3
437	O=C(c1cn(SSCC[N+])c2ccccc21)c3cccc4ccccc43	7.3
438	O=C(c1cn([C@@H]([N+])C(CC)CC)c2ccccc12)c3cccc4ccccc43	7.3
439	O=C(c1cn(C[C@H]([N+](C)C)c2ccco2)c3ccccc31)c4cccc5ccccc54	7.3
440	O=C(c1cn(CC[N+](C)C)c2ccccc12)c3cccc4ccccc43	7.3
441	O=C(c1cn(c2ccccc21)CCC#C)c3cccc4ccccc43	7.3

442	O=C(c1cn(CC([N+](CC)CC)(C)C)c2ccccc21)c3cccc4ccccc43	7.3
443	O=C(c1cn(C[C@H]([N+](CC#C)C)C)c2ccccc21)c3cccc4ccccc43	7.2
444	O=C(c1cn(C[C@H]([N+]C)COC)c2ccccc21)c3cccc4ccccc43	7.2
445	O=C(c1cn(CC[N+]([C@H](CC)C)C)c2ccccc21)c3cccc4ccccc43	7.2
446	O=C(c1cn(CC[N+]2CCC2)c3ccccc31)c4cccc5ccccc54	7.2
447	O=C(c1cn(CC[N+]2C[C@@H]2C)c3ccccc31)c4cccc5ccccc54	7.2
448	O=C(c1cn(c2ccccc21)CO[C@@H](C[N+])C)c3cccc4ccccc43	7.2
449	O[C@@H](CCn1cc(c2ccccc21)C(=O)c3cccc4ccccc43)C[N+]	7.2
450	O=S(=O)(CC[N+])Cn1cc(c2ccccc21)C(=O)c3cccc4ccccc43	7.1
451	O=C(c1cn(C[C@@H]([N+])[C@H](CC)C)c2ccccc21)c3cccc4ccccc43	7.1
452	O=C(c1cn(C[C@@H]([N+](C)C)CN)c2ccccc21)c3cccc4ccccc43	7.1
453	O=C(c1cn([C@@H]([N+]C)C2CC2)c3ccccc31)c4cccc5ccccc54	7.1
454	O=C(c1cn(C[C@@H]([N+])c2cccs2)c3ccccc31)c4cccc5ccccc54	7.1
455	O=C(c1cn(C[C@@H]([N+]C)C)c2ccccc21)c3cccc4ccccc43	7.1
456	O=C(c1cn(C[C@@H]([N+](C)C)C)c2ccccc21)c3cccc4ccccc43	7.1
457	O=C(c1cn(C[C@@H]2C[N+]CC2)c3ccccc31)c4cccc5ccccc54	7.1
458	O=C(c1cn([C@H](C2CCC2)C[N+])c3ccccc31)c4cccc5ccccc54	7.1
459	O=C(c1cn(CC[C@@H](C[N+]C)C)c2ccccc21)c3cccc4ccccc43	7.1
460	O=C(c1cn(c2ccccc21)C[N+](CC=C)C)c3cccc4ccccc43	7.1
461	O=C(c1cn(c2ccccc21)COCC)c3cccc4ccccc43	7.1
462	O=C(c1cn(C[N+](CC)CC)c2ccccc21)c3cccc4ccccc43	7
463	O=C(c1cn([C@@H]([N+])C(C)C)c2ccccc21)c3cccc4ccccc43	7
464	O=C(c1cn(CC[N+]2CCCCC2)c3ccccc31)c4cccc5ccccc54	7
465	O=C(c1cn(C[C@H]([N+](C)C)CC)c2ccccc21)c3cccc4ccccc43	7
466	O=C(c1cn(CC[N+](CCC)C)c2ccccc21)c3cccc4ccccc43	7
467	O=C(c1cn(C[C@H]2CC[N+]2C)c3ccccc31)c4cccc5ccccc54	7
468	O=C(c1cn(CC2C[N+]C2)c3ccccc31)c4cccc5ccccc54	7
469	O=C(c1cn(C[C@@H](C[N+])C)c2ccccc21)c3cccc4ccccc43	7
470	O=C(c1cn(CC[N+]C)c2ccccc21)c3cccc4ccccc43	7
471	O=C(c1cn([C@@H]([N+])[C@H]2C[C@H]2C)c3ccccc13)c4cccc5ccccc54	7
472	O=C(c1cn(C[N+](CC)C)c2ccccc21)c3cccc4ccccc43	6.9
473	O=C(c1cn(CC[N+](CC2CC2)C)c3ccccc31)c4cccc5ccccc54	6.9
474	Fc1c(-n2cc(c3ccccc32)C(=O)c4cccc5ccccc54)ccc(F)c1C[N+]	6.9
475	O=C(c1cn([C@@H]2CNCCC[N+]2CC)c3ccccc13)c4cccc5ccccc54	6.9
476	O=C(c1cn(C[C@@H]([N+]C)C#N)c2ccccc21)c3cccc4ccccc43	6.9
477	O=C(c1cn(c2ccccc21)C[S+](C)C)c3cccc4ccccc43	6.9
478	O=C(c1cn(-[n+]2cscc2C)c3ccccc13)c4cccc5ccccc54	6.9
479	O=S(=O)(N1CC[N+](C[C@@H]1C)C)n2cc(c3ccccc32)C(=O)c4cccc5ccccc54	6.9
480	O=C(c1cn(C[N+]2CC2)c3ccccc31)c4cccc5ccccc54	6.9
481	O=C(c1cn(C[C@H]([C@@H]([N+])C)C)c2ccccc21)c3cccc4ccccc43	6.8
482	O=C(c1cn(CC[N+](CC(C)C)C)c2ccccc21)c3cccc4ccccc43	6.8
483	O=C(c1cn([C@@H]2[C@H](CCC[N+]2)C)c3ccccc31)c4cccc5ccccc54	6.8

484	O=C(c1cn(c2ccccc21)CSCC[N+])c3cccc4ccccc43	6.8
485	O=C(c1cn(C[C@@H]([N+]CC)COC)c2ccccc21)c3cccc4ccccc43	6.8
486	O=C(c1cn(C[C@@H]([N+]C)CC)c2ccccc21)c3cccc4ccccc43	6.8
487	O=C(c1cn(C[C@H](C(N)=[N+])C)c2ccccc21)c3cccc4ccccc43	6.8
488	OC[C@H]([N+]C)Cn1cc(c2ccccc21)C(=O)c3cccc4ccccc43	6.8
489	O=C(c1cn(c2ccccc21)CSCCC[N+])c3cccc4ccccc43	6.7
490	O=C(c1cn(c2ccccc21)C[N+](C)C)c3cccc4ccccc43	6.7
491	O=C(c1cn(C2=CCC[N+](C2)CC)c3ccccc13)c4cccc5ccccc54	6.7
492	O=C(c1cn(CC[N+]2CCC[C@@H]2C)c3ccccc31)c4cccc5ccccc54	6.7
493	O=C(c1cn(c2ccccc21)C[N+](CC#C)C)c3cccc4ccccc43	6.7
494	O=C(c1cn(CC[N+]2CCC[C@H]2CC)c3ccccc31)c4cccc5ccccc54	6.7
495	O=C(c1cn(C[N+]C(C)C)c2ccccc21)c3cccc4ccccc43	6.6
496	O=C(c1cn(CC[N+]2CC2)c3ccccc31)c4cccc5ccccc54	6.6
497	O=C(c1cn(CCCC[N+])c2ccccc21)c3cccc4ccccc43	6.4
498	O[C@H](n1cc(c2ccccc21)C(=O)c3cccc4ccccc43)[C@@H]([N+]C)CC	6.4
499	O=C(c1cn(OC[N+](C)C)c2ccccc12)c3cccc4ccccc43	6.3
500	O=C(c1c2ccccc2n(C3=CCC[N+](C3)C)c1)c4cccc5ccccc54	6.3

Table S12. List, SMILE and predicted pK_i values for Series 2 in CB_2 receptor.

N°	SMILES	Pred pK_i
1	O=C(c1c2ccccn2c(C[C@H]3CCCC[N+]3C)c1C)c4cccc5ccccc54	8.7
2	O=C(c1cc(n2c1cns2)C[C@H]3CCCC[N+]3C)c4cccc5ccccc54	8.6
3	OCc1c(c2ccccc2n1C[C@H]3CCCC[N+]3C)C(=O)c4cccc5ccccc54	8.5
4	O=C(c1c2c(OC)cccc2n(n1)C[C@H]3CCCC[N+]3C)c4cccc5ccccc54	8.5
5	O=C(c1c2c[nH]cc2n(n1)C[C@H]3CCCC[N+]3C)c4cccc5ccccc54	8.5
6	Clc1ccn2c(c(cc2C[C@H]3CCCC[N+]3C)C(=O)c4cccc5ccccc54)c1	8.5
7	O=C(c1cc(n2ccccc12)C[C@H]3CCCC[N+]3C)c4cccc5ccccc54	8.5
8	O=C(c1c2n(c(n1)C[C@H]3CCCC[N+]3C)ccs2)c4cccc5ccccc54	8.5
9	O=C(c1cc(n2CCCc12)C[C@H]3CCCC[N+]3C)c4cccc5ccccc54	8.5
10	O=C(c1c2c(n(n1)C[C@H]3CCCC[N+]3C)cccn2)c4cccc5ccccc54	8.4
11	O=C(c1c2CCCc2n(n1)C[C@H]3CCCC[N+]3C)c4cccc5ccccc54	8.4
12	O=C(c1c2ccccn2c(C[C@H]3CCCC[N+]3C)c1N)c4cccc5ccccc54	8.4
13	O=C(c1c(c(n(n1)C[C@H]2CCCC[N+]2C)C)C)c3cccc4ccccc43	8.4
14	O=C(n1c2c(c(n1)C[C@H]3CCCC[N+]3C)cccn2)c4cccc5ccccc54	8.4
15	O=C(c1cc(n2ccc(cc12)C)C[C@H]3CCCC[N+]3C)c4cccc5ccccc54	8.4
16	O=C(c1c(n(C[C@H]2CCCC[N+]2C)c3ccc(cc13)CC)C)c4cccc5ccccc54	8.4
17	O=C(c1c2ccccc2n(C[C@H]3CCCC[N+]3C)c1C)c4cccc5ccccc54	8.4
18	O=C(c1c2cc(O)ccc2n(C[C@H]3CCCC[N+]3C)c1C)c4cccc5ccccc54	8.3
19	O=C(c1c2-n(scco2)cc([nH]1)C[C@H]3CCCC[N+]3C)c4cccc5ccccc54	8.3
20	O=C(c1c(n(C[C@H]2CCCC[N+]2C)c3ccc(cc13)C)C)c4cccc5ccccc54	8.3
21	O=C(c1c2COCCc2n(n1)C[C@H]3CCCC[N+]3C)c4cccc5ccccc54	8.3

22	O=C(c1c2cccc(O)c2n(C[C@H]3CCCC[N+]3C)c1C)c4cccc5ccccc54	8.3
23	Fc1cccc2c1c(nn2C[C@H]3CCCC[N+]3C)C(=O)c4cccc5ccccc54	8.3
24	O=C(c1cc(C[C@H]2CCCC[N+]2C)cc3c1cc[nH]3)c4cccc5ccccc54	8.3
25	O=C(c1cc(C[C@H]2CCCC[N+]2C)cc3c1ccn3C)c4cccc5ccccc54	8.3
26	O=C(c1c2cc[nH]c2c(o1)C[C@H]3CCCC[N+]3C)c4cccc5ccccc54	8.3
27	O=C(c1cn(C[C@H]2CCCC[N+]2C)c3cccc(c31)C(OC)=O)c4cccc5ccccc54	8.3
28	O=C(c1c2cc(N)ccc2n(n1)C[C@H]3CCCC[N+]3C)c4cccc5ccccc54	8.3
29	O=C(c1c2c(n(n1)C[C@H]3CCCC[N+]3C)ccs2)c4cccc5ccccc54	8.2
30	O=C(c1c2cnsc2c(s1)C[C@H]3CCCC[N+]3C)c4cccc5ccccc54	8.2
31	O=C(c1cc2c(c(C[C@H]3CCCC[N+]3C)c1)csn2)c4cccc5ccccc54	8.2
32	O=C(c1c2CCCCc2n(n1)C[C@H]3CCCC[N+]3C)c4cccc5ccccc54	8.2
33	Clc1c(cc(n1C)C[C@H]2CCCC[N+]2C)C(=O)c3cccc4ccccc43	8.2
34	O=C(c1c2ccc(O)cc2n(n1)C[C@H]3CCCC[N+]3C)c4cccc5ccccc54	8.2
35	O=C(c1cn(C[C@H]2CCCC[N+]2C)c3cccc(OC)c31)c4cccc5ccccc54	8.2
36	O=C(c1cc2CCNc2c(C[C@H]3CCCC[N+]3C)c1)c4cccc5ccccc54	8.2
37	Brc1c(cc(n1C)C[C@H]2CCCC[N+]2C)C(=O)c3cccc4ccccc43	8.2
38	O=C(c1cc(C[C@H]2CCCC[N+]2C)cc3c[nH]cc31)c4cccc5ccccc54	8.2
39	O=C(c1c2n(c(C[C@H]3CCCC[N+]3C)c1)cco2)c4cccc5ccccc54	8.2
40	O=C(c1cc(C[C@H]2CCCC[N+]2C)c(s1)N)c3cccc4ccccc43	8.2
41	O=C(c1c2ccc(cc2n(C[C@H]3CCCC[N+]3C)c1C)C)c4cccc5ccccc54	8.2
42	O=C(c1c2cc(OC)ccc2n(C[C@H]3CCCC[N+]3C)c1C)c4cccc5ccccc54	8.2
43	O=C(c1c(c(c(s1)C[C@H]2CCCC[N+]2C)C)C)c3cccc4ccccc43	8.1
44	O=C(c1cc(C[C@H]2CCCC[N+]2C)cn3cccc13)c4cccc5ccccc54	8.1
45	Clc1cccc2c1c(nn2C[C@H]3CCCC[N+]3C)C(=O)c4cccc5ccccc54	8.1
46	O=C(c1c2c(sc(n2)N)cc(C[C@H]3CCCC[N+]3C)c1)c4cccc5ccccc54	8.1
47	O=C(c1c(N)c(n2CCCc12)C[C@H]3CCCC[N+]3C)c4cccc5ccccc54	8.1
48	O=C(c1cc(C[C@H]2CCCC[N+]2C)cc3c1OCCC3)c4cccc5ccccc54	8.1
49	O=C(c1c2-n(sccs2)c(C[C@H]3CCCC[N+]3C)c[nH]1)c4cccc5ccccc54	8.1
50	O=C(c1c2ccc(OC)cc2n(C[C@H]3CCCC[N+]3C)c1C)c4cccc5ccccc54	8.1
51	O=C(c1cc2c(c[nH]c2c(C[C@H]3CCCC[N+]3C)c1)C)c4cccc5ccccc54	8.1
52	O=C(c1cn(CC2CC2)c(C[C@H]3CCCC[N+]3C)c1)c4cccc5ccccc54	8.1
53	O=C(c1c(OC)c(cc(C[C@H]2CCCC[N+]2C)c1)CC)c3cccc4ccccc43	8.1
54	O=C(c1cc2c(c(C[C@H]3CCCC[N+]3C)c1)c[nH]n2)c4cccc5ccccc54	8.1
55	O=C(c1c2c(OCCO2)c(s1)C[C@H]3CCCC[N+]3C)c4cccc5ccccc54	8.1
56	O=C(c1c(n(C[C@H]2CCCC[N+]2C)c3ccccc13)C=O)c4cccc5ccccc54	8.1
57	O=C1CCCc2c1c(sc2C[C@H]3CCCC[N+]3C)C(=O)c4cccc5ccccc54	8.1
58	O=C(c1cc(n2c1ccs2)C[C@H]3CCCC[N+]3C)c4cccc5ccccc54	8.1
59	O=C(c1cn(n2[nH]ccsn12)C[C@H]3CCCC[N+]3C)c4cccc5ccccc54	8.1
60	O=C(c1c(n(C[C@H]2CCCC[N+]2C)c(c1C)C)N)c3cccc4ccccc43	8.1
61	O=C(c1cc(n(c1C)C)C[C@H]2CCCC[N+]2C)c3cccc4ccccc43	8.1
62	O=C(c1c2-n(sccs2)cc([nH]1)C[C@H]3CCCC[N+]3C)c4cccc5ccccc54	8
63	Fc1ccc2c(c(nn2C[C@H]3CCCC[N+]3C)C(=O)c4cccc5ccccc54)c1	8

64	O=C(c1c(c(n(C[C@H]2CCCC[N+]2C)c1C)C)C)c3cccc4ccccc43	8
65	O=C(c1c(OC)nc(N)c(C[C@H]2CCCC[N+]2C)c1)c3cccc4ccccc43	8
66	S=C1N(N=C(N1C)C[C@H]2CCCC[N+]2C)C(=O)c3cccc4ccccc43	8
67	O=C(c1c2CC(CCc2c(s1)C[C@H]3CCCC[N+]3C)(C)C)c4cccc5ccccc54	8
68	S=C1N(N=C(N1CC)C[C@H]2CCCC[N+]2C)C(=O)c3cccc4ccccc43	8
69	O=C(c1c2n(c(C[C@H]3CCCC[N+]3C)c1)ccs2)c4cccc5ccccc54	8
70	O=C(c1cn(C[C@H]2CCCC[N+]2C)c3c[nH]cc13)c4cccc5ccccc54	8
71	O=C(c1cc(C[C@H]2CCCC[N+]2C)ccc1S([O-])(=O)=O)c3cccc4ccccc43	8
72	O=C(c1cc2CCCNc2c(C[C@H]3CCCC[N+]3C)c1)c4cccc5ccccc54	8
73	O=C(c1cn(C[C@H]2CCCC[N+]2C)c3c1cc[nH]3)c4cccc5ccccc54	8
74	O=C(N1C(N(C[C@H]2CCCC[N+]2C)c3ccccc31)=N)c4cccc5ccccc54	8
75	O=C(c1cn(C[C@H]2CCCC[N+]2C)c3C=CNC(=O)c31)c4cccc5ccccc54	8
76	FC(F)(F)Cn1c(c(cc1C[C@H]2CCCC[N+]2C)C(=O)c3cccc4ccccc43)C	8
77	O=C(c1cc(C[C@H]2CCCC[N+]2C)cc3c1nccc3C)c4cccc5ccccc54	8
78	O=C(c1cc(n2ccncc12)C[C@H]3CCCC[N+]3C)c4cccc5ccccc54	8
79	O=C(c1c2ccsc2n(n1)C[C@H]3CCCC[N+]3C)c4cccc5ccccc54	8
80	FC(F)Oc1ccc2c(c(nn2C[C@H]3CCCC[N+]3C)C(=O)c4cccc5ccccc54)c1	8
81	Clc1ccc2c(c(nn2C[C@H]3CCCC[N+]3C)C(=O)c4cccc5ccccc54)c1	8
82	O=C(c1cc(C[C@H]2CCCC[N+]2C)c3ccnnn31)c4cccc5ccccc54	8
83	O=C(c1c2C[C@@H]3C[C@@H]3c2n(n1)C[C@H]4CCCC[N+]4C)c5cccc6ccccc65	8
84	Clc1c(nn(C[C@H]2CCCC[N+]2C)c1C)C(=O)c3cccc4ccccc43	8
85	O=C(c1cc(n(c1C)CC)C[C@H]2CCCC[N+]2C)c3cccc4ccccc43	8
86	O=C(c1c2cnsc2n(n1)C[C@H]3CCCC[N+]3C)c4cccc5ccccc54	8
87	O=C(c1c2ccc(cc2n(n1)C[C@H]3CCCC[N+]3C)C)c4cccc5ccccc54	8
88	O=C(c1cc(C[C@H]2CCCC[N+]2C)cc3c1ccn3CC)c4cccc5ccccc54	8
89	O=C(c1cc(C[C@H]2CCCC[N+]2C)cc3c1nc(O)cc3C)c4cccc5ccccc54	7.9
90	O=C(c1c2ccccc2c(o1)C[C@H]3CCCC[N+]3C)c4cccc5ccccc54	7.9
91	O=C(n1c2C(SC=Cc2c(n1)C[C@H]3CCCC[N+]3C)=O)c4cccc5ccccc54	7.9
92	O=C(c1c2cc(ccc2n(n1)C[C@H]3CCCC[N+]3C)C)c4cccc5ccccc54	7.9
93	Clc1ccc2c(c(n(C[C@H]3CCCC[N+]3C)c2c1)C)C(=O)c4cccc5ccccc54	7.9
94	Oc1c(c2ccccc2cc1C[C@H]3CCCC[N+]3C)C(=O)c4cccc5ccccc54	7.9
95	O=C(c1c2c(cc(C[C@H]3CCCC[N+]3C)c1)csn2)c4cccc5ccccc54	7.9
96	Clc1c(OC)c(cc(C[C@H]2CCCC[N+]2C)c1)C(=O)c3cccc4ccccc43	7.9
97	O=C(c1c2c(cc(C[C@H]3CCCC[N+]3C)c1)ccc(O)n2)c4cccc5ccccc54	7.9
98	O=C(c1cc(n2C=CSC(=O)c12)C[C@H]3CCCC[N+]3C)c4cccc5ccccc54	7.9
99	O=C(c1c2c(c(o1)C[C@H]3CCCC[N+]3C)ccs2)c4cccc5ccccc54	7.9
100	O=C(c1c2ccccc2n(n1)C[C@H]3CCCC[N+]3C)c4cccc5ccccc54	7.9
101	Clc1ccc2c(nn(C[C@H]3CCCC[N+]3C)c2c1)C(=O)c4cccc5ccccc54	7.9
102	O=C(c1cc(c(N)c(C[C@H]2CCCC[N+]2C)c1)C(=O)N)c3cccc4ccccc43	7.9
103	O=C(c1cc(C[C@H]2CCCC[N+]2C)c3C=CSC(=O)n31)c4cccc5ccccc54	7.9
104	Clc1c(c2cc(Cl)ccc2n1C[C@H]3CCCC[N+]3C)C(=O)c4cccc5ccccc54	7.9
105	O=C(c1c2cocc2n(n1)C[C@H]3CCCC[N+]3C)c4cccc5ccccc54	7.9

106	O=C(c1c2cnsc2c(o1)C[C@H]3CCCC[N+]3C)c4cccc5ccccc54	7.9
107	O=C(c1cn(C[C@H]2CCCC[N+]2C)c3C=COC(=O)c31)c4cccc5ccccc54	7.9
108	Fc1c(OCC)c(cc(C[C@H]2CCCC[N+]2C)c1)C(=O)c3cccc4ccccc43	7.9
109	Clc1c(Cl)c(nn1C[C@H]2CCCC[N+]2C)C(=O)c3cccc4ccccc43	7.9
110	O=C(c1cn(n2n1scns2)C[C@H]3CCCC[N+]3C)c4cccc5ccccc54	7.9
111	O=C(c1cn(C[C@H]2CCCC[N+]2C)c3CCCc13)c4cccc5ccccc54	7.9
112	O=C(c1c2cccn2n(n1)C[C@H]3CCCC[N+]3C)c4cccc5ccccc54	7.9
113	O=C(c1c2cscc2n(n1)C[C@H]3CCCC[N+]3C)c4cccc5ccccc54	7.9
114	O=C(c1c2c(c(s1)C[C@H]3CCCC[N+]3C)ccs2)c4cccc5ccccc54	7.9
115	Clc1c(c2cc(OC)ccc2n1C[C@H]3CCCC[N+]3C)C(=O)c4cccc5ccccc54	7.9
116	Clc1c(N)cc(C[C@H]2CCCC[N+]2C)cc1C(=O)c3cccc4ccccc43	7.8
117	O=C(c1c2cnccc2n(n1)C[C@H]3CCCC[N+]3C)c4cccc5ccccc54	7.8
118	O=C(c1cc2c(c([nH]c2c(C[C@H]3CCCC[N+]3C)c1)C)C)c4cccc5ccccc54	7.8
119	O=C(c1c2cc([N+]([O-])=O)ccc2n(n1)C[C@H]3CCCC[N+]3C)c4cccc5ccccc54	7.8
120	O=C1N(c2cscc2N1C[C@H]3CCCC[N+]3C)C(=O)c4cccc5ccccc54	7.8
121	O=C(c1cn(C[C@H]2CCCC[N+]2C)cc3-n1occs3)c4cccc5ccccc54	7.8
122	O=C(c1cn(C[C@H]2CCCC[N+]2C)c3C=CSC(=O)c31)c4cccc5ccccc54	7.8
123	O=C(c1c-2[nH]ccsn2c(C[C@H]3CCCC[N+]3C)c[nH]1)c4cccc5ccccc54	7.8
124	Brc1cc(cc(C[C@H]2CCCC[N+]2C)c1NC)C(=O)c3cccc4ccccc43	7.8
125	O=C(c1cc(n2cc[nH]c12)C[C@H]3CCCC[N+]3C)c4cccc5ccccc54	7.8
126	O=C(c1c2cscc2cc(C[C@H]3CCCC[N+]3C)c1)c4cccc5ccccc54	7.8
127	O=C(c1c2cc[nH]c2c([nH]1)C[C@H]3CCCC[N+]3C)c4cccc5ccccc54	7.8
128	Fc1c(c(cc(C[C@H]2CCCC[N+]2C)c1)C(=O)c3cccc4ccccc43)C=O	7.8
129	Oc1c(cc(C[C@H]2CCCC[N+]2C)cc1CO)C(=O)c3cccc4ccccc43	7.8
130	O=C1C(=C2C(SC=CS2)=C1C[C@H]3CCCC[N+]3C)C(=O)c4cccc5ccccc54	7.8
131	O=C(c1c2cocc2cc(C[C@H]3CCCC[N+]3C)c1)c4cccc5ccccc54	7.8
132	O=C(c1cn(C[C@H]2CCCC[N+]2C)c3csnc31)c4cccc5ccccc54	7.8
133	O=C(c1c2C(SC=Cc2c(s1)C[C@H]3CCCC[N+]3C)=O)c4cccc5ccccc54	7.8
134	Oc1c(N)cc(cc1C[C@H]2CCCC[N+]2C)C(=O)c3cccc4ccccc43	7.8
135	O=C(c1c2ccccc2c(s1)C[C@H]3CCCC[N+]3C)c4cccc5ccccc54	7.8
136	Clc1c(c2ccccc2n1C[C@H]3CCCC[N+]3C)C(=O)c4cccc5ccccc54	7.8
137	O=C(c1cn(C[C@H]2CCCC[N+]2C)cc3-n1sccs3)c4cccc5ccccc54	7.8
138	O=C(c1c2C(SC=Cc2c(o1)C[C@H]3CCCC[N+]3C)=O)c4cccc5ccccc54	7.8
139	Clc1cc(cc(C[C@H]2CCCC[N+]2C)c1NC)C(=O)c3cccc4ccccc43	7.8
140	O=C(c1c2C=CCCc2c(s1)C[C@H]3CCCC[N+]3C)c4cccc5ccccc54	7.8
141	O=C(c1cn(c(C[C@H]2CCCC[N+]2C)c1)C)c3cccc4ccccc43	7.8
142	O=C(c1c2c(c([nH]1)C[C@H]3CCCC[N+]3C)cccn2)c4cccc5ccccc54	7.8
143	O=C(c1c2c(n[nH]n2)cc(C[C@H]3CCCC[N+]3C)c1)c4cccc5ccccc54	7.8
144	Clc1c(c2c(cc(cc2n1C[C@H]3CCCC[N+]3C)C)C)C(=O)c4cccc5ccccc54	7.8
145	O=C(c1c2c(c(s1)C[C@H]3CCCC[N+]3C)cc[nH]2)c4cccc5ccccc54	7.8
146	O=C(c1c2c(scn2)cc(C[C@H]3CCCC[N+]3C)c1)c4cccc5ccccc54	7.8
147	Clc1c(c2cc(CC)ccc2n1C[C@H]3CCCC[N+]3C)C(=O)c4cccc5ccccc54	7.8

148	O=C(c1c2c(cc(C[C@H]3CCCC[N+]3C)c1)cncn2)c4cccc5ccccc54	7.8
149	Fc1cccc2c1c(cn2C[C@H]3CCCC[N+]3C)C(=O)c4cccc5ccccc54	7.8
150	O=C(c1cc(n(c1C)CC=C)C[C@H]2CCCC[N+]2C)c3cccc4ccccc43	7.8
151	O=C(c1c2c(cc(C[C@H]3CCCC[N+]3C)c1)cco2)c4cccc5ccccc54	7.8
152	O=C(c1c2cc(ccc2n(n1)C[C@H]3CCCC[N+]3C)C#N)c4cccc5ccccc54	7.8
153	O=C(c1cn(C[C@H]2CCCC[N+]2C)c3cccc(c31)C)c4cccc5ccccc54	7.8
154	O=C(c1cn(C[C@H]2CCCC[N+]2C)cc3-n1scns3)c4cccc5ccccc54	7.8
155	Clc1ccc2c(c(c(n2C[C@H]3CCCC[N+]3C)C)C(=O)c4cccc5ccccc54)c1	7.8
156	Clc1cccc2c1n(nc2C[C@H]3CCCC[N+]3C)C(=O)c4cccc5ccccc54	7.8
157	O=C(c1cn(C[C@H]2CCCC[N+]2C)cc3-n1scco3)c4cccc5ccccc54	7.8
158	O=C(c1c2ccoc2cc(C[C@H]3CCCC[N+]3C)c1)c4cccc5ccccc54	7.8
159	Clc1c(OCC)c(cc(C[C@H]2CCCC[N+]2C)c1)C(=O)c3cccc4ccccc43	7.8
160	O=C(c1c2-n(occo2)cc([nH]1)C[C@H]3CCCC[N+]3C)c4cccc5ccccc54	7.8
161	O=C(c1cn(C[C@H]2CCCC[N+]2C)c(n1)CCC)c3cccc4ccccc43	7.8
162	Fc1ccc2c(c(n(C[C@H]3CCCC[N+]3C)c2c1)C)C(=O)c4cccc5ccccc54	7.7
163	O=C(c1cc2cc[nH]c2c(C[C@H]3CCCC[N+]3C)c1)c4cccc5ccccc54	7.7
164	O=C(c1cc(C[C@H]2CCCC[N+]2C)cc3c1cc(o3)C)c4cccc5ccccc54	7.7
165	Clc1cccc2c1c(cn2C[C@H]3CCCC[N+]3C)C(=O)c4cccc5ccccc54	7.7
166	O[C@@H]1CCCc2c1c(nn2C[C@H]3CCCC[N+]3C)C(=O)c4cccc5ccccc54	7.7
167	O=C(c1c2c(n(n1)C[C@H]3CCCC[N+]3C)cns2)c4cccc5ccccc54	7.7
168	Clc1c(c2cc(ccc2n1C[C@H]3CCCC[N+]3C)C)C(=O)c4cccc5ccccc54	7.7
169	O[C@@H](c1cc(cc(C[C@H]2CCCC[N+]2C)c1)C(=O)c3cccc4ccccc43)C	7.7
170	O=C(c1cn(C[C@H]2CCCC[N+]2C)c3c1ncs3)c4cccc5ccccc54	7.7
171	Oc1c(cc(C[C@H]2CCCC[N+]2C)cc1C)C(=O)c3cccc4ccccc43	7.7
172	O=C(c1c2cc3c(OCO3)cc2n(C[C@H]4CCCC[N+]4C)c1C)c5cccc6ccccc65	7.7
173	O=C(c1cn(C[C@H]2CCCC[N+]2C)c3cccnc31)c4cccc5ccccc54	7.7
174	O=C(c1c2c(ocn2)cc(C[C@H]3CCCC[N+]3C)c1)c4cccc5ccccc54	7.7
175	Fc1c(cc(C[C@H]2CCCC[N+]2C)cc1CO)C(=O)c3cccc4ccccc43	7.7
176	Oc1c(C(=O)c2cccc3ccccc32)cc(N)c(O)c1C[C@H]4CCCC[N+]4C	7.7
177	O=C(c1cn(C[C@H]2CCCC[N+]2C)c3ccoc31)c4cccc5ccccc54	7.7
178	O=C1C(=C2C(OC=CS2)=C1C[C@H]3CCCC[N+]3C)C(=O)c4cccc5ccccc54	7.7
179	O=C(c1cn(C[C@H]2CCCC[N+]2C)c3c(OC)cccc13)c4cccc5ccccc54	7.7
180	O=C(c1c2cncn2n(n1)C[C@H]3CCCC[N+]3C)c4cccc5ccccc54	7.7
181	O=C(c1c(c(n(C[C@H]2CCCC[N+]2C)c1)N)C#N)c3cccc4ccccc43	7.7
182	O=C(c1c2c(cc(C[C@H]3CCCC[N+]3C)c1)ccs2)c4cccc5ccccc54	7.7
183	O=C(n1cc(C[C@H]2CCCC[N+]2C)c3C=COC(=O)c31)c4cccc5ccccc54	7.7
184	O=C(C=1CC=CN(C[C@H]2CCCC[N+]2C)C1)c3cccc4ccccc43	7.7
185	O=C(C1=CC=CN(C[C@H]2CCCC[N+]2C)C1)c3cccc4ccccc43	7.7
186	O=C(c1c2ccncn2c(C[C@H]3CCCC[N+]3C)c1)c4cccc5ccccc54	7.7
187	Brc1c(c(sc1C[C@H]2CCCC[N+]2C)C(=O)c3cccc4ccccc43)C	7.7
188	Brc1c(OC)c(cc(C[C@H]2CCCC[N+]2C)c1)C(=O)c3cccc4ccccc43	7.7
189	O=C(c1cc(C[C@H]2CCCC[N+]2C)c3n1cns3)c4cccc5ccccc54	7.7

190	O=C(c1c-2[nH]ccsn2cc([nH]1)C[C@H]3CCCC[N+]3C)c4cccc5ccccc54	7.7
191	O=C(c1c2ccsc2n(C[C@H]3CCCC[N+]3C)c1C)c4cccc5ccccc54	7.7
192	Fc1ccc2c(nn(C[C@H]3CCCC[N+]3C)c2c1)C(=O)c4cccc5ccccc54	7.7
193	Clc1cc(c(F)c(C[C@H]2CCCC[N+]2C)c1)C(=O)c3cccc4ccccc43	7.7
194	Fc1c(SC)c(cc(C[C@H]2CCCC[N+]2C)c1)C(=O)c3cccc4ccccc43	7.7
195	O=C(c1cc2c([nH]cn2)c(C[C@H]3CCCC[N+]3C)c1)c4cccc5ccccc54	7.7
196	Clc1c(OC)c(O)c(C[C@H]2CCCC[N+]2C)cc1C(=O)c3cccc4ccccc43	7.7
197	O=C(c1cn(C[C@H]2CCCC[N+]2C)c3ccc(O)cc31)c4cccc5ccccc54	7.6
198	O=C(c1c2c(cc(cc2n(C[C@H]3CCCC[N+]3C)c1)C)C)c4cccc5ccccc54	7.6
199	Brc1c(O)c(cc(C[C@H]2CCCC[N+]2C)c1O)C(=O)c3cccc4ccccc43	7.6
200	O=C(c1cc(C[C@H]2CCCC[N+]2C)c3ccc4ccccc4n13)c5cccc6ccccc65	7.6
201	Clc1c(cc(C[C@H]2CCCC[N+]2C)cc1C)C(=O)c3cccc4ccccc43	7.6
202	O=C(c1c(sc(C[C@H]2CCCC[N+]2C)c1)SC)c3cccc4ccccc43	7.6
203	O=C(c1c2c(OCO2)cc(C[C@H]3CCCC[N+]3C)c1)c4cccc5ccccc54	7.6
204	O=C(c1c2ccccc2cc(C[C@H]3CCCC[N+]3C)c1)c4cccc5ccccc54	7.6
205	O=C(c1cn(n2n1occs2)C[C@H]3CCCC[N+]3C)c4cccc5ccccc54	7.6
206	Clc1c(c2ccc(Cl)cc2n1C[C@H]3CCCC[N+]3C)C(=O)c4cccc5ccccc54	7.6
207	O=C(c1c2c(n(C[C@H]3CCCC[N+]3C)c1)cc(cn2)C)c4cccc5ccccc54	7.6
208	Clc1ccc(C[C@H]2CCCC[N+]2C)cc1C(=O)c3cccc4ccccc43	7.6
209	O=C(c1cc(C[C@H]2CCCC[N+]2C)c3cccc(n13)C)c4cccc5ccccc54	7.6
210	Clc1c(OC)cc2c(n(C[C@H]3CCCC[N+]3C)cc2C(=O)c4cccc5ccccc54)c1	7.6
211	O=C(n1cc(C[C@H]2CCCC[N+]2C)c3C=CNC(=O)c31)c4cccc5ccccc54	7.6
212	O=C(c1nn(C[C@H]2CCCC[N+]2C)c3cccn13)c4cccc5ccccc54	7.6
213	O=C(c1cc(n(C2CC2)c1)C[C@H]3CCCC[N+]3C)c4cccc5ccccc54	7.6
214	Clc1c(O)c(cc(C[C@H]2CCCC[N+]2C)c1O)C(=O)c3cccc4ccccc43	7.6
215	Clc1c(c2cc(F)ccc2n1C[C@H]3CCCC[N+]3C)C(=O)c4cccc5ccccc54	7.6
216	Clc1c(c2ccc(cc2n1C[C@H]3CCCC[N+]3C)C)C(=O)c4cccc5ccccc54	7.6
217	Clc1c(cc(C[C@H]2CCCC[N+]2C)cc1CC)C(=O)c3cccc4ccccc43	7.6
218	Clc1c(c2cc(c(cc2n1C[C@H]3CCCC[N+]3C)C)C)C(=O)c4cccc5ccccc54	7.6
219	O=C(c1cc(C[C@H]2CCCC[N+]2C)cc3c1OCCO3)c4cccc5ccccc54	7.6
220	O=S(=O)(N)c1cc(cc(C[C@H]2CCCC[N+]2C)c1)C(=O)c3cccc4ccccc43	7.6
221	Clc1c(Cl)c(sc1C[C@H]2CCCC[N+]2C)C(=O)c3cccc4ccccc43	7.6
222	O=C(c1cc(n(C[C@H]2CCCC[N+]2C)c1)C)c3cccc4ccccc43	7.6
223	O=C(c1cc(C[C@H]2CCCC[N+]2C)cc(N(C)C)c1)c3cccc4ccccc43	7.6
224	O=C(c1cn(C[C@H]2CCCC[N+]2C)cc3-n1occo3)c4cccc5ccccc54	7.6
225	Fc1cccc2c(c(n(C[C@H]3CCCC[N+]3C)c12)C)C(=O)c4cccc5ccccc54	7.6
226	Fc1c(N)cc(C[C@H]2CCCC[N+]2C)cc1C(=O)c3cccc4ccccc43	7.6
227	O=C(c1c(n(C[C@H]2CCCC[N+]2C)c3c(cccc13)C)C)c4cccc5ccccc54	7.6
228	O=C(c1cn(C[C@H]2CCCC[N+]2C)c3cccc(O)c31)c4cccc5ccccc54	7.6
229	O=C(c1cn(C[C@H]2CCCC[N+]2C)c3cc(OC)cc(OC)c31)c4cccc5ccccc54	7.6
230	O=C(c1c2cnccc2c(s1)C[C@H]3CCCC[N+]3C)c4cccc5ccccc54	7.6
231	O=C(c1cn(C[C@H]2CCCC[N+]2C)c3c1C(=O)C=CS3)c4cccc5ccccc54	7.6

232	Oc1c(c2cc(ccc2n1C[C@H]3CCCC[N+]3C)C)C(=O)c4cccc5ccccc54	7.6
233	O=C(c1cn(C[C@H]2CCCC[N+]2C)c3cc[nH]c31)c4cccc5ccccc54	7.6
234	Fc1ccc2c(c(c(n2C[C@H]3CCCC[N+]3C)C)C(=O)c4cccc5ccccc54)c1	7.6
235	OCCc1c(C[C@H]2CCCC[N+]2C)cc(s1)C(=O)c3cccc4ccccc43	7.5
236	O=C(c1cc[n+](C[C@H]2CCCC[N+]2C)c3cc(N)ccc13)c4cccc5ccccc54	7.5
237	Clc1c(c(cc(C[C@H]2CCCC[N+]2C)c1)C(=O)c3cccc4ccccc43)C=O	7.5
238	O=C(c1cn(C[C@H]2CCCC[N+]2C)c3ccc(cc31)-c4ccco4)c5cccc6ccccc65	7.5
239	O=C(C=1CCCN(C[C@H]2CCCC[N+]2C)C1)c3cccc4ccccc43	7.5
240	Clc1c(O)c(cc(C[C@H]2CCCC[N+]2C)c1)C(=O)c3cccc4ccccc43	7.5
241	O=C(c1cc(S([O-])(=O)=O)cc(C[C@H]2CCCC[N+]2C)c1)c3cccc4ccccc43	7.5
242	Clc1c(Cl)c(O)c(C[C@H]2CCCC[N+]2C)cc1C(=O)c3cccc4ccccc43	7.5
243	O=C(c1cn(C[C@H]2CCCC[N+]2C)c3c1cns3)c4cccc5ccccc54	7.5
244	Fc1cc(O)c(C[C@H]2CCCC[N+]2C)cc1C(=O)c3cccc4ccccc43	7.5
245	O=C(C1=CN(C[C@H]2CCCC[N+]2C)C3=CSC(=O)N31)c4cccc5ccccc54	7.5
246	O=C(c1c2ccncc2n(n1)C[C@H]3CCCC[N+]3C)c4cccc5ccccc54	7.5
247	O=C(c1cn(C[C@H]2CCCC[N+]2C)c3cocc31)c4cccc5ccccc54	7.5
248	O=C(c1c2ccncc2c(o1)C[C@H]3CCCC[N+]3C)c4cccc5ccccc54	7.5
249	O=C(c1cn(C[C@H]2CCCC[N+]2C)c3ccc(cc31)CC)c4cccc5ccccc54	7.5
250	O=C(n1cc2-n([nH]ccs2)c(C[C@H]3CCCC[N+]3C)c1)c4cccc5ccccc54	7.5
251	Fc1c(cc(C[C@H]2CCCC[N+]2C)cc1C)C(=O)c3cccc4ccccc43	7.5
252	O=S1(=O)N(c2ccccc2N1C[C@H]3CCCC[N+]3C)C(=O)c4cccc5ccccc54	7.5
253	Clc1c(Cl)cc(C[C@H]2CCCC[N+]2C)c(O)c1C(=O)c3cccc4ccccc43	7.5
254	Fc1ccc(C[C@H]2CCCC[N+]2C)cc1C(=O)c3cccc4ccccc43	7.5
255	Brc1cc(cc(C[C@H]2CCCC[N+]2C)c1)C(=O)c3cccc4ccccc43	7.5
256	O=C(c1c2ccc(OC)cc2n(n1)C[C@H]3CCCC[N+]3C)c4cccc5ccccc54	7.5
257	O=C(c1cn(C[C@H]2CCCC[N+]2C)c3ccccc31)c4cccc5ccccc54	7.5
258	Clc1ccc2c(c(cn2C[C@H]3CCCC[N+]3C)C(=O)c4cccc5ccccc54)c1	7.5
259	O=C(c1cc(C[C@H]2CCCC[N+]2C)cc3cccn31)c4cccc5ccccc54	7.5
260	O=C(c1cn(C[C@H]2CCCC[N+]2C)cc3-n1onco3)c4cccc5ccccc54	7.5
261	O=C(c1cn(C[C@H]2CCCC[N+]2C)c3ccsc31)c4cccc5ccccc54	7.5
262	O=C(c1cc(C[C@H]2CCCC[N+]2C)cc(n1)C)c3cccc4ccccc43	7.5
263	Clc1cc(c(c(C[C@H]2CCCC[N+]2C)c1)C)C(=O)c3cccc4ccccc43	7.5
264	O=C(c1cn(n2cccc12)C[C@H]3CCCC[N+]3C)c4cccc5ccccc54	7.5
265	Fc1ccc(OC[C@H]2CCCC[N+]2C)c(CC(=O)c3cccc4ccccc43)c1	7.5
266	O=C(c1cc(C[C@H]2CCCC[N+]2C)cc3c1OCC3)c4cccc5ccccc54	7.5
267	Brc1cc(c(F)c(C[C@H]2CCCC[N+]2C)c1)C(=O)c3cccc4ccccc43	7.5
268	Fc1c(F)cc(C[C@H]2CCCC[N+]2C)c(O)c1C(=O)c3cccc4ccccc43	7.5
269	O=C(c1cn(n2n1scco2)C[C@H]3CCCC[N+]3C)c4cccc5ccccc54	7.5
270	Oc1c(C(=O)c2cccc3ccccc32)cc(cc1C[C@H]4CCCC[N+]4C)C	7.5
271	O=C(c1c(N)c(C[C@H]2CCCC[N+]2C)c(s1)C)c3cccc4ccccc43	7.5
272	Clc1c(C(=O)c2cccc3ccccc32)cc(Cl)cc1C[C@H]4CCCC[N+]4C	7.5
273	O=C(c1c2n(ncs2)c(C[C@H]3CCCC[N+]3C)c1)c4cccc5ccccc54	7.5

274	O=C(c1c2C(=O)NC=Cc2c([nH]1)C[C@H]3CCCC[N+]3C)c4cccc5ccccc54	7.5
275	O=C(c1cn(C[C@H]2CCCC[N+]2C)c3ccc(C(C)(C)C)cc13)c4cccc5ccccc54	7.5
276	O=C(c1cn(C[C@H]2CCCC[N+]2C)cc3-n1onno3)c4cccc5ccccc54	7.5
277	O=C(c1c2C(=O)C=CSc2c(s1)C[C@H]3CCCC[N+]3C)c4cccc5ccccc54	7.5
278	Fc1ccc(C[C@H]2CCCC[N+]2C)c(O)c1C(=O)c3cccc4ccccc43	7.5
279	Oc1c(cc(OC)cc1C[C@H]2CCCC[N+]2C)C(=O)c3cccc4ccccc43	7.5
280	O=C(c1c(n(C[C@H]2CCCC[N+]2C)c3c(CC)cccc13)C)c4cccc5ccccc54	7.5
281	Oc1c(CC)cc(cc1C[C@H]2CCCC[N+]2C)C(=O)c3cccc4ccccc43	7.5
282	FC(F)c1cc(nn1C[C@H]2CCCC[N+]2C)C(=O)c3cccc4ccccc43	7.5
283	O=C(c1c2c(nccn2)cc(C[C@H]3CCCC[N+]3C)c1)c4cccc5ccccc54	7.5
284	O=C(c1c2c(ncnn2)cc(C[C@H]3CCCC[N+]3C)c1)c4cccc5ccccc54	7.5
285	O=C(c1cn(C[C@H]2CCCC[N+]2C)c3ccc(cc31)C)c4cccc5ccccc54	7.5
286	O=C(c1cn(C[C@H]2CCCC[N+]2C)c3ccc(OC)cc31)c4cccc5ccccc54	7.5
287	O=C(c1c(N#C)ccc(C[C@H]2CCCC[N+]2C)c1)c3cccc4ccccc43	7.4
288	Clc1c(F)c(cc(C[C@H]2CCCC[N+]2C)c1)C(=O)c3cccc4ccccc43	7.4
289	O=C(c1cn(C[C@H]2CCCC[N+]2C)c3cscc31)c4cccc5ccccc54	7.4
290	O=C(c1cn(C[C@H]2CCCC[N+]2C)c3c1SC(=O)N3)c4cccc5ccccc54	7.4
291	O=C(c1c2C(OC=Cc2c([nH]1)C[C@H]3CCCC[N+]3C)=O)c4cccc5ccccc54	7.4
292	O=C(c1cn(C[C@H]2CCCC[N+]2C)c3cc4c(OCO4)cc31)c5cccc6ccccc65	7.4
293	O=C(n1cc(C[C@H]2CCCC[N+]2C)c3C=CSC(=O)c31)c4cccc5ccccc54	7.4
294	O=C(c1cc(n2cccc2n1)C[C@H]3CCCC[N+]3C)c4cccc5ccccc54	7.4
295	Clc1cc(C[C@H]2CCCC[N+]2C)cc(n1)C(=O)c3cccc4ccccc43	7.4
296	FC(F)(F)c1ccc2c(c(cn2C[C@H]3CCCC[N+]3C)C(=O)c4cccc5ccccc54)c1	7.4
297	Brc1cc(C[C@H]2CCCC[N+]2C)cc(c1C)C(=O)c3cccc4ccccc43	7.4
298	O=C(c1c-2[nH]ncsn2cc([nH]1)C[C@H]3CCCC[N+]3C)c4cccc5ccccc54	7.4
299	O=C(c1cc(C[C@H]2CCCC[N+]2C)c(s1)CCC)c3cccc4ccccc43	7.4
300	Clc1c(OC)cc(C[C@H]2CCCC[N+]2C)cc1C(=O)c3cccc4ccccc43	7.4
301	Fc1c(C(=O)c2cccc3ccccc32)cc(F)cc1C[C@H]4CCCC[N+]4C	7.4
302	O=C(c1cn(C[C@H]2CCCC[N+]2C)cc3-n1oncs3)c4cccc5ccccc54	7.4
303	Oc1ccc(cc1C[C@H]2CCCC[N+]2C)C(=O)c3cccc4ccccc43	7.4
304	Clc1cc(c(O)c(C[C@H]2CCCC[N+]2C)c1)C(=O)c3cccc4ccccc43	7.4
305	O=C(c1c2ccsc2cc(C[C@H]3CCCC[N+]3C)c1)c4cccc5ccccc54	7.4
306	O=C(c1cn(C[C@H]2CCCC[N+]2C)cc3-n1ocno3)c4cccc5ccccc54	7.4
307	Fc1c(OC)cc(C[C@H]2CCCC[N+]2C)cc1C(=O)c3cccc4ccccc43	7.4
308	Brc1cc(c(O)c(C[C@H]2CCCC[N+]2C)c1O)C(=O)c3cccc4ccccc43	7.4
309	O=C(c1c2cnccc2cc(C[C@H]3CCCC[N+]3C)c1)c4cccc5ccccc54	7.4
310	Clc1c(c2cccc(c2n1C[C@H]3CCCC[N+]3C)CC)C(=O)c4cccc5ccccc54	7.4
311	Oc1c(c2ccccc2n1C[C@H]3CCCC[N+]3C)C(=O)c4cccc5ccccc54	7.4
312	Brc1cc(cn1C[C@H]2CCCC[N+]2C)C(=O)c3cccc4ccccc43	7.4
313	Fc1ccc2c(c(cn2C[C@H]3CCCC[N+]3C)C(=O)c4cccc5ccccc54)c1	7.4
314	O=C(c1cc(C[C@H]2CCCC[N+]2C)cc3ccnn31)c4cccc5ccccc54	7.4
315	O=C(c1cc(cc(C[C@H]2CCCC[N+]2C)c1)CC)c3cccc4ccccc43	7.4

316	O=C(c1c(sc(C[C@H]2CCCC[N+]2C)c1)C(=O)N)c3cccc4ccccc43	7.4
317	O=C(c1cn(C[C@H]2CCCC[N+]2C)c3c1c(OC)nc(n3)N)c4cccc5ccccc54	7.4
318	O=C(c1c2ccncc2cc(C[C@H]3CCCC[N+]3C)c1)c4cccc5ccccc54	7.4
319	O=C1c2csnc2N(C[C@H]3CCCC[N+]3C)C=C1C(=O)c4cccc5ccccc54	7.4
320	O=C(c1c2ccccc2c([nH]1)C[C@H]3CCCC[N+]3C)c4cccc5ccccc54	7.4
321	Clc1c(c2cccc(Cl)c2n1C[C@H]3CCCC[N+]3C)C(=O)c4cccc5ccccc54	7.4
322	Brc1cc(cc(C[C@H]2CCCC[N+]2C)c1O)C(=O)c3cccc4ccccc43	7.4
323	O=C(c1c2c(nncn2)cc(C[C@H]3CCCC[N+]3C)c1)c4cccc5ccccc54	7.4
324	O=C(c1cn(C[C@H]2CCCC[N+]2C)c3cc(ccc13)C)c4cccc5ccccc54	7.4
325	O=C(c1c2CCCCc2c([nH]1)C[C@H]3CCCC[N+]3C)c4cccc5ccccc54	7.4
326	O=C(c1cc(cc(C[C@H]2CCCC[N+]2C)c1)C)c3cccc4ccccc43	7.4
327	O=C(c1cc(C[C@H]2CCCC[N+]2C)cc3c(cccc31)C)c4cccc5ccccc54	7.4
328	O=C(c1c2cnsc2c([nH]1)C[C@H]3CCCC[N+]3C)c4cccc5ccccc54	7.4
329	O=C(c1cn(C[C@H]2CCCC[N+]2C)c3c1ccs3)c4cccc5ccccc54	7.4
330	O=C(c1cn(C[C@H]2CCCC[N+]2C)c3ccnn31)c4cccc5ccccc54	7.4
331	Clc1cc(c(O)c(C[C@H]2CCCC[N+]2C)c1O)C(=O)c3cccc4ccccc43	7.4
332	O=C(c1cn(C[C@H]2CCCC[N+]2C)c3cc(c(cc13)C)C)c4cccc5ccccc54	7.4
333	Oc1c2ccccc2c(cc1C[C@H]3CCCC[N+]3C)C(=O)c4cccc5ccccc54	7.4
334	O=C(c1c2c(ncs2)cc(C[C@H]3CCCC[N+]3C)c1)c4cccc5ccccc54	7.4
335	Clc1cc(cc(C[C@H]2CCCC[N+]2C)c1)C(=O)c3cccc4ccccc43	7.4
336	Brc1c(Cl)c(cc(C[C@H]2CCCC[N+]2C)c1)C(=O)c3cccc4ccccc43	7.4
337	O=C(n1cc(C[C@H]2CCCC[N+]2C)c3c1C(=O)C=CS3)c4cccc5ccccc54	7.4
338	O=C(c1cc(N)c([nH]1)C[C@H]2CCCC[N+]2C)c3cccc4ccccc43	7.4
339	Oc1c(sc(c1C[C@H]2CCCC[N+]2C)C)C(=O)c3cccc4ccccc43	7.4
340	O=C(c1cn(C[C@H]2CCCC[N+]2C)c3c(O)cccc31)c4cccc5ccccc54	7.4
341	O=C(c1cn(C[C@H]2CCCC[N+]2C)c3cccc([N+]([O-])=O)c31)c4cccc5ccccc54	7.4
342	O=C(c1cn(n2-c(s1)cocn2)C[C@H]3CCCC[N+]3C)c4cccc5ccccc54	7.3
343	Oc1c(C(=O)c2cccc3ccccc32)ccc(O)c1C[C@H]4CCCC[N+]4C	7.3
344	O=C(c1cc(C[C@H]2CCCC[N+]2C)cc(C(C)(C)C)c1)c3cccc4ccccc43	7.3
345	Clc1cc(cc(C[C@H]2CCCC[N+]2C)c1CC)C(=O)c3cccc4ccccc43	7.3
346	Clc1c(c2cccc(c2n1C[C@H]3CCCC[N+]3C)C)C(=O)c4cccc5ccccc54	7.3
347	O=C(c1cn(C[C@H]2CCCC[N+]2C)c3ccc(C(C)C)cc13)c4cccc5ccccc54	7.3
348	O=C(c1cn(C[C@H]2CCCC[N+]2C)c3cc(O)ccc31)c4cccc5ccccc54	7.3
349	O=C(c1cn(C[C@H]2CCCC[N+]2C)c3ccc(OC(C)C)cc31)c4cccc5ccccc54	7.3
350	O=C(c1c(c(c([nH]1)C[C@H]2CCCC[N+]2C)C)CC)c3cccc4ccccc43	7.3
351	O=C(c1c2c(onn2)cc(C[C@H]3CCCC[N+]3C)c1)c4cccc5ccccc54	7.3
352	Clc1cc(C[C@H]2CCCC[N+]2C)cc(c1C)C(=O)c3cccc4ccccc43	7.3
353	O=C(c1c2c(scn2)c([nH]1)C[C@H]3CCCC[N+]3C)c4cccc5ccccc54	7.3
354	O=C(c1cn(C[C@H]2CCCC[N+]2C)c3c1c(O)nc(n3)N)c4cccc5ccccc54	7.3
355	Brc1cc(O)c(C[C@H]2CCCC[N+]2C)cc1C(=O)c3cccc4ccccc43	7.3
356	O=C(c1cn(C[C@H]2CCCC[N+]2C)c3c1csn3)c4cccc5ccccc54	7.3
357	O=C(c1cc(c(o1)C[C@H]2CCCC[N+]2C)CC#N)c3cccc4ccccc43	7.3

358	O=C(c1c2cc3c(OCCO3)cc2n(C[C@H]4CCCC[N+]4C)c1)c5cccc6ccccc65	7.3
359	O=C(c1cc(OC)cc(C[C@H]2CCCC[N+]2C)c1)c3cccc4ccccc43	7.3
360	Fc1c(F)cc(C[C@H]2CCCC[N+]2C)cc1C(=O)c3cccc4ccccc43	7.3
361	OCc1cc(cc(C[C@H]2CCCC[N+]2C)c1)C(=O)c3cccc4ccccc43	7.3
362	Fc1c(O)c(cc(C[C@H]2CCCC[N+]2C)c1)C(=O)c3cccc4ccccc43	7.3
363	O=C(c1cc(SC)cc(C[C@H]2CCCC[N+]2C)c1)c3cccc4ccccc43	7.3
364	O=C(c1cc2c(ocn2)c(C[C@H]3CCCC[N+]3C)c1)c4cccc5ccccc54	7.3
365	Brc1c(F)c(cc(C[C@H]2CCCC[N+]2C)c1)C(=O)c3cccc4ccccc43	7.3
366	O=C(c1cn(C[C@H]2CCCC[N+]2C)c3c1cn[nH]3)c4cccc5ccccc54	7.3
367	Clc1c(c2cccc(OC)c2n1C[C@H]3CCCC[N+]3C)C(=O)c4cccc5ccccc54	7.3
368	O=C(c1cc(NC)cc(C[C@H]2CCCC[N+]2C)c1)c3cccc4ccccc43	7.3
369	FC(F)(F)c1c(O)c(cc(C[C@H]2CCCC[N+]2C)c1)C(=O)c3cccc4ccccc43	7.3
370	Fc1c(cc(C[C@H]2CCCC[N+]2C)cc1C(F)(F)F)C(=O)c3cccc4ccccc43	7.3
371	O=C(c1c(OCC)ncc(C[C@H]2CCCC[N+]2C)c1)c3cccc4ccccc43	7.3
372	O=C(c1cn(C[C@H]2CCCC[N+]2C)c3ccncc31)c4cccc5ccccc54	7.3
373	O=C(c1cc(C[C@H]2CCCC[N+]2C)cc(C(C)C)c1)c3cccc4ccccc43	7.3
374	O=C(c1cn(C[C@H]2CCCC[N+]2C)c3c(OC)cc(cc13)C)c4cccc5ccccc54	7.3
375	O=C(c1cn(C[C@H]2CCCC[N+]2C)c3ccc(OCC)cc31)c4cccc5ccccc54	7.3
376	O=C(c1cc(cc(C[C@H]2CCCC[N+]2C)c1)COC)c3cccc4ccccc43	7.3
377	Clc1c(Cl)cc(C[C@H]2CCCC[N+]2C)cc1C(=O)c3cccc4ccccc43	7.3
378	O=C(c1cn(C[C@H]2CCCC[N+]2C)c3CC[N+]Cc13)c4cccc5ccccc54	7.3
379	O=C(c1cc2CCCc2c(C[C@H]3CCCC[N+]3C)c1)c4cccc5ccccc54	7.3
380	O=C(c1c(c(c([nH]1)C[C@H]2CCCC[N+]2C)C)C)c3cccc4ccccc43	7.3
381	Clc1ccc2c(n(C[C@H]3CCCC[N+]3C)cc2C(=O)c4cccc5ccccc54)c1	7.3
382	Fc1c(N)c(cc(C[C@H]2CCCC[N+]2C)c1)C(=O)c3cccc4ccccc43	7.3
383	O=C(c1cn(C[C@H]2CCCC[N+]2C)c3ccc([N+]([O-])=O)cc31)c4cccc5ccccc54	7.2
384	O=C(c1cn(C[C@H]2CCCC[N+]2C)c3ccc(cc31)C#N)c4cccc5ccccc54	7.2
385	O=C(c1c2C(SC=Cc2c([nH]1)C[C@H]3CCCC[N+]3C)=O)c4cccc5ccccc54	7.2
386	O=C(C1=CN(C[C@H]2CCCC[N+]2C)C=3C(=O)C=CC3S1)c4cccc5ccccc54	7.2
387	Fc1c(c(cc(C[C@H]2CCCC[N+]2C)c1)C(=O)c3cccc4ccccc43)C#N	7.2
388	Brc1c(cc(s1)C[C@H]2CCCC[N+]2C)C(=O)c3cccc4ccccc43	7.2
389	Fc1cc(c(O)c(C[C@H]2CCCC[N+]2C)c1)C(=O)c3cccc4ccccc43	7.2
390	O=C(c1cccc(C[C@H]2CCCC[N+]2C)c1)c3cccc4ccccc43	7.2
391	O=C(c1c(OC)ncc(C[C@H]2CCCC[N+]2C)c1)c3cccc4ccccc43	7.2
392	O=C(n1c([n+](C[C@H]2CCCC[N+]2C)c3ccccc31)C)c4cccc5ccccc54	7.2
393	O=C(c1cn(C[C@H]2CCCC[N+]2C)c3c1nccn3)c4cccc5ccccc54	7.2
394	O=C(c1c2c(nno2)cc(C[C@H]3CCCC[N+]3C)c1)c4cccc5ccccc54	7.2
395	O=C(c1cc(cc(C[C@H]2CCCC[N+]2C)c1N)C)c3cccc4ccccc43	7.2
396	Clc1c(cc(C[C@H]2CCCC[N+]2C)cc1C(F)(F)F)C(=O)c3cccc4ccccc43	7.2
397	O=C(c1cn(C[C@H]2CCCC[N+]2C)c3c1c(O)ncn3)c4cccc5ccccc54	7.2
398	Clc1ccc(c(OC[C@H]2CCCC[N+]2C)c1)CC(=O)c3cccc4ccccc43	7.2
399	O=C(c1cc(OCC)cc(C[C@H]2CCCC[N+]2C)c1)c3cccc4ccccc43	7.2

400	Clc1c(c2cccc(F)c2n1C[C@H]3CCCC[N+]3C)C(=O)c4cccc5ccccc54	7.2
401	O=C(c1cn(n2n1sncs2)C[C@H]3CCCC[N+]3C)c4cccc5ccccc54	7.2
402	Fc1ccc2c(n(C[C@H]3CCCC[N+]3C)cc2C(=O)c4cccc5ccccc54)c1	7.2
403	O=C(c1c(sc(C[C@H]2CCCC[N+]2C)c1)OC)c3cccc4ccccc43	7.2
404	O=C(c1cn(n2ncsn2s1)C[C@H]3CCCC[N+]3C)c4cccc5ccccc54	7.2
405	Clc1cccc2c1n(C[C@H]3CCCC[N+]3C)cc2C(=O)c4cccc5ccccc54	7.2
406	O=C(c1c2c(c([nH]1)C[C@H]3CCCC[N+]3C)ccs2)c4cccc5ccccc54	7.2
407	FC(F)(F)c1ccc2c(cn(C[C@H]3CCCC[N+]3C)c2c1)C(=O)c4cccc5ccccc54	7.2
408	Brc1cc(c(N)c(C[C@H]2CCCC[N+]2C)c1)C(=O)c3cccc4ccccc43	7.2
409	O=C(c1cn(C[C@H]2CCCC[N+]2C)c3c1c(OC)ncn3)c4cccc5ccccc54	7.2
410	Brc1cc(c(O)c(C[C@H]2CCCC[N+]2C)c1)C(=O)c3cccc4ccccc43	7.2
411	O=C(c1cn(C[C@H]2CCCC[N+]2C)c3cccn31)c4cccc5ccccc54	7.2
412	Fc1c(OCC)cc(C[C@H]2CCCC[N+]2C)cc1C(=O)c3cccc4ccccc43	7.2
413	Clc1c(F)cc(C[C@H]2CCCC[N+]2C)cc1C(=O)c3cccc4ccccc43	7.2
414	Brc1cc(C[C@H]2CCCC[N+]2C)cc(n1)C(=O)c3cccc4ccccc43	7.1
415	Fc1cccc2c1n(C[C@H]3CCCC[N+]3C)cc2C(=O)c4cccc5ccccc54	7.1
416	O=C(c1cn(C[C@H]2CCCC[N+]2C)c3c(ccc(c31)C)C)c4cccc5ccccc54	7.1
417	Clc1cc(c(N)c(C[C@H]2CCCC[N+]2C)c1)C(=O)c3cccc4ccccc43	7.1
418	O=C(c1cn(n2[nH]ccn2s1)C[C@H]3CCCC[N+]3C)c4cccc5ccccc54	7.1
419	O=C(n1cc(n2[nH]ncsn12)C[C@H]3CCCC[N+]3C)c4cccc5ccccc54	7.1
420	O=C(c1cn(C[C@H]2CCCC[N+]2C)c3c1cccc3C(C)C)c4cccc5ccccc54	7.1
421	O=C(c1cc[n+](C[C@H]2CCCC[N+]2C)c3ccccc13)c4cccc5ccccc54	7.1
422	O=C(c1cc(C[C@H]2CCCC[N+]2C)c(s1)C(C)C)c3cccc4ccccc43	7.1
423	O=C(c1cn(C[C@H]2CCCC[N+]2C)c3cc(ccc13)C(=O)N)c4cccc5ccccc54	7.1
424	O=C(c1cn(C[C@H]2CCCC[N+]2C)c3c(OCC)cccc13)c4cccc5ccccc54	7.1
425	O=C(c1cn(n2cnsn2s1)C[C@H]3CCCC[N+]3C)c4cccc5ccccc54	7.1
426	O=C(c1cn(C[C@H]2CCCC[N+]2C)c3c1cco3)c4cccc5ccccc54	7.1
427	FC(F)(F)c1cc(cc(C[C@H]2CCCC[N+]2C)c1)C(=O)c3cccc4ccccc43	7.1
428	O=C(c1c2c(nc(C[C@H]3CCCC[N+]3C)c1)cccn2)c4cccc5ccccc54	7.1
429	O=C(c1cn(C[C@H]2CCCC[N+]2C)c3cnsc31)c4cccc5ccccc54	7.1
430	O=C(c1cc(c([nH]1)C[C@H]2CCCC[N+]2C)C)c3cccc4ccccc43	7.1
431	O=C(c1cn(n2ccsn2s1)C[C@H]3CCCC[N+]3C)c4cccc5ccccc54	7.1
432	Fc1c(F)c(O)c(C[C@H]2CCCC[N+]2C)cc1C(=O)c3cccc4ccccc43	7.1
433	Clc1c2c(n(C[C@H]3CCCC[N+]3C)cc2C(=O)c4cccc5ccccc54)ncn1	7.1
434	O=C(c1cn(C[C@H]2CCCC[N+]2C)c3c1cc(cc3C)C)c4cccc5ccccc54	7.1
435	O=C(c1cn(C[C@H]2CCCC[N+]2C)c3c(cccc31)C)c4cccc5ccccc54	7.1
436	O=C(C=1C2=CSC(=O)N2C=C(C[C@H]3CCCC[N+]3C)C1)c4cccc5ccccc54	7.1
437	Fc1cc(C[C@H]2CCCC[N+]2C)cc(c1C)C(=O)c3cccc4ccccc43	7.1
438	O=C(c1c2C(=O)C=CSc2c([nH]1)C[C@H]3CCCC[N+]3C)c4cccc5ccccc54	7.1
439	O=S(=O)(c1cc(cc(C[C@H]2CCCC[N+]2C)c1)C(=O)c3cccc4ccccc43)C	7.1
440	O=C(c1cn(n2-c(s1)cscn2)C[C@H]3CCCC[N+]3C)c4cccc5ccccc54	7.1
441	Oc1c(OC)cc(cc1C[C@H]2CCCC[N+]2C)C(=O)c3cccc4ccccc43	7.1

442	O=C(c1cn(C[C@H]2CCCC[N+]2C)c3cc(OC)ccc31)c4cccc5ccccc54	7.1
443	Fc1cc(cc(C[C@H]2CCCC[N+]2C)c1)C(=O)c3cccc4ccccc43	7.1
444	O=C(c1c2ccnn2cc(C[C@H]3CCCC[N+]3C)c1)c4cccc5ccccc54	7.1
445	O=C(c1cn(C[C@H]2CCCC[N+]2C)c3c1cccc3CC)c4cccc5ccccc54	7
446	Sc1c2c(n(C[C@H]3CCCC[N+]3C)cc2C(=O)c4cccc5ccccc54)ncn1	7
447	O=C(c1cn(C[C@H]2CCCC[N+]2C)c3c1scn3)c4cccc5ccccc54	7
448	Clc1c(cc(s1)C[C@H]2CCCC[N+]2C)C(=O)c3cccc4ccccc43	7
449	O=C(c1cn(n2cncc12)C[C@H]3CCCC[N+]3C)c4cccc5ccccc54	7
450	O=C(c1c(N)c(OC)cc(C[C@H]2CCCC[N+]2C)c1)c3cccc4ccccc43	7
451	O=C(c1c(c(c(s1)C[C@H]2CCCC[N+]2C)C(=O)N)C)c3cccc4ccccc43	7
452	O=C(c1cn(C[C@H]2CCCC[N+]2C)c3c1SC(S3)=O)c4cccc5ccccc54	7
453	O=C(c1cn(C[C@H]2CCCC[N+]2C)c3cn[nH]c31)c4cccc5ccccc54	7
454	O=C(c1cc(C[C@H]2CCCC[N+]2C)c(s1)CCOC)c3cccc4ccccc43	7
455	O=C(c1cn(C[C@H]2CCCC[N+]2C)cc3-n1snco3)c4cccc5ccccc54	7
456	O=C(c1cn(C[C@H]2CCCC[N+]2C)c(SC)n1)c3cccc4ccccc43	7
457	O=C(c1cn(C[C@H]2CCCC[N+]2C)c3c1NC(S3)=O)c4cccc5ccccc54	7
458	Clc1c(N)c(cc(C[C@H]2CCCC[N+]2C)c1)C(=O)c3cccc4ccccc43	7
459	Oc1c(cc(C[C@H]2CCCC[N+]2C)cn1)C(=O)c3cccc4ccccc43	7
460	Brc1c(Cl)cc(C[C@H]2CCCC[N+]2C)cc1C(=O)c3cccc4ccccc43	7
461	O=C(c1cn(C[C@H]2CCCC[N+]2C)c3c1cccn3)c4cccc5ccccc54	6.9
462	Fc1cc(c(N)c(C[C@H]2CCCC[N+]2C)c1)C(=O)c3cccc4ccccc43	6.9
463	Clc1c(Cl)c([nH]c1C[C@H]2CCCC[N+]2C)C(=O)c3cccc4ccccc43	6.9
464	OCc1cc(cn1C[C@H]2CCCC[N+]2C)C(=O)c3cccc4ccccc43	6.9
465	O=C(c1cc(C[C@H]2CCCC[N+]2C)c(s1)CC)c3cccc4ccccc43	6.9
466	Clc1ccc2c(n(C[C@H]3CCCC[N+]3C)cc2C(=O)c4cccc5ccccc54)c1C	6.9
467	O=C(c1cn(C[C@H]2CCCC[N+]2C)c3c1cnc(n3)C)c4cccc5ccccc54	6.9
468	O=C(c1c2cc(OC)c(OC)cc2n(C[C@H]3CCCC[N+]3C)c1)c4cccc5ccccc54	6.9
469	O=C(c1c2c(nnnn2)cc(C[C@H]3CCCC[N+]3C)c1)c4cccc5ccccc54	6.9
470	O=C(c1c2ccc3ccccc3c2n(C[C@H]4CCCC[N+]4C)c1)c5cccc6ccccc65	6.9
471	Brc1c(N)c(cc(C[C@H]2CCCC[N+]2C)c1)C(=O)c3cccc4ccccc43	6.9
472	Brc1cc([nH]c1C(=O)c2cccc3ccccc32)C[C@H]4CCCC[N+]4C	6.9
473	O=C(c1c(cc[n+](C[C@H]2CCCC[N+]2C)c1)C)c3cccc4ccccc43	6.9
474	O=C(c1cn(C[C@H]2CCCC[N+]2C)c3c1cncn3)c4cccc5ccccc54	6.9
475	O=C(c1c(nc(s1)C[C@H]2CCCC[N+]2C)CC(C)C)c3cccc4ccccc43	6.9
476	O=C(c1cn(C[C@H]2CCCC[N+]2C)c3c1ccc(c3C)C)c4cccc5ccccc54	6.9
477	O=C(c1c2ccoc2c([nH]1)C[C@H]3CCCC[N+]3C)c4cccc5ccccc54	6.9
478	O=C(c1cn(C[C@H]2CCCC[N+]2C)c3c1ccc(OC)n3)c4cccc5ccccc54	6.9
479	O=C(c1cn(C[C@H]2CCCC[N+]2C)c3cncn31)c4cccc5ccccc54	6.8
480	O=C(c1c2ccncc2c([nH]1)C[C@H]3CCCC[N+]3C)c4cccc5ccccc54	6.8
481	O=C(c1cc(n(C[C@H]2CCCC[N+]2C)c1)C[N+]C)c3cccc4ccccc43	6.8
482	O=C(c1c2c([C@H]3CC[C@@H]2C3)c([nH]1)C[C@H]4CCCC[N+]4C)c5cccc6ccccc65	6.8
483	O=C(c1cn(C[C@H]2CCCC[N+]2C)c3cnccc31)c4cccc5ccccc54	6.8

484	O=C(c1cc(c[n+](C[C@H]2CCCC[N+]2C)c1)C)c3cccc4ccccc43	6.8
485	OCc1c(C[C@H]2CCCC[N+]2C)cc(s1)C(=O)c3cccc4ccccc43	6.8
486	O=C(c1cn(C[C@H]2CCCC[N+]2C)c3c1ccc(n3)C)c4cccc5ccccc54	6.8
487	O=C(c1c2c(nc(C[C@H]3CCCC[N+]3C)c1)cncn2)c4cccc5ccccc54	6.8
488	Brc1cc(c[n+](C[C@H]2CCCC[N+]2C)c1)C(=O)c3cccc4ccccc43	6.7
489	Fc1c(cc(C[C@H]2CCCC[N+]2C)cn1)C(=O)c3cccc4ccccc43	6.7
490	O=C(c1cc(nn2cccc12)C[C@H]3CCCC[N+]3C)c4cccc5ccccc54	6.7
491	O=C(c1cn(C[C@H]2CCCC[N+]2C)c3cnncc31)c4cccc5ccccc54	6.7
492	O=C(c1ccc[n+](C[C@H]2CCCC[N+]2C)c1)c3cccc4ccccc43	6.7
493	O=C(c1cn(C[C@H]2CCCC[N+]2C)cc3-n1sncs3)c4cccc5ccccc54	6.7
494	O=C(n1c[n+](C[C@H]2CCCC[N+]2C)c3ccccc31)c4cccc5ccccc54	6.7
495	O=C(c1cn(n2c1ccn2)C[C@H]3CCCC[N+]3C)c4cccc5ccccc54	6.6
496	Brc1c(cn(n1)C[C@H]2CCCC[N+]2C)C(=O)c3cccc4ccccc43	6.6
497	O=C(c1cn(C[C@H]2CCCC[N+]2C)c(C[N+])c1)c3cccc4ccccc43	6.6
498	O=C(c1cc(C[C@H]2CCCC[N+]2C)c(s1)C[N+])c3cccc4ccccc43	6.5
499	O=C(c1cn(C[C@H]2CCCC[N+]2C)c3nccn31)c4cccc5ccccc54	6.4
500	Brc1c(c([nH]c1C[C@H]2CCCC[N+]2C)C(=O)c3cccc4ccccc43)C#N	6.3

Table S13. List, SMILE and predicted pK_i values for Series 3 in CB_2 receptor.

N°	SMILES	Pred pK_i
1	O=C(c1c2ccccc2n(C[C@H]3CCCC[N+]3C)c1)c4cc5ccccc5c6ccccc64	8.3
2	O=C(c1cn(C[C@H]2CCCC[N+]2C)c3ccccc31)C4=C/C(Nc5ccccc54)=N/N	8.2
3	Clc1cccc(c1NC(=O)c2c3ccccc3n(C[C@H]4CCCC[N+]4C)c2)C(F)(F)F	8.2
4	O=S(=O)(c1c2ccccc2n(C[C@H]3CCCC[N+]3C)c1)c4cccc5ccc(nc54)C	8.1
5	O=C(N[C@@H]1CCCC[C@H]1SCC)c2c3ccccc3n(C[C@H]4CCCC[N+]4C)c2	8.1
6	O=C(c1cn(C[C@H]2CCCC[N+]2C)c3ccccc31)c4c5ccccc5cc6ccccc64	8.1
7	O=C(n1c2ccccc2c3CCCCc31)c4cn(C[C@H]5CCCC[N+]5C)c6ccccc64	8.1
8	O=S(=O)(c1c2ccccc2n(C[C@H]3CCCC[N+]3C)c1)c4cccc5cccnc54	8.1
9	Clc1ccc(nc1C(=O)c2c3ccccc3n(C[C@H]4CCCC[N+]4C)c2)NC	8.1
10	O=C1C=C(c2ccccc2N1C)C(=O)c3cn(C[C@H]4CCCC[N+]4C)c5ccccc53	8.1
11	O=C(c1c2ccccc2n(C[C@H]3CCCC[N+]3C)c1)c4cc(nc5ccc(cc45)C)C	8
12	O=S(=O)(c1cn(C[C@H]2CCCC[N+]2C)c3ccccc31)c4ccccc4S(=O)(=O)C	8
13	O=C(c1c2ccccc2n(C[C@H]3CCCC[N+]3C)c1)c4c5cccc(N)c5c(C)cn4	8
14	Brc1c(nn(c1C(=O)c2c3ccccc3n(C[C@H]4CCCC[N+]4C)c2)C)C	8
15	O=C(c1c2ccccc2n(C[C@H]3CCCC[N+]3C)c1)c4c5cc(N)ccc5ccn4	8
16	O=C(Oc1ccccc1C(C)(C)C)c2c3ccccc3n(C[C@H]4CCCC[N+]4C)c2	7.9
17	O=C(c1c2ccccc2n(C[C@H]3CCCC[N+]3C)c1)c4cc(cc(c4O)C)C	7.9
18	O=S(=O)(N)c1ccccc1C(=O)c2c3ccccc3n(C[C@H]4CCCC[N+]4C)c2	7.9
19	O=S(=O)(c1ccccc1C(=O)c2c3ccccc3n(C[C@H]4CCCC[N+]4C)c2)C	7.9
20	O=C(c1cn(C[C@H]2CCCC[N+]2C)c3ccccc31)c4ccc(c5ccccc54)C	7.9
21	O=C(c1cn(C[C@H]2CCCC[N+]2C)c3ccccc31)c4cnnc5ccccc54	7.9

22	Fc1ccc(S(=O)(=O)C)c(C(=O)c2c3ccccc3n(C[C@H]4CCCC[N+]4C)c2)c1	7.9
23	O=C(c1cn(C[C@H]2CCCC[N+]2C)c3ccccc31)c4c5ccccc5nc6CCCc46	7.9
24	O=C(c1cn(C[C@H]2CCCC[N+]2C)c3ccccc31)c4c5ccccc5c(O)nn4	7.9
25	O=C(c1cn(C[C@H]2CCCC[N+]2C)c3ccccc31)c4cc(nc5ccccc54)C	7.9
26	O=C(c1cn(C[C@H]2CCCC[N+]2C)c3ccccc31)c4ccccc4C(=O)C	7.9
27	O=C(c1c2ccccc2n(C[C@H]3CCCC[N+]3C)c1)c4c5cc(cc(c5nc(c4)C)C)C	7.9
28	O=C(c1cn(C[C@H]2CCCC[N+]2C)c3ccccc31)c4c(oc5ccccc54)CC	7.9
29	O=C(c1c2ccccc2n(C[C@H]3CCCC[N+]3C)c1)c4cc(N)ccc4C	7.9
30	O=C([C@@H]1c2ccsc2CCS1)c3c4ccccc4n(C[C@H]5CCCC[N+]5C)c3	7.8
31	Fc1ccc2c(nc(cc2C(=O)c3c4ccccc4n(C[C@H]5CCCC[N+]5C)c3)C)c1	7.8
32	O=C(c1c2ccccc2n(C[C@H]3CCCC[N+]3C)c1)c4cc(nc5c(C)cccc45)C	7.8
33	O=C(c1c2ccccc2n(C[C@H]3CCCC[N+]3C)c1)c4c5CCC(Cc5c(s4)C)(C)C	7.8
34	O=C(c1cn(C[C@H]2CCCC[N+]2C)c3ccccc31)c4c(O)ccc5ccccc54	7.8
35	Clc1cc(Cl)cc(C(=O)c2c3ccccc3n(C[C@H]4CCCC[N+]4C)c2)c1OC	7.8
36	O=C(N1c2ccc(N)cc2CCC1)c3c4ccccc4n(C[C@H]5CCCC[N+]5C)c3	7.8
37	O=C(c1c2ccccc2n(C[C@H]3CCCC[N+]3C)c1)c4ccc(OC)cc4C	7.8
38	O=C(N[C@@H]1C[C@@H](CC[C@H]1C(C)C)C)c2c3ccccc3n(C[C@H]4CCCC[N+]4C)c2	7.8
39	O=C(c1c2ccccc2n(C[C@H]3CCCC[N+]3C)c1)c4cc(ccc4SC)C	7.8
40	Clc1cc(c2ccccc2n1)C(=O)c3cn(C[C@H]4CCCC[N+]4C)c5ccccc53	7.8
41	Oc1cccc2c1N(CCC2)C(=O)c3c4ccccc4n(C[C@H]5CCCC[N+]5C)c3	7.8
42	Fc1ccc2c(N(CCN2C)C(=O)c3c4ccccc4n(C[C@H]5CCCC[N+]5C)c3)c1	7.8
43	O=C(c1cn(C[C@H]2CCCC[N+]2C)c3ccccc31)c4c(OC)ccc5ccccc54	7.7
44	O=C(c1cc(n(c1C)C)C)c2c3ccccc3n(C[C@H]4CCCC[N+]4C)c2	7.7
45	O=C([C@@H]1c2ccccc2CCC1)c3cn(C[C@H]4CCCC[N+]4C)c5ccccc53	7.7
46	O=C(c1c2ccccc2n(C[C@H]3CCCC[N+]3C)c1)c4cccc(OC)c4N	7.7
47	O=C(n1c(c(c2ccccc21)C)C)c3cn(C[C@H]4CCCC[N+]4C)c5ccccc53	7.7
48	O=C(c1c2c(nn1C)CCC2)c3c4ccccc4n(C[C@H]5CCCC[N+]5C)c3	7.7
49	O=S(=O)(c1c2ccccc2n(C[C@H]3CCCC[N+]3C)c1)c4ccccc4C(OC)=O	7.7
50	Fc1cc(C(=O)c2c3ccccc3n(C[C@H]4CCCC[N+]4C)c2)cc(c1)C	7.7
51	O=C(c1c(cccc1N)C)c2c3ccccc3n(C[C@H]4CCCC[N+]4C)c2	7.7
52	O=C(O[C@@H]1CCCC[C@H]1CC)c2cn(C[C@H]3CCCC[N+]3C)c4ccccc42	7.7
53	O=C(c1cn(C[C@H]2CCCC[N+]2C)c3ccccc31)c4ccccc4NC	7.7
54	O=C(c1cn(C[C@H]2CCCC[N+]2C)c3ccccc31)c4cc(OC)nc5ccccc54	7.7
55	O=C(c1cn(C[C@H]2CCCC[N+]2C)c3ccccc31)c4cc(nc5ccccc54)N	7.7
56	Clc1ccc(c2ccccc12)C(=O)c3cn(C[C@H]4CCCC[N+]4C)c5ccccc53	7.7
57	Brc1ccc([N+]([O-])=O)cc1C(=O)c2c3ccccc3n(C[C@H]4CCCC[N+]4C)c2	7.7
58	O=C(N1c2ccccc2C[C@@H](C1)C)c3cn(C[C@H]4CCCC[N+]4C)c5ccccc53	7.7
59	O=C(c1c2ccccc2n(C[C@H]3CCCC[N+]3C)c1)c4cccc(c4N)C	7.6
60	O=C(c1c2ccccc2n(C[C@H]3CCCC[N+]3C)c1)c4ccc5CCc6cccc4c65	7.6
61	Clc1ccc(NC)c(C(=O)c2c3ccccc3n(C[C@H]4CCCC[N+]4C)c2)c1	7.6
62	Clc1ccc(NCC)c(C(=O)c2c3ccccc3n(C[C@H]4CCCC[N+]4C)c2)c1	7.6
63	O=C(c1cn(C[C@H]2CCCC[N+]2C)c3ccccc31)c4c5ccccc5ccc4C	7.6

64	Clc1ccc(Cl)nc1C(=O)c2c3ccccc3n(C[C@H]4CCCC[N+]4C)c2	7.6
65	O=C(c1cn(C[C@H]2CCCC[N+]2C)c3ccccc31)c4cc(CC)ccc4CC	7.6
66	O=C(c1c2ccccc2n(C[C@H]3CCCC[N+]3C)c1)c4c5ccc(N)cc5ccn4	7.6
67	O=C(c1ccc(cc1C)C)c2c3ccccc3n(C[C@H]4CCCC[N+]4C)c2	7.6
68	Fc1cc(F)cnc1C(=O)c2c3ccccc3n(C[C@H]4CCCC[N+]4C)c2	7.6
69	O=C(N[C@@H]1CCCC[C@H]1C(C)C)c2c3ccccc3n(C[C@H]4CCCC[N+]4C)c2	7.6
70	FC(F)Oc1ccccc1C(=O)c2c3ccccc3n(C[C@H]4CCCC[N+]4C)c2	7.6
71	Clc1ccc(N)cc1S(=O)(=O)c2c3ccccc3n(C[C@H]4CCCC[N+]4C)c2	7.6
72	Clc1cc(Cl)c(Cl)c(C(=O)c2c3ccccc3n(C[C@H]4CCCC[N+]4C)c2)c1O	7.6
73	O=C(c1c2ccccc2n(C[C@H]3CCCC[N+]3C)c1)c4cccc(c4)C	7.6
74	O=C(Oc1ccccc1SC)c2c3ccccc3n(C[C@H]4CCCC[N+]4C)c2	7.6
75	O=C(c1c2ccccc2n(C[C@H]3CCCC[N+]3C)c1)c4cccc5CCOc45	7.6
76	O=C(c1cccc2c1CCCN2)c3c4ccccc4n(C[C@H]5CCCC[N+]5C)c3	7.6
77	Oc1c(C(=O)c2c3ccccc3n(C[C@H]4CCCC[N+]4C)c2)c(cc(n1)C)C	7.6
78	O=C(c1c2ccccc2n(C[C@H]3CCCC[N+]3C)c1)c4cccc(c4)C#C	7.6
79	Oc1cccc(C(=O)c2c3ccccc3n(C[C@H]4CCCC[N+]4C)c2)c1O	7.6
80	O=S(=O)(c1cn(C[C@H]2CCCC[N+]2C)c3ccccc31)c4cn(c5ccccc54)C	7.5
81	Clc1c(ccc(F)c1C(=O)c2c3ccccc3n(C[C@H]4CCCC[N+]4C)c2)C	7.5
82	Fc1cccc2c1N(CCC2)C(=O)c3c4ccccc4n(C[C@H]5CCCC[N+]5C)c3	7.5
83	O=C(c1c2ccccc2n(C[C@H]3CCCC[N+]3C)c1)c4cccc(C(C)(C)C)c4	7.5
84	Clc1cccc(N(C(=O)c2c3ccccc3n(C[C@H]4CCCC[N+]4C)c2)CC)c1	7.5
85	Fc1cc(C)ccc1C(=O)c2c3ccccc3n(C[C@H]4CCCC[N+]4C)c2	7.5
86	Brc1ccc(F)c(C(=O)c2c3ccccc3n(C[C@H]4CCCC[N+]4C)c2)c1	7.5
87	O=C(c1cn(C[C@H]2CCCC[N+]2C)c3ccccc31)c4cncc5ccccc54	7.5
88	O=C([C@H]1c2ccccc2OCC1)c3c4ccccc4n(C[C@H]5CCCC[N+]5C)c3	7.5
89	O=S(=O)(c1c2ccccc2n(C[C@H]3CCCC[N+]3C)c1)c4cccc5cc(cnc54)C	7.5
90	O=C(N1[C@@H](CCC[C@H](C1)C)C)c2c3ccccc3n(C[C@H]4CCCC[N+]4C)c2	7.5
91	O=C(c1cn(C[C@H]2CCCC[N+]2C)c3ccccc31)c4c5ccccc5ccn4	7.5
92	Clc1cc(N)c2ccnc(c2c1)C(=O)c3c4ccccc4n(C[C@H]5CCCC[N+]5C)c3	7.5
93	O=C(c1c2ccccc2n(C[C@H]3CCCC[N+]3C)c1)c4cc(C)ccn4	7.5
94	O=C(c1c2ccccc2n(C[C@H]3CCCC[N+]3C)c1)c4ccccc4C(C)C	7.5
95	O=C(c1c2ccccc2n(C[C@H]3CCCC[N+]3C)c1)c4ccc(c(N)c4)C	7.5
96	Fc1ccc(N)c(C(=O)c2c3ccccc3n(C[C@H]4CCCC[N+]4C)c2)c1	7.5
97	O=C(c1c2ccccc2n(C[C@H]3CCCC[N+]3C)c1)c4cccc(c4O)C	7.5
98	Fc1ccc2c(c(C(=O)c3c4ccccc4n(C[C@H]5CCCC[N+]5C)c3)cc(n2)C)c1	7.5
99	O=C(c1cn(C[C@H]2CCCC[N+]2C)c3ccccc31)c4ccccc4-c5cnccc5	7.5
100	Brc1cc(O)c(O)cc1C(=O)c2cn(C[C@H]3CCCC[N+]3C)c4ccccc42	7.5
101	Brc1cc(F)ccc1C(=O)c2c3ccccc3n(C[C@H]4CCCC[N+]4C)c2	7.5
102	O=C(c1c2ccccc2n(C[C@H]3CCCC[N+]3C)c1)c4cccc(c4)C(C)(C)C#N	7.5
103	Clc1ccc(N)cc1C(=O)c2c3ccccc3n(C[C@H]4CCCC[N+]4C)c2	7.5
104	O=S(=O)(c1cn(C[C@H]2CCCC[N+]2C)c3ccccc31)c4csc5ccccc54	7.5
105	O=C(c1cn(C[C@H]2CCCC[N+]2C)c3ccccc31)c4cccc5Cc6ccccc6-c45	7.5

106	O=C(c1c2ccccc2n(C[C@H]3CCCC[N+]3C)c1)c4c5c(N)cccc5ccn4	7.5
107	O=C(c1c2ccccc2n(C[C@H]3CCCC[N+]3C)c1)c4ccccc4SCC	7.5
108	O=S(=O)(N1CCC[C@H]2CCCC[C@@H]21)c3c4ccccc4n(C[C@H]5CCCC[N+]5C)c3	7.5
109	O=C(c1c2ccccc2n(C[C@H]3CCCC[N+]3C)c1)c4cccc5ccoc54	7.5
110	Clc1cc(c(OC)c(c1)C)C(=O)c2c3ccccc3n(C[C@H]4CCCC[N+]4C)c2	7.5
111	O=C(c1c2ccccc2n(C[C@H]3CCCC[N+]3C)c1)c4c5ccc(c(N)c5ccn4)C	7.5
112	O=C(c1c2ccccc2n(C[C@H]3CCCC[N+]3C)c1)c4ccccc4OC	7.5
113	Brc1cc(F)c(N)cc1C(=O)c2c3ccccc3n(C[C@H]4CCCC[N+]4C)c2	7.5
114	Clc1ccc(Cl)c(C(=O)c2c3ccccc3n(C[C@H]4CCCC[N+]4C)c2)c1Cl	7.4
115	Clc1cc(c(cc1C(=O)c2cn(C[C@H]3CCCC[N+]3C)c4ccccc42)C)C	7.4
116	O=C(N1c2ccc[nH+]c2N(CC1)CC)c3c4ccccc4n(C[C@H]5CCCC[N+]5C)c3	7.4
117	O=C(N1c2ccccc2S[C@H](C1)C)c3cn(C[C@H]4CCCC[N+]4C)c5ccccc53	7.4
118	Clc1ccc(C(=O)c2c3ccccc3n(C[C@H]4CCCC[N+]4C)c2)c(C)c1	7.4
119	C[N+]1CCCC[C@@H]1Cn2cc(Nc3cccc4cccnc43)c5ccccc52	7.4
120	Clc1c(cccc1C(=O)c2c3ccccc3n(C[C@H]4CCCC[N+]4C)c2)C	7.4
121	O=S(=O)(N1CCC[C@H]1C(=O)c2c3ccccc3n(C[C@H]4CCCC[N+]4C)c2)C	7.4
122	Clc1cccc(N)c1C(=O)c2c3ccccc3n(C[C@H]4CCCC[N+]4C)c2	7.4
123	Clc1cc(C)ccc1C(=O)c2c3ccccc3n(C[C@H]4CCCC[N+]4C)c2	7.4
124	O=C(c1cn(C[C@H]2CCCC[N+]2C)c3ccccc31)c4cccc(OC)c4OC	7.4
125	O=C(c1cn(C[C@H]2CCCC[N+]2C)c3ccccc31)c4ccccc4N	7.4
126	O=C(c1cn(C[C@H]2CCCC[N+]2C)c3ccccc31)c4cc(c(cc4OC)C)C	7.4
127	O=C(c1c2ccccc2n(C[C@H]3CCCC[N+]3C)c1)c4cccc(OC)c4	7.4
128	O=C(c1c2ccccc2n(C[C@H]3CCCC[N+]3C)c1)c4cccc(N)c4C	7.4
129	Brc1ccc(N)cc1C(=O)c2c3ccccc3n(C[C@H]4CCCC[N+]4C)c2	7.4
130	O=C(Nc1ccccc1C(C)C)c2cn(C[C@H]3CCCC[N+]3C)c4ccccc42	7.4
131	O=S(=O)(c1cn(C[C@H]2CCCC[N+]2C)c3ccccc31)c4cccc5ccccc54	7.4
132	O=C(N1c2ccccc2CC[C@H]1C)c3cn(C[C@H]4CCCC[N+]4C)c5ccccc53	7.4
133	O=C(c1c2ccccc2n(C[C@H]3CCCC[N+]3C)c1)c4cccc(SCC#N)c4	7.4
134	O=C(N1c2ccccc2[C@@H](CC1)C)c3cn(C[C@H]4CCCC[N+]4C)c5ccccc53	7.4
135	O=[S@@](c1ccccc1C(=O)c2c3ccccc3n(C[C@H]4CCCC[N+]4C)c2)CC	7.4
136	Fc1ccc(N)c2c1ccnc2C(=O)c3c4ccccc4n(C[C@H]5CCCC[N+]5C)c3	7.4
137	Clc1cccc2c1cccc2S(=O)(=O)c3c4ccccc4n(C[C@H]5CCCC[N+]5C)c3	7.4
138	O=C(c1c2ccccc2n(C[C@H]3CCCC[N+]3C)c1)c4ccccc4CC	7.4
139	O=C(N1c2ccccc2N(CC1)C)c3cn(C[C@H]4CCCC[N+]4C)c5ccccc53	7.4
140	O=C(c1c2ccccc2n(C[C@H]3CCCC[N+]3C)c1)c4c(oc(c4)C)C	7.4
141	Clc1ccc(nc1C(=O)c2c3ccccc3n(C[C@H]4CCCC[N+]4C)c2)N	7.4
142	O=C(c1c2ccccc2n(C[C@H]3CCCC[N+]3C)c1)c4ccccc4SCC#N	7.4
143	Fc1cccc(F)c1NC(=O)c2c3ccccc3n(C[C@H]4CCCC[N+]4C)c2	7.4
144	O=C(c1c2ccccc2n(C[C@H]3CCCC[N+]3C)c1)c4cccc(N)c4	7.4
145	Oc1cccc(C(=O)c2c3ccccc3n(C[C@H]4CCCC[N+]4C)c2)c1	7.4
146	O=C(c1c2ccccc2n(C[C@H]3CCCC[N+]3C)c1)c4c(C(C)C)ccs4	7.4
147	O=C(c1c2ccccc2n(C[C@H]3CCCC[N+]3C)c1)c4cccc(c4C)C	7.4

148	O=C(c1cn(C[C@H]2CCCC[N+]2C)c3ccccc31)c4c(sc(n4)C)-c5ccccc5	7.4
149	O=C(N1c2cc(ccc2O[C@@H](C1)C)C)c3c4ccccc4n(C[C@H]5CCCC[N+]5C)c3	7.4
150	Clc1ccc(cc1S(=O)(=O)c2c3ccccc3n(C[C@H]4CCCC[N+]4C)c2)C	7.4
151	O=C(c1cn(C[C@H]2CCCC[N+]2C)c3ccccc31)[C@H]4c5ccccc5CCS4	7.4
152	Clc1c(c(Cl)ccc1S(=O)(=O)c2c3ccccc3n(C[C@H]4CCCC[N+]4C)c2)C	7.4
153	Brc1cccc(C(=O)c2c3ccccc3n(C[C@H]4CCCC[N+]4C)c2)c1N	7.4
154	O=C(N1c2ccccc2C[C@H]1C)c3cn(C[C@H]4CCCC[N+]4C)c5ccccc53	7.4
155	O=C(N1c2ccccc2N(CC1)CC)c3cn(C[C@H]4CCCC[N+]4C)c5ccccc53	7.4
156	O=C(c1c2ccccc2n(C[C@H]3CCCC[N+]3C)c1)c4cc(ccc4OC)C	7.4
157	Clc1cc(C(=O)c2c3ccccc3n(C[C@H]4CCCC[N+]4C)c2)ccc1OC	7.4
158	O=C(c1ccc(c(O)c1C)C)c2c3ccccc3n(C[C@H]4CCCC[N+]4C)c2	7.4
159	O=C(C1=CCCCCC1)c2c3ccccc3n(C[C@H]4CCCC[N+]4C)c2	7.4
160	Clc1ccc(F)c(C(=O)c2c3ccccc3n(C[C@H]4CCCC[N+]4C)c2)c1	7.4
161	O=C(c1c2ccccc2n(C[C@H]3CCCC[N+]3C)c1)c4c5cccc(N)c5cc(n4)C	7.4
162	Clc1cccc(O)c1C(=O)c2c3ccccc3n(C[C@H]4CCCC[N+]4C)c2	7.4
163	O=C([C@H]1CCCC[C@@H]1CO)c2c3ccccc3n(C[C@H]4CCCC[N+]4C)c2	7.4
164	Fc1cccc(C(=O)c2cn(C[C@H]3CCCC[N+]3C)c4ccccc42)c1OCC	7.4
165	Fc1ccc(cc1C(=O)c2c3ccccc3n(C[C@H]4CCCC[N+]4C)c2)C(F)(F)F	7.4
166	Brc1cncc(C(=O)c2c3ccccc3n(C[C@H]4CCCC[N+]4C)c2)c1	7.4
167	O=C(c1c2ccccc2n(C[C@H]3CCCC[N+]3C)c1)c4ccncc4C	7.4
168	O=C(c1cn(C[C@H]2CCCC[N+]2C)c3ccccc31)c4ccnc5ccccc54	7.4
169	Clc1cc(C(=O)c2c3ccccc3n(C[C@H]4CCCC[N+]4C)c2)cc(NC)n1	7.4
170	O=C(N1CCC[C@H]2CCC[C@H]21)c3c4ccccc4n(C[C@H]5CCCC[N+]5C)c3	7.4
171	Fc1ccc(cc1C(=O)c2c3ccccc3n(C[C@H]4CCCC[N+]4C)c2)C	7.3
172	Fc1ccc(F)c(C(=O)c2c3ccccc3n(C[C@H]4CCCC[N+]4C)c2)c1	7.3
173	S=C(N)c1ccccc1NC(=O)c2cn(C[C@H]3CCCC[N+]3C)c4ccccc42	7.3
174	O=C(N1CCS[C@H]1CCC)c2c3ccccc3n(C[C@H]4CCCC[N+]4C)c2	7.3
175	O=C(c1c2ccccc2n(C[C@H]3CCCC[N+]3C)c1)c4ccccc4	7.3
176	Fc1ccc(N)cc1C(=O)c2c3ccccc3n(C[C@H]4CCCC[N+]4C)c2	7.3
177	Clc1cccc(Cl)c1C(=O)c2c3ccccc3n(C[C@H]4CCCC[N+]4C)c2	7.3
178	O=C(C1CCSCC1)c2c3ccccc3n(C[C@H]4CCCC[N+]4C)c2	7.3
179	Brc1ccc(OC)c(C(=O)c2c3ccccc3n(C[C@H]4CCCC[N+]4C)c2)c1	7.3
180	Fc1cccc(C2(CC2)C(=O)c3c4ccccc4n(C[C@H]5CCCC[N+]5C)c3)c1	7.3
181	O=C(n1c(nc2ccccc21)C)c3cn(C[C@H]4CCCC[N+]4C)c5ccccc53	7.3
182	O=C(c1c2ccccc2n(C[C@H]3CCCC[N+]3C)c1)c4cc(ccc4C(C)C)C	7.3
183	O=C(N1c2ccc[nH+]c2N(CC1)C)c3c4ccccc4n(C[C@H]5CCCC[N+]5C)c3	7.3
184	O=C(N[C@@H]1CCCC[C@H]1CC)c2cn(C[C@H]3CCCC[N+]3C)c4ccccc42	7.3
185	O=S(=O)(c1c2ccccc2n(C[C@H]3CCCC[N+]3C)c1)c4cccc(c4C(OC)=O)C	7.3
186	Oc1cc(O)cc(C(=O)c2c3ccccc3n(C[C@H]4CCCC[N+]4C)c2)c1	7.3
187	O=C(c1c2ccccc2n(C[C@H]3CCCC[N+]3C)c1)c4csc(c4CC)C	7.3
188	O=C(c1c2ccccc2n(C[C@H]3CCCC[N+]3C)c1)c4ccnc(NCC)c4	7.3
189	O=S1(=O)c2ccccc2N(CC1)C(=O)c3cn(C[C@H]4CCCC[N+]4C)c5ccccc53	7.3

190	Brc1cc(c(c(c1C)C)C)C(=O)c2c3ccccc3n(C[C@H]4CCCC[N+]4C)c2	7.3
191	O=C(c1c2ccccc2n(C[C@H]3CCCC[N+]3C)c1)/C(=N/OC)c4ccccc4	7.3
192	C[N+]1CCCC[C@@H]1Cn2cc(c3ccccc32)C(c4cccc5ccccc54)=C	7.3
193	Brc1cccc(F)c1C(=O)c2c3ccccc3n(C[C@H]4CCCC[N+]4C)c2	7.3
194	Clc1cc(Cl)cc(Cl)c1C(=O)c2c3ccccc3n(C[C@H]4CCCC[N+]4C)c2	7.3
195	O=C(C1=C(c2ccccc2C1)C)c3c4ccccc4n(C[C@H]5CCCC[N+]5C)c3	7.3
196	Clc1cccc([N+]([O-])=O)c1C(=O)c2c3ccccc3n(C[C@H]4CCCC[N+]4C)c2	7.3
197	Clc1ccc(cc1C(=O)c2c3ccccc3n(C[C@H]4CCCC[N+]4C)c2)C	7.3
198	O=C(c1c2ccccc2n(C[C@H]3CCCC[N+]3C)c1)c4cccc(O)c4C	7.3
199	O=C(c1c2ccccc2n(C[C@H]3CCCC[N+]3C)c1)c4c5cccc(N)c5ccn4	7.3
200	O=C(N1c2ccc(OC)cc2CCC1)c3c4ccccc4n(C[C@H]5CCCC[N+]5C)c3	7.3
201	Clc1ccc(SCC)c(C(=O)c2c3ccccc3n(C[C@H]4CCCC[N+]4C)c2)c1	7.3
202	O[C@@H](c1ccccc1NC(=O)c2cn(C[C@H]3CCCC[N+]3C)c4ccccc42)C	7.3
203	O=C(c1c2ccccc2n(C[C@H]3CCCC[N+]3C)c1)c4c(C)csc4	7.3
204	FC(F)Oc1c(sc(c1)C)C(=O)c2c3ccccc3n(C[C@H]4CCCC[N+]4C)c2	7.3
205	Clc1cccc(Cl)c1NC(=O)c2c3ccccc3n(C[C@H]4CCCC[N+]4C)c2	7.3
206	Fc1ccccc1/C=C(/C(=O)c2c3ccccc3n(C[C@H]4CCCC[N+]4C)c2)C	7.3
207	Clc1cccc(C(=O)c2c3ccccc3n(C[C@H]4CCCC[N+]4C)c2)c1N	7.3
208	O=C(c1c2ccccc2n(C[C@H]3CCCC[N+]3C)c1)C4=C(OCCC4)C	7.3
209	O=C(c1c2ccccc2n(C[C@H]3CCCC[N+]3C)c1)c4cccc(NC(C)C)c4	7.3
210	Fc1c(cccc1C(=O)c2c3ccccc3n(C[C@H]4CCCC[N+]4C)c2)C	7.3
211	Brc1ccc(C(=O)c2c3ccccc3n(C[C@H]4CCCC[N+]4C)c2)c(C)c1	7.3
212	O=C(c1cn(C[C@H]2CCCC[N+]2C)c3ccccc31)c4cc(O)nc5ccccc54	7.3
213	Oc1ccc(C(C)C)cc1C(=O)c2c3ccccc3n(C[C@H]4CCCC[N+]4C)c2	7.3
214	Clc1cccc(S(=O)(=O)c2c3ccccc3n(C[C@H]4CCCC[N+]4C)c2)c1F	7.2
215	Brc1ccc(cc1C(=O)c2c3ccccc3n(C[C@H]4CCCC[N+]4C)c2)C	7.2
216	Fc1cccc(C(=O)c2cn(C[C@H]3CCCC[N+]3C)c4ccccc42)c1	7.2
217	Clc1c(cc(cc1C(=O)c2c3ccccc3n(C[C@H]4CCCC[N+]4C)c2)C)C	7.2
218	FC(Sc1ccccc1C(=O)c2c3ccccc3n(C[C@H]4CCCC[N+]4C)c2)F	7.2
219	Brc1ccc(c(C(=O)c2c3ccccc3n(C[C@H]4CCCC[N+]4C)c2)c1)C	7.2
220	O=C(N1c2cc(cc(c2CCC1)C)C)c3c4ccccc4n(C[C@H]5CCCC[N+]5C)c3	7.2
221	Brc1ccccc1S(=O)(=O)c2c3ccccc3n(C[C@H]4CCCC[N+]4C)c2	7.2
222	FC(F)Oc1cccc(C(=O)c2c3ccccc3n(C[C@H]4CCCC[N+]4C)c2)c1	7.2
223	Fc1cc(F)cc2c1N(CCC2)C(=O)c3c4ccccc4n(C[C@H]5CCCC[N+]5C)c3	7.2
224	Clc1cnccc1C(=O)c2c3ccccc3n(C[C@H]4CCCC[N+]4C)c2	7.2
225	O=C(c1cn(C[C@H]2CCCC[N+]2C)c3ccccc31)c4ccccc4-n5cccc5	7.2
226	Fc1ccc(c2ccccc12)C(=O)c3cn(C[C@H]4CCCC[N+]4C)c5ccccc53	7.2
227	Clc1cccc2c1cccc2C(=O)c3c4ccccc4n(C[C@H]5CCCC[N+]5C)c3	7.2
228	FC(F)(F)c1ccccc1S(=O)(=O)c2c3ccccc3n(C[C@H]4CCCC[N+]4C)c2	7.2
229	Fc1ccccc1S(=O)(=O)c2c3ccccc3n(C[C@H]4CCCC[N+]4C)c2	7.2
230	O=[S@](c1c2ccccc2n(C[C@H]3CCCC[N+]3C)c1)c4cccc(N)c4C#N	7.2
231	O=C(C1CCCCCCC1)c2cn(C[C@H]3CCCC[N+]3C)c4ccccc42	7.2

232	O=C(c1c2ccccc2n(C[C@H]3CCCC[N+]3C)c1)c4csc(c4)CC	7.2
233	O=C(C1(CCCC1)c2cccs2)c3c4ccccc4n(C[C@H]5CCCC[N+]5C)c3	7.2
234	Clc1cccc(C(=O)c2c3ccccc3n(C[C@H]4CCCC[N+]4C)c2)c1C	7.2
235	Clc1ccc2c(nccc2c1N)C(=O)c3c4ccccc4n(C[C@H]5CCCC[N+]5C)c3	7.2
236	O=C(c1c2ccccc2n(C[C@H]3CCCC[N+]3C)c1)c4cccc(SCC#C)c4	7.2
237	O[C@H](c1cn(C[C@H]2CCCC[N+]2C)c3ccccc31)c4cccc5ccccc54	7.2
238	O=C(c1c2ccccc2n(C[C@H]3CCCC[N+]3C)c1)C(=O)c4c[nH]c5ccccc54	7.2
239	O=C(c1c2ccccc2n(C[C@H]3CCCC[N+]3C)c1)c4cc(c(OC)c(c4C)C)C	7.2
240	O=C(c1c2ccccc2n(C[C@H]3CCCC[N+]3C)c1)c4cccc5C(=O)c6ccccc6-c54	7.2
241	O=C(c1c([nH]c2ccc(cc21)C)C)c3c4ccccc4n(C[C@H]5CCCC[N+]5C)c3	7.2
242	FC(F)Oc1ccsc1C(=O)c2c3ccccc3n(C[C@H]4CCCC[N+]4C)c2	7.2
243	Fc1c(F)cc(F)c(C(=O)c2c3ccccc3n(C[C@H]4CCCC[N+]4C)c2)c1	7.2
244	Fc1cc(N)c2ccnc(C(=O)c3c4ccccc4n(C[C@H]5CCCC[N+]5C)c3)c2c1	7.2
245	O=C(c1c2ccccc2n(C[C@H]3CCCC[N+]3C)c1)c4cc(SC)ccc4C	7.2
246	Fc1cc(C(=O)c2c3ccccc3n(C[C@H]4CCCC[N+]4C)c2)cc(C(F)(F)F)c1	7.2
247	O=C(c1c(ccc(C(C)(C)C)c1)C)c2c3ccccc3n(C[C@H]4CCCC[N+]4C)c2	7.1
248	O=C(c1c2ccccc2n(C[C@H]3CCCC[N+]3C)c1)c4cccnc4OCC	7.1
249	O=C(c1cn(C[C@H]2CCCC[N+]2C)c3ccccc31)c4cc(cc5ccccc54)C#N	7.1
250	O=C(c1cn(C[C@H]2CCCC[N+]2C)c3ccccc31)c4c(snn4)-c5ccccc5	7.1
251	O=C(c1c2ccccc2n(C[C@H]3CCCC[N+]3C)c1)c4ccccc4CO	7.1
252	O=C(N1c2cc(ccc2OCC1)C)c3c4ccccc4n(C[C@H]5CCCC[N+]5C)c3	7.1
253	Clc1cccc(C(=O)c2c3ccccc3n(C[C@H]4CCCC[N+]4C)c2)c1	7.1
254	O=C(C(C1CC1)C2CC2)c3c4ccccc4n(C[C@H]5CCCC[N+]5C)c3	7.1
255	O=C(c1c2ccccc2n(C[C@H]3CCCC[N+]3C)c1)c4cccc(c4)CC#N	7.1
256	Oc1ccc(O)c(C(=O)c2c3ccccc3n(C[C@H]4CCCC[N+]4C)c2)c1	7.1
257	Clc1cccc(C(=N)c2c3ccccc3n(C[C@H]4CCCC[N+]4C)c2)c1	7.1
258	O=C(c1c2ccccc2n(C[C@H]3CCCC[N+]3C)c1)c4csc(c4C)C	7.1
259	O=C(c1c2ccccc2n(C[C@H]3CCCC[N+]3C)c1)c4c5cc(cc(N)c5ccn4)C	7.1
260	Clc1cc(F)cc(C(=O)c2c3ccccc3n(C[C@H]4CCCC[N+]4C)c2)c1O	7.1
261	Clc1ccccc1C(=O)c2c3ccccc3n(C[C@H]4CCCC[N+]4C)c2	7.1
262	Clc1cccc(F)c1NC(=O)c2c3ccccc3n(C[C@H]4CCCC[N+]4C)c2	7.1
263	O=C(c1c2ccccc2n(C[C@H]3CCCC[N+]3C)c1)[C@@H]4COc5ccccc5O4	7.1
264	Clc1cccc(S(=O)(=O)c2c3ccccc3n(C[C@H]4CCCC[N+]4C)c2)c1C	7.1
265	Sc1ccccc1C(=O)c2c3ccccc3n(C[C@H]4CCCC[N+]4C)c2	7.1
266	Clc1cccc(C(=O)c2c3ccccc3n(C[C@H]4CCCC[N+]4C)c2)c1F	7.1
267	O=C(c1c2ccccc2n(C[C@H]3CCCC[N+]3C)c1)c4cccc5c4cccn5	7.1
268	O=C(c1c2ccccc2n(C[C@H]3CCCC[N+]3C)c1)c4cc(C)cs4	7.1
269	S=C(N)Cc1ccccc1C(=O)c2c3ccccc3n(C[C@H]4CCCC[N+]4C)c2	7.1
270	O=S(=O)(c1c2ccccc2n(C[C@H]3CCCC[N+]3C)c1)c4c(C)ccc(c4)C	7.1
271	Brc1cccc([C@H](O)c2c3ccccc3n(C[C@H]4CCCC[N+]4C)c2)c1	7.1
272	Fc1ccc(cc1S(=O)(=O)c2c3ccccc3n(C[C@H]4CCCC[N+]4C)c2)C	7.1
273	Fc1ccc2c(N(CCC2)C(=O)c3c4ccccc4n(C[C@H]5CCCC[N+]5C)c3)c1	7.1

274	O=S(=O)(c1c2ccccc2n(C[C@H]3CCCC[N+]3C)c1)c4c[nH]c5cc(N)ccc54	7.1
275	Clc1cncc(Cl)c1C(=O)c2c3ccccc3n(C[C@H]4CCCC[N+]4C)c2	7.1
276	O=C(c1c2ccccc2n(C[C@H]3CCCC[N+]3C)c1)c4c(O)ccc(c4)C	7.1
277	Clc1c(N)cccc1C(=O)c2c3ccccc3n(C[C@H]4CCCC[N+]4C)c2	7.1
278	Fc1cccc(C(=O)c2cn(C[C@H]3CCCC[N+]3C)c4ccccc42)c1NC	7.1
279	Fc1cc(F)ccc1C(=O)c2c3ccccc3n(C[C@H]4CCCC[N+]4C)c2	7.1
280	O=C(c1cn(C[C@H]2CCCC[N+]2C)c3ccccc13)c4cccc5cc6ccccc6cc54	7
281	O=C(CN1c2ccccc2C[C@@H]1C)c3cn(C[C@H]4CCCC[N+]4C)c5ccccc53	7
282	Clc1cccc(c1NC(=O)c2c3ccccc3n(C[C@H]4CCCC[N+]4C)c2)C	7
283	Clc1ccc(C(=O)c2c3ccccc3n(C[C@H]4CCCC[N+]4C)c2)cc1C	7
284	Fc1ccccc1C(=O)c2c3ccccc3n(C[C@H]4CCCC[N+]4C)c2	7
285	O=C(N1CCS[C@@H](C1)CC)c2c3ccccc3n(C[C@H]4CCCC[N+]4C)c2	7
286	O=C(N[C@@H]1c2ccccc2C[C@H]1C)c3cn(C[C@H]4CCCC[N+]4C)c5ccccc53	7
287	Clc1cc(F)c(C(=O)c2c3ccccc3n(C[C@H]4CCCC[N+]4C)c2)cc1	7
288	O=C(N1CCC[C@H](C1)CC)c2c3ccccc3n(C[C@H]4CCCC[N+]4C)c2	7
289	O=C(N(c1ccccn1)C)c2c3ccccc3n(C[C@H]4CCCC[N+]4C)c2	7
290	Fc1cccnc1C(=O)c2c3ccccc3n(C[C@H]4CCCC[N+]4C)c2	7
291	O=C(NCC(CC)CC)c1c2ccccc2n(C[C@H]3CCCC[N+]3C)c1	7
292	O=C([C@@H]1CSCCS1)c2c3ccccc3n(C[C@H]4CCCC[N+]4C)c2	7
293	O=C(c1cn(C[C@H]2CCCC[N+]2C)c3ccccc31)c4cccc5ccccc54	7
294	Brc1c(C)ccc(C(=O)c2c3ccccc3n(C[C@H]4CCCC[N+]4C)c2)c1	7
295	Oc1cc(OC)cc(C(=O)c2c3ccccc3n(C[C@H]4CCCC[N+]4C)c2)c1	7
296	O=C(c1c2ccccc2n(C[C@H]3CCCC[N+]3C)c1)c4ccnc5ccc(OC)cc54	7
297	Brc1cccc(C(=O)c2c3ccccc3n(C[C@H]4CCCC[N+]4C)c2)c1C	7
298	Clc1c(cc(O)c(C(=O)c2c3ccccc3n(C[C@H]4CCCC[N+]4C)c2)c1C)C	7
299	O=C(c1c2ccccc2n(C[C@H]3CCCC[N+]3C)c1)c4cc(cc(c4OC)C)C	7
300	O=C(N1c2ccccc2O[C@H](C1)C)c3cn(C[C@H]4CCCC[N+]4C)c5ccccc53	7
301	O=[S@@](Cc1cccc(c1)C(=O)c2c3ccccc3n(C[C@H]4CCCC[N+]4C)c2)C	7
302	O=S(=O)(c1c2ccccc2n(C[C@H]3CCCC[N+]3C)c1)c4cccs4	7
303	Clc1ccc2c(N(C[C@@H](O2)C)C(=O)c3c4ccccc4n(C[C@H]5CCCC[N+]5C)c3)c1	7
304	Clc1cccc(c1C(=O)c2c3ccccc3n(C[C@H]4CCCC[N+]4C)c2)C	7
305	Fc1cccc(C(=O)c2cn(C[C@H]3CCCC[N+]3C)c4ccccc42)c1N	7
306	O=C(c1c2ccccc2n(C[C@H]3CCCC[N+]3C)c1)c4c(C)ccs4	7
307	O=C(O[C@@H](C(C)C)C)c1c2ccccc2n(C[C@H]3CCCC[N+]3C)c1	7
308	Brc1ccc(F)cc1C(=O)c2c3ccccc3n(C[C@H]4CCCC[N+]4C)c2	7
309	O=C(c1c2ccccc2n(C[C@H]3CCCC[N+]3C)c1)c4cc(OC)cc(OC)c4	7
310	Brc1cc(C(=O)c2c3ccccc3n(C[C@H]4CCCC[N+]4C)c2)cs1	7
311	O=C([C@@H](SC)CC)c1c2ccccc2n(C[C@H]3CCCC[N+]3C)c1	7
312	O=C(c1c2ccccc2n(C[C@H]3CCCC[N+]3C)c1)c4csc5c4CC[C@H](C5)C	7
313	C[N+]1CCCC[C@@H]1Cn2cc(c3ccccc32)-c4non-5cc[nH]cc5s4	7
314	Brc1ccsc1S(=O)(=O)c2c3ccccc3n(C[C@H]4CCCC[N+]4C)c2	7
315	N=C(c1c2ccccc2n(C[C@H]3CCCC[N+]3C)c1)c4ccccc4	7

316	O=C(C1CCCCC1)c2c3ccccc3n(C[C@H]4CCCC[N+]4C)c2	7
317	Fc1cccc(c1NCC)C(=O)c2cn(C[C@H]3CCCC[N+]3C)c4ccccc42	7
318	Fc1cccc(C(=O)c2cn(C[C@H]3CCCC[N+]3C)c4ccccc42)c1NN	7
319	O=C(c1cn(C[C@H]2CCCC[N+]2C)c3ccccc31)c4ccccc4C(OC)=O	7
320	O=C(c1c2ccccc2n(C[C@H]3CCCC[N+]3C)c1)c4ccccc4C	7
321	O=C(N1c2ccccc2OCC1)c3cn(C[C@H]4CCCC[N+]4C)c5ccccc53	7
322	Brc1ccc(Cl)cc1C(=O)c2c3ccccc3n(C[C@H]4CCCC[N+]4C)c2	6.9
323	O=S(=O)(c1c2ccccc2n(C[C@H]3CCCC[N+]3C)c1)c4ccccc4CC	6.9
324	O=C(c1cn(C[C@H]2CCCC[N+]2C)c3ccccc31)c4c(-c5ccccc5)ccs4	6.9
325	O=C(N1c2ccccc2C[C@@H]([N+])C1)c3cn(C[C@H]4CCCC[N+]4C)c5ccccc53	6.9
326	O=C([C@@H]1C[C@H]2CC[C@@H]1O2)c3c4ccccc4n(C[C@H]5CCCC[N+]5C)c3	6.9
327	Clc1ccc2c(N(CCO2)C(=O)c3c4ccccc4n(C[C@H]5CCCC[N+]5C)c3)c1	6.9
328	O=C(c1c2ccccc2n(C[C@H]3CCCC[N+]3C)c1)c4cccnc4SC	6.9
329	Clc1cccc(Cl)c1OC(=O)c2c3ccccc3n(C[C@H]4CCCC[N+]4C)c2	6.9
330	Clc1cc(F)ccc1S(=O)(=O)c2c3ccccc3n(C[C@H]4CCCC[N+]4C)c2	6.9
331	C[N+]1CCCC[C@@H]1Cn2cc(Oc3cccc(CC)c3)c4ccccc42	6.9
332	O=C(Nc1ccccc1OC)c2cn(C[C@H]3CCCC[N+]3C)c4ccccc24	6.9
333	Clc1c(F)cccc1C(=O)c2cn(C[C@H]3CCCC[N+]3C)c4ccccc42	6.9
334	FC(F)CN(C1CC1)C(=O)c2c3ccccc3n(C[C@H]4CCCC[N+]4C)c2	6.9
335	Clc1ccccc1S(=O)(=O)c2c3ccccc3n(C[C@H]4CCCC[N+]4C)c2	6.9
336	Clc1cccc(F)c1CS(=O)(=O)c2c3ccccc3n(C[C@H]4CCCC[N+]4C)c2	6.9
337	Clc1cc(Cl)cc(O)c1C(=O)c2c3ccccc3n(C[C@H]4CCCC[N+]4C)c2	6.9
338	Fc1cnccc1C(=O)c2c3ccccc3n(C[C@H]4CCCC[N+]4C)c2	6.9
339	O=C(C1(CC1)c2cccc(c2)C)c3c4ccccc4n(C[C@H]5CCCC[N+]5C)c3	6.9
340	O=C(C[C@@H]1c2ccccc2CCO1)c3cn(C[C@H]4CCCC[N+]4C)c5ccccc53	6.9
341	Clc1ccc(C(=O)c2c3ccccc3n(C[C@H]4CCCC[N+]4C)c2)cc1OC	6.9
342	O=C(c1c2ccccc2n(C[C@H]3CCCC[N+]3C)c1)c4ccccc4C#N	6.9
343	O=C(c1c2ccccc2n(C[C@H]3CCCC[N+]3C)c1)c4cccc(C[N+](C)C)c4	6.9
344	O=C(c1c2ccccc2n(C[C@H]3CCCC[N+]3C)c1)c4ccccc4CC#N	6.9
345	FC(F)(F)c1ccccc1C(=O)c2c3ccccc3n(C[C@H]4CCCC[N+]4C)c2	6.9
346	O=C(N1CCc2cccc(c21)C)c3c4ccccc4n(C[C@H]5CCCC[N+]5C)c3	6.9
347	O=C([C@H]1c2ccsc2CCC1)c3c4ccccc4n(C[C@H]5CCCC[N+]5C)c3	6.9
348	Brc1ccc(Cl)c(C(=O)c2c3ccccc3n(C[C@H]4CCCC[N+]4C)c2)c1	6.9
349	O=C(Nc1ccccc1SC)c2cn(C[C@H]3CCCC[N+]3C)c4ccccc24	6.9
350	Fc1ccc(F)c(C(=O)c2c3ccccc3n(C[C@H]4CCCC[N+]4C)c2)c1OC	6.9
351	Clc1cccc(C2(CC2)C(=O)c3c4ccccc4n(C[C@H]5CCCC[N+]5C)c3)c1	6.9
352	O=C(c1cn(C[C@H]2CCCC[N+]2C)c3ccccc31)[C@H]4c5ccccc5CCO4	6.9
353	O=C(N(c1ccc(cc1)C)C)c2c3ccccc3n(C[C@H]4CCCC[N+]4C)c2	6.9
354	Clc1c(F)c(C(=O)c2c3ccccc3n(C[C@H]4CCCC[N+]4C)c2)ccn1	6.9
355	Oc1ccccc1C(=O)c2c3ccccc3n(C[C@H]4CCCC[N+]4C)c2	6.9
356	Clc1ccc(Cl)c(S(=O)(=O)c2c3ccccc3n(C[C@H]4CCCC[N+]4C)c2)c1	6.9
357	O=C(c1c2ccccc2n(C[C@H]3CCCC[N+]3C)c1)c4cccc(COC)c4	6.9

358	O=C([C@@H](C1CCCC1)C)c2c3ccccc3n(C[C@H]4CCCC[N+]4C)c2	6.8
359	O=C(c1c2ccccc2n(C[C@H]3CCCC[N+]3C)c1)C(c4ccccc4)=C	6.8
360	Fc1ccc2c(N(CCO2)C(=O)c3c4ccccc4n(C[C@H]5CCCC[N+]5C)c3)c1	6.8
361	O=S(=O)(c1cc(N)cc(c1)C(=O)c2c3ccccc3n(C[C@H]4CCCC[N+]4C)c2)C	6.8
362	Fc1c(C)ccc(C(=O)c2c3ccccc3n(C[C@H]4CCCC[N+]4C)c2)c1	6.8
363	O=S(=O)(c1c2ccccc2n(C[C@H]3CCCC[N+]3C)c1)c4ccccc4	6.8
364	O=C(c1cn(C[C@H]2CCCC[N+]2C)c3ccccc31)c4ccccc4-c5ncc[nH]5	6.8
365	Clc1cccc(Cl)c1OCC(=O)c2c3ccccc3n(C[C@H]4CCCC[N+]4C)c2	6.8
366	O=C(c1c2ccccc2n(C[C@H]3CCCC[N+]3C)c1)c4c(CC)ccs4	6.8
367	O=C(c1cn(C[C@H]2CCCC[N+]2C)c3ccccc31)c4cccc5c4nccn5	6.8
368	O=C(N1c2cnccc2OCC1)c3c4ccccc4n(C[C@H]5CCCC[N+]5C)c3	6.8
369	O=C(c1c2ccccc2n(C[C@H]3CCCC[N+]3C)c1)c4cccs4	6.8
370	O=C(c1c2ccccc2n(C[C@H]3CCCC[N+]3C)c1)c4cccc5cnccc54	6.8
371	O=C(C(CC)CC)c1c2ccccc2n(C[C@H]3CCCC[N+]3C)c1	6.8
372	FC(F)(F)c1cccnc1C(=O)c2c3ccccc3n(C[C@H]4CCCC[N+]4C)c2	6.8
373	O=C(c1csc2CCCCCc12)c3c4ccccc4n(C[C@H]5CCCC[N+]5C)c3	6.8
374	O=C(c1c2ccccc2n(C[C@H]3CCCC[N+]3C)c1)c4ccsc4N	6.8
375	Oc1ccc2c(SCO2)c1C(=O)c3c4ccccc4n(C[C@H]5CCCC[N+]5C)c3	6.8
376	Brc1ccccc1C(=O)c2c3ccccc3n(C[C@H]4CCCC[N+]4C)c2	6.8
377	Clc1ccc2c(C(C(=O)c3c4ccccc4n(C[C@H]5CCCC[N+]5C)c3)=CC(=O)N2)c1	6.8
378	O=C(N1c2ccc(cc2CCC1)C)c3c4ccccc4n(C[C@H]5CCCC[N+]5C)c3	6.8
379	O=C(c1c2ccccc2n(C[C@H]3CCCC[N+]3C)c1)C(CC(C)C)(C)C	6.8
380	O=C(N1CCC[C@H]1C(C)C)c2c3ccccc3n(C[C@H]4CCCC[N+]4C)c2	6.8
381	O=C(c1c2ccccc2n(C[C@H]3CCCC[N+]3C)c1)/C(OC)=C/c4ccccc4	6.8
382	O=C(c1cn(C[C@H]2CCCC[N+]2C)c3ccccc31)C4=CC(=O)Nc5ccccc54	6.8
383	O=C(c1c2ccccc2n(C[C@H]3CCCC[N+]3C)c1)/C(C)=C/C	6.8
384	FC(F)(F)c1cccc(c1N)C(=O)c2c3ccccc3n(C[C@H]4CCCC[N+]4C)c2	6.8
385	O=C(C1=CCCCC1)c2c3ccccc3n(C[C@H]4CCCC[N+]4C)c2	6.8
386	Brc1cccc(C(=O)c2c3ccccc3n(C[C@H]4CCCC[N+]4C)c2)c1	6.8
387	Clc1ccc2c(CCCN2C(=O)c3c4ccccc4n(C[C@H]5CCCC[N+]5C)c3)c1	6.8
388	O=C(N[C@H]1CC=CCC1)c2c3ccccc3n(C[C@H]4CCCC[N+]4C)c2	6.7
389	Sc1c(C(=O)c2c3ccccc3n(C[C@H]4CCCC[N+]4C)c2)cccn1	6.7
390	Brc1ccc(OC)cc1C(=O)c2c3ccccc3n(C[C@H]4CCCC[N+]4C)c2	6.7
391	O=C([C@@H](C1CC1)C)c2c3ccccc3n(C[C@H]4CCCC[N+]4C)c2	6.7
392	O=C(Oc1ccccc1C)c2c3ccccc3n(C[C@H]4CCCC[N+]4C)c2	6.7
393	Clc1ccc(SC)cc1C(=O)c2c3ccccc3n(C[C@H]4CCCC[N+]4C)c2	6.7
394	O=C(N(c1cccc(c1)C)C)c2c3ccccc3n(C[C@H]4CCCC[N+]4C)c2	6.7
395	FC(F)(F)[C@H]1CCC[C@H](C1)C(=O)c2c3ccccc3n(C[C@H]4CCCC[N+]4C)c2	6.7
396	O=C(C1CCCCCC1)c2c3ccccc3n(C[C@H]4CCCC[N+]4C)c2	6.7
397	Clc1cccc(F)c1C(=O)c2c3ccccc3n(C[C@H]4CCCC[N+]4C)c2	6.7
398	Fc1ccc2c(OCCN2C(=O)c3c4ccccc4n(C[C@H]5CCCC[N+]5C)c3)c1	6.7
399	Fc1cc(F)c(F)c(C(=O)c2c3ccccc3n(C[C@H]4CCCC[N+]4C)c2)c1F	6.7

400	O=C(c1c2ccccc2n(C[C@H]3CCCC[N+]3C)c1)c4cccc(OC(C)C)c4	6.7
401	Fc1c(F)cccc1C(=O)c2cn(C[C@H]3CCCC[N+]3C)c4ccccc42	6.7
402	O=S(=O)(c1c2ccccc2n(C[C@H]3CCCC[N+]3C)c1)Cc4cccc(OC)c4	6.7
403	O=C(c1c2ccccc2n(C[C@H]3CCCC[N+]3C)c1)c4cccc(CC[N+])c4	6.7
404	O=C(c1c2ccccc2n(C[C@H]3CCCC[N+]3C)c1)c4csc(c4)C	6.7
405	O=S(=O)(c1c2ccccc2n(C[C@H]3CCCC[N+]3C)c1)c4c[nH]c5ccccc54	6.7
406	O=C(c1c2ccccc2n(C[C@H]3CCCC[N+]3C)c1)c4c(C)ccc(c4)C	6.7
407	FC[C@@H]1CCCN1S(=O)(=O)c2c3ccccc3n(C[C@H]4CCCC[N+]4C)c2	6.7
408	Clc1cccc(Cl)c1CS(=O)(=O)c2c3ccccc3n(C[C@H]4CCCC[N+]4C)c2	6.7
409	Fc1ccc(F)c(C(=O)C(=O)c2c3ccccc3n(C[C@H]4CCCC[N+]4C)c2)c1	6.7
410	O=C(N(CC(C)C)C)c1c2ccccc2n(C[C@H]3CCCC[N+]3C)c1	6.7
411	Fc1cccc(F)c1C(=O)c2c3ccccc3n(C[C@H]4CCCC[N+]4C)c2	6.7
412	O=C(c1c2ccccc2n(C[C@H]3CCCC[N+]3C)c1)c4ccsc4	6.7
413	Clc1ccc(c(C(=O)c2c3ccccc3n(C[C@H]4CCCC[N+]4C)c2)c1)C	6.7
414	OC1(CCCCC1)C(=O)c2c3ccccc3n(C[C@H]4CCCC[N+]4C)c2	6.7
415	O=C(c1c2ccccc2n(C[C@H]3CCCC[N+]3C)c1)c4cccc(c4)C=C	6.7
416	Clc1cccc(C(=O)c2c3ccccc3n(C[C@H]4CCCC[N+]4C)c2)c1O	6.7
417	C[N+]1CCCC[C@@H]1Cn2cc(O[C@H]3c4ccccc4CCC3)c5ccccc52	6.7
418	O=C([C@H]1CCCC[C@@H]1C)c2c3ccccc3n(C[C@H]4CCCC[N+]4C)c2	6.7
419	O=C(NC1CCCCCC1)c2c3ccccc3n(C[C@H]4CCCC[N+]4C)c2	6.6
420	O=C(c1c2ccccc2n(C[C@H]3CCCC[N+]3C)c1)c4cccc(C[N+])c4	6.6
421	O=C([C@@H](CC1CCCC1)C)c2c3ccccc3n(C[C@H]4CCCC[N+]4C)c2	6.6
422	O=C(c1c2ccccc2n(C[C@H]3CCCC[N+]3C)c1)c4cccc(OCC)c4	6.6
423	O=C(c1c2ccccc2n(C[C@H]3CCCC[N+]3C)c1)c4c(SC)nsc4SC	6.6
424	O=C(c1c2ccccc2n(C[C@H]3CCCC[N+]3C)c1)c4csc(CCC)c4	6.6
425	Clc1ccccc1[C@@H](O)c2c3ccccc3n(C[C@H]4CCCC[N+]4C)c2	6.6
426	O=C(c1c(cccc1[N+]([O-])=O)C)c2c3ccccc3n(C[C@H]4CCCC[N+]4C)c2	6.6
427	Clc1ccc(Cl)cc1Oc2c3ccccc3n(C[C@H]4CCCC[N+]4C)c2	6.6
428	O=C([C@H]1c2ccccc2CC[N+]1)c3cn(C[C@H]4CCCC[N+]4C)c5ccccc53	6.6
429	Fc1cccc(c1C)C(=O)c2cn(C[C@H]3CCCC[N+]3C)c4ccccc42	6.6
430	O=C([C@@H](C(C)C)C)c1c2ccccc2n(C[C@H]3CCCC[N+]3C)c1	6.6
431	O=C(c1c2ccccc2n(C[C@H]3CCCC[N+]3C)c1)c4cncnc4C(C)C	6.6
432	O=C(c1c2ccccc2n(C[C@H]3CCCC[N+]3C)c1)c4cccc(c4)C(=O)N	6.6
433	Clc1ccccc1CC(=O)c2c3ccccc3n(C[C@H]4CCCC[N+]4C)c2	6.6
434	Clc1ccc(-n2cccn2)cc1C(=O)c3c4ccccc4n(C[C@H]5CCCC[N+]5C)c3	6.6
435	O[C@@H](c1c2ccccc2n(C[C@H]3CCCC[N+]3C)c1)c4cccc(c4)C	6.6
436	Fc1c(F)ccc(C(=O)c2c3ccccc3n(C[C@H]4CCCC[N+]4C)c2)c1F	6.5
437	Fc1ccccc1OCC(=O)c2c3ccccc3n(C[C@H]4CCCC[N+]4C)c2	6.5
438	O=C([C@H]1CCC[C@H]([N+])[C@@H]1C)c2c3ccccc3n(C[C@H]4CCCC[N+]4C)c2	6.5
439	O=C(C[C@H](CCC)C)c1c2ccccc2n(C[C@H]3CCCC[N+]3C)c1	6.5
440	O=C(c1c2ccccc2n(C[C@H]3CCCC[N+]3C)c1)c4cccnc4SC(C)C	6.5
441	O=S(=O)(C[C@H]1CCCO1)c2c3ccccc3n(C[C@H]4CCCC[N+]4C)c2	6.5

442	Fc1ccc(C(=O)C(=O)c2c3ccccc3n(C[C@H]4CCCC[N+]4C)c2)cc1	6.5
443	O=C(c1c2ccccc2n(C[C@H]3CCCC[N+]3C)c1)c4c(ncc(N)c4)C	6.5
444	Fc1ccccc1[C@@H](O)c2c3ccccc3n(C[C@H]4CCCC[N+]4C)c2	6.5
445	O=C(NC1CCCCC1)c2c3ccccc3n(C[C@H]4CCCC[N+]4C)c2	6.5
446	O=S(=O)(c1c2ccccc2n(C[C@H]3CCCC[N+]3C)c1)c4cc[nH]c4	6.5
447	Oc1c(OC)cccc1C(=O)c2c3ccccc3n(C[C@H]4CCCC[N+]4C)c2	6.4
448	O=C(Nc1ccccc1CC)c2cn(C[C@H]3CCCC[N+]3C)c4ccccc42	6.4
449	Oc1ccc(OC)cc1C(=O)c2c3ccccc3n(C[C@H]4CCCC[N+]4C)c2	6.4
450	O=C(c1c2ccccc2n(C[C@H]3CCCC[N+]3C)c1)c4cncnc4C	6.4
451	O=C(c1c2ccccc2n(C[C@H]3CCCC[N+]3C)c1)[C@@H](CC#C)C	6.4
452	Fc1ccc(F)c(C(=O)c2c3ccccc3n(C[C@H]4CCCC[N+]4C)c2)c1F	6.4
453	O=C(C[C@H](c1ccccc1)C)c2c3ccccc3n(C[C@H]4CCCC[N+]4C)c2	6.4
454	Oc1ccc(C(=O)c2c3ccccc3n(C[C@H]4CCCC[N+]4C)c2)c(c1)C	6.4
455	Clc1ccc(F)cc1C(=O)c2c3ccccc3n(C[C@H]4CCCC[N+]4C)c2	6.4
456	Clc1ccc(S(=O)(=O)C)cc1C(=O)c2c3ccccc3n(C[C@H]4CCCC[N+]4C)c2	6.4
457	O=C(c1c2ccccc2n(C[C@H]3CCCC[N+]3C)c1)[C@@H](OCC=C)C	6.4
458	O=C([C@H]1CCCC[C@H]1C[N+])c2c3ccccc3n(C[C@H]4CCCC[N+]4C)c2	6.4
459	O=C(N[C@@H]1CCCC[C@H]1C[N+])c2c3ccccc3n(C[C@H]4CCCC[N+]4C)c2	6.4
460	Fc1c(ccc(F)c1C(=O)c2c3ccccc3n(C[C@H]4CCCC[N+]4C)c2)C	6.4
461	O=S(=O)(Nc1ccccc1C)c2cn(C[C@H]3CCCC[N+]3C)c4ccccc42	6.4
462	O=C(N[C@@H]1C=CCCC1)c2c3ccccc3n(C[C@H]4CCCC[N+]4C)c2	6.3
463	Oc1ccc(C(=O)c2c3ccccc3n(C[C@H]4CCCC[N+]4C)c2)cc1OC	6.3
464	O=C([C@H]([N+]1CCCCCC1)C)c2cn(C[C@H]3CCCC[N+]3C)c4ccccc42	6.3
465	O=C(N1c2cc(OC)ccc2OCC1)c3c4ccccc4n(C[C@H]5CCCC[N+]5C)c3	6.3
466	Clc1cccc([C@@H](O)c2c3ccccc3n(C[C@H]4CCCC[N+]4C)c2)c1	6.3
467	O=C([C@@H](OCCC)C)c1c2ccccc2n(C[C@H]3CCCC[N+]3C)c1	6.3
468	O=S(=O)(c1cn(C[C@H]2CCCC[N+]2C)c3ccccc31)Cc4ccccc4C	6.3
469	O=C(N[C@H](c1cccs1)C)c2c3ccccc3n(C[C@H]4CCCC[N+]4C)c2	6.2
470	O=C(c1cn(C[C@H]2CCCC[N+]2C)c3ccccc31)Cc4ccccc4C	6.2
471	Brc1c(C(=O)c2c3ccccc3n(C[C@H]4CCCC[N+]4C)c2)cccn1	6.2
472	O=C(Nc1c(C)csc1)c2c3ccccc3n(C[C@H]4CCCC[N+]4C)c2	6.2
473	O=C(C[C@@H]1C=CCC1)c2c3ccccc3n(C[C@H]4CCCC[N+]4C)c2	6.2
474	O=C(c1c2ccccc2n(C[C@H]3CCCC[N+]3C)c1)/C=C/C(C)(C)C	6.2
475	Fc1ccc(c(NC(=O)c2c3ccccc3n(C[C@H]4CCCC[N+]4C)c2)c1)C	6.2
476	O=C([C@@H]1[C@](C1)(CC)C)c2c3ccccc3n(C[C@H]4CCCC[N+]4C)c2	6.2
477	O=C(Nc1ccc([nH+]c1C)N)c2c3ccccc3n(C[C@H]4CCCC[N+]4C)c2	6.1
478	O=C(CC1CCCCC1)c2c3ccccc3n(C[C@H]4CCCC[N+]4C)c2	6.1
479	Clc1cc(Cl)ccc1CS(=O)(=O)c2c3ccccc3n(C[C@H]4CCCC[N+]4C)c2	6.1
480	O=C(C[C@H]1C[C@H]2CC[C@@H]1C2)c3c4ccccc4n(C[C@H]5CCCC[N+]5C)c3	6.1
481	Fc1ccc(c(C(=O)c2c3ccccc3n(C[C@H]4CCCC[N+]4C)c2)c1)C	6.1
482	O=C([C@@H]1CCC[C@@H](C1)C)c2c3ccccc3n(C[C@H]4CCCC[N+]4C)c2	6
483	O=C(c1c2ccccc2n(C[C@H]3CCCC[N+]3C)c1)c4cnccc4C	6

484	O=C(c1c2ccccc2n(C[C@H]3CCCC[N+]3C)c1)/C=C/C(C)C	6
485	O=C([C@@]1(C[C@@H]1C[N+])c2cccs2)c3c4ccccc4n(C[C@H]5CCCC[N+]5C)c3	6
486	Fc1ccc([C@@H](O)C(=O)c2c3ccccc3n(C[C@H]4CCCC[N+]4C)c2)cc1C	5.9
487	O=C([C@H]1[C@@H](CCC[N+]1)C)c2cn(C[C@H]3CCCC[N+]3C)c4ccccc42	5.9
488	O=C(c1c2ccccc2n(C[C@H]3CCCC[N+]3C)c1)/C=C/CC	5.9
489	O=C(c1c2ccccc2n(C[C@H]3CCCC[N+]3C)c1)CCc4ccccc4	5.9
490	O=C(CC1CCCCCC1)c2c3ccccc3n(C[C@H]4CCCC[N+]4C)c2	5.9
491	O=C([C@H]1c2cccc(c2C[N+]C1)C)c3c4ccccc4n(C[C@H]5CCCC[N+]5C)c3	5.9
492	O=C(c1c2ccccc2n(C[C@H]3CCCC[N+]3C)c1)COCC(C)C	5.8
493	O=C(CC(CC)CC)c1c2ccccc2n(C[C@H]3CCCC[N+]3C)c1	5.7
494	Cl[C@@H](/C=C/C(=O)c1c2ccccc2n(C[C@H]3CCCC[N+]3C)c1)C	5.7
495	O=C(CC1CCC1)c2c3ccccc3n(C[C@H]4CCCC[N+]4C)c2	5.6
496	O=C([C@H]1CCCC[C@@H]1[N+])c2c3ccccc3n(C[C@H]4CCCC[N+]4C)c2	5.6
497	O=C(N[C@H]1CCSC1)c2c3ccccc3n(C[C@H]4CCCC[N+]4C)c2	5.6
498	O=C(c1c2ccccc2n(C[C@H]3CCCC[N+]3C)c1)Cc4ccsc4	5.4
499	FC(F)(F)[C@@H](CC(=O)c1c2ccccc2n(C[C@H]3CCCC[N+]3C)c1)C	5.3
500	O=C([C@H]([N+])c1ccc(CC)cc1)c2c3ccccc3n(C[C@H]4CCCC[N+]4C)c2	4.5

Table S14. List, SMILE and predicted pK_i values for Series 4 in CB_2 receptor.

N°	SMILES	Pred pK_i
1	O=C(C1C(C1(C)C)(C)C)c2c3ccccc3n(Cc4ccc(cc4)C#N)c2	10.2
2	O=C(C1C(C1(C)C)(C)C)c2c3ccccc3n(CC[C@H]4CCC(=O)N4)c2	10.1
3	O=S1(=O)CC[C@H](C1)CCn2cc(C(=O)C3C(C3(C)C)(C)C)c4ccccc42	10.1
4	O=C(C1C(C1(C)C)(C)C)c2c3ccccc3n(CC[C@@H]4C(C(OC4)=O)=C)c2	10
5	O=C(C1C(C1(C)C)(C)C)c2c3ccccc3n(Cc4ccc5c(non5)c4)c2	10
6	O=C(C1C(C1(C)C)(C)C)c2cn(c3ccccc32)CCSS([O-])(=O)=O	9.9
7	Clc1cc(Cn2cc(C(=O)C3C(C3(C)C)(C)C)c4ccccc42)ccn1	9.8
8	O=C(C1C(C1(C)C)(C)C)c2c3ccccc3n(CCc4ccncc4)c2	9.8
9	Fc1c(F)ccc(Cn2cc(C(=O)C3C(C3(C)C)(C)C)c4ccccc42)c1	9.7
10	O=C(C1C(C1(C)C)(C)C)c2c3ccccc3n(CC[C@@H]([S@](=O)C)C)c2	9.6
11	O=S(=O)(CCCn1cc(C(=O)C2C(C2(C)C)(C)C)c3ccccc31)C	9.6
12	Clc1c(F)ccc(Cn2cc(C(=O)C3C(C3(C)C)(C)C)c4ccccc42)c1	9.6
13	FC(F)(F)C[C@@H](O)Cn1cc(C(=O)C2C(C2(C)C)(C)C)c3ccccc31	9.6
14	O=C(C1C(C1(C)C)(C)C)c2c3ccccc3n(Cc4ccncc4)c2	9.6
15	O=C(C1C(C1(C)C)(C)C)c2c3ccccc3n(CCCC=4[N-]N=NN4)c2	9.6
16	Fc1ccc(Cn2cc(C(=O)C3C(C3(C)C)(C)C)c4ccccc42)cc1	9.6
17	FC1(F)CCC(CC1)Cn2cc(C(=O)C3C(C3(C)C)(C)C)c4ccccc42	9.6
18	Brc1cc(Cn2cc(C(=O)C3C(C3(C)C)(C)C)c4ccccc42)cs1	9.5
19	Fc1ccc(Cn2cc(C(=O)C3C(C3(C)C)(C)C)c4ccccc42)cc1OC	9.5
20	O[C@H](C1CC1)Cn2cc(C(=O)C3C(C3(C)C)(C)C)c4ccccc42	9.5
21	O=C(C1C(C1(C)C)(C)C)c2c3ccccc3n(Cc4cnccc4)c2	9.5

22	O=C(C1C(C1(C)C)(C)C)c2c3ccccc3n(CC4CCSCC4)c2	9.5
23	O=C(C1C(C1(C)C)(C)C)c2c3ccccc3n(C[C@H](CC(C)(C)C)C)c2	9.5
24	O=C(C1C(C1(C)C)(C)C)c2c3ccccc3n(CCCc4c(noc4C)C)c2	9.5
25	O[C@H]([C@@H](CC)C)Cn1cc(C(=O)C2C(C2(C)C)(C)C)c3ccccc31	9.5
26	O=C(C1C(C1(C)C)(C)C)c2c3ccccc3n(C[C@H](c4ccsc4)C)c2	9.4
27	Fc1ccc(Cn2cc(C(=O)C3C(C3(C)C)(C)C)c4ccccc42)cc1C	9.4
28	O=C(C1C(C1(C)C)(C)C)c2c3ccccc3n(CCCN(C)C(=O)C)c2	9.4
29	O=C(C1C(C1(C)C)(C)C)c2c3ccccc3n(Cc4csc([N+]([O-])=O)c4)c2	9.4
30	ClC(CCn1cc(C(=O)C2C(C2(C)C)(C)C)c3ccccc31)(C)C	9.4
31	Fc1c(F)c(F)cc(Cn2cc(C(=O)C3C(C3(C)C)(C)C)c4ccccc42)c1	9.4
32	O=C(C1C(C1(C)C)(C)C)c2cn(CCC(C)(C)C#N)c3ccccc32	9.4
33	S=C(N)CCCn1cc(C(=O)C2C(C2(C)C)(C)C)c3ccccc31	9.4
34	Clc1cccc(Cn2cc(C(=O)C3C(C3(C)C)(C)C)c4ccccc42)c1	9.3
35	Fc1c(F)ccc(Sn2cc(C(=O)C3C(C3(C)C)(C)C)c4ccccc42)c1	9.3
36	Fc1ccc(O)c(Cn2cc(C(=O)C3C(C3(C)C)(C)C)c4ccccc42)c1	9.3
37	Fc1cn(nn1)Cn2cc(C(=O)C3C(C3(C)C)(C)C)c4ccccc42	9.3
38	O=C(C1C(C1(C)C)(C)C)c2c3ccccc3n(CCCc4cn[nH]c4C)c2	9.3
39	Clc1ccc(s1)Cn2cc(C(=O)C3C(C3(C)C)(C)C)c4ccccc42	9.3
40	O=C(C1C(C1(C)C)(C)C)c2c3ccccc3n(Cc4cnc(O)cc4)c2	9.3
41	O=C(C1C(C1(C)C)(C)C)c2c3ccccc3n(CC[C@@H]4COCCC4)c2	9.3
42	Fc1c(O)ccc(Cn2cc(C(=O)C3C(C3(C)C)(C)C)c4ccccc42)c1	9.3
43	Fc1ccc(CCn2cc(C(=O)C3C(C3(C)C)(C)C)c4ccccc42)cc1C	9.3
44	FC(F)(F)CCNn1cc(C(=O)C2C(C2(C)C)(C)C)c3ccccc31	9.3
45	O=C(C1C(C1(C)C)(C)C)c2cn(SCc3cnccc3)c4ccccc42	9.2
46	FC(SCn1cc(C(=O)C2C(C2(C)C)(C)C)c3ccccc31)(F)F	9.2
47	O=C(C1C(C1(C)C)(C)C)c2c3ccccc3n(CCCCC#N)c2	9.2
48	O=C(C1C(C1(C)C)(C)C)c2cn(CCSCCOC)c3ccccc32	9.2
49	O=C(C1C(C1(C)C)(C)C)c2c3ccccc3n(CCC4CCC4)c2	9.2
50	O=S(=O)(CCCn1cc(C(=O)C2C(C2(C)C)(C)C)c3ccccc31)CC#C	9.2
51	FC(F)(F)CCCn1cc(C(=O)C2C(C2(C)C)(C)C)c3ccccc31	9.2
52	Clc1ccc(Cn2cc(C(=O)C3C(C3(C)C)(C)C)c4ccccc42)cc1	9.2
53	O=C(C1C(C1(C)C)(C)C)c2c3ccccc3n(CC[C@@H](C(C)C)C#N)c2	9.2
54	Clc1c(F)ccc(Sn2cc(C(=O)C3C(C3(C)C)(C)C)c4ccccc42)c1	9.2
55	O=C(C1C(C1(C)C)(C)C)c2c3ccccc3n(CCC4=CCCCC4)c2	9.2
56	O=C(C1C(C1(C)C)(C)C)c2c3ccccc3n(Cc4ccsc4)c2	9.2
57	O=C(C1C(C1(C)C)(C)C)c2c3ccccc3n(COc4ccncc4)c2	9.2
58	O=C(C1C(C1(C)C)(C)C)c2c3ccccc3n(COCc4ccncc4)c2	9.2
59	O=C(C1C(C1(C)C)(C)C)c2c3ccccc3n(CSCCOC)c2	9.2
60	O=C(C1C(C1(C)C)(C)C)c2c3ccccc3n(CCCN(S(=O)(=O)C)C)c2	9.2
61	Fc1cc(Cn2cc(C(=O)C3C(C3(C)C)(C)C)c4ccccc42)ccn1	9.2
62	O=C(C1C(C1(C)C)(C)C)c2c3ccccc3n(COC4CCOCC4)c2	9.2
63	O=C(C1C(C1(C)C)(C)C)c2cn(c3ccccc32)CCC(C)(C)C	9.1

64	O=C(C1C(C1(C)C)(C)C)c2cn(Sc3ccncc3)c4ccccc42	9.1
65	O=C(C1C(C1(C)C)(C)C)c2c3ccccc3n(CC[C@@H]4CCOC4)c2	9.1
66	O=S(=O)(CCS(=O)(=O)n1cc(C(=O)C2C(C2(C)C)(C)C)c3ccccc31)C	9.1
67	FC(Sn1cc(C(=O)C2C(C2(C)C)(C)C)c3ccccc31)(F)C(F)F	9.1
68	O=C(C1C(C1(C)C)(C)C)c2cn(NCCC(C)(C)C)c3ccccc32	9.1
69	O=C(C1C(C1(C)C)(C)C)c2c3ccccc3n(CC4CCCC4)c2	9.1
70	O=C(C1C(C1(C)C)(C)C)c2cn(OCc3ccsc3)c4ccccc42	9.1
71	FCCCCCn1cc(C(=O)C2C(C2(C)C)(C)C)c3ccccc31	9.1
72	O=C(C1C(C1(C)C)(C)C)c2cn(SCCS(=O)(=O)C)c3ccccc32	9.1
73	Clc1ccc(Cn2cc(C(=O)C3C(C3(C)C)(C)C)c4ccccc42)cc1F	9.1
74	O[C@H](C1CC1)CCn2cc(C(=O)C3C(C3(C)C)(C)C)c4ccccc42	9.1
75	O=C(C1C(C1(C)C)(C)C)c2cn(Sc3cnc(cn3)C#N)c4ccccc42	9.1
76	O=C(C1C(C1(C)C)(C)C)c2c3ccccc3n(CC[C@@H](C)C#N)c2	9.1
77	FC1(F)CC(C1)Cn2cc(C(=O)C3C(C3(C)C)(C)C)c4ccccc42	9.1
78	O=C(C1C(C1(C)C)(C)C)c2c3ccccc3n(CCn4ccc(n4)C)c2	9.1
79	Fc1c(C)ccc(Cn2cc(C(=O)C3C(C3(C)C)(C)C)c4ccccc42)c1	9.1
80	O=C(C1C(C1(C)C)(C)C)c2c3ccccc3n(CC[C@H]4CCC[C@H](C4)C)c2	9.1
81	O=C(C1C(C1(C)C)(C)C)c2cn(CC[C@@H]3C[C@H]4CC[C@@H]3C4)c5ccccc52	9.1
82	O=C(C1C(C1(C)C)(C)C)c2c3ccccc3n(CCSC(C)(C)C)c2	9.1
83	FC(F)(Cn1cc(C(=O)C2C(C2(C)C)(C)C)c3ccccc31)[C@H](F)C(F)(F)F	9.1
84	O=C(C1C(C1(C)C)(C)C)c2c3ccccc3n(CC/C=C(\CO)C)c2	9.1
85	O=C(C1C(C1(C)C)(C)C)c2c3ccccc3n(CC(CC)CC)c2	9.1
86	O=C(C1C(C1(C)C)(C)C)c2c3ccccc3n(CCCCC=C)c2	9.1
87	SC(=S)NCCn1cc(C(=O)C2C(C2(C)C)(C)C)c3ccccc31	9.1
88	Fc1cccc(CSn2cc(C(=O)C3C(C3(C)C)(C)C)c4ccccc42)c1	9
89	O=C(C1C(C1(C)C)(C)C)c2c3ccccc3n(CCCSCC#N)c2	9
90	Fc1ccc([C@H](O)n2cc(C(=O)C3C(C3(C)C)(C)C)c4ccccc42)cc1	9
91	O=C(C1C(C1(C)C)(C)C)c2c3ccccc3n(CCc4ccc[nH]4)c2	9
92	O=C(C1C(C1(C)C)(C)C)c2c3ccccc3n(Cc4cc(C(OC)=O)co4)c2	9
93	O=C(C1C(C1(C)C)(C)C)c2c3ccccc3n(Cc4ccc(o4)C=O)c2	9
94	Fc1c(F)ccc(CCn2cc(C(=O)C3C(C3(C)C)(C)C)c4ccccc42)c1	9
95	O=C(C1C(C1(C)C)(C)C)c2c3ccccc3n(Cc4ccco4)c2	9
96	O=C(C1C(C1(C)C)(C)C)c2c3ccccc3n(CC[C@H](CC)C)c2	9
97	O=C(C1C(C1(C)C)(C)C)c2c3ccccc3n(Cc4ccncn4)c2	9
98	FC(F)(F)OCCn1cc(C(=O)C2C(C2(C)C)(C)C)c3ccccc31	9
99	O=C(C1C(C1(C)C)(C)C)c2c3ccccc3n(CCc4ccco4)c2	9
100	O=C(C1C(C1(C)C)(C)C)c2c3ccccc3n(C[C@H]4[C@@H](C4)CO)c2	9
101	O=C(C1C(C1(C)C)(C)C)c2c3ccccc3n(CC4=CC[C@@H](CC4)C)c2	9
102	Fc1cc(F)ccc1Cn2cc(C(=O)C3C(C3(C)C)(C)C)c4ccccc42	9
103	Clc1cccc(Sn2cc(C(=O)C3C(C3(C)C)(C)C)c4ccccc42)c1	9
104	O=C(C1C(C1(C)C)(C)C)c2c3ccccc3n(CSC(C)C)c2	9
105	O=C(C1C(C1(C)C)(C)C)c2c3ccccc3n(CCSC4CCOCC4)c2	9

106	Fc1cccc(Cn2cc(C(=O)C3C(C3(C)C)(C)C)c4ccccc42)c1	9
107	O=C(C1C(C1(C)C)(C)C)c2c3ccccc3n(CNc4ccncn4)c2	9
108	O=C(C1C(C1(C)C)(C)C)c2c3ccccc3n(CCCC(C)(C)C)c2	9
109	FC(F)(F)CSCn1cc(C(=O)C2C(C2(C)C)(C)C)c3ccccc31	9
110	O=C(C1C(C1(C)C)(C)C)c2cn(NCc3cccs3)c4ccccc42	9
111	O=C(C1C(C1(C)C)(C)C)c2c3ccccc3n(C[C@@H]4CC[C@H](C4)C)c2	9
112	O=C(C1C(C1(C)C)(C)C)c2c3ccccc3n(Cc4ccnc(c4)C)c2	9
113	Clc1ccc(Sn2cc(C(=O)C3C(C3(C)C)(C)C)c4ccccc42)cc1	9
114	O=C(C1C(C1(C)C)(C)C)c2c3ccccc3n(CCCC(C)C)c2	9
115	O=C(C1C(C1(C)C)(C)C)c2cn(c3ccccc32)CCSCC=C	9
116	O=C(C1C(C1(C)C)(C)C)c2cn(SCc3cccs3)c4ccccc42	8.9
117	FC(F)(COCn1cc(C(=O)C2C(C2(C)C)(C)C)c3ccccc31)C(F)F	8.9
118	O=C(C1C(C1(C)C)(C)C)c2c3ccccc3n(CSC4CCOCC4)c2	8.9
119	O=C(C1C(C1(C)C)(C)C)c2c3ccccc3n(CCCCSC)c2	8.9
120	O=C(C1C(C1(C)C)(C)C)c2c3ccccc3n(CC[C@@H](OC)C)c2	8.9
121	O=C(C1C(C1(C)C)(C)C)c2cn(SCC(C)C)c3ccccc32	8.9
122	O=C(C1C(C1(C)C)(C)C)c2c3ccccc3n(CCC4CCC(CC4)C)c2	8.9
123	O=C(C1C(C1(C)C)(C)C)c2cn(Sc3cc(ncn3)C)c4ccccc42	8.9
124	O=C(C1C(C1(C)C)(C)C)c2c3ccccc3n(CSC4CCCC4)c2	8.9
125	O=C(C1C(C1(C)C)(C)C)c2c3ccccc3n(Cc4ccoc4)c2	8.9
126	O=C(C1C(C1(C)C)(C)C)c2c3ccccc3n(CC4=CCCCC4)c2	8.9
127	Fc1c(F)ccc(CSn2cc(C(=O)C3C(C3(C)C)(C)C)c4ccccc42)c1	8.9
128	O=C(C1C(C1(C)C)(C)C)c2c3ccccc3n(C[C@H](C4CC4)C)c2	8.9
129	O=C(C1C(C1(C)C)(C)C)c2c3ccccc3n(C[n+]4ccc(C)cc4)c2	8.9
130	O=C(C1C(C1(C)C)(C)C)c2c3ccccc3n(CCS(=O)(=O)C)c2	8.9
131	O=C(C1C(C1(C)C)(C)C)c2cn(SC3CCCCC3)c4ccccc42	8.9
132	Fc1cccc(Sn2cc(C(=O)C3C(C3(C)C)(C)C)c4ccccc42)c1	8.9
133	O=C(C1C(C1(C)C)(C)C)c2c3ccccc3n(C[S@@](=O)[C@H](CC)C)c2	8.9
134	O=C(C1C(C1(C)C)(C)C)c2c3ccccc3n(C[C@H](CCC)C)c2	8.9
135	Fc1ccc(Sn2cc(C(=O)C3C(C3(C)C)(C)C)c4ccccc42)cc1	8.9
136	O=C(C1C(C1(C)C)(C)C)c2c3ccccc3n(CCC(C)C)c2	8.9
137	O=C(C1C(C1(C)C)(C)C)c2c3ccccc3n(CCC4CCCC4)c2	8.9
138	O=C(C1C(C1(C)C)(C)C)c2c3ccccc3n(C[N+]CCS([O-])(=O)=O)c2	8.9
139	O=C(C1C(C1(C)C)(C)C)c2c3ccccc3n(CCCc4cnccc4)c2	8.9
140	O=C(C1C(C1(C)C)(C)C)c2c3ccccc3n(C[N+]C(C)(C)C)c2	8.9
141	Brc1ccc[n+](Cn2cc(C(=O)C3C(C3(C)C)(C)C)c4ccccc42)c1	8.9
142	O=C(C1C(C1(C)C)(C)C)c2c3ccccc3n(CC4CC(C4)C)c2	8.9
143	FC(F)(F)COCn1cc(C(=O)C2C(C2(C)C)(C)C)c3ccccc31	8.9
144	O=C(C1C(C1(C)C)(C)C)c2cn(SCCc3ccncc3)c4ccccc42	8.9
145	O=C(C1C(C1(C)C)(C)C)c2c3ccccc3n(CCSSC)c2	8.9
146	O=C(C1C(C1(C)C)(C)C)c2c3ccccc3n(Cc4cscc4C)c2	8.9
147	O=C(C1C(C1(C)C)(C)C)c2cn(Sc3ccc(o3)C=O)c4ccccc42	8.9

148	O=C(C1C(C1(C)C)(C)C)c2c3ccccc3n(CC[C@H](CCC)C#N)c2	8.9
149	Clc1cnn(Cn2cc(C(=O)C3C(C3(C)C)(C)C)c4ccccc42)c1	8.9
150	O=C(C1C(C1(C)C)(C)C)c2c3ccccc3n(CCC[C@@H]4CCOC4)c2	8.9
151	O=C(C1C(C1(C)C)(C)C)c2c3ccccc3n(CSCC(C)C)c2	8.8
152	FC(F)CCn1cc(C(=O)C2C(C2(C)C)(C)C)c3ccccc31	8.8
153	O=C(C1C(C1(C)C)(C)C)c2cn(SCCC(C)C)c3ccccc32	8.8
154	FC(SCCn1cc(C(=O)C2C(C2(C)C)(C)C)c3ccccc31)(F)F	8.8
155	Clc1cc(F)ccc1Sn2cc(C(=O)C3C(C3(C)C)(C)C)c4ccccc42	8.8
156	O=C(C1C(C1(C)C)(C)C)c2cn(SC[C@@H]3CCOC3)c4ccccc42	8.8
157	O=S1(=O)CC[C@@H](Sn2cc(C(=O)C3C(C3(C)C)(C)C)c4ccccc42)C1	8.8
158	O=C(C1C(C1(C)C)(C)C)c2c3ccccc3n(CC[C@H](O)C(C)=C)c2	8.8
159	FC(F)(F)COCCn1cc(C(=O)C2C(C2(C)C)(C)C)c3ccccc31	8.8
160	O=C(C1C(C1(C)C)(C)C)c2c3ccccc3n(CSc4cccs4)c2	8.8
161	O=C(C1C(C1(C)C)(C)C)c2c3ccccc3n(CCCC4CC4)c2	8.8
162	O=C(C1C(C1(C)C)(C)C)c2c3ccccc3n(CC/C=C\CC)c2	8.8
163	O=C(C1C(C1(C)C)(C)C)c2c3ccccc3n(CCCc4c[nH]nc4C)c2	8.8
164	O=C(C1C(C1(C)C)(C)C)c2cn(SCCC(C)(C)C)c3ccccc32	8.8
165	O=C(C1C(C1(C)C)(C)C)c2c3ccccc3n(CC4=CCCC4)c2	8.8
166	O=C(C1C(C1(C)C)(C)C)c2c3ccccc3n(Cc4cc(cs4)C#N)c2	8.8
167	O=C(C1C(C1(C)C)(C)C)c2c3ccccc3n(CC[C@H](O)CC)c2	8.8
168	O=C(C1C(C1(C)C)(C)C)c2c3ccccc3n(CCc4cnccn4)c2	8.8
169	ClC(Cl)=CCn1cc(C(=O)C2C(C2(C)C)(C)C)c3ccccc31	8.8
170	O=C(C1C(C1(C)C)(C)C)c2c3ccccc3n(CCCSCC)c2	8.8
171	ClC1(Cl)[C@H](C1)COn2cc(C(=O)C3C(C3(C)C)(C)C)c4ccccc42	8.8
172	O=C(C1C(C1(C)C)(C)C)c2c3ccccc3n(CS[C@@H](CC)C)c2	8.8
173	FCCCCn1cc(C(=O)C2C(C2(C)C)(C)C)c3ccccc31	8.8
174	O=C(C1C(C1(C)C)(C)C)c2c3ccccc3n(CCC4CCOCC4)c2	8.8
175	SCC1(Cn2cc(C(=O)C3C(C3(C)C)(C)C)c4ccccc42)CC1	8.8
176	FC(F)(F)C[C@H](Cn1cc(C(=O)C2C(C2(C)C)(C)C)c3ccccc31)C	8.8
177	O=C(C1C(C1(C)C)(C)C)c2cn(OCCS(=O)(=O)C)c3ccccc32	8.8
178	O=C(C1C(C1(C)C)(C)C)c2c3ccccc3n(CCCC4CCCC4)c2	8.8
179	O=C(C1C(C1(C)C)(C)C)c2cn(c3ccccc32)CCSCCC	8.8
180	O=C(C1C(C1(C)C)(C)C)c2cn(c3ccccc32)/C=C\CC(C)C	8.8
181	O=C(C1C(C1(C)C)(C)C)c2c3ccccc3n(CC[C@@H](SC)C)c2	8.8
182	O=C(C1C(C1(C)C)(C)C)c2cn(NCC[C@@H](O)C)c3ccccc32	8.8
183	O=[S@](CC(C)C)Cn1cc(C(=O)C2C(C2(C)C)(C)C)c3ccccc31	8.8
184	O=C(C1C(C1(C)C)(C)C)c2c3ccccc3n(CCCC(C)=C)c2	8.8
185	O=C(C1C(C1(C)C)(C)C)c2c3ccccc3n(CCc4cnccc4)c2	8.8
186	O=C(C1C(C1(C)C)(C)C)c2cn(CCSCC)c3ccccc32	8.8
187	O=C(C1C(C1(C)C)(C)C)c2c3ccccc3n([C@@H](O)c4ccsc4)c2	8.8
188	O=C(C1C(C1(C)C)(C)C)c2c3ccccc3n(CSCC4CC4)c2	8.8
189	O=C(C1C(C1(C)C)(C)C)c2cn(SCCc3cnccc3)c4ccccc42	8.8

190	O=C(C1C(C1(C)C)(C)C)c2c3ccccc3n(CC[S@@](=O)C)c2	8.8
191	O=C(C1C(C1(C)C)(C)C)c2c3ccccc3n(C[n+]4ccccc4)c2	8.7
192	O=C(C1C(C1(C)C)(C)C)c2cn(SCCSCC)c3ccccc32	8.7
193	O=C(C1C(C1(C)C)(C)C)c2c3ccccc3n(C[C@H](SCC)C)c2	8.7
194	O=C(C1C(C1(C)C)(C)C)c2c3ccccc3n(CCCSC)c2	8.7
195	O=C(C1C(C1(C)C)(C)C)c2cn(NCCCSC)c3ccccc32	8.7
196	FC(F)COCCn1cc(C(=O)C2C(C2(C)C)(C)C)c3ccccc31	8.7
197	O=C(C1C(C1(C)C)(C)C)c2cn(SCC3CCOCC3)c4ccccc42	8.7
198	O=C(C1C(C1(C)C)(C)C)c2cn(NCCC(C)C)c3ccccc32	8.7
199	FC(F)(CCCn1cc(C(=O)C2C(C2(C)C)(C)C)c3ccccc31)C(F)(F)F	8.7
200	O=C(C1C(C1(C)C)(C)C)c2c3ccccc3n(CCSc4cnn[nH]4)c2	8.7
201	Fc1ccc([S@@](=O)n2cc(C(=O)C3C(C3(C)C)(C)C)c4ccccc42)cc1	8.7
202	O=C(C1C(C1(C)C)(C)C)c2cn(SCc3ccsc3)c4ccccc42	8.7
203	O=C(C1C(C1(C)C)(C)C)c2c3ccccc3n(Cc4ccc(s4)C)c2	8.7
204	ClC(Cl)CCn1cc(C(=O)C2C(C2(C)C)(C)C)c3ccccc31	8.7
205	BrC(CCn1cc(C(=O)C2C(C2(C)C)(C)C)c3ccccc31)=C	8.7
206	Fc1cncc(CCn2cc(C(=O)C3C(C3(C)C)(C)C)c4ccccc42)c1	8.7
207	FC(F)(F)CCOn1cc(C(=O)C2C(C2(C)C)(C)C)c3ccccc31	8.7
208	O=C(C1C(C1(C)C)(C)C)c2c3ccccc3n(C[C@@H]([N+])CCC)c2	8.7
209	FC(F)(F)[C@@H](O)CSn1cc(C(=O)C2C(C2(C)C)(C)C)c3ccccc31	8.7
210	O=C(C1C(C1(C)C)(C)C)c2c3ccccc3n(CCCCCC#C)c2	8.7
211	O=C(C1C(C1(C)C)(C)C)c2c3ccccc3n(COCC4CC4)c2	8.7
212	O=C(C1C(C1(C)C)(C)C)c2cn([C@H](CCSC)C)c3ccccc32	8.7
213	FC(F)(F)C[N+](CCn1cc(C(=O)C2C(C2(C)C)(C)C)c3ccccc31)C	8.7
214	FCCCNn1cc(C(=O)C2C(C2(C)C)(C)C)c3ccccc31	8.7
215	Clc1ccc(s1)CCn2cc(C(=O)C3C(C3(C)C)(C)C)c4ccccc42	8.7
216	O=C(C1C(C1(C)C)(C)C)c2c3ccccc3n(CC4=CCCOC4)c2	8.7
217	Clc1cc(F)c(Cn2cc(C(=O)C3C(C3(C)C)(C)C)c4ccccc42)cc1	8.7
218	O=C(C1C(C1(C)C)(C)C)c2c3ccccc3n(CCC=C(C)C)c2	8.7
219	O=C(C1C(C1(C)C)(C)C)c2cn(NC[C@H]3CC=CCC3)c4ccccc42	8.7
220	O=C(C1C(C1(C)C)(C)C)c2c3ccccc3n(CCCCC(C)C)c2	8.7
221	O=C(C1C(C1(C)C)(C)C)c2cn(c3ccccc32)CCSCC(C)C	8.7
222	O=C(C1C(C1(C)C)(C)C)c2c3ccccc3n(CCCC(C)(C)C#N)c2	8.7
223	O=C(C1C(C1(C)C)(C)C)c2c3ccccc3n(CCCCC(=O)C)c2	8.7
224	O=C(C1C(C1(C)C)(C)C)c2c3ccccc3n(CC[C@H]4CO4)c2	8.6
225	O=C(C1C(C1(C)C)(C)C)c2cn([S@](=O)CCCC)c3ccccc32	8.6
226	O=C(C1C(C1(C)C)(C)C)c2c3ccccc3n(C[S@](=O)CCC)c2	8.6
227	O=C(C1C(C1(C)C)(C)C)c2c3ccccc3n(CCC4CC4)c2	8.6
228	O=C(C1C(C1(C)C)(C)C)c2c3ccccc3n(CCCOC)c2	8.6
229	FC(F)(F)CSn1cc(C(=O)C2C(C2(C)C)(C)C)c3ccccc31	8.6
230	O=C(C1C(C1(C)C)(C)C)c2c3ccccc3n(CCc4cncs4)c2	8.6
231	O=C(C1C(C1(C)C)(C)C)c2c3ccccc3n(CCCOC(C)C)c2	8.6

232	O=C(C1C(C1(C)C)(C)C)c2cn(SCC3CCCC3)c4ccccc42	8.6
233	FCCOCCn1cc(C(=O)C2C(C2(C)C)(C)C)c3ccccc31	8.6
234	O=C(C1C(C1(C)C)(C)C)c2cn(NC[C@@H]3CCC=CO3)c4ccccc42	8.6
235	O=C(C1C(C1(C)C)(C)C)c2c3ccccc3n(COC4CC4)c2	8.6
236	O=C(C1C(C1(C)C)(C)C)c2cn(SCC3CCCCC3)c4ccccc42	8.6
237	O=C(C1C(C1(C)C)(C)C)c2cn(SC3COC3)c4ccccc42	8.6
238	O=C(C1C(C1(C)C)(C)C)c2c3ccccc3n(C[n+]4csc(c4)C)c2	8.6
239	FC(F)(Cn1cc(C(=O)C2C(C2(C)C)(C)C)c3ccccc31)C(F)F	8.6
240	FC([S@@](=O)n1cc(C(=O)C2C(C2(C)C)(C)C)c3ccccc31)(F)C(F)F	8.6
241	Clc1cc(CCn2cc(C(=O)C3C(C3(C)C)(C)C)c4ccccc42)cs1	8.6
242	Fc1cnc(Cn2cc(C(=O)C3C(C3(C)C)(C)C)c4ccccc42)cc1	8.6
243	O=C(C1C(C1(C)C)(C)C)c2cn(OCC3CCC3)c4ccccc42	8.6
244	Brc1ccc(o1)Cn2cc(C(=O)C3C(C3(C)C)(C)C)c4ccccc42	8.6
245	S=C(N)CCSn1cc(C(=O)C2C(C2(C)C)(C)C)c3ccccc31	8.6
246	O=S(=O)(CC(C)C)Cn1cc(C(=O)C2C(C2(C)C)(C)C)c3ccccc31	8.6
247	O=C(C1C(C1(C)C)(C)C)c2c3ccccc3n(CCc4ccoc4)c2	8.6
248	Cl[C@@H](F)C([S@@](=O)n1cc(C(=O)C2C(C2(C)C)(C)C)c3ccccc31)(F)F	8.6
249	O=C(C1C(C1(C)C)(C)C)c2c3ccccc3n(Cc4ccc(o4)C)c2	8.6
250	O=C(C1C(C1(C)C)(C)C)c2c3ccccc3n(CCC4CCCC4)c2	8.6
251	O=C(C1C(C1(C)C)(C)C)c2cn(SC[C@H](CC)C)c3ccccc32	8.5
252	O=C(C1C(C1(C)C)(C)C)c2cn(SSC3CCCCC3)c4ccccc42	8.5
253	O=C(C1C(C1(C)C)(C)C)c2c3ccccc3n(CSCC#N)c2	8.5
254	O=C(C1C(C1(C)C)(C)C)c2cn(c3ccccc32)/C=C\C=C/CC	8.5
255	O=C(C1C(C1(C)C)(C)C)c2cn(NCCSC)c3ccccc32	8.5
256	O=C(C1C(C1(C)C)(C)C)c2c3ccccc3n(COCCC(C)C)c2	8.5
257	O=C(C1C(C1(C)C)(C)C)c2cn(NCCC#N)c3ccccc32	8.5
258	O=C(C1C(C1(C)C)(C)C)c2c3ccccc3n(CCCCCCC)c2	8.5
259	O=C(C1C(C1(C)C)(C)C)c2c3ccccc3n(CCCC4CCCCC4)c2	8.5
260	O=C(C1C(C1(C)C)(C)C)c2cn(NCC3CCC3)c4ccccc42	8.5
261	O=C(C1C(C1(C)C)(C)C)c2c3ccccc3n(CO[C@H]4CCOC4)c2	8.5
262	O=C(C1C(C1(C)C)(C)C)c2c3ccccc3n(COCC(C)C)c2	8.5
263	O=C(C1C(C1(C)C)(C)C)c2c3ccccc3n(CC[N+]4CC=CCC4)c2	8.5
264	FC(F)(F)COn1cc(C(=O)C2C(C2(C)C)(C)C)c3ccccc31	8.5
265	FC(SCn1cc(C(=O)C2C(C2(C)C)(C)C)c3ccccc31)F	8.5
266	O=C(C1C(C1(C)C)(C)C)c2c3ccccc3n(CC[S@@](=O)CC)c2	8.5
267	O[C@H](CCCn1cc(C(=O)C2C(C2(C)C)(C)C)c3ccccc31)C	8.5
268	O=C(C1C(C1(C)C)(C)C)c2cn(NCCCCC)c3ccccc32	8.5
269	O=C(C1C(C1(C)C)(C)C)c2c3ccccc3n(C/C=C/C(C)C)c2	8.5
270	O=C(C1C(C1(C)C)(C)C)c2c3ccccc3n(c2)CC=C(C)C	8.5
271	Clc1ccc(s1)CSn2cc(C(=O)C3C(C3(C)C)(C)C)c4ccccc42	8.5
272	O=C(C1C(C1(C)C)(C)C)c2c3ccccc3n(CC[N+]4CCC[C@@H](C4)C)c2	8.5
273	O=C(C1C(C1(C)C)(C)C)c2c3ccccc3n(C[C@@H]4[C@H](C4)C)c2	8.5

274	O=C(C1C(C1(C)C)(C)C)c2c3ccccc3n(CSCCC)c2	8.5
275	FC(CCn1cc(C(=O)C2C(C2(C)C)(C)C)c3ccccc31)=C(F)F	8.5
276	Fc1cccc(SCn2cc(C(=O)C3C(C3(C)C)(C)C)c4ccccc42)c1	8.5
277	O=C(C1C(C1(C)C)(C)C)c2cn(SC3CCOCC3)c4ccccc42	8.5
278	O=C(C1C(C1(C)C)(C)C)c2c3ccccc3n(CCCC#CC)c2	8.5
279	Fc1cccc(CCn2cc(C(=O)C3C(C3(C)C)(C)C)c4ccccc42)c1	8.5
280	FC(F)(F)CCn1cc(C(=O)C2C(C2(C)C)(C)C)c3ccccc31	8.5
281	O=C(C1C(C1(C)C)(C)C)c2cn(SC[C@@H](O)CC)c3ccccc32	8.5
282	O=C(C1C(C1(C)C)(C)C)c2cn(SCCOC)c3ccccc32	8.5
283	O=C(C1C(C1(C)C)(C)C)c2c3ccccc3n(COC(C)C)c2	8.5
284	O=C(C1C(C1(C)C)(C)C)c2c3ccccc3n(CCCOCC=C)c2	8.5
285	Clc1ccc(s1)CNn2cc(C(=O)C3C(C3(C)C)(C)C)c4ccccc42	8.5
286	Cl/C(=C\Cn1cc(C(=O)C2C(C2(C)C)(C)C)c3ccccc31)C	8.5
287	FCCCOn1cc(C(=O)C2C(C2(C)C)(C)C)c3ccccc31	8.4
288	O=C(C1C(C1(C)C)(C)C)c2cn(Sc3ccncn3)c4ccccc42	8.4
289	O=C(C1C(C1(C)C)(C)C)c2c3ccccc3n(CSCCCCC)c2	8.4
290	O=C(C1C(C1(C)C)(C)C)c2c3ccccc3n(CC[C@H]([N+]C)C)c2	8.4
291	O=S(=O)(Cn1cc(C(=O)C2C(C2(C)C)(C)C)c3ccccc31)CC	8.4
292	O=C(C1C(C1(C)C)(C)C)c2cn(SC3CCCC3)c4ccccc42	8.4
293	O=C(C1C(C1(C)C)(C)C)c2c3ccccc3n(S(=O)(=O)NC4CCC4)c2	8.4
294	O=C(C1C(C1(C)C)(C)C)c2c3ccccc3n(C[S@@](=O)c4cccs4)c2	8.4
295	O=C(C1C(C1(C)C)(C)C)c2cn(c3ccccc32)C(CCCC)=C	8.4
296	O=C(C1C(C1(C)C)(C)C)c2c3ccccc3n(CC[C@@H](O)C)c2	8.4
297	O=C(C1C(C1(C)C)(C)C)c2c3ccccc3n(CSCC4CCCC4)c2	8.4
298	O=C(C1C(C1(C)C)(C)C)c2c3ccccc3n(C/C=C\CO)c2	8.4
299	O=C(C1C(C1(C)C)(C)C)c2c3ccccc3n(CCCO[N+]([O-])=O)c2	8.4
300	O=C(C1C(C1(C)C)(C)C)c2cn(Sc3ncns3)c4ccccc42	8.4
301	O=C(C1C(C1(C)C)(C)C)c2c3ccccc3n(CCOC=C)c2	8.4
302	O=C(C1C(C1(C)C)(C)C)c2cn(Sc3cnccc3)c4ccccc42	8.4
303	O=C(C1C(C1(C)C)(C)C)c2c3ccccc3n(CCCCC#C)c2	8.4
304	Cl/C=C/COn1cc(C(=O)C2C(C2(C)C)(C)C)c3ccccc31	8.4
305	O=C(C1C(C1(C)C)(C)C)c2c3ccccc3n(CCCOC=C)c2	8.4
306	O=C(C1C(C1(C)C)(C)C)c2c3ccccc3n(CSCC)c2	8.4
307	O=C(C1C(C1(C)C)(C)C)c2c3ccccc3n(CCSC(C)C)c2	8.4
308	FC(F)(F)CC[N+]Cn1cc(C(=O)C2C(C2(C)C)(C)C)c3ccccc31	8.4
309	FC(F)(F)CCCCn1cc(C(=O)C2C(C2(C)C)(C)C)c3ccccc31	8.4
310	O=C(C1C(C1(C)C)(C)C)c2cn(NC[C@@H]3CCOC3)c4ccccc42	8.4
311	O=C(C1C(C1(C)C)(C)C)c2cn(OCC3CC3)c4ccccc42	8.4
312	O=C(C1C(C1(C)C)(C)C)c2cn(c3ccccc32)CCSC	8.4
313	O=C(C1C(C1(C)C)(C)C)c2c3ccccc3n(COCC)c2	8.4
314	O=C(C1C(C1(C)C)(C)C)c2c3ccccc3n(C[C@@H]4C=CCC4)c2	8.4
315	FC(F)CNn1cc(C(=O)C2C(C2(C)C)(C)C)c3ccccc31	8.4

316	FC(F)C[N+](Cn1cc(C(=O)C2C(C2(C)C)(C)C)c3ccccc31)C	8.4
317	FCCCn1cc(C(=O)C2C(C2(C)C)(C)C)c3ccccc31	8.4
318	O=C(C1C(C1(C)C)(C)C)c2c3ccccc3n(C[N+][C@H]4[C@H](CCC4)C)c2	8.4
319	SCCCn1cc(C(=O)C2C(C2(C)C)(C)C)c3ccccc31	8.4
320	O=C(C1C(C1(C)C)(C)C)c2cn(NCCC)c3ccccc32	8.4
321	FC[C@H](O)Cn1cc(C(=O)C2C(C2(C)C)(C)C)c3ccccc31	8.4
322	O=C(C1C(C1(C)C)(C)C)c2c3ccccc3n(CC[N+]C(C)(C)C)c2	8.3
323	O=C(C1C(C1(C)C)(C)C)c2cn(NCCCC#N)c3ccccc32	8.3
324	O=C(C1C(C1(C)C)(C)C)c2cn(SCCCO)c3ccccc32	8.3
325	O=C(C1C(C1(C)C)(C)C)c2c3ccccc3n(C[N+]C4CC4)c2	8.3
326	O=C(C1C(C1(C)C)(C)C)c2c3ccccc3n(CCCSC(N)=[N+])c2	8.3
327	O=C(C1C(C1(C)C)(C)C)c2c3ccccc3n(Cc4ccc(o4)C#N)c2	8.3
328	O=C(C1C(C1(C)C)(C)C)c2cn(SCC3CC3)c4ccccc42	8.3
329	O=C(C1C(C1(C)C)(C)C)c2cn(SSSC)c3ccccc32	8.3
330	O=C(C1C(C1(C)C)(C)C)c2cn(OCCSCC)c3ccccc32	8.3
331	O=C(C1C(C1(C)C)(C)C)c2cn(OCSC)c3ccccc32	8.3
332	SCCCCn1cc(C(=O)C2C(C2(C)C)(C)C)c3ccccc31	8.3
333	O=C(C1C(C1(C)C)(C)C)c2cn(N/C=C/C)c3ccccc32	8.3
334	O=C(C1C(C1(C)C)(C)C)c2c3ccccc3n(CCCCOC)c2	8.3
335	O=C(C1C(C1(C)C)(C)C)c2c3ccccc3n(CCC(CC)CC)c2	8.3
336	O=C(C1C(C1(C)C)(C)C)c2c3ccccc3n(CSCCCC)c2	8.3
337	O=C(C1C(C1(C)C)(C)C)c2c3ccccc3n(C[N+]4CCCCC4)c2	8.3
338	O=C(C1C(C1(C)C)(C)C)c2c3ccccc3n(CC[C@H]4C=CCC4)c2	8.3
339	Cl/C=C/Cn1cc(C(=O)C2C(C2(C)C)(C)C)c3ccccc31	8.3
340	O=C(C1C(C1(C)C)(C)C)c2c3ccccc3n(c2)CC#CCCC	8.3
341	O=C(C1C(C1(C)C)(C)C)c2c3ccccc3n(C/C=C/CC)c2	8.3
342	O=C(C1C(C1(C)C)(C)C)c2cn(SS[C@@H](CC)C)c3ccccc32	8.3
343	O=C(C1C(C1(C)C)(C)C)c2c3ccccc3n(COCCC)c2	8.3
344	I/C=C/Cn1cc(C(=O)C2C(C2(C)C)(C)C)c3ccccc31	8.3
345	O=C(C1C(C1(C)C)(C)C)c2c3ccccc3n(CCC#C)c2	8.3
346	O=C(C1C(C1(C)C)(C)C)c2c3ccccc3n(CCCC=C)c2	8.3
347	FC(F)(F)CCSn1cc(C(=O)C2C(C2(C)C)(C)C)c3ccccc31	8.3
348	O=C(C1C(C1(C)C)(C)C)c2c3ccccc3n(C[N+][C@H]4CCSC4)c2	8.3
349	O=C(C1C(C1(C)C)(C)C)c2cn(NCC#CC)c3ccccc32	8.3
350	O=C(C1C(C1(C)C)(C)C)c2c3ccccc3n(CC[C@H]4CCCC[N+]4)c2	8.3
351	O=C(C1C(C1(C)C)(C)C)c2c3ccccc3n(Cc4ccc(o4)CO)c2	8.3
352	O=C(C1C(C1(C)C)(C)C)c2c3ccccc3n(c2)CC#CCC#C	8.3
353	Cl[C@@H](F)C(Sn1cc(C(=O)C2C(C2(C)C)(C)C)c3ccccc31)(F)F	8.3
354	O=C(C1C(C1(C)C)(C)C)c2c3ccccc3n(C[C@H](CC(C)C)C#N)c2	8.3
355	SC(=S)NCn1cc(C(=O)C2C(C2(C)C)(C)C)c3ccccc31	8.3
356	O=C(C1C(C1(C)C)(C)C)c2c3ccccc3n(CCC[C@@H]([N+]C)C)c2	8.3
357	FC(Sn1cc(C(=O)C2C(C2(C)C)(C)C)c3ccccc31)(F)F	8.2

358	O=C(C1C(C1(C)C)(C)C)c2c3ccccc3n(C[C@H]4CCCC[N+]4)c2	8.2
359	O=C(C1C(C1(C)C)(C)C)c2c3ccccc3n(CCCC#N)c2	8.2
360	O=C(C1C(C1(C)C)(C)C)c2c3ccccc3n(CCC4(N=N4)C)c2	8.2
361	O=C(C1C(C1(C)C)(C)C)c2c3ccccc3n(c2)CC#CC	8.2
362	O=C(C1C(C1(C)C)(C)C)c2c3ccccc3n(CCCC#C)c2	8.2
363	FC(F)(F)Cn1cc(C(=O)C2C(C2(C)C)(C)C)c3ccccc31	8.2
364	O=C(C1C(C1(C)C)(C)C)c2c3ccccc3n(c2)CC#CCC	8.2
365	O=C(C1C(C1(C)C)(C)C)c2cn(OC[C@H]3CS3)c4ccccc42	8.2
366	SCCSn1cc(C(=O)C2C(C2(C)C)(C)C)c3ccccc31	8.2
367	ClC(Cl)=CCOn1cc(C(=O)C2C(C2(C)C)(C)C)c3ccccc31	8.2
368	O=C(C1C(C1(C)C)(C)C)c2cn(SSCC)c3ccccc32	8.2
369	O=C(C1C(C1(C)C)(C)C)c2cn(NCc3ccco3)c4ccccc42	8.2
370	Cl/C(Cn1cc(C(=O)C2C(C2(C)C)(C)C)c3ccccc31)=C\Cl	8.2
371	O=C(C1C(C1(C)C)(C)C)c2c3ccccc3n(CC/C=C/C)c2	8.2
372	SCCCOn1cc(C(=O)C2C(C2(C)C)(C)C)c3ccccc31	8.2
373	O=C(C1C(C1(C)C)(C)C)c2cn(SCCCC)c3ccccc32	8.2
374	O=C(C1C(C1(C)C)(C)C)c2c3ccccc3n(CCCCCC)c2	8.2
375	O=C(C1C(C1(C)C)(C)C)c2c3ccccc3n(C[N+][C@@H]4C(C4)(C)C)c2	8.2
376	O=C(C1C(C1(C)C)(C)C)c2c3ccccc3n(COC4CCCC4)c2	8.2
377	FC(F)CSn1cc(C(=O)C2C(C2(C)C)(C)C)c3ccccc31	8.2
378	O=C(C1C(C1(C)C)(C)C)c2c3ccccc3n(C[N+]C4CCCC4)c2	8.2
379	FC(F)COn1cc(C(=O)C2C(C2(C)C)(C)C)c3ccccc31	8.2
380	FCCCSn1cc(C(=O)C2C(C2(C)C)(C)C)c3ccccc31	8.2
381	F[C@H]1CC[C@@H]([N+]Cn2cc(C(=O)C3C(C3(C)C)(C)C)c4ccccc42)C1	8.2
382	O=C(C1C(C1(C)C)(C)C)c2c3ccccc3n(C[N+][C@H]4[C@@H](C4)C)c2	8.2
383	O=C(C1C(C1(C)C)(C)C)c2c3ccccc3n(CCCO)c2	8.1
384	O=C(C1C(C1(C)C)(C)C)c2c3ccccc3n(CC[N+]4[C@@H](C4)C)c2	8.1
385	O=C(C1C(C1(C)C)(C)C)c2cn(SC[C@H]3CCC[N+]3)c4ccccc42	8.1
386	FC(F)Cn1cc(C(=O)C2C(C2(C)C)(C)C)c3ccccc31	8.1
387	O=C(C1C(C1(C)C)(C)C)c2c3ccccc3n(C[N+]C4CCC4)c2	8.1
388	O=C(C1C(C1(C)C)(C)C)c2c3ccccc3n(C[C@@H]4C[C@@H](OC)C[N+]4)c2	8.1
389	O=C(C1C(C1(C)C)(C)C)c2c3ccccc3n(C/C=C/C)c2	8.1
390	O=C(C1C(C1(C)C)(C)C)c2cn(c3ccccc32)/C=C\CCCC	8.1
391	ClC(Cl)Cn1cc(C(=O)C2C(C2(C)C)(C)C)c3ccccc31	8.1
392	O=C(C1C(C1(C)C)(C)C)c2cn(SCCC#C)c3ccccc32	8.1
393	O=C(C1C(C1(C)C)(C)C)c2c3ccccc3n(C[S+]4CCCC4)c2	8.1
394	O=C(C1C(C1(C)C)(C)C)c2c3ccccc3n(C[n+]4cscc4)c2	8.1
395	O=C(C1C(C1(C)C)(C)C)c2c3ccccc3n(C[N+]Cc4ccc[nH]4)c2	8.1
396	Br/C=C/Cn1cc(C(=O)C2C(C2(C)C)(C)C)c3ccccc31	8.1
397	O=C(C1C(C1(C)C)(C)C)c2c3ccccc3n(C[C@@H]([N+])CC(C)C)c2	8.1
398	O=C(C1C(C1(C)C)(C)C)c2c3ccccc3n(CCCCCCC#N)c2	8.1
399	O=C(C1C(C1(C)C)(C)C)c2c3ccccc3n(C[S@@](=O)CC)c2	8.1

400	O=C(C1C(C1(C)C)(C)C)c2c3ccccc3n(C[S@@](=O)C4CCCC4)c2	8.1
401	O=C(C1C(C1(C)C)(C)C)c2c3ccccc3n(C[N+]CC=4[N-]N=NN4)c2	8.1
402	O=C(C1C(C1(C)C)(C)C)c2c3ccccc3n(CC[N+]4CC[C@H](C4)C)c2	8.1
403	O=C(C1C(C1(C)C)(C)C)c2cn(OC[C@@H]3[C@H](C3)C)c4ccccc42	8.1
404	O=C(C1C(C1(C)C)(C)C)c2c3ccccc3n(C/C=C/C#C)c2	8
405	SC[C@@H]([N+])CCn1cc(C(=O)C2C(C2(C)C)(C)C)c3ccccc31	8
406	O=C(C1C(C1(C)C)(C)C)c2c3ccccc3n(CCCCC)c2	8
407	O=C(C1C(C1(C)C)(C)C)c2cn(NCC3CC3)c4ccccc42	8
408	O=C(C1C(C1(C)C)(C)C)c2c3ccccc3n(CC4CC4)c2	8
409	O=C(C1C(C1(C)C)(C)C)c2c3ccccc3n(S(=O)(=O)NCC(C)(C)C)c2	8
410	O=C(C1C(C1(C)C)(C)C)c2cn(SSC(C)C)c3ccccc32	8
411	O=C(C1C(C1(C)C)(C)C)c2c3ccccc3n(C[N+][C@@H](CC)C)c2	8
412	O=C(C1C(C1(C)C)(C)C)c2c3ccccc3n(CC[N+]C(C)C)c2	8
413	O=C(C1C(C1(C)C)(C)C)c2cn(OCCSC)c3ccccc32	8
414	O=C(C1C(C1(C)C)(C)C)c2c3ccccc3n(C[N+]CC(C)=C)c2	8
415	O=C(C1C(C1(C)C)(C)C)c2c3ccccc3n(CC[C@@H]4CC[N+]C4)c2	8
416	O=C(C1C(C1(C)C)(C)C)c2c3ccccc3n(CCC[S+](C)C)c2	8
417	FC1(F)CC[N+](C1)CCn2cc(C(=O)C3C(C3(C)C)(C)C)c4ccccc42	8
418	O=C(C1C(C1(C)C)(C)C)c2cn(c3ccccc32)CCSCC#C	8
419	O=C(C1C(C1(C)C)(C)C)c2c3ccccc3n(C[N+]CC4CCC4)c2	8
420	O=C(C1C(C1(C)C)(C)C)c2c3ccccc3n(CNC=4CCCCC[N+]4)c2	8
421	O=C(C1C(C1(C)C)(C)C)c2c3ccccc3n(CCC=C)c2	8
422	O=C(C1C(C1(C)C)(C)C)c2c3ccccc3n(COCC#C)c2	8
423	O=C(C1C(C1(C)C)(C)C)c2cn(SCC#CCO)c3ccccc32	7.9
424	O=C(C1C(C1(C)C)(C)C)c2c3ccccc3n(C[N+]C(C)C)c2	7.9
425	ClC(C[N+]Cn1cc(C(=O)C2C(C2(C)C)(C)C)c3ccccc31)=C	7.9
426	O=C(C1C(C1(C)C)(C)C)c2c3ccccc3n(CSCC=C)c2	7.9
427	O=C(C1C(C1(C)C)(C)C)c2cn([C@@H]([N+])Cc3cnccc3)c4ccccc42	7.9
428	O=C(C1C(C1(C)C)(C)C)c2c3ccccc3n(COC/C=C/C)c2	7.9
429	FCCSn1cc(C(=O)C2C(C2(C)C)(C)C)c3ccccc31	7.9
430	O=C(C1C(C1(C)C)(C)C)c2c3ccccc3n(CCCC)c2	7.9
431	O=C(C1C(C1(C)C)(C)C)c2cn(SC/C=C/C)c3ccccc32	7.9
432	O=C(C1C(C1(C)C)(C)C)c2cn(NCC)c3ccccc32	7.9
433	FC(Sn1cc(C(=O)C2C(C2(C)C)(C)C)c3ccccc31)F	7.9
434	S/C(NCCn1cc(C(=O)C2C(C2(C)C)(C)C)c3ccccc31)=[N+]\C	7.9
435	O=C(C1C(C1(C)C)(C)C)c2cn([C@@H]([N+])CC3CC3)c4ccccc42	7.9
436	FCCOn1cc(C(=O)C2C(C2(C)C)(C)C)c3ccccc31	7.9
437	SCCn1cc(C(=O)C2C(C2(C)C)(C)C)c3ccccc31	7.9
438	O=C(C1C(C1(C)C)(C)C)c2c3ccccc3n(CC[N+][C@H](C4CC4)C)c2	7.9
439	O=C(C1C(C1(C)C)(C)C)c2c3ccccc3n(C[N+]CC(C)C)c2	7.9
440	IC#CCn1cc(C(=O)C2C(C2(C)C)(C)C)c3ccccc31	7.9
441	O=C(C1C(C1(C)C)(C)C)c2cn(SCC=C(C)C)c3ccccc32	7.9

442	O=C(C1C(C1(C)C)(C)C)c2c3ccccc3n(C[C@H]([N+]CC#C)C)c2	7.8
443	O=C(C1C(C1(C)C)(C)C)c2c3ccccc3n(C[N+]4CCCC4)c2	7.8
444	O=C(C1C(C1(C)C)(C)C)c2c3ccccc3n(CC[N+]4CCCC4)c2	7.8
445	O=C(C1C(C1(C)C)(C)C)c2c3ccccc3n(CCC)c2	7.8
446	SCCOn1cc(C(=O)C2C(C2(C)C)(C)C)c3ccccc31	7.8
447	FC(F)CNS(=O)(=O)n1cc(C(=O)C2C(C2(C)C)(C)C)c3ccccc31	7.8
448	O=C(C1C(C1(C)C)(C)C)c2c3ccccc3n(C[C@H]4CC[N+]4)c2	7.8
449	BrC(C[N+]Cn1cc(C(=O)C2C(C2(C)C)(C)C)c3ccccc31)=C	7.8
450	FCCOCn1cc(C(=O)C2C(C2(C)C)(C)C)c3ccccc31	7.8
451	O=C(C1C(C1(C)C)(C)C)c2c3ccccc3n(C[N+]Cc4ccoc4)c2	7.8
452	O=C(C1C(C1(C)C)(C)C)c2c3ccccc3n([C@H](O)CC[N+](C)C)c2	7.8
453	O=C(C1C(C1(C)C)(C)C)c2c3ccccc3n(C[C@H]4CS4)c2	7.8
454	O=C(C1C(C1(C)C)(C)C)c2cn(OCCCC)c3ccccc32	7.8
455	O=C(C1C(C1(C)C)(C)C)c2c3ccccc3n(C[N+]CCC)c2	7.8
456	O=C(C1C(C1(C)C)(C)C)c2cn([C@@H]([N+])CC3CCCC3)c4ccccc42	7.8
457	FC(Cn1cc(C(=O)C2C(C2(C)C)(C)C)c3ccccc31)=C	7.8
458	O=C(C1C(C1(C)C)(C)C)c2cn(NCC#C)c3ccccc32	7.8
459	FC(F)(F)C[N+]Cn1cc(C(=O)C2C(C2(C)C)(C)C)c3ccccc31	7.8
460	O=C(C1C(C1(C)C)(C)C)c2c3ccccc3n(C[N+]CCSC)c2	7.7
461	O=C(C1C(C1(C)C)(C)C)c2c3ccccc3n([C@@H]([N+])CCC(C)C)c2	7.7
462	O=C(C1C(C1(C)C)(C)C)c2c3ccccc3n(CCC#CC)c2	7.7
463	O=C(C1C(C1(C)C)(C)C)c2cn(SCC#CC)c3ccccc32	7.7
464	FC(F)C[N+]Cn1cc(C(=O)C2C(C2(C)C)(C)C)c3ccccc31	7.7
465	O=C(C1C(C1(C)C)(C)C)c2c3ccccc3n(CC[N+]4CCC4)c2	7.7
466	O=C(C1C(C1(C)C)(C)C)c2c3ccccc3n(CC[N+](CC)C)c2	7.7
467	O=C(C1C(C1(C)C)(C)C)c2cn(SCC#C)c3ccccc32	7.7
468	O=C(C1C(C1(C)C)(C)C)c2c3ccccc3n(CSC)c2	7.7
469	O=C(C1C(C1(C)C)(C)C)c2cn([C@@H]([N+])CC(C)C)c3ccccc32	7.7
470	O=C(C1C(C1(C)C)(C)C)c2c3ccccc3n(C[C@@H]4CCC[N+]4)c2	7.6
471	O=C(C1C(C1(C)C)(C)C)c2c3ccccc3n(C[N+]CC#C)c2	7.6
472	O=C(C1C(C1(C)C)(C)C)c2c3ccccc3n(CC[N+]4CC4)c2	7.6
473	O=C(C1C(C1(C)C)(C)C)c2cn(c3ccccc32)[C@@H](O)CC#C	7.6
474	FCC[N+]Cn1cc(C(=O)C2C(C2(C)C)(C)C)c3ccccc31	7.6
475	O=C(C1C(C1(C)C)(C)C)c2cn(SCSC)c3ccccc32	7.6
476	S/C(=[N+]/Cn1cc(C(=O)C2C(C2(C)C)(C)C)c3ccccc31)N	7.5
477	O=C(C1C(C1(C)C)(C)C)c2cn([C@@H]([N+])CCCCC)c3ccccc32	7.5
478	O=C(C1C(C1(C)C)(C)C)c2c3ccccc3n(COC)c2	7.5
479	FC(F)(F)C[N+]CCn1cc(C(=O)C2C(C2(C)C)(C)C)c3ccccc31	7.5
480	O=C(C1C(C1(C)C)(C)C)c2c3ccccc3n(CC[C@H]([N+])C4CC4)c2	7.5
481	SCn1cc(C(=O)C2C(C2(C)C)(C)C)c3ccccc31	7.4
482	O=C(C1C(C1(C)C)(C)C)c2c3ccccc3n(C[N+]CC#CC)c2	7.4
483	O=C(C1C(C1(C)C)(C)C)c2c3ccccc3n(CC[C@H]4CCC[N+]4)c2	7.4

484	O=C(C1C(C1(C)C)(C)C)c2cn([C@@H]([N+])CCCC)c3ccccc32	7.4
485	FCCn1cc(C(=O)C2C(C2(C)C)(C)C)c3ccccc31	7.4
486	O=C(C1C(C1(C)C)(C)C)c2c3ccccc3n(c2)CC#C	7.4
487	SCC[N+]Cn1cc(C(=O)C2C(C2(C)C)(C)C)c3ccccc31	7.3
488	O=C(C1C(C1(C)C)(C)C)c2c3ccccc3n(C[N+]CC)c2	7.3
489	O=C(C1C(C1(C)C)(C)C)c2c3ccccc3n(CC[N+]C)c2	7.3
490	O=C(C1C(C1(C)C)(C)C)c2cn([C@@H]([N+])CSC)c3ccccc32	7.2
491	O=C(C1C(C1(C)C)(C)C)c2c3ccccc3n(CC[N+]CC)c2	7.2
492	O=C(C1C(C1(C)C)(C)C)c2cn(SC[C@H]([N+])CC)c3ccccc32	7.2
493	O=C(C1C(C1(C)C)(C)C)c2c3ccccc3n(C[N+]4CC4)c2	7.1
494	FCSn1cc(C(=O)C2C(C2(C)C)(C)C)c3ccccc31	7
495	O=C(C1C(C1(C)C)(C)C)c2cn([C@@H]([N+])CC#C)c3ccccc32	7
496	O=C(C1C(C1(C)C)(C)C)c2cn(SSCC[N+])c3ccccc32	6.8
497	O=C(C1C(C1(C)C)(C)C)c2cn([C@@H]([N+])CCC)c3ccccc32	6.8
498	O=C(C1C(C1(C)C)(C)C)c2c3ccccc3n([C@@H]([N+])C4CC4)c2	6.7
499	O=C(C1C(C1(C)C)(C)C)c2c3ccccc3n(C[N+]C)c2	6.5
500	O=C(C1C(C1(C)C)(C)C)c2c3ccccc3n([C@@H]([N+])CC)c2	6.4

Table S15. List, SMILE and predicted pK_i values for Series 5 in CB_2 receptor.

N°	SMILES	Pred pK_i
1	Clc1ccn2c(c(C(=O)C3C(C3(C)C)(C)C)cc2CCCC(F)(F)F)c1	9.9
2	FC(F)(F)CCCc1cc(C(=O)C2C(C2(C)C)(C)C)c3c(CCCO3)c1	9.8
3	FC(F)(F)CCCc1cc(C(=O)C2C(C2(C)C)(C)C)c3cc(ccn13)C	9.8
4	FC(F)(F)CCCn1cc(n2n1[nH]ccs2)C(=O)C3C(C3(C)C)(C)C	9.8
5	FC(F)(F)CCCn1c2CCCC(=O)c2c(n1)C(=O)C3C(C3(C)C)(C)C	9.8
6	FC(F)(F)CCCn1c2C=CSC(=O)c2c(n1)C(=O)C3C(C3(C)C)(C)C	9.8
7	FC(F)(F)CCCc1cc(C(=O)C2C(C2(C)C)(C)C)cc(c1N)C	9.7
8	Brc1c(C(=O)C2C(C2(C)C)(C)C)cc(n1C)CCCC(F)(F)F	9.7
9	FC(F)(F)CCCc1cc(C(=O)C2C(C2(C)C)(C)C)c3ccccn31	9.7
10	FC(F)(F)CCCc1cc(C(=O)C2C(C2(C)C)(C)C)c3cccc(n31)C	9.7
11	FC(F)(F)CCCc1c(N)cc(F)c(C(=O)C2C(C2(C)C)(C)C)c1	9.7
12	FC(F)(F)CCCc1cc(C(=O)C2C(C2(C)C)(C)C)c3C(SC=Cn31)=O	9.7
13	FC(F)(F)CCCc1cc(C(=O)C2C(C2(C)C)(C)C)c3C(=O)NC=Cn31	9.7
14	FC(F)(F)CCCc1nc(C(=O)C2C(C2(C)C)(C)C)c3n1ccs3	9.6
15	FC(F)(F)CCCc1cc(c2CCCn12)C(=O)C3C(C3(C)C)(C)C	9.6
16	FC(F)(F)CCCc1c(sc(C(=O)C2C(C2(C)C)(C)C)c1)N	9.6
17	FC(F)(F)CCCc1c(c(C(=O)C2C(C2(C)C)(C)C)c3ccccn13)C	9.6
18	FC(F)(F)CCCn1cc(C(=O)C2C(C2(C)C)(C)C)c3c(OC)cccc31	9.6
19	FC(F)(F)CCCn1c2c(c(n1)C(=O)C3C(C3(C)C)(C)C)C(=O)C=CS2	9.6
20	FC(F)(F)CCCc1cc(C(=O)C2C(C2(C)C)(C)C)c3ccsn31	9.6
21	FC(F)(F)CCCc1c2C=COC(=O)n2c(C(=O)C3C(C3(C)C)(C)C)c1	9.6

22	FC(F)(F)CCCn1c2cc(O)ccc2c(n1)C(=O)C3C(C3(C)C)(C)C	9.6
23	FC(F)(F)CCCc1cc(n2cc[nH]c12)C(=O)C3C(C3(C)C)(C)C	9.6
24	FC(F)(F)CCCc1c2C=CSC(=O)c2n(n1)C(=O)C3C(C3(C)C)(C)C	9.6
25	FC(F)(F)CCCc1cc2cccnc2c(C(=O)C3C(C3(C)C)(C)C)c1	9.5
26	FC(F)(F)CCCc1cc2c(c(C(=O)C3C(C3(C)C)(C)C)c1)cc[nH]2	9.5
27	FC(F)(F)CCCc1cc(C(=O)C2C(C2(C)C)(C)C)c(n1C)C	9.5
28	FC(F)(F)CCCc1c(N)c(cc(C(=O)C2C(C2(C)C)(C)C)c1)CC	9.5
29	Brc1cc(C(=O)C2C(C2(C)C)(C)C)cc(CCCC(F)(F)F)c1N	9.5
30	FC(F)(F)CCCc1cc(C(=O)C2C(C2(C)C)(C)C)c3C(OC=Cn31)=O	9.5
31	Brc1c(nn(CCCC(F)(F)F)c1C)C(=O)C2C(C2(C)C)(C)C	9.5
32	FC(F)(F)CCCc1cc2c(nc(O)cc2C)c(C(=O)C3C(C3(C)C)(C)C)c1	9.5
33	FC(F)(F)CCCc1cc(C(=O)C2C(C2(C)C)(C)C)cc3CCNc31	9.5
34	FC(F)(F)CCCc1cc(F)c(c(C(=O)C2C(C2(C)C)(C)C)c1)C(=O)C	9.5
35	FC(F)(F)CCCc1nc(C(=O)C2C(C2(C)C)(C)C)c3ccncn31	9.5
36	FC(F)(F)CCCc1cc(C(=O)C2C(C2(C)C)(C)C)cc3c[nH]cc31	9.5
37	Fc1c(cc(CCCC(F)(F)F)cc1C(=O)C2C(C2(C)C)(C)C)CO	9.5
38	Clc1c(C(=O)C2C(C2(C)C)(C)C)cc(n1C)CCCC(F)(F)F	9.5
39	FC(F)(F)CCCn1cc(C(=O)C2C(C2(C)C)(C)C)c3cc[nH]c31	9.5
40	FC(F)(F)CCCn1c2CCCc2c(n1)C(=O)C3C(C3(C)C)(C)C	9.5
41	FC(F)(F)CCCOc1ccc(F)cc1CC(=O)C2C(C2(C)C)(C)C	9.5
42	FC(F)(F)CCCc1cc(C(=O)C2C(C2(C)C)(C)C)cc3c1[nH]cc3CC	9.5
43	FC(F)(F)CCCc1c2C=CSC(=O)n2c(C(=O)C3C(C3(C)C)(C)C)c1	9.5
44	FC(F)(F)CCCn1c2C=CSC(=O)c2c(C(=O)C3C(C3(C)C)(C)C)c1	9.4
45	FC(F)(F)CCCc1cc(C(=O)C2C(C2(C)C)(C)C)c3n1ccs3	9.4
46	Clc1cccc2c1c(nn2CCCC(F)(F)F)C(=O)C3C(C3(C)C)(C)C	9.4
47	FC(F)(F)CCCc1c2C=CSC(=O)c2n(C(=O)C3C(C3(C)C)(C)C)c1	9.4
48	FC(F)(F)CCCc1cc(C(=O)C2C(C2(C)C)(C)C)c3C(SC=Nn31)=O	9.4
49	FC(F)(F)CCCc1c2C=CSC(=O)c2c(s1)C(=O)C3C(C3(C)C)(C)C	9.4
50	FC(F)(F)CCCc1cc(C(=O)C2C(C2(C)C)(C)C)cn1C	9.4
51	FC(F)(F)CCCc1c2c(c(s1)C(=O)C3C(C3(C)C)(C)C)cns2	9.4
52	FC(F)(F)CCCc1cn2cccc2c(C(=O)C3C(C3(C)C)(C)C)c1	9.4
53	FC(F)(F)CCCc1cc(c(c(C(=O)C2C(C2(C)C)(C)C)c1)C#N)C	9.4
54	FC(F)(F)CCCn1c2c(c(C(=O)C3C(C3(C)C)(C)C)c1)C(=O)C=CS2	9.4
55	FC(F)(F)CCCn1cc(C(=O)C2C(C2(C)C)(C)C)c3c1csn3	9.4
56	FC(F)(F)CCCc1c2CCCC(=O)c2c(s1)C(=O)C3C(C3(C)C)(C)C	9.4
57	FC(F)(F)CCCn1c2CCCCc2c(n1)C(=O)C3C(C3(C)C)(C)C	9.4
58	FC(F)(F)CCCn1c2ccsc2c(n1)C(=O)C3C(C3(C)C)(C)C	9.4
59	FC(F)(F)CCCc1c2ccnnn2c(C(=O)C3C(C3(C)C)(C)C)c1	9.4
60	FC(F)(F)CCCc1cc(C(=O)C2C(C2(C)C)(C)C)c(n1CC=C)C	9.4
61	FC(F)(F)CCCc1c(N)ccc(C(=O)C2C(C2(C)C)(C)C)c1	9.4
62	FC(F)(F)CCCc1cc(C(=O)C2C(C2(C)C)(C)C)cc3c1[nH]cc3C	9.4
63	FC(F)(F)CCCc1cc(C(=O)C2C(C2(C)C)(C)C)cc3c1[nH]cn3	9.4

64	FC(F)(F)CCCc1cc(OCC)c(F)c(C(=O)C2C(C2(C)C)(C)C)c1	9.4
65	FC(F)(F)CCCn1c2c(c(C(=O)C3C(C3(C)C)(C)C)c1)C(SC=N2)=O	9.4
66	FC(F)(F)CCCc1cc(C(=O)C2C(C2(C)C)(C)C)cc3cc[nH]c31	9.4
67	FC(F)(F)CCCc1cc2c(nccc2C)c(C(=O)C3C(C3(C)C)(C)C)c1	9.4
68	FC(F)(F)CCCN1c2ccccc2N(C(=O)C3C(C3(C)C)(C)C)C1=N	9.4
69	FC(F)(F)CCCc1c2c(c([nH]1)C(=O)C3C(C3(C)C)(C)C)cc[nH]2	9.4
70	FC(F)(F)CCCc1c2C=CSC(=O)c2c(o1)C(=O)C3C(C3(C)C)(C)C	9.4
71	FC(F)(F)CCCn1cc(C(=O)C2C(C2(C)C)(C)C)c3cc(O)ccc31	9.4
72	FC(F)(F)CCCc1cc2csnc2c(C(=O)C3C(C3(C)C)(C)C)c1	9.4
73	FC(F)(F)CCCc1c(N)c(C(=O)C2C(C2(C)C)(C)C)c3ccccn31	9.3
74	Brc1cc(CCCC(F)(F)F)cc(C(=O)C2C(C2(C)C)(C)C)c1OC	9.3
75	Clc1c(nn(CCCC(F)(F)F)c1C)C(=O)C2C(C2(C)C)(C)C	9.3
76	FC(F)(F)CCCc1cc(C(=O)C2C(C2(C)C)(C)C)c(n1CC)C	9.3
77	FC(F)(F)CCCn1c2cc(ccc2c(n1)C(=O)C3C(C3(C)C)(C)C)C	9.3
78	FC(F)(F)CCCc1c2n(c(C(=O)C3C(C3(C)C)(C)C)c1)C(=O)C=CS2	9.3
79	Clc1c(C(=O)C2C(C2(C)C)(C)C)c3ccccc3n1CCCC(F)(F)F	9.3
80	FC(F)(F)CCCn1c2ccc(F)cc2c(n1)C(=O)C3C(C3(C)C)(C)C	9.3
81	FC(F)(F)CCCc1c2N=CSC(=O)n2c(C(=O)C3C(C3(C)C)(C)C)c1	9.3
82	FC(F)(F)CCCn1c2CCOCc2c(n1)C(=O)C3C(C3(C)C)(C)C	9.3
83	FC(F)(F)CCCc1c2C=CNC(=O)c2c(o1)C(=O)C3C(C3(C)C)(C)C	9.3
84	FC(F)(F)CCCn1cc(C(=O)C2C(C2(C)C)(C)C)c3CCCc31	9.3
85	FC(F)(F)CCCc1cc(C(=O)C2C(C2(C)C)(C)C)c3ccncn31	9.3
86	FC(F)(F)CCCc1c2ccccc2c(s1)C(=O)C3C(C3(C)C)(C)C	9.3
87	FC(F)(F)CCCc1c2ccsc2c(o1)C(=O)C3C(C3(C)C)(C)C	9.3
88	Clc1cc(N)c(CCCC(F)(F)F)cc1C(=O)C2C(C2(C)C)(C)C	9.3
89	Clc1cc(C(=O)C2C(C2(C)C)(C)C)cc(CCCC(F)(F)F)c1N	9.3
90	FC(F)(F)CCCn1c2ccc(cc2c(n1)C(=O)C3C(C3(C)C)(C)C)C	9.3
91	FC(F)(F)CCCn1cc(c2C(=O)CC(Cc21)(C)C)C(=O)C3C(C3(C)C)(C)C	9.3
92	FC(F)(F)CCCc1cc(C(=O)C2C(C2(C)C)(C)C)c3ccn(c3c1)C	9.3
93	FC(F)(F)CCCn1cc(C(=O)C2C(C2(C)C)(C)C)c3c[nH]cc31	9.3
94	FC(F)(F)CCCc1c(c(c([nH]1)C(=O)C2C(C2(C)C)(C)C)C(=O)C)C	9.3
95	FC(F)(F)CCCc1nc(C(=O)C2C(C2(C)C)(C)C)cc3cccn31	9.3
96	FC(F)(F)CCCn1cc(C(=O)C2C(C2(C)C)(C)C)c3c1cc(cn3)C	9.3
97	Brc1c(sc(CCCC(F)(F)F)c1C)C(=O)C2C(C2(C)C)(C)C	9.3
98	FC(F)(F)CCCc1c2c(c(o1)C(=O)C3C(C3(C)C)(C)C)cns2	9.3
99	Clc1ccc2c(c(nn2CCCC(F)(F)F)C(=O)C3C(C3(C)C)(C)C)c1	9.3
100	FC(F)(F)CCCc1cc(N)c(F)c(C(=O)C2C(C2(C)C)(C)C)c1	9.3
101	FC(F)(F)CCCn1c2ccccc2c(n1)C(=O)C3C(C3(C)C)(C)C	9.3
102	FC(F)(F)CCCc1cc(C(=O)C2C(C2(C)C)(C)C)cn1CC3CC3	9.3
103	FC(F)(F)CCCc1cc(C(=O)C2C(C2(C)C)(C)C)cc3c1cco3	9.3
104	FC(F)(F)CCCN1c2ccccc2N(S1(=O)=O)C(=O)C3C(C3(C)C)(C)C	9.3
105	FC(F)(F)CCCc1cc(C(=O)C2C(C2(C)C)(C)C)cc3CCCNc31	9.3

106	FC(F)(F)CCCc1c(O)c(O)c(O)c(C(=O)C2C(C2(C)C)(C)C)c1	9.3
107	FC(F)(F)CCCn1cc(C(=O)C2C(C2(C)C)(C)C)c3c1cccn3	9.3
108	FC(F)(F)CCCn1cc(C(=O)C2C(C2(C)C)(C)C)c3ccc(O)cc31	9.2
109	FC(F)(F)CCCc1cc(C(=O)C2C(C2(C)C)(C)C)c(n1CCC)C	9.2
110	FC(F)(F)CCCc1c2C=COC(=O)c2c([nH]1)C(=O)C3C(C3(C)C)(C)C	9.2
111	FC(F)(F)CCCc1cc2c(ncs2)c(C(=O)C3C(C3(C)C)(C)C)c1	9.2
112	FC(F)(F)CCCn1cc2-n(sccs2)c(C(=O)C3C(C3(C)C)(C)C)c1	9.2
113	FC(F)(F)CCCc1cc2COC(=O)c2c(C(=O)C3C(C3(C)C)(C)C)c1	9.2
114	FC(F)(F)CCCc1cc2cncnc2c(C(=O)C3C(C3(C)C)(C)C)c1	9.2
115	FC(F)(F)CCCn1cc(C(=O)C2C(C2(C)C)(C)C)c3c1ccc(C(C)(C)C)c3	9.2
116	FC(F)(F)CCCn1c2ccc(cc2c(C(=O)C3C(C3(C)C)(C)C)c1)C	9.2
117	Clc1c(C(=O)C2C(C2(C)C)(C)C)c3cc(c(cc3n1CCCC(F)(F)F)C)C	9.2
118	FC(F)(F)CCCc1c(c(c([nH]1)C(=O)C2C(C2(C)C)(C)C)C(=O)C)CC	9.2
119	FC(F)(F)CCCn1c2c(ncs2)c(C(=O)C3C(C3(C)C)(C)C)c1	9.2
120	FC(F)(F)CCCn1cc(C(=O)C2C(C2(C)C)(C)C)c3cc(C(C)C)ccc31	9.2
121	Clc1c(C(=O)C2C(C2(C)C)(C)C)c3ccc(cc3n1CCCC(F)(F)F)C	9.2
122	FC(F)(F)CCCc1cc2c(c(C(=O)C3C(C3(C)C)(C)C)c1)cns2	9.2
123	FC(F)(F)CCCn1c2ccc(cc2c(C(=O)C3C(C3(C)C)(C)C)c1O)C	9.2
124	FC(F)(F)CCCn1cc(C(=O)C2C(C2(C)C)(C)C)c3cc(CC)ccc31	9.2
125	FC(F)(F)CCCc1cc(C(=O)C2C(C2(C)C)(C)C)c(n1CC(F)(F)F)C	9.2
126	FC(F)(F)CCCc1c2c(c(o1)C(=O)C3C(C3(C)C)(C)C)C(=O)C=CS2	9.2
127	Brc1cc(CCCC(F)(F)F)cc(C(=O)C2C(C2(C)C)(C)C)c1F	9.2
128	FC(F)(F)CCCn1c(c(C(=O)C2C(C2(C)C)(C)C)c3ccccc31)C	9.2
129	Fc1c(cc(CCCC(F)(F)F)cc1C(=O)C2C(C2(C)C)(C)C)C	9.2
130	Fc1c(cc(CCCC(F)(F)F)cc1C(=O)C2C(C2(C)C)(C)C)C(F)(F)F	9.2
131	FC(F)(F)CCCc1c(c(c(s1)C(=O)C2C(C2(C)C)(C)C)C)C	9.2
132	FC(F)(F)CCCn1c(O)c(C(=O)C2C(C2(C)C)(C)C)c3ccccc31	9.2
133	FC(F)(F)CCCc1cc(CC)c(OC)c(C(=O)C2C(C2(C)C)(C)C)c1	9.2
134	FC(F)(F)CCCc1cc2c(OCC2)c(C(=O)C3C(C3(C)C)(C)C)c1	9.2
135	FC(F)(F)CCCn1nc(C(=O)C2C(C2(C)C)(C)C)c3cccn31	9.2
136	Clc1c(cc(CCCC(F)(F)F)cc1C(=O)C2C(C2(C)C)(C)C)C	9.2
137	FC(F)(F)CCCn1cc(C(=O)C2C(C2(C)C)(C)C)c3C(=O)NC=Cc31	9.2
138	FC(F)(F)CCCn1c2c(C[C@@H]3C[C@H]23)c(n1)C(=O)C4C(C4(C)C)(C)C	9.2
139	FC(F)(F)CCCc1cc(C(=O)C2C(C2(C)C)(C)C)cn1CC	9.2
140	FC(F)(F)CCCn1c2cccc(F)c2c(n1)C(=O)C3C(C3(C)C)(C)C	9.2
141	Clc1c(C(=O)C2C(C2(C)C)(C)C)c3cc(F)ccc3n1CCCC(F)(F)F	9.2
142	Clc1c(C(=O)C2C(C2(C)C)(C)C)c3cc(ccc3n1CCCC(F)(F)F)C	9.2
143	FC(F)(F)CCCc1c(O)c(N)cc(C(=O)C2C(C2(C)C)(C)C)c1	9.2
144	FC(F)(F)CCCc1c2c(c(s1)C(=O)C3C(C3(C)C)(C)C)C(=O)C=CS2	9.2
145	FC(F)(F)CCCn1c2ccc(cc2c(C(=O)C3C(C3(C)C)(C)C)c1C)C	9.2
146	FC(F)(F)CCCc1cc(C(=O)C2C(C2(C)C)(C)C)cc3CCCc31	9.1
147	FC(F)(F)CCCn1cc(C(=O)C2C(C2(C)C)(C)C)c3c1ccs3	9.1

148	FC(F)(F)CCCc1c2ccsc2c(s1)C(=O)C3C(C3(C)C)(C)C	9.1
149	FC(F)(F)CCCc1cc(C(=O)C2C(C2(C)C)(C)C)cc(c1)CO	9.1
150	FC(F)(F)CCCn1cc(C(=O)C2C(C2(C)C)(C)C)c3ccc(OC)cc31	9.1
151	FC(F)(F)CCCN1C=CC=C(C(=O)C2C(C2(C)C)(C)C)C1	9.1
152	Clc1ccc2c(n(nc2C(=O)C3C(C3(C)C)(C)C)CCCC(F)(F)F)c1	9.1
153	FC(F)(F)CCCn1cc(C(=O)C2C(C2(C)C)(C)C)c3cc(OC)c(OC)cc31	9.1
154	FC(F)(F)CCCn1cc(C(=O)C2C(C2(C)C)(C)C)c3cc(F)ccc31	9.1
155	FC(F)(F)CCCc1cc(C(=O)C2C(C2(C)C)(C)C)c(s1)SC	9.1
156	FC(F)(F)CCCn1cc(C(=O)C2C(C2(C)C)(C)C)c3cc(-c4ccco4)ccc31	9.1
157	FC(F)(F)CCCc1cn(C(=O)C2C(C2(C)C)(C)C)c3C(=O)NC=Cc31	9.1
158	FC(F)(F)CCCn1cc(C(=O)C2C(C2(C)C)(C)C)c3c1cco3	9.1
159	Clc1ccc2c(c(C(=O)C3C(C3(C)C)(C)C)cn2CCCC(F)(F)F)c1	9.1
160	FC(F)(F)CCCc1c2C=COC(=O)c2c(s1)C(=O)C3C(C3(C)C)(C)C	9.1
161	FC(F)(F)CCCc1c2C=COC(=O)c2c(o1)C(=O)C3C(C3(C)C)(C)C	9.1
162	FC(F)(F)CCCc1cc(C(=O)C2C(C2(C)C)(C)C)c3cnccn31	9.1
163	FC(F)(F)CCCn1c2c(c(C(=O)C3C(C3(C)C)(C)C)c1)C(OC=N2)=O	9.1
164	FC(F)(F)CCCc1c2c(n(C(=O)C3C(C3(C)C)(C)C)c1)C(=O)C=CS2	9.1
165	FC(F)(F)CCCn1c2c(c(n1)C(=O)C3C(C3(C)C)(C)C)ccs2	9.1
166	FC(F)(F)CCCn1cc(C(=O)C2C(C2(C)C)(C)C)c3c(F)cccc31	9.1
167	FC(F)(F)CCCc1c(c(c(o1)C(=O)C2C(C2(C)C)(C)C)C)C	9.1
168	FC(F)(F)CCCc1cc(F)c(c(C(=O)C2C(C2(C)C)(C)C)c1)C=O	9.1
169	FC(F)(F)CCCn1cc(C(=O)C2C(C2(C)C)(C)C)c3cc4c(OCO4)cc31	9.1
170	FC(F)(F)CCCn1c2cc(ccc2c(C(=O)C3C(C3(C)C)(C)C)c1C)C	9.1
171	FC(F)(F)CCCc1cc(C(=O)C2C(C2(C)C)(C)C)c3ccon31	9.1
172	FC(F)(F)CCCc1c(O)cc(F)c(C(=O)C2C(C2(C)C)(C)C)c1	9.1
173	FC(F)(F)CCCn1c2ccncc2c(n1)C(=O)C3C(C3(C)C)(C)C	9.1
174	FC(F)(F)CCCn1c(N)c(c(C(=O)C2C(C2(C)C)(C)C)c1)C#N	9.1
175	FC(F)(F)CCCc1ccc(F)c(C(=O)C2C(C2(C)C)(C)C)c1O	9.1
176	FC(F)(F)CCCn1c2c(c(n1)C(=O)C3C(C3(C)C)(C)C)cns2	9.1
177	FC(F)(F)CCCn1cc(C(=O)C2C(C2(C)C)(C)C)c3c(cccc31)C(OC)=O	9.1
178	FC(F)(F)CCCC1=C2C(SC=CS2)=C(C(=O)C3C(C3(C)C)(C)C)C1=O	9.1
179	FC(F)(F)CCCc1c2ccccc2c(o1)C(=O)C3C(C3(C)C)(C)C	9.1
180	FC(F)(F)CCCc1cc(c(O)c(C(=O)C2C(C2(C)C)(C)C)c1)C	9.1
181	FC(F)(F)CCCc1cc(cc(C(=O)C2C(C2(C)C)(C)C)c1)[C@@H](O)C	9.1
182	FC(F)(F)CCCc1cc(C(=O)C2C(C2(C)C)(C)C)c(s1)C(=O)C	9.1
183	FC(F)(F)CCCn1cc2-n(occs2)c(C(=O)C3C(C3(C)C)(C)C)c1	9.1
184	FC(F)(F)CCCc1c2C=COC(=O)c2n(C(=O)C3C(C3(C)C)(C)C)c1	9.1
185	FC(F)(F)CCCc1cc(C(=O)C2C(C2(C)C)(C)C)cc3c1csn3	9.1
186	FC(F)(F)CCCc1cc(C(=O)C2C(C2(C)C)(C)C)cn1CCC	9.1
187	FC(F)(F)CCCc1c(NC)ccc(C(=O)C2C(C2(C)C)(C)C)c1	9.1
188	Brc1c(O)c(C(=O)C2C(C2(C)C)(C)C)cc(CCCC(F)(F)F)c1O	9.1
189	FC(F)(F)CCCc1cc2ccc(O)nc2c(C(=O)C3C(C3(C)C)(C)C)c1	9.1

190	FC(F)(F)CCCc1cc(C(=O)C2C(C2(C)C)(C)C)cc(c1)C	9.1
191	FC(F)(F)CCCc1cc2ccnnc2c(C(=O)C3C(C3(C)C)(C)C)c1	9.1
192	FC(F)(F)CCCn1c2cocc2c(n1)C(=O)C3C(C3(C)C)(C)C	9.1
193	FC(F)(F)CCCn1cc(C(=O)C2C(C2(C)C)(C)C)c3ccccc31	9.1
194	FC(F)(F)CCCn1c2cc(F)ccc2c(n1)C(=O)C3C(C3(C)C)(C)C	9.1
195	FC(F)(F)CCCn1cc(C(=O)C2C(C2(C)C)(C)C)c3cc(OC)cc(c31)C	9.1
196	FC(F)(F)CCCc1c(c(c([nH]1)C(=O)C2C(C2(C)C)(C)C)CC)C	9
197	Clc1c(cc(CCCC(F)(F)F)cc1C(=O)C2C(C2(C)C)(C)C)C(F)(F)F	9
198	FC(F)(F)CCCn1c2cscc2c(n1)C(=O)C3C(C3(C)C)(C)C	9
199	FC(F)(F)CCCc1cc(C(=O)C2C(C2(C)C)(C)C)cc3c(c([nH]c31)C)C	9
200	FC(F)(F)CCCn1cc(C(=O)C2C(C2(C)C)(C)C)c3cc(c(cc31)C)C	9
201	FC(F)(F)CCCn1cc2-n(occo2)c(C(=O)C3C(C3(C)C)(C)C)c1	9
202	FC(F)(F)CCCc1c2C=CCCc2c(s1)C(=O)C3C(C3(C)C)(C)C	9
203	FC(F)(F)CCCc1cc(C(=O)C2C(C2(C)C)(C)C)c3n1cns3	9
204	FC(F)(F)CCCc1c(O)c(cc(C(=O)C2C(C2(C)C)(C)C)c1)CC	9
205	Clc1c(Cl)c(nn1CCCC(F)(F)F)C(=O)C2C(C2(C)C)(C)C	9
206	FC(F)(F)CCCc1cc2ccoc2c(C(=O)C3C(C3(C)C)(C)C)c1	9
207	FC(F)(F)CCCc1c2C=CSC(=O)c2c([nH]1)C(=O)C3C(C3(C)C)(C)C	9
208	FC(F)(F)CCCc1cc2c(OCCO2)c(C(=O)C3C(C3(C)C)(C)C)c1	9
209	FC(F)(F)CCCn1cc(sc-2cocnn21)C(=O)C3C(C3(C)C)(C)C	9
210	FC(F)(F)CCCn1cc2-n(oncs2)c(C(=O)C3C(C3(C)C)(C)C)c1	9
211	FC(F)(F)CCCn1c2c(c(C(=O)C3C(C3(C)C)(C)C)c1)ccs2	9
212	FC(F)(F)CCCn1c2ccc(cc2c(C(=O)C3C(C3(C)C)(C)C)c1)C(F)(F)F	9
213	FC(F)(F)CCCn1cc(C(=O)C2C(C2(C)C)(C)C)c3cc4c(OCCO4)cc31	9
214	FC(F)(F)CCCc1cc(cc(C(=O)C2C(C2(C)C)(C)C)c1)CC#N	9
215	FC(F)(F)CCCn1cc2-n(scns2)c(C(=O)C3C(C3(C)C)(C)C)c1	9
216	FC(F)(F)CCCn1c2ccc(cc2c(n1)C(=O)C3C(C3(C)C)(C)C)C#N	9
217	FC(F)(F)CCCc1c2c(c([nH]1)C(=O)C3C(C3(C)C)(C)C)C(=O)C=CS2	9
218	Clc1c(O)c(C(=O)C2C(C2(C)C)(C)C)cc(CCCC(F)(F)F)c1O	9
219	FC(F)(F)CCCn1cc(C(=O)C2C(C2(C)C)(C)C)c3c1cc[nH]3	9
220	FC(F)(F)CCCc1cc(C(=O)C2C(C2(C)C)(C)C)cc3cocc31	9
221	FC(F)(F)CCCc1cc(C(=O)C2C(C2(C)C)(C)C)c3n1ncs3	9
222	FC(F)(F)CCC[n+]1cc(C(=O)C2C(C2(C)C)(C)C)c(n1C)N	9
223	FC(F)(F)CCCc1c2CCCCc2c([nH]1)C(=O)C3C(C3(C)C)(C)C	9
224	FC(F)(F)CCCc1cc(C(=O)C2C(C2(C)C)(C)C)c(s1)OC	9
225	FC(F)(F)CCCn1cc(C(=O)C2C(C2(C)C)(C)C)c3cccc(O)c31	9
226	Clc1c(CC)cc(CCCC(F)(F)F)cc1C(=O)C2C(C2(C)C)(C)C	9
227	FC(F)(F)CCCc1c2C=CNC(=O)c2c([nH]1)C(=O)C3C(C3(C)C)(C)C	9
228	FC(F)(F)CCCn1cc(C(=O)C2C(C2(C)C)(C)C)c3cccc(OC)c31	9
229	Clc1cccc2c1n(CCCC(F)(F)F)cc2C(=O)C3C(C3(C)C)(C)C	9
230	Clc1c(C(=O)C2C(C2(C)C)(C)C)c3cc(Cl)ccc3n1CCCC(F)(F)F	9
231	Clc1cc(CCCC(F)(F)F)cc(C(=O)C2C(C2(C)C)(C)C)c1F	9

232	FC(F)(F)CCCc1c(O)c(F)c(F)c(C(=O)C2C(C2(C)C)(C)C)c1	9
233	Clc1c(OC)cc2c(n(CCCC(F)(F)F)cc2C(=O)C3C(C3(C)C)(C)C)c1	9
234	FC(F)(F)CCCn1cc(C(=O)C2C(C2(C)C)(C)C)c3cc(OC)ccc31	9
235	FC(F)(F)CCCc1cc(C(=O)C2C(C2(C)C)(C)C)cn1C(C)C	9
236	FC(F)(F)CCCn1c2C=COC(=O)c2c(C(=O)C3C(C3(C)C)(C)C)c1	9
237	FC(F)(F)CCCn1c2ccc([N+]([O-])=O)cc2c(n1)C(=O)C3C(C3(C)C)(C)C	9
238	FC(F)(F)CCCn1cc(C(=O)C2C(C2(C)C)(C)C)c3cc(OC(C)C)ccc31	9
239	FC(F)(F)CCCn1cc2-n(onco2)c(C(=O)C3C(C3(C)C)(C)C)c1	9
240	FC(F)(F)CCCc1cc(sc1CCC)C(=O)C2C(C2(C)C)(C)C	8.9
241	FC(F)(F)CCCc1c(O)c(cc(C(=O)C2C(C2(C)C)(C)C)c1)C(C)(C)C	8.9
242	FC(F)(F)CCCn1cc(C(=O)C2C(C2(C)C)(C)C)c3ccc(cc31)C	8.9
243	FC(F)(F)CCCn1cc(C(=O)C2C(C2(C)C)(C)C)c3cocc31	8.9
244	FC(F)(F)CCCn1cc(C(=O)C2C(C2(C)C)(C)C)c3cc([N+]([O-])=O)ccc31	8.9
245	FC(F)(F)CCCn1c2c(c(C(=O)C3C(C3(C)C)(C)C)c1)C(=O)NC(S2)=O	8.9
246	FC(F)(F)CCCc1c2cnccc2c(o1)C(=O)C3C(C3(C)C)(C)C	8.9
247	FC(F)(F)CCCn1cc(C(=O)C2C(C2(C)C)(C)C)c3cc(cc(c31)C)C	8.9
248	FC(F)(F)CCCc1cc2c(nco2)c(C(=O)C3C(C3(C)C)(C)C)c1	8.9
249	FC(F)(F)CCCn1cc(C(=O)C2C(C2(C)C)(C)C)c3ccc(c(c31)C)C	8.9
250	FC(F)(F)CCCn1c2ccc(cc2c(C(=O)C3C(C3(C)C)(C)C)c1)C#N	8.9
251	Brc1cc(C(=O)C2C(C2(C)C)(C)C)cc(CCCC(F)(F)F)c1NC	8.9
252	FC(F)(F)CCCc1c2c(c([nH]1)C(=O)C3C(C3(C)C)(C)C)cns2	8.9
253	FC(F)(F)CCCc1cc(n2c1cns2)C(=O)C3C(C3(C)C)(C)C	8.9
254	Clc1c(C(=O)C2C(C2(C)C)(C)C)c3cccc(F)c3n1CCCC(F)(F)F	8.9
255	FC(F)(F)CCCn1c2c(c(C(=O)C3C(C3(C)C)(C)C)c1)c(OC)nc(n2)N	8.9
256	FC(F)(F)CCCc1cc(cc(C(=O)C2C(C2(C)C)(C)C)c1O)C	8.9
257	FC(F)(F)CCCc1cc(C(=O)C2C(C2(C)C)(C)C)c(s1)C=O	8.9
258	FC(F)(F)CCCc1c2c(n(C(=O)C3C(C3(C)C)(C)C)c1)C(=O)C=CO2	8.9
259	FC(F)(F)CCCc1cc(C(=O)C2C(C2(C)C)(C)C)cc(c1C)C	8.9
260	FC(F)(F)CCCn1cc(C(=O)C2C(C2(C)C)(C)C)c3cccn31	8.9
261	FC(F)(F)CCCc1cc(SC)cc(C(=O)C2C(C2(C)C)(C)C)c1	8.9
262	FC(F)(F)CCCn1cc(C(=O)C2C(C2(C)C)(C)C)c3cc(cc(OC)c31)C	8.9
263	FC(F)(F)CCCn1cc(sn2n1ccs2)C(=O)C3C(C3(C)C)(C)C	8.9
264	FC(F)(F)CCCc1c2c(c(o1)C(=O)C3C(C3(C)C)(C)C)cco2	8.9
265	Clc1cc(C(=O)C2C(C2(C)C)(C)C)cc(CCCC(F)(F)F)c1NC	8.9
266	FC(F)(F)CCCn1cc(C(=O)C2C(C2(C)C)(C)C)c3ccc(cc31)C(=O)N	8.9
267	FC(F)(F)CCCn1c2cc(OC)ccc2c(n1)C(=O)C3C(C3(C)C)(C)C	8.9
268	Clc1cccc2c1c(C(=O)C3C(C3(C)C)(C)C)cn2CCCC(F)(F)F	8.9
269	FC(F)(F)CCCn1cc(C(=O)C2C(C2(C)C)(C)C)c3cc(OCC)ccc31	8.9
270	FC(F)(F)CCCc1cc(F)c(F)c(C(=O)C2C(C2(C)C)(C)C)c1	8.9
271	FC(F)(F)CCCc1c2c(nco2)cc(C(=O)C3C(C3(C)C)(C)C)c1	8.9
272	Clc1c(C(=O)C2C(C2(C)C)(C)C)c3ccc(Cl)cc3n1CCCC(F)(F)F	8.9
273	FC(F)(F)CCCc1cc(F)c(F)c(C(=O)C2C(C2(C)C)(C)C)c1O	8.9

274	FC(F)(F)CCCc1c(O)c2ccccc2c(C(=O)C3C(C3(C)C)(C)C)c1	8.9
275	FC(F)(F)CCCn1c2c(c(C(=O)C3C(C3(C)C)(C)C)c1)cns2	8.9
276	FC(F)(F)CCCc1cc2c(OCO2)c(C(=O)C3C(C3(C)C)(C)C)c1	8.9
277	FC(F)(F)CCCn1cc(n2n1scco2)C(=O)C3C(C3(C)C)(C)C	8.9
278	FC(F)(F)CCCc1c2c(c(s1)C(=O)C3C(C3(C)C)(C)C)CCC(C2)(C)C	8.9
279	FC(F)(F)CCCn1c2c(c(C(=O)C3C(C3(C)C)(C)C)c1)c(O)nc(n2)N	8.9
280	FC(F)(F)CCCn1c2cnsc2c(n1)C(=O)C3C(C3(C)C)(C)C	8.9
281	FC(F)(F)CCCn1cc(C(=O)C2C(C2(C)C)(C)C)c3cccc(C(C)C)c31	8.9
282	FC(F)(F)CCCc1cc(cc(C(=O)C2C(C2(C)C)(C)C)c1)CC	8.9
283	Clc1cc(CCCC(F)(F)F)cc(C(=O)C2C(C2(C)C)(C)C)c1C#N	8.9
284	Clc1ccc2c(n(CCCC(F)(F)F)cc2C(=O)C3C(C3(C)C)(C)C)c1	8.9
285	FC(F)(F)CCCn1c2c(cccc2c(C(=O)C3C(C3(C)C)(C)C)c1)C	8.9
286	FC(F)(F)CCCn1cc(C(=O)C2C(C2(C)C)(C)C)c3cscc31	8.9
287	FC(F)(F)CCCn1cc(C(=O)C2C(C2(C)C)(C)C)c3ccc(F)cc31	8.9
288	Clc1c(F)cc(CCCC(F)(F)F)cc1C(=O)C2C(C2(C)C)(C)C	8.8
289	FC(F)(F)CCCn1c2cnccc2c(n1)C(=O)C3C(C3(C)C)(C)C	8.8
290	FC(F)(F)CCCc1c2C=CC(=O)Nc2c(s1)C(=O)C3C(C3(C)C)(C)C	8.8
291	FC(F)(F)CCCc1ccc(F)c(C(=O)C2C(C2(C)C)(C)C)c1	8.8
292	Clc1cc(CCCC(F)(F)F)cc(C(=O)C2C(C2(C)C)(C)C)c1	8.8
293	FC(F)(F)CCCc1cc2c(nccn2)c(C(=O)C3C(C3(C)C)(C)C)c1	8.8
294	FC(F)(F)CCCc1cc2cscc2c(C(=O)C3C(C3(C)C)(C)C)c1	8.8
295	FC(F)(F)CCC[n+]1c2n(c(C(=O)C3C(C3(C)C)(C)C)c1)CCCS2	8.8
296	Brc1c(O)c(C(=O)C2C(C2(C)C)(C)C)c(O)c(CCCC(F)(F)F)c1	8.8
297	FC(F)(F)CCCn1cc(C(=O)C2C(C2(C)C)(C)C)c3cccc(F)c31	8.8
298	FC(F)(F)CCCc1cc(C(=O)C2C(C2(C)C)(C)C)cn1C3CC3	8.8
299	FC(F)(F)CCCc1c2c(n(C(=O)C3C(C3(C)C)(C)C)c1)C(SC=N2)=O	8.8
300	FC(F)(F)CCCc1c(c(c([nH]1)C(=O)C2C(C2(C)C)(C)C)C)C	8.8
301	FC(F)(F)CCCc1c2c(c([nH]1)C(=O)C3C(C3(C)C)(C)C)C(SC=N2)=O	8.8
302	FC(F)(F)CCCc1cc2c(cccc2c(C(=O)C3C(C3(C)C)(C)C)c1)C	8.8
303	FC(F)(F)CCCN1C2=CSC(=O)N2C(C(=O)C3C(C3(C)C)(C)C)=C1	8.8
304	FC(F)(F)CCCn1c2cc(F)ccc2c(C(=O)C3C(C3(C)C)(C)C)c1C	8.8
305	Brc1cc(CCCC(F)(F)F)cc(C(=O)C2C(C2(C)C)(C)C)c1Cl	8.8
306	FC(F)(F)CCCn1c2cc(OC)cc(OC)c2c(C(=O)C3C(C3(C)C)(C)C)c1	8.8
307	FC(F)(F)CCCc1cc2cnccc2c(C(=O)C3C(C3(C)C)(C)C)c1	8.8
308	Clc1ccc(CCCC(F)(F)F)c(O)c1C(=O)C2C(C2(C)C)(C)C	8.8
309	Fc1c(C(=O)C2C(C2(C)C)(C)C)cc(CCCC(F)(F)F)cc1C[N+]	8.8
310	FC(F)(F)CCCn1cc(C(=O)C2C(C2(C)C)(C)C)c3cccc(OCC)c31	8.8
311	FC(F)(F)CCCc1cc2c(N=CC(S2)=O)c(C(=O)C3C(C3(C)C)(C)C)c1	8.8
312	FC(F)(F)CCCc1cc(C(=O)C2C(C2(C)C)(C)C)cc(c1O)C(C)C	8.8
313	Clc1c(O)c(C(=O)C2C(C2(C)C)(C)C)c(O)c(CCCC(F)(F)F)c1	8.8
314	FC(F)(F)CCCc1cc2c(n[nH]n2)c(C(=O)C3C(C3(C)C)(C)C)c1	8.8
315	FC(F)(F)CCCc1cc(OCC)cc(C(=O)C2C(C2(C)C)(C)C)c1	8.8

316	FC(F)(F)CCCn1c2ccc(F)cc2c(C(=O)C3C(C3(C)C)(C)C)c1C	8.8
317	FC(F)(F)CCCc1c2cnccc2c(s1)C(=O)C3C(C3(C)C)(C)C	8.8
318	FC(F)(F)CCCc1c2ccsc2c([nH]1)C(=O)C3C(C3(C)C)(C)C	8.8
319	FC(F)(F)CCCn1cc(C(=O)C2C(C2(C)C)(C)C)c3cn[nH]c31	8.8
320	FC(F)(F)CCCc1cc2ccncc2c(C(=O)C3C(C3(C)C)(C)C)c1	8.8
321	FC(F)(F)CCCn1cc(C(=O)C2C(C2(C)C)(C)C)c3c(cccc31)C	8.8
322	FC(F)(F)CCCn1cc2-n(scco2)c(C(=O)C3C(C3(C)C)(C)C)c1	8.8
323	FC(F)(F)CCCn1c2ccncc2c(C(=O)C3C(C3(C)C)(C)C)c1	8.8
324	Brc1c(cc(C(=O)C2C(C2(C)C)(C)C)cc1CCCC(F)(F)F)C	8.8
325	FC(F)(F)CCCc1cc2ccsc2c(C(=O)C3C(C3(C)C)(C)C)c1	8.8
326	FC(F)(F)CCCN1C=C(C(=O)C2C(C2(C)C)(C)C)CCC1	8.8
327	FC(F)(F)CCCc1cc2ccccc2c(C(=O)C3C(C3(C)C)(C)C)c1O	8.8
328	FC(F)(F)CCCc1cc2ccnn2c(C(=O)C3C(C3(C)C)(C)C)c1	8.8
329	Clc1c(Cl)cc(CCCC(F)(F)F)cc1C(=O)C2C(C2(C)C)(C)C	8.8
330	FC(F)(F)CCCn1c2cccn2c(n1)C(=O)C3C(C3(C)C)(C)C	8.8
331	FC(F)(F)CCCc1c2c(NC(S2)=O)c(s1)C(=O)C3C(C3(C)C)(C)C	8.8
332	FC(F)(F)CCCn1cc(C(=O)C2C(C2(C)C)(C)C)c3c1nccn3	8.8
333	Brc1c(CCCC(F)(F)F)cc([nH]1)C(=O)C2C(C2(C)C)(C)C	8.8
334	FC(F)(F)CCC[n+]1cc(n2CCCc21)C(=O)C3C(C3(C)C)(C)C	8.8
335	FC(F)(F)CCCc1cc(C(=O)C2C(C2(C)C)(C)C)cc(C3(CC3)C#N)c1	8.8
336	FC(F)(F)CCCc1cc(F)c(O)c(C(=O)C2C(C2(C)C)(C)C)c1	8.8
337	Brc1cc(CCCC(F)(F)F)c(O)c(C(=O)C2C(C2(C)C)(C)C)c1	8.7
338	FC(F)(F)CCCc1cc(F)c(c(C(=O)C2C(C2(C)C)(C)C)c1)C#N	8.7
339	FC(F)(F)CCCc1cc(cc(C(=O)C2C(C2(C)C)(C)C)c1)C(C)(C)C	8.7
340	FC(F)(F)CCCc1c2ccccc2cc(C(=O)C3C(C3(C)C)(C)C)c1	8.7
341	Brc1cc(CCCC(F)(F)F)cc(C(=O)C2C(C2(C)C)(C)C)c1C	8.7
342	FC(F)(F)CCCn1cc(C(=O)C2C(C2(C)C)(C)C)c3c1ncs3	8.7
343	FC(F)(F)CCCc1ccc(c(C(=O)C2C(C2(C)C)(C)C)c1)C#C	8.7
344	FC(F)(F)CCCc1ccc(c(C(=O)C2C(C2(C)C)(C)C)c1)C#N	8.7
345	FC(F)(F)CCCc1cc(F)c(N)c(C(=O)C2C(C2(C)C)(C)C)c1	8.7
346	FC(F)(F)c1cc(CCCC(F)(F)F)cc(C(=O)C2C(C2(C)C)(C)C)c1O	8.7
347	FC(F)(F)CCCN1c2c(C(=O)C(C(=O)C3C(C3(C)C)(C)C)=C1)csn2	8.7
348	FC(F)(F)CCCn1cc(C(=O)C2C(C2(C)C)(C)C)c3cccc(c31)CC	8.7
349	Brc1cc(CCCC(F)(F)F)cc(C(=O)C2C(C2(C)C)(C)C)c1O	8.7
350	FC(F)(F)CCCN1C=CCC(C(=O)C2C(C2(C)C)(C)C)=C1	8.7
351	FC(F)(F)CCCc1c2ccccc2c([nH]1)C(=O)C3C(C3(C)C)(C)C	8.7
352	FC(F)(F)CCCn1cc(C(=O)C2C(C2(C)C)(C)C)c3ccc(cc31)C(F)(F)F	8.7
353	FC(F)(F)CCCc1cccc(C(=O)C2C(C2(C)C)(C)C)c1	8.7
354	FC(F)(F)CCCn1c2c(c(C(=O)C3C(C3(C)C)(C)C)c1)c(OC)ncn2	8.7
355	Clc1c(Cl)c(sc1CCCC(F)(F)F)C(=O)C2C(C2(C)C)(C)C	8.7
356	FC(F)(F)CCCc1cc2cccn2c(C(=O)C3C(C3(C)C)(C)C)c1	8.7
357	FC(F)(F)CCCn1c2c(c(C(=O)C3C(C3(C)C)(C)C)c1C)ccs2	8.7

358	FC(F)(F)CCCn1c2c(c(C(=O)C3C(C3(C)C)(C)C)c1)cco2	8.7
359	Clc1cc(CCCC(F)(F)F)c(O)c(C(=O)C2C(C2(C)C)(C)C)c1	8.7
360	FC(F)(F)CCCc1cc2c(nnnn2)c(C(=O)C3C(C3(C)C)(C)C)c1	8.7
361	FC(F)(F)CCCn1cc(n2cccc21)C(=O)C3C(C3(C)C)(C)C	8.7
362	FC(F)(F)CCCc1cc(C(=O)C2C(C2(C)C)(C)C)cc(N(C)C)c1	8.7
363	FC(F)(F)CCCn1cc2-n(ocns2)c(C(=O)C3C(C3(C)C)(C)C)c1	8.7
364	FC(F)(F)CCCc1cc([n+]([O-])c2ccccc21)C(=O)C3C(C3(C)C)(C)C	8.7
365	FC(F)(F)CCCn1cc(C(=O)C2C(C2(C)C)(C)C)cc1C[N+]C	8.7
366	FC(F)(F)CCCn1c2c(c(C(=O)C3C(C3(C)C)(C)C)c1)ccc(OC)n2	8.7
367	FC(F)(F)CCCn1c2c(c(C(=O)C3C(C3(C)C)(C)C)c1)C(=O)C=NO2	8.7
368	Clc1cc(CCCC(F)(F)F)cc(C(=O)C2C(C2(C)C)(C)C)c1O	8.7
369	FC(F)(F)CCCc1cccc(C(=O)C2C(C2(C)C)(C)C)c1O	8.7
370	FC(F)(F)CCCn1c2c(c(C(=O)C3C(C3(C)C)(C)C)c1)csn2	8.7
371	FC(F)(F)CCCc1c2c(cc(C(=O)C3C(C3(C)C)(C)C)c1)cns2	8.7
372	FC(F)(F)CCCn1cc(C(=O)C2C(C2(C)C)(C)C)c3c1NC(S3)=O	8.7
373	FC(F)(F)CCCc1c(CC)c(c(s1)C(=O)C2C(C2(C)C)(C)C)C#N	8.6
374	FC(F)(F)CCCc1cc([N+]([O-])=O)c(O)c(C(=O)C2C(C2(C)C)(C)C)c1	8.6
375	Clc1ccc2c(n(CCCC(F)(F)F)cc2C(=O)C3C(C3(C)C)(C)C)c1C	8.6
376	FC(F)(F)CCCn1cc(C(=O)C2C(C2(C)C)(C)C)cc1C	8.6
377	FC(F)(F)CCCc1cc(F)c(c(C(=O)C2C(C2(C)C)(C)C)c1)C	8.6
378	FC(F)(F)CCCc1cc(F)c(SC)c(C(=O)C2C(C2(C)C)(C)C)c1	8.6
379	FC(F)(F)CCCn1cc(C(=O)C2C(C2(C)C)(C)C)c3c(ccc(OC)c31)C	8.6
380	FC(F)(F)CCCc1c(sc(C(=O)C2C(C2(C)C)(C)C)c1)C(C)C	8.6
381	FC(F)(F)CCCc1cc2c(nncn2)c(C(=O)C3C(C3(C)C)(C)C)c1	8.6
382	FC(F)(F)CCCc1cc2c(OC(S2)=O)c(C(=O)C3C(C3(C)C)(C)C)c1	8.6
383	Clc1ccc(CCCC(F)(F)F)cc1C(=O)C2C(C2(C)C)(C)C	8.6
384	FC(F)(F)CCCn1cc(C(=O)C2C(C2(C)C)(C)C)c3c(O)cccc31	8.6
385	Brc1cc([nH]c1C(=O)C2C(C2(C)C)(C)C)CCCC(F)(F)F	8.6
386	FC(F)(F)CCCc1c(c(c(s1)C(=O)C2C(C2(C)C)(C)C)C#N)C	8.6
387	FC(F)(F)CCCn1cc(C(=O)C2C(C2(C)C)(C)C)c3c1SC(=O)N3	8.6
388	FC(F)(F)CCCn1c2c(SC(S2)=O)c(C(=O)C3C(C3(C)C)(C)C)c1	8.6
389	Brc1c(Cl)cc(CCCC(F)(F)F)cc1C(=O)C2C(C2(C)C)(C)C	8.6
390	FC(F)(F)CCCn1cc(C(=O)C2C(C2(C)C)(C)C)c3C[N+]CCc31	8.6
391	FC(F)(F)c1cn(CCCC(F)(F)F)cc1C(=O)C2C(C2(C)C)(C)C	8.6
392	Brc1cc(C(=O)C2C(C2(C)C)(C)C)cn1CCCC(F)(F)F	8.6
393	Clc1ccc(OC)c2c1n(CCCC(F)(F)F)cc2C(=O)C3C(C3(C)C)(C)C	8.6
394	FC(F)(F)CCCc1cc(C(=O)C2C(C2(C)C)(C)C)c3c(n1)cccn3	8.6
395	FC(F)(F)CCCn1c2cc(cc(c2c(C(=O)C3C(C3(C)C)(C)C)c1)C)C	8.6
396	Clc1c(OC)cc(CCCC(F)(F)F)cc1C(=O)C2C(C2(C)C)(C)C	8.6
397	FC(F)(F)CCCc1cc(F)cc(C(=O)C2C(C2(C)C)(C)C)c1	8.6
398	FC(F)(F)CCCc1cn2c(c(C(=O)C3C(C3(C)C)(C)C)c1)ccn2	8.6
399	FC(F)(F)CCCn1c2c(O)cccc2c(C(=O)C3C(C3(C)C)(C)C)c1C	8.6

400	FC(F)(F)CCCc1cc(sc1CC)C(=O)C2C(C2(C)C)(C)C	8.6
401	FC(F)(F)CCCn1cc(C(=O)C2C(C2(C)C)(C)C)c3ccncc31	8.6
402	FC(F)(F)CCCc1cc(C(C)C)cc(C(=O)C2C(C2(C)C)(C)C)c1	8.6
403	FC(F)(F)CCCc1cc2c(OC(=O)C=N2)c(C(=O)C3C(C3(C)C)(C)C)c1	8.6
404	FC(F)(F)CCCC1=CN(N2C(SC=C12)=O)C(=O)C3C(C3(C)C)(C)C	8.6
405	FC(F)(F)CCCn1c2c(c(C(=O)C3C(C3(C)C)(C)C)c1)cccn2	8.6
406	FC(F)(F)CCCc1cc(c([nH]1)C(=O)C2C(C2(C)C)(C)C)C	8.5
407	FC(F)(F)CCCc1cc(C(=O)C2C(C2(C)C)(C)C)c(o1)C	8.5
408	FC(F)(F)CCCn1cc(C(=O)C2C(C2(C)C)(C)C)c3ccc4ccccc4c31	8.5
409	Clc1c(Cl)c([nH]c1CCCC(F)(F)F)C(=O)C2C(C2(C)C)(C)C	8.5
410	FC(F)(F)CCCc1cc2c(onn2)c(C(=O)C3C(C3(C)C)(C)C)c1	8.5
411	FC(F)(F)CCCc1cc(C(=O)C2C(C2(C)C)(C)C)cc(c1)C(F)(F)F	8.5
412	FC(F)(F)CCCn1c(c(c(C(=O)C2C(C2(C)C)(C)C)c1N)C)C	8.5
413	FC(F)(F)CCCn1cc(C(=O)C2C(C2(C)C)(C)C)c3c(C)ccc(c31)C	8.5
414	FC(F)(F)CCCn1cc(C(=O)C2C(C2(C)C)(C)C)c3c1cns3	8.5
415	FC(F)(F)CCCc1cc2c(ncnn2)c(C(=O)C3C(C3(C)C)(C)C)c1	8.5
416	FC(F)(F)CCCc1cc(OC)cc(C(=O)C2C(C2(C)C)(C)C)c1	8.5
417	FC(F)(F)CCCn1c2c(SC(=O)C=N2)c(C(=O)C3C(C3(C)C)(C)C)c1	8.5
418	FC(F)(F)CCCc1cc(F)cc(C(=O)C2C(C2(C)C)(C)C)c1O	8.5
419	FC(F)(F)CCCc1cn(C(=O)C2C(C2(C)C)(C)C)c[n+]1C	8.5
420	FC(F)(F)CCCc1ccc(c(C(=O)C2C(C2(C)C)(C)C)c1)C	8.5
421	FC(F)(F)CCCc1c2c(c([nH]1)C(=O)C3C(C3(C)C)(C)C)cco2	8.5
422	Brc1c(C(=O)C2C(C2(C)C)(C)C)cc(s1)CCCC(F)(F)F	8.5
423	FC(F)(F)CCCc1cc2c(nno2)c(C(=O)C3C(C3(C)C)(C)C)c1	8.5
424	FC(F)(F)CCCc1cc(C(=O)C2C(C2(C)C)(C)C)c3c(n1)ccnn3	8.5
425	FC(F)(F)CCCc1cc(cc(C(=O)C2C(C2(C)C)(C)C)c1)C#N	8.5
426	FC(F)(F)CCCn1c2c(c(C(=O)C3C(C3(C)C)(C)C)c1)c(O)ncn2	8.5
427	FC(F)(F)CCCc1cc(F)c(NC)c(C(=O)C2C(C2(C)C)(C)C)c1	8.5
428	Sc1ccc(CCCC(F)(F)F)cc1C(=O)C2C(C2(C)C)(C)C	8.5
429	FC(F)(F)CCCc1cn2-c(sncs2)c([nH]1)C(=O)C3C(C3(C)C)(C)C	8.5
430	Clc1c(Cl)cc(CCCC(F)(F)F)c(O)c1C(=O)C2C(C2(C)C)(C)C	8.5
431	FC(F)(F)CCCC1=CN2C(SC=C2C(C(=O)C3C(C3(C)C)(C)C)=C1)=O	8.5
432	Brc1cc(CCCC(F)(F)F)cc(C(=O)C2C(C2(C)C)(C)C)c1	8.5
433	FC(F)(F)CCC[n+]1cc(n2CCCCCc21)C(=O)C3C(C3(C)C)(C)C	8.5
434	FC(F)(F)CCCn1cc(C(=O)C2C(C2(C)C)(C)C)c3ccc(nc31)C	8.5
435	FC(F)(F)CCCc1cc(sc1CO)C(=O)C2C(C2(C)C)(C)C	8.5
436	FC(F)(F)CCCn1cc2-n(onno2)c(C(=O)C3C(C3(C)C)(C)C)c1	8.4
437	Clc1cc(CCCC(F)(F)F)c(O)c(C(=O)C2C(C2(C)C)(C)C)c1C	8.4
438	FC(F)(F)CCCc1c[n+](C(=O)C2C(C2(C)C)(C)C)cn1C	8.4
439	Brc1cc(CCCC(F)(F)F)c(N)c(C(=O)C2C(C2(C)C)(C)C)c1	8.4
440	Clc1c(Cl)c(O)c(CCCC(F)(F)F)cc1C(=O)C2C(C2(C)C)(C)C	8.4
441	FC(F)(F)CCCc1ccc(N#C)c(C(=O)C2C(C2(C)C)(C)C)c1	8.4

442	FC(F)(F)CCCn1cc2-n(snco2)c(C(=O)C3C(C3(C)C)(C)C)c1	8.4
443	FC(F)(F)CCCc1cc(sc1CCO)C(=O)C2C(C2(C)C)(C)C	8.4
444	FC(F)(F)CCCn1cc(C(=O)C2C(C2(C)C)(C)C)c(n1)C(=O)C	8.4
445	FC(F)(F)CCCn1cc(C(=O)C2C(C2(C)C)(C)C)c3c1cn[nH]3	8.4
446	FC(F)(F)CCC[n+]1c(N)ccc(C(=O)C2C(C2(C)C)(C)C)c1	8.4
447	FC(F)(F)CCCn1c2c(c(C(=O)C3C(C3(C)C)(C)C)c1)cncn2	8.4
448	FC(F)(F)CCCc1c[n+](cc(C(=O)C2C(C2(C)C)(C)C)c1)C	8.4
449	FC(F)(F)CCCn1cc2-n(ocno2)c(C(=O)C3C(C3(C)C)(C)C)c1	8.4
450	FC(F)(F)CCCc1cc(nc2cccn21)C(=O)C3C(C3(C)C)(C)C	8.4
451	FC(F)(F)CCCc1c2cnoc2cc(C(=O)C3C(C3(C)C)(C)C)c1	8.4
452	FC(F)(F)CCCc1c2c([C@H]3CC[C@@H]2C3)c([nH]1)C(=O)C4C(C4(C)C)(C)C	8.4
453	FC(F)(F)CCCc1c(c(c([nH]1)C(=O)C2C(C2(C)C)(C)C)C#N)C	8.4
454	FC(F)(F)CCCc1cc(C(=O)C2C(C2(C)C)(C)C)cc3cncn31	8.4
455	FC(F)(F)CCCc1cc(cc(C(=O)C2C(C2(C)C)(C)C)c1N)C	8.4
456	FC(F)(F)CCCc1cnc(c(C(=O)C2C(C2(C)C)(C)C)c1)C#N	8.3
457	FC(F)(F)CCCn1cc2-n(sncs2)c(C(=O)C3C(C3(C)C)(C)C)c1	8.3
458	FC(F)(F)CCCn1cc(C(=O)C2C(C2(C)C)(C)C)c3cncn31	8.3
459	FC(F)(F)CCCn1cc(C(=O)C2C(C2(C)C)(C)C)cc1CO	8.3
460	FC(F)(F)CCCc1c2ccncc2c([nH]1)C(=O)C3C(C3(C)C)(C)C	8.3
461	Clc1c(C(=O)C2C(C2(C)C)(C)C)cc(s1)CCCC(F)(F)F	8.3
462	Brc1c(C(=O)C2C(C2(C)C)(C)C)cc(o1)CCCC(F)(F)F	8.3
463	FC(F)(F)CCCn1cc2-n([nH]ccs2)c(C(=O)C3C(C3(C)C)(C)C)c1	8.3
464	FC(F)(F)CCCc1cc2c(c(C(=O)C3C(C3(C)C)(C)C)c1)ccs2	8.3
465	FC(F)(F)CCCn1c2c(SC(O2)=O)c(C(=O)C3C(C3(C)C)(C)C)c1	8.3
466	FC(F)(F)CCCn1c2c(c(C(=O)C3C(C3(C)C)(C)C)c1)cnc(n2)C	8.3
467	FC(F)(F)CCCn1cc(C(=O)C2C(C2(C)C)(C)C)cc1C[N+]	8.3
468	FC(F)(F)CCCc1c2c(scn2)c([nH]1)C(=O)C3C(C3(C)C)(C)C	8.3
469	FC(F)(F)CCCc1cc(S(=O)(=O)N)cc(C(=O)C2C(C2(C)C)(C)C)c1	8.2
470	FC(F)(F)CCCc1cc(C(=O)C2C(C2(C)C)(C)C)c(s1)C#N	8.2
471	FC(F)(F)CCCc1cc(C(=O)C2C(C2(C)C)(C)C)c(s1)C	8.2
472	FC(F)(F)CCCc1cc(C(=O)C2C(C2(C)C)(C)C)c3c(scn3)n1	8.2
473	FC(F)(F)CCCn1cc(C(=O)C2C(C2(C)C)(C)C)c3ccnn31	8.2
474	FC(F)(F)CCCc1cc(C(=O)C2C(C2(C)C)(C)C)c(c(n1)C)C#N	8.2
475	FC(F)(F)CCCc1c(C[N+])cc([nH]1)C(=O)C2C(C2(C)C)(C)C	8.2
476	Clc1c2c(n(CCCC(F)(F)F)cc2C(=O)C3C(C3(C)C)(C)C)ncn1	8.2
477	FC(F)(F)CCCn1c2c(c(C(=O)C3C(C3(C)C)(C)C)c1)con2	8.2
478	Clc1c(C(=O)C2C(C2(C)C)(C)C)cc(o1)CCCC(F)(F)F	8.1
479	Brc1c(c([nH]c1CCCC(F)(F)F)C(=O)C2C(C2(C)C)(C)C)C#N	8.1
480	Clc1cc(CCCC(F)(F)F)cc(C(=O)C2C(C2(C)C)(C)C)c1C	8.1
481	FC(F)(F)CCCn1c2nccn2c(C(=O)C3C(C3(C)C)(C)C)c1	8.1
482	Clc1c[n+](C(=O)C2C(C2(C)C)(C)C)cn1CCCC(F)(F)F	8.1
483	FC(F)(F)CCCc1cc(sc1C[N+])C(=O)C2C(C2(C)C)(C)C	8.1

484	FC(F)(F)CCCc1c(CC[N+])cc([nH]1)C(=O)C2C(C2(C)C)(C)C	8.1
485	FC(F)(F)CCCc1cc2c(c(C(=O)C3C(C3(C)C)(C)C)c1)cncn2	8.1
486	FC(F)(F)CCCn1c2cncn2c(C(=O)C3C(C3(C)C)(C)C)c1	8.1
487	FC(F)(F)CCCn1c2c(c(C(=O)C3C(C3(C)C)(C)C)c1)cno2	8.1
488	Sc1c2c(n(CCCC(F)(F)F)cc2C(=O)C3C(C3(C)C)(C)C)ncn1	8.1
489	FC(F)(F)CCCn1c2ccccc2[n+](C(=O)C3C(C3(C)C)(C)C)c1	8.1
490	FC(F)(F)CCCn1cc(C(=O)C2C(C2(C)C)(C)C)c(n1)C(F)F	8.1
491	FC(F)(F)CCCn1c2cnncc2c(C(=O)C3C(C3(C)C)(C)C)c1	8
492	FC(F)(F)CCC[n+]1cc(F)c(OC)c(C(=O)C2C(C2(C)C)(C)C)c1	8
493	FC(F)(F)CCCn1c2c(c(C(=O)C3C(C3(C)C)(C)C)c1)ccnn2	8
494	FC(F)(F)CCCn1c2c(snn2)c(C(=O)C3C(C3(C)C)(C)C)c1	8
495	FC(F)(F)CCC[n+]1cc(cc(C(=O)C2C(C2(C)C)(C)C)c1)C	7.9
496	FC(F)(F)CCC[n+]1ccc(c(C(=O)C2C(C2(C)C)(C)C)c1)C	7.8
497	FC(F)(F)CCCn1c2c(c(C(=O)C3C(C3(C)C)(C)C)c1)c(ncn2)NO	7.8
498	FC(F)(F)CCC[n+]1cccc(C(=O)C2C(C2(C)C)(C)C)c1	7.7
499	Brc1c[n+](CCCC(F)(F)F)cc(C(=O)C2C(C2(C)C)(C)C)c1	7.7
500	FC(F)(F)CCC[n+]1cc(C(=O)C2C(C2(C)C)(C)C)cc3ccccc31	7.6

Table S16. List, SMILE and predicted pK_i values for Series 6 in CB_2 receptor.

N°	SMILES	Pred pK_i
1	Clc1c(nn(C)c1)C(=O)c2c3ccccc3n(CCCC(F)(F)F)c2	10.2
2	Clc1cccc2c1c(no2)-c3c4ccccc4n(CCCC(F)(F)F)c3	10.1
3	FC(F)(F)CCCn1cc(c2ccccc21)C(=O)c3ccn4ccsc34	10
4	Brc1c[nH]nc1C(=O)c2c3ccccc3n(CCCC(F)(F)F)c2	9.9
5	Clc1cc(N)ccc1C(=O)c2c3ccccc3n(CCCC(F)(F)F)c2	9.9
6	FC(F)(F)CCCn1cc(C=2[C@@H]3CCCC[C@H]3ON2)c4ccccc41	9.8
7	FC(F)(F)CCCn1cc(c2ccccc21)C(=O)c3cccc4c3cc[nH]4	9.8
8	FC(F)(F)CCCn1cc(C(=O)[C@H]2C[C@H]3C=C[C@@H]2C3)c4ccccc41	9.8
9	FC(F)(F)CCCn1cc(C(=O)[C@@]23CCC[C@H]4C[C@@H](C2)CC[C@H]43)c5ccccc51	9.8
10	FC(F)(F)CCCn1cc(c2ccccc21)C(=O)[C@@H](C(C)(C)C)C#N	9.7
11	FC(F)(F)CCCn1cc(c2ccccc21)C(=O)c3ccccc3OC	9.7
12	FC(F)(F)CCCn1cc(c2ccccc21)-c3c4cc(OC)ccc4on3	9.7
13	FC(F)(F)CCCn1cc(-c2c3c(no2)CCCC3)c4ccccc41	9.7
14	Clc1c(Cl)snc1C(=O)c2c3ccccc3n(CCCC(F)(F)F)c2	9.7
15	FC(F)(F)CCCn1cc(C(=O)C2(OC)CCCC2)c3ccccc31	9.7
16	FC(F)(F)CCCn1c2ccccc2c(S(=O)(=O)c3ccccc3F)c1	9.6
17	FC(F)(F)CCCn1cc(-c2c3c(no2)CCC3)c4ccccc41	9.6
18	S=C(N)[C@H](C(=O)c1c2ccccc2n(CCCC(F)(F)F)c1)CC	9.6
19	FC(F)(F)CCCn1cc(c2ccccc21)C(=O)c3csc(c3CC)C	9.6
20	FC(F)(F)CCCn1cc(C=2[C@@H]3CCC[C@@H]3ON2)c4ccccc41	9.6
21	FC(F)(F)CCCn1cc(c2ccccc21)C(=O)c3c4c(OCCO4)cs3	9.6

22	FC(F)(F)CCCn1cc(c2ccccc21)-c3c(C)c(no3)C	9.6
23	FC(F)(F)CCCn1cc(-c2c3c(no2)CC[C@@H](C3)C)c4ccccc41	9.6
24	Brc1c(noc1-c2c3ccccc3n(CCCC(F)(F)F)c2)C	9.6
25	FC(F)(F)CCCn1c2ccccc2c(C(=O)C34CC5CC(C3)CC(C4)C5)c1	9.6
26	Brc1ccc2c(c(no2)-c3c4ccccc4n(CCCC(F)(F)F)c3)c1	9.5
27	Brc1c(onc1-c2c3ccccc3n(CCCC(F)(F)F)c2)C	9.5
28	Clc1cc(c(cc1C(=O)c2cn(CCCC(F)(F)F)c3ccccc32)C)C	9.5
29	FC(F)(F)CCCn1cc(C(=O)[C@H](CCC)C)c2ccccc21	9.5
30	FC(F)(F)CCCn1cc(c2ccccc21)-c3c4ccccc4on3	9.5
31	Clc1c[nH]nc1C(=O)c2c3ccccc3n(CCCC(F)(F)F)c2	9.5
32	FC(F)(F)CCCn1cc(c2ccccc21)C(=O)c3c([N+]([O-])=O)ccc(c3)C	9.5
33	Clc1ccc2c(c(no2)-c3c4ccccc4n(CCCC(F)(F)F)c3)c1	9.5
34	Brc1cc(C(=O)c2c3ccccc3n(CCCC(F)(F)F)c2)cc(c1)C	9.4
35	FC(F)(F)CCCn1cc(C(=O)[C@H](CCC)CC)c2ccccc21	9.4
36	FC(F)(F)CCCn1cc(c2ccccc21)C(=O)c3ccoc3C	9.4
37	FC(F)(F)CCCn1cc(C(=O)[C@H]2CCOC2)c3ccccc31	9.4
38	FC(F)(F)CCCn1cc(c2ccccc21)C(=O)c3cc[nH]c3C	9.4
39	FC(F)(F)CCCn1cc(-n2c3c(nn2)CCCC3)c4ccccc41	9.4
40	FC(F)(F)CCCn1cc(c2ccccc21)-c3c4cc(O)ccc4on3	9.4
41	FC(F)(F)CCCn1cc(c2ccccc21)-c3c4cc(F)ccc4on3	9.4
42	FC(F)(F)CCCn1cc(c2ccccc21)C(=O)c3c(oc(n3)C)C	9.4
43	Clc1c(cccc1C(=O)c2c3ccccc3n(CCCC(F)(F)F)c2)C	9.4
44	FC(F)(F)CCCn1cc(c2ccccc21)C(=O)c3c([nH]c(c3)C)C	9.4
45	FC(F)(F)CCCn1cc(C(=O)[C@@H](CCC)C#N)c2ccccc21	9.3
46	FC(F)(F)CCCn1cc(c2ccccc21)C(=O)C3=CSCCO3	9.3
47	FC(F)(F)CCCn1cc(c2ccccc21)C(=O)[C@H](SCC)C(C)C	9.3
48	FC(F)(F)CCCn1cc(c2ccccc21)-c3c(C)c(no3)C(F)(F)F	9.3
49	FC(F)(F)CCCn1cc(c2ccccc21)C(=O)c3cc(ccc3SC)C	9.3
50	FC(F)(F)CCCn1cc(c2ccccc21)C(=O)c3cc(F)ccc3OC	9.3
51	FC(F)(F)CCCn1cc(C(=O)C2C3CC4CC(CC2C4)C3)c5ccccc51	9.3
52	FC(F)(F)CCCn1cc(c2ccccc21)C(=O)c3ccncc3C	9.3
53	FC(F)(F)CCCn1cc(c2ccccc21)C(=O)[C@H](SC)CC	9.3
54	FC(F)(F)CCCn1cc(C(=O)C2[C@H]3CCCC[C@@H]23)c4ccccc41	9.3
55	FC(F)(F)CCCn1cc(C(=O)C(C2CC2)C3CC3)c4ccccc41	9.3
56	FC(F)(F)CCCn1c2ccccc2c(C(=O)C3(CCCC3)CC)c1	9.3
57	FC(F)(F)CCCn1cc(c2ccccc21)C(=O)c3cccc(F)c3	9.3
58	FC(F)(F)CCCn1cc(c2ccccc21)-c3c4c(on3)ccc(c4)C	9.3
59	FC(F)(F)CCCn1cc(C(=O)[C@@H]2[C@](C2)(CC)C)c3ccccc31	9.3
60	FC(F)(F)CCCn1cc(c2ccccc21)C(=O)c3c(cc(o3)C)C	9.3
61	Clc1cn[nH]c1C(=O)c2c3ccccc3n(CCCC(F)(F)F)c2	9.3
62	FC(F)(F)CCCn1cc(-c2c3c(on2)CCC3)c4ccccc41	9.3
63	FC(F)(F)CCCn1cc(C(=O)[C@H]2CCCCO2)c3ccccc31	9.3

64	FC(F)(F)CCCn1c2ccccc2c(C(=O)C(C(F)(F)F)C(F)(F)F)c1	9.3
65	FC(F)(F)CCCn1cc(c2ccccc21)-c3c4ccc(O)cc4on3	9.3
66	FC(F)(F)CCCn1cc(C(=O)N2CCC[C@H]([C@H]2C)C)c3ccccc31	9.3
67	Clc1ccccc1C(=O)c2c3ccccc3n(CCCC(F)(F)F)c2	9.3
68	Clc1ccncc1C(=O)c2c3ccccc3n(CCCC(F)(F)F)c2	9.3
69	FC(F)(F)CCCn1cc(c2ccccc21)C(=O)NC[C@@H](CC)C	9.2
70	FC(F)(F)CCCn1cc(c2ccccc21)C(=O)CCCCC#C	9.2
71	FC(F)(F)CCCn1cc(c2ccccc21)C(=O)c3c(C)ccs3	9.2
72	FC(F)(F)CCCn1cc(C(=O)[C@@H](c2ccccc2)C)c3ccccc31	9.2
73	FC(F)(F)CCCn1cc(c2ccccc21)C(=O)c3cnc(s3)C	9.2
74	FC(F)(F)CCCn1c2ccccc2c(C(=O)[C@@H](COC)C)c1	9.2
75	FC(F)(F)CCCn1cc(c2ccccc21)C(=O)c3cncs3	9.2
76	FC(F)(F)[C@H]1CCCC[C@H]1C(=O)c2c3ccccc3n(CCCC(F)(F)F)c2	9.2
77	FC(F)(F)CCCn1cc(c2ccccc21)C(=O)[C@H](OC)c3ccccc3	9.2
78	FC(F)(F)CCCn1cc(c2ccccc21)C(=O)c3c(F)cc(F)cc3F	9.2
79	FC(F)(F)CCCn1cc(C(=O)[C@@H]2C(C2)(C)C)c3ccccc31	9.2
80	Brc1ccsc1C(=O)c2c3ccccc3n(CCCC(F)(F)F)c2	9.2
81	Fc1cc(C(=O)c2c3ccccc3n(CCCC(F)(F)F)c2)cc(c1)C	9.2
82	FC(F)(F)CCCn1cc(c2ccccc21)C(=O)c3c(ccs3)C#N	9.2
83	Clc1c(F)cccc1C(=O)c2cn(CCCC(F)(F)F)c3ccccc32	9.2
84	FC(F)(F)CCCn1cc(C(=O)[C@H]2CCCS2)c3ccccc31	9.2
85	Clc1cc(C)ccc1C(=O)c2c3ccccc3n(CCCC(F)(F)F)c2	9.2
86	FC(F)(F)CCCn1cc(c2ccccc21)C(=O)c3c(SC)ccs3	9.2
87	FC(F)(F)CCCn1cc(c2ccccc21)C(=O)c3c(CC)ccs3	9.2
88	Clc1cc(C(=O)c2c3ccccc3n(CCCC(F)(F)F)c2)c(Cl)s1	9.2
89	FC(F)(F)CCCn1cc(C(=O)C2=CCCCCC2)c3ccccc31	9.2
90	FC(F)(F)CCCn1c2ccccc2c(C(=O)C3(CCCC3)c4cccs4)c1	9.2
91	FC(F)(F)CCCn1cc(c2ccccc21)-c3c(C)cno3	9.2
92	FC(F)(F)CCCn1cc(C(=O)[C@H]2CSCCS2)c3ccccc31	9.2
93	Cl[C@@H](C(=O)c1c2ccccc2n(CCCC(F)(F)F)c1)c3ccccc3	9.2
94	FC(F)(C(F)(F)C(=O)c1c2ccccc2n(CCCC(F)(F)F)c1)C(F)(F)F	9.2
95	Clc1cc(F)c(cc1C(=O)c2c3ccccc3n(CCCC(F)(F)F)c2)C	9.2
96	FC(F)(F)CCCn1cc(c2ccccc21)-c3c4ccc(F)cc4on3	9.2
97	FC(F)(F)CCCn1cc(c2ccccc21)C(=O)c3ccc(F)c(N)c3	9.1
98	FC(F)(F)CCCn1cc(c2ccccc21)C(=O)c3nccs3	9.1
99	FC(F)(F)CCCn1cc(c2ccccc21)C(=O)c3cc[nH]c3	9.1
100	FC(F)(C(=O)c1c2ccccc2n(CCCC(F)(F)F)c1)C(F)F	9.1
101	FC(F)(F)CCCn1c2ccccc2c(C(=O)N3CC[C@H](C3)C)c1	9.1
102	FC(F)(F)CCCn1cc(c2ccccc21)C(=O)c3cc(c(s3)C)C	9.1
103	FC(F)(F)CCCn1cc(c2ccccc21)C(=O)c3ccccc3C(OC)=O	9.1
104	FC1(F)CCC(CC1)C(=O)c2c3ccccc3n(CCCC(F)(F)F)c2	9.1
105	FC(F)(F)CCCn1cc(C(=O)C2(CC2)c3cccc(c3)C)c4ccccc41	9.1

106	FC1(F)CC(C1)C(=O)c2c3ccccc3n(CCCC(F)(F)F)c2	9.1
107	Clc1cccc(C(=O)c2c3ccccc3n(CCCC(F)(F)F)c2)c1	9.1
108	FC(F)(F)CCCn1cc(c2ccccc21)C(=O)c3ccc(C)cc3	9.1
109	FC(F)(F)CCCn1cc(c2ccccc21)C(=O)[C@H](C(C)C)c3ccccc3	9.1
110	Clc1ccsc1C(=O)c2c3ccccc3n(CCCC(F)(F)F)c2	9.1
111	SC[C@@H](C(=O)c1c2ccccc2n(CCCC(F)(F)F)c1)C	9.1
112	FC(F)(F)CCCn1cc(c2ccccc21)C(=O)c3cc(F)ccc3[N+]([O-])=O	9.1
113	FC(F)(F)CCCn1cc(c2ccccc21)C(=O)c3csc(n3)C	9.1
114	FC(F)(F)CCCn1cc(C(=O)C2(CC2)c3ccccc3)c4ccccc41	9.1
115	FC1(F)C[C@@H]1C(=O)c2c3ccccc3n(CCCC(F)(F)F)c2	9.1
116	FC(F)(F)CCCn1cc(c2ccccc21)C(=O)c3ccccc3OCC	9.1
117	FC(F)(F)CCCn1cc(c2ccccc21)C(=O)c3c(sc(c3)C)C	9.1
118	FC(F)(F)CCCn1cc(c2ccccc21)C(=O)C3=CCCC3	9.1
119	FC(F)(F)CCCn1cc(c2ccccc21)C(=O)c3cccc4c3cc(o4)C	9.1
120	F[C@@H]1CCN(C1)C(=O)c2c3ccccc3n(CCCC(F)(F)F)c2	9.1
121	FC(F)(F)CCCn1c2ccccc2c(C(=O)C(CC(C)C)(C)C)c1	9.1
122	FC(F)(F)CCCn1cc(C(=O)C2CCSCC2)c3ccccc31	9.1
123	Br[C@@H](C(=O)c1c2ccccc2n(CCCC(F)(F)F)c1)C(C)(C)C	9.1
124	FC(F)(F)CCCn1cc(c2ccccc21)-c3c4ccccc4no3	9.1
125	FC(F)(F)CCCn1cc(C(=O)C2CC(=O)C2)c3ccccc31	9.1
126	FC(F)(F)CCCn1cc(c2ccccc21)C(=O)c3nc(C)cs3	9.1
127	Clc1ccc2c(onc2-c3c4ccccc4n(CCCC(F)(F)F)c3)c1	9.1
128	FC(F)(F)CCCn1cc(C(=O)N2CCS[C@@H]2CCC)c3ccccc31	9.1
129	FC(F)(F)CCCn1cc(-n2c3ccc(cc3nn2)C)c4ccccc41	9.1
130	FC(F)(F)CCCn1cc(C(=O)[C@H](CC)C)c2ccccc21	9.1
131	FC(F)(F)CCCn1cc(C(=O)[C@@H]2CCCC[C@@H]2C)c3ccccc31	9.1
132	FC(F)(F)CCCn1cc(c2ccccc21)C(=O)N[C@H](C(C)C)C	9
133	FC(F)(F)CCCn1cc(C(=O)[C@@H]([S@@](=O)CC)C)c2ccccc21	9
134	FC(F)(F)CCCn1cc(C(=O)C2=COCCC2)c3ccccc31	9
135	FC(F)(F)CCCn1cc(-n2c3ccccc3nn2)c4ccccc41	9
136	Brc1ccoc1C(=O)c2c3ccccc3n(CCCC(F)(F)F)c2	9
137	FC(F)(F)CCCn1c2ccccc2c(C(=O)C(C(F)(F)F)(C(F)(F)F)C)c1	9
138	FC(F)(F)CCCn1cc(c2ccccc21)C(=O)c3csnc3C	9
139	ClC(Cl)=C[C@H]1C([C@H]1C(=O)c2c3ccccc3n(CCCC(F)(F)F)c2)(C)C	9
140	FC(F)(F)CCCn1cc(C(=O)[C@@H](C(C)C)C)c2ccccc21	9
141	FC(F)(F)CCCn1cc(c2ccccc21)C(=O)c3c(C)cco3	9
142	FC(F)(F)CCCn1cc(c2ccccc21)-c3c(snn3)C	9
143	FC(F)(F)CCCn1cc(c2ccccc21)C(=O)c3cccc(c3F)C	9
144	FC(F)(F)CCCn1cc(C(=O)[C@@H]2[C@@H](CCC2)C#N)c3ccccc31	9
145	Brc1csc(C(=O)c2c3ccccc3n(CCCC(F)(F)F)c2)c1	9
146	Brc1cc(c(o1)C(=O)c2c3ccccc3n(CCCC(F)(F)F)c2)C	9
147	FC(F)(F)CCCn1cc(c2ccccc21)C(=O)c3ccc(o3)N	9

148	Clc1cc(F)cc(C(=O)c2c3ccccc3n(CCCC(F)(F)F)c2)c1	9
149	FC(F)(F)CCCn1cc(C(=O)[C@@H]2CC=CCC2)c3ccccc31	9
150	FC(F)(F)CCCn1c2ccccc2c(C(=O)C34C5C[C@@H](C3)C[C@@H](C4)C5)c1	9
151	FC(F)(F)CCCn1cc(c2ccccc21)C(=O)c3cc(F)ccc3N	9
152	Clc1ccc(F)cc1C(=O)c2c3ccccc3n(CCCC(F)(F)F)c2	9
153	FC(F)(F)CCCn1cc(c2ccccc21)C(=O)[C@H](OC)C	9
154	FC(F)(F)CCCn1cc(c2ccccc21)C(=O)[C@H](OC)CC	9
155	FC(F)(F)CCCn1cc(c2ccccc21)-c3cc(C)cnn3	9
156	FC(F)(F)CCCn1cc(c2ccccc21)C(=O)NCCC(C)C	9
157	FC(F)(F)CCCn1cc(C(=O)C2CCCC2)c3ccccc31	9
158	FC(F)(F)CCCn1cc(c2ccccc21)-c3c(C)con3	9
159	FC(F)(F)CCCn1cc(C(=O)[C@@H](c2ccsc2)C)c3ccccc31	9
160	FC(F)(F)CCCn1cc(C(=O)C2C(C2(C)C)(C)C)c3ccccc31	9
161	FC(F)(F)CCCn1cc(C(=O)[C@H]2C[C@@H]3CC[C@H]2C3)c4ccccc41	9
162	FC(F)(F)CCCn1cc(c2ccccc21)C(=O)c3ccccc3CC	9
163	FC(F)(F)CCCn1cc(C(=O)C(CC)CC)c2ccccc21	9
164	FC(F)(F)CCCn1cc(C(=O)[C@@H](CC#C)C)c2ccccc21	8.9
165	FC(F)(F)CCCn1cc(c2ccccc21)C(=O)c3ccc(F)cc3C	8.9
166	Clc1ccc(o1)C(=O)c2c3ccccc3n(CCCC(F)(F)F)c2	8.9
167	FC(F)(F)CCCn1cc(c2ccccc21)C(=O)Cc3cc[nH]c3	8.9
168	FC(F)(F)CCCn1cc(-n2c(C(C)C)cnn2)c3ccccc31	8.9
169	FC(F)(F)CCCn1cc(C(=O)C2CCC2)c3ccccc31	8.9
170	FC(F)(F)CCCn1cc(c2ccccc21)C(=O)c3ccc(O)c(F)c3	8.9
171	FC(F)(F)CCCn1cc(c2ccccc21)C(=O)c3ccc(N)cc3F	8.9
172	Clc1ccc(s1)C(=O)c2c3ccccc3n(CCCC(F)(F)F)c2	8.9
173	Clc1ccc2c(n(nn2)-c3c4ccccc4n(CCCC(F)(F)F)c3)c1	8.9
174	FC(F)(F)CCCn1cc(c2ccccc21)C(=O)C(CC)=C	8.9
175	FC(F)(F)CCCn1cc(c2ccccc21)C(=O)NCC/C=C/C	8.9
176	FC(F)(F)CCCn1cc(c2ccccc21)C(=O)NC(CC)(CC)C#N	8.9
177	ClC1(Cl)[C@@H]([C@@]1(CC)C(=O)c2c3ccccc3n(CCCC(F)(F)F)c2)C	8.9
178	FC(F)(F)CCCn1cc(c2ccccc21)C(=O)c3c(F)cccn3	8.9
179	Sc1ccccc1C(=O)c2c3ccccc3n(CCCC(F)(F)F)c2	8.9
180	Clc1cnccc1C(=O)c2c3ccccc3n(CCCC(F)(F)F)c2	8.9
181	F[C@@H](C(=O)c1c2ccccc2n(CCCC(F)(F)F)c1)C	8.9
182	FC(F)(F)CCCn1cc(-c2c3c(on2)CC[C@@H](C3)C)c4ccccc41	8.9
183	FC(F)(F)CCCn1cc(c2ccccc21)-c3c4ccccc4cnn3	8.9
184	FC(F)(F)CCCn1cc(c2ccccc21)C(=O)/C(C)=C/C	8.9
185	FC(F)(F)CCCn1cc(C(=O)[C@@H]2CCC[C@@H](O)C2)c3ccccc31	8.9
186	FC(F)CN(C1CC1)C(=O)c2c3ccccc3n(CCCC(F)(F)F)c2	8.9
187	FC(F)(F)CCCn1c2ccccc2c(C(=O)[C@](O)(C(F)(F)F)C)c1	8.9
188	FC(F)(F)CCCn1cc(c2ccccc21)C(=O)[C@H]3[C@H](CCO3)C	8.9
189	FC(F)(F)CCCn1cc(c2ccccc21)C(=O)c3coc(n3)C	8.9

190	FC(F)(F)CCCn1cc(c2ccccc21)C(=O)NCCCC	8.9
191	FC(F)(F)CCCn1cc(C(=O)[C@H]2CCSC2)c3ccccc31	8.9
192	ClC1(Cl)[C@](C1)(C(=O)c2c3ccccc3n(CCCC(F)(F)F)c2)C	8.9
193	FC(F)(F)CCCn1cc(C(=O)[C@H](C(C)C)CC)c2ccccc21	8.9
194	FC(F)(F)CCCn1cc(-c2c3c(on2)CCCC3)c4ccccc41	8.9
195	FC(F)(F)CCCn1cc(C(=O)C2CCCCCCC2)c3ccccc31	8.9
196	FC(F)(F)CCCn1cc(c2ccccc21)C(=O)C3=CCCCO3	8.9
197	FC(F)(F)CCCn1cc(c2ccccc21)C(=O)[C@@H](CC)C#N	8.9
198	FC(F)(F)CCCn1cc(c2ccccc21)C(=O)c3c(ocn3)C	8.9
199	FC(F)(F)CCCn1cc(c2ccccc21)C(=O)C3=C(OCCS3)C	8.9
200	FC(F)(F)CCCn1cc(C(=O)N2[C@@H](CC[C@@H]2C)C)c3ccccc31	8.9
201	FC(F)(F)CCCn1cc(c2ccccc21)C(=O)c3ccc(s3)C	8.9
202	FC(F)(F)CCCn1cc(c2ccccc21)C(=O)C3=COCC3	8.9
203	FC(F)(F)CCCn1cc(c2ccccc21)C(=O)Cc3cccs3	8.9
204	FC(F)(F)CCCn1cc(c2ccccc21)C(OC(CC)CC)=O	8.8
205	FC(F)(F)CCCn1cc(C(=O)C2(CC2)C#N)c3ccccc31	8.8
206	FC(F)(F)CCCn1c2ccccc2c(C(=O)C3(CCC3)C#N)c1	8.8
207	FC(F)(F)CCCn1cc(C(=O)[C@H]2CCC[C@@H](C2)C)c3ccccc31	8.8
208	FC(F)(F)CCCn1cc(c2ccccc21)C(=O)c3cccc(c3)C	8.8
209	FC(F)(F)CCCn1cc(c2ccccc21)C(=O)c3ccc(F)cc3	8.8
210	FC(F)(F)CCCn1cc(c2ccccc21)C(=O)c3ccoc3	8.8
211	FC(F)(F)CCCn1cc(c2ccccc21)C(=O)c3ccc(O)cc3F	8.8
212	FC(F)(F)CCCn1cc(c2ccccc21)C(=O)/C=C/C(C)(C)C	8.8
213	FC(F)(F)CCCn1cc(-n2c3ccc(F)cc3nn2)c4ccccc41	8.8
214	FC(F)(F)CCCn1cc(C(=O)[C@@H]2CC[C@@H](O2)C)c3ccccc31	8.8
215	Brc1ccc(s1)C(=O)c2c3ccccc3n(CCCC(F)(F)F)c2	8.8
216	FC(F)(F)CCCn1cc(c2ccccc21)C(=O)C3=CCCCC3	8.8
217	S[C@@H]([C@@H](CC)C)C(=O)c1c2ccccc2n(CCCC(F)(F)F)c1	8.8
218	BrC(Br)C(=O)c1c2ccccc2n(CCCC(F)(F)F)c1	8.8
219	FC(F)(F)CCCn1cc(C(=O)[C@H]2[C@H](C2)C(OC)=O)c3ccccc31	8.8
220	FC(F)(F)CCCn1cc(c2ccccc21)C(=O)c3cc4ccoc4s3	8.8
221	FC(F)(F)CCCn1cc(C(=O)[C@@H]2CCO[C@H]2C)c3ccccc31	8.8
222	FC(F)(F)CCCn1cc(c2ccccc21)C(=O)c3ccccc3	8.8
223	FC(F)(F)CCCn1c2ccccc2c(C(=O)C3(CCC3)COC)c1	8.8
224	SC(C(=O)c1c2ccccc2n(CCCC(F)(F)F)c1)(C)C	8.8
225	FC(F)(F)CCCn1cc(c2ccccc21)C(=O)c3cscn3	8.8
226	S[C@@H](C(=O)c1c2ccccc2n(CCCC(F)(F)F)c1)C(C)C	8.8
227	FC(F)(F)CCCn1cc(c2ccccc21)C(=O)CCC=O	8.8
228	FC(F)(F)CCCn1c2ccccc2c(C(=O)N3CC=CC3)c1	8.8
229	FC(F)(F)CCCn1cc(c2ccccc21)-c3cc(on3)C	8.8
230	FC(F)(F)CCCn1cc(C(=O)[C@@H](C2CC2)C)c3ccccc31	8.8
231	Clc1ccc(Cl)c(C(=O)c2c3ccccc3n(CCCC(F)(F)F)c2)c1	8.8

232	ClC(Cl)C(=O)c1c2ccccc2n(CCCC(F)(F)F)c1	8.8
233	FC(F)(F)CCCn1cc(c2ccccc21)C(SC(C)C)=O	8.8
234	FC(F)(F)CCCn1cc(c2ccccc21)C(=O)c3c(n[nH]n3)C	8.8
235	FC(F)(F)C1(CC1)C(=O)c2c3ccccc3n(CCCC(F)(F)F)c2	8.8
236	FC(F)(F)CCCn1cc(C(=O)[C@@]2(CCCS2)C)c3ccccc31	8.8
237	FC(F)(F)CCCn1cc(c2ccccc21)C(=O)c3ccc(o3)C	8.8
238	FC(F)(F)CCCn1cc(c2ccccc21)C(=O)c3ccc(F)cc3F	8.8
239	FC(F)(F)CCCn1cc(c2ccccc21)-c3cc(no3)CC	8.8
240	FC(F)(F)CCCn1cc(c2ccccc21)C(=O)c3c([N+]([O-])=O)cn[nH]3	8.8
241	FC(F)(F)CCCn1cc(C(OC(C(C)C)C(C)C)=O)c2ccccc21	8.8
242	FC(F)(F)CCCn1cc(c2ccccc21)C(=O)C(C)=C	8.8
243	Brc1cc(C(=O)c2c3ccccc3n(CCCC(F)(F)F)c2)cs1	8.8
244	FC(F)(F)CCCn1cc(c2ccccc21)C(=O)c3cc(C)cs3	8.8
245	S=C(N)[C@H](CCC)C(=O)c1c2ccccc2n(CCCC(F)(F)F)c1	8.7
246	FC(F)(F)CCCn1cc(c2ccccc21)C(=O)c3c(C)csc3	8.7
247	FC(F)(F)CCCn1cc(c2ccccc21)C(=O)C(CCCC)=C	8.7
248	FC(F)(F)CCCn1cc(C(=O)C[C@H](C(C)C)CC)c2ccccc21	8.7
249	FC(F)(F)CCCn1c2ccccc2c(C(=O)[C@@H](CSC)C)c1	8.7
250	FC(F)(F)CCCn1cc(C(=O)[C@@H](c2cccs2)C)c3ccccc31	8.7
251	FC(F)(F)CCCn1c2ccccc2c(C(=O)N3CCCC3)c1	8.7
252	Clc1nnc(s1)-c2c3ccccc3n(CCCC(F)(F)F)c2	8.7
253	FC(F)(F)CCCn1c2ccccc2c(C(=O)C(C(F)(F)F)(C)C)c1	8.7
254	FC(F)(F)CCCn1cc(c2ccccc21)C(=O)c3ccncc3CC	8.7
255	FC(F)(F)CCCn1cc(c2ccccc21)C(=O)c3c(oc(c3)C)C	8.7
256	Clc1c(scn1)C(=O)c2c3ccccc3n(CCCC(F)(F)F)c2	8.7
257	FC(F)(F)CCCn1cc(S(=O)(=O)CC=C)c2ccccc21	8.7
258	FC(F)(F)CCCn1c2ccccc2c(C(=O)C3(CC3)C)c1	8.7
259	FC(F)(F)CCCn1cc(C(=O)[C@@H]2CC32CCC3)c4ccccc41	8.7
260	FC(F)(F)CCCn1cc(C(=O)C2[C@@H]3CCC[C@H]23)c4ccccc41	8.7
261	FC(F)(F)CCCn1cc(c2ccccc21)C(=O)C3=C(OCCO3)C	8.7
262	FC(F)(F)CCCn1cc(C(=O)[C@@H](C2CCCC2)C)c3ccccc31	8.7
263	FC(F)(F)CCCn1cc(C(=O)C2(CC2)c3ccc(F)cc3)c4ccccc41	8.7
264	FC(F)(F)CCCn1cc(C(=O)[C@@H]2[C@@H]([N+]([O-])=O)C2)c3ccccc31	8.7
265	Clc1cc(F)ccc1C(=O)c2c3ccccc3n(CCCC(F)(F)F)c2	8.7
266	FC(F)(F)CCCn1cc(C(=O)C2(CCC2)c3cccc(F)c3)c4ccccc41	8.7
267	FC(F)(F)CCCn1cc(c2ccccc21)C(=O)c3c(cc([nH]3)C)C	8.7
268	Cl[C@@H](C(=O)c1c2ccccc2n(CCCC(F)(F)F)c1)C	8.7
269	FC(F)(F)CCCn1c2ccccc2c(C(=O)C3(CCCC3)C)c1	8.7
270	FC(F)(F)CCCn1c2ccccc2c(C(=O)C(CO)(C)C)c1	8.6
271	ClC(C(=O)c1c2ccccc2n(CCCC(F)(F)F)c1)=C	8.6
272	FC(F)(F)CCCn1cc(C(=O)C(O)(CC)CC)c2ccccc21	8.6
273	FC(F)(F)CCCn1cc(c2ccccc21)C(=O)[C@@H]3c4ccccc4C3	8.6

274	FC(F)(F)CCCn1cc(c2ccccc21)C(=O)CS(=O)(=O)CC#C	8.6
275	FC(F)(F)CCCn1cc(c2ccccc21)-c3cc(C)c(nn3)C	8.6
276	FC(F)(F)CCCn1cc(C(=O)C2CCCCC2)c3ccccc31	8.6
277	FC(F)(F)CCCn1cc(c2ccccc21)C(SC)=O	8.6
278	FC(F)(F)CCCn1cc(C(=O)C(C)C)c2ccccc21	8.6
279	FC(F)(F)CCCn1cc(c2ccccc21)C(SCCC)=O	8.6
280	FC(F)(F)CCCn1cc(c2ccccc21)C(=O)c3cccc(OC)c3F	8.6
281	FC(F)(F)CCCn1cc(c2ccccc21)C(=O)c3ccccc3SC	8.6
282	FC(F)(F)CCCn1cc(c2ccccc21)C(=O)c3ccccc3C#N	8.6
283	FC(F)(F)CCCn1cc(c2ccccc21)C(=O)c3c(oc(c3)C)C(F)(F)F	8.6
284	FC(F)(F)CCCn1c2ccccc2c(C(=O)C(CC)(C)C)c1	8.6
285	Br[C@H](C(=O)c1c2ccccc2n(CCCC(F)(F)F)c1)C	8.6
286	FC(F)(F)CCCn1cc(C(=O)[C@@H]2C[C@@H]2C3CC3)c4ccccc41	8.6
287	FC(F)(F)CCCn1cc(c2ccccc21)C(=O)[C@H](O)C(C)(C)C	8.6
288	Clc1ccc(s1)[C@H](C(=O)c2c3ccccc3n(CCCC(F)(F)F)c2)C	8.6
289	FC(C(=O)c1c2ccccc2n(CCCC(F)(F)F)c1)(C)C	8.6
290	FC(F)(F)CCCn1cc(c2ccccc21)C(SCC)=O	8.6
291	FC(F)(F)CCCn1cc(c2ccccc21)-c3nnc4n3ccs4	8.6
292	FC(F)(F)CCCn1cc(c2ccccc21)C(=O)c3cocn3	8.6
293	FC(F)(F)CCCn1cc(c2ccccc21)C(=O)CC3(CCCCC3)C	8.6
294	FC(F)(F)CCCn1cc(C(=O)[C@@H](C2CCOCC2)C)c3ccccc31	8.5
295	FC(F)(F)CCCn1cc(c2ccccc21)C=3CCON3	8.5
296	FC(F)(F)CCCn1cc(c2ccccc21)C(SC(C)(C)C)=O	8.5
297	FC(F)(F)CCCn1cc(c2ccccc21)C(=O)/C=C(/C(C)C)C	8.5
298	FC(F)(F)CCCn1cc(C(=O)CC(C2CC2)C3CC3)c4ccccc41	8.5
299	ClC(F)(F)C(=O)c1c2ccccc2n(CCCC(F)(F)F)c1	8.5
300	FC(F)(F)CCCn1c2ccccc2c(S(=O)(=O)C(CC)CC)c1	8.5
301	ClC1(Cl)C[C@H]1C(=O)c2c3ccccc3n(CCCC(F)(F)F)c2	8.5
302	FC(F)(F)CCCn1cc(c2ccccc21)C(=O)c3ccsc3	8.5
303	Cl[C@H](F)C(S(=O)(=O)c1c2ccccc2n(CCCC(F)(F)F)c1)(F)F	8.5
304	FC(F)(F)CCCn1cc(C(=O)C2CCC(O)CC2)c3ccccc31	8.5
305	FC(F)(F)CCCn1cc(C(=O)C2CC=CC2)c3ccccc31	8.5
306	FC(F)(F)CCCn1cc(c2ccccc21)C(=O)[C@H]([N+](C)C)c3cnccc3	8.5
307	FC(F)(F)CCCn1cc(S(=O)(=O)CC(C)=C)c2ccccc21	8.5
308	FC(F)(F)CCCn1cc(c2ccccc21)C(=O)/C=C/CCCC	8.5
309	FC(F)(F)CCCn1cc(c2ccccc21)C(=O)c3cncc(F)c3	8.5
310	FC(F)(F)CCCn1cc(C(=O)[C@H](CC#N)C)c2ccccc21	8.5
311	FC(F)(F)CCCn1cc(c2ccccc21)C(=O)CN#C	8.5
312	FC(F)(F)CCCn1cc(C(=O)C2(O)CCC2)c3ccccc31	8.5
313	FC(F)(F)CCCn1cc(C(=O)C2CC2)c3ccccc31	8.5
314	FC(F)(F)CCCn1cc(c2ccccc21)C(=O)c3csc(c3)C	8.5
315	FC(F)(F)CCCn1c2ccccc2c(C(=O)[C@@H](S(=O)(=O)C(C)C)C)c1	8.5

316	BrC(C(=O)c1c2ccccc2n(CCCC(F)(F)F)c1)(C)C	8.5
317	ClC1(CC(C1)(C(=O)c2c3ccccc3n(CCCC(F)(F)F)c2)C)C	8.5
318	FC(F)(F)CCCn1cc(c2ccccc21)C(=O)CC(CC)CC	8.5
319	FC(F)(F)CCCn1c2ccccc2c(C(=O)C3(CCCCC3)C)c1	8.5
320	SCc1cc(on1)-c2c3ccccc3n(CCCC(F)(F)F)c2	8.5
321	FC(F)(F)CCCn1cc(c2ccccc21)C(=O)c3ccco3	8.5
322	FC(F)(F)CCCn1cc(C(=O)C2CCOCC2)c3ccccc31	8.5
323	FC(F)(F)CCCn1cc(c2ccccc21)C(OCC3CC3)=O	8.5
324	FC(F)(F)CCCn1cc(c2ccccc21)C(=O)C/C=C/CC	8.5
325	FC(F)(F)CCCn1c2ccccc2c(C(=O)N3CCSC3)c1	8.5
326	FC(F)(F)CCCn1cc(c2ccccc21)C(=O)N[C@@H](C3CCC3)C	8.4
327	S=C(N)[C@@H](C(=O)c1c2ccccc2n(CCCC(F)(F)F)c1)C(C)C	8.4
328	FC(F)(F)CCCn1cc(c2ccccc21)C(=O)CC(C)=C	8.4
329	FCCCS(=O)(=O)c1c2ccccc2n(CCCC(F)(F)F)c1	8.4
330	FC(F)(F)CCCn1cc(c2ccccc21)C(=O)C(C(C)C)=C	8.4
331	FC(F)(F)CCCn1c2ccccc2c(C(=O)C3(CCCCCC3)C#N)c1	8.4
332	FC(F)(F)CCCn1cc(c2ccccc21)-c3ccno3	8.4
333	FC(F)(F)CCCn1cc(c2ccccc21)-c3cc(no3)C4CC4	8.4
334	FC(F)(F)CCCn1cc(c2ccccc21)C(=O)c3ccc([nH]3)C	8.4
335	FC(F)(F)CCCn1cc(c2ccccc21)-c3ccon3	8.4
336	FC(F)(F)CCCn1cc(C(=O)[C@@H]2CCCO2)c3ccccc31	8.4
337	FC(F)(F)CCCn1cc(c2ccccc21)C(=O)[C@@H](SCC)C	8.4
338	FC(F)(F)CCCn1cc(c2ccccc21)C(=O)/C(C)=C/CO	8.4
339	FC(F)(F)CCCn1cc(c2ccccc21)-c3cc(no3)C	8.4
340	FC(F)(F)CCCn1cc(c2ccccc21)C(SCF)=O	8.4
341	S=C(N)C[C@H](C(=O)c1c2ccccc2n(CCCC(F)(F)F)c1)C	8.4
342	FC(F)(F)CCCn1cc(c2ccccc21)C(=O)C/C=C/C	8.4
343	Br[C@@H](C(=O)c1c2ccccc2n(CCCC(F)(F)F)c1)C(C)C	8.4
344	BrC(C(=O)c1c2ccccc2n(CCCC(F)(F)F)c1)=C	8.4
345	S[C@H](C(=O)c1c2ccccc2n(CCCC(F)(F)F)c1)C	8.4
346	FC(F)(F)CCCn1cc(c2ccccc21)C(=O)C(F)=C(C)C	8.4
347	FC(F)(F)CCCn1cc(c2ccccc21)C(=O)/C=C(/CC)C	8.4
348	FC(F)(F)CCCn1cc(c2ccccc21)C(=O)c3ccncc3F	8.4
349	FC(F)(F)CCCn1cc([S@@](=O)CC)c2ccccc21	8.4
350	FC(F)(F)CCCn1cc(CC2C(C2(C)C)(C)C)c3ccccc31	8.4
351	FC(F)(F)CCCn1cc(C(=O)C(OC)(C)C)c2ccccc21	8.4
352	FC(F)(F)CCCn1cc(c2ccccc21)C(=O)NCCC	8.4
353	FC(F)(F)CCCn1cc(c2ccccc21)C(=O)c3cccc(F)c3F	8.4
354	FC(F)(F)CCCn1cc(c2ccccc21)C(=O)c3cccs3	8.4
355	FC(F)(F)CCCn1cc(c2ccccc21)C(=O)CC[C@@H](O)C	8.4
356	FC(F)(F)CCCn1c2ccccc2c(C(=O)C3CC(C3)(C)C)c1	8.3
357	FC(F)(F)CCCn1cc(c2ccccc21)C(=O)/C=C/CCC	8.3

358	FC(F)(F)CCCn1cc(c2ccccc21)C(OC(C)C)=O	8.3
359	FC(F)(F)CCCn1cc(c2ccccc21)C(OC3(CC3)C)=O	8.3
360	FC(F)(F)CCCn1cc(c2ccccc21)C(=O)c3ccccc3F	8.3
361	FC(F)(F)CCCn1cc(C(=O)[C@@H](n2cncn2)C)c3ccccc31	8.3
362	FC(F)(F)CCCn1cc(c2ccccc21)C(=O)NCC(F)F	8.3
363	ClC(Cl)(Cl)C(=O)c1c2ccccc2n(CCCC(F)(F)F)c1	8.3
364	FC(F)(F)CCCn1cc(c2ccccc21)C(=O)CCCCC	8.3
365	FC(F)(C(=O)c1c2ccccc2n(CCCC(F)(F)F)c1)C(F)(F)F	8.3
366	FC(F)(F)CCCn1cc(c2ccccc21)C(=O)N[C@@H](CCC)C	8.3
367	FC(F)(F)CCCn1cc(c2ccccc21)C(=O)C[C@H](SC)C	8.3
368	FC(F)(F)CCCn1cc(c2ccccc21)C(=O)C=C(C3CC3)C4CC4	8.3
369	FC(F)(F)CCCn1cc(c2ccccc21)C(=O)CC#CC	8.3
370	FC(F)(F)CCCn1cc(C(=O)[C@@H]2[C@@H](C2)C)c3ccccc31	8.3
371	FC(F)(F)CCCn1cc(c2ccccc21)C(=O)CSCCC	8.3
372	FC(F)(F)CCCn1cc(c2ccccc21)C(=O)C3=C(OCCC3)C	8.3
373	FC(F)(F)CCCn1cc(C(=O)[C@H]2COCCC2)c3ccccc31	8.3
374	FC(F)(F)CCCn1cc(S(=O)(=O)CCCC)c2ccccc21	8.3
375	FC(F)(F)CCCn1cc(c2ccccc21)C(O[C@H](CC)C)=O	8.3
376	FC(F)(F)CCCn1cc(c2ccccc21)C(=O)C=C3CCC3	8.3
377	FC(F)(F)CCCn1cc(C(=O)[C@@H]([N+](C)C)[C@H](CC)C)c2ccccc21	8.2
378	FC(F)(F)CCCn1cc(c2ccccc21)C(=O)/C=C/C(C)C	8.2
379	FC(F)(F)CCCn1cc(c2ccccc21)C(=O)CC(C)C	8.2
380	FC(F)(F)CCCn1cc(c2ccccc21)C(=O)CC(C)(C)C	8.2
381	FC(F)(F)CCCn1cc(OC(C(F)(F)F)C(F)(F)F)c2ccccc21	8.2
382	FC(F)(F)CCCn1cc(C(=O)[C@@H]2C([C@H]2C=C(C)C)(C)C)c3ccccc31	8.2
383	FC(F)(F)CCCn1cc(C(=O)CC(C(C)C)C(C)C)c2ccccc21	8.2
384	FC(F)(F)CCCn1cc(c2ccccc21)C(=O)[C@@H](SC)C	8.2
385	FC(F)(F)CCCn1cc(c2ccccc21)C(=O)CCC#C	8.2
386	FC(F)(F)CCCn1cc(c2ccccc21)C(=O)c3coc(c3)C	8.2
387	FC(F)(F)CCCn1cc(c2ccccc21)C(=O)CC=C	8.2
388	Clc1ccc(nn1)-c2c3ccccc3n(CCCC(F)(F)F)c2	8.2
389	FC(F)(F)CS(=O)(=O)c1c2ccccc2n(CCCC(F)(F)F)c1	8.2
390	FC(F)(F)CCCn1c2ccccc2c(C(=O)C(C)(C)C)c1	8.2
391	FC(F)(F)CCCn1cc(c2ccccc21)C(=O)CCC(F)(F)F	8.2
392	FC(F)(F)CCCn1cc(c2ccccc21)C(=O)NOCC=C	8.2
393	FC(F)(F)CCCn1cc(c2ccccc21)C(=O)C[C@H](CCC)C	8.2
394	FC(F)(F)CCCn1cc(c2ccccc21)C(=O)NCC3CCC3	8.2
395	FC(F)(F)CCCn1cc(c2ccccc21)C(=O)NC3(CC3)C	8.2
396	FC(S(=O)(=O)c1c2ccccc2n(CCCC(F)(F)F)c1)(F)C(F)F	8.2
397	FC(F)(F)CCCn1cc(C(=O)[C@@H](n2cccn2)C)c3ccccc31	8.2
398	FC(F)(F)CCCn1cc(c2ccccc21)C(=O)c3ccns3	8.2
399	FC(F)(F)CCCn1cc(c2ccccc21)C(=O)c3cc(F)ccc3F	8.2

400	FC(F)(F)CCCn1cc(c2ccccc21)C([S@@](=O)CC)=O	8.2
401	FC(F)(F)CCCn1cc(C(=O)C[C@@H](C(C)C)C)c2ccccc21	8.2
402	FC(F)(F)CCCn1cc(c2ccccc21)C(=O)CC3CCCC3	8.2
403	FC(F)(F)CCCn1cc(c2ccccc21)C(=O)Cc3cccc(F)c3	8.2
404	FC(F)(F)CCCn1cc(c2ccccc21)C(=O)C[C@@H](CC)C	8.1
405	FC(F)(F)CCCn1cc(c2ccccc21)C(=O)[C@@H]([N+](C)C)C(C)C	8.1
406	FC(F)(F)CCCn1cc(c2ccccc21)C(=O)C3=CC[N+]CC3	8.1
407	FC(F)(F)CCCn1cc(c2ccccc21)C(O[C@H](C3CC3)C)=O	8.1
408	FC(F)(F)CCCn1cc(C(=O)CCC(C)C)c2ccccc21	8.1
409	FC(F)(F)CCCn1cc(c2ccccc21)C(=O)c3c(F)cc(F)cn3	8.1
410	FC(F)(F)CCCn1cc(c2ccccc21)C(=O)CCC=C	8.1
411	FC(F)(F)CCCn1cc(c2ccccc21)C(=O)/C=C/C=C/C	8.1
412	FC(F)(F)CCCn1cc(c2ccccc21)C(=O)CC3CC3	8.1
413	FC(F)(F)CCCn1cc(c2ccccc21)C(=O)CC3CCC3	8.1
414	FC(F)(F)CCCn1c2ccccc2c(C(=O)[C@@H](C[N+](C)C)C)c1	8.1
415	FC(F)(F)CCCn1cc(C(=O)CCC2CC2)c3ccccc31	8.1
416	FC(F)(F)CCCn1cc(c2ccccc21)C(=O)c3ccc[nH]3	8.1
417	FC(F)(F)CCCn1cc(c2ccccc21)C(=O)NCC	8.1
418	FC(F)(F)CCCn1cc([S@@](=O)CCC)c2ccccc21	8.1
419	FC(F)(F)CCCn1cc(c2ccccc21)C(=O)CCC	8.1
420	FC(F)(F)CCCn1cc(C(=O)NCC(C)C)c2ccccc21	8.1
421	FC(F)(F)CCCn1cc(c2ccccc21)C(=O)C(CCC)=C	8.1
422	FC(F)(F)CCCn1cc(c2ccccc21)C(=O)CCO[N+]([O-])=O	8.1
423	FC(F)(F)CCCn1cc(c2ccccc21)C(=O)C=C(C)C	8.1
424	Clc1cc(on1)-c2c3ccccc3n(CCCC(F)(F)F)c2	8.1
425	FC(F)(F)CCCn1cc(c2ccccc21)C(=O)NCCC=C	8.1
426	FC(F)(F)CCCn1cc(C(=O)C2=CC[C@H]([N+])C2)c3ccccc31	8.1
427	FC(F)(F)CCCn1cc(c2ccccc21)C(=O)[C@H]([N+](C)C)c3ccccc3	8.1
428	FC(F)(F)CCCn1cc(c2ccccc21)C(=O)/C=C(\C3CC3)C	8
429	FC(F)(F)CCCn1c2ccccc2c(C(=O)C(CCC)(C)C)c1	8
430	FC(F)(F)CCCn1cc(c2ccccc21)C(=O)C[C@@H](C(F)(F)F)C	8
431	FC(F)(F)CCCn1cc(c2ccccc21)C(=O)/C=C/CC	8
432	FC(F)(F)CCCn1cc(c2ccccc21)C(=O)CC	8
433	FC(F)(F)CCCn1cc(c2ccccc21)C(=O)CSC(F)(F)F	8
434	FC(F)(F)CCCn1cc(c2ccccc21)C(=O)c3cnsn3	8
435	FC(F)(F)CCCn1cc(C(=O)[C@@H]2CC[N+](C2)C)c3ccccc31	8
436	FC(F)(F)CCCn1cc(c2ccccc21)C(=O)c3c(ncs3)C	8
437	FC(F)(F)CCCn1cc(c2ccccc21)C(=O)C[C@@H](C3CC3)C	8
438	FC(F)(F)CCCn1cc(OCC2CC2)c3ccccc31	8
439	FC(F)(F)CCCn1cc(c2ccccc21)C(=O)CCC#N	8
440	FC(F)(F)CCCn1cc(c2ccccc21)C(=O)C=C(SC)SC	7.9
441	FC(F)(F)CCCn1c2ccccc2c(C(=O)[C@@]3(CCC[N+]3)C)c1	7.9

442	FC(F)(F)CCCn1cc(C(=O)C2=CCC[N+](C2)C)c3ccccc31	7.9
443	FC(F)(F)CCCn1c2ccccc2c(C(=O)C3(CCC3)C[N+])c1	7.9
444	FC(F)(F)CCCn1cc(c2ccccc21)C(=O)Cc3cc(F)c(F)cc3F	7.9
445	FC(F)(F)CCCn1cc(c2ccccc21)C(=O)CC3CCCCC3	7.9
446	FC(F)(F)CCCn1cc(c2ccccc21)C(=O)/C=C/SC	7.9
447	FC(F)(F)CCCn1c2ccccc2c(C(=O)[C@@]([N+])(C(F)(F)F)C)c1	7.9
448	FC(F)(F)CCCn1cc(S(=O)(=O)CC2CCC2)c3ccccc31	7.9
449	FC(F)(F)CCCn1cc(C(=O)[C@@H]2CC32CC[N+]CC3)c4ccccc41	7.9
450	FC(F)(F)CCCn1cc(C(=O)[C@H]([N+]C)CC)c2ccccc21	7.9
451	FC(F)(F)CCCn1cc(c2ccccc21)C(=O)N[C@@H](CC)C	7.9
452	FC(F)(F)CCCn1cc(c2ccccc21)C(=O)/C=C/C	7.9
453	FC(F)(F)CCCn1cc(c2ccccc21)C(=O)C=C	7.9
454	FC(F)(F)CCCn1cc(c2ccccc21)C(=O)Cc3c(nco3)C	7.9
455	FC(F)(F)CCCn1cc(OC[C@H]2CS2)c3ccccc31	7.9
456	F[C@@H]1C[C@H]1C(=O)c2c3ccccc3n(CCCC(F)(F)F)c2	7.9
457	FC(F)(F)CCCn1cc(c2ccccc21)C(=O)C(F)(F)F	7.9
458	FC(F)(F)CCCn1cc(c2ccccc21)C(=O)CCCC#C	7.9
459	FC(F)(F)CCCn1cc(c2ccccc21)C(=O)CCCC	7.8
460	FC(F)(F)CCCn1cc(C(=O)[C@@H]2CC[N+]2)c3ccccc31	7.8
461	FC(F)(F)CCCn1cc(c2ccccc21)C(=O)CCCC=C	7.8
462	FC(F)(F)CCCn1cc(c2ccccc21)C(=O)CSC	7.8
463	SCC(=O)c1c2ccccc2n(CCCC(F)(F)F)c1	7.8
464	FC(F)(F)CCCn1cc(c2ccccc21)C(=O)C=C3CCCCC3	7.8
465	FC(F)(F)CCCn1cc(c2ccccc21)C(=O)Cc3ccc[nH]3	7.8
466	FC(F)(F)CCCn1cc(c2ccccc21)C(=O)Cc3ccoc3	7.8
467	FC(F)(F)CCCn1cc(C(=O)[C@@H]([N+](CCC)C)C)c2ccccc21	7.7
468	FC(F)(F)CCCn1cc(C(=O)[C@H]([N+]C)C(C)C)c2ccccc21	7.7
469	FC(F)(F)CCCn1cc(c2ccccc21)C(=O)C=C3CCC(CC3)C	7.7
470	FC(F)(F)CCCn1c2ccccc2c(C(=O)C3(CC3)C(N)=[N+])c1	7.7
471	FC(F)(F)CCCn1cc(C(=O)[C@H]([N+])C2CCCC2)c3ccccc31	7.7
472	FC(F)(F)CCCn1cc(c2ccccc21)C(=O)[C@H]([N+])C(C)(C)C	7.7
473	FC(F)(F)CCCn1cc(c2ccccc21)C(=O)CSC(C)(C)C	7.7
474	FC(F)(F)CCCn1cc(C(=O)[C@@H]([N+](CC)C)C)c2ccccc21	7.7
475	FC(F)(F)CCCn1c2ccccc2c(C(=O)C3([N+])CC3)c1	7.6
476	FC(F)(F)CCCn1cc(C(=O)[C@H]2CSC[N+]2)c3ccccc31	7.6
477	FC(F)(F)CCCn1cc(c2ccccc21)C(=O)CCCC#N	7.6
478	FC(F)(F)CCCn1cc(c2ccccc21)C(=O)CSCC3CC3	7.6
479	FC(F)(F)CCCn1cc(c2ccccc21)C(=O)CCc3ccco3	7.6
480	FC(F)(F)CCCn1cc(c2ccccc21)C(=O)C[S@@](=O)C	7.6
481	FC(F)(F)CCCn1cc(c2ccccc21)C(=O)C[C@H](C)C#N	7.6
482	FC(F)(F)CCCn1cc(c2ccccc21)C(=O)CSCC	7.6
483	FC(F)(F)CCCn1cc(C(=O)[C@H]2[C@@H](CCC[N+]2)C)c3ccccc31	7.6

484	FC(F)(F)CCCn1cc(C(=O)[C@H](C[N+])CC)c2ccccc21	7.6
485	FC(F)(F)CCCn1cc(c2ccccc21)C(=O)CC(F)(F)F	7.6
486	FC(F)(F)CCCn1cc(C(=O)[C@@H]([N+](C)C)CC)c2ccccc21	7.5
487	FC(F)(F)CCCn1cc(c2ccccc21)C(=O)C[C@H](CC)C#N	7.5
488	FC(F)(F)CCCn1cc(c2ccccc21)C(=O)CCOC	7.5
489	FC(F)(F)CCCn1cc(C(=O)C[C@H](O)C(C)C)c2ccccc21	7.5
490	FC(F)(F)CCCn1cc(C(=O)[C@@H]2[C@H]([N+])C2)c3ccccc31	7.4
491	FC(F)(F)CCCn1cc(C(=O)[C@H]([N+])C(C)C)c2ccccc21	7.3
492	FC(F)(F)CCCn1cc(C(=O)C[C@@H](C(C)C)C[N+])c2ccccc21	7.2
493	FC(F)(F)CCCn1cc(c2ccccc21)C(=O)C[N+](C)(C)C	7.1
494	FC(F)(F)CCCn1cc(c2ccccc21)C(=O)CCCC[N+]	7
495	FC(F)(F)CCCn1cc(c2ccccc21)C(=O)CS(=O)(=O)C	7
496	FC(F)(F)CCCn1cc(C(=O)CS(=O)(=O)CC)c2ccccc21	7
497	FC(F)(F)CCCn1cc(C(=O)[C@H]([N+])CCC)c2ccccc21	7
498	FC(F)(F)CCCn1cc(C(=O)C[C@H]2CCC[N+]2)c3ccccc31	6.9
499	FC(F)(F)CCCn1cc(c2ccccc21)C(=O)N[C@@H](CC[N+])C	6.9
500	FC(F)(F)CCCn1cc(c2ccccc21)C(=O)CC[N+]	6.8
